# Supplementary material for: Regio- and enantioselective synthesis of acyclic quaternary carbons via organocatalytic addition of organoborates to (Z)-Enediketones
Source: Nat Commun. 2024 Jan 13;15:504. doi: 10.1038/s41467-024-44744-y (PMC10787796; doi:10.1038/s41467-024-44744-y)
Supplement: Supplementary file 1 — Supplementary Information [file 41467_2024_44744_MOESM1_ESM.pdf]

Supplementary Information

Regio- and Enantioselective Synthesis of Acyclic All-Carbon Quaternary Centers via Organocatalytic Nucleophilic Addition of Organoborates to (Z)-Enediketones

Po-Kai, Peng [a], Andrew Isho [a], and Jeremy, A. May[a,*]

[a.] *Department of Chemistry, University of Houston, 3585 Cullen Blvd., Fleming Room 112, Houston, TX 77204-5003, USA*

[*] *Email: jmay@uh.edu*

TABLE OF CONTENTS

General considerations: S–2

Synthesis of Potassium trifluoroborate salts. S–3

Synthesis of BINOL-based catalysts: S–4

General procedure for the synthesis of (Z)-endiketones: S–8

General procedure 1 (GP1) for the Synthesis of (E)-endiketones and/or (Z)-endiketones : S–8

General procedure 2 (GP2) for the synthesis of (Z)-endiketones: S–8

General procedure 3 (GP3) for the synthesis of (Z)-endiketones: S–8

Reaction Optimization S–14

Control experiments S–14

1. ***Catalyst effects*** S–14
2. ***Solvent effects*** S–15
3. ***Temperature effects*** S–15
4. ***Other conditions:*** S–16
5. ***Different boron sources:*** S–16

General procedure 4 (GP4) or the conjugate addition S–17

Transformations of 1,4-diones S–30

Crystal structure of compound 9 .... S–33

^{1}H and ^{13}C{1H} NMR Spectra S–38

HPLC Data S–147

Computional Details: S–180

General considerations:

All reactions were carried out in flame– or oven–dried glassware. THF, toluene, and CH_2Cl_2 (DCM) were purged with argon and dried over activated alumina columns. Flash chromatography was performed on 60Å silica gel (EMD Chemicals Inc). Preparative plate chromatography was performed on EMD silica gel plates, 60Å, with UV–254 indicator. Chemical names were generated using Cambridge Soft ChemBioDraw Ultra 12.0. Analysis by HPLC was performed on a Shimadzu Prominence LC (LC–20AB) equipped with a SPD–20A UV–Vis detector and a Chiralpak or Chiralcel (250 mm x 4.6 mm) column (see below for column details). Analytical thin layer chromatography was performed on EMD silica gel/TLC plates with fluorescent detector 254 nm. The ^{1}H, ^{13}C and ^{19}F NMR spectra were recorded on a JEOL ECA–600 II, ECA–500, or ECX–400P spectrometer using the residual solvent peak as an internal standard ($CDCl_3$: 7.26 ppm for ^{1}H NMR and 77.00 ppm for $^{13}C\{^{1}H\}$ NMR). NMR yields were determined by the addition of 0.5 equivalent of methyl (4–nitrophenyl) carboxylate as an internal standard to the crude reaction mixture. HRMS analyses were performed under contract by UT Austin's mass spectrometric facility with Q-TOF, G6546A, Q-TOF, G6530A, or 6890N Network gas chromatography system by using ESI and CI methods. X-ray analyses were conducted under contract by UH's X-ray facility under department of chemistry. Optical Rotation measurements were performed at AbbVie, North Chicago, using a Rudolph Research Analytical Automatic Polarimeter (Model APIV/6W) at 23 °C using a 1 dm cell. Measurements were recorded in triplicate, then averaged.

HPLC columns for separation of enantiomers:

Chiralpak AY–3: Amylose tris–(5–chloro–2–methylphenylcarbamate) coated on 3 μm silica gel
Chiralpak AD–H: Amylose tris–(3,5–dimethylphenylcarbamate) coated on 5 μm silica gel
Chiralpak ID: Amylose tris–(3–chlorophenylcarbamate) immobilized on 5 μm silica gel
Chiralcel OJ–H: Cellulose tris–(4–methylbenzoate) coated on 5 μm silica gel
Chiralcel OD–H: Cellulose tris–(3,5–dimethylphenylcarbamate) coated on 5 μm silica gel
Chiralpak AS–H: Amylose tris–[(*S*)–α–methylbenzylcarbamate) coated on 5 μm silica gel

Materials:

Commercially available compounds were purchased from Sigma Aldrich, BTC, Acros, TCI, Ambeed, and Alfa Aesar and were used without further purification.

Synthesis of Potassium trifluoroborate salts.

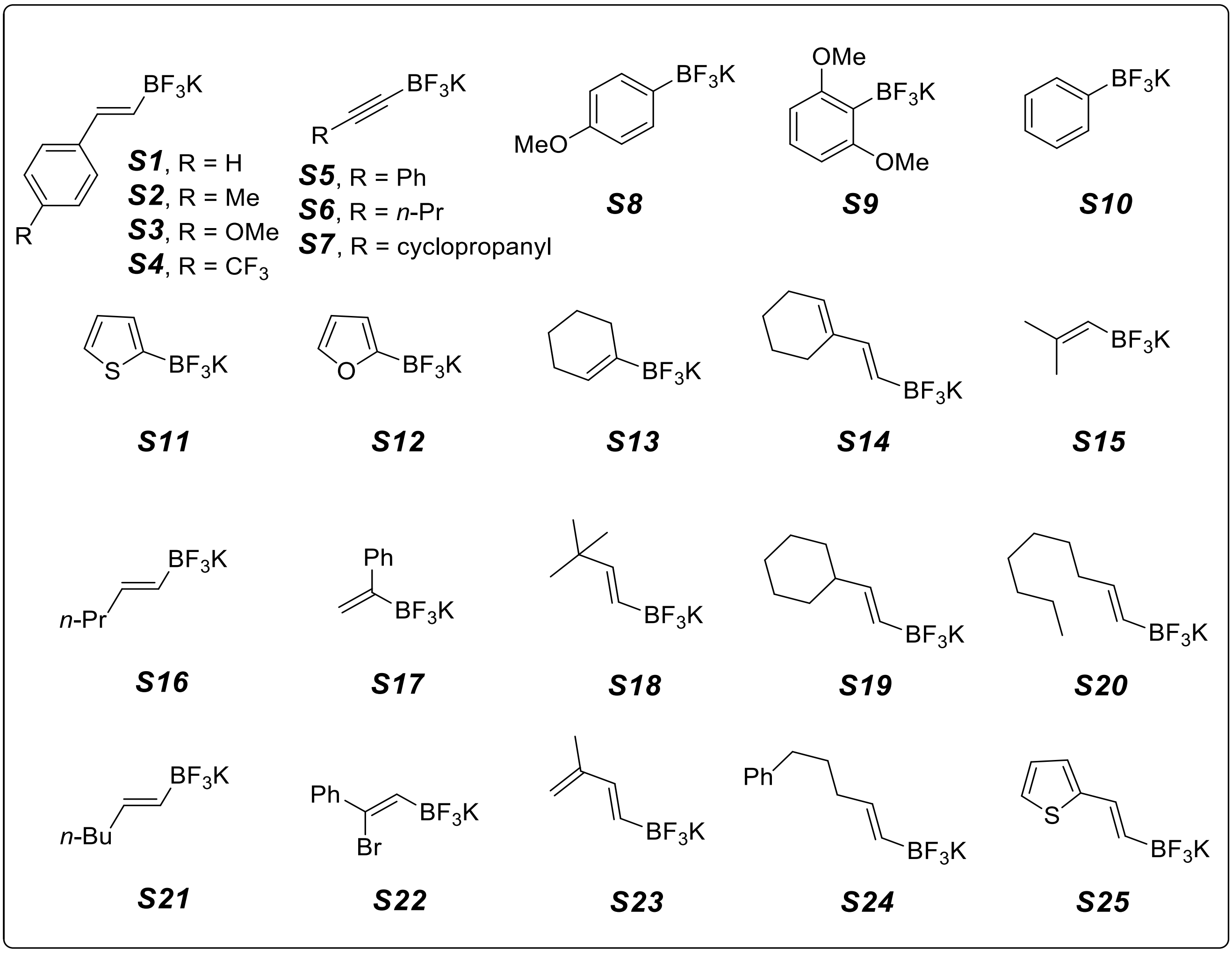

Supplementary Figure 1. Substrates of Potassium trifluoroborate salts.

Compounds **S1** to **S17**, were prepared by following these previously published procedures:

- For the synthesis of **S1**, **S3**, **S4**, **S15**, **S16**, **S19**, **S20**, **S21**, **S24**, see: Ellman, J. A.; Brak, K., *J. Am. Chem. Soc..* **2009**, 131, 3850.
- For the synthesis of **S5**, **S6**, **S10**, **S11**, **S12**, see: Sundstrom, S.; Nguyen, T. S.; May, J. A., *Org. Lett.* **2020**, 22, 1355.
- For the synthesis of **S13**, **S23**, **S25**, see: Liao, S.; Porta, A.; Cheng, X.; Ma, X.; Zanoni, G.; Zhang, L., *Angew. Chem. Int. Ed.* **2018**, *57*, 8250.
- For the synthesis of **S2**, **S7**, **S8**, **S9** see: Nguyen, T. N.; Setthakarn, K.; May, J. A., *Org. Lett.*, **2019**, *21*, 7837.
- For the synthesis of **S–14** see: Henderson, L.; Knight, D. W.; Rutkowski, P.; Williams, A. C., *Tetrahedron Lett.* **2012**, *53*, 4654.
- For the synthesis of **S15, S17, S18** see: Stefani, H. A.; Khan, A. N.; Manarin, F.; Vendramini, P. H.; Eberlin, M. N., *Tetrahedron Lett.* **2013**, *54*, 6204.
- For the synthesis of **S22**, see: Fang. X.; Sun, J.; Tong, X., *Chem. Comm.* **2010**, *46*, 3800.

Synthesis of BINOL-based catalysts:

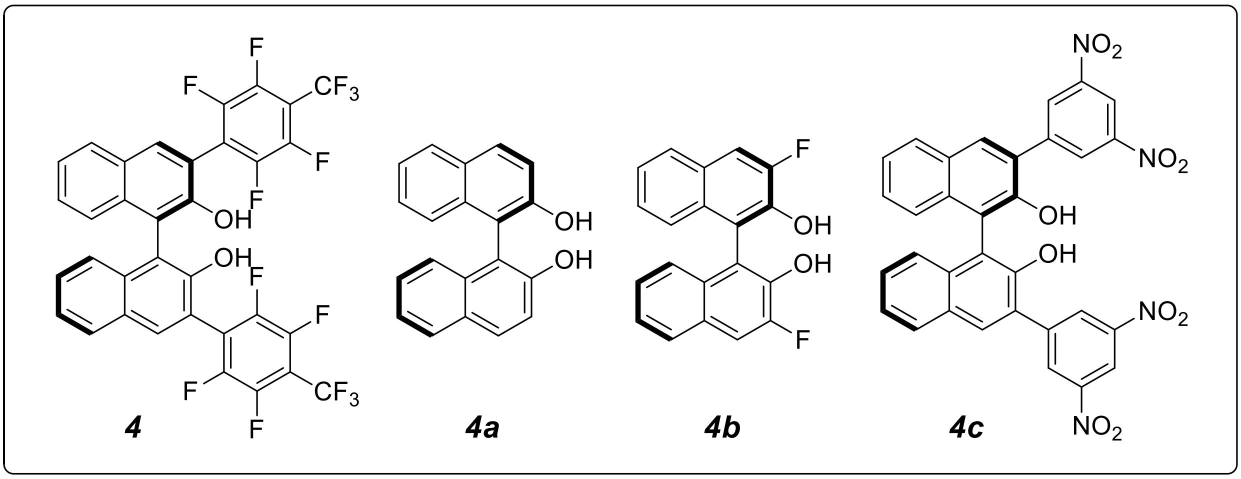

Supplementary Figure 2. Substrates of BINOL catalysts.

(*R*)–BINOL **4a** was purchased from BTC and used without further purification. Derivatives **4**, **4b–4c** were prepared by following previously published procedures. All spectral properties were identical to those reported in the literature.[1]

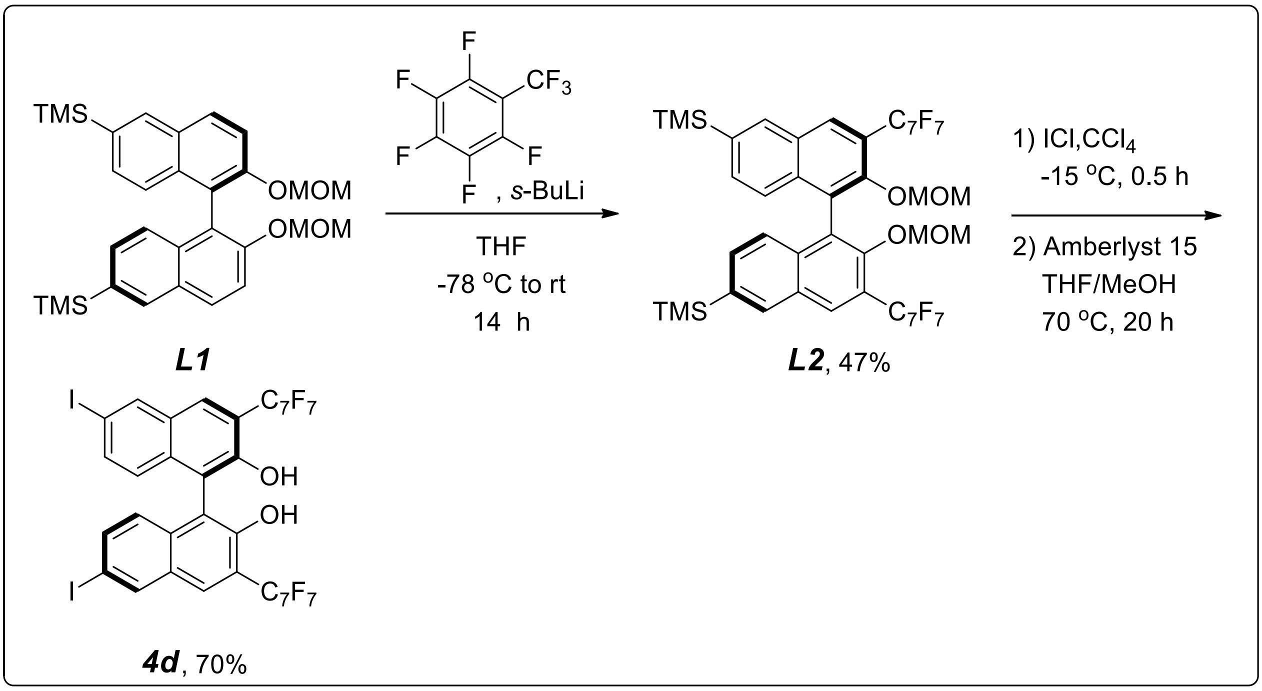

To a solution of (*R*)-2,2′-bis(methoxymethyloxy)-6,6′- bistrimethylsilyl-1,1′-binaphthyl[2] (**L1**, 554 mg, 1.07 mmol) in THF (30 mL) was added a solution of *s*-butyllithium (1.35 M, 3.5 mL) in cyclohexane dropwise at -78 °C. The reaction mixture was stirred for 0.5 h. Perfluorotoluene (1.1 mL) was then added at the same temperature. The resulting reaction mixture was allowed to stir at room

temperature for an additional 14 h. After the reaction completed, the mixture was quenched with NH_4Cl (aq). The two layers were extracted with ether, and the organic layers were collected and dried over $MgSO_4$. After filtration and concentration under reduced pressure, the crude product was purified by silica gel column chromatography (2.5% EtOAc in hexane) to afford the desired product **L2** in 47% yield as a white solid.

To a solution of **L2** (404 mg, 0.42 mmol) in anhydrous CCl_4 (0.2 M) was added iodine monochloride (ICl, 0.1 mL, 4.0 equiv) dropwise at -15 °C. The mixture was allowed to stir at the same temperature for 0.5 h. After the reaction completed, the mixture was quenched with $Na_2S_2O_3$ (aq). The two layers were extracted with ether, and the organic layers were collected and dried over $MgSO_4$. After filtration and concentration under reduced pressure, the crude product was redissolved in a mixture of methanol (15 mL) and THF (15 mL). Amberlyst–15 (2.0 g/mmol) was then added to the solution, and the resulting reaction mixture was allowed to stir at 70 °C for an additional 18 h. After the completion of the reaction, the solution was filtered and the solvent was removed under reduced pressure. The crude product was purified by silica gel column chromatography (2.0% EtOAc in hexane) to afford the desired product **4d** in 70% yield (282 mg, 0.29 mmol) as a yellow solid. **IR (neat)** 1477, 1331, 1144, 988 cm^{-1}; **^{1}H NMR** (400 MHz, $CDCl_3$) δ 8.36 (d, J = 2.0 Hz, 2H), 7.95 (s, 2H), 7.69 (d, J = 8.5, 1.5 Hz, 2H), 6.94 (d, J = 8.5 Hz, 2H), 5.34 (s, 2H); **^{13}C{^{1}H} NMR** (100 MHz, $CDCl_3$) δ 150.4, 145.6 (m), 143.0 (m), 137.8, 137.6, 133.0, 132.6, 130.4, 125.5, 120.8 (-**C**F_3, q, $^1J_{C\text{–}F}$= 272 Hz), 120.3 (m), 116.5, 111.2, 109.4, 90.4; **^{19}F NMR** (375 MHz, $CDCl_3$) δ -56.11 (-C**F**$_3$, t, J = 21.6 Hz, 6F), -137.58 (dd, J = 12.4, 22.1 Hz, 2F), -138.00 (dd, J = 12.3, 22.1 Hz, 2F), -140.2–-140.5 (m, 4F); **HRMS (ESI)** m/z Calculated for $C_{34}H_{10}F_{14}I_2O_2$ $[M\text{-}H]^+$: 968.8474; found: 968.8467. **Rf**: 0.43 (EtOAc : hexane = 1 : 10).

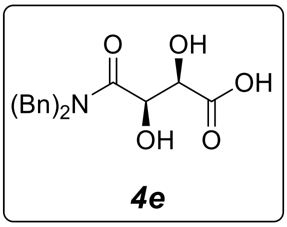

4e was prepared by following previously published procedures.[3]

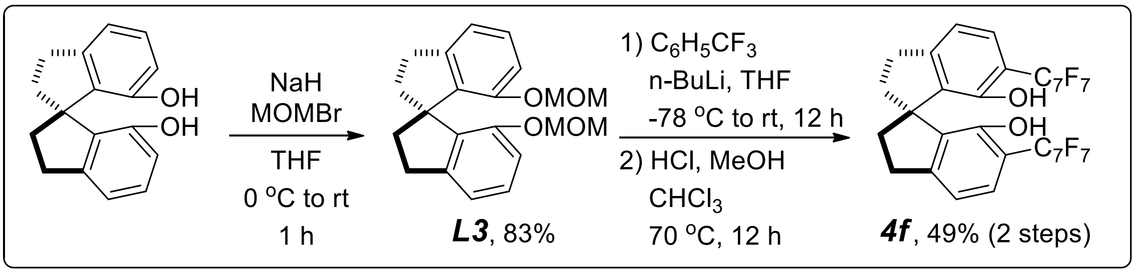

Derivatives **L3** and **4f** was prepared by following previously published procedures.[4]

To a flame-dried flask equipped with a stir-bar and an addition funnel was added NaH (60% dispersion in mineral oil, 593 mg, 14.8 mmol, 6.0 equiv) and THF (15 mL) at 0 °C. (*S*)-SPINOL (623

mg, 2.47 mmol, 1.0 equiv) was then added to the above suspension in one portion. The reaction mixture was allowed to stir at 0 °C for 2 h. MOM-Br (0.63 mL, 7.68 mmol, 2.2 equiv) was then added dropwise. The reaction was allowed to stir at 0 °C for 10 min. After completion, the reaction mixture was quenched with saturated aq. NH_4Cl, extracted with Et_2O, and washed with brine. The organic layer was dried with $MgSO_4$, and the solvent was removed via rotary evaporation. The crude product was purified by silica gel column chromatography (10% EtOAc in hexane) to afford the desired product **L3** in 83% yield (694 mg, 2.03 mmol) as white solid. All spectral properties were identical to those reported in the literature.

To a flame-dried flask equipped with a magnetic stir bar was added **L3** (694 mg, 2.03 mmol, 1 equiv) and THF (14 mL). The reaction mixture was then cooled to 0 °C, had the addition of 2.5 M *n*-BuLi (3.6 ml, 8.88 mmol, 3.0 equiv), and was allowed to stir for 2 h. The reaction temperature was decreased to -78 °C and perfluorotoluene (2.9 ml, 20.72 mmol, 7 equiv) was added dropwise via syringe. The reaction mixture was then warmed up to ambient temperature and stirred at this temperature for 12h. After completion, the reaction was quenched with saturated $NH_4Cl_{(aq)}$, extracted with Et_2O, and washed with brine. After the removal of solvents via rotary evaporation, the crude product mixture was dissolved in $CHCl_3$ (6 mL) for the next step without further purification.

To a solution of MOM–protected perfluorotoluyl–SPINOL in $CHCl_3$ (6 mL) was added conc. HCl (4.0 mL) and MeOH (4.0 mL) at ambient temperature. The reaction mixture was allowed to stir at 70 °C for 5 h. After the reaction completed, it was quenched with saturated $NaHCO_{3(aq)}$, extracted with Et_2O, and wash with brine. The organic layer was collected, and then the solvent was removed under reduced pressure. The crude product was purified via flash column chromatography with appropriate eluents (EtOAc : hexane = 1 : 10) to give the product **4f** as a white solid (682 mg, 0.996 mmol) in 49% yield (2 steps)

IR (neat) 1482, 1337, 1144 cm^{-1}; **^{1}H NMR** (400 MHz, $CDCl_3$) δ 7.19 (d, *J* = 2.0 Hz, 2H), 7.04 (d, *J* = 2.0 Hz, 2H), 4.83 (s, 2H), 3.16–3.08 (m, 4H), 2.48–2.43 (m, 2H), 2.31–2.23 (m, 2H); **^{13}C{^{1}H} NMR** (100 MHz, $CDCl_3$) δ 150.5, 148.7, 145.7–145.5, 143.3–142.7,132.2, 130.4,, 121.3 (m), 120.9 (-**C**F_3, q, $^1J_{C\text{–}F}$= 274 Hz), 118.3, 112.3, 108.5 (m), 57.9, 37.4, 31.3; **^{19}F NMR** (375 MHz, $CDCl_3$) δ -56.12 (-C**F**$_3$, t, *J* = 22.5 Hz, 6F), -137.4–-138.5 (m, 2F), -138.23 (dd, *J* = 15.0, 22.5 Hz, 2F), -140.9–-141.1 (m, 2F), -141.3–-141.6 (m, 2F); **HRMS (ESI)** m/z Calculated for $C_{34}H_{10}F_{14}I_2O_2$ $[M\text{-}H]^+$: 968.8474; found: 968.8467. **Rf**: 0.43 (EtOAc : hexane = 1 : 10).

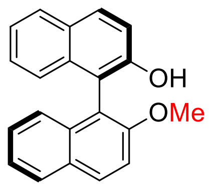

(*R*)–monomethylated BINOL **4g** was prepared by following previously published procedures. All spectral properties were identical to those reported in the literature.[5]

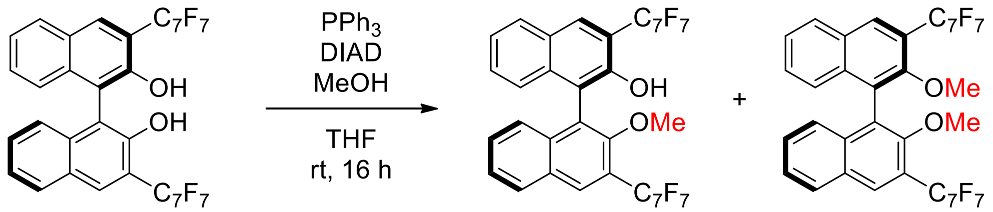

(*R*)–monomethylated–3,3′–$(C_7F_7)_2$–BINOL **4h** was prepared by following previously published procedures.[6]

To a solution of **4** (502 mg, 0.7 mmol, 1.0 equiv) and triphenyl phosphine (PPh_3, 217 mg, 1.1 equiv) in THF (12 mL, 0.3 M) was added MeOH (50 μL, 1.5 equiv) and DIAD (0.47 mL, 1.09 equiv) at room temperature, and the resulting mixture was allowed to stir at the same temperature for 16 h. The reaction was monitored with TLC. After completion of the reaction, the solvent was removed under reduced pressure and the crude product was purified by silica gel chromatography (DCM : hexane = 1 : 8 to 1 : 5) to give the methylated product **4h** (212 mg, 0.289 mmol) as white solid in 41% yield and dimethylated BINOL in 29% yield.

IR (neat) 1480, 1339, 1141 cm^{-1}; **^{1}H NMR** (500 MHz, $CDCl_3$) δ 8.05 (s, 1H), 8.00–7.93 (m, 3H), 7.56 (t, *J* = 10.0 Hz, 1H), 7.48–7.40 (m, 3H), 7.36 (d, *J* = 10.0 Hz, 1H), 7.25–7.23 (m, 1H), 5.30 (-O**H**, s, 1H), 3.25 (-C**H**$_3$, s, 3H); **^{13}C{^{1}H} NMR** (150 MHz, $CDCl_3$) δ 154.8, 148.6, 145.2 (t, *J* = 76.9 Hz), 143.5 (d, *J* = 83.0 Hz), 134.5, 134.2, 133.5, 132.7, 130.4, 128.9–128.5, 126.4, 125.0, 124.8, 124.7, 121.8–121.7, 120.3, 120.1, 115.7, 115.3, 109.2, 61.4; **^{19}F NMR** (375 MHz, $CDCl_3$) δ -56.1 (-C**F**$_3$, t, *J* = 22.5 Hz, 6F), -137.8–-138.2 (m, 4F), -141.0–-141.2 (m, 2F), -140.4–-140.6 (m, 2F); **HRMS (ESI)** m/z Calculated for $C_{35}H_{14}F_{14}O_2$ $[M+Na]^+$: 755.0662; found: 755.0649.

General procedure for the synthesis of (Z)-endiketones:

General procedure 1 (GP1) for the Synthesis of (E)-endiketones and/or (Z)-endiketones :

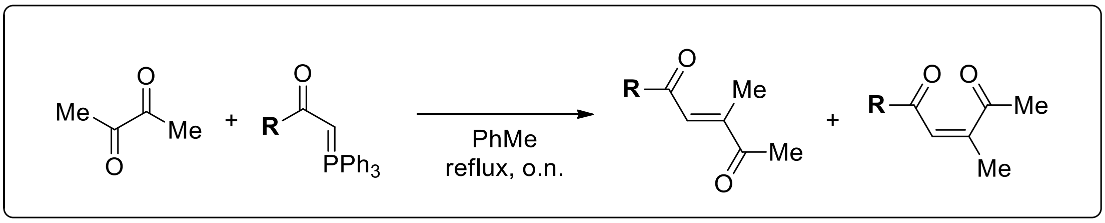

To a solution of the corresponding phosphorane (1.0 equiv) in anhydrous toluene (0.2 M) was added diketone (1.0 equiv) dropwise at room temperature. The mixture was allowed to stir at 110 ºC for 24 h. After the reaction completed, the solvent was removed, and the crude product was purified by column chromatography (v/v: (EtOAc: hexane = 1 : 10) to give *E*–isomer of product and the Z–isomer product. (Note: Some substrates only generated the *E* isomer.)

General procedure 2 (GP2) for the synthesis of (Z)-endiketones:

To a solution of corresponding phosphorane (1.0 equiv) in anhydrous toluene (0.2 M) was added diketone (1.0 equiv) dropwise at room temperature. The mixture was allowed to stir at 110 ºC for 24 h. After the reaction completed, the solvent was removed, and the crude product was purified by column chromatography (v/v: (EtOAc: hexane = 1 : 10) to give *E*–isomer of product, which is directly redissolved in toluene. The solution was then irradiated with 20 W CFL for another 24 h. After the reaction completed, the solvent was removed and the crude product was purified by column chromatography (v/v: (EtOAc: hexane = 1 : 10) to afford the Z–isomer.

General procedure 3 (GP3) for the synthesis of (Z)-endiketones:

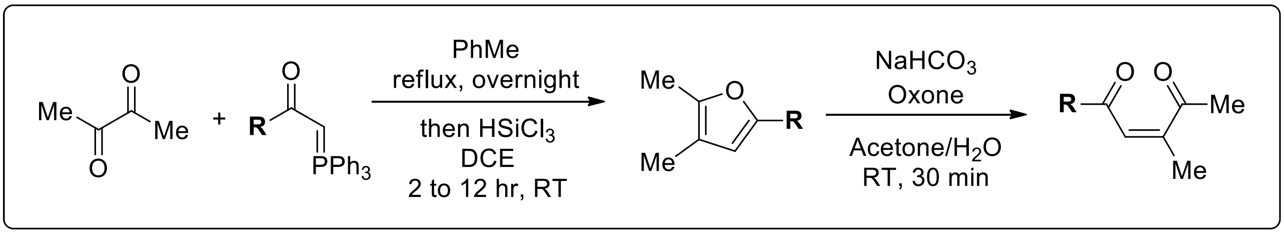

Representative Synthesis of corresponding furan:[7]

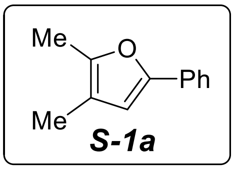

Furans were synthesized following a procedure published by Zhou and coworkers [7] using butanedione (0.5 mL, 1.0 equiv), the corresponding phosphorane (2.08 g, 1.0 equiv,), trichlorosilane (1.1 mL, 2.0 equiv). Purification was performed via flash column chromatography with appropriate eluents (EtOAc : hexane = 1 : 10) to give the product **S–1a** as a white solid (629 mg, 3.65 mmol) in 67% yield. **^{1}H NMR** (400 MHz, $CDCl_3$) δ 7.62 (d, *J* = 8.0 Hz, 2H), 7.36 (t, *J* = 8.0 Hz, 2H), 7.21 (t, *J* = 8.0 Hz,

1H), 6.46 (s, 1H), 2.29 (s, 3H), 2.00 (s, 3H); **$^{13}C\{^1H\}$ NMR** (100 MHz, $CDCl_3$) δ 150.8, 147.3, 131.2, 128.5, 126.5, 123.1, 116.1, 108.3, 11.5, 9.9. All spectral properties were identical to those reported in the literature.[7]

Synthesis of (Z)-endiketones

To a round-bottomed flask equipped with a magnetic stir bar was added the furan (1.0 equiv), acetone (6.4 mL/mmol), $NaHCO_{3(aq)}$ (3.2 mL/mmol), water (1.6 mL/mmol), and oxone (2.0 equiv) in sequence at ambient temperature. The reaction mixture was allowed to stir another 0.5 h to 1 h. After the reaction completed, the mixture was quenched with $NH_4Cl_{(aq)}$ and washed with ethyl acetate, the combined organic layer was then dried over $MgSO_4$, the solvent was removed under reduced pressure, and the crude product was purified via flash column chromatography on silica gel with appropriate eluents to give the corresponding (*Z*)-endiketone.

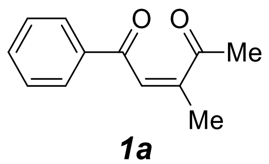

1a

Following GP3, the enediketone **1a** was prepared from (benzoylmethylene)triphenylphosphorane (5.0 g, 13.1 mmol), 2,3-butanedione (1.3 mL), $HSiCl_3$ (2.7 mL), acetone (30 mL), $NaHCO_{3(aq)}$ (20 mL), water (5 mL), and oxone (5.5 g). Purification was accomplished via flash column chromatography with appropriate eluents (EtOAc : hexane = 1 : 3.5) to give the product **1a** as a white solid (1.29 g, 6.85 mmol) in 52% yield (2 steps). **IR (neat)** 1694, 1658, 1602, 1576. 1449, cm^{-1}; **^1H NMR** (600 MHz, $CDCl_3$) δ 7.94 (d, *J* = 6.0 Hz, 2H), 7.58 (t, *J* = 6.0 Hz, 1H),7.47 (d, *J* = 6.0 Hz, 2H), 6.82 (s, 1H), 2.36 (s, 3H), 2.11 (s, 3H); **$^{13}C\{^1H\}$ NMR** (150 MHz, $CDCl_3$) δ 206.8, 189.2, 157.3, 136.8, 133.3, 128.7, 128.5, 121.5, 28.2, 20.7; **HRMS (ESI)** m/z Calculated for $C_{12}H_{12}O_2$ $[M+Na]^+$: 211.0730; found: 211.0733. **Rf**: 0.33 (EtOAc : hexane = 1 : 3).

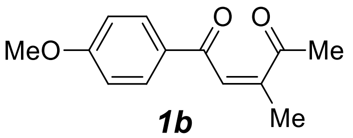

1b

Following GP3, the enediketone **1b** was prepared from the corresponding phosphorane (1.08 g), 2,3-butanedione (0.2 mL), $HSiCl_3$ (0.5 mL), acetone (4.0 mL), $NaHCO_{3(aq)}$ (1.8 mL), water (0.9 mL), and oxone (335 mg). Purification was accomplished via flash column chromatography with appropriate eluents (EtOAc : hexane = 1 : 3) to give the product **1b** (56 mg, 0.26 mmol) as a white solid in 11% (2 steps). **IR (neat)** 1669, 1652, 1597, 1511, 1255 cm^{-1} ; **^1H NMR** (400 MHz, $CDCl_3$) δ 7.92 (d, *J* = 8.0 Hz, 2H), 6.92 (d, *J* = 8.0 Hz, 2H), 6.77 (s, 1H), 3.85 (s, 3H), 2.33 (s, 3H), 2.07 (s, 3H); **$^{13}C\{^1H\}$ NMR** (100 MHz, $CDCl_3$) δ 207.0, 187.6, 163.7, 156.3, 130.8, 129.7, 121.5, 113.8, 55.4, 28.2, 20.5; **HRMS (ESI)** m/z Calculated for $C_{13}H_{14}O_3$ $[M+H]^+$: 219.1016; found: 219.1020. **Rf**: 0.2 (EtOAc : hexane = 1 : 3).

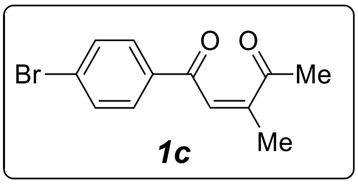

Following GP3, the enediketone **1c** was prepared from the corresponding phosphorane (1.0 g), 2,3-butanedione (0.2 mL), $HSiCl_3$ (0.4 mL), acetone (6.0 mL), $NaHCO_{3(aq)}$ (3.0 mL), water (1.5 mL), and oxone (538 mg). Purification was accomplished via flash column chromatography with appropriate eluents (EtOAc : hexane = 1 : 3) to give the product **1c** as a white solid (175 mg, 0.65 mmol) in 34% (2 steps). **IR (neat)** 1739, 1696, 1663, 1614. 1580, 1434 cm^{-1}; **^{1}H NMR** (400 MHz, $CDCl_3$) δ 7.75 (d, J = 8.0 Hz, 2H), 7.55 (d, J = 8.0 Hz, 2H), 6.72 (s, 1H), 2.31 (s, 3H), 2.07 (s, 3H); **^{13}C{^{1}H} NMR** (100 MHz, $CDCl_3$) δ 206.4, 188.0, 157.7, 135.3, 131.8, 129.8, 128.4, 120.9, 28.0, 20.5; **HRMS (ESI)** m/z Calculated for $C_{12}H_{11}BrO_2$ $[M+H]^+$: 267.0015; found: 267.0009. **Rf**: 0.60 (EtOAc : hexane = 1 : 3).

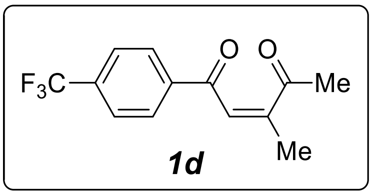

Following GP3, the enediketone **1d** was prepared from the corresponding phosphorane (1.08 g), 2,3-butanedione (0.2 mL), $HSiCl_3$ (0.5 mL), acetone (6.0 mL), $NaHCO_{3(aq)}$ (3.0 mL), water (1.5 mL), and oxone (593 mg). Purification was accomplished via flash column chromatography with appropriate eluents (EtOAc : hexane = 1 : 3.5) to give the product **1d** (191 mg, 0.75 mmol) as a white solid in 33% (2 steps). **IR (neat)** 1697, 1664, 1602, 1324 cm^{-1} ; **^{1}H NMR** (400 MHz, $CDCl_3$) δ 8.01 (d, J = 8.0 Hz, 2H), 7.70 (d, J = 8.0 Hz, 2H), 6.77 (s, 1H), 2.34 (s, 3H), 2.11 (s, 3H); **^{13}C{^{1}H} NMR** (100 MHz, $CDCl_3$) δ 206.3, 188.2, 158.4, 139.4, 134.3 (q, $^2J_{C-F}$= 33 Hz), 128.7, 125.6 (d, $^3J_{C-F}$ = 3.0 Hz), 123.4 (q, $^1J_{C-F}$= 272 Hz), 27.9, 20.7; **^{19}F NMR** (375 MHz, $CDCl_3$) δ -63.03 ($-CF_3$); **HRMS (ESI)** m/z Calculated for $C_{13}H_{11}F_3O_2$ [M+H]+: 257.0784; found: 257.0782. **Rf**: 0.39 (EtOAc : hexane = 1 : 3).

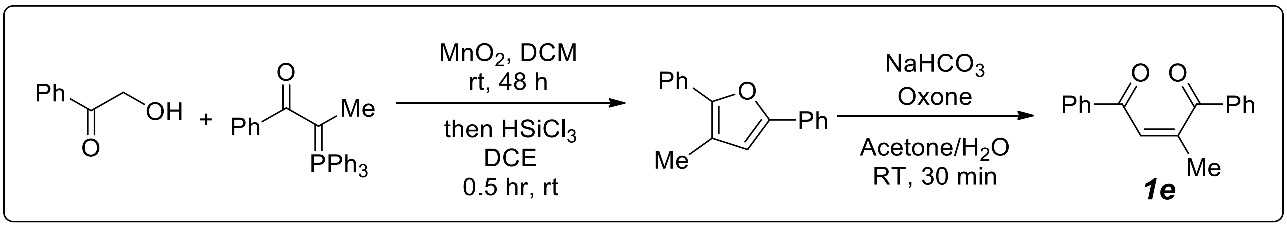

To a round-bottomed flask was added appropriate phosphorane (1.01 g, 2.55 mmol, 1.0 equiv), 2-hydroxy-1-phenylethanone (770 mg, 2.2 equiv), and dichloromethane (13 mL, 0.2 M) at the ambient temperature. The resulting mixture was allowed to stir at the same temperature for another 48 h. After the reaction completed, the solvent was removed under reduced pressure and dissolved in DCE (12 mL) for the next step without further purification. To the above solution was then added trichlorosilane ($HSiCl_3$, 0.5 mL) at the room temperature. The mixture was stirred at rt for 0.5 h. After completion of the reaction, the solvent was removed under reduced pressure and the crude product was purified via flash column chromatography with appropriate eluents (EtOAc : hexane = 1 : 15) to give the furan derivative (340 mg, 1.45 mmol) as a white solid in 56% yield. All spectral properties were identical to

those reported in the literature.[7]

To a round-bottomed flask equipped with a magnetic stir bar was added furan derivatives (300 mg, 1.28 mmol, 1.0 equiv), acetone (8.0 mL/mmol), $NaHCO_{3(aq)}$ (4.0 mL/mmol), water (2.0 mL/mmol), and oxone (788 mg, 2.0 equiv) subsequently at ambient temperature. The reaction mixture was allowed to stir another 0.5 h. After the reaction completed, the mixture was quenched with $NH_4Cl_{(aq)}$ and washed with ethyl acetate, the combined organic layer was then dried over $MgSO_4$, and the solvent was removed under reduced pressure. Purification was accomplished via flash column chromatography with appropriate eluents (EtOAc : hexane = 1 : 3) to give the product **1e** (267 mg, 0.19 mmol) as a white solid in 83% yield. **IR (neat)** 1658, 1602, 1448, 1223 cm^{-1} ; **^{1}H NMR** (500 MHz, $CDCl_3$) δ 7.93–7.91 (m, 4H), 7.57–7.54 (m, 2H), 7.47–7.43 (m, 4H), 7.16 (d, $^4J_{Me-H}$ = 1.5 Hz, 1H), 2.25 (d, $^4J_{Me-H}$ = 1.5 Hz, 3H); **^{13}C{^{1}H} NMR** (100 MHz, $CDCl_3$) δ 199.4, 188.0, 156.4, 136.8, 134.4, 133.3, 133.2, 128.8, 128.6, 128.5, 128.3, 122.8, 22.2; **HRMS (ESI)** m/z Calculated for $C_{17}H_{14}O_2$ $[M+H]^+$: 251.1067; found: 251.1070. **Rf**: 0.44 (EtOAc : hexane = 1 : 3).

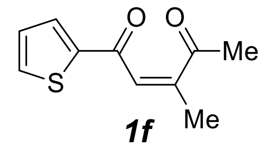

1f

Following GP3, the enediketone **1f** was prepared from the corresponding phosphorane (810 mg, 2.1 mmol), 2,3-butanedione (0.2 mL), $HSiCl_3$ (0.4 mL), acetone (5.5 mL), $NaHCO_{3(aq)}$ (2.7 mL), water (1.4 mL), and oxone (520 mg). Purification was accomplished via flash column chromatography with appropriate eluents (EtOAc : hexane = 1 : 4) to give title product **1f** (56 mg, 0.19 mmol) as a white solid in 23% yield (2 steps). **IR (neat)** 1690, 1646, 1598, 1414, 1272 cm^{-1}; **^{1}H NMR** (400 MHz, $CDCl_3$) δ 7.74 (d, J = 4.0 Hz, 1H), 7.66 (d, J = 4.0 Hz, 1H), 7.14 (t, J = 4.0 Hz, 1H), 6.64 (s, 1H), 2.36 (s, 3H), 2.08 (s, 3H); **^{13}C{^{1}H} NMR** (100 MHz, $CDCl_3$) δ 206.8, 181.1, 157.6, 144.1, 134.7, 132.4, 128.4, 120.6, 28.2, 20.6; **HRMS (ESI)** m/z Calculated for $C_{10}H_{10}O_2S$ $[M+Na]^+$: 217.0294; found: 217.0294. **Rf**: 0.32 (EtOAc : hexane = 1 : 3).

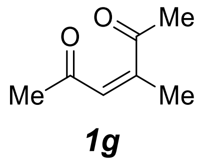

1g

Following GP1, the enediketone **1g** was prepared in 10 % as colorless liquid from (acetylmethylene)triphenylphosphorane, 2,3-butanedione (1.2 mL) in toluene (2.7 mL). All spectral properties were identical to those reported in the literature.[8]

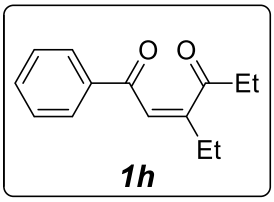

Following GP3, the enediketone **1h** was prepared from (benzoylmethylene)triphenylphosphorane (2.0 g, 5.25 mmol), 3,4-hexanedione (1.6 mL), $HSiCl_3$ (1.0 mL), acetone (8.0 mL), $NaHCO_{3(aq)}$ (4.0 mL), water (2.0 mL), and oxone (922 mg). Purification was accomplished via flash column chromatography with appropriate eluents (EtOAc : hexane = 1 : 6) to give the product **1h** (105 mg, 0.48 mmol) as a white solid in 11% (2 steps). **IR (neat)** 1738, 1699, 1365, 1217 cm^{-1}; **^{1}H NMR** (600 MHz, $CDCl_3$) δ 7.96–7.93 (m, 2H), 7.58–7.46 (m, 3H), 6.80 (s, 1H), 2.60–2.41 (m, 4H), 1.23–1.18 (m, 6H); **^{13}C{^{1}H} NMR** (100 MHz, $CDCl_3$) δ 209.9, 189.2, 163.8, 137.2, 133.2, 128.7, 128.3, 119.3, 34.7, 27.8, 11.4, 7.7; **HRMS (ESI)** m/z Calculated for $C_{14}H_{16}O_2$ $[M+Na]^+$: 239.1043; found: 239.1047. **Rf**: 0.55 (EtOAc : hexane = 1 : 3).

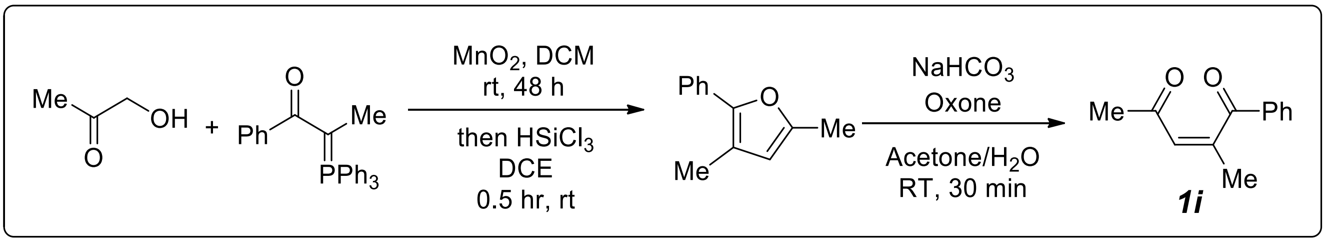

Following the procedure from synthesis of **1e**, the enediketone **1i** was prepared from the corresponding phosphorane (1.0 g, 2.53 mmol), 1-hydroxy-2-propanone (0.5 mL, 3.0 equiv), $HSiCl_3$ (0.5 mL), acetone (9.6 mL), $NaHCO_{3(aq)}$ (5.0 mL), water (2.4 mL), and oxone (922 mg). Purification was accomplished via flash column chromatography with appropriate eluents (EtOAc : hexane = 1 : 2) to give the product **1i** (240 mg, 1.27 mmol) as a white solid in 50% (2 steps) **IR (neat)** 1691, 1664, 1614, 1449, 1362 cm^{-1}; **^{1}H NMR** (600 MHz, $CDCl_3$) δ 7.88–7.85 (m, 2H), 7.57–7.54 (m, 1H), 7.47–7.45 (m, 2H), 6.38 (s, 1H), 2.16 (s, 3H), 2.12 (s, 3H); **^{13}C{^{1}H} NMR** (150 MHz, $CDCl_3$) δ 199.2, 195.9, 154.1, 134.4, 133.3, 128.7, 128.3, 126.4, 29.9, 21.6; **HRMS (ESI)** m/z Calculated for $C_{12}H_{12}O_2$ $[M+H]^+$: 189.0910; found: 189.0911. **Rf**: 0.30 (EtOAc : hexane = 1 : 3).

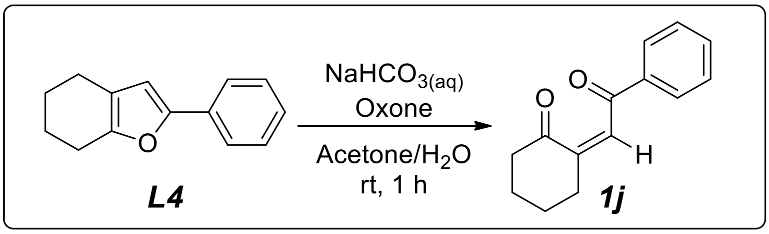

The corresponding furan **L4** was prepared by the previously published procedures.[9]

To a round-bottomed flask equipped with a magnetic stir bar was added furan derivatives **L4** (198mg, 1.0 mmol, 1.0 equiv), acetone (6.4 mL/mmol), $NaHCO_{3(aq)}$ (3.2 mL/mmol), water (1.6 mL/mmol), and oxone (615 mg, 2.0 equiv) subsequently at ambient temperature. The reaction mixture was allowed to stir for another 1 h. After the reaction completed, the mixture was quenched with $NH_4Cl_{(aq)}$ and washed

with ethyl acetate (10 mL, 3 times), the combined organic layer was then dried over $MgSO_4$, the solvent was removed under reduced pressure, the crude product was purified via flash column chromatography on silica gel with appropriate eluents (EtOAc : hexane = 1 : 6 to 1 : 1) to give the corresponding (*Z*)-endiketone **1j** (153 mg, 0.714 mmol) in 71% yield as white solid. **IR (neat)** 2937, 1702, 1661, 1620, 1445, 1368 cm^{-1}; **^{1}H NMR** (500 MHz, $CDCl_3$) δ 7.91 (d, *J* = 10.0 Hz, 2H), 7.55 (t. *J* = 10.0 Hz, 1H), 7.45 (t, *J* = 10.0 Hz, 2H), 6.49 (s, 1H), 2.71 (t. *J* = 5.0 Hz, 2H), 2.58 (t. *J* = 5.0 Hz, 2H) 1.99–1.90 (m, 4H); **^{13}C{^{1}H} NMR** (150 MHz, $CDCl_3$) δ 203.8, 192.9, 150.9, 136.5, 133.2, 128.5, 127.8, 42.9, 36.0, 26.0, 25.8; **HRMS (ESI)** m/z Calculated for $C_{14}H_{14}O_2$ $[M+H]^+$: 215.1067; found: 215.1069. **Rf**: 0.16 (EtOAc : hexane = 1 : 3).

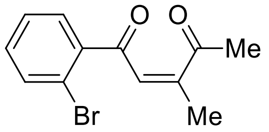

1k

Following GP2, the enediketone **1k** was prepared from the corresponding phosphorane (1.05 g, 2.3 mmol), 2,3-butanedione (0.2 mL). Purification was accomplished via flash column chromatography with appropriate eluents (EtOAc : hexane = 1 : 6) to give title product **1k** (145 mg, 0.19 mmol) as a white solid in 24% yield. **IR (neat)** 1738, 1700, 1365 cm^{-1}; **^{1}H NMR** (400 MHz, $CDCl_3$) δ 7.61 (d, *J* = 8.0 Hz, 1H), 7.46 (d, *J* = 8.0 Hz, 1H), 7.40–7.29 (m, 2H), 6.54 (s, 1H), 2.37 (s, 3H), 2.07 (s, 3H); **^{13}C{^{1}H} NMR** (100 MHz, $CDCl_3$) δ 206.6, 191.5, 156.9, 140.1, 133.5, 132.2, 129.8, 127.5, 124.2, 119.5, 28.0, 20.6; **HRMS (ESI)** m/z Calculated for $C_{12}H_{11}BrO_2$ $[M+Na]^+$: 288.9835; found: 288.9843. **Rf**: 0.42 (EtOAc : hexane = 1 : 3).

Reaction Optimization

Control experiments

1. Catalyst effects

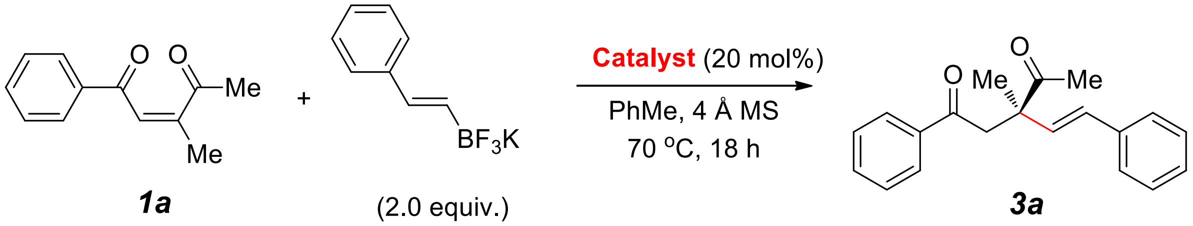

1a **3a**

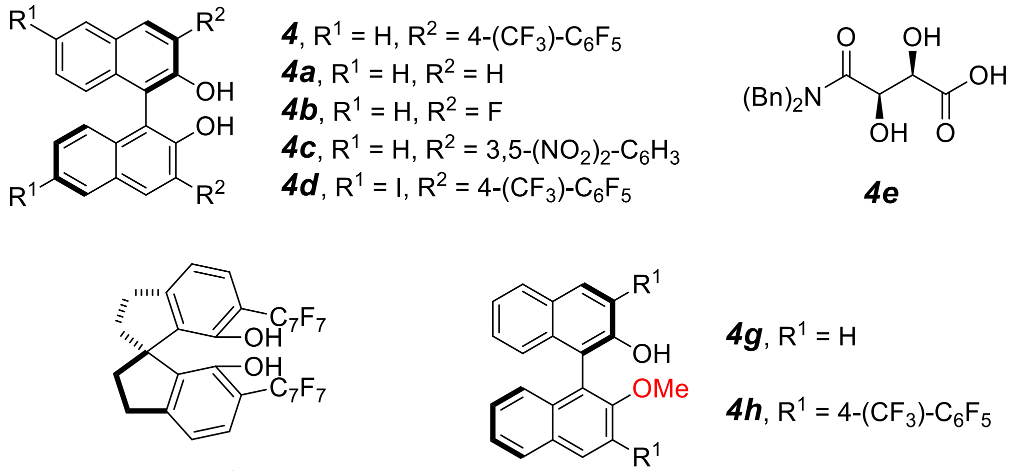

4e **4f**

entry	catalyst	Yield (%)[a]	er [b]
**1**	**-**	69	50:50
**2**	**4**	99	96:04
**3**	**4a**	85	75:25
**4**	**4b**	87	89:11
**5**	**4c**	99	90:10
**6**	**4d**	99	94:06
**7**	**4e**	91	50:50
**8**	**4f**	99	50:50
**9**	**4g**	56	58:42
**10**	**4h**	85	53:47

[a] Isolated yield. [b] Determined by HPLC with chiral stationary phase.

Supplementary Table 1. Evaluation of BINOL catalysts.

2. *Solvent effects*

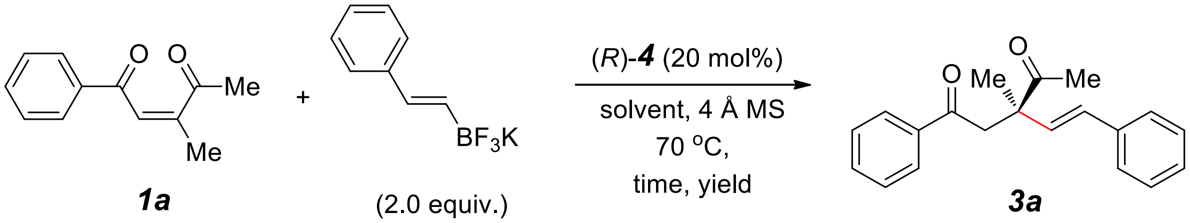

entry [a]	solvent	time (h)	yield (%) [b]	er [c]
**1**	PhMe	18	99	96:04
**2**	DCE	18	97	85:15
**3**	ACN	18	99	59:41
**4**	cyclohexane	18	88	93:07
**5**	EtOAc	11	90	69:31
**6**	EtOH	17	77	65:35
**7**	$PhCF_3$	4	95	95:05

[a] All reactions were conducted with 0.21 mmol starting material at 0.2 M. [b] Isolated yield. [c] Determined by HPLC with chiral stationary phase.

Supplementary Table 2. Evaluation of solvents.

3. *Temperature effects*

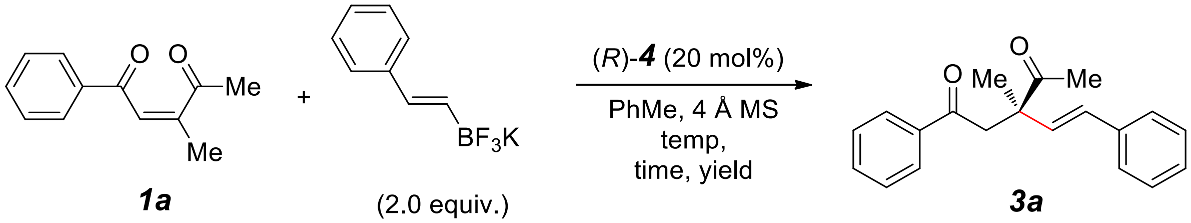

entry [a]	temp. (°C)	time (h)	yield (%) [b]	er [c]
**1**	20	88	80	95:05
**2** [d]	50	24	64	95:05
**3**	70	18	97	96:04
**4**	90	3	80	94:06
**5** [e]	145	0.5	77	93:07

[a] All reactions were conducted with 0.21 mmol starting material at 0.2 M. [b] Isolated yield. [c] Determined by HPLC with chiral stationary phase. [d] Reaction did not complete. [e] Performed in sealed tube.

Supplementary Table 3. Evaluation of temperature.

4. *Other conditions:*

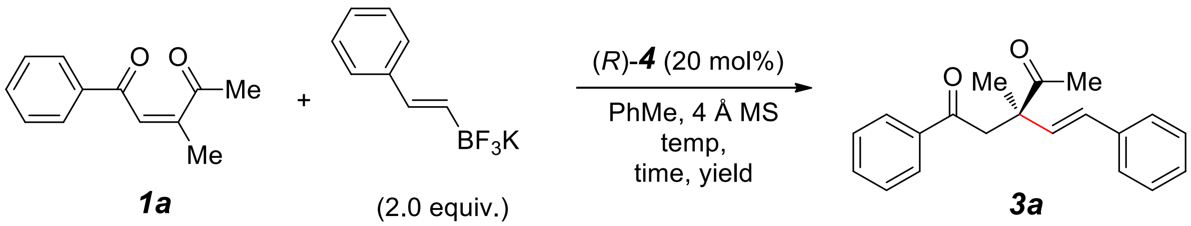

entry [a]	difference	time (h)	yield (%) [b]	er [c]
**1**	No change	18	97	96:04
**2**	BF_3 Et_2O (1.0 equiv) was used under -10 °C	0.33	66	54:46
**3**	3 Å MS was used instead of 4 Å MS	18	99	96:04
**4**	10 mol% of **4** was used	18	87	89:11
**5**	30 mol% of **4** was used	18	99	96:04
**6**	Catalyst **4** was recycled for reused	18	97	95:05

[a] All reactions were conducted on 0.21 mmol starting material in 0.2 M solvent. [b] Isolated yield. [c] Determined by HPLC with chiral stationary phase.

Supplementary Table 4. Evaluation of different conditions.

5. *Different boron sources:*

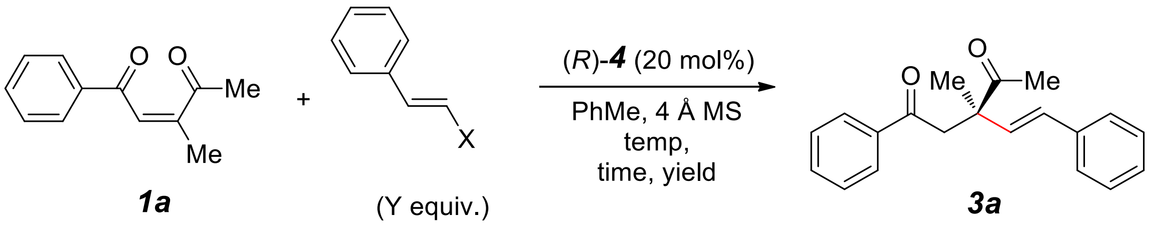

entry [a]	X	Y	temp (°C)	time (h)	yield (%) [b]	er [c]
**1**	BF_3K	2.0	70	18	97	96:04
**2**	$B(OH)_2$	2.0	70	1.5	99	72:28
**3**	$B(OH)_2$	1.5	20	5	99	80:20
**4** [d]	$B(OH)_2$	2.0	70	4	99 [e]	50:50
**5**	Bpin	2.0	70	48	26 [f]	N.D.

[a] All reactions were conducted on 0.21 mmol starting material in 0.2 M solvent. [b] Isolated yield. [c] Determined by HPLC with chiral stationary phase. [d] Without catalyst. [e] NMR yield, CH_2Br_2 was used as NMR standard. [f] Reaction did not complete.

Supplementary Table 5. Evaluation of different boron sources.

The potassium trifluoroborate salt (entry 1.) was found to be the best nucleophile.

General procedure 4 (GP4) or the conjugate addition

To a flame–dried round-bottomed flask equipped with magnetic stir bar and activated 4 Å molecular sieve (53 mg, 250 mg/mmol) was added endiketone **1a** (40 mg, 0.21 mmol, 1.0 equiv), potassium trifluoroborate (1.2–2.0 equiv,), (*R*)–3,3′–(C_7F_7)$_2$–BINOL (20–40 mol%) followed by the addition of toluene (1 mL, 0.2 M) at room temperature. The reaction mixture was then allowed to stir at 70 ºC for 18–64 h. After the reaction completed, the reaction was cooled down to room temperature, and the solvent was removed under reduced pressure. The crude mixture was directly purified via flash column chromatography on silica gel with appropriate eluents to give the desired product.

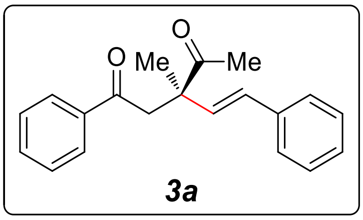

Following GP4, the product was synthesized from diketone **1a** (40 mg, 0.21 mmol, 1.0 equiv), potassium (*E*)–trifluoro(styryl)borate (98mg, 0.47 mmol, 2.2 equiv,), (*R*)–3,3′–(C_7F_7)$_2$–BINOL (30.0 mg, 0.04 mmol, 0.2 equiv). Purification was accomplished via flash column chromatography with appropriate eluents (EtOAc : hexane = 1 : 10) to give the product as a colorless gel **3a** (54 mg, 0.18 mmol) in 99% yield. **IR (neat)** 1706, 1682, 1448, 1350 cm^{-1}.; **^{1}H NMR** (400 MHz, $CDCl_3$) δ 7.98 (d, *J* = 8.0 Hz, 2H), 7.61–7.58 (m, 1H), 7.50–7.47 (m, 2H), 7.43–7.41 (m, 2H), 7.35 (t, *J* = 8.0 Hz, 2H), 7.29–7.28 (m, 1H), 6.54 (d, *J* = 16.0 Hz, 1H), 6.50 (d, *J* = 16.0 Hz, 1H), 3.61 (d, *J* = 16.0 Hz, 1H), 3.54 (d, *J* = 16.0 Hz, 1H), 2.34 (s, 3H), 1.58 (s, 3H); **^{13}C{^{1}H} NMR** (100 MHz, $CDCl_3$) δ 210.5, 197.5, 136.8, 136.7, 133.2, 132.8, 129.9, 128.6, 128.5, 128.0, 127.7, 126.4, 51.5 (-**C**), 48.4 (-**C**H_2), 26.7 (-**C**H_3), 21.8 (-**C**H_3); **HRMS (ESI)** m/z Calculated for $C_{20}H_{20}O_2$ [M+Na]$^+$: 315.1356; found: 315.1362. **Rf**: 0.54 (EtOAc : hexane = 1 : 3). **HPLC Chiralcel ID** (hexanes/ i-PrOH = 90:10, 1.0mL/min, UV-254 detector, tr (major) = 7.29 min, tr (minor) = 7.77 min).

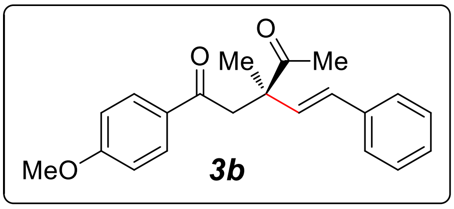

Following GP4, the product was synthesized from diketone **1b** (43 mg, 0.197 mmol), potassium (*E*)–trifluoro(styryl)borate (89 mg, 0.42 mmol), (*R*)–3,3′–(C_7F_7)$_2$–BINOL (31.0 mg, 0.04 mmol). Purification was accomplished via flash column chromatography with appropriate eluents (EtOAc : hexane = 1 : 10) to give the product **3b** as a colorless gel (59 mg, 0.183 mmol) in 93% yield. **IR (neat)** 2915, 1696, 1670, 1595, 1348, 1169 cm^{-1}; **^{1}H NMR** (400 MHz, $CDCl_3$) δ 7.93 (d, *J* = 8.0 Hz, 2H), 7.38 (d, *J* = 8.0 Hz, 2H), 7.32 (d, *J* = 8.0 Hz, 2H), 7.26–7.22 (m, 1H), 6.92 (d, *J* = 8.0 Hz, 2H), 6.51 (s, 2H), 3.87 (s, 3H), 3.53 (d, *J* = 16.0 Hz, 1H), 3.47 (d, *J* = 16.0 Hz, 1H), 2.31 (s, 3H), 1.53 (s, 3H); **^{13}C{^{1}H} NMR** (100 MHz, $CDCl_3$) δ 210.7, 195.9, 163.5, 136.7, 132.9, 130.3, 129.7, 128.5, 127.6, 126.3, 113.6, 55.4, 51.3, 48.2, 26.7, 21.7; **HRMS (ESI)** m/z Calculated for $C_{21}H_{22}O_3$ [M+Na]$^+$: 345.1461; found: 345.1469. **Rf**: 0.40 (EtOAc : hexane = 1 : 3). **HPLC Chiralcel ID** (hexanes/ i-PrOH = 90:10, 1.0mL/min, UV-254 detector, tr (major) = 15.7 min, tr (minor) = 19.4 min), **$[\alpha]_D^{20}$** = -107.1 (c = 0.03, acetone).

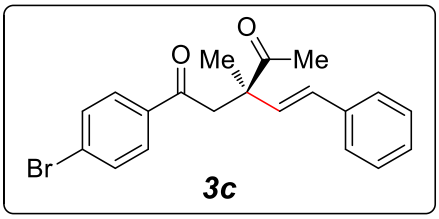

Following GP4, the product was synthesized from diketone **1c** (54 mg, 0.20 mmol, 1.0 equiv), potassium (*E*)–trifluoro(styryl)borate (86 mg, 0.41 mmol, 2.0 equiv,), (*R*)–3,3′–$(C_7F_7)_2$–BINOL (30.0 mg, 0.04 mmol, 0.2 equiv). Purification was accomplished via flash column chromatography with appropriate eluents (EtOAc : hexane = 1 : 10) to give the product **3c** as a colorless gel (61 mg, 0.164 mmol) in 81% yield. **IR (neat)** 1739, 1695, 1365, 1188 cm^{-1}; **^{1}H NMR** (400 MHz, $CDCl_3$) δ 7.83 (d, *J* = 8.0 Hz, 2H), 7.62 (d, *J* = 8.0 Hz, 2H), 7.41 (d, *J* = 8.0 Hz, 2H), 7.35 (t, *J* = 8.0 Hz, 2H), 7.30–7.28 (m, 1H), 6.56 (d, *J* = 16.0 Hz, 1H), 6.51 (d, *J* = 16.0 Hz, 1H), 3.54 (d, *J* = 20.0 Hz, 1H), 3.47 (d, *J* = 20.0 Hz, 1H), 2.33 (s, 3H), 1.58 (s, 3H); **^{13}C{^{1}H} NMR** (100 MHz, $CDCl_3$) δ 210.6, 196.5, 136.6, 135.4, 132.4, 131.8, 130.1, 129.5, 128.6, 128.4, 127.8, 126.3, 51.5, 48.3, 26.7, 21.7; **HRMS (ESI)** m/z Calculated for $C_{20}H_{19}BrO_2$ $[M+H]^+$: 371.0641; found: 371.0641. **Rf**: 0.76 (EtOAc : hexane = 1 : 3). **HPLC Chiralcel ID** (hexanes/ i-PrOH = 90:10, 1.0mL/min, UV-254 detector, tr (major) = 7.40 min, tr (minor) = 8.40 min), **$[\alpha]_D^{20}$** = -8.2 (c = 0.015, acetone).

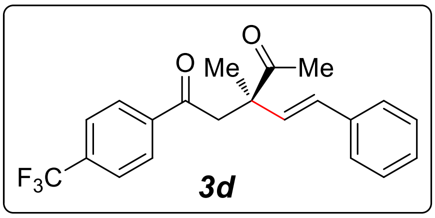

Following GP4, the product was synthesized from diketone **1d** (50 mg, 0.20 mmol), potassium (*E*)–trifluoro(styryl)borate (90 mg, 0.42 mmol), (*R*)–3,3′–$(C_7F_7)_2$–BINOL (30.0 mg, 0.04 mmol). Purification was accomplished via flash column chromatography with appropriate eluents (EtOAc : hexane = 1 : 15) to give the product **3d** as a colorless gel (59 mg, 0.164 mmol) in 84% yield. **IR (neat)** 1695, 1670, 1596, 1322 cm^{-1}; **^{1}H NMR** (400 MHz, $CDCl_3$) δ 8.07 (d, *J* = 8.0 Hz, 2H), 7.74 (d, *J* = 8.0 Hz, 2H), 7.42–7.28 (m, 5H), 6.58 (d, *J* = 16.0 Hz, 1H), 6.52 (d, *J* = 16.0 Hz, 1H), 3.58 (d, *J* = 20.0 Hz, 1H), 3.51 (d, *J* = 20.0 Hz, 1H), 2.33 (s, 3H), 1.60 (s, 3H); **^{13}C{^{1}H} NMR** (100 MHz, $CDCl_3$) δ 210.5, 196.7, 139.3, 136.5, 134.4 (q, $^2J_{C-F}$= 33 Hz), 132.2, 130.2, 128.6, 128.3, 127.9, 126.3, 125.2 (d, $^3J_{C-F}$ = 4.0 Hz), 123.5 (q, $^1J_{C-F}$= 272 Hz), 51.6, 48.5, 26.6, 21.7; **^{19}F NMR** (375 MHz, $CDCl_3$) δ -63.02 (-C**F**$_3$); **HRMS (ESI)** m/z Calculated for $C_{21}H_{19}F_3O_2$ $[M+H]^+$: 361.1410; found: 361.1413. **Rf**: 0.65 (EtOAc : hexane = 1 : 3). **HPLC Chiralcel ID** (hexanes/ i-PrOH = 90:10, 1.0mL/min, UV-254 detector, tr (major) = 4.7 min, tr (minor) = 5.3 min), **$[\alpha]_D^{20}$** = -214.3 (c = 0.8, acetone).

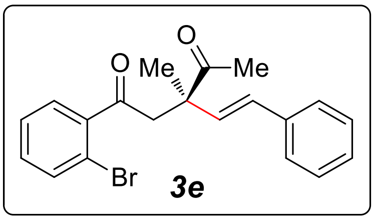

Following GP4, the product was synthesized from diketone **1k** (50 mg, 0.20 mmol), potassium (*E*)–trifluoro(styryl)borate (84 mg, 0.40 mmol, 2.0 equiv), (*R*)–3,3′–$(C_7F_7)_2$–BINOL (30.0 mg, 0.04 mmol). Purification was accomplished via flash column chromatography with appropriate eluents (EtOAc : hexane = 1 : 10) to give the product **3e** as a colorless gel (45 mg, 0.121 mmol) in 65% yield. **IR (neat)** 1738, 1700, 1365, 1190 cm^{-1}; **^{1}H NMR** (400 MHz, $CDCl_3$) δ 7.53 (d, *J* = 8.0 Hz, 1H), 7.44–7.26 (m, 8H), 6.53 (d, *J* = 16.0 Hz, 1H), 6.44 (d,

J = 16.0 Hz, 1H), 3.47 (d, *J* = 18.0 Hz, 1H), 3.41 (d, *J* = 18.0 Hz, 1H), 2.29 (s, 3H), 1.57 (s, 3H); **^{13}C{^{1}H} NMR** (100 MHz, $CDCl_3$) δ 210.0, 201.8, 141.4, 136.6, 133.5, 132.0, 131.5, 130.2, 128.6, 128.4, 127.8, 127.4, 126.4, 118.3, 52.2, 51.7, 26.4, 21.5 **HRMS (ESI)** m/z Calculated for $C_{20}H_{19}BrO_2$ $[M+Na]^+$: 393.0461; found: 393.0463. **Rf**: 0.54 (EtOAc : hexane = 1 : 3). **HPLC Chiralcel ID** (hexanes/ i-PrOH = 90:10, 1.0mL/min, UV-254 detector, tr (major) = 7.7 min, tr (minor) = 8.9 min).

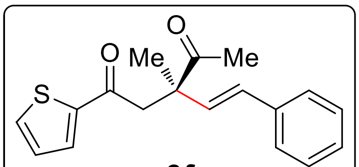

3f

Following GP4, the product was synthesized from diketone **1f** (39 mg, 0.20 mmol), potassium (*E*)–trifluoro(styryl)borate (81 mg, 0.40 mmol, 2.0 equiv), (*R*)–3,3′–$(C_7F_7)_2$–BINOL (30.0 mg, 0.04 mmol). Purification was accomplished via flash column chromatography with appropriate eluents (EtOAc : hexane = 1 : 10) to give the product **3f** as a colorless gel (55 mg, 0.184 mmol) in 94% yield. **IR (neat)** 1705, 1655, 1414, 1354 cm^{-1}; **^{1}H NMR** (400 MHz, $CDCl_3$) δ 7.71 (d, *J* = 4.0 Hz, 1H), 7.63 (d, *J* = 4.0 Hz, 1H), 7.38 (d, *J* = 8.0 Hz, 2H), 7.32 (d, *J* = 8.0 Hz, 2H), 7.26–7.23 (m, 1H), 7.12 (d, *J* = 4.0 Hz, 1H), 6.53 (d, *J* = 16.0 Hz, 1H), 6.47 (d, *J* = 16.0 Hz, 1H), 3.50 (d, *J* = 16.0 Hz, 1H), 3.43 (d, *J* = 16.0 Hz, 1H), 2.29 (s, 3H), 1.54 (s, 3H); **^{13}C{^{1}H} NMR** (100 MHz, $CDCl_3$) δ 210.3, 190.5, 143.9, 136.6, 133.8, 132.4, 132.0, 130.0, 128.6, 128.1, 127.8, 126.3, 51.6, 48.6, 26.6, 21.6; **HRMS (ESI)** m/z Calculated for $C_{18}H_{18}O_2S$ $[M+Na]^+$: 321.0920; found: 321.0927. **Rf**: 0.50 (EtOAc : hexane = 1 : 3). **HPLC Chiralcel ID** (hexanes/ i-PrOH = 90:10, 1.0mL/min, UV-254 detector, tr (major) = 10.6 min, tr (minor) = 12.3 min), **$[\alpha]_D^{20}$** = -98.2 (c = 0.02, acetone).

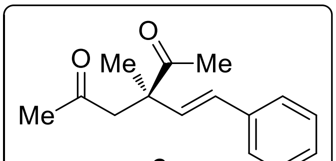

3g

Following GP4, the product was synthesized from diketone **1g** (25 mg, 0.20 mmol), potassium (*E*)–trifluoro(styryl)borate (88 mg, 0.42 mmol, 2.0 equiv), (*R*)–3,3′–$(C_7F_7)_2$–BINOL (29.0 mg, 0.04 mmol). Purification was accomplished via flash column chromatography with appropriate eluents (EtOAc : hexane = 1 : 15) to give the product **3g** as a colorless gel (20 mg, 0.087 mmol) in 44% yield. **IR (neat)** 2984, 1703, 1356 cm^{-1}; **^{1}H NMR** (400 MHz, $CDCl_3$) δ 7.38–7.30 (m, 4H), 7.26–7.23 (m, 1H), 6.47 (d, *J* = 16.0 Hz, 1H), 6.39 (d, *J* = 16.0 Hz, 1H), 2.99 (d, *J* = 20.0 Hz, 1H), 2.91 (d, *J* = 20.0 Hz, 1H), 2.21 (s, 3H), 2.14 (s, 3H), 1.45 (s, 3H); **^{13}C{^{1}H} NMR** (100 MHz, $CDCl_3$) δ 210.5, 206.3, 136.6, 132.3, 129.9, 128.6, 127.8, 126.3, 52.6, 51.5, 30.4, 26.5, 21.4; **HRMS (ESI)** m/z Calculated for $C_{15}H_{18}O_2$ $[M+H]^+$: 231.1380; found: 231.1378. **Rf**: 0.54 (EtOAc : hexane = 1 : 3). **HPLC Chiralcel ID** (hexanes/ i-PrOH = 90:10, 1.0mL/min, UV-254 detector, tr (major) = 6.3 min, tr (minor) = 9.9 min)..

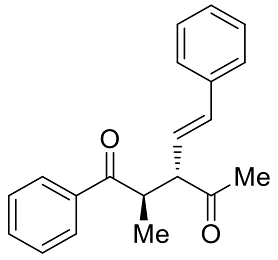

5h

Following GP4, the product was synthesized from diketone **1i** (38 mg, 0.20 mmol, 1.0 equiv), potassium (*E*)–trifluoro(styryl)borate (89 mg, 0.42 mmol), (*R*)–3,3′–(C_7F_7)$_2$–BINOL (30.0 mg, 0.04 mmol, 0.2 equiv). Purification was accomplished via flash column chromatography with appropriate eluents (EtOAc : hexane = 1 : 10) to give the product **5h** as a colorless gel (37 mg, 0.126 mmol) in 63% yield.**IR (neat)** 2970, 1711, 1676, 1447 cm^{-1}; **^{1}H NMR** (400 MHz, $CDCl_3$) δ 7.99 (d, *J* = 8.0 Hz, 2H), 7.57 (t, *J* = 8.0 Hz, 1H), 7.48 (t, *J* = 8.0 Hz, 2H), 7.41 (d, *J* = 8.0 Hz, 2H), 7.37–7.34 (m, 2H), 7.29 (d, *J* = 8.0 Hz, 1H), 6.72 (d, *J* = 16.0 Hz, 1H), 6.01 (dd, *J* = 16.0, 12.0 Hz, 1H), 4.01–3.93 (m, 1H), 3.87 (t, *J* = 12.0 Hz, 1H), 2.27 (s, 3H), 1.17 (d, *J* = 8.0 Hz, 3H); **^{13}C{^{1}H} NMR** (100 MHz, $CDCl_3$) δ 208.8, 203.4, 136.3, 135.9, 135.8, 133.1, 128.9, 128.6, 128.4, 128.0, 126.3, 125.2, 60.1, 42.5, 29.5, 16.3; **HRMS (ESI)** m/z Calculated for $C_{20}H_{20}O_2$ [M+Na]$^+$: 315.1356; found: 315.1360. **Rf**: 0.67 (EtOAc : hexane = 1 : 3). HPLC **Chiralpak AS–H** (hexanes/ i-PrOH = 90:10, 1.0mL/min, UV-254 detector, tr (major) = 6.8 min, tr (minor) = 7.4 min).

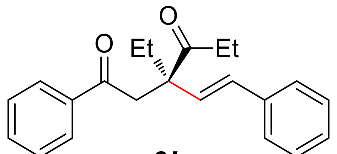

3i

Following GP4, the product was synthesized from diketone **1h** (43 mg, 0.20 mmol, 1.0 equiv), potassium (*E*)–trifluoro(styryl)borate (89 mg, 0.42 mmol), (*R*)–3,3′–(C_7F_7)$_2$–BINOL (30.0 mg, 0.04 mmol, 0.2 equiv). Purification was accomplished via flash column chromatography with appropriate eluents (EtOAc : hexane = 1 : 10) to give the product **3i** as a colorless gel (50 mg, 0.156 mmol) in 78% yield. **IR (neat)** 2969, 1705, 1683, 1448, 747 cm^{-1}; **^{1}H NMR** (500 MHz, $CDCl_3$) δ 7.97–7.96 (m, 2H), 7.58–7.54 (m, 1H), 7.46 (t, *J* = 8.0 Hz, 2H), 7.37–7.35 (m, 2H), 7.30 (t, *J* = 8.0 Hz, 2H), 7.24–7.21 (m, 1H), 6.57 (d, *J* = 20.0 Hz, 1H), 6.41 (d, *J* = 20.0 Hz, 1H), 3.71 (d, *J* = 15.0 Hz, 1H), 3.49 (d, *J* = 15.0 Hz, 1H), 2.81–1.73 (m, 1H), 2.64–1.56 (m, 1H), 2.03–1.99 (m, 2H), 1.08 (t, *J* = 10.0 Hz, 3H), 0.89 (t, *J* = 10.0 Hz, 3H); **^{13}C{^{1}H} NMR** (150 MHz, $CDCl_3$) δ 212.8, 197.7, 136.9, 136.7, 133.1, 131.3, 130.1, 128.5, 128.1, 127.8, 127.5, 126.2, 55.1, 44.8, 32.5, 28.6, 8.8, 7.9; **HRMS (ESI)** m/z Calculated for $C_{22}H_{24}O_2$ [M+Na]$^+$: 343.1669; found: 343.1678. **Rf**: 0.67 (EtOAc : hexane = 1 : 3). **HPLC Chiralcel ID** (hexanes/ i-PrOH = 90:10, 1.0mL/min, UV-254 detector, tr (major) = 5.8 min, tr (minor) = 6.4 min), **$[\alpha]_D^{20}$** = -41.6 (c = 0.025, acetone).

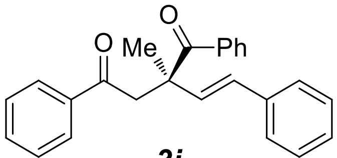

3j

Following GP4, the product was synthesized from diketone **1e** (50 mg, 0.20 mmol, 1.0 equiv), potassium (*E*)–trifluoro(styryl)borate (80 mg, 0.38 mmol), (*R*)–3,3′–(C_7F_7)$_2$–BINOL (60.0 mg, 0.08 mmol, 0.4 equiv). Purification was accomplished via flash column chromatography with appropriate eluents (EtOAc : hexane = 1 : 10) to give the product **3j** as a white solid (60 mg, 0.169 mmol) in 83% yield. **IR (neat)** 2931, 1668, 1596, 1447 cm^{-1}; **^{1}H NMR** (400 MHz, $CDCl_3$) δ 7.95 (d, *J* = 7.2 Hz, 2H), 7.83 (d, *J* = 7.2 Hz, 2H), 7.55 (t,

J = 7.2 Hz, 1H), 7.46–7.30 (m, 9H), 7.26–7.23 (m, 1H), 6.80 (d, J = 16.4 Hz, 1H), 6.60 (d, J = 16.4 Hz, 1H), 3.79 (d, J = 17.6 Hz, 1H), 3.65 (d, J = 17.6 Hz, 1H)), 1.72 (s, 3H); **^{13}C{^{1}H} NMR** (100 MHz, $CDCl_3$) δ 205.2, 197.1, 138.2, 136.8, 136.7, 134.0, 133.1, 131.2, 129.2, 128.62, 128.56, 128.50, 128.00, 127.99, 127.7, 126.3, 50.8, 50.5, 24.0; **HRMS (ESI)** m/z Calculated for $C_{25}H_{22}O_2$ $[M+H]^+$: 355.1700; found: 355.1693. **Rf**: 0.63 (EtOAc : hexane = 1 : 3) **HPLC Chiralcel ID** (hexanes/ i-PrOH = 90:10, 1.0mL/min, UV-254 detector, tr (major) = 11.2 min, tr (minor) = 9.7 min), **$[\alpha]_D^{20}$** = -21.5 (c = 0.03, acetone)..

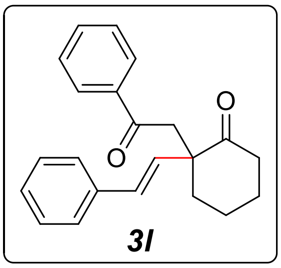

Following GP4, the product was synthesized from diketone **1j** (44 mg, 0.205 mmol), potassium (E)–trifluoro(styryl)borate (86 mg, 0.40 mmol, 2.0 equiv), (R)–3,3′–(C_7F_7)$_2$–BINOL (29.0 mg, 0.04 mmol). Purification was accomplished via flash column chromatography with appropriate eluents (EtOAc : hexane = 1 : 15 to 1 : 10) to give the product **3l** as a colorless gel (61 mg, 0.191 mmol) in 93% yield. **IR (neat)** 2931, 1684, 1447, 1207 cm^{-1}; **^{1}H NMR** (500 MHz, $CDCl_3$) δ 7.93 (d, J = 7.2 Hz, 2H), 7.52 (d, J = 7.4 Hz, 1H), 7.42 (t, J = 7.8 Hz, 2H), 7.34–7.28 (m, 4H), 7.24–7.21 (m, 1H), 6.36 (d, J = 16.5 Hz, 1H), 6.31 (d, J = 16.5 Hz, 1H), 3.65 (d, J = 17.8 Hz, 1H), 3.28 (d, J = 17.8 Hz, 1H), 2.57–2.48 (m, 2H), 2.25–2.15 (m, 2H), 2.03–1.81 (m, 4H); **^{13}C{^{1}H} NMR** (150 MHz, $CDCl_3$) δ 211.4, 197.4, 137.1, 136.5, 133.0, 132.9, 131.0, 128.6, 128.4, 127.9, 127.7, 126.2, 52.8 (-**C**), 47.5, 39.4, 35.9, 25.9, 22.0; **HRMS (ESI)** m/z Calculated for $C_{22}H_{22}O_2$ $[M+H]^+$: 319.1693; found: 319.1702. **Rf**: 0.76 (EtOAc : hexane = 1 : 3). HPLC Chiralcel ID (hexanes/ i-PrOH = 90:10, 1.0mL/min, UV-254 detector). **HPLC Chiralcel ID** (hexanes/ i-PrOH = 90:10, 1.0mL/min, UV-254 detector, tr (major) = 7.3 min, tr (minor) = 9.5 min).

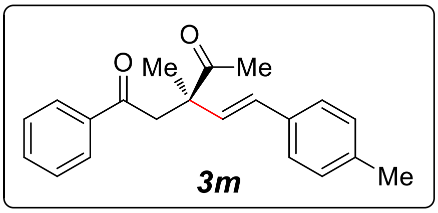

Following GP4, the product was synthesized from diketone **1a** (40 mg, 0.212 mmol), potassium ($1E$)–2–(4–methoxyphenyl)ethenyl trifluoroborate (103 mg, 0.46 mmol, 2.1 equiv,), (R)–3,3′–(C_7F_7)$_2$–BINOL (30.0 mg, 0.04 mmol, 0.4 equiv). Purification was accomplished via flash column chromatography with appropriate eluents (EtOAc : hexane = 1 : 10) to give the product **3m** as a white solid (61 mg, 0.199 mmol) in 94% yield. **IR (neat)** 2926, 1703, 1675, 1449, 1346 cm^{-1}; **^{1}H NMR** (400 MHz, $CDCl_3$) δ 7.96–7.94 (m, 2H), 7.58–7.54 (m, 1H), 7.47–7.43 (m, 2H), 7.28–7.26 (m, 2H), 7.12 (d, J = 8.0 Hz, 2H), 6.50 (d, J = 16.0 Hz, 1H), 6.43 (d, J = 16.0 Hz, 1H), 3.56 (d, J = 20.0 Hz, 1H), 3.49 (d, J = 20.0 Hz, 1H), 2.33 (s, 3H), 2.30 (s, 3H), 1.53 (s, 3H); **^{13}C{^{1}H} NMR** (125 MHz, $CDCl_3$) δ 210.7, 197.5, 137.6, 136.7, 133.9, 133.2, 131.6, 129.7, 129.3, 128.5, 128.0, 126.2, 51.4, 48.4, 26.6, 21.7, 21.1; **HRMS (ESI)** m/z Calculated for $C_{21}H_{22}O_2$ $[M+H]^+$: 329.1512; found: 329.1521. **Rf**: 0.57 (EtOAc : hexane = 1 : 3). **HPLC Chiralcel ID** (hexanes/ i-PrOH = 90:10, 1.0mL/min, UV-254 detector, tr (major) = 8.3 min, tr (minor) = 9.0 min), **$[\alpha]_D^{20}$** = -44.4 (c = 0.0185, acetone)..

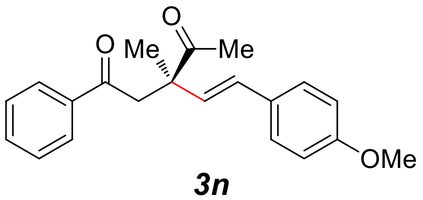

3n

Following GP4, the product was synthesized from diketone **1a** (42 mg, 0.22 mmol), potassium (*1E*)–2–(4–methoxyphenyl)ethenyl trifluoroborate (60 mg, 0.25 mmol, 1.2 equiv,), (*R*)–3,3′–(C_7F_7)$_2$–BINOL (60.0 mg, 0.08 mmol, 0.4 equiv). Purification was accomplished via flash column chromatography with appropriate eluents (EtOAc : hexane = 1 : 6) to give the product **3n** as a colorless gel (70 mg, 0.217 mmol) in 97% yield. **IR (neat)** 2933, 1704, 1684, 1606, 1511, 1448 cm^{-1}; **^{1}H NMR** (400 MHz, $CDCl_3$) δ 7.95 (d, *J* = 8.0 Hz, 2H), 7.58–7.54 (m, 1H), 7.45 (t, *J* = 8.0 Hz, 2H), 7.32 (d, *J* = 12.0 Hz, 2H), 6.86 (d, *J* = 12.0 Hz, 2H), 6.47 (d, *J* = 16.0 Hz, 1H), 6.34 (d, *J* = 16.0 Hz, 1H), 3.80 (s, 3H), 3.57 (d, *J* = 20.0 Hz, 1H), 3.48 (d, *J* = 20.0 Hz, 1H), 2.30 (s, 3H), 1.53 (s, 3H); **^{13}C{^{1}H} NMR** (100 MHz, $CDCl_3$) δ 210.8, 197.6, 159.3, 136.7, 133.2, 130.4, 129.4, 129.3, 128.5, 128.0, 127.5, 114.0, 55.3, 51.3, 48.5, 26.6, 21.7; **HRMS (ESI)** m/z Calculated for $C_{21}H_{22}O_3$ [M+Na]$^+$: 345.1461; found: 345.1470. **Rf**: 0.50 (EtOAc : hexane = 1 : 3). **HPLC Chiralcel ID** (hexanes/ i-PrOH = 90:10, 1.0 mL/min, UV-254 detector, tr (major) = 13.3 min, tr (minor) = 15.8 min), **[α]$_D$^{20}** = -72.5 (c = 0.035, acetone)..

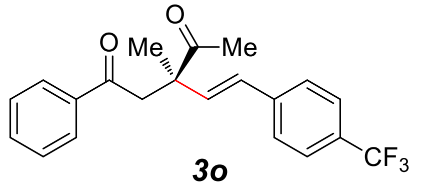

3o

Following GP4, the product was synthesized from diketone **1a** (41 mg, 0.218 mmol), potassium (*1E*)–2–(4–trifluoromethylphenyl)ethenyl trifluoroborate (122 mg, 0.44 mmol, 2.0 equiv,), (*R*)–3,3′–(C_7F_7)$_2$–BINOL (30.0 mg, 0.04 mmol, 0.2 equiv). Purification was accomplished via flash column chromatography with appropriate eluents (EtOAc : hexane = 1 : 9) to give the product **3o** as a colorless gel (73 mg, 0.202 mmol) in 94% yield. **IR (neat)** 1699, 1683, 1448, 1319 cm^{-1}; **^{1}H NMR** (500 MHz, $CDCl_3$) δ 7.96–7.94 (m, 2H), 7.58–7.54 (m, 1H), 7.47–7.43 (m, 2H), 7.28–7.26 (m, 2H), 7.12 (d, *J* = 8.0 Hz, 2H), 6.50 (d, *J* = 16.0 Hz, 1H), 6.43 (d, *J* = 16.0 Hz, 1H), 3.68–3.43 (m, 2H), 2.32 (s, 3H), 1.55 (s, 3H); **^{13}C{^{1}H} NMR** (125 MHz, $CDCl_3$) δ 210.2, 197.3, 140.2, 136.5, 135.5, 133.4, 129.5 (q, $^2J_{C-F}$= 32 Hz), 128.65, 128.61, 128.0, 126.5, 125.6 (q, $^3J_{C-F}$ = 3.7 Hz), 124.1 (q, $^1J_{C-F}$= 270 Hz), 51.6, 48.4, 26.8, 21.8; **^{19}F NMR** (375 MHz, $CDCl_3$) δ -62.42 (-**CF_3**); **HRMS (ESI)** m/z Calculated for $C_{21}H_{19}F_3O_3$ [M+H]$^+$: 361.1410; found: 361.1418. **Rf**: 0.63 (EtOAc : hexane = 1 : 3. **HPLC Chiralcel ID** (hexanes/ i-PrOH = 92:8, 1.0 mL/min, UV-254 detector, tr (major) = 7.1 min, tr (minor) = 7.6 min), **[α]$_D$^{20}** = -61.8 (c = 0.035, acetone)..

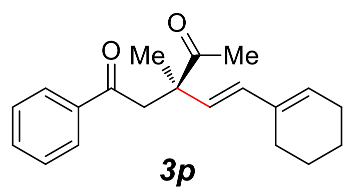

3p

Following GP4, the product was synthesized from diketone **1a** (41 mg, 0.21 mmol), potassium (*1E*)–2–(1–cyclohexen–1–yl)ethenyl trifluoroborate (89 mg, 0.41 mmol, 2.0 equiv,), (*R*)–3,3′–(C_7F_7)$_2$–BINOL (30.0 mg, 0.04 mmol, 0.2 equiv). Purification was accomplished via flash column chromatography with appropriate eluents (EtOAc : hexane = 1 : 10) to give the product **3p** as a colorless gel (45 mg) in 70% yield. **IR**

(neat) 2930, 1705, 1684, 1448, 1350 cm^{-1}; ^{1}**H NMR** (500 MHz, $CDCl_3$) δ 7.92 (d, *J* = 4.0 Hz, 2H), 7.54 (t, *J* = 7.5 Hz, 1H), 7.44 (t, *J* = 7.5 Hz, 2H), 6.15 (d, *J* = 16.5 Hz, 1H), 5.76–5.72 (m, 2H), 3.51 (d, *J* = 18.0 Hz, 1H), 3.39 (d, *J* = 18.0 Hz, 1H), 2.24 (s, 3H), 2.11–2.10 (m, 4H), 1.68–1.56 (m, 4H), 1.45 (s, 3H); ^{13}**C{^{1}H} NMR** (125 MHz, $CDCl_3$) δ 211.0, 197.7, 136.8, 135.2, 133.5, 133.1, 130.2, 128.5, 128.4, 128.0, 51.1, 48.4, 26.5, 25.8, 24.5, 22.4, 22.3, 21.7; **HRMS (ESI)** m/z Calculated for $C_{20}H_{24}O_2$ $[M+Na]^+$: 319.1669; found: 319.1671. **Rf**: 0.68 (EtOAc : hexane = 1 : 3. **HPLC Chiralpak AD–H** (hexanes/ i-PrOH = 99:1, 1.0mL/min, UV-254 detector, tr (major) = 12.5 min, tr (minor) = 14.1 min), $[\alpha]_D^{20}$ = -16.1 (c = 0.0225, acetone).

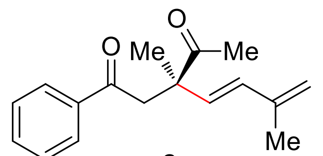

3q

Following GP4, the product was synthesized from diketone **1a** (40 mg, 0.21 mmol), potassium (*E*)-trifluoro(3-methylbuta-1,3-dien-1-yl)trifluoroborate (74 mg, 0.42 mmol, 2.0 equiv,), (*R*)–3,3′–$(C_7F_7)_2$–BINOL (45 mg, 0.06 mmol, 0.3 equiv). Purification was accomplished via flash column chromatography with appropriate eluents (EtOAc : hexane = 1 : 10) to give the product **3q** as a colorless gel (19 mg, 0.074 mmol) in 35% yield. **IR (neat)** 1706, 1683, 1448, 1350 cm^{-1}; ^{1}**H NMR** (500 MHz, $CDCl_3$) δ 7.93 (d, *J* = 5.0 Hz, 2H), 7.56 (t, *J* = 5.0 Hz, 1H), 7.45 (t, *J* = 5.0 Hz, 2H), 6.26 (d, *J* = 16.5 Hz, 1H), 6.15 (d, *J* = 15.0 Hz, 1H), 5.87 (d, *J* = 15.0 Hz, 1H), 4.99 (d, *J* = 5.0 Hz, 1H), 3.51 (d, *J* = 20.0 Hz, 1H), 3.42 (d, *J* = 20.0 Hz, 1H), 2.26 (s, 3H), 1.83 (s, 3H), 1.47 (s, 3H); ^{13}**C{^{1}H} NMR** (125 MHz, $CDCl_3$) δ 210.7, 197.5, 141.5, 136.8, 133.2, 132.7, 132.6, 128.5, 128.0, 117.0, 51.2 (-**C**), 48.4 (-**C**H_2), 26.6 (-**C**H_3), 21.7 (-**C**H_3), 18.6 (-**C**H_3); **HRMS (ESI)** m/z Calculated for $C_{17}H_{20}O_2$ $[M+Na]^+$: 279.1356; found: 279.1354. **Rf**: 0.68 (EtOAc : hexane = 1 : 3. **HPLC Chiralcel AS–H** (hexanes/ i-PrOH = 92:8, 0.7 mL/min, UV-190 detector, tr (major) = 9.1 min, tr (minor) = 10.0 min), $[\alpha]_D^{20}$ = -18.2 (c = 0.0095, acetone).

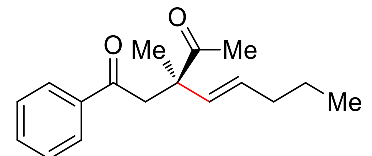

3r

Following GP4, the product was synthesized from diketone **1a** (40 mg, 0.21 mmol, 1.0 equiv), potassium (*E*)–(pentenyl)trifluoroborate (80 mg, 0.45 mmol, 2.0 equiv,), (*R*)–3,3′–$(C_7F_7)_2$–BINOL (30.0 mg, 0.04 mmol, 0.2 equiv) in $PhCF_3$ (1 mL). Purification was accomplished via flash column chromatography with appropriate eluents (EtOAc : hexane = 1 : 10) to give the product **3r** as a colorless gel (51 mg, 0.197 mmol) in 91% yield. **IR (neat)** 1739, 1710, 1685, 1449. 1351 cm^{-1} ; ^{1}**H NMR** (400 MHz, $CDCl_3$) δ 7.92 (d, *J* = 8.0 Hz, 2H), 7.53 (t, *J* = 8.0 Hz, 1H), 7.43 (t, *J* = 8.0 Hz, 2H), 5.69–5.53 (m, 2H), 3.46 (d, *J* = 16.0 Hz, 1H), 3.35 (d, *J* = 16.0 Hz, 1H), 2.23 (s, 3H), 2.01 (dd, *J* = 8.0, 4.0 Hz, 2H), 1.39–1.35 (m, 5H), 0.87 (t, *J* = 8.0 Hz, 3H); ^{13}**C{^{1}H} NMR** (100 MHz, $CDCl_3$) δ 211.0. 197.7, 136.8, 133.1, 131.0, 128.4, 127.9, 51.1, 48.1, 34.7, 26.2, 22.3, 21.8, 13.5; **HRMS (APCI)** m/z Calculated for $C_{17}H_{22}O_2$ $[M+H]^+$: 259.1693; found: 259.1693. **Rf**: 0.74 (EtOAc : hexane = 1 : 3). **HPLC Chiralcel OD–H** (hexanes/ i-PrOH = 99:1, 1.0 mL/min, UV-254 detector, tr (major) = 11.9 min, tr (minor) = 11.4 min), $[\alpha]_D^{20}$ = -19.2 (c = 0.025, acetone).

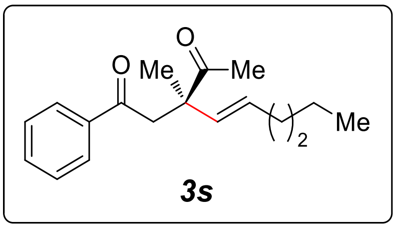

Following GP4, the product was synthesized from diketone **1a** (40 mg, 0.21 mmol, 1.0 equiv), potassium (*E*)–(hexenyl)trifluoroborate (84 mg, 0.44 mmol, 2.0 equiv,), (*R*)–3,3′–$(C_7F_7)_2$–BINOL (45.0 mg, 0.06 mmol, 0.3 equiv) in $PhCF_3$ (1 mL). Purification was accomplished via flash column chromatography with appropriate eluents (EtOAc : hexane = 1 : 15) to give the product **3s** as a colorless gel (48 mg, 0.176 mmol) in 85% yield. **IR (neat)** 1706, 1684, 1448, 1349. 1002 cm^{-1} ; **^{1}H NMR** (600 MHz, $CDCl_3$) δ 7.92 (d, *J* = 7.5 Hz, 2H), 7.54 (t, *J* = 7.3 Hz, 1H), 7.43 (t, *J* = 7.6 Hz, 2H), 5.66 (d, *J* = 15.7 Hz, 1H), 5.58 (dt, *J* = 15.7, 6.6 Hz, 1H), 3.45 (d, *J* = 17.8 Hz, 1H), 3.36 (d, *J* = 17.7 Hz, 1H), 2.23 (s, 3H), 2.04 (q, *J* = 7.0 Hz, 2H), 1.39 (s, 3H), 1.35–1.25 (m, 13H), 0.87 (t, *J* = 7.1 Hz, 3H); **^{13}C{^{1}H} NMR** (150 MHz, $CDCl_3$) δ 211.1. 197.7, 136.9, 133.1, 132.9, 131.2, 128.5, 128.0, 51.2, 48.1, 32.4, 31.4, 26.3, 22.1, 21.8, 13.9; **HRMS (ESI)** m/z Calculated for $C_{18}H_{24}O_2$ $[M+Na]^+$: 295.1669; found: 295.1670. **Rf**: 0.70 (EtOAc : hexane = 1 : 3). **HPLC Chiralcel OD–H** (hexanes/ i-PrOH = 99:1, 1.0 mL/min, UV-254 detector, tr (major) = 10.6 min, tr (minor) = 10.0 min), **$[\alpha]_D^{20}$** = -47.9 (c = 0.024, acetone).

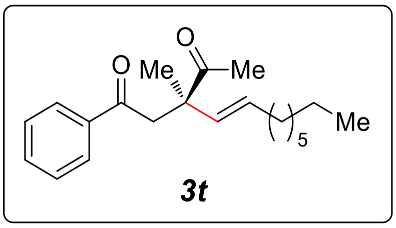

Following GP4, the product was synthesized from diketone **1a** (40 mg, 0.21 mmol, 1.0 equiv), potassium (*E*)–(nonenyl)trifluoroborate (94 mg, 0.42 mmol, 2.0 equiv,), (*R*)–3,3′–$(C_7F_7)_2$–BINOL (45 mg, 0.06 mmol, 0.3 equiv) in $PhCF_3$ (1 mL). Purification was accomplished via flash column chromatography with appropriate eluents (EtOAc : hexane = 1 : 15) to give the product **3t** as a colorless gel (60 mg, 0.191 mmol) in 90% yield. **IR (neat)** 1707, 1684, 1448, 1349. 1002 cm^{-1} ; **^{1}H NMR** (500 MHz, $CDCl_3$) δ 7.92 (d, *J* = 7.4 Hz, 2H), 7.54 (t, *J* = 7.4 Hz, 1H), 7.43 (t, *J* = 7.7 Hz, 2H), 5.66 (d, *J* = 15.8 Hz, 1H), 5.58 (dt, *J* = 15.7, 6.5 Hz, 1H), 3.45 (d, *J* = 17.8 Hz, 1H), 3.36 (d, *J* = 17.8 Hz, 1H), 2.23 (s, 3H), 2.03 (q, *J* = 7.0 Hz, 2H), 1.39–1.25 (m, 13H), 0.87 (t, *J* = 5.0 Hz, 3H); **^{13}C{^{1}H} NMR** (125 MHz, $CDCl_3$) δ 211.1. 197.7, 136.9, 133.0, 132.8, 131.3, 128.5, 128.0, 51.2 (-**C**), 48.2 (-**C**H_2), 32.7 (-**C**H_2), 31.8 (-**C**H_2), 29.2 (-**C**H_2), 29.05 (-**C**H_2), 29.02 (-**C**H_2), 26.3, 22.6 (-**C**H_2), 21.8, 14.1; **HRMS (ESI)** m/z Calculated for $C_{21}H_{30}O_2$ $[M+Na]^+$: 337.2138; found: 337.2141. **Rf**: 0.72 (EtOAc : hexane = 1 : 3). **HPLC Chiralcel OD–H** (hexanes/ i-PrOH = 99:1, 1.0 mL/min, UV-254 detector, tr (major) = 9.2 min, tr (minor) = 8.7 min), **$[\alpha]_D^{20}$** = -24.2 (c = 0.03, acetone).

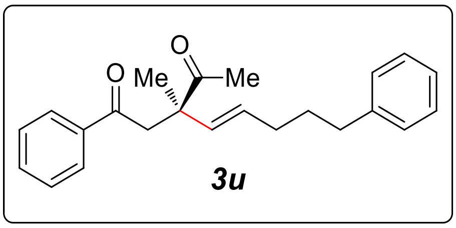

Following GP4, the product was synthesized from diketone **1a** (40 mg, 0.21 mmol, 1.0 equiv), potassium (*E*)-(5-phenylpent-1-enyl)trifluoroborate (106 mg, 0.42 mmol, 2.0 equiv,), (*R*)–3,3′–$(C_7F_7)_2$–BINOL (48.0 mg, 0.06 mmol, 0.3 equiv) in $PhCF_3$ (1 mL). Purification was accomplished via flash column chromatography with appropriate eluents (EtOAc : hexane = 1 :

10) to give the product **3u** as a colorless gel (71 mg, 0.21 mmol) in 99% yield. **IR (neat)** 1705, 1683, 1448, 1349. 1224 cm^{-1}; **^{1}H NMR** (500 MHz, $CDCl_3$) δ 7.93 (d, *J* = 7.7 Hz, 2H), 7.54 (t, *J* = 7.3 Hz, 1H), 7.44 (t, *J* = 7.5 Hz, 2H), 7.27 (t, *J* = 7.4 Hz, 2H), 7.19–7.15 (m, 3H), 5.70 (d, *J* = 15.8 Hz, 1H), 5.63–5.57 (m, 1H), 3.46 (d, *J* = 17.8 Hz, 1H), 3.37 (d, *J* = 17.8 Hz, 1H), 2.60 (t, *J* = 7.5 Hz, 2H), 2.24 (s, 3H), 2.11–2.07 (m, 2H), 1.70 (p, *J* = 7.5 Hz, 2H), 1.40 (s, 3H); **^{13}C{^{1}H} NMR** (125 MHz, $CDCl_3$) δ 210.9. 197.6, 142.1, 136.8, 133.4, 133.1, 130.6, 128.5, 128.3, 128.2, 127.9, 125.7, 51.1 (-**C**), 48.1 (-**C**H_2), 35.3 (-**C**H_2), 32.2 (-**C**H_2), 30.9 (-**C**H_2), 26.3 (-**C**H_3), 21.8 (-**C**H_3); **HRMS (ESI)** m/z Calculated for $C_{23}H_{26}O_2$ $[M+Na]^+$: 357.1825; found: 357.1827. **Rf**: 0.70 (EtOAc : hexane = 1 : 3). **HPLC Chiralcel AD–H** (hexanes/ i-PrOH = 98:2, 1.0 mL/min, UV-254 detector, tr (major) = 12.9 min, tr (minor) = 12.0 min), **$[\alpha]_D^{20}$** = -17.8 (c = 0.035, acetone).

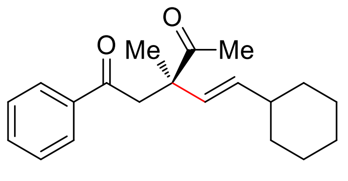

3v

Following GP4, the product was synthesized from diketone **1a** (40 mg, 0.212 mmol, 1.0 equiv), potassium (*E*)–(cyclohexanyl)ethenyl trifluoroborate (90 mg, 0.42 mmol, 2.0 equiv,), (*R*)–3,3′–$(C_7F_7)_2$–BINOL (60.0 mg, 0.08 mmol). Purification was accomplished via flash column chromatography with appropriate eluents (EtOAc : hexane = 1 : 20) to give the product **3v** as a colorless gel (62 mg, 0.208 mmol) in 98% yield. **IR (neat)** 2922, 1707, 1684, 1597, 1448, 1349 cm^{-1} ; **^{1}H NMR** (500 MHz, $CDCl_3$) δ 7.93 (d, *J* = 5.0 Hz, 2H), 7.55 (t, *J* = 5.0 Hz, 1H), 7.44 (t, *J* = 5.0 Hz, 2H), 5.62 (d, *J* = 20.0 Hz, 1H), 5.52 (dd, *J* = 20.0, 5.0 Hz, 1H), 3.44 (d, *J* = 20.0 Hz, 1H), 3.35 (d, *J* = 20.0 Hz, 1H), 2.22 (s, 3H), 1. 98–1.92 (m, 1H), 1.72–1.63 (m, 5H), 1.39 (s, 3H), 1.26–0.99 (m, 5H); **^{13}C{^{1}H} NMR** (125 MHz, $CDCl_3$) δ 211.2. 197.8, 136.9, 133.1, 130.5, 128.5, 128.0, 51.1 (-**C**), 48.1 (-**C**H_2), 40.9, 32.9 (-**C**H_2), 26.2, 26.0 (-**C**H_2), 25.9 (-**C**H_2), 21.7; **HRMS (ESI)** m/z Calculated for $C_{20}H_{26}O_2$ $[M+Na]^+$: 321.1825; found: 321.1826. **Rf**: 0.75 (EtOAc : hexane = 1 : 3). **HPLC Chiralcel OD–H** (hexanes/ i-PrOH = 99:1, 1.0 mL/min, UV-254 detector, tr (major) = 11.0 min, tr (minor) = 10.2 min), **$[\alpha]_D^{20}$** = -32.8 (c = 0.031, acetone).

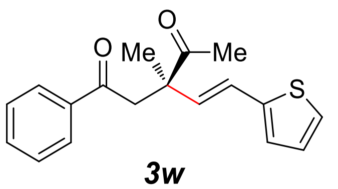

3w

Following GP4, the product was synthesized from diketone **1a** (40 mg, 0.21 mmol, 1.0 equiv), potassium (*E*)–(thienyl)ethenyl trifluoroborate (90 mg, 0.42 mmol, 2.0 equiv,), (*R*)–3,3′–$(C_7F_7)_2$–BINOL (47.0 mg, 0.06 mmol, 0.3 equiv) in PhMe (1 mL). Purification was accomplished via flash column chromatography with appropriate eluents (EtOAc : hexane = 1 : 10) to give the product **3w** as a colorless gel (62 mg, 0.20 mmol) in 98% yield. **IR (neat)** 1704, 1681, 1447. 1349 cm^{-1} ; **^{1}H NMR** (500 MHz, $CDCl_3$) δ 7.94 (d, *J* = 7.9 Hz, 2H), 7.56 (t, *J* = 7.5 Hz, 1H), 7.45 (t, *J* = 7.7 Hz, 2H), 7.29–7.27 (m, 1H), 7.21 (d, *J* = 5.0 Hz, 1H), 7.16–7.15 (m, 1H), 6.55 (d, *J* = 16.3 Hz, 1H), 6.35 (d, *J* = 16.2 Hz, 1H), 3.55 (d, *J* = 17.9 Hz, 1H), 3.48 (d, *J* = 17.9 Hz, 1H), 2.30 (s, 3H), 1.52 (s, 3H); **^{13}C{^{1}H} NMR** (125 MHz, $CDCl_3$) δ 210.5. 197.4, 139.3, 136.6, 133.2, 132.5, 128.5, 128.0, 126.2, 124.7, 124.2, 122.2, 51.3 (-**C**), 48.4 (-**C**H_2), 26.6 (-**C**H_3), 21.6 (-**C**H_3); **HRMS**

(ESI) m/z Calculated for $C_{18}H_{18}O_2S$ $[M+Na]^+$: 321.0920; found: 321.0922. **Rf**: 0.674 (EtOAc : hexane = 1 : 3). **HPLC Chiralcel AS–H** (hexanes/ i-PrOH = 92:8, 0.7 mL/min, UV-254 detector, tr (major) = 19.3 min, tr (minor) = 24.2 min), **$[\alpha]_D^{20}$** = -67.7 (c = 0.031, acetone)..

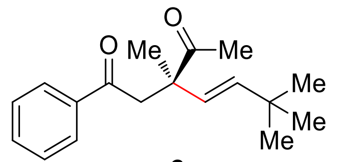

3x

Following GP4, the product was synthesized from diketone **1a** (40 mg, 0.21 mmol, 1.0 equiv), potassium (3,3-dimethylbut-1-en-1-yl)trifluoroborate (75 mg, 2.0 equiv), (*R*)–3,3′–$(C_7F_7)_2$–BINOL (61.0 mg, 0.08 mmol, 0.04 equiv). Purification was accomplished via flash column chromatography with appropriate eluents (EtOAc : hexane = 1 : 20) to give the product **3x** as a colorless gel (47 mg, 0.204 mmol) in 81% yield. **IR (neat)** 2957, 1707, 1685, 1597, 1461, 1349 cm^{-1} ; **^{1}H NMR** (500 MHz, $CDCl_3$) δ 7.92 (d, *J* = 5.0 Hz, 2H), 7.53 (t, *J* = 5.0 Hz, 1H), 7.43 (t, *J* = 5.0 Hz, 2H), 5.59 (d, *J* = 20.0 Hz, 1H), 5.53 (d, *J* = 20.0 Hz, 1H), 3.43 (d, *J* = 20.0 Hz, 1H), 3.35 (d, *J* = 20.0 Hz, 1H), 2.20 (s, 3H), 1.39 (s, 3H), 0.98 (s, 9H); **^{13}C{^{1}H} NMR** (125 MHz, $CDCl_3$) δ 211.1. 197.8, 141.9, 137.0, 133.0, 128.5, 128.0, 127.7, 50.9 (-**C**), 48.0 (-**C**H_2), 33.0 (-**C**), 29.4 (-**C**H_3), 26.1 (-**C**H_3), 21.6 (-**C**H_3); **HRMS (ESI)** m/z Calculated for $C_{18}H_{24}O_2$ $[M+Na]^+$: 295.1669; found: 295.1672. **Rf**: 0.75 (EtOAc : hexane = 1 : 3). **HPLC Chiralcel OD–H** (hexanes/ i-PrOH = 99:1, 1.0 mL/min, UV-254 detector, tr (major) = 9.1 min, tr (minor) = 8.6 min), **$[\alpha]_D^{20}$** = -43.7 (c = 0.0235, acetone)..

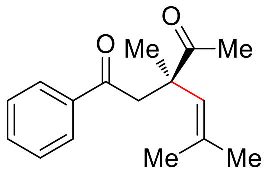

3y

Following GP4, the product was synthesized from diketone **1a** (40 mg, 0.212 mmol, 1.0 equiv), potassium 2–methyl–1–propenyltrifluoroborate (68 mg, 2.0 equiv,), (*R*)–3,3′–$(C_7F_7)_2$–BINOL (30.0 mg, 0.04 mmol). Purification was accomplished via flash column chromatography with appropriate eluents (EtOAc : hexane = 1 : 10) to give the product **3y** as a colorless gel (50 mg, 0.204 mmol) in 94% yield. **IR (neat)** 2930, 1705, 1685, 1596, 1448, 1348 cm^{-1} ; **^{1}H NMR** (500 MHz, $CDCl_3$) δ 7.92 (d, *J* = 5.0 Hz, 2H), 7.53 (t, *J* = 5.0 Hz, 1H), 7.43 (t, *J* = 5.0 Hz, 2H), 5.34 (s, 1H), 3.44 (d, *J* = 20.0 Hz, 1H), 3.39 (d, *J* = 20.0 Hz, 1H), 2.17 (s, 3H), 1.67 (s, 3H), 1.52 (s, 3H), 1.45 (s, 3H); **^{13}C{^{1}H} NMR** (125 MHz, $CDCl_3$) δ 210.8. 198.4, 137.6, 135.4, 132.9, 128.4, 127.9, 127.8, 51.0, 45.9, 27.1, 25.6, 23.9, 18.5; **HRMS (ESI)** m/z Calculated for $C_{16}H_{20}O_2$ $[M+Na]^+$: 267.1356; found: 267.1362. **Rf**: 0.68 (EtOAc : hexane = 1 : 3). HPLC Chiralcel OD–H (hexanes/ i-PrOH = 95:5, 1.0mL/min, UV-254 detector). **HPLC Chiralcel OD–H** (hexanes/ i-PrOH = 95:5, 1.0 mL/min, UV-254 detector, tr (major) = 6.9 min, tr (minor) = 7.3 min), **$[\alpha]_D^{20}$** = -14.7 (c = 0.025, acetone).

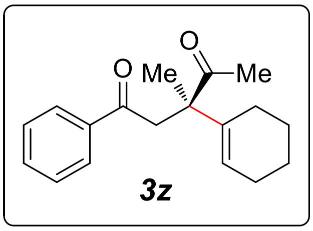

Following GP4, the product was synthesized from diketone **1a** (40 mg, 0.21 mmol), potassium cyclohex–1–en–1–yltrifluoroborate (78 mg, 0.41 mmol, 2.0 equiv,), (*R*)–3,3′–$(C_7F_7)_2$–BINOL (64 mg, 0.09 mmol, 0.4 equiv) in $PhCF_3$ (1 mL). Purification was accomplished via flash column chromatography with appropriate eluents (EtOAc : hexane = 1 : 10) to give the product **3z** as a colorless gel (25 mg) in 47% yield. **IR (neat)** 2929, 1707, 1684, 1448. 1351 cm^{-1} ; ^{1}**H NMR** (400 MHz, $CDCl_3$) δ 7.93 (d, *J* = 8.0 Hz, 2H), 7.55 (t, *J* = 5.0 Hz, 1H), 7.44 (t, *J* = 8.0 Hz, 2H), 5.72–5.70 (m, 1H), 3.45 (d, *J* = 16.0 Hz, 1H), 3.34 (d, *J* = 16.0 Hz, 1H), 2.13 (s, 3H), 2.07–2.06 (m, 2H), 1.93–1.83 (m, 2H), 1.54–1.48 (m, 4H), 1.44 (s, 3H); ^{13}**C{^{1}H} NMR** (100 MHz, $CDCl_3$) δ 210.3. 198.8, 137.7, 132.9, 128.5, 128.0, 124.0, 55.3, 44.2, 25.7, 25.3, 25.2, 23.0, 21.9, 20.4; **HRMS (ESI)** m/z Calculated for $C_{18}H_{22}O_2$ $[M+H]^+$: 271.1693; found: 271.1698. **Rf**: 0.68 (EtOAc : hexane = 1 : 3). HPLC Chiralcel ID (hexanes/ i-PrOH = 92:8, 1.0mL/min, UV-254 detector). **HPLC Chiralcel ID** (hexanes/ i-PrOH = 98:2, 1.0 mL/min, UV-254 detector, tr (major) = 15.0 min, tr (minor) = 16.6 min), $[\alpha]_D^{20}$ = 64.6 (c = 0.005, acetone)..

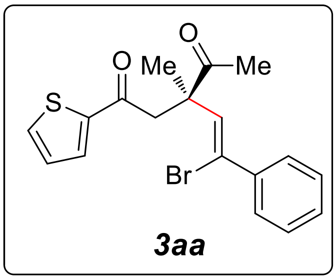

Following GP4, the product was synthesized from diketone **1f** (23 mg, 0.12 mmol), corresponding potassium trifluoroborate (40 mg, 0.16 mmol, 1.5 equiv,), (*R*)–3,3′–$(C_7F_7)_2$–BINOL (24 mg, 0.033 mmol, 0.28 equiv) in PhMe (1 mL). Purification was accomplished via flash column chromatography with appropriate eluents (EtOAc : hexane = 1 : 10) to give the product **3aa** as a colorless gel (23 mg) in 56% yield. **IR (neat)** 1708, 1680, 1450, 1335 cm^{-1} ; ^{1}**H NMR** (400 MHz, $CDCl_3$) δ 7.8 (d, *J* = 1.0, 4.0 Hz, 1H), 7.63 (d, *J* = 1.0, 4.0 Hz, 1H), 7.49–7.47 (m, 2H), 7.34–7.31 (m, 3H), 7.13–7.11 (m, 1H), 6.75 (s, 1H), 3.94 (d, *J* = 16.0 Hz, 1H), 3.58 (d, *J* = 16.0 Hz, 1H) , 2.33 (s, 3H), 1.60 (s, 3H); ^{13}**C{^{1}H} NMR** (100 MHz, $CDCl_3$) δ 209.0. 191.2, 144.7, 139.9, 134.8, 133.8, 132.3, 128.9, 128.3, 128.2, 127.6, 125.3, 53.0, 44.1, 25.9, 24.1; **HRMS (ESI)** m/z Calculated for $C_{18}H_{17}BrO_2S$ $[M+Na]^+$: 399.0025; found: 399.0024. **Rf**: 0.24 (EtOAc : hexane = 1 : 3). **HPLC Chiralcel ID** (hexanes/ i-PrOH = 90:10, 1.0 mL/min, UV-254 detector, tr (major) = 6.3 min, tr (minor) = 6.7 min).

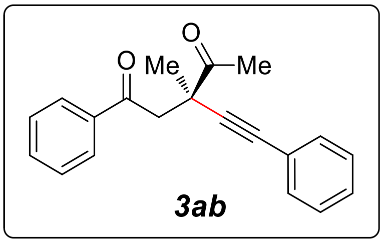

Following GP4, the product was synthesized from diketone **1a** (40 mg, 0.21 mmol, 1.0 equiv), potassium (2–phenylethynyl)trifluoroborate (92 mg, 2.0 equiv), (*R*)–3,3′–$(C_7F_7)_2$–BINOL (30.0 mg, 0.042 mmol). Purification was accomplished via flash column chromatography with appropriate eluents (EtOAc : hexane = 1 : 10) to give the product **3ab** as a colorless gel (32 mg, 0.110 mmol) in 52% yield. **IR (neat)** 1739, 1721, 1685, 1352 cm^{-1}; ^{1}**H NMR** (400 MHz, $CDCl_3$) δ 7.96 (d, *J* = 8.0 Hz, 2H), 7.57 (t, *J* = 8.0 Hz, 1H), 7.46 (t, *J* = 8.0 Hz, 2H), 7.38–7.36 (m, 2H), 7.28–7.26 (m, 3H), 3.76 (d, *J* = 20.0 Hz, 1H), , 3.55 (d, *J* = 20.0 Hz, 1H), 2.64 (s, 3H), 1.56 (s, 3H); ^{13}**C{^{1}H} NMR** (100 MHz, $CDCl_3$) δ 208.5, 196.2,

136.3, 133.2, 131.5, 128.5, 128.1, 128.1, 128.0, 122.9, 91.3, 84.0, 49.0, 45.0, 27.3, 25.9; **HRMS (APCI)** m/z Calculated for $C_{20}H_{18}O_2$ $[M+H]^+$: 291.1380; found: 291.1380. **Rf**: 0.63 (EtOAc : hexane = 1 : 3). **HPLC Chiralcel ID** (hexanes/ i-PrOH = 92:8, 1.0 mL/min, UV-254 detector, tr (major) = 5.5 min, tr (minor) = 5.2 min).

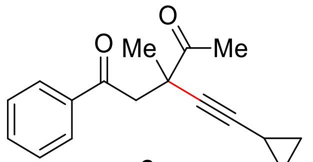

3ac

Following GP4, the product was synthesized from diketone **1a** (24 mg, 0.13 mmol, 1.0 equiv), potassium (2–cyclopropylethynyl)trifluoroborate (150 mg, 2.0 equiv), (*R*)–3,3′–$(C_7F_7)_2$–BINOL (30.0 mg, 0.042 mmol). Purification was accomplished via flash column chromatography with appropriate eluents (EtOAc : hexane = 1 : 10) to give the product **3ac** as a colorless gel (28 mg, 0.110 mmol) in 86% yield. **IR (neat)** 2976, 2238, 1714, 1684, 1448, 1339 cm^{-1}; **^{1}H NMR** (400 MHz, $CDCl_3$) δ 7.93 (d, *J* = 8.0 Hz, 2H), 7.56 (t, *J* = 8.0 Hz, 1H), 7.45 (t, *J* = 8.0 Hz, 2H), 3.61 (d, *J* = 16.0 Hz, 1H), 3.40 (d, *J* = 16.0 Hz, 1H), 2.52 (s, 3H), 1.41 (s, 3H), 1.23–1.16 (m, 1H), 0.73–0.68 (m, 2H), 0.60–0.56 (m, 2H); **^{13}C{^{1}H} NMR** (100 MHz, $CDCl_3$) δ 209.2, 196.5, 136.5, 133.2, 128.5, 128.1, 87.3, 77.2, 49.0, 44.5, 27.1, 26.1, 8.14, 8.11, -0.5.; **Rf**: 0.83 (EtOAc : hexane = 1 : 3); **HRMS (ESI)** m/z Calculated for $C_{17}H_{18}O_2$ $[M+H]^+$: 255.1380; found: 255.1382.

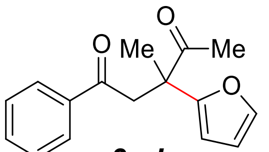

3ad

Following GP4, the product was synthesized from from diketone **1a** (40 mg, 0.212 mmol, 1.0 equiv), potassium furan-2-trifluoroborate (79 mg, 2.0 equiv), (*R*)–3,3′–$(C_7F_7)_2$–BINOL (30.0 mg, 0.04 mmol, 0.2 equiv). Purification was accomplished via flash column chromatography with appropriate eluents (EtOAc : hexane = 1 : 8) to give the product **3ad** as a colorless gel (42 mg, 0.164 mmol) in 77% yield. **IR (neat)** 1739, 1684, 1352, 1217 cm^{-1}; **^{1}H NMR** (400 MHz, $CDCl_3$) δ 7.94 (d, *J* = 8.0 Hz, 2H), 7.56 (t, *J* = 8.0 Hz, 1H), 7.45 (t, *J* = 8.0 Hz, 2H), 7.36 (s, 1H), 6.33 (s, 1H), 6.20 (d, *J* = 4.0 Hz, 1H), 3.88 (d, *J* = 16.0 Hz, 1H), 3.55 (d, *J* = 16.0 Hz, 1H), 2.19 (s, 3H), 1.70 (s, 3H); **^{13}C{^{1}H} NMR** (100 MHz, $CDCl_3$) δ 207.6, 197.3, 155.5, 142.0, 136.8, 133.1, 128.5, 128.0, 110.5, 106.5, 50.6, 45.5, 25.9, 21.1; **Rf**: 0.60 (EtOAc : hexane = 1 : 3); **HRMS (APCI)** m/z Calculated for $C_{16}H_{16}O_3$ $[M+H]^+$: 257.1172; found: 257.1170.

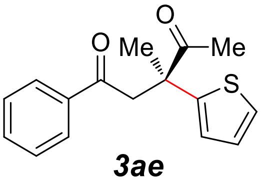

3ae

Following GP4, the product was synthesized from from diketone **1a** (40 mg, 0.21 mmol, 1.0 equiv), potassium thiophene-2-trifluoroborate (71 mg, 0.37 mmol, 1.8 equiv), (*R*)–3,3′–$(C_7F_7)_2$–BINOL (30 mg, 0.04 mmol, 0.2 equiv) in $PhCF_3$ (1.0 mL). Purification was accomplished via flash column chromatography with appropriate eluents (EtOAc : hexane = 1 : 10) to give the product **3ae** as a colorless gel (45 mg, 0.165 mmol) in 78% yield. **IR (neat)** 1708, 1682, 1350, 1227 cm^{-1}; **^{1}H NMR** (400 MHz, $CDCl_3$) δ 7.94 (d, *J* = 8.0 Hz, 2H), 7.56 (t, *J* = 8.0 Hz, 1H), 7.45 (t, *J* = 8.0 Hz, 2H), 7.26–7.25 (m, 1H), 7.00–6.98 (m, 2H), 3.99 (d, *J* = 20.0 Hz, 1H), 3.55 (d, *J* = 20.0 Hz,

1H), 2.24 (s, 3H), 1.87 (s, 3H); **$^{13}C\{^1H\}$ NMR** (100 MHz, $CDCl_3$) δ 208.2, 197.1, 147.0, 136.7, 133.2, 128.5, 128.0, 127.1, 124.8, 124.5, 51.5, 49.6, 25.6, 23.5.; **HRMS (APCI)** m/z Calculated for $C_{16}H_{16}O_2S$ $[M+H]^+$: 273.0944; found: 273.0946. **Rf**: 0.62 (EtOAc : hexane = 1 : 3). **HPLC Chiralcel ID** (hexanes/ i-PrOH = 90:10, 1.0 mL/min, UV-254 detector, tr (major) = 8.6 min, tr (minor) = 7.7 min).

Transformations of 1,4-diones

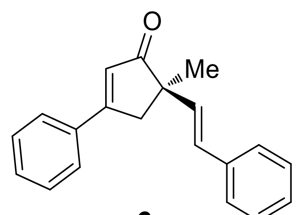

9

To a solution of **3a** (50 mg, 0.2 mmol) in MeOH (1.0 mL) was added 1.0 M NaOH (aq) (0.04 mL, 0.04 mmol, 1.1 equiv) at room temperature under air. The resulting mixture was allowed to stir at 70 °C for another 16 h. After the reaction completed, the solvent was removed under reduced pressure and the crude mixture was purified via flash column chromatography with appropriate eluents (EtOAc : hexane = 1 : 10) to give the product **9** as a white solid (46 mg, 0.18 mmol) in 98% yield. **IR (neat)** 1688, 1595, 1449, 1192 cm^{-1}.; **^{1}H NMR** (500 MHz, $CDCl_3$) δ 7.71–7.69 (m, 2H), 7.51–7.46 (m, 3H), 7.36 (d, *J* = 10.0 Hz, 2H), 7.30–7.26 (m, 2H), 7.20 (t, *J* = 7.5 Hz, 1H), 6.56–6.51 (m, 2H), 6.31 (d, *J* = 16.0 Hz, 1H), 3.34 (d, *J* = 18.0 Hz, 1H), 3.04 (d, *J* = 18.0 Hz, 1H), 1.46 (s, 3H); **^{13}C{^{1}H} NMR** (150 MHz, $CDCl_3$) δ 210.3, 170.7, 136.9, 133.8, 132.7, 131.4, 128.9, 128.5, 127.4, 126.9, 126.2, 124.8, 50.5, 43.9, 24.1; **HRMS (ESI)** m/z Calculated for $C_{20}H_{18}O$ $[M+H]^+$: 275.1430; found: 275.1427. **Rf**: 0.54 (EtOAc : hexane = 1 : 3). **HPLC Chiralcel ID** (hexanes/ i-PrOH = 90:10, 1.0 mL/min, UV-254 detector, tr (major) = 16.5 min, tr (minor) = 10.5 min), **$[\alpha]_D^{20}$** = -7.8 (c = 0.02, acetone)..

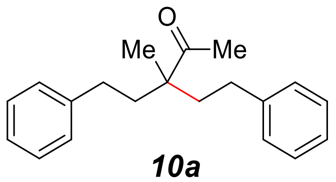

10a

To a solution of **3a** (56 mg, 0.19 mmol) in MeOH (2.0 mL) was added Pd/C (20 mg, 0.1 equiv) at room temperature. The resulting mixture was allowed to stir at room temperature (20 °C) for another 18 h under hydrogen balloon. After the reaction completed, the mixture was filtered, and the solvent was removed under reduced pressure to give the desired product **10a** (45 mg, 0.16 mmol) in 84% yield without further purification. **IR (neat)** 2930, 1770, 1497, 1453, 1354 cm^{-1}.; **^{1}H NMR** (400 MHz, $CDCl_3$) δ 7.30–7.27 (m, 2H), 7.20–7.16 (m, 3H), 2.52 (td, *J* = 12.0, 4.0 Hz, 1H), 2.38 (td, *J* = 12.0, 4.0 Hz, 1H), 2.14 (s, 3H), 1.90 (td, *J* = 12.0, 4.0 Hz, 1H), 1.73 (td, *J* = 12.0, 4.0 Hz, 1H), 1.65–1.59 (m, 1H), 1.50 (td, *J* = 12.0, 4.0 Hz, 1H), 1.32–1.20 (m, 5H), 1.17 (s, 3H), 1.13–1.08 (m, 1H), 0.88 (t, *J* = 8.0 Hz, 3H); **^{13}C{^{1}H} NMR** (100 MHz, $CDCl_3$) δ 213.1, 142.0, 128.5, 128.2, 125.9, 51.4, 40.5, 30.8, 25.4, 20.7; **HRMS (ESI)** m/z Calculated for $C_{20}H_{24}O$ $[M+Na]^+$: 303.1719; found: 303.1717. **Rf**: 0.48 (EtOAc : hexane = 1 : 10).

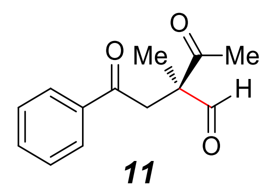

11

A solution of **3a** (50 mg, 0.17 mmol) in DCM (2.0 mL) and MeOH (1.0 mL) was purged with O_3 at -78 °C. The resulting mixture was allowed to stir at the same temperature for another 1.5 h. The reaction was monitored with TLC. After the completion of the reaction, the mixture was purged under O_2 for 20 min followed by the addition of Me_2S (12 μL). The resulting mixture was allowed to stir for overnight. The solvent was removed under reduced pressure to give the desired product **11** (29 mg, 0.133 mmol) in 78% yield without further purification. **^{1}H NMR** (400 MHz, $CDCl_3$) δ 9.96 (s, 1H), 7.95 (d, *J* = 8.0 Hz, 2H), 7.60 (t, *J* = 8.0 Hz, 1H), 7.48 (t,

J = 8.0 Hz, 2H), 3.67 (s, 2H), 2.32 (s, 3H), 1.44 (s, 3H); **^{13}C{^{1}H} NMR** (100 MHz, $CDCl_3$) δ 206.0, 200.4, 197.0, 135.7, 133.7, 128.7, 128.2, 61.2, 44.5, 27.2, 18.9; **HRMS (ESI)** m/z Calculated for $C_{13}H_{14}O_3$ $[M+Na]^+$: 241.0835; found: 241.0831. **Rf**: 0.26 (EtOAc : hexane = 1 : 3). **HPLC Chiralcel OD–H** (hexanes/ i-PrOH = 90:10, 1.0 mL/min, UV-254 detector, tr (major) = 13.8 min, tr (minor) = 13.1 min).

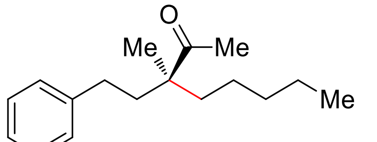

10q

To a solution of **3q** (44 mg, 0.17 mmol) in MeOH (mL) was added Pd/C (18 mg, 0.1 equiv) at room temperature (20 °C). The resulting mixture was purged by hydrogen balloon was allowed to stir at room temperature for another 18 h under H_2. After the reaction completed, the mixture was filtered, and the solvent was removed under reduced pressure to give the desired product **10q** (37 mg) in 89% yield without further purification. **IR (neat)** 2930, 1701, 1454, 1353 cm^{-1}.; **^{1}H NMR** (500 MHz, $CDCl_3$) δ 7.30–7.27 (m, 2H), 7.20–7.16 (m, 3H), 2.52 (td, *J* = 13.0, 5.0 Hz, 1H), 2.38 (td, *J* = 13.0, 5.0 Hz, 1H), 2.14 (s, 3H), 1.90 (td, *J* = 13.0, 5.0 Hz, 1H), 1.73 (td, *J* = 13.0, 5.0 Hz, 1H), 1.65–1.59 (m, 1H), 1.52–1.46 (m, 1H), 1.32–1.20 (m, 5H), 1.17 (s, 3H), 1.13–1.08 (m, 1H), 0.88 (t, *J* = 10.0 Hz, 3H); **^{13}C{^{1}H} NMR** (125 MHz, $CDCl_3$) δ 213.6, 142.2, 128.4, 128.2, 125.9, 51.4, 40.5, 38.6, 32.4, 30.9, 25.4, 24.0, 22.5, 20.5, 14.0; **HRMS (ESI)** m/z Calculated for $C_{20}H_{20}O_2$ $[M+Na]^+$: 269.1876; found: 269.1873. **Rf**: 0.48 (EtOAc : hexane = 1 : 10). **HPLC Chiralpak AD–H** (hexanes/ i-PrOH = 99:1, 0.5 mL/min, UV-190 detector, tr (major) = 11.2 min, tr (minor) = 10.3 min), **$[\alpha]_D^{20}$** = -17.7 (c = 0.01, acetone)..

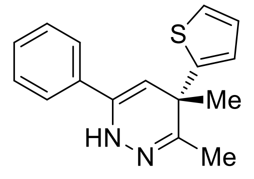

12

To a solution of **3x** (44 mg, 0.16 mmol) in toluene (1 mL) was added hydrazine hydrate (50 μL, 5.0 equiv) at room temperature (20 °C). The resulting mixture was allowed to stir at 70 °C for 1 h. After the reaction completed, the solvent was removed under reduced pressure and dissolved in DCE (1 mL) for the next step without further purification. To the above solution was added potassium *tert*–butoxide (60 mg, 3.0 equiv) at the room temperature. The mixture was stirred at 70 °C for another 16 h. After the reaction completed, the solvent was removed under reduced pressure and the residue was purified by flash column chromatography with appropriate eluents (EtOAc : hexane = 1 : 10) to give title product **12** as a colorless gel (33 mg, 0.12 mmol) in 76% yield. **IR (neat)** 1738, 1654, 1482, 1371 cm^{-1}.; **^{1}H NMR** (500 MHz, $CDCl_3$) δ 7.47–7.36 (m, 6H), 7.26–7.19 (m, 2H), 6.98–6.98 (m, 2H), 4.82 (s, 1H), 1.93 (s, 3H), 1.66 (s, 3H); **^{13}C{^{1}H} NMR** (125 MHz, $CDCl_3$) δ 152.9, 145.1, 137.7, 134.8, 128.8, 128.7, 126.8, 125.4, 124.7, 123.6, 100.4, 39.1, 26.6, 18.5; **HRMS (ESI)** m/z Calculated for $C_{16}H_{16}N_2S$ $[M+H]^+$: 269.1107; found: 269.1106. **Rf**: 0.35 (EtOAc : hexane = 1 : 3). **HPLC Chiralcel OD–H** (hexanes/ i-PrOH = 90:10, 1.0 mL/min, UV-254 detector, tr (major) = 8.6 min, tr (minor) = 7.1 min).

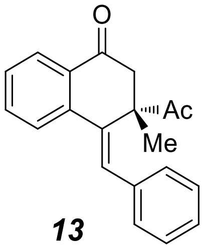

13

To a flame-dried round-bottomed flask equipped with a magnetic stir bar was added pallidum acetate ($Pd(OAc)_2$, 10 mol%), triphenyl phosphine (PPh_3, 15 mol%) and potassium carbonate (K_2CO_3, 1.1 equiv) under N_2. The solution of **3e** (38 mg, 0.10 mmol) in degassed PhMe (2.0 mL) was then added to the above mixture at room temperature (20 °C). The resulting solution was allowed to stir at 120 °C for another 6 h. After the reaction completed, the solvent was removed under reduced pressure and the residue was purified by flash column chromatography with appropriate eluents (EtOAc : hexane = 1 : 10) to give title product **13** as a colorless gel (23 mg, 0.079 mmol) in 77% yield. **IR (neat)** 1709, 1680, 1450, 1355 cm^{-1}.; **^{1}H NMR** (500 MHz, $CDCl_3$) δ 8.04 (dd, *J* = 7.8, 1.1 Hz, 1H), 7.68 (d, *J* = 7.8 Hz, 1H), 7.59 (td, *J* = 7.8, 1.2 Hz, 1H), 7.45–7.42 (m, 2H), 7.39–7.33 (m, 3H), 7.31–7.29 (m, 2H), 3.14 (d, *J* = 16.6, 5.0 Hz, 1H), 2.51 (d, *J* = 16.6 Hz, 1H), 1.96 (s, 3H), 1.18 (s, 3H); **^{13}C{^{1}H} NMR** (125 MHz, $CDCl_3$) δ 208.7, 195.5, 142.0, 138.4, 137.4, 134.2, 131.2, 130.9, 128.7, 128.0, 127.7, 126.8, 126.3, 57.0, 48.7, 25.6, 23.7; **HRMS (ESI)** m/z Calculated for $C_{20}H_{18}O_2$ $[M+Na]^+$: 313.1199; found: 313.1198. **Rf**: 0.42 (EtOAc : hexane = 1 : 3). **HPLC Chiralcel ID** (hexanes/ i-PrOH = 90:10, 1.0 mL/min, UV-254 detector, tr (major) = 20.9 min, tr (minor) = 17.6 min), **$[\alpha]_D^{20}$** = -282.8 (c = 0.006, acetone).

Crystal structure of compound 9

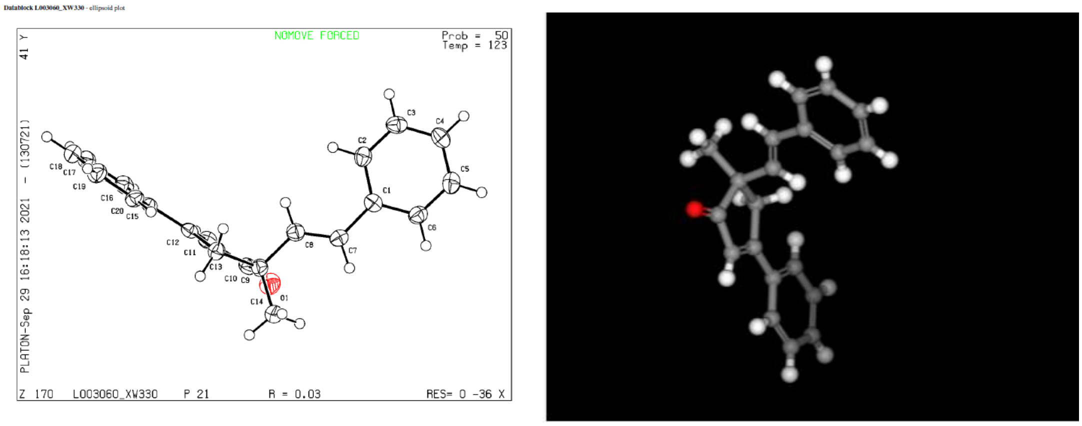

Supplementary Figure 3. Crystal data and structure of compound 9 (ellipsoids at 50% probability)

CCDC deposition number 2121715

Crystal data and structure refinement for compound 8.

Identification code	L003060_XW330	
Empirical formula	C20 H18 O	
Formula weight	274.34	
Temperature	123(2) K	
Wavelength	1.54178 Å	
Crystal system	Monoclinic	
Space group	P 21	
Unit cell dimensions	a = 6.45490(10) Å	α= 90°.
	b = 9.6916(2) Å	β= 97.1360(10)°.
	c = 12.0710(2) Å	γ = 90°.
Volume	749.29(2) Å^3	
Z	2	
Density (calculated)	1.216 Mg/m^3	
Absorption coefficient	0.564 mm^{-1}	
F(000)	292	
Crystal size	0.22 x 0.09 x 0.07 mm^3	
Theta range for data collection	3.690 to 65.596°.	
Index ranges	-7<=h<=6, -11<=k<=11, -14<=l<=13	
Reflections collected	11034	
Independent reflections	2482 [R(int) = 0.0270]	
Completeness to theta = 65.596°	96.2 %	
Absorption correction	Empirical	
Max. and min. transmission	0.7527 and 0.6184	
Refinement method	Full-matrix least-squares on F^2	
Data / restraints / parameters	2482 / 1 / 191	

Goodness-of-fit on F^2	1.074
Final R indices [I>2sigma(I)]	R1 = 0.0318, wR2 = 0.0794
R indices (all data)	R1 = 0.0319, wR2 = 0.0795
Absolute structure parameter	0.06(14)
Largest diff. peak and hole	0.134 and -0.159 e.Å^{-3}

Atomic coordinates (x 10^4) and equivalent isotropic displacement parameters (Å^{2}x 10^3)
for l003060_xw330. U(eq) is defined as one third of the trace of the orthogonalized U^{ij} tensor.

	x	y	z	U(eq)
O(1)	4208(2)	6503(2)	484(1)	40(1)
C(1)	3519(3)	7500(2)	4206(2)	28(1)
C(2)	3699(3)	6289(2)	4843(2)	31(1)
C(3)	2444(4)	6068(2)	5682(2)	33(1)
C(4)	1000(4)	7050(2)	5903(2)	35(1)
C(5)	833(4)	8263(2)	5295(2)	36(1)
C(6)	2070(3)	8479(2)	4458(2)	32(1)
C(7)	4690(3)	7741(2)	3253(2)	29(1)
C(8)	6136(3)	6929(2)	2922(2)	30(1)
C(9)	7189(3)	7080(2)	1871(2)	28(1)
C(10)	5872(3)	6158(2)	1001(2)	29(1)
C(11)	6953(3)	4847(2)	946(2)	28(1)
C(12)	8868(3)	4907(2)	1532(2)	25(1)
C(13)	9303(3)	6321(2)	2039(2)	28(1)
C(14)	7306(4)	8551(2)	1450(2)	35(1)
C(15)	10423(3)	3800(2)	1679(2)	25(1)
C(16)	10014(3)	2484(2)	1223(2)	30(1)
C(17)	11508(4)	1454(2)	1395(2)	34(1)
C(18)	13419(4)	1707(2)	2020(2)	37(1)
C(19)	13838(4)	3001(2)	2478(2)	35(1)
C(20)	12361(3)	4040(2)	2306(2)	29(1)

Bond lengths [Å] and angles [°] for compound 8.

O(1)-C(10)	1.220(3)
C(1)-C(6)	1.392(3)
C(1)-C(2)	1.400(3)
C(1)-C(7)	1.472(3)

C(2)-C(3)	1.390(3)
C(3)-C(4)	1.381(3)
C(4)-C(5)	1.383(3)
C(5)-C(6)	1.380(3)
C(7)-C(8)	1.319(3)
C(8)-C(9)	1.519(3)
C(9)-C(14)	1.519(3)
C(9)-C(13)	1.541(3)
C(9)-C(10)	1.549(3)
C(10)-C(11)	1.456(3)
C(11)-C(12)	1.347(3)
C(12)-C(15)	1.465(3)
C(12)-C(13)	1.513(3)
C(15)-C(20)	1.399(3)
C(15)-C(16)	1.401(3)
C(16)-C(17)	1.386(3)
C(17)-C(18)	1.386(3)
C(18)-C(19)	1.384(3)
C(19)-C(20)	1.385(3)

C(6)-C(1)-C(2)	117.61(19)
C(6)-C(1)-C(7)	119.15(18)
C(2)-C(1)-C(7)	123.14(18)
C(3)-C(2)-C(1)	120.9(2)
C(4)-C(3)-C(2)	120.2(2)
C(3)-C(4)-C(5)	119.53(19)
C(6)-C(5)-C(4)	120.2(2)
C(5)-C(6)-C(1)	121.5(2)
C(8)-C(7)-C(1)	126.41(19)
C(7)-C(8)-C(9)	127.04(19)
C(14)-C(9)-C(8)	114.53(17)
C(14)-C(9)-C(13)	114.33(18)
C(8)-C(9)-C(13)	109.00(16)
C(14)-C(9)-C(10)	111.13(17)
C(8)-C(9)-C(10)	104.21(17)
C(13)-C(9)-C(10)	102.50(16)
O(1)-C(10)-C(11)	127.7(2)
O(1)-C(10)-C(9)	124.3(2)

C(11)-C(10)-C(9)	107.98(17)
C(12)-C(11)-C(10)	110.59(18)
C(11)-C(12)-C(15)	127.00(19)
C(11)-C(12)-C(13)	111.33(18)
C(15)-C(12)-C(13)	121.65(17)
C(12)-C(13)-C(9)	105.20(16)
C(20)-C(15)-C(16)	118.48(19)
C(20)-C(15)-C(12)	119.77(19)
C(16)-C(15)-C(12)	121.74(18)
C(17)-C(16)-C(15)	120.21(19)
C(18)-C(17)-C(16)	120.7(2)
C(19)-C(18)-C(17)	119.7(2)
C(18)-C(19)-C(20)	120.2(2)
C(19)-C(20)-C(15)	120.8(2)

Symmetry transformations used to generate equivalent atoms:

Supplementary Table 6.

Anisotropic displacement parameters ($Å^2$x 10^3)for l003060_xw330. The anisotropic displacement factor exponent takes the form: $-2\pi^2[h^2a^{*2}U^{11} + ... + 2 h k a^* b^* U^{12}]$

	U^{11}	U^{22}	U^{33}	U^{23}	U^{13}	U^{12}
O(1)	34(1)	49(1)	35(1)	3(1)	-5(1)	7(1)
C(1)	29(1)	30(1)	25(1)	-4(1)	2(1)	-5(1)
C(2)	32(1)	30(1)	30(1)	-6(1)	2(1)	2(1)
C(3)	43(1)	31(1)	25(1)	1(1)	0(1)	-4(1)
C(4)	37(1)	42(1)	26(1)	-5(1)	8(1)	-4(1)
C(5)	35(1)	37(1)	36(1)	-5(1)	5(1)	5(1)
C(6)	38(1)	27(1)	32(1)	0(1)	2(1)	1(1)
C(7)	32(1)	24(1)	30(1)	1(1)	1(1)	1(1)
C(8)	30(1)	33(1)	28(1)	1(1)	3(1)	1(1)
C(9)	29(1)	30(1)	25(1)	0(1)	4(1)	4(1)
C(10)	27(1)	38(1)	23(1)	5(1)	3(1)	2(1)
C(11)	30(1)	32(1)	22(1)	-1(1)	0(1)	-2(1)
C(12)	28(1)	27(1)	20(1)	1(1)	6(1)	-3(1)
C(13)	26(1)	27(1)	31(1)	-2(1)	3(1)	0(1)
C(14)	43(1)	31(1)	33(1)	2(1)	11(1)	5(1)
C(15)	29(1)	26(1)	20(1)	2(1)	7(1)	-2(1)

C(16)	37(1)	30(1)	23(1)	-4(1)	5(1)	-4(1)
C(17)	50(1)	26(1)	30(1)	-3(1)	14(1)	0(1)
C(18)	40(1)	31(1)	42(1)	5(1)	14(1)	7(1)
C(19)	31(1)	32(1)	43(1)	5(1)	5(1)	0(1)
C(20)	29(1)	24(1)	33(1)	2(1)	3(1)	-2(1)

Supplementary Table 7.

Hydrogen coordinates (x 10^4) and isotropic displacement parameters (Å^{2}x 10^3) for l003060_xw330.

	x	y	z	U(eq)
H(2)	4692	5610	4700	37
H(3)	2581	5239	6105	40
H(4)	128	6893	6469	42
H(5)	-137	8950	5455	43
H(6)	1929	9315	4044	39
H(7)	4374	8561	2834	34
H(8)	6564	6169	3392	36
H(11)	6387	4058	550	34
H(13A)	10352	6816	1655	34
H(13B)	9829	6247	2843	34
H(14A)	7990	8558	770	53
H(14B)	8112	9117	2024	53
H(14C)	5892	8928	1287	53
H(16)	8708	2298	794	36
H(17)	11219	565	1081	41
H(18)	14436	995	2134	44
H(19)	15143	3177	2911	42
H(20)	12667	4928	2618	35

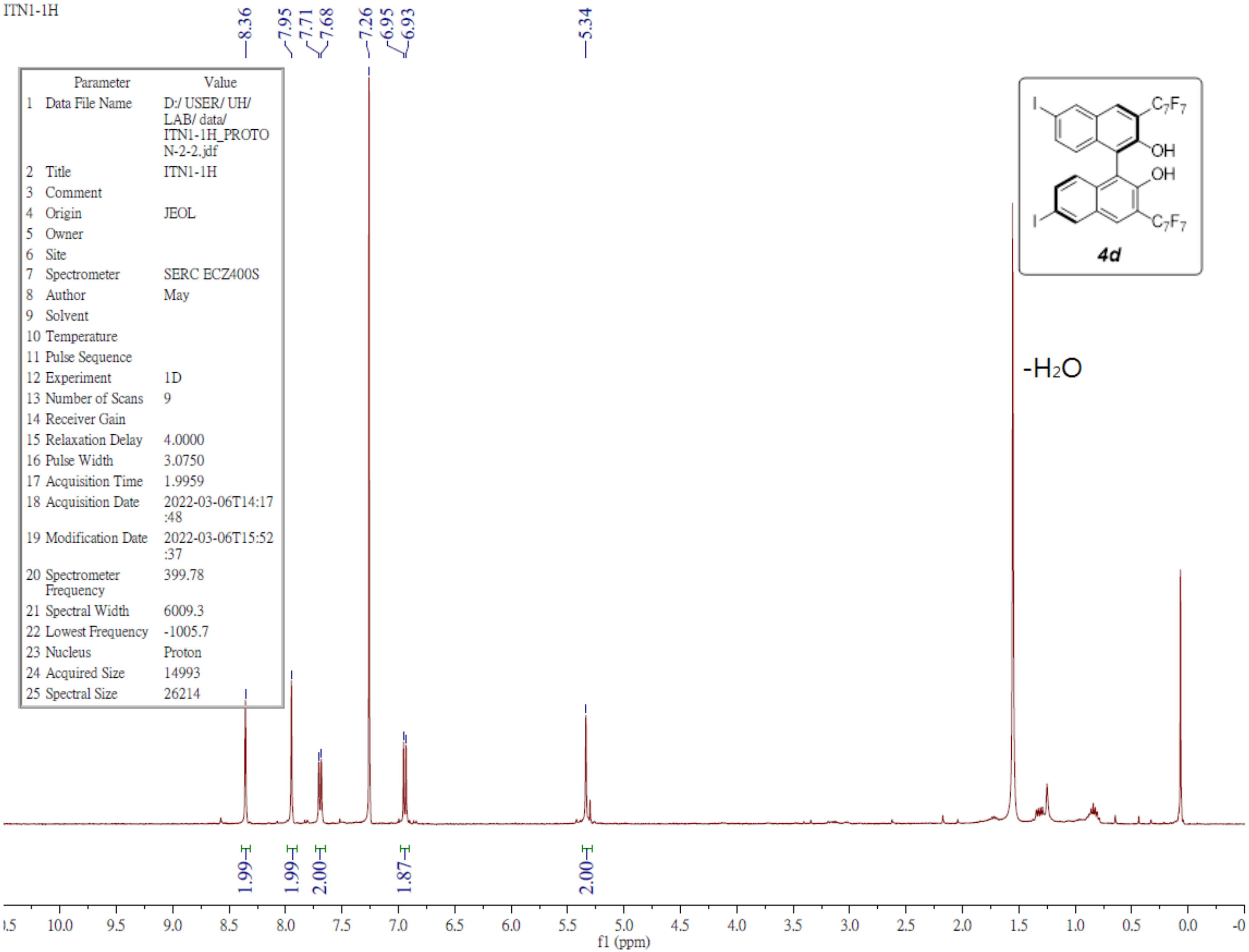
ITN1-1H
8.36
7.95
7.71
7.68
7.26
6.95
6.93
5.34
Parameter Value
1 Data File Name D:/ USER/ UH/ LAB/ data/ ITN1-1H_PROTON-2-2.jdf
2 Title ITN1-1H
3 Comment
4 Origin JEOL
5 Owner
6 Site
7 Spectrometer SERC ECZ400S
8 Author May
9 Solvent
10 Temperature
11 Pulse Sequence
12 Experiment 1D
13 Number of Scans 9
14 Receiver Gain
15 Relaxation Delay 4.0000
16 Pulse Width 3.0750
17 Acquisition Time 1.9959
18 Acquisition Date 2022-03-06T14:17:48
19 Modification Date 2022-03-06T15:52:37
20 Spectrometer Frequency 399.78
21 Spectral Width 6009.3
22 Lowest Frequency -1005.7
23 Nucleus Proton
24 Acquired Size 14993
25 Spectral Size 26214
I
C7F7
OH
OH
I
C7F7
4d
-H2O
1.99
1.99
2.00
1.87
2.00
10.0 9.5 9.0 8.5 8.0 7.5 7.0 6.5 6.0 5.5 5.0 4.5 4.0 3.5 3.0 2.5 2.0 1.5 1.0 0.5 0.0
f1 (ppm)

ITN1

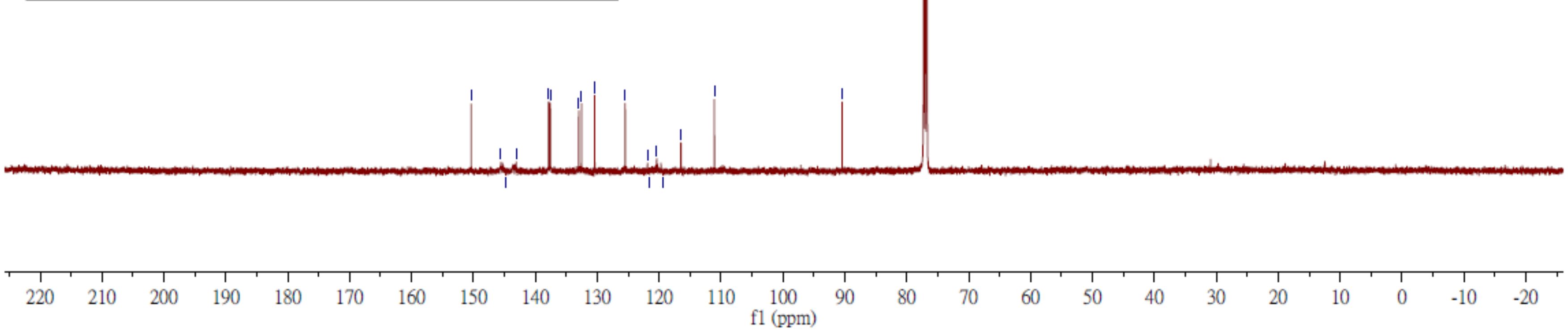

	Parameter	Value
1	Data File Name	D:/ USER/ UH/ LAB/ data/ ITN1_CARBON-4-2.jdf
2	Title	ITN1
3	Comment	
4	Origin	JEOL
5	Owner	
6	Site	
7	Spectrometer	SERC ECZ500R
8	Author	May
9	Solvent	
10	Temperature	
11	Pulse Sequence	
12	Experiment	1D
13	Number of Scans	10000
14	Receiver Gain	
15	Relaxation Delay	2.0000
16	Pulse Width	3.6177
17	Acquisition Time	0.9931
18	Acquisition Date	2021-11-21T17:02:48
19	Modification Date	2022-04-09T16:16:16
20	Spectrometer Frequency	125.71
21	Spectral Width	31645.4
22	Lowest Frequency	-3265.5
23	Nucleus	Carbon13
24	Acquired Size	39284
25	Spectral Size	104858

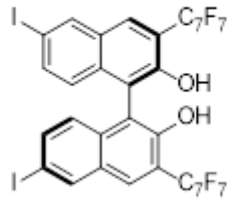

4d

ITN1-1H

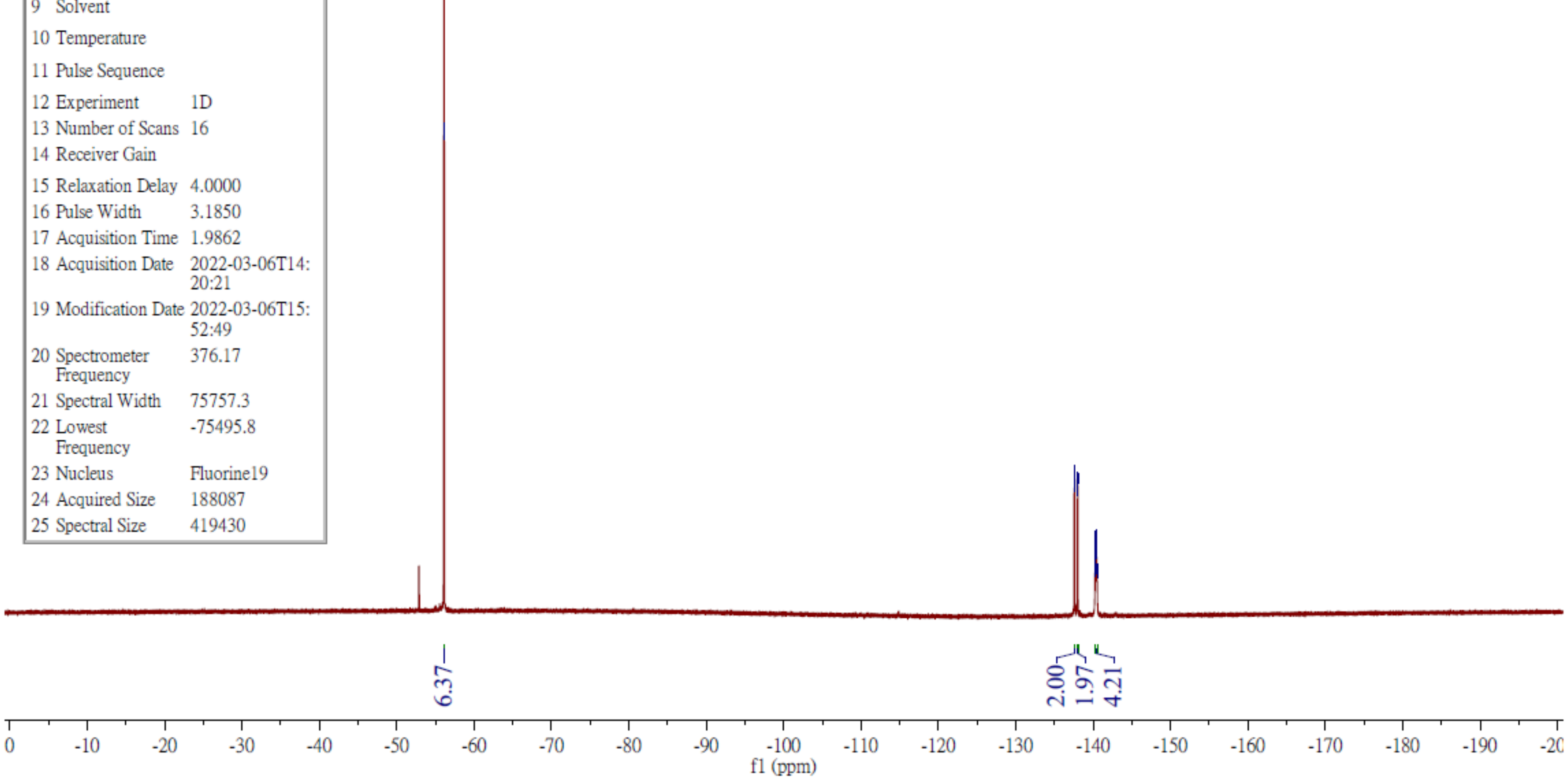

	Parameter	Value
1	Data File Name	D:/ USER/ UH/ LAB/ data/ ITN1-1H_Fluorine-2-2.jdf
2	Title	ITN1-1H
3	Comment	
4	Origin	JEOL
5	Owner	
6	Site	
7	Spectrometer	SERC ECZ400S
8	Author	May
9	Solvent	
10	Temperature	
11	Pulse Sequence	
12	Experiment	1D
13	Number of Scans	16
14	Receiver Gain	
15	Relaxation Delay	4.0000
16	Pulse Width	3.1850
17	Acquisition Time	1.9862
18	Acquisition Date	2022-03-06T14:20:21
19	Modification Date	2022-03-06T15:52:49
20	Spectrometer Frequency	376.17
21	Spectral Width	75757.3
22	Lowest Frequency	-75495.8
23	Nucleus	Fluorine19
24	Acquired Size	188087
25	Spectral Size	419430

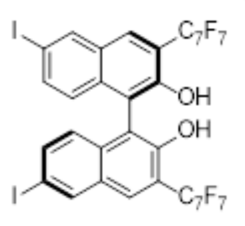

4d

L-002-034

	Parameter	Value
1	Data File Name	D:/ USER/ UH/ LAB/ data/ L-002-034_PROTON-2-1.jdf
2	Title	L-002-034
3	Comment	
4	Origin	JEOL
5	Owner	
6	Site	
7	Spectrometer	SERC ECZ400S
8	Author	May
9	Solvent	
10	Temperature	
11	Pulse Sequence	
12	Experiment	1D
13	Number of Scans	16
14	Receiver Gain	
15	Relaxation Delay	4.0000
16	Pulse Width	3.0750
17	Acquisition Time	1.9959
18	Acquisition Date	2021-08-27T16:58:51
19	Modification Date	2021-08-27T17:00:30
20	Spectrometer Frequency	399.78
21	Spectral Width	7512.0
22	Lowest Frequency	-1757.1
23	Nucleus	Proton
24	Acquired Size	14993
25	Spectral Size	32768

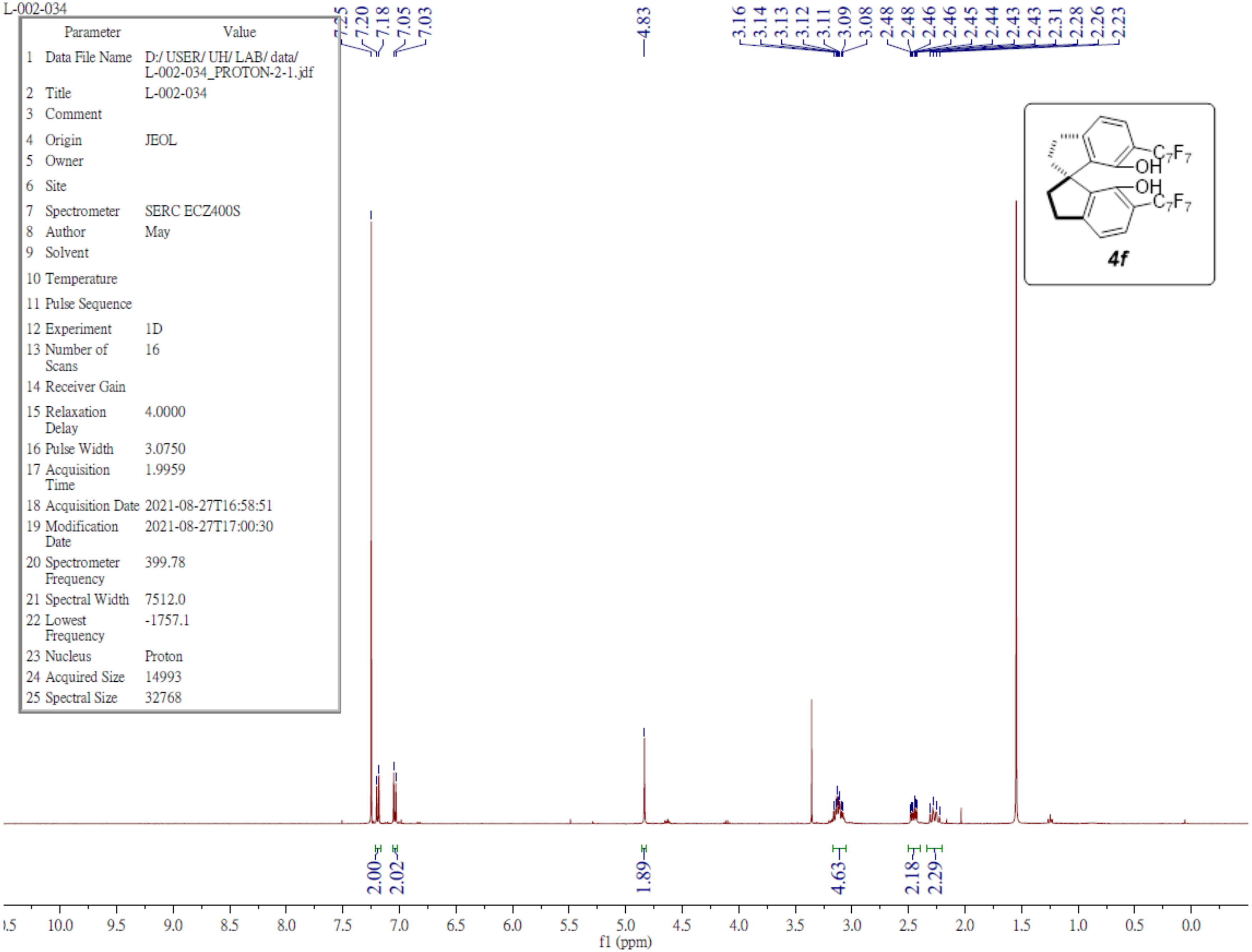

4f

spinol

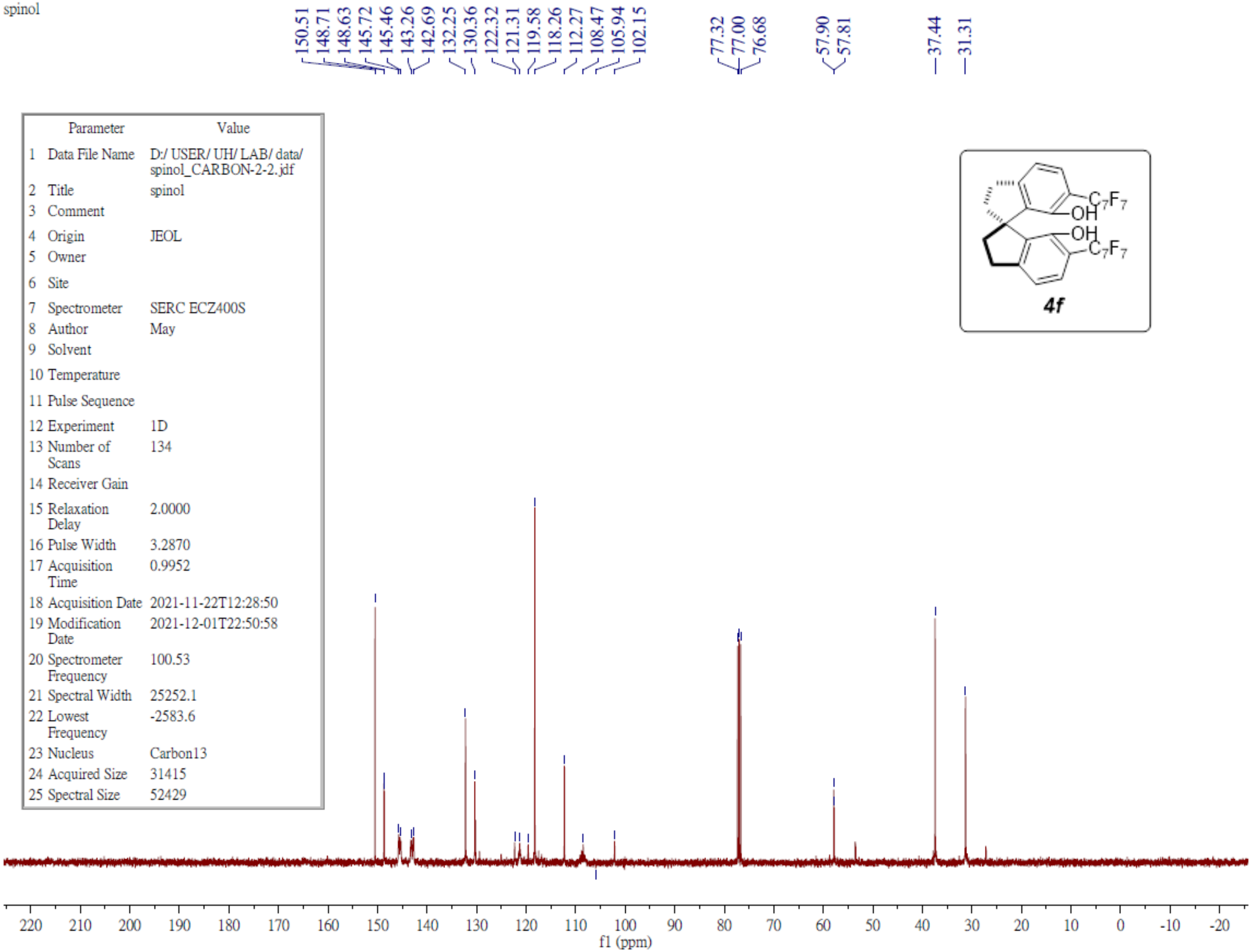

	Parameter	Value
1	Data File Name	D:/ USER/ UH/ LAB/ data/ spinol_CARBON-2-2.jdf
2	Title	spinol
3	Comment	
4	Origin	JEOL
5	Owner	
6	Site	
7	Spectrometer	SERC ECZ400S
8	Author	May
9	Solvent	
10	Temperature	
11	Pulse Sequence	
12	Experiment	1D
13	Number of Scans	134
14	Receiver Gain	
15	Relaxation Delay	2.0000
16	Pulse Width	3.2870
17	Acquisition Time	0.9952
18	Acquisition Date	2021-11-22T12:28:50
19	Modification Date	2021-12-01T22:50:58
20	Spectrometer Frequency	100.53
21	Spectral Width	25252.1
22	Lowest Frequency	-2583.6
23	Nucleus	Carbon13
24	Acquired Size	31415
25	Spectral Size	52429

4f

L-002-034

	Parameter	Value
1	Data File Name	D:/ USER/ UH/ LAB/ data/ L-002-034_Fluorine-2-2.jdf
2	Title	L-002-034
3	Comment	
4	Origin	JEOL
5	Owner	
6	Site	
7	Spectrometer	SERC ECZ400S
8	Author	May
9	Solvent	
10	Temperature	
11	Pulse Sequence	
12	Experiment	1D
13	Number of Scans	16
14	Receiver Gain	
15	Relaxation Delay	4.0000
16	Pulse Width	3.1850
17	Acquisition Time	1.9862
18	Acquisition Date	2021-08-27T16:54:17
19	Modification Date	2021-08-27T18:00:23
20	Spectrometer Frequency	376.17
21	Spectral Width	75757.3
22	Lowest Frequency	-75495.8
23	Nucleus	Fluorine19
24	Acquired Size	188087
25	Spectral Size	419430

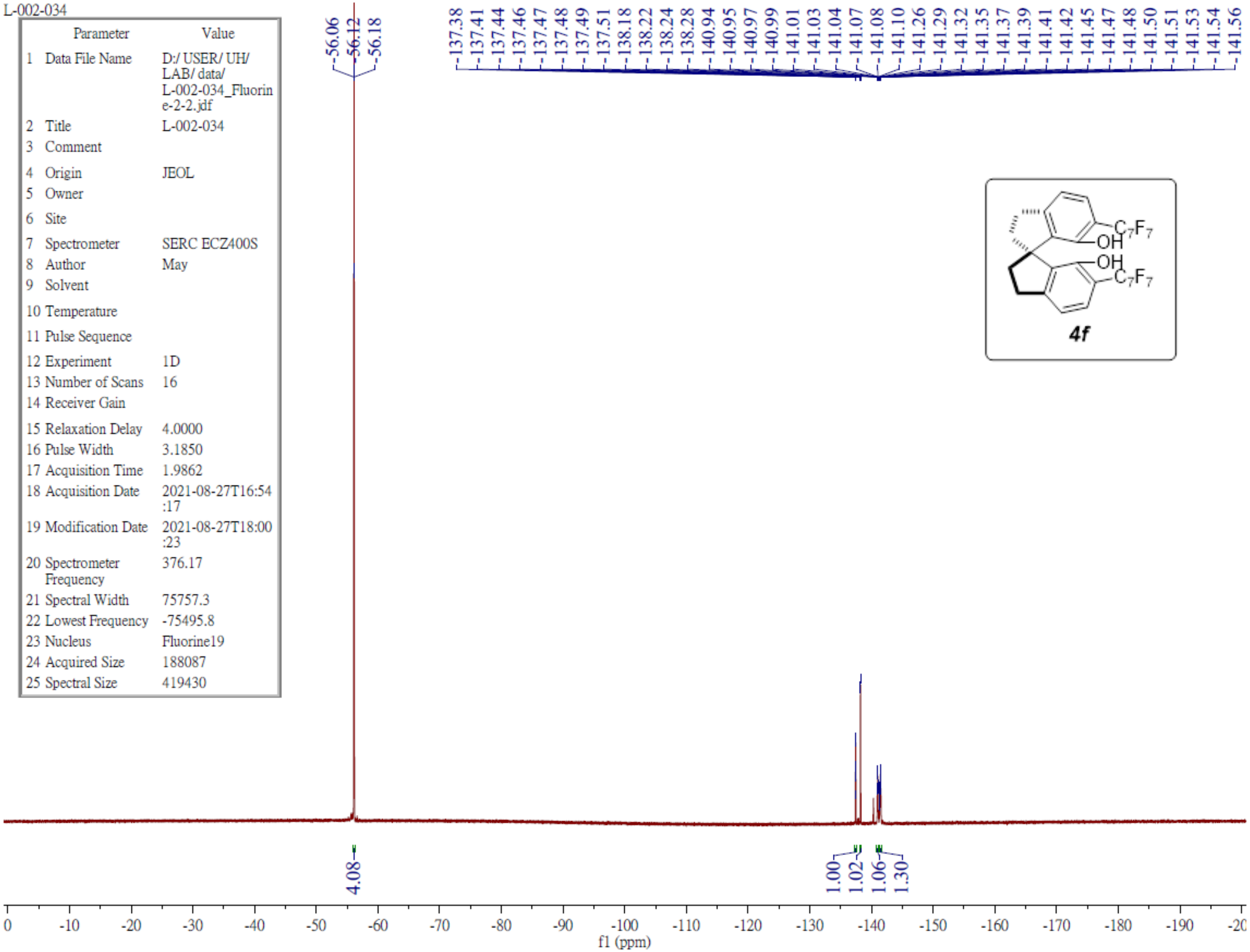

4f

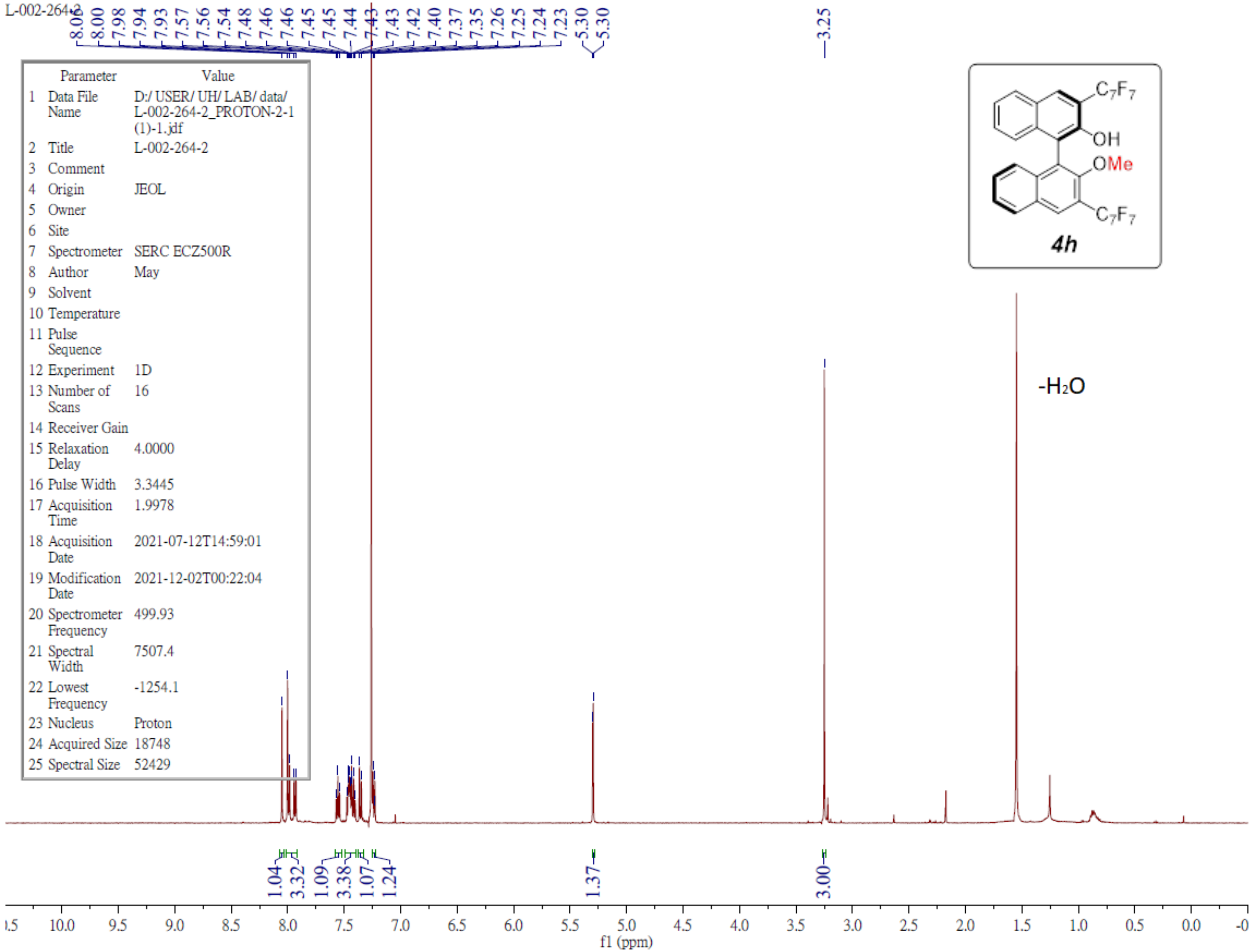

	Parameter	Value
1	Data File Name	D:/ USER/ UH/ LAB/ data/ L-002-264-2_PROTON-2-1 (1)-1.jdf
2	Title	L-002-264-2
3	Comment	
4	Origin	JEOL
5	Owner	
6	Site	
7	Spectrometer	SERC ECZ500R
8	Author	May
9	Solvent	
10	Temperature	
11	Pulse Sequence	
12	Experiment	1D
13	Number of Scans	16
14	Receiver Gain	
15	Relaxation Delay	4.0000
16	Pulse Width	3.3445
17	Acquisition Time	1.9978
18	Acquisition Date	2021-07-12T14:59:01
19	Modification Date	2021-12-02T00:22:04
20	Spectrometer Frequency	499.93
21	Spectral Width	7507.4
22	Lowest Frequency	-1254.1
23	Nucleus	Proton
24	Acquired Size	18748
25	Spectral Size	52429

L-002-264-2

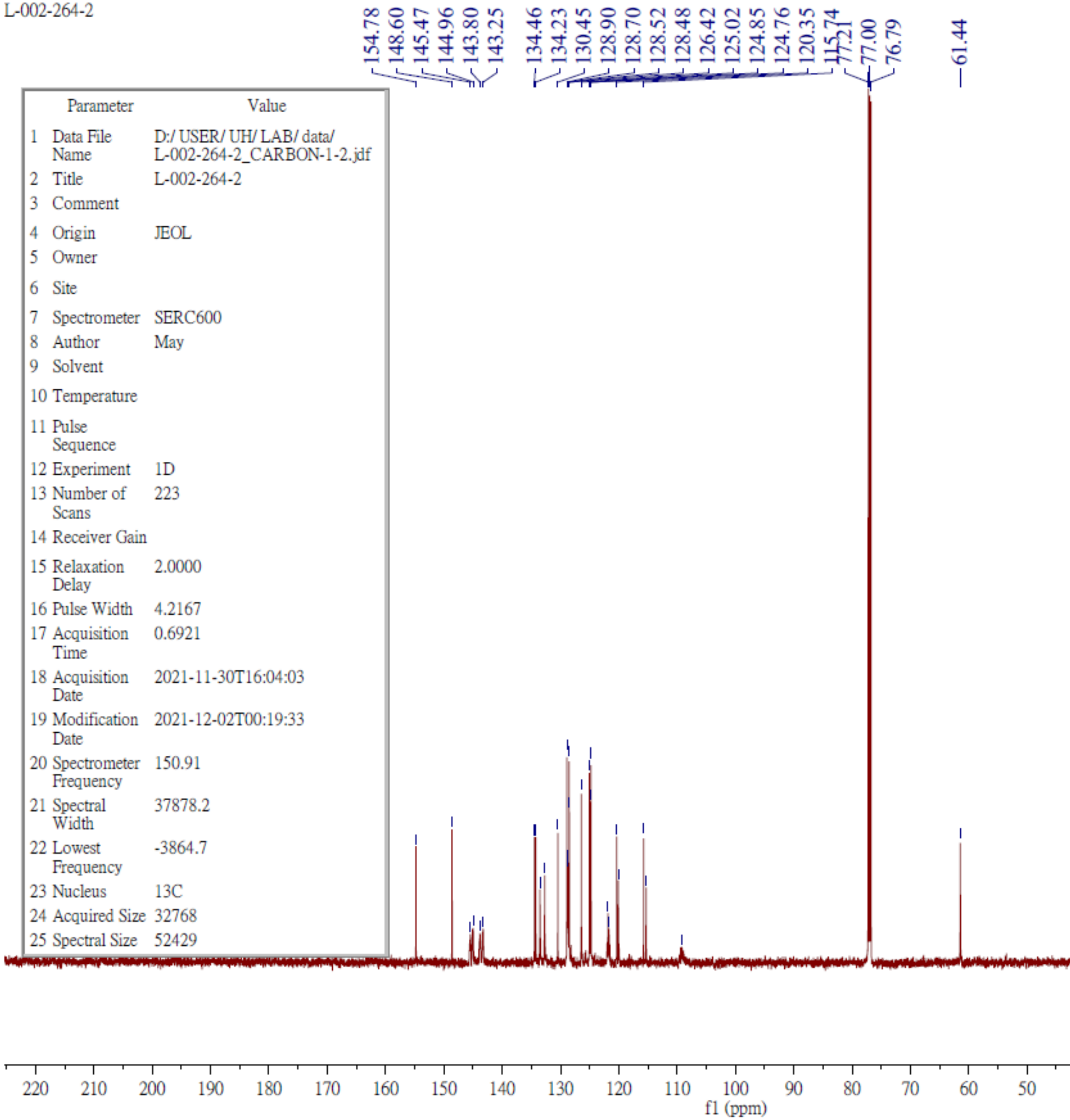

	Parameter	Value
1	Data File Name	D:/ USER/ UH/ LAB/ data/ L-002-264-2_CARBON-1-2.jdf
2	Title	L-002-264-2
3	Comment	
4	Origin	JEOL
5	Owner	
6	Site	
7	Spectrometer	SERC600
8	Author	May
9	Solvent	
10	Temperature	
11	Pulse Sequence	
12	Experiment	1D
13	Number of Scans	223
14	Receiver Gain	
15	Relaxation Delay	2.0000
16	Pulse Width	4.2167
17	Acquisition Time	0.6921
18	Acquisition Date	2021-11-30T16:04:03
19	Modification Date	2021-12-02T00:19:33
20	Spectrometer Frequency	150.91
21	Spectral Width	37878.2
22	Lowest Frequency	-3864.7
23	Nucleus	13C
24	Acquired Size	32768
25	Spectral Size	52429

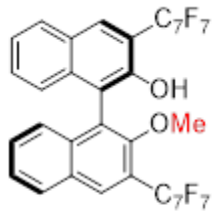

4h

L-003-264-2

Parameter		Value
1	Data File Name	D:/ USER/ UH/ LAB/ data/ L-003-264-2_Fluorine-2-1 (1)-1.jdf
2	Title	L-003-264-2
3	Comment	
4	Origin	JEOL
5	Owner	
6	Site	
7	Spectrometer	SERC ECZ400S
8	Author	May
9	Solvent	
10	Temperature	
11	Pulse Sequence	
12	Experiment	1D
13	Number of Scans	16
14	Receiver Gain	
15	Relaxation Delay	4.0000
16	Pulse Width	3.1850
17	Acquisition Time	1.9862
18	Acquisition Date	2021-11-30T16:16:09
19	Modification Date	2021-12-02T00:20:16
20	Spectrometer Frequency	376.17
21	Spectral Width	75757.3
22	Lowest Frequency	-75495.8
23	Nucleus	Fluorine19
24	Acquired Size	188087
25	Spectral Size	419430

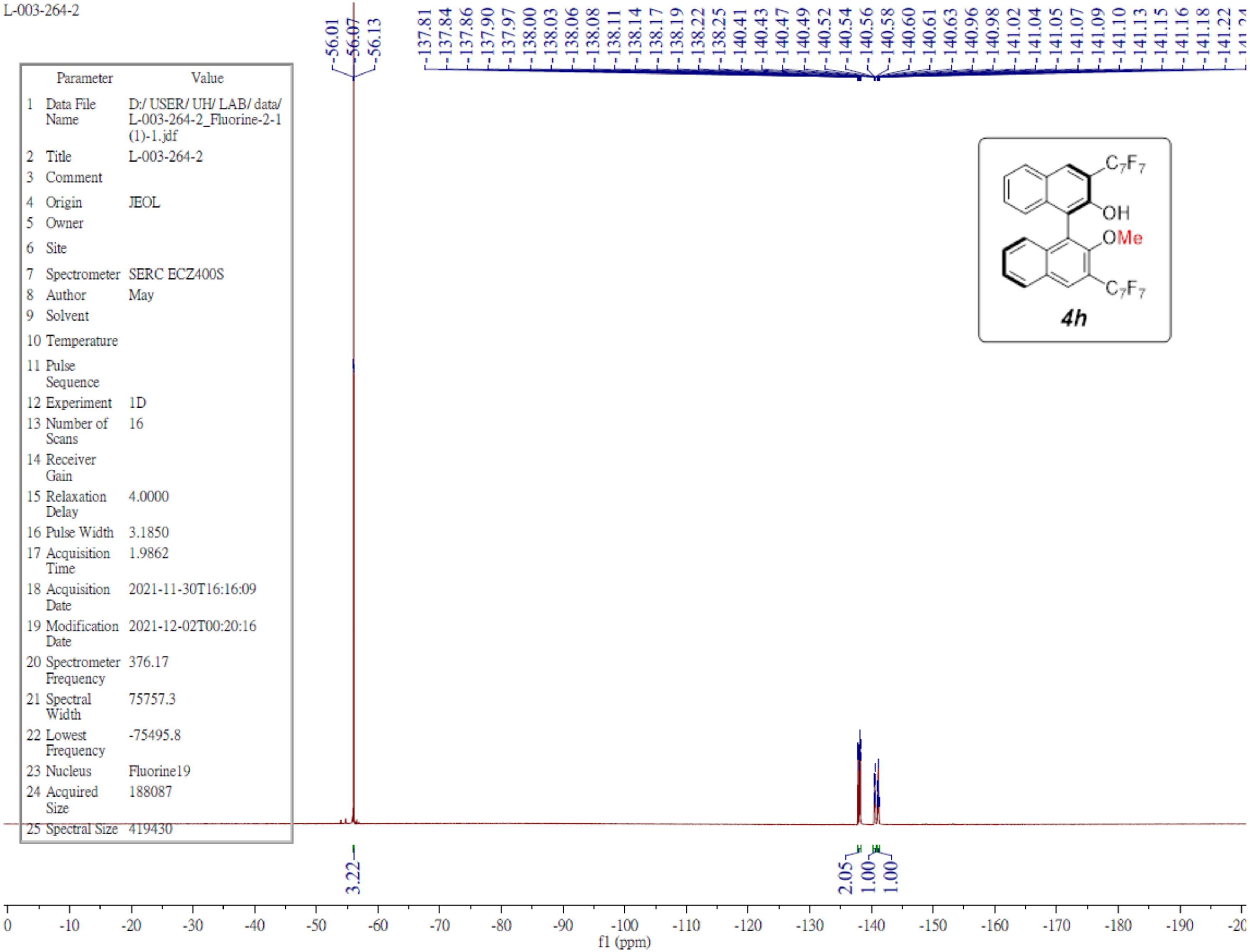

4h

L-002-149

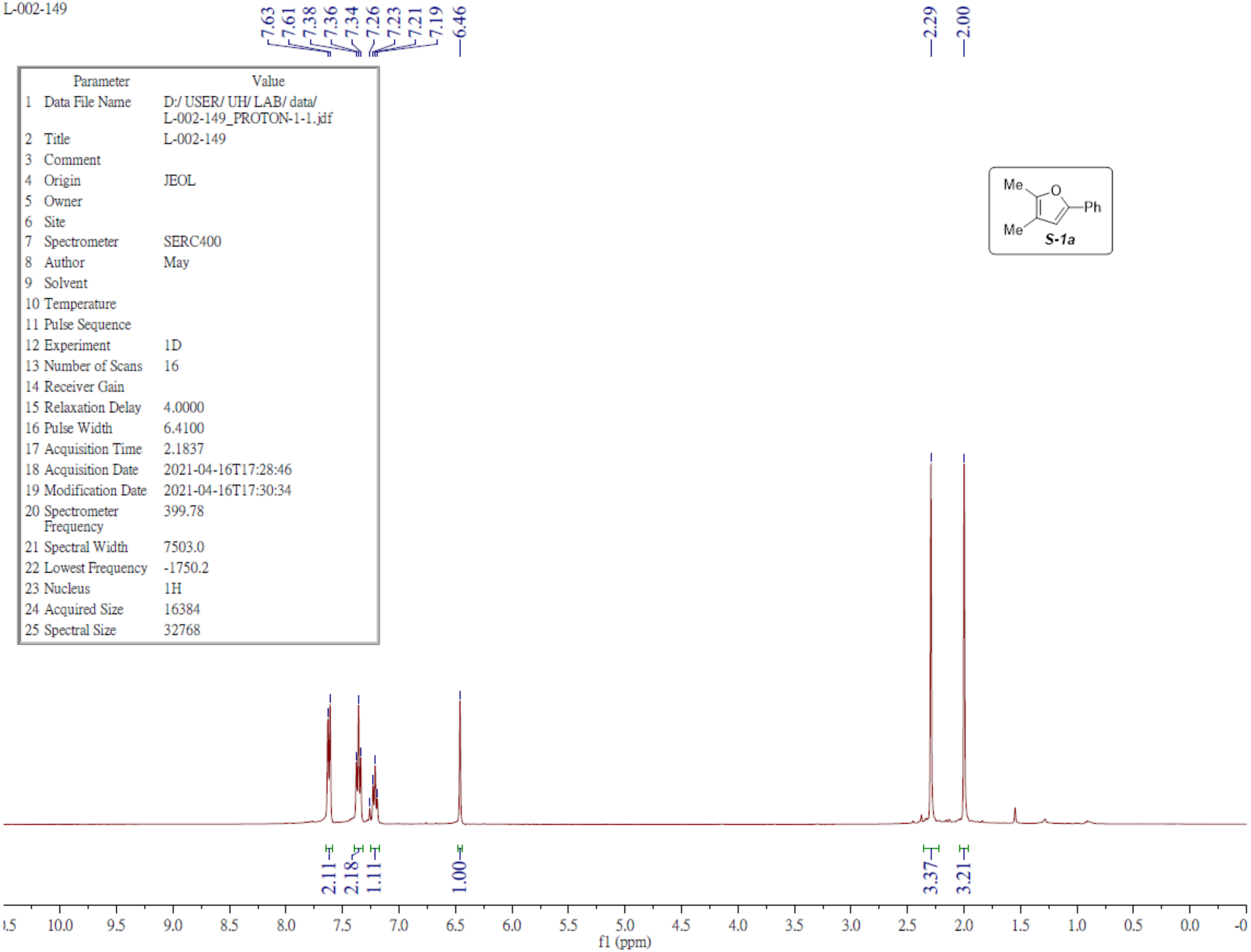

	Parameter	Value
1	Data File Name	D:/ USER/ UH/ LAB/ data/ L-002-149_PROTON-1-1.jdf
2	Title	L-002-149
3	Comment	
4	Origin	JEOL
5	Owner	
6	Site	
7	Spectrometer	SERC400
8	Author	May
9	Solvent	
10	Temperature	
11	Pulse Sequence	
12	Experiment	1D
13	Number of Scans	16
14	Receiver Gain	
15	Relaxation Delay	4.0000
16	Pulse Width	6.4100
17	Acquisition Time	2.1837
18	Acquisition Date	2021-04-16T17:28:46
19	Modification Date	2021-04-16T17:30:34
20	Spectrometer Frequency	399.78
21	Spectral Width	7503.0
22	Lowest Frequency	-1750.2
23	Nucleus	1H
24	Acquired Size	16384
25	Spectral Size	32768

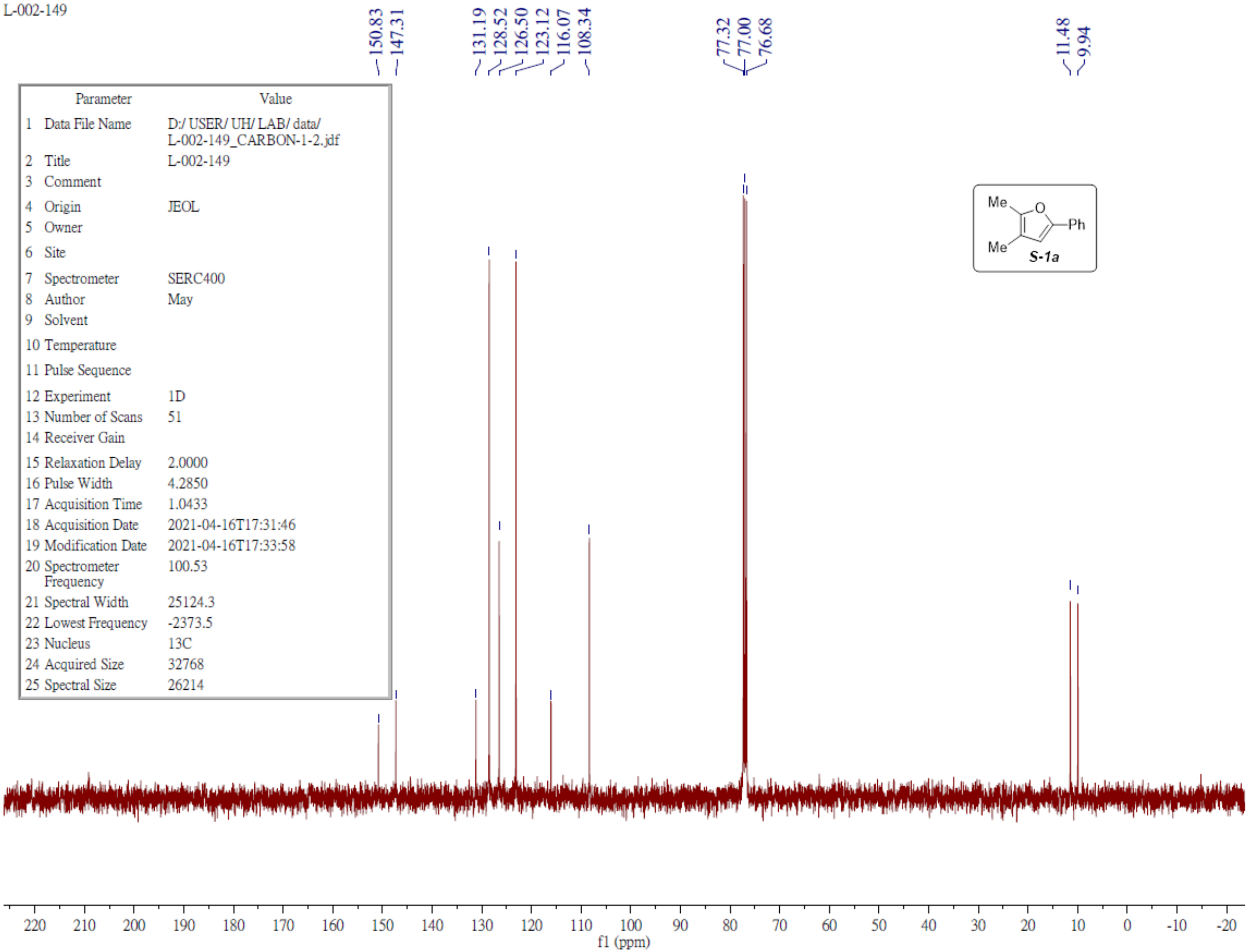

L-002-149
150.83
147.31
131.19
128.52
126.50
123.12
116.07
108.34
77.32
77.00
76.68
11.48
9.94
Parameter | Value
1 Data File Name | D:/ USER/ UH/ LAB/ data/ L-002-149_CARBON-1-2.jdf
2 Title | L-002-149
3 Comment
4 Origin | JEOL
5 Owner
6 Site
7 Spectrometer | SERC400
8 Author | May
9 Solvent
10 Temperature
11 Pulse Sequence
12 Experiment | 1D
13 Number of Scans | 51
14 Receiver Gain
15 Relaxation Delay | 2.0000
16 Pulse Width | 4.2850
17 Acquisition Time | 1.0433
18 Acquisition Date | 2021-04-16T17:31:46
19 Modification Date | 2021-04-16T17:33:58
20 Spectrometer Frequency | 100.53
21 Spectral Width | 25124.3
22 Lowest Frequency | -2373.5
23 Nucleus | 13C
24 Acquired Size | 32768
25 Spectral Size | 26214
Me
O
Ph
Me
S-1a
220 210 200 190 180 170 160 150 140 130 120 110 100 90 80 70 60 50 40 30 20 10 0 -10 -20
f1 (ppm)

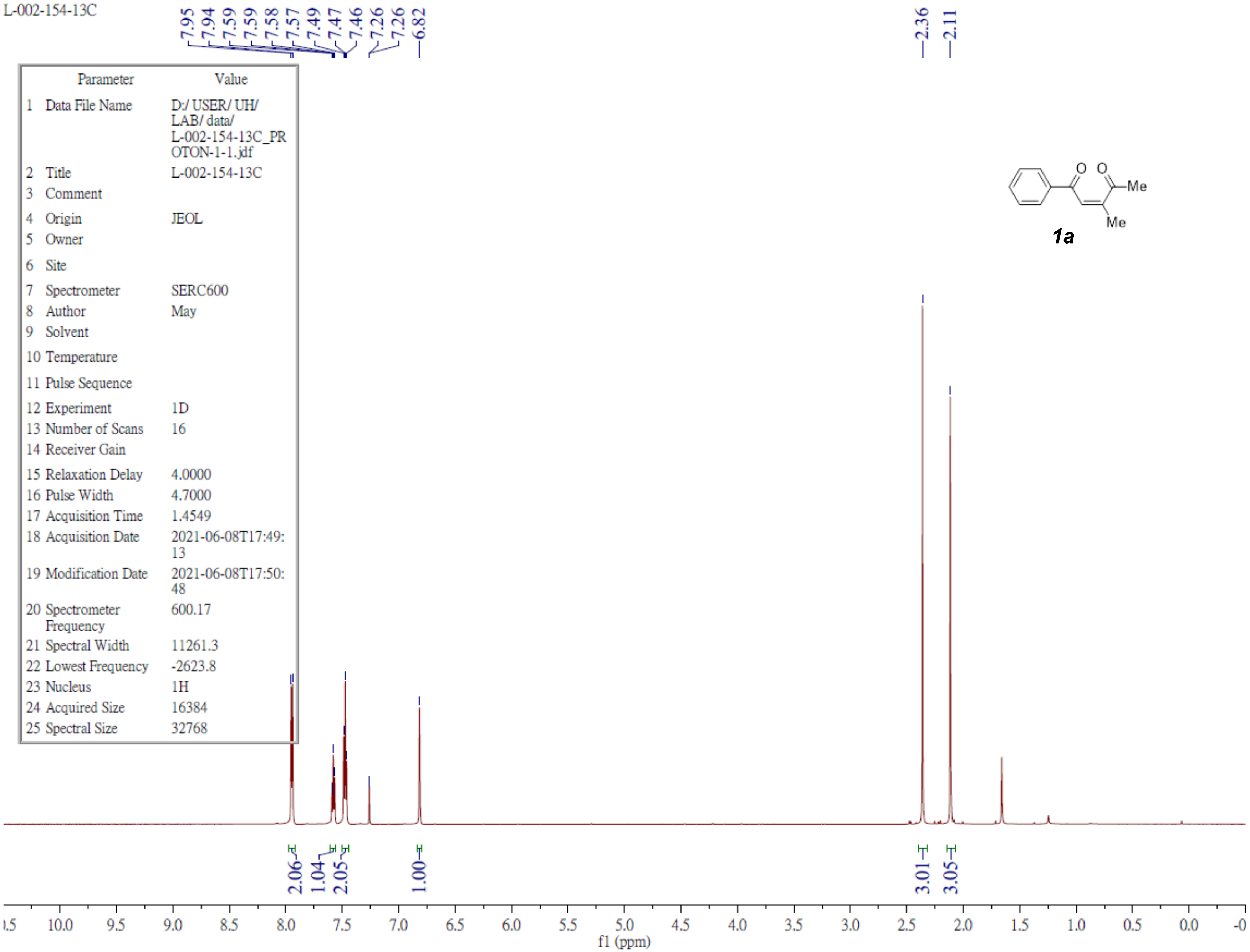
L-002-154-13C
7.95
7.94
7.59
7.59
7.58
7.57
7.49
7.47
7.46
7.26
7.26
6.82
2.36
2.11
Parameter
Value
1 Data File Name
D:/ USER/ UH/ LAB/ data/ L-002-154-13C_PROTON-1-1.jdf
2 Title
L-002-154-13C
3 Comment
4 Origin
JEOL
5 Owner
6 Site
7 Spectrometer
SERC600
8 Author
May
9 Solvent
10 Temperature
11 Pulse Sequence
12 Experiment
1D
13 Number of Scans
16
14 Receiver Gain
15 Relaxation Delay
4.0000
16 Pulse Width
4.7000
17 Acquisition Time
1.4549
18 Acquisition Date
2021-06-08T17:49:13
19 Modification Date
2021-06-08T17:50:48
20 Spectrometer Frequency
600.17
21 Spectral Width
11261.3
22 Lowest Frequency
-2623.8
23 Nucleus
1H
24 Acquired Size
16384
25 Spectral Size
32768
O
O
Me
Me
1a
2.06
1.04
2.05
1.00
3.01
3.05
f1 (ppm)

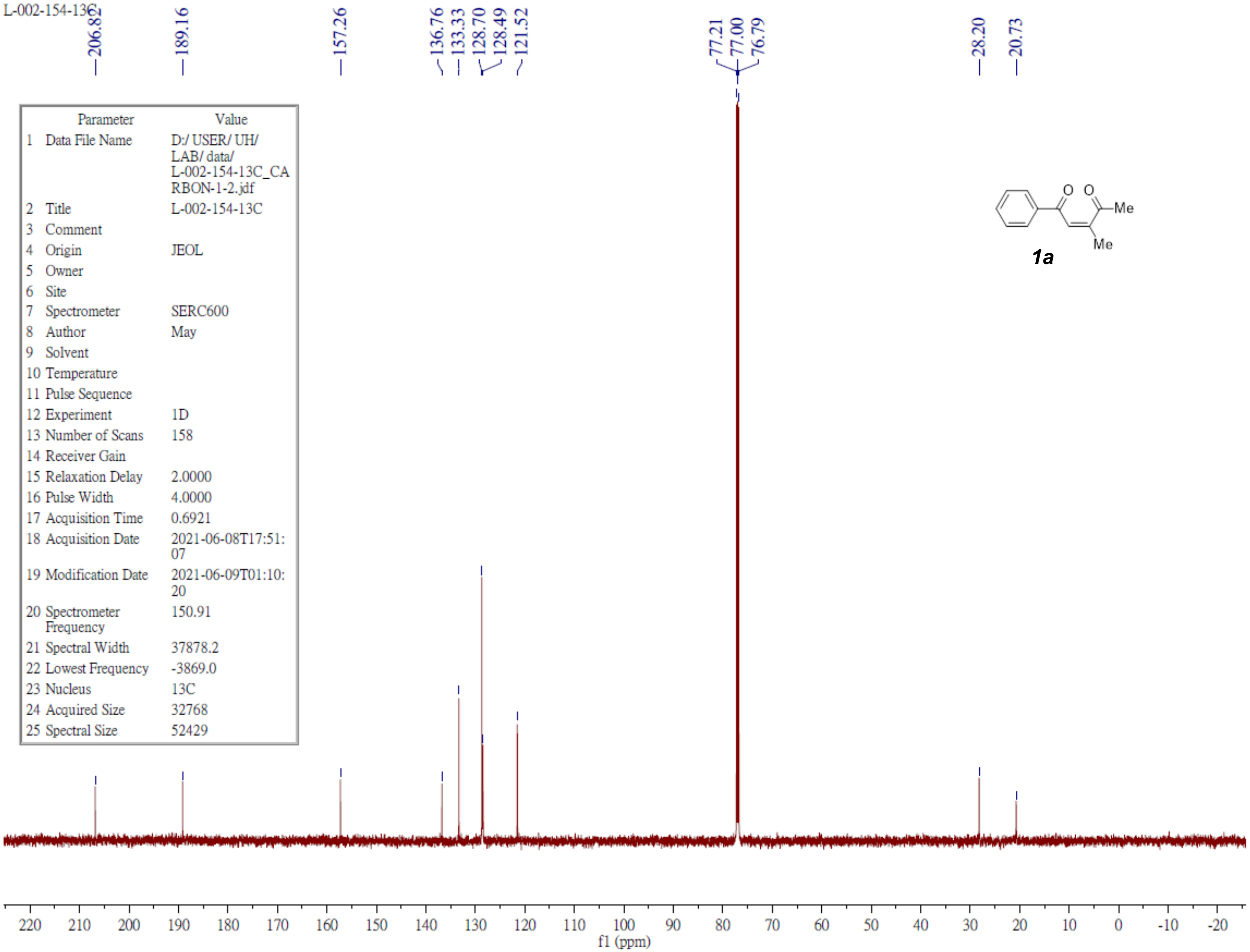

	Parameter	Value
1	Data File Name	D:/ USER/ UH/ LAB/ data/ L-002-154-13C_CARBON-1-2.jdf
2	Title	L-002-154-13C
3	Comment	
4	Origin	JEOL
5	Owner	
6	Site	
7	Spectrometer	SERC600
8	Author	May
9	Solvent	
10	Temperature	
11	Pulse Sequence	
12	Experiment	1D
13	Number of Scans	158
14	Receiver Gain	
15	Relaxation Delay	2.0000
16	Pulse Width	4.0000
17	Acquisition Time	0.6921
18	Acquisition Date	2021-06-08T17:51:07
19	Modification Date	2021-06-09T01:10:20
20	Spectrometer Frequency	150.91
21	Spectral Width	37878.2
22	Lowest Frequency	-3869.0
23	Nucleus	13C
24	Acquired Size	32768
25	Spectral Size	52429

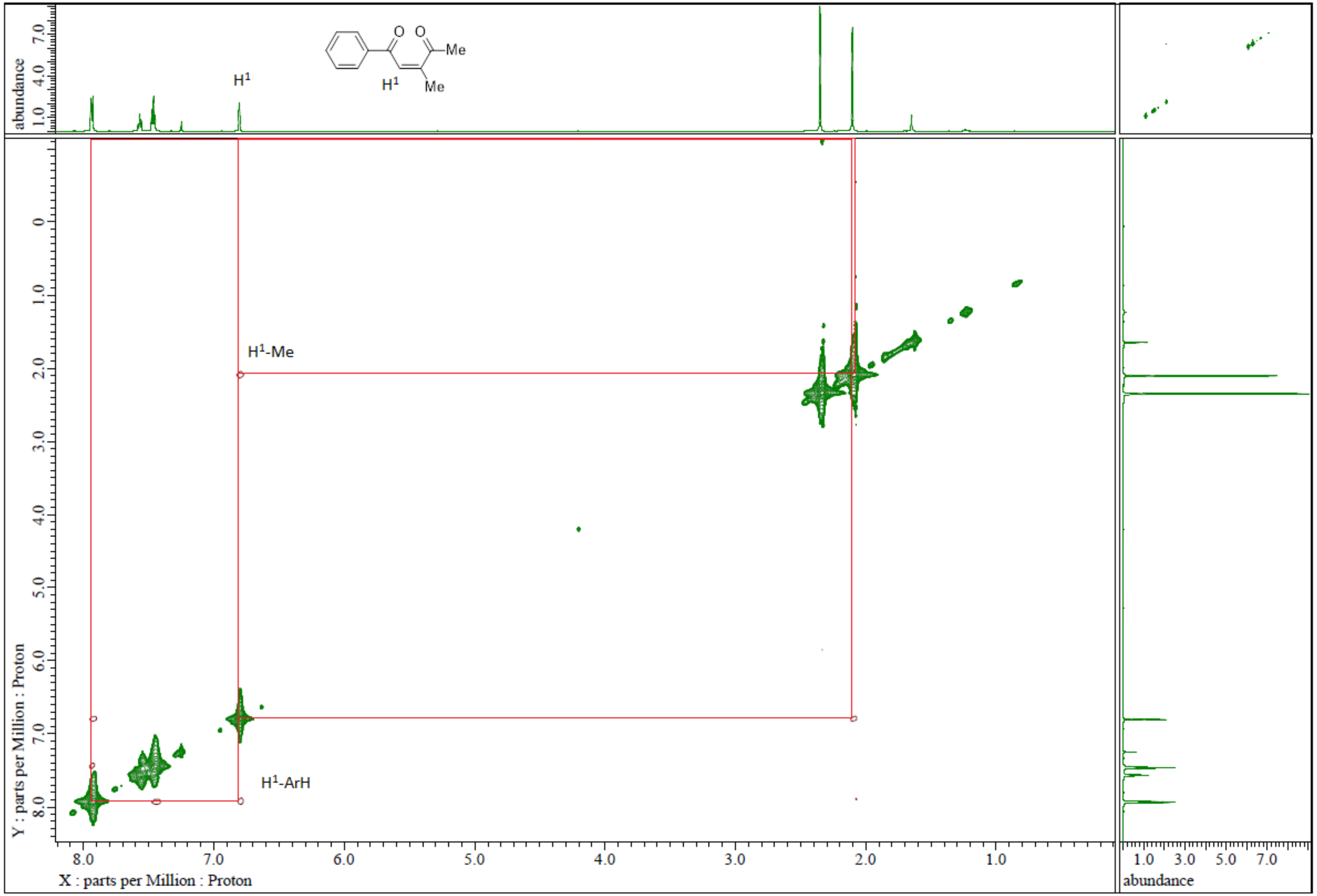

NOSEY spectrum of 1a

L-005-140

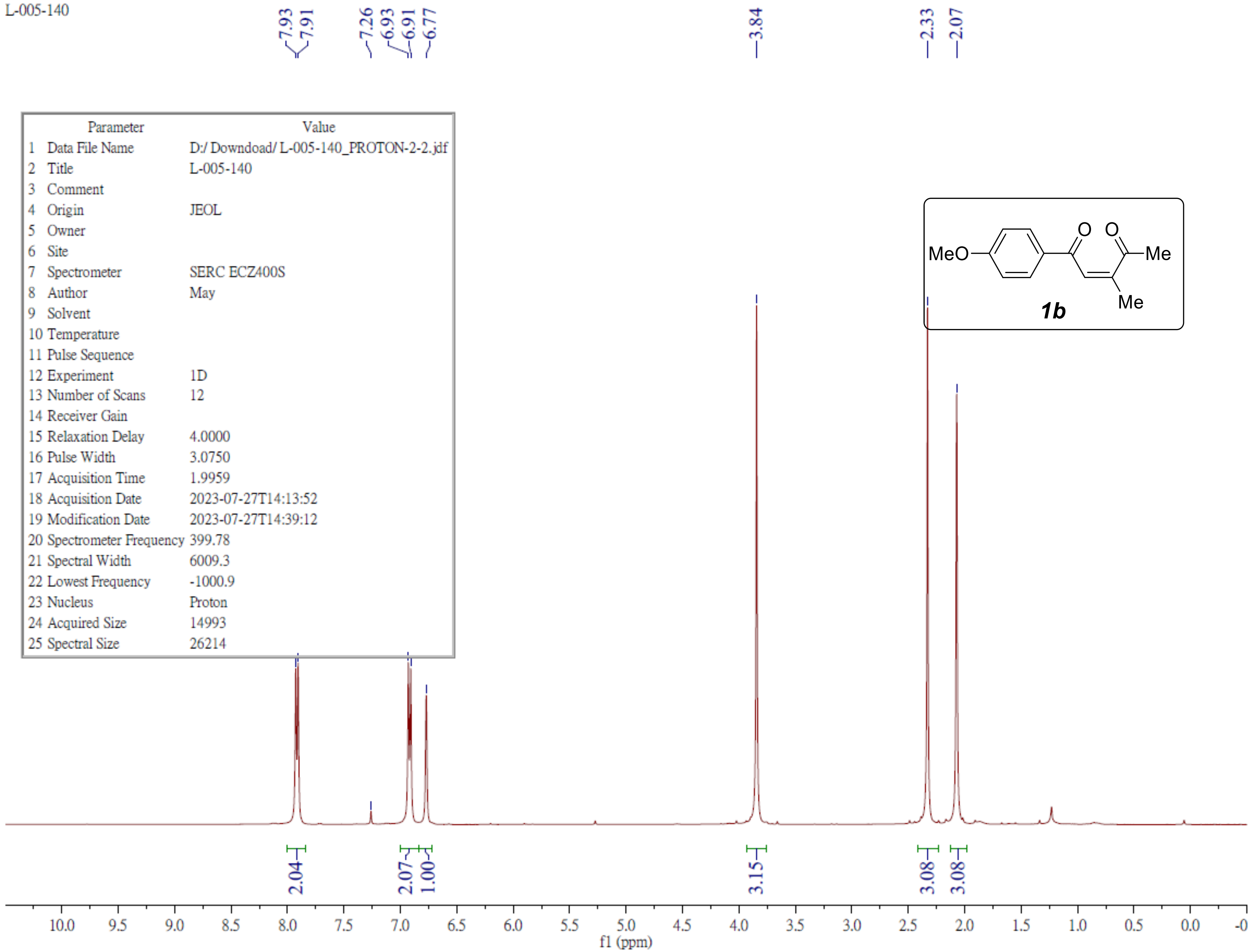

	Parameter	Value
1	Data File Name	D:/ Downdoad/ L-005-140_PROTON-2-2.jdf
2	Title	L-005-140
3	Comment	
4	Origin	JEOL
5	Owner	
6	Site	
7	Spectrometer	SERC ECZ400S
8	Author	May
9	Solvent	
10	Temperature	
11	Pulse Sequence	
12	Experiment	1D
13	Number of Scans	12
14	Receiver Gain	
15	Relaxation Delay	4.0000
16	Pulse Width	3.0750
17	Acquisition Time	1.9959
18	Acquisition Date	2023-07-27T14:13:52
19	Modification Date	2023-07-27T14:39:12
20	Spectrometer Frequency	399.78
21	Spectral Width	6009.3
22	Lowest Frequency	-1000.9
23	Nucleus	Proton
24	Acquired Size	14993
25	Spectral Size	26214

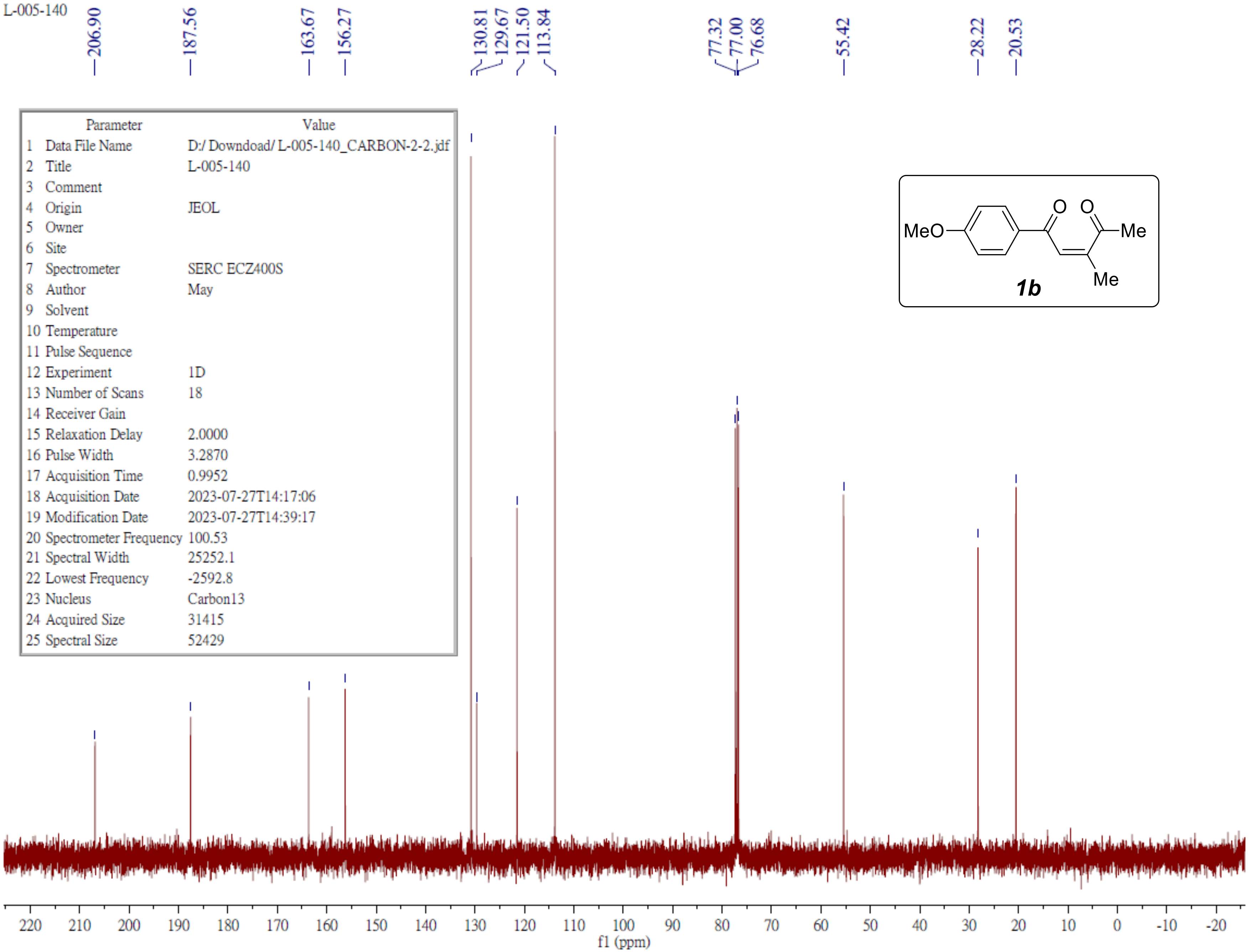
L-005-140
206.90
187.56
163.67
156.27
130.81
129.67
121.50
113.84
77.32
77.00
76.68
55.42
28.22
20.53
Parameter Value
1 Data File Name D:/ Downdoad/ L-005-140_CARBON-2-2.jdf
2 Title L-005-140
3 Comment
4 Origin JEOL
5 Owner
6 Site
7 Spectrometer SERC ECZ400S
8 Author May
9 Solvent
10 Temperature
11 Pulse Sequence
12 Experiment 1D
13 Number of Scans 18
14 Receiver Gain
15 Relaxation Delay 2.0000
16 Pulse Width 3.2870
17 Acquisition Time 0.9952
18 Acquisition Date 2023-07-27T14:17:06
19 Modification Date 2023-07-27T14:39:17
20 Spectrometer Frequency 100.53
21 Spectral Width 25252.1
22 Lowest Frequency -2592.8
23 Nucleus Carbon13
24 Acquired Size 31415
25 Spectral Size 52429
MeO
O
O
Me
Me
1b
220 210 200 190 180 170 160 150 140 130 120 110 100 90 80 70 60 50 40 30 20 10 0 -10 -20
f1 (ppm)

L-003-045-13C

Parameter		Value
1	Data File Name	D:/ USER/ UH/ LAB/ data/ L-003-045-13C_PROTON-2-2.jdf
2	Title	L-003-045-13C
3	Comment	
4	Origin	JEOL
5	Owner	
6	Site	
7	Spectrometer	SERC ECZ400S
8	Author	May
9	Solvent	
10	Temperature	
11	Pulse Sequence	
12	Experiment	1D
13	Number of Scans	16
14	Receiver Gain	
15	Relaxation Delay	4.0000
16	Pulse Width	3.0750
17	Acquisition Time	1.9959
18	Acquisition Date	2021-09-10T14:45:10
19	Modification Date	2021-09-11T18:33:24
20	Spectrometer Frequency	399.78
21	Spectral Width	6009.3
22	Lowest Frequency	-1000.9
23	Nucleus	Proton
24	Acquired Size	14993
25	Spectral Size	26214

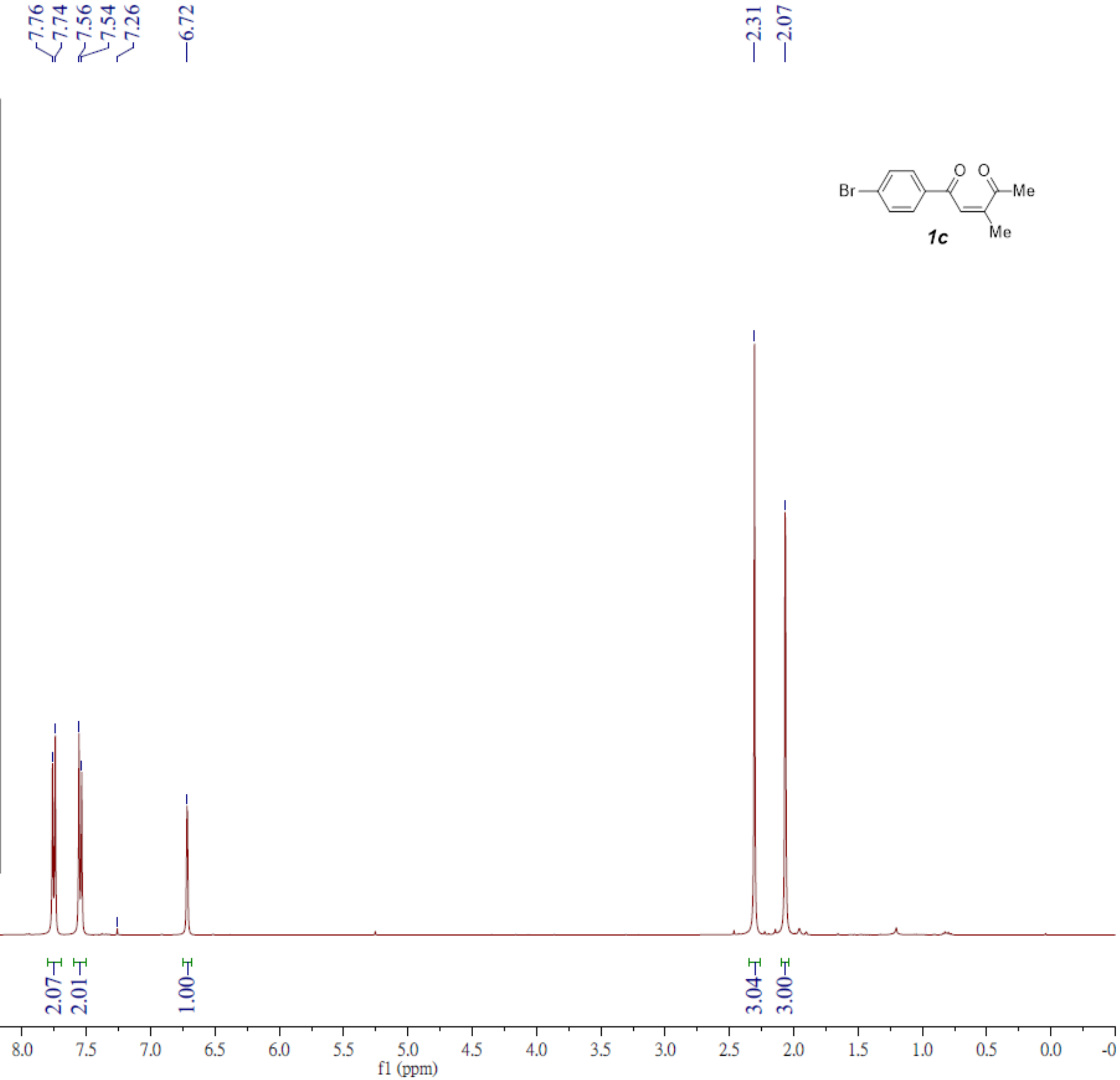

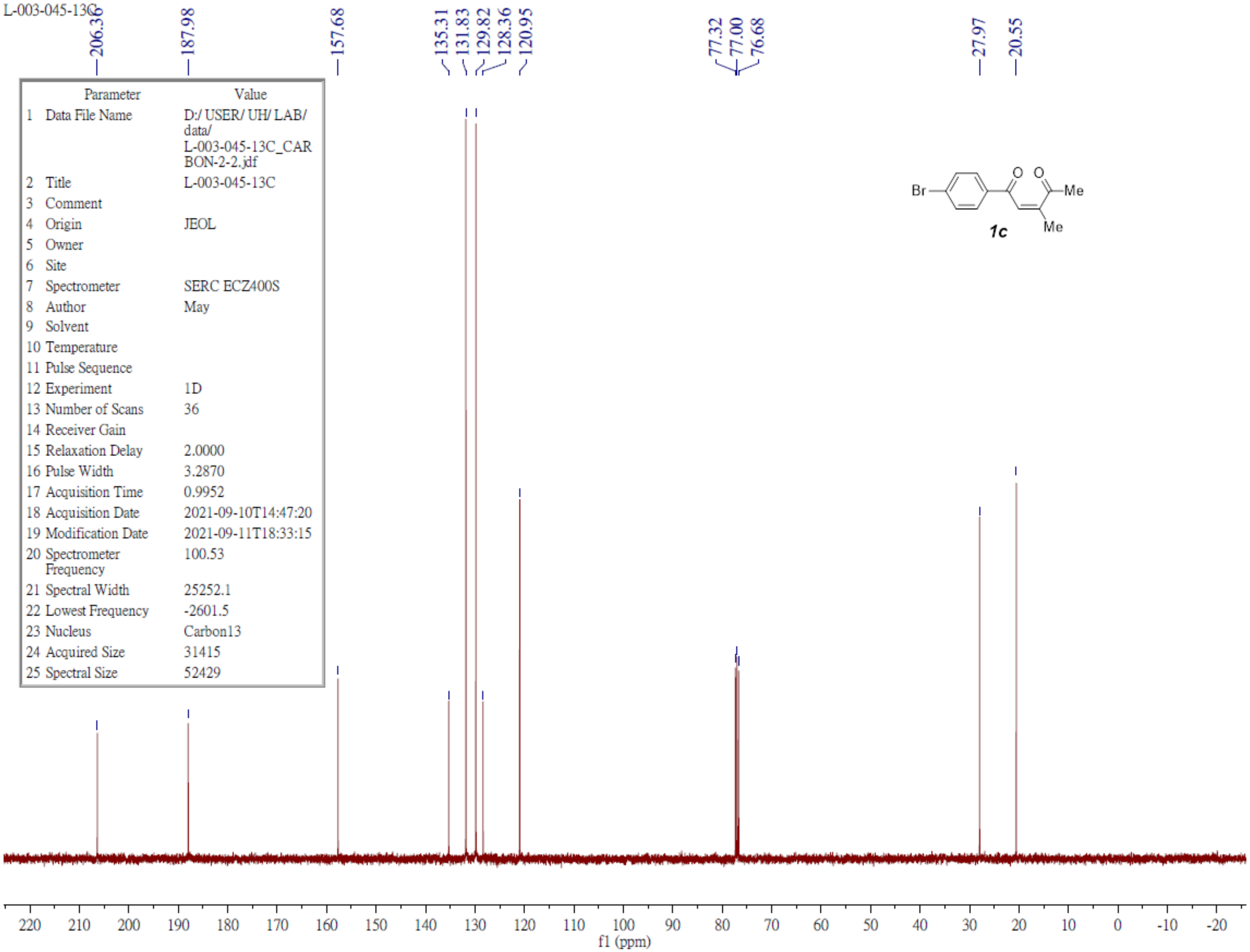
L-003-045-13C
206.36
187.98
157.68
135.31
131.83
129.82
128.36
120.95
77.32
77.00
76.68
27.97
20.55
Parameter | Value
1 Data File Name | D:/ USER/ UH/ LAB/ data/ L-003-045-13C_CARBON-2-2.jdf
2 Title | L-003-045-13C
3 Comment
4 Origin | JEOL
5 Owner
6 Site
7 Spectrometer | SERC ECZ400S
8 Author | May
9 Solvent
10 Temperature
11 Pulse Sequence
12 Experiment | 1D
13 Number of Scans | 36
14 Receiver Gain
15 Relaxation Delay | 2.0000
16 Pulse Width | 3.2870
17 Acquisition Time | 0.9952
18 Acquisition Date | 2021-09-10T14:47:20
19 Modification Date | 2021-09-11T18:33:15
20 Spectrometer Frequency | 100.53
21 Spectral Width | 25252.1
22 Lowest Frequency | -2601.5
23 Nucleus | Carbon13
24 Acquired Size | 31415
25 Spectral Size | 52429
Br
O
O
Me
Me
1c
220 210 200 190 180 170 160 150 140 130 120 110 100 90 80 70 60 50 40 30 20 10 0 -10 -20
f1 (ppm)

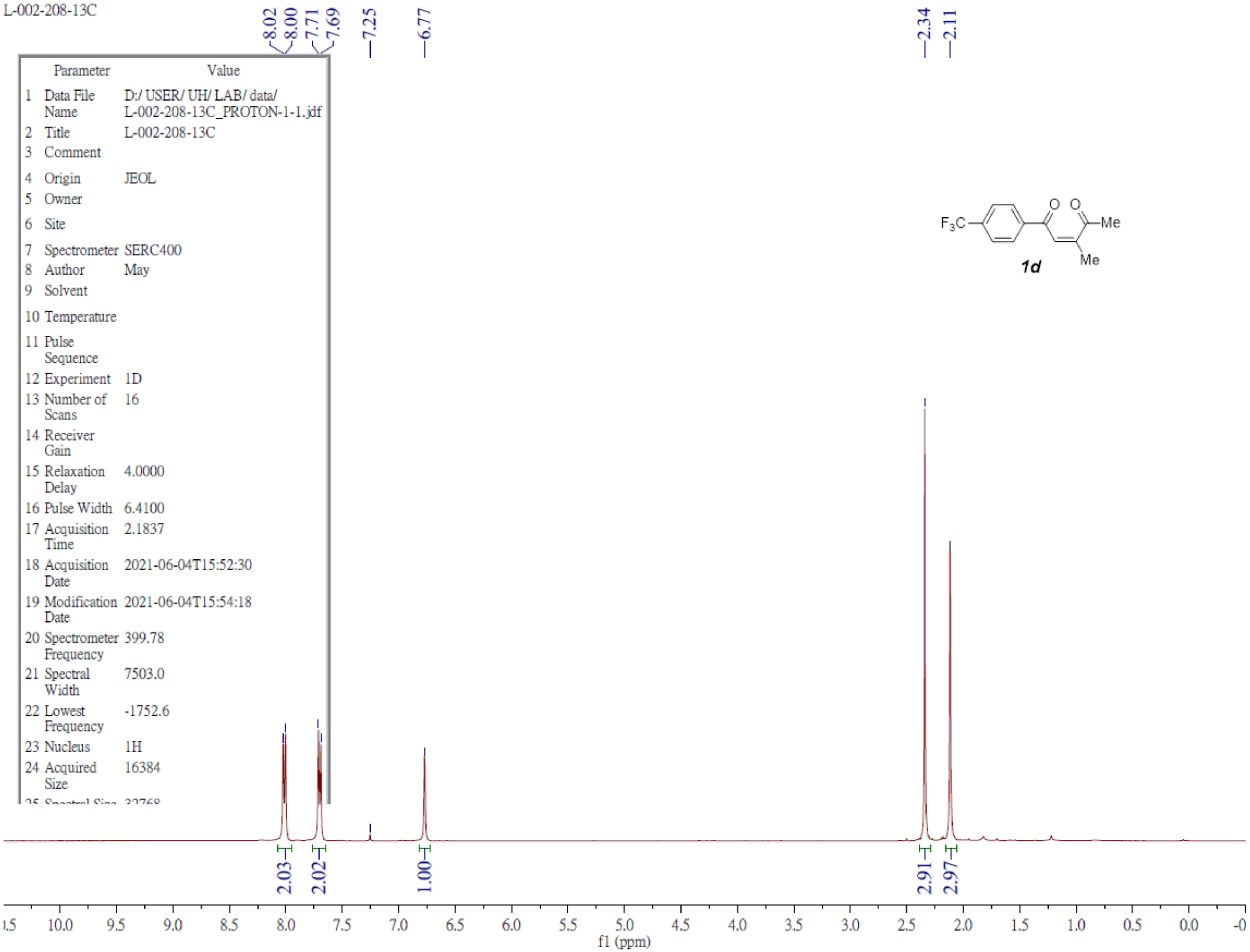
L-002-208-13C
8.02
8.00
7.71
7.69
7.25
6.77
2.34
2.11
Parameter Value
1 Data File Name D:/ USER/ UH/ LAB/ data/ L-002-208-13C_PROTON-1-1.jdf
2 Title L-002-208-13C
3 Comment
4 Origin JEOL
5 Owner
6 Site
7 Spectrometer SERC400
8 Author May
9 Solvent
10 Temperature
11 Pulse Sequence
12 Experiment 1D
13 Number of Scans 16
14 Receiver Gain
15 Relaxation Delay 4.0000
16 Pulse Width 6.4100
17 Acquisition Time 2.1837
18 Acquisition Date 2021-06-04T15:52:30
19 Modification Date 2021-06-04T15:54:18
20 Spectrometer Frequency 399.78
21 Spectral Width 7503.0
22 Lowest Frequency -1752.6
23 Nucleus 1H
24 Acquired Size 16384
2.03
2.02
1.00
2.91
2.97
f1 (ppm)
F3C
O
O
Me
Me

1d

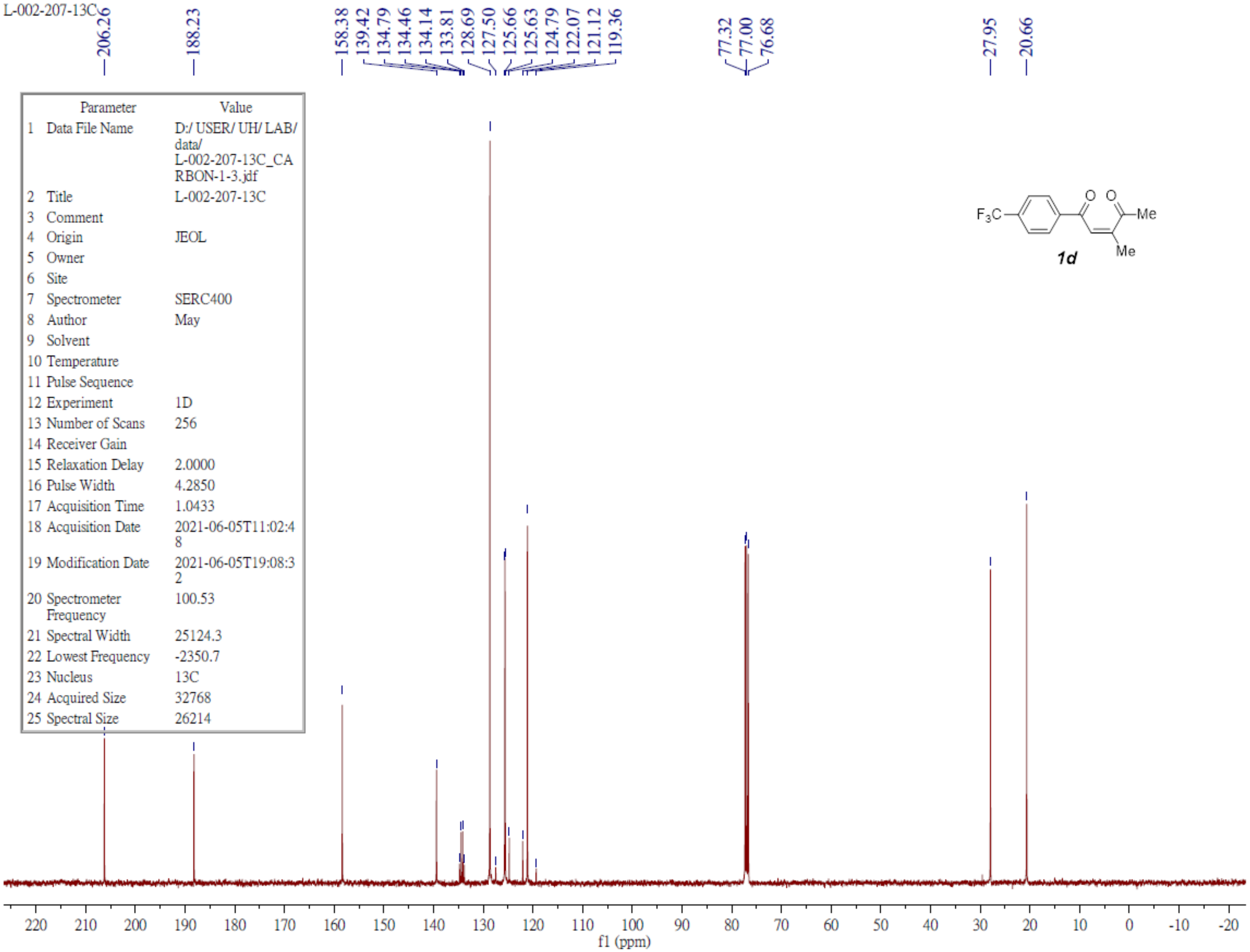
L-002-207-13C
206.26
188.23
158.38
139.42
134.79
134.46
134.14
133.81
128.69
127.50
125.66
125.63
124.79
122.07
121.12
119.36
77.32
77.00
76.68
27.95
20.66
Parameter Value
1 Data File Name D:/ USER/ UH/ LAB/ data/ L-002-207-13C_CARBON-1-3.jdf
2 Title L-002-207-13C
3 Comment
4 Origin JEOL
5 Owner
6 Site
7 Spectrometer SERC400
8 Author May
9 Solvent
10 Temperature
11 Pulse Sequence
12 Experiment 1D
13 Number of Scans 256
14 Receiver Gain
15 Relaxation Delay 2.0000
16 Pulse Width 4.2850
17 Acquisition Time 1.0433
18 Acquisition Date 2021-06-05T11:02:48
19 Modification Date 2021-06-05T19:08:32
20 Spectrometer Frequency 100.53
21 Spectral Width 25124.3
22 Lowest Frequency -2350.7
23 Nucleus 13C
24 Acquired Size 32768
25 Spectral Size 26214
F3C
O
O
Me
Me
1d
220 210 200 190 180 170 160 150 140 130 120 110 100 90 80 70 60 50 40 30 20 10 0 -10 -20
f1 (ppm)

L-002-207-F

	Parameter	Value
1	Data File Name	D:/ USER/ UH/ LAB/ data/ L-002-207-F_Fluorine-2-2.jdf
2	Title	L-002-207-F
3	Comment	
4	Origin	JEOL
5	Owner	
6	Site	
7	Spectrometer	SERC ECZ400S
8	Author	May
9	Solvent	
10	Temperature	
11	Pulse Sequence	
12	Experiment	1D
13	Number of Scans	4
14	Receiver Gain	
15	Relaxation Delay	4.0000
16	Pulse Width	3.1850
17	Acquisition Time	1.9862
18	Acquisition Date	2023-07-14T12:15:51
19	Modification Date	2023-07-14T12:28:45
20	Spectrometer Frequency	376.17
21	Spectral Width	75757.3
22	Lowest Frequency	-75495.8
23	Nucleus	Fluorine19
24	Acquired Size	188087
25	Spectral Size	419430

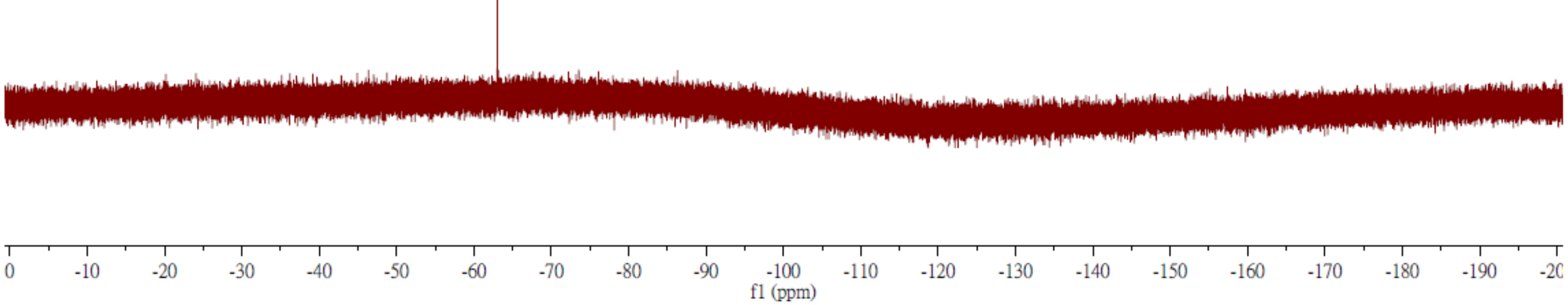

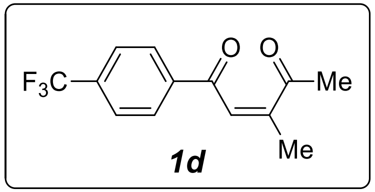

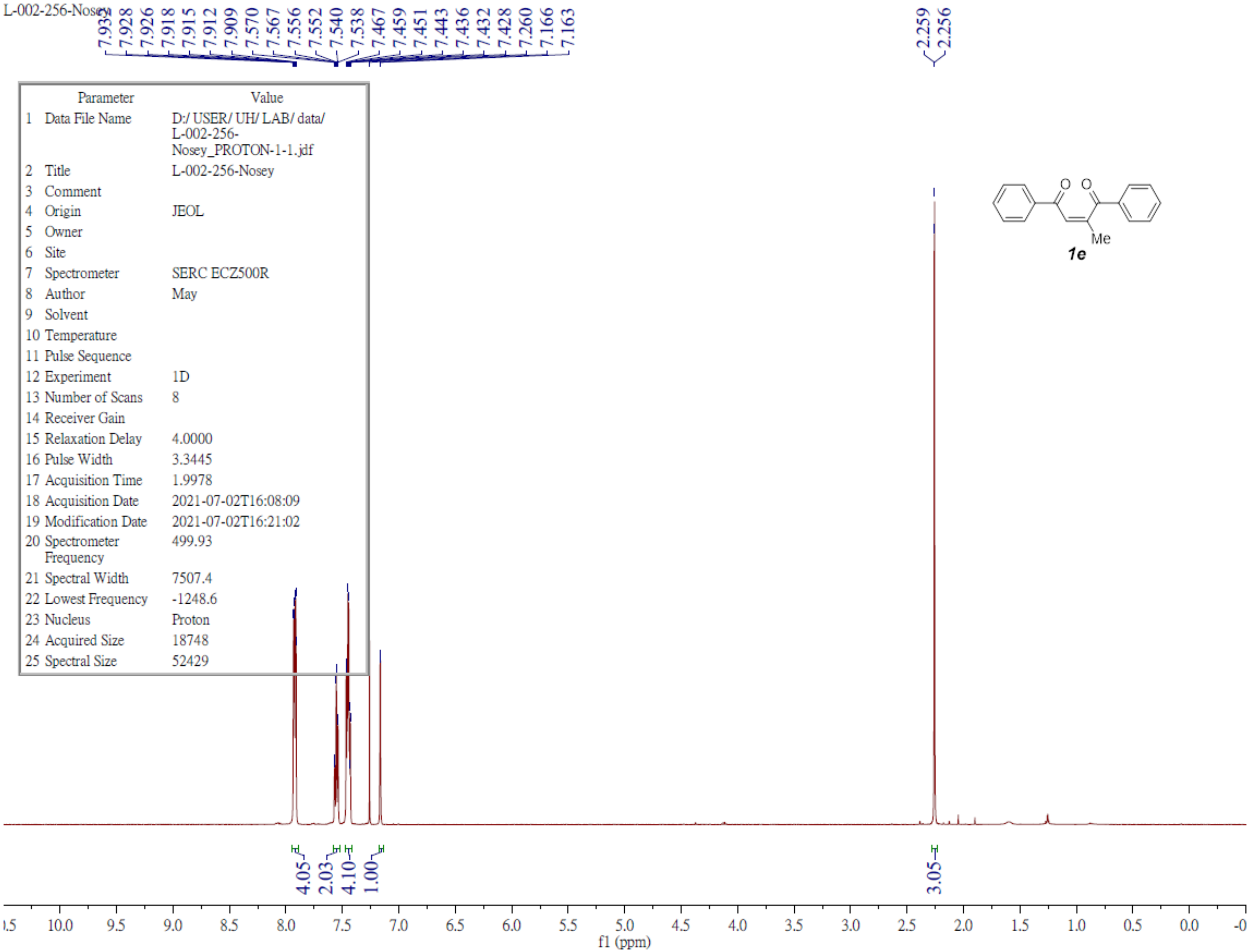

	Parameter	Value
1	Data File Name	D:/ USER/ UH/ LAB/ data/ L-002-256-Nosey_PROTON-1-1.jdf
2	Title	L-002-256-Nosey
3	Comment	
4	Origin	JEOL
5	Owner	
6	Site	
7	Spectrometer	SERC ECZ500R
8	Author	May
9	Solvent	
10	Temperature	
11	Pulse Sequence	
12	Experiment	1D
13	Number of Scans	8
14	Receiver Gain	
15	Relaxation Delay	4.0000
16	Pulse Width	3.3445
17	Acquisition Time	1.9978
18	Acquisition Date	2021-07-02T16:08:09
19	Modification Date	2021-07-02T16:21:02
20	Spectrometer Frequency	499.93
21	Spectral Width	7507.4
22	Lowest Frequency	-1248.6
23	Nucleus	Proton
24	Acquired Size	18748
25	Spectral Size	52429

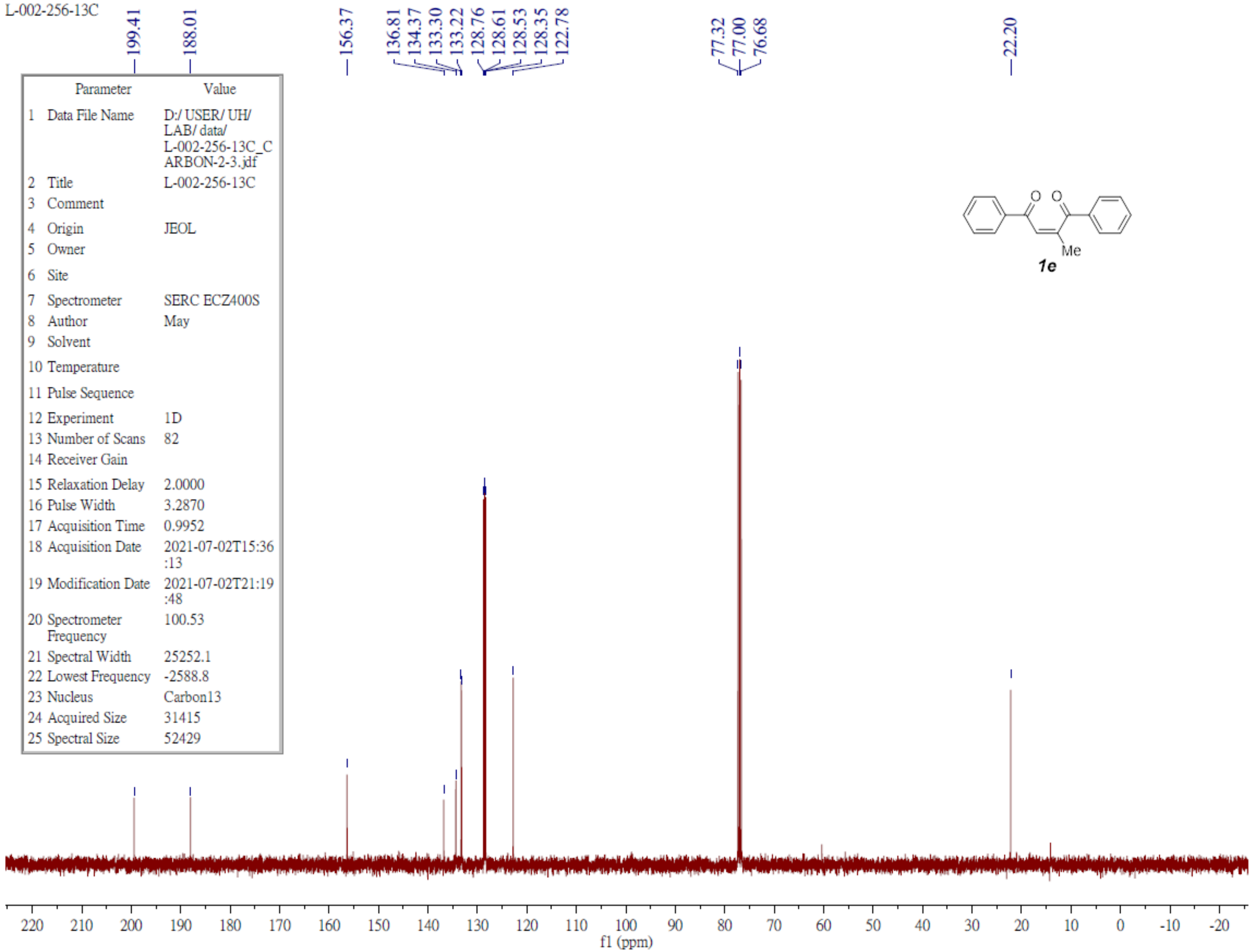

	Parameter	Value
1	Data File Name	D:/ USER/ UH/ LAB/ data/ L-002-256-13C_CARBON-2-3.jdf
2	Title	L-002-256-13C
3	Comment	
4	Origin	JEOL
5	Owner	
6	Site	
7	Spectrometer	SERC ECZ400S
8	Author	May
9	Solvent	
10	Temperature	
11	Pulse Sequence	
12	Experiment	1D
13	Number of Scans	82
14	Receiver Gain	
15	Relaxation Delay	2.0000
16	Pulse Width	3.2870
17	Acquisition Time	0.9952
18	Acquisition Date	2021-07-02T15:36:13
19	Modification Date	2021-07-02T21:19:48
20	Spectrometer Frequency	100.53
21	Spectral Width	25252.1
22	Lowest Frequency	-2588.8
23	Nucleus	Carbon13
24	Acquired Size	31415
25	Spectral Size	52429

1e

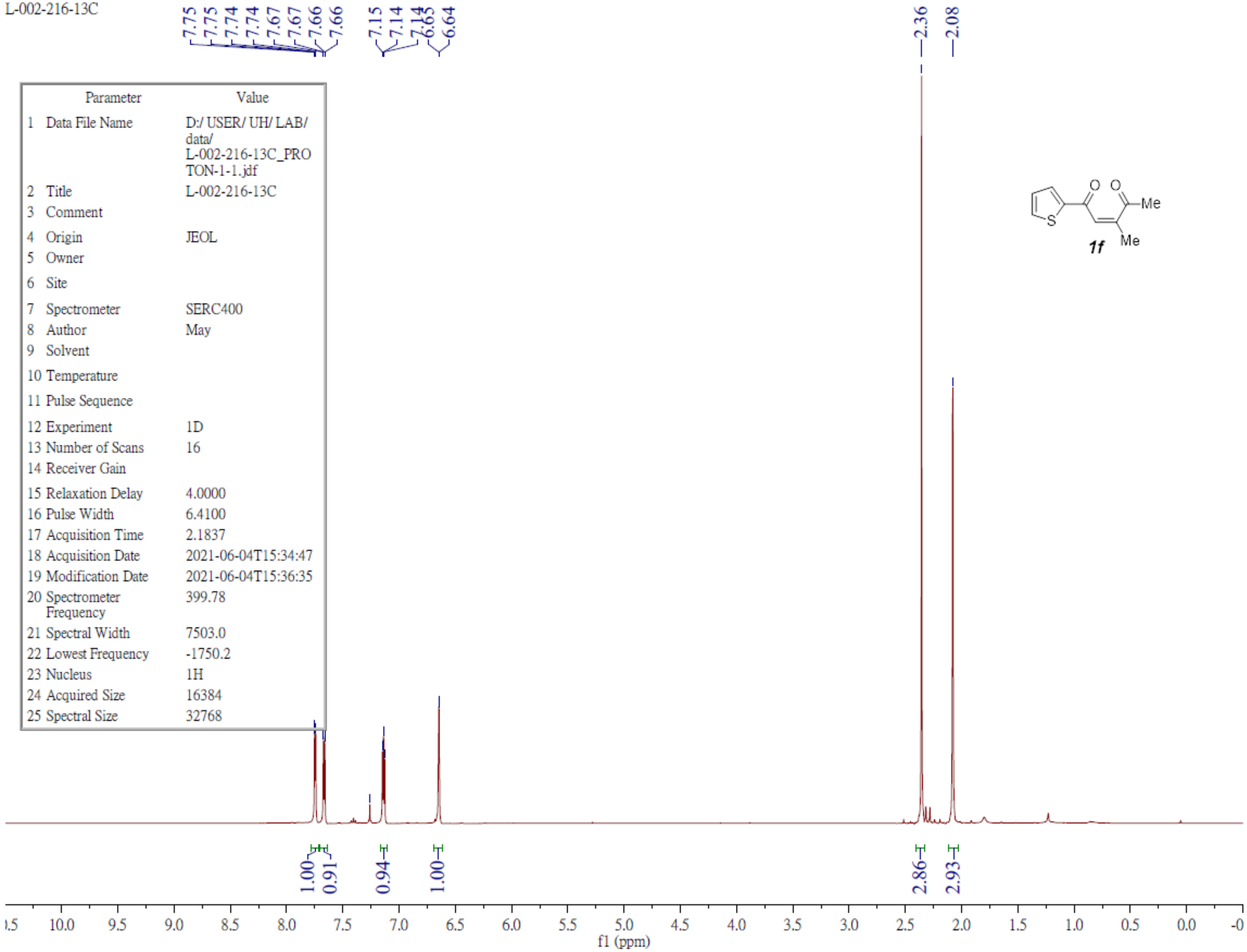

	Parameter	Value
1	Data File Name	D:/ USER/ UH/ LAB/ data/ L-002-216-13C_PROTON-1-1.jdf
2	Title	L-002-216-13C
3	Comment	
4	Origin	JEOL
5	Owner	
6	Site	
7	Spectrometer	SERC400
8	Author	May
9	Solvent	
10	Temperature	
11	Pulse Sequence	
12	Experiment	1D
13	Number of Scans	16
14	Receiver Gain	
15	Relaxation Delay	4.0000
16	Pulse Width	6.4100
17	Acquisition Time	2.1837
18	Acquisition Date	2021-06-04T15:34:47
19	Modification Date	2021-06-04T15:36:35
20	Spectrometer Frequency	399.78
21	Spectral Width	7503.0
22	Lowest Frequency	-1750.2
23	Nucleus	1H
24	Acquired Size	16384
25	Spectral Size	32768

L-002-216-13C

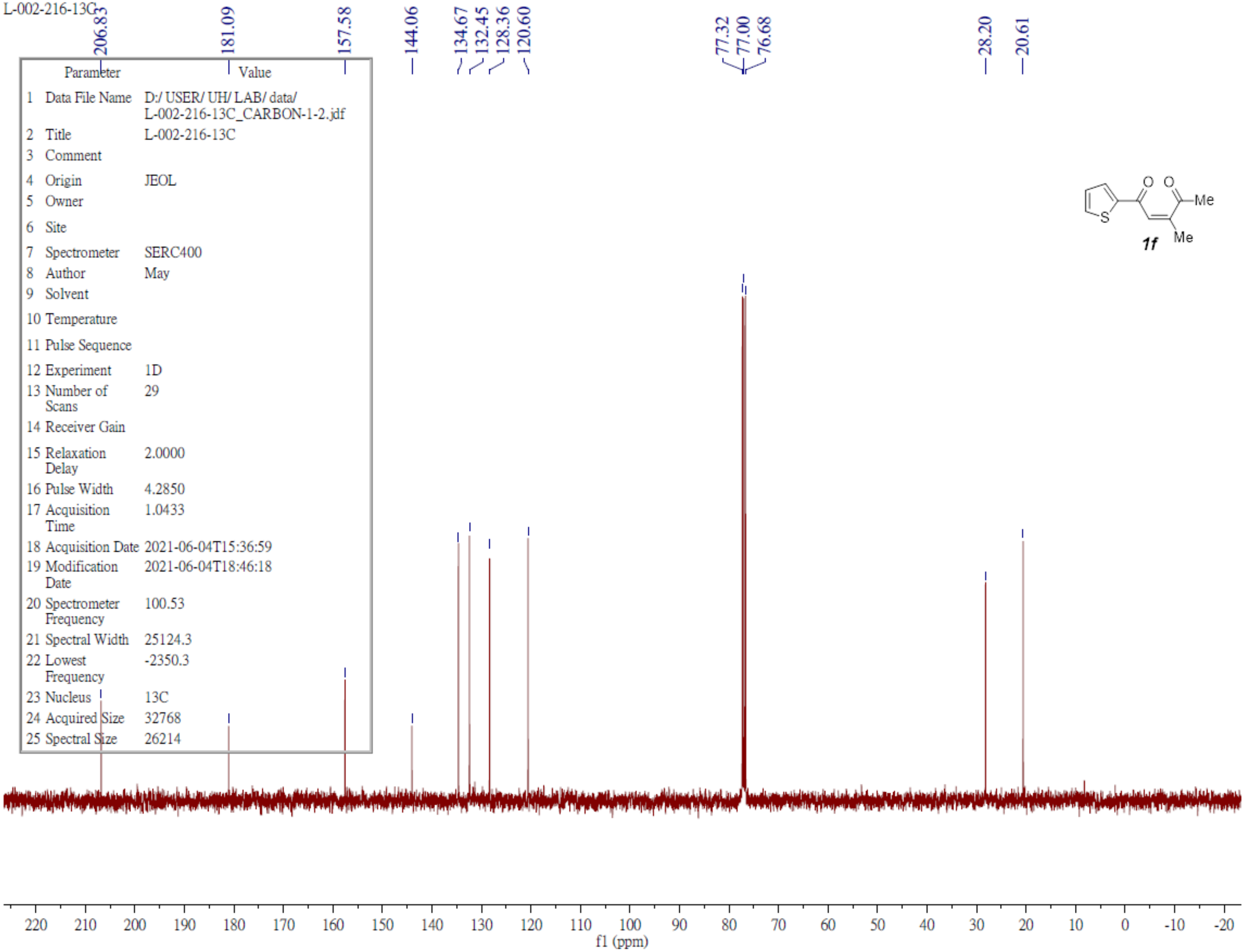

	Parameter	Value
1	Data File Name	D:/ USER/ UH/ LAB/ data/ L-002-216-13C_CARBON-1-2.jdf
2	Title	L-002-216-13C
3	Comment	
4	Origin	JEOL
5	Owner	
6	Site	
7	Spectrometer	SERC400
8	Author	May
9	Solvent	
10	Temperature	
11	Pulse Sequence	
12	Experiment	1D
13	Number of Scans	29
14	Receiver Gain	
15	Relaxation Delay	2.0000
16	Pulse Width	4.2850
17	Acquisition Time	1.0433
18	Acquisition Date	2021-06-04T15:36:59
19	Modification Date	2021-06-04T18:46:18
20	Spectrometer Frequency	100.53
21	Spectral Width	25124.3
22	Lowest Frequency	-2350.3
23	Nucleus	13C
24	Acquired Size	32768
25	Spectral Size	26214

1f

L-002-223

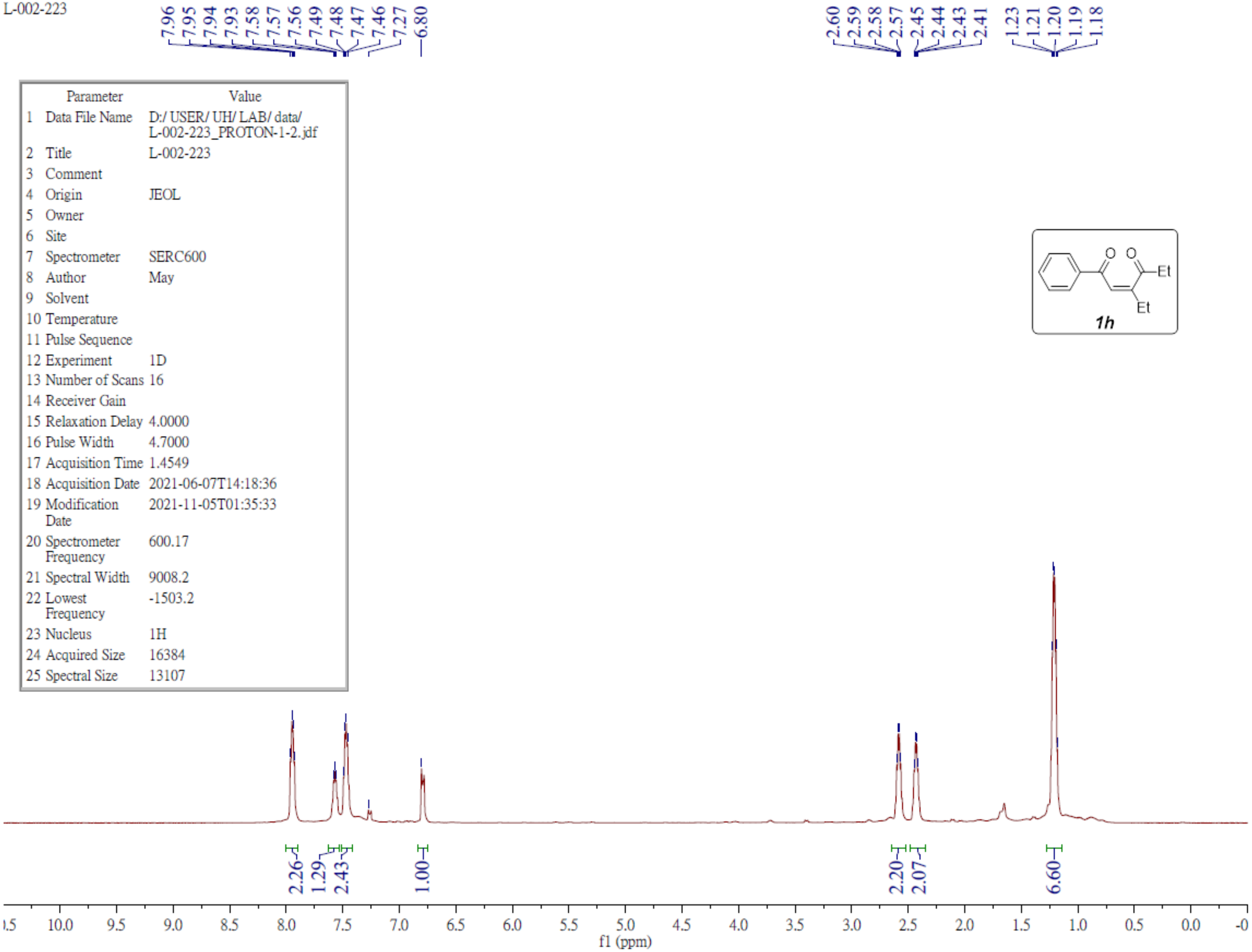

Parameter	Value
1 Data File Name	D:/ USER/ UH/ LAB/ data/ L-002-223_PROTON-1-2.jdf
2 Title	L-002-223
3 Comment	
4 Origin	JEOL
5 Owner	
6 Site	
7 Spectrometer	SERC600
8 Author	May
9 Solvent	
10 Temperature	
11 Pulse Sequence	
12 Experiment	1D
13 Number of Scans	16
14 Receiver Gain	
15 Relaxation Delay	4.0000
16 Pulse Width	4.7000
17 Acquisition Time	1.4549
18 Acquisition Date	2021-06-07T14:18:36
19 Modification Date	2021-11-05T01:35:33
20 Spectrometer Frequency	600.17
21 Spectral Width	9008.2
22 Lowest Frequency	-1503.2
23 Nucleus	1H
24 Acquired Size	16384
25 Spectral Size	13107

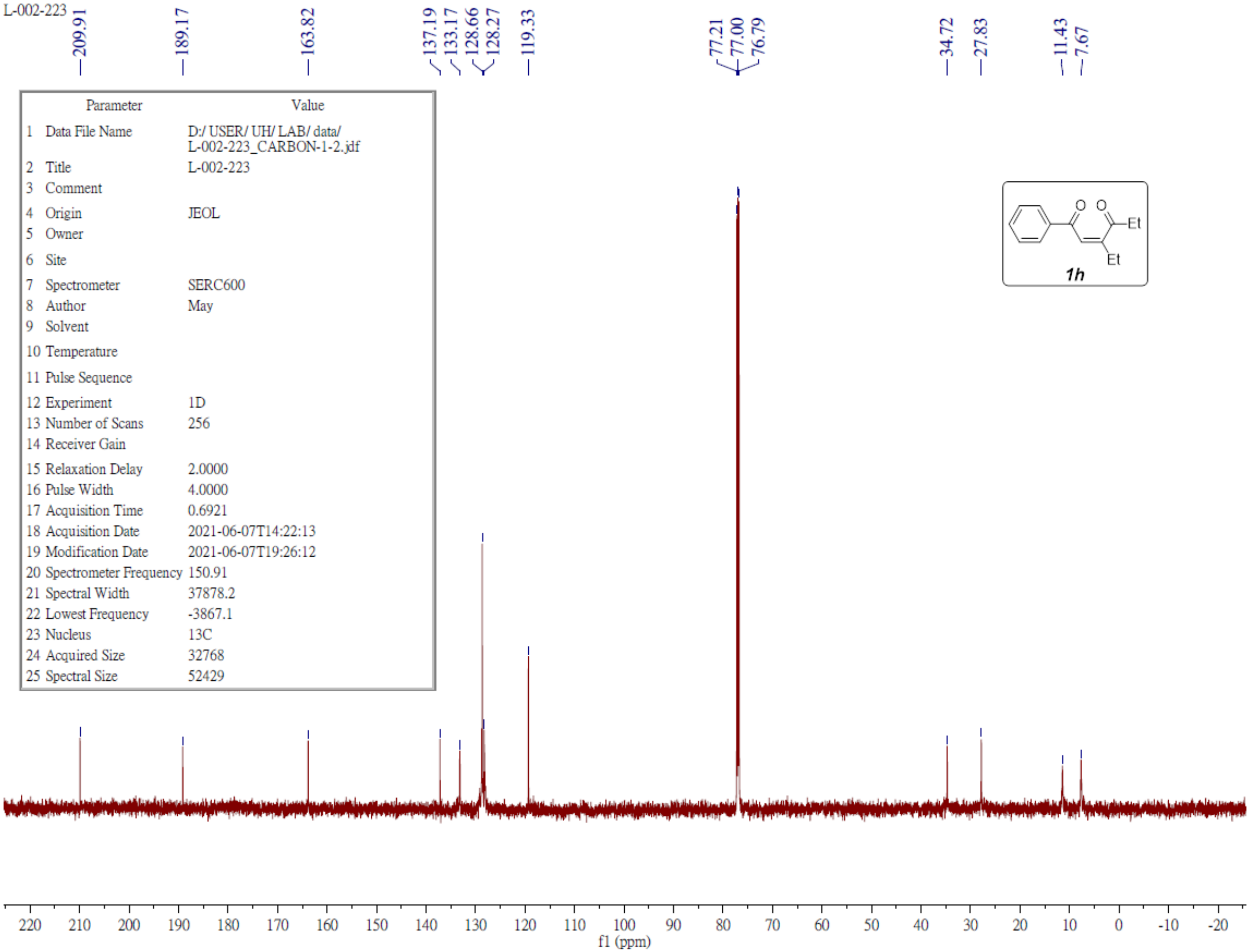
L-002-223
209.91
189.17
163.82
137.19
133.17
128.66
128.27
119.33
77.21
77.00
76.79
34.72
27.83
11.43
7.67
Parameter Value
1 Data File Name D:/ USER/ UH/ LAB/ data/ L-002-223_CARBON-1-2.jdf
2 Title L-002-223
3 Comment
4 Origin JEOL
5 Owner
6 Site
7 Spectrometer SERC600
8 Author May
9 Solvent
10 Temperature
11 Pulse Sequence
12 Experiment 1D
13 Number of Scans 256
14 Receiver Gain
15 Relaxation Delay 2.0000
16 Pulse Width 4.0000
17 Acquisition Time 0.6921
18 Acquisition Date 2021-06-07T14:22:13
19 Modification Date 2021-06-07T19:26:12
20 Spectrometer Frequency 150.91
21 Spectral Width 37878.2
22 Lowest Frequency -3867.1
23 Nucleus 13C
24 Acquired Size 32768
25 Spectral Size 52429
O
O
Et
Et
1h
220 210 200 190 180 170 160 150 140 130 120 110 100 90 80 70 60 50 40 30 20 10 0 -10 -20
f1 (ppm)

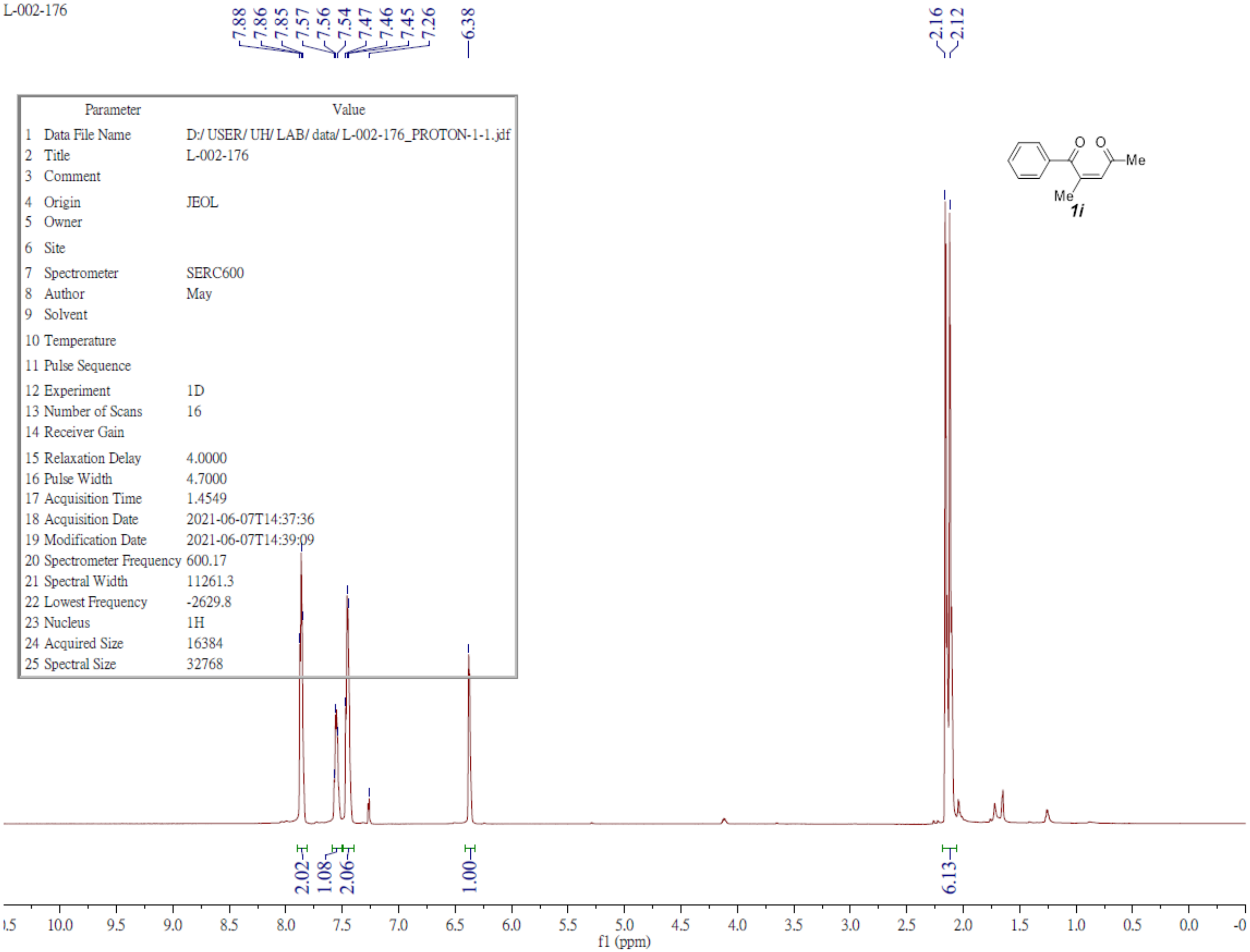

	Parameter	Value
1	Data File Name	D:/ USER/ UH/ LAB/ data/ L-002-176_PROTON-1-1.jdf
2	Title	L-002-176
3	Comment	
4	Origin	JEOL
5	Owner	
6	Site	
7	Spectrometer	SERC600
8	Author	May
9	Solvent	
10	Temperature	
11	Pulse Sequence	
12	Experiment	1D
13	Number of Scans	16
14	Receiver Gain	
15	Relaxation Delay	4.0000
16	Pulse Width	4.7000
17	Acquisition Time	1.4549
18	Acquisition Date	2021-06-07T14:37:36
19	Modification Date	2021-06-07T14:39:09
20	Spectrometer Frequency	600.17
21	Spectral Width	11261.3
22	Lowest Frequency	-2629.8
23	Nucleus	1H
24	Acquired Size	16384
25	Spectral Size	32768

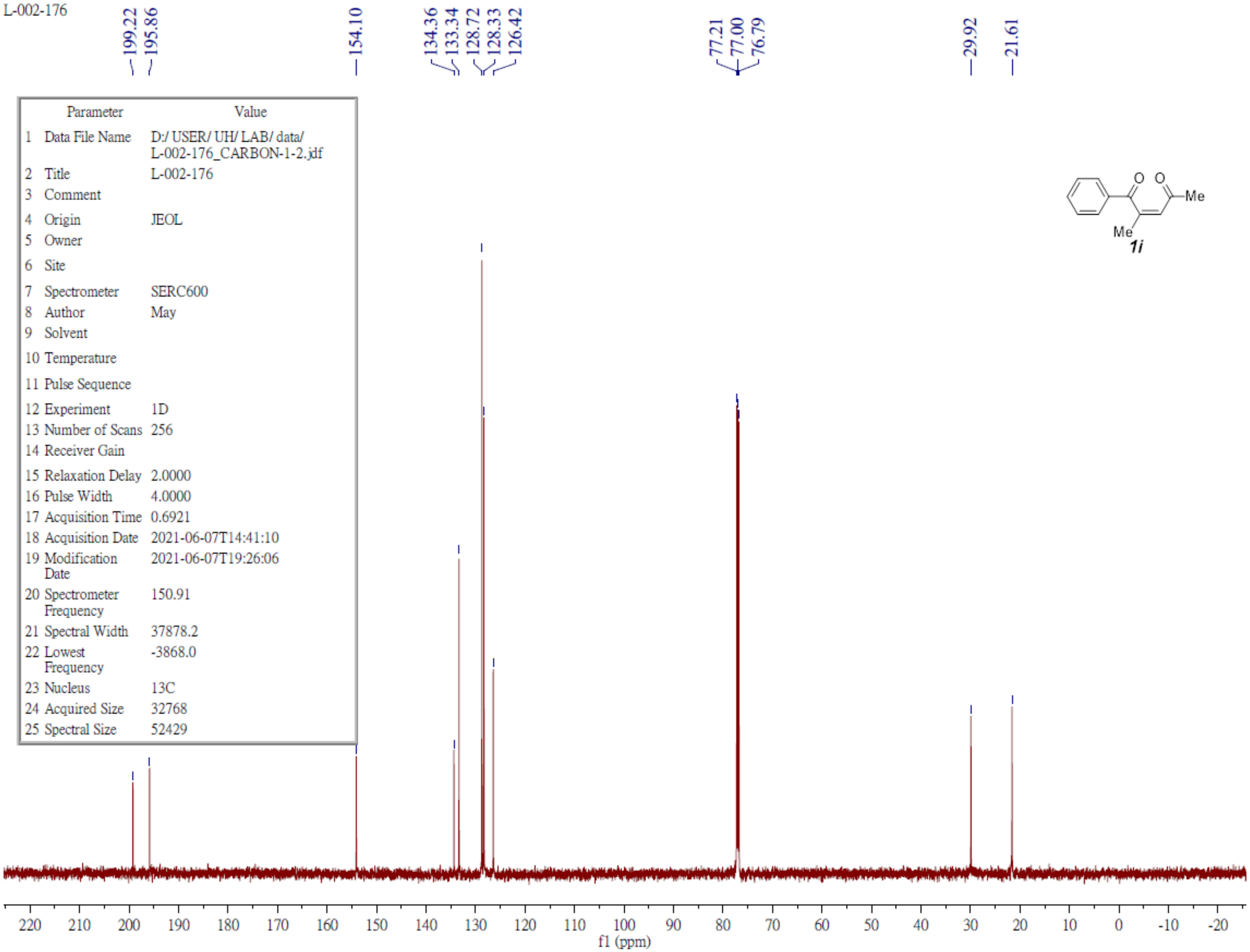

	Parameter	Value
1	Data File Name	D:/ USER/ UH/ LAB/ data/ L-002-176_CARBON-1-2.jdf
2	Title	L-002-176
3	Comment	
4	Origin	JEOL
5	Owner	
6	Site	
7	Spectrometer	SERC600
8	Author	May
9	Solvent	
10	Temperature	
11	Pulse Sequence	
12	Experiment	1D
13	Number of Scans	256
14	Receiver Gain	
15	Relaxation Delay	2.0000
16	Pulse Width	4.0000
17	Acquisition Time	0.6921
18	Acquisition Date	2021-06-07T14:41:10
19	Modification Date	2021-06-07T19:26:06
20	Spectrometer Frequency	150.91
21	Spectral Width	37878.2
22	Lowest Frequency	-3868.0
23	Nucleus	13C
24	Acquired Size	32768
25	Spectral Size	52429

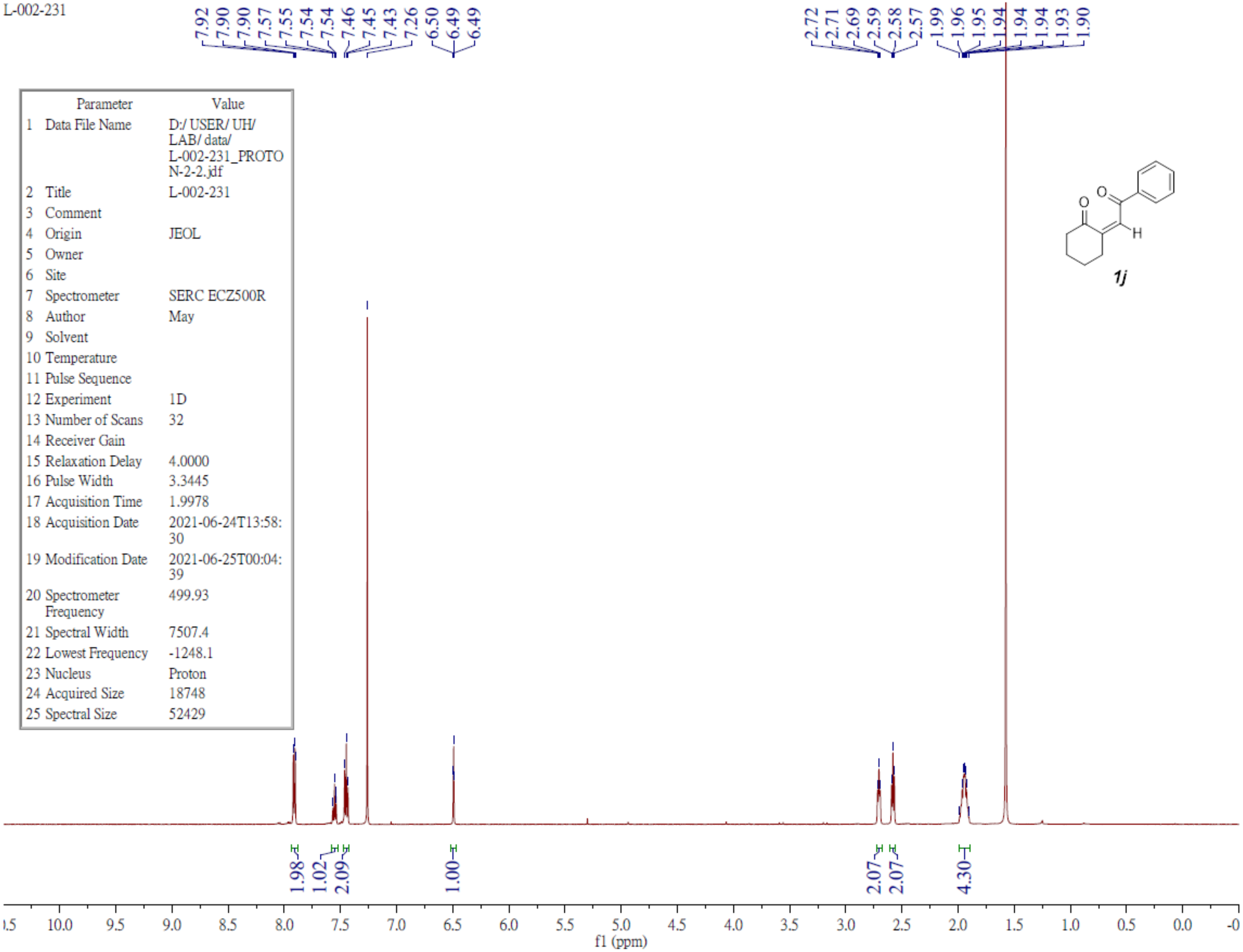

	Parameter	Value
1	Data File Name	D:/ USER/ UH/ LAB/ data/ L-002-231_PROTON-2-2.jdf
2	Title	L-002-231
3	Comment	
4	Origin	JEOL
5	Owner	
6	Site	
7	Spectrometer	SERC ECZ500R
8	Author	May
9	Solvent	
10	Temperature	
11	Pulse Sequence	
12	Experiment	1D
13	Number of Scans	32
14	Receiver Gain	
15	Relaxation Delay	4.0000
16	Pulse Width	3.3445
17	Acquisition Time	1.9978
18	Acquisition Date	2021-06-24T13:58:30
19	Modification Date	2021-06-25T00:04:39
20	Spectrometer Frequency	499.93
21	Spectral Width	7507.4
22	Lowest Frequency	-1248.1
23	Nucleus	Proton
24	Acquired Size	18748
25	Spectral Size	52429

1j

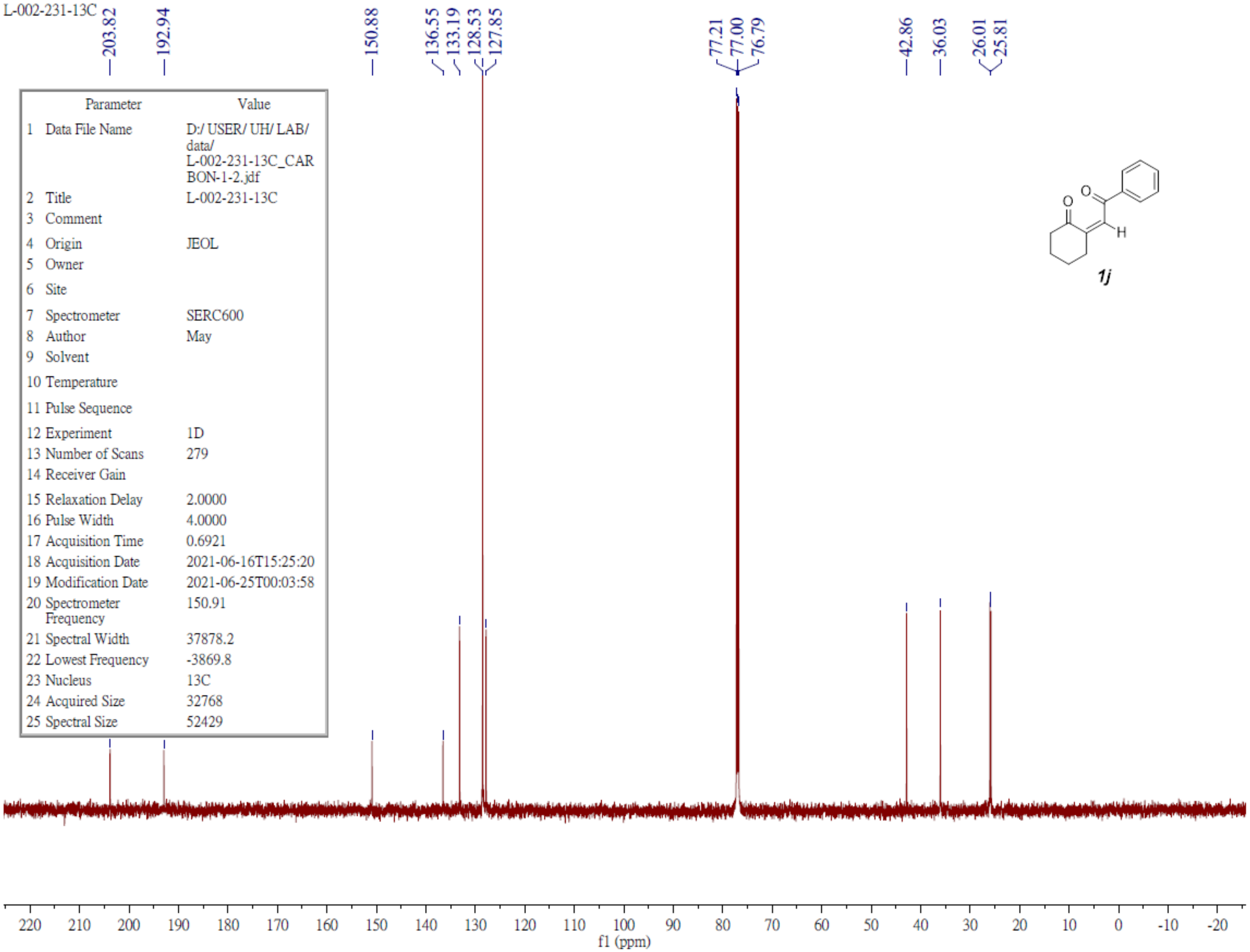

	Parameter	Value
1	Data File Name	D:/ USER/ UH/ LAB/ data/ L-002-231-13C_CARBON-1-2.jdf
2	Title	L-002-231-13C
3	Comment	
4	Origin	JEOL
5	Owner	
6	Site	
7	Spectrometer	SERC600
8	Author	May
9	Solvent	
10	Temperature	
11	Pulse Sequence	
12	Experiment	1D
13	Number of Scans	279
14	Receiver Gain	
15	Relaxation Delay	2.0000
16	Pulse Width	4.0000
17	Acquisition Time	0.6921
18	Acquisition Date	2021-06-16T15:25:20
19	Modification Date	2021-06-25T00:03:58
20	Spectrometer Frequency	150.91
21	Spectral Width	37878.2
22	Lowest Frequency	-3869.8
23	Nucleus	13C
24	Acquired Size	32768
25	Spectral Size	52429

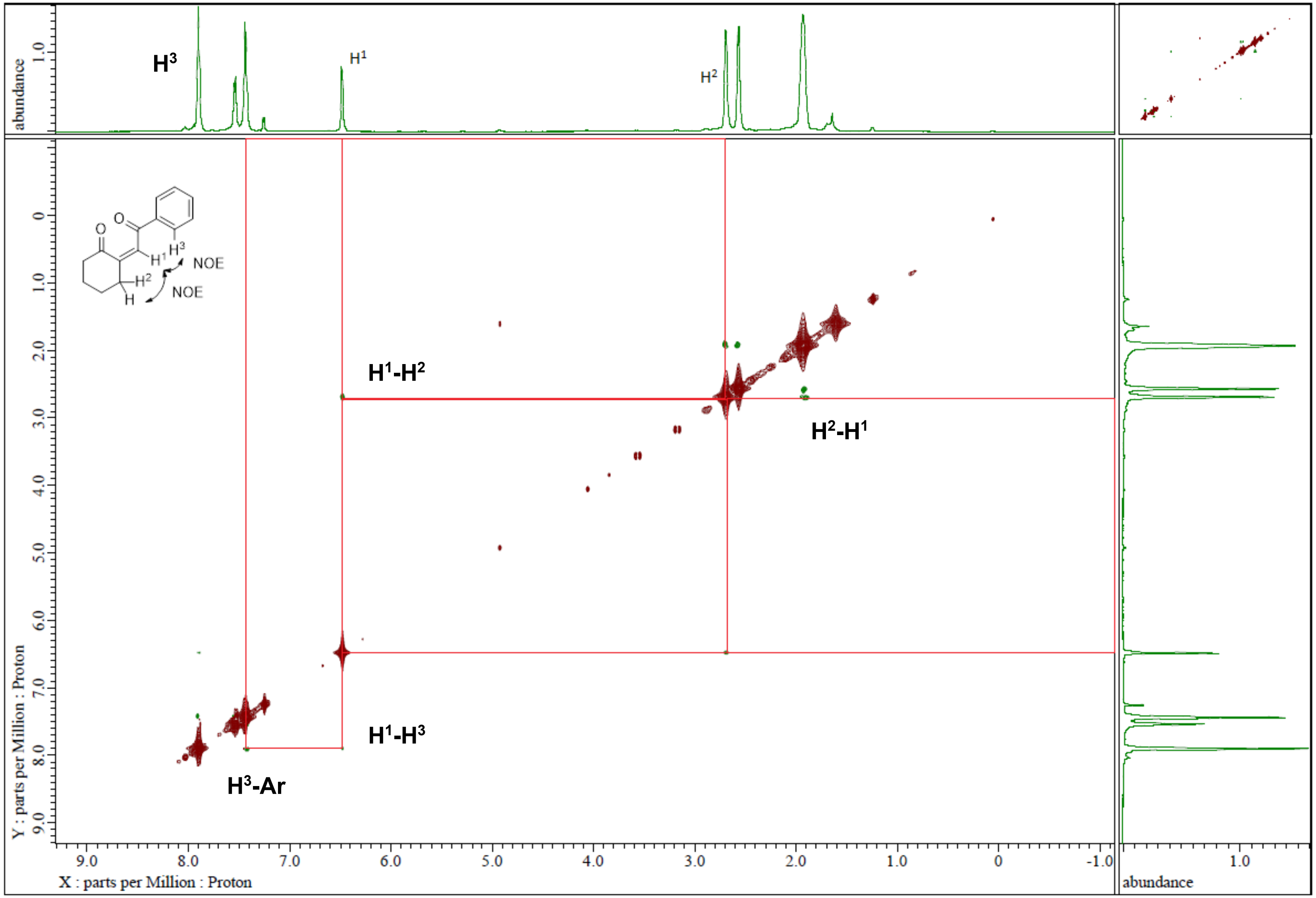

NOSEY spectrum of 1j

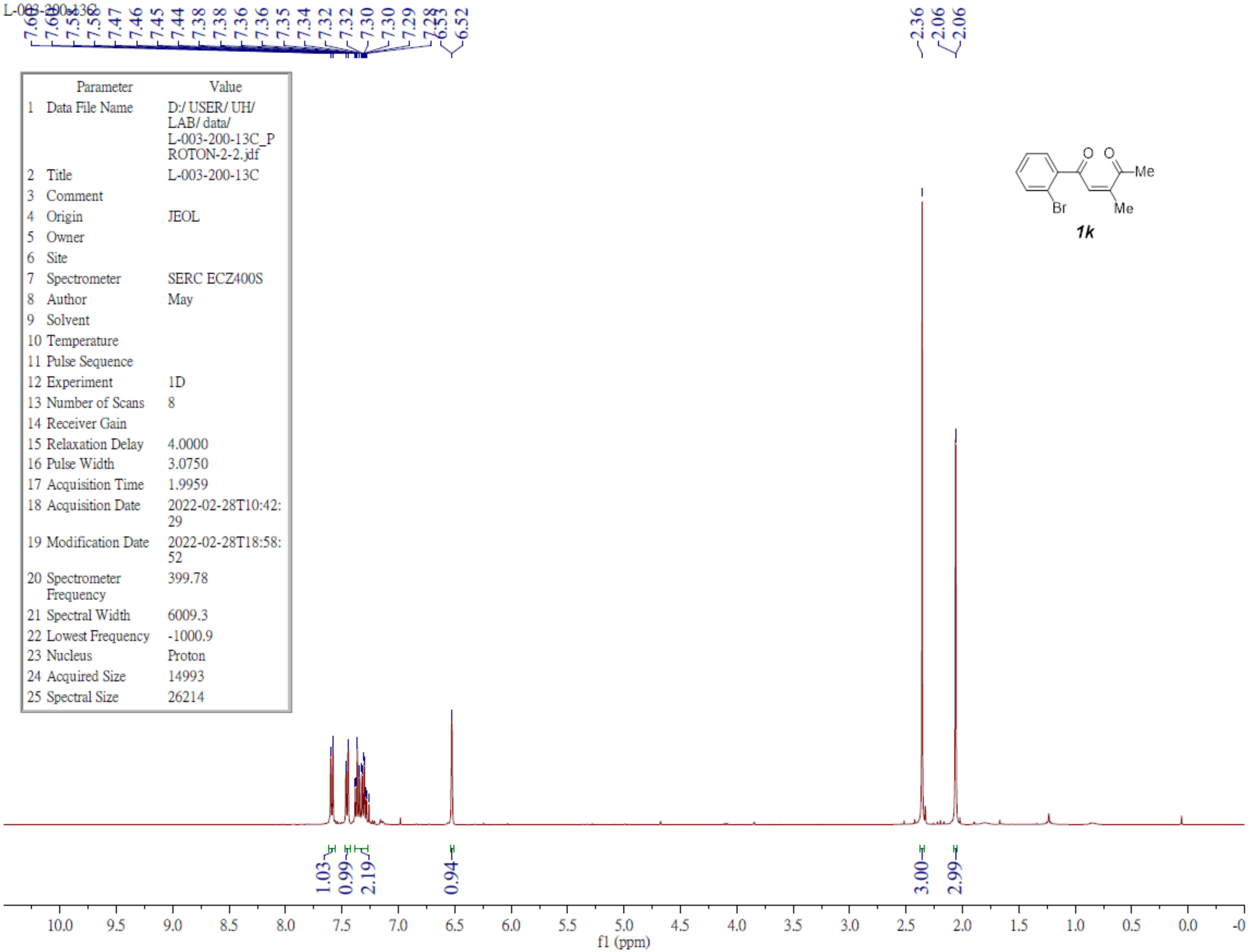

Parameter	Value
1 Data File Name	D:/ USER/ UH/ LAB/ data/ L-003-200-13C_PROTON-2-2.jdf
2 Title	L-003-200-13C
3 Comment	
4 Origin	JEOL
5 Owner	
6 Site	
7 Spectrometer	SERC ECZ400S
8 Author	May
9 Solvent	
10 Temperature	
11 Pulse Sequence	
12 Experiment	1D
13 Number of Scans	8
14 Receiver Gain	
15 Relaxation Delay	4.0000
16 Pulse Width	3.0750
17 Acquisition Time	1.9959
18 Acquisition Date	2022-02-28T10:42:29
19 Modification Date	2022-02-28T18:58:52
20 Spectrometer Frequency	399.78
21 Spectral Width	6009.3
22 Lowest Frequency	-1000.9
23 Nucleus	Proton
24 Acquired Size	14993
25 Spectral Size	26214

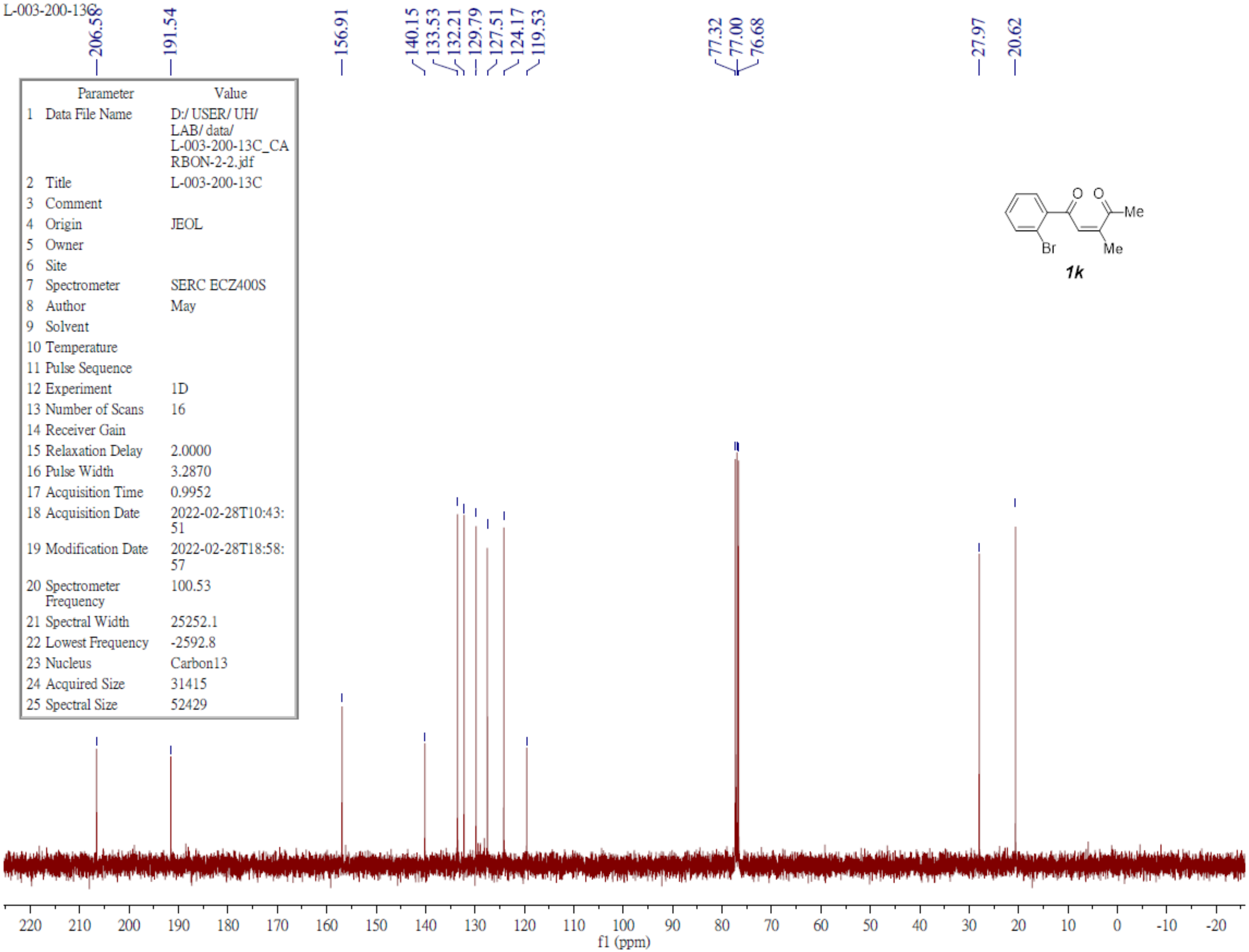

1k

	Parameter	Value
1	Data File Name	D:/ USER/ UH/ LAB/ data/ L-003-200-13C_CARBON-2-2.jdf
2	Title	L-003-200-13C
3	Comment	
4	Origin	JEOL
5	Owner	
6	Site	
7	Spectrometer	SERC ECZ400S
8	Author	May
9	Solvent	
10	Temperature	
11	Pulse Sequence	
12	Experiment	1D
13	Number of Scans	16
14	Receiver Gain	
15	Relaxation Delay	2.0000
16	Pulse Width	3.2870
17	Acquisition Time	0.9952
18	Acquisition Date	2022-02-28T10:43:51
19	Modification Date	2022-02-28T18:58:57
20	Spectrometer Frequency	100.53
21	Spectral Width	25252.1
22	Lowest Frequency	-2592.8
23	Nucleus	Carbon13
24	Acquired Size	31415
25	Spectral Size	52429

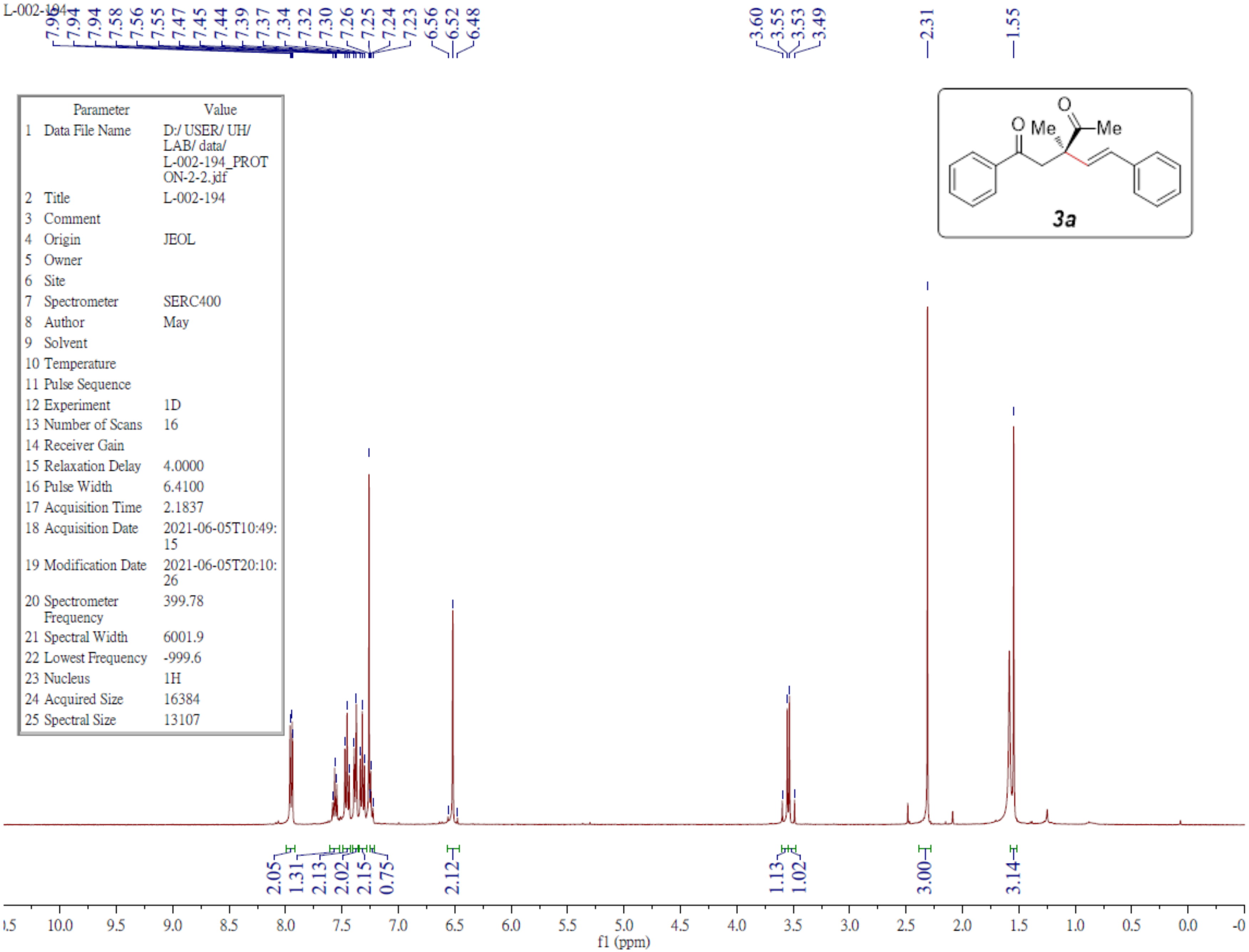

	Parameter	Value
1	Data File Name	D:/ USER/ UH/ LAB/ data/ L-002-194_PROTON-2-2.jdf
2	Title	L-002-194
3	Comment	
4	Origin	JEOL
5	Owner	
6	Site	
7	Spectrometer	SERC400
8	Author	May
9	Solvent	
10	Temperature	
11	Pulse Sequence	
12	Experiment	1D
13	Number of Scans	16
14	Receiver Gain	
15	Relaxation Delay	4.0000
16	Pulse Width	6.4100
17	Acquisition Time	2.1837
18	Acquisition Date	2021-06-05T10:49:15
19	Modification Date	2021-06-05T20:10:26
20	Spectrometer Frequency	399.78
21	Spectral Width	6001.9
22	Lowest Frequency	-999.6
23	Nucleus	1H
24	Acquired Size	16384
25	Spectral Size	13107

3a

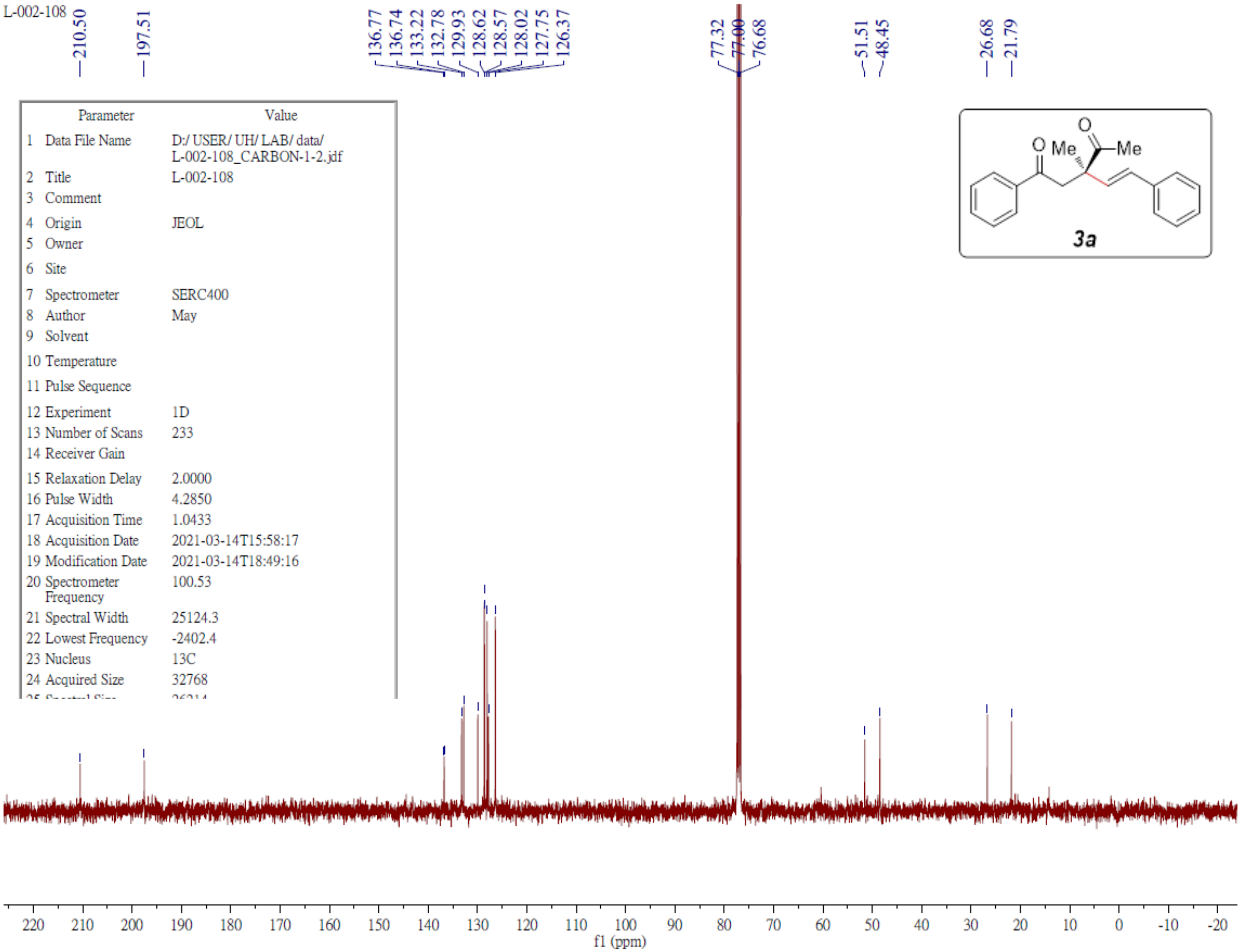
L-002-108
210.50
197.51
136.77
136.74
133.22
132.78
129.93
128.62
128.57
128.02
127.75
126.37
77.32
77.00
76.68
51.51
48.45
26.68
21.79
Parameter
Value
1 Data File Name
D:/ USER/ UH/ LAB/ data/ L-002-108_CARBON-1-2.jdf
2 Title
L-002-108
3 Comment
4 Origin
JEOL
5 Owner
6 Site
7 Spectrometer
SERC400
8 Author
May
9 Solvent
10 Temperature
11 Pulse Sequence
12 Experiment
1D
13 Number of Scans
233
14 Receiver Gain
15 Relaxation Delay
2.0000
16 Pulse Width
4.2850
17 Acquisition Time
1.0433
18 Acquisition Date
2021-03-14T15:58:17
19 Modification Date
2021-03-14T18:49:16
20 Spectrometer Frequency
100.53
21 Spectral Width
25124.3
22 Lowest Frequency
-2402.4
23 Nucleus
13C
24 Acquired Size
32768
O
Me
O
Me
3a
220
210
200
190
180
170
160
150
140
130
120
110
100
90
80
70
60
50
40
30
20
10
0
-10
-20
f1 (ppm)

DEPT-135 spectrum of 4a

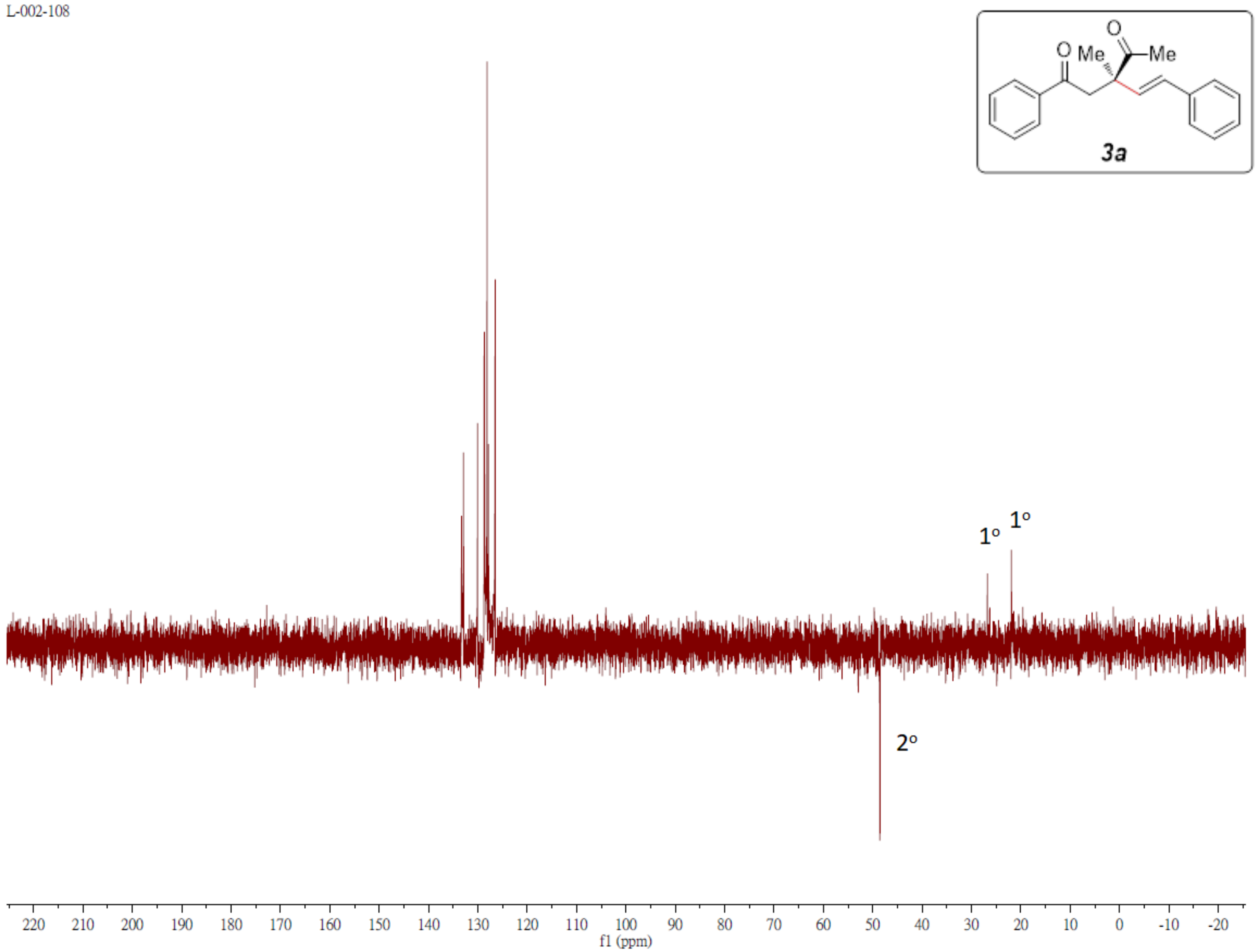

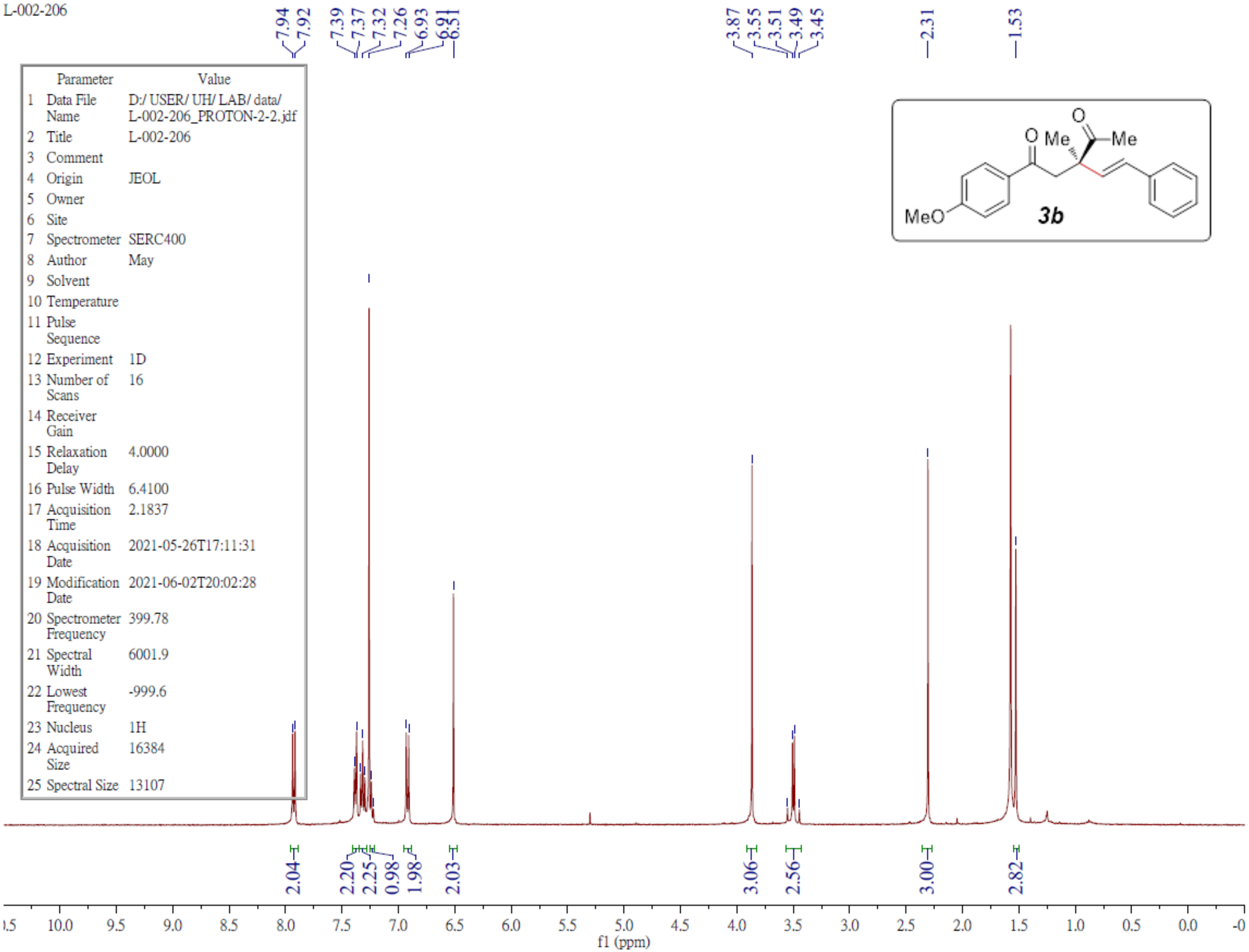

Parameter		Value
1	Data File Name	D:/ USER/ UH/ LAB/ data/ L-002-206_PROTON-2-2.jdf
2	Title	L-002-206
3	Comment	
4	Origin	JEOL
5	Owner	
6	Site	
7	Spectrometer	SERC400
8	Author	May
9	Solvent	
10	Temperature	
11	Pulse Sequence	
12	Experiment	1D
13	Number of Scans	16
14	Receiver Gain	
15	Relaxation Delay	4.0000
16	Pulse Width	6.4100
17	Acquisition Time	2.1837
18	Acquisition Date	2021-05-26T17:11:31
19	Modification Date	2021-06-02T20:02:28
20	Spectrometer Frequency	399.78
21	Spectral Width	6001.9
22	Lowest Frequency	-999.6
23	Nucleus	1H
24	Acquired Size	16384
25	Spectral Size	13107

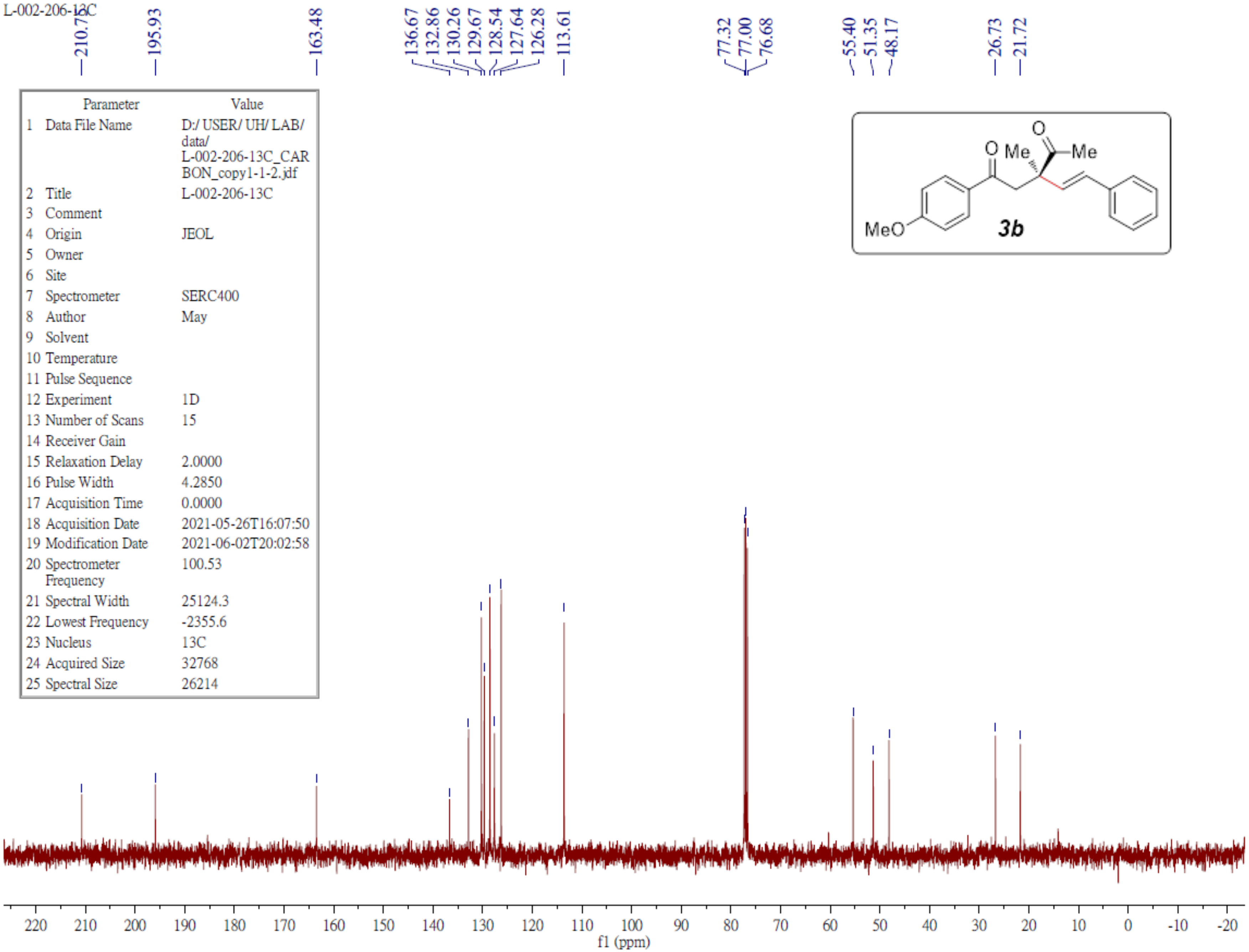

	Parameter	Value
1	Data File Name	D:/ USER/ UH/ LAB/ data/ L-002-206-13C_CARBON_copy1-1-2.jdf
2	Title	L-002-206-13C
3	Comment	
4	Origin	JEOL
5	Owner	
6	Site	
7	Spectrometer	SERC400
8	Author	May
9	Solvent	
10	Temperature	
11	Pulse Sequence	
12	Experiment	1D
13	Number of Scans	15
14	Receiver Gain	
15	Relaxation Delay	2.0000
16	Pulse Width	4.2850
17	Acquisition Time	0.0000
18	Acquisition Date	2021-05-26T16:07:50
19	Modification Date	2021-06-02T20:02:58
20	Spectrometer Frequency	100.53
21	Spectral Width	25124.3
22	Lowest Frequency	-2355.6
23	Nucleus	13C
24	Acquired Size	32768
25	Spectral Size	26214

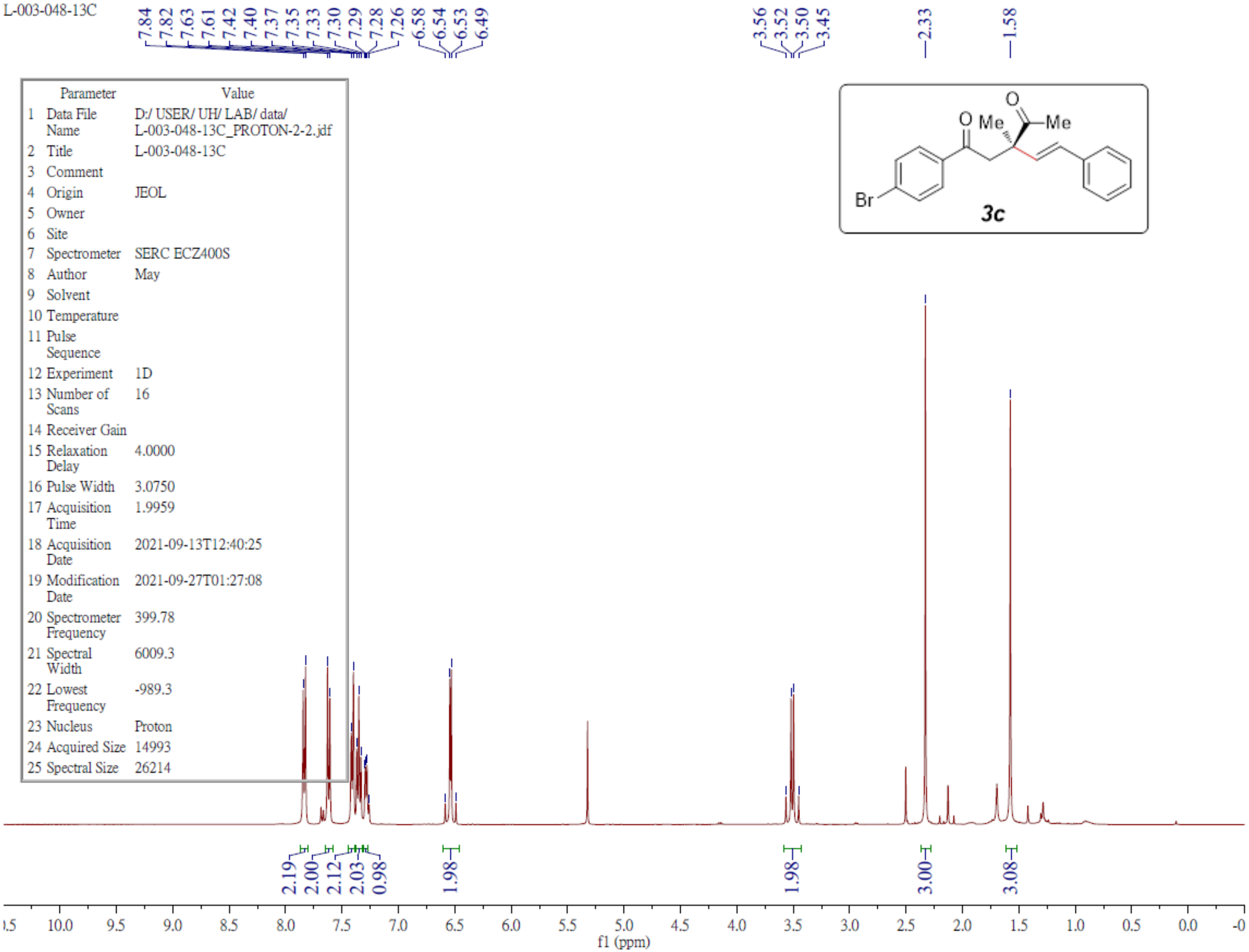
L-003-048-13C
7.84 7.82 7.63 7.61 7.42 7.40 7.37 7.35 7.33 7.30 7.29 7.28 7.26 6.58 6.54 6.53 6.49
3.56 3.52 3.50 3.45
2.33
1.58
Parameter Value
1 Data File Name D:/ USER/ UH/ LAB/ data/ L-003-048-13C_PROTON-2-2.jdf
2 Title L-003-048-13C
3 Comment
4 Origin JEOL
5 Owner
6 Site
7 Spectrometer SERC ECZ400S
8 Author May
9 Solvent
10 Temperature
11 Pulse Sequence
12 Experiment 1D
13 Number of Scans 16
14 Receiver Gain
15 Relaxation Delay 4.0000
16 Pulse Width 3.0750
17 Acquisition Time 1.9959
18 Acquisition Date 2021-09-13T12:40:25
19 Modification Date 2021-09-27T01:27:08
20 Spectrometer Frequency 399.78
21 Spectral Width 6009.3
22 Lowest Frequency -989.3
23 Nucleus Proton
24 Acquired Size 14993
25 Spectral Size 26214
O
O Me
Me
Br
3c
2.19 2.00 2.12 2.03 0.98
1.98
1.98
3.00
3.08
10.0 9.5 9.0 8.5 8.0 7.5 7.0 6.5 6.0 5.5 5.0 4.5 4.0 3.5 3.0 2.5 2.0 1.5 1.0 0.5 0.0
f1 (ppm)

L-003-048-13C

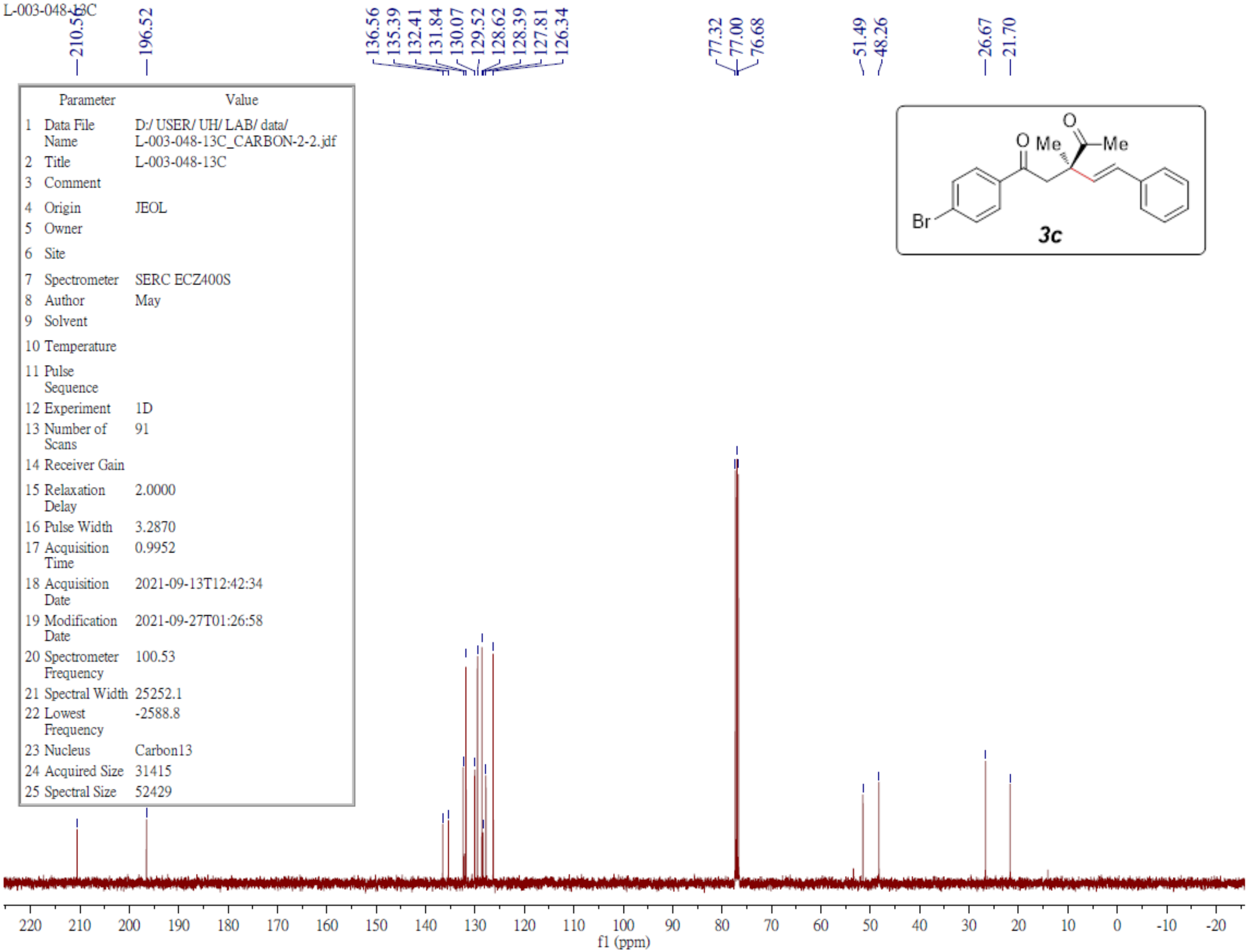

	Parameter	Value
1	Data File Name	D:/ USER/ UH/ LAB/ data/ L-003-048-13C_CARBON-2-2.jdf
2	Title	L-003-048-13C
3	Comment	
4	Origin	JEOL
5	Owner	
6	Site	
7	Spectrometer	SERC ECZ400S
8	Author	May
9	Solvent	
10	Temperature	
11	Pulse Sequence	
12	Experiment	1D
13	Number of Scans	91
14	Receiver Gain	
15	Relaxation Delay	2.0000
16	Pulse Width	3.2870
17	Acquisition Time	0.9952
18	Acquisition Date	2021-09-13T12:42:34
19	Modification Date	2021-09-27T01:26:58
20	Spectrometer Frequency	100.53
21	Spectral Width	25252.1
22	Lowest Frequency	-2588.8
23	Nucleus	Carbon13
24	Acquired Size	31415
25	Spectral Size	52429

3c

L-002-208-13C

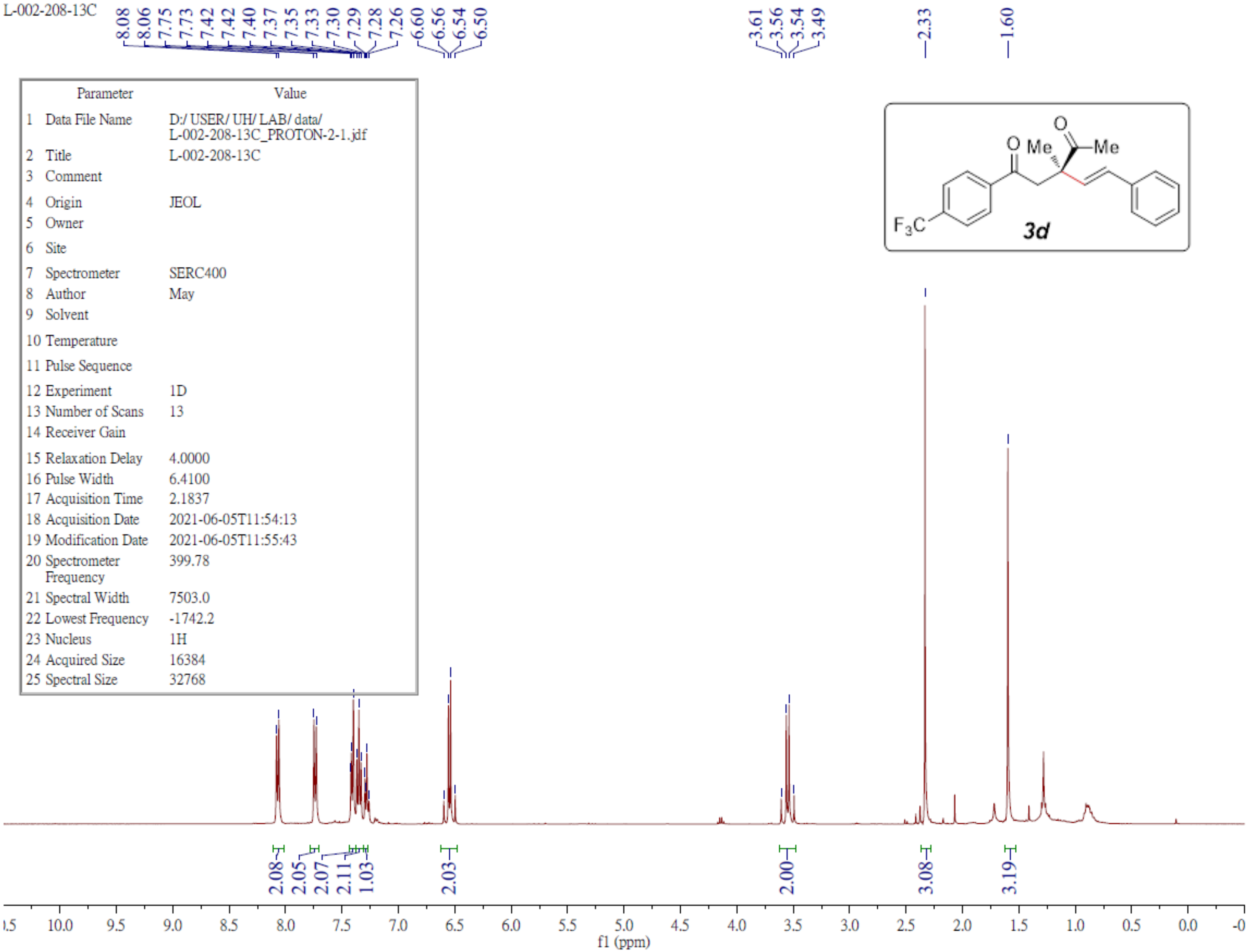

	Parameter	Value
1	Data File Name	D:/ USER/ UH/ LAB/ data/ L-002-208-13C_PROTON-2-1.jdf
2	Title	L-002-208-13C
3	Comment	
4	Origin	JEOL
5	Owner	
6	Site	
7	Spectrometer	SERC400
8	Author	May
9	Solvent	
10	Temperature	
11	Pulse Sequence	
12	Experiment	1D
13	Number of Scans	13
14	Receiver Gain	
15	Relaxation Delay	4.0000
16	Pulse Width	6.4100
17	Acquisition Time	2.1837
18	Acquisition Date	2021-06-05T11:54:13
19	Modification Date	2021-06-05T11:55:43
20	Spectrometer Frequency	399.78
21	Spectral Width	7503.0
22	Lowest Frequency	-1742.2
23	Nucleus	1H
24	Acquired Size	16384
25	Spectral Size	32768

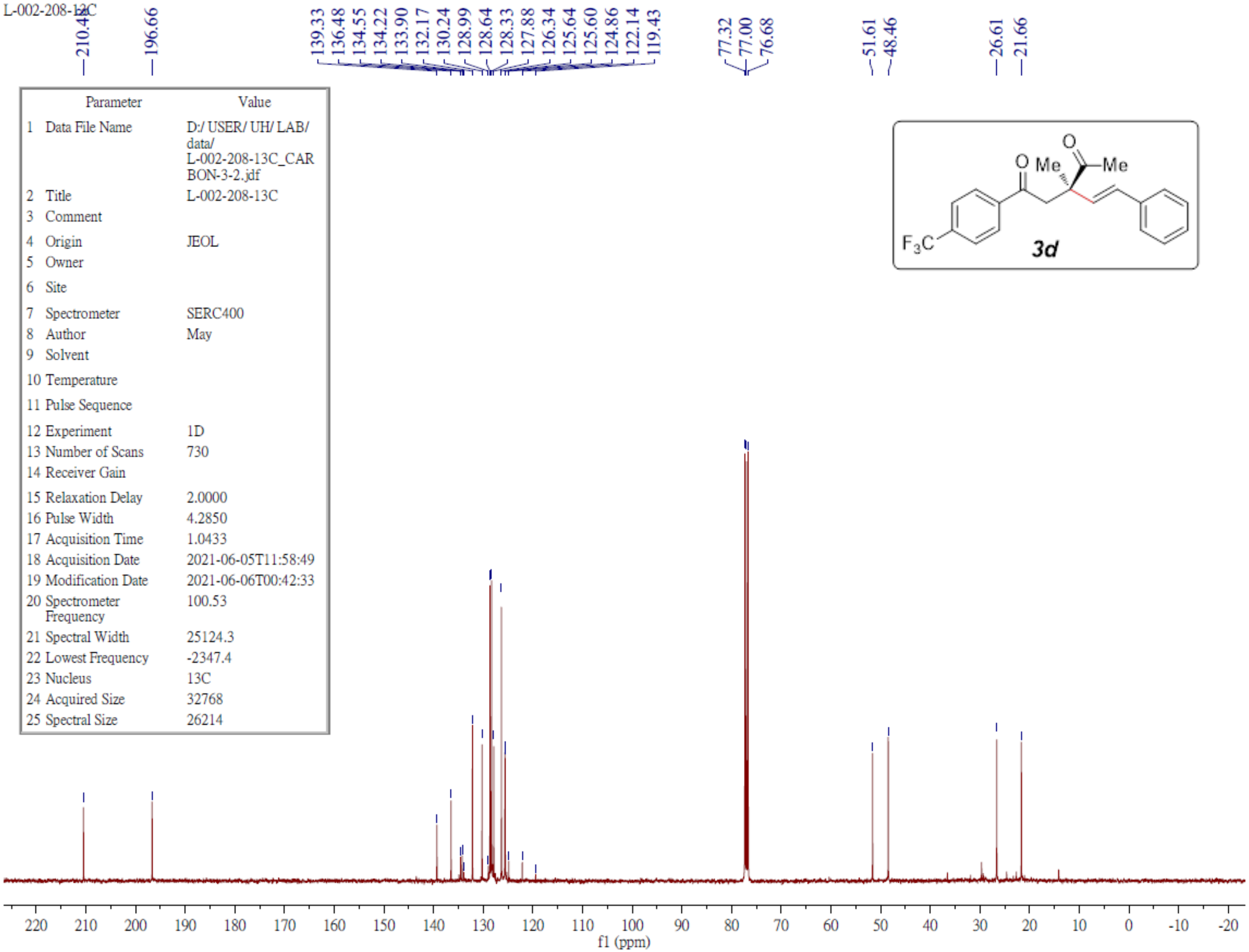

	Parameter	Value
1	Data File Name	D:/ USER/ UH/ LAB/ data/ L-002-208-13C_CARBON-3-2.jdf
2	Title	L-002-208-13C
3	Comment	
4	Origin	JEOL
5	Owner	
6	Site	
7	Spectrometer	SERC400
8	Author	May
9	Solvent	
10	Temperature	
11	Pulse Sequence	
12	Experiment	1D
13	Number of Scans	730
14	Receiver Gain	
15	Relaxation Delay	2.0000
16	Pulse Width	4.2850
17	Acquisition Time	1.0433
18	Acquisition Date	2021-06-05T11:58:49
19	Modification Date	2021-06-06T00:42:33
20	Spectrometer Frequency	100.53
21	Spectral Width	25124.3
22	Lowest Frequency	-2347.4
23	Nucleus	13C
24	Acquired Size	32768
25	Spectral Size	26214

3d

L-002-214-F

Parameter		Value
1	Data File Name	D:/ USER/ UH/ LAB/ data/ L-002-214-F_Fluorine-2-2.jdf
2	Title	L-002-214-F
3	Comment	
4	Origin	JEOL
5	Owner	
6	Site	
7	Spectrometer	SERC ECZ400S
8	Author	May
9	Solvent	
10	Temperature	
11	Pulse Sequence	
12	Experiment	1D
13	Number of Scans	5
14	Receiver Gain	
15	Relaxation Delay	4.0000
16	Pulse Width	3.1850
17	Acquisition Time	1.9862
18	Acquisition Date	2023-07-14T12:08:54
19	Modification Date	2023-07-14T12:28:37
20	Spectrometer Frequency	376.17
21	Spectral Width	75757.3
22	Lowest Frequency	-75495.8
23	Nucleus	Fluorine19
24	Acquired Size	188087
25	Spectral Size	419430

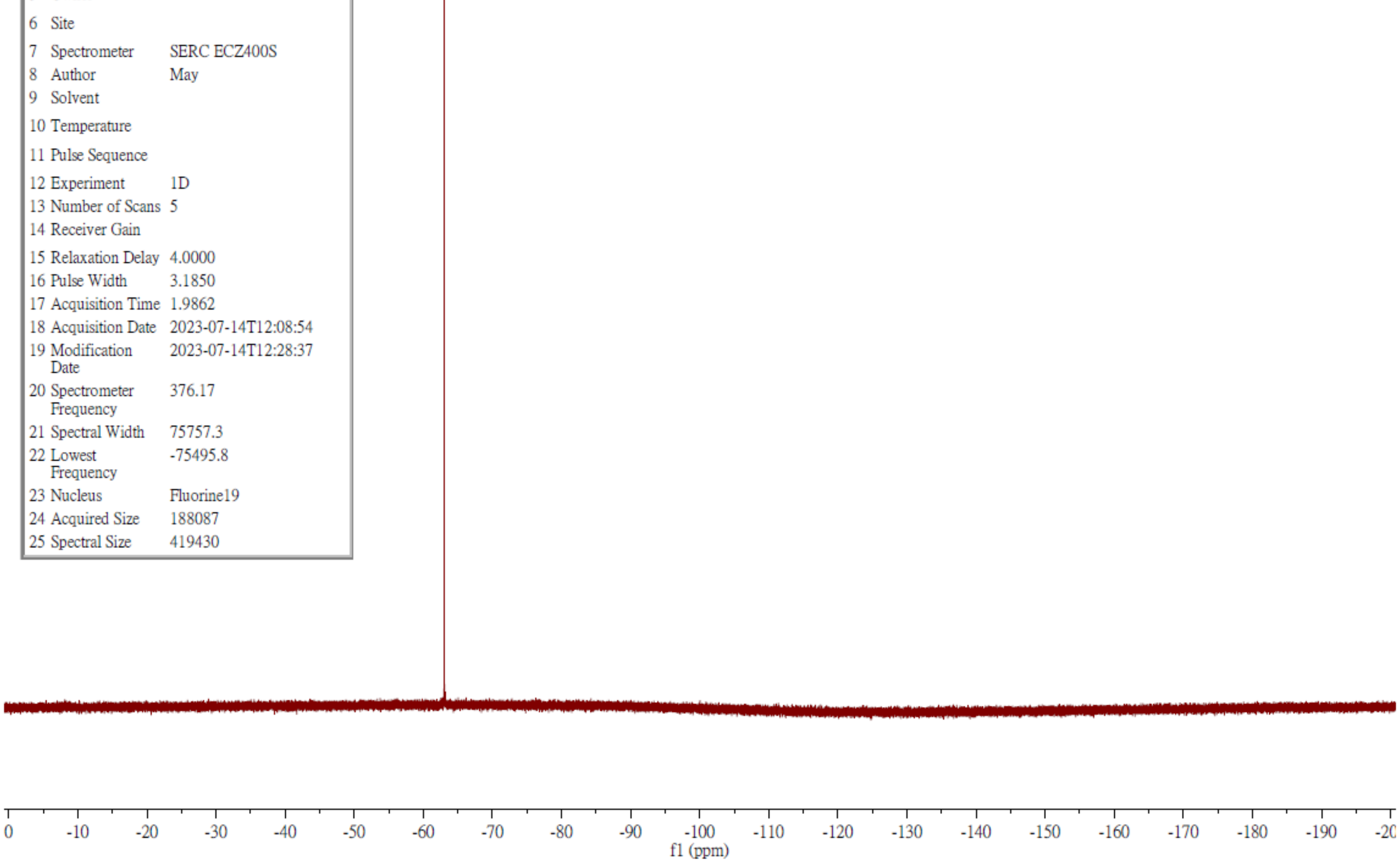

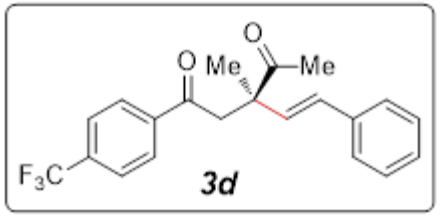

L-003-213

	Parameter	Value
1	Data File Name	D:/ USER/ UH/ LAB/ data/ L-003-213_PROTON-2-2.jdf
2	Title	L-003-213
3	Comment	
4	Origin	JEOL
5	Owner	
6	Site	
7	Spectrometer	SERC ECZ400S
8	Author	May
9	Solvent	
10	Temperature	
11	Pulse Sequence	
12	Experiment	1D
13	Number of Scans	16
14	Receiver Gain	
15	Relaxation Delay	4.0000
16	Pulse Width	3.0750
17	Acquisition Time	1.9959
18	Acquisition Date	2022-02-28T10:23:44
19	Modification Date	2022-02-28T18:58:46
20	Spectrometer Frequency	399.78
21	Spectral Width	6009.3
22	Lowest Frequency	-1001.3
23	Nucleus	Proton
24	Acquired Size	14993
25	Spectral Size	26214

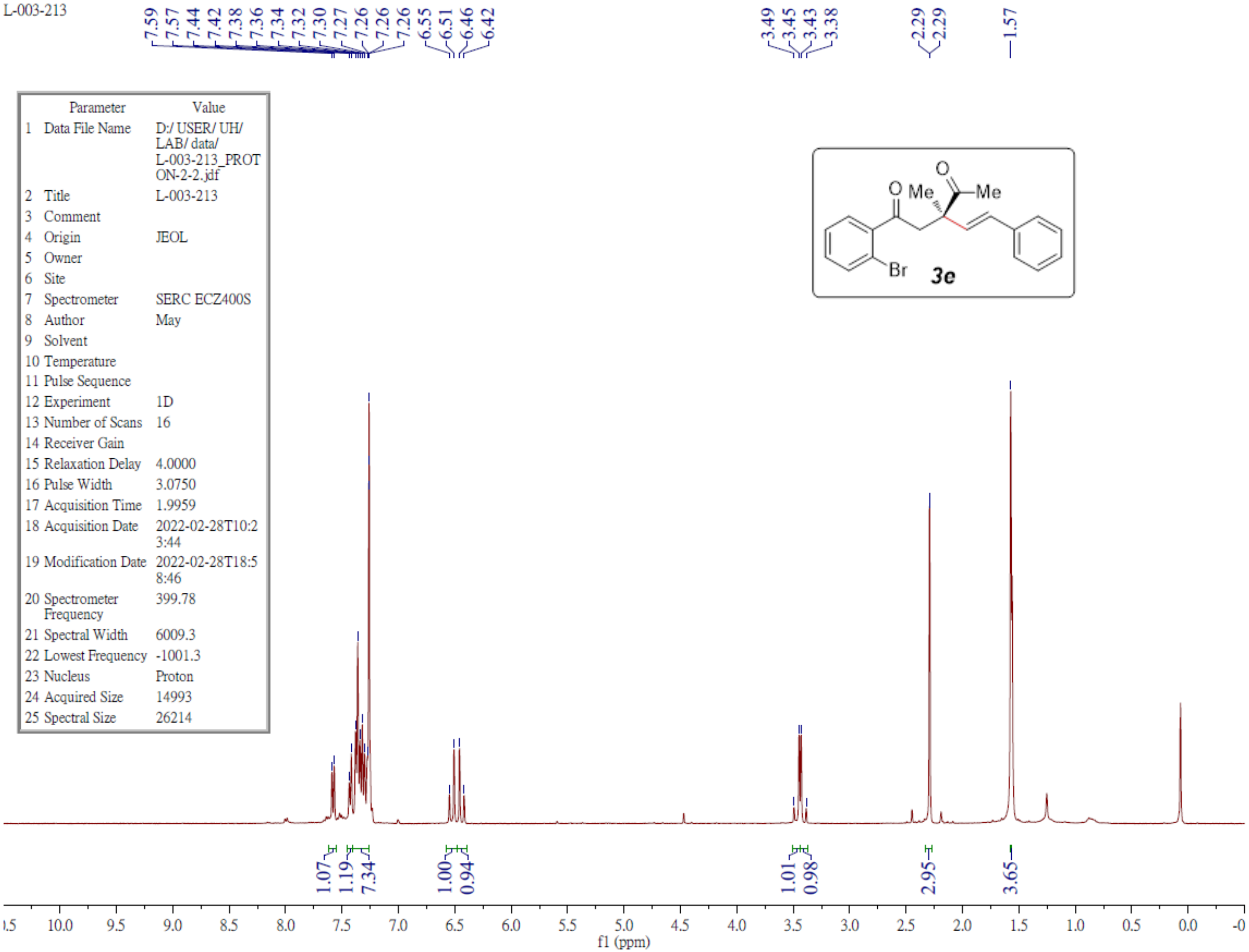

3e

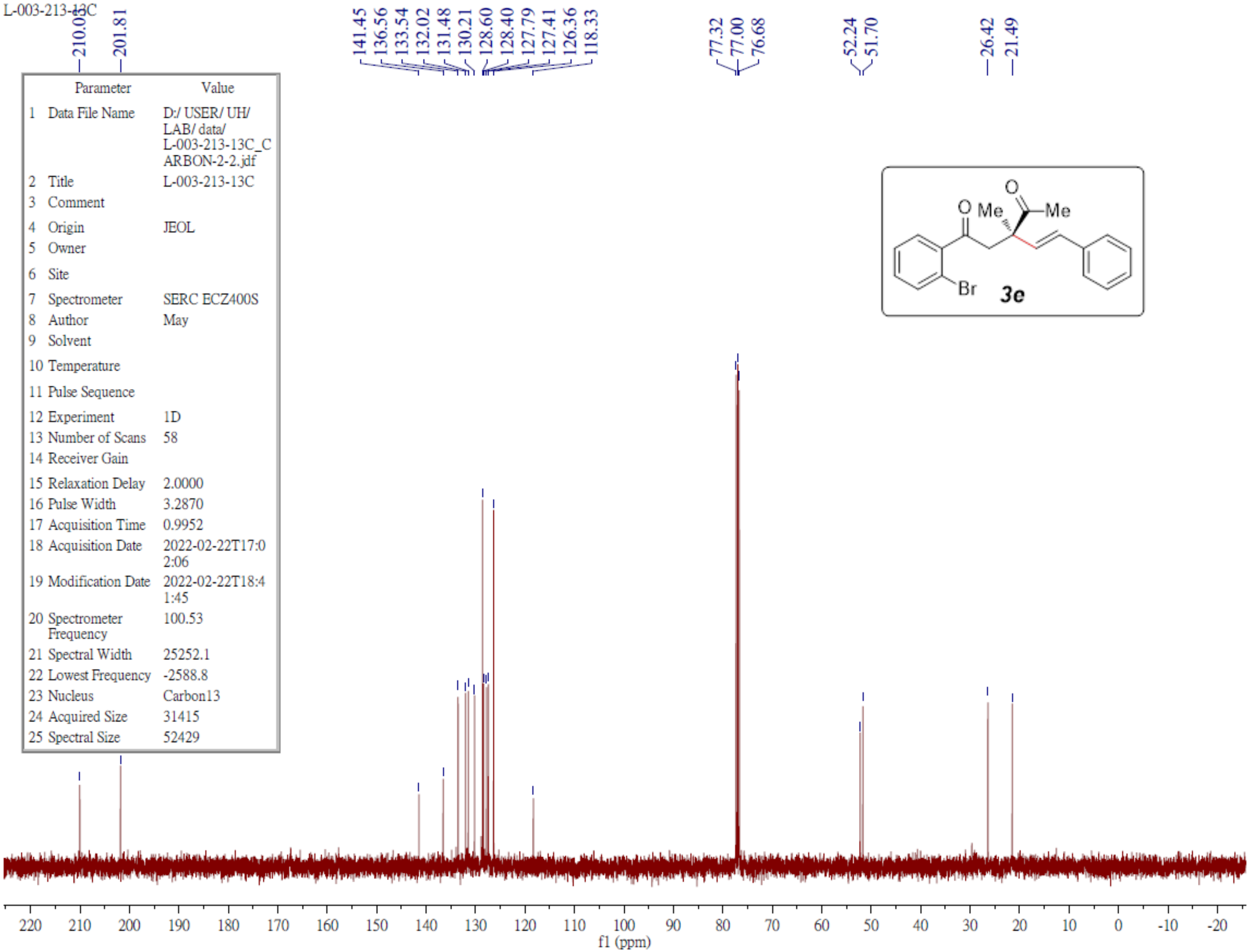

	Parameter	Value
1	Data File Name	D:/ USER/ UH/ LAB/ data/ L-003-213-13C_CARBON-2-2.jdf
2	Title	L-003-213-13C
3	Comment	
4	Origin	JEOL
5	Owner	
6	Site	
7	Spectrometer	SERC ECZ400S
8	Author	May
9	Solvent	
10	Temperature	
11	Pulse Sequence	
12	Experiment	1D
13	Number of Scans	58
14	Receiver Gain	
15	Relaxation Delay	2.0000
16	Pulse Width	3.2870
17	Acquisition Time	0.9952
18	Acquisition Date	2022-02-22T17:02:06
19	Modification Date	2022-02-22T18:41:45
20	Spectrometer Frequency	100.53
21	Spectral Width	25252.1
22	Lowest Frequency	-2588.8
23	Nucleus	Carbon13
24	Acquired Size	31415
25	Spectral Size	52429

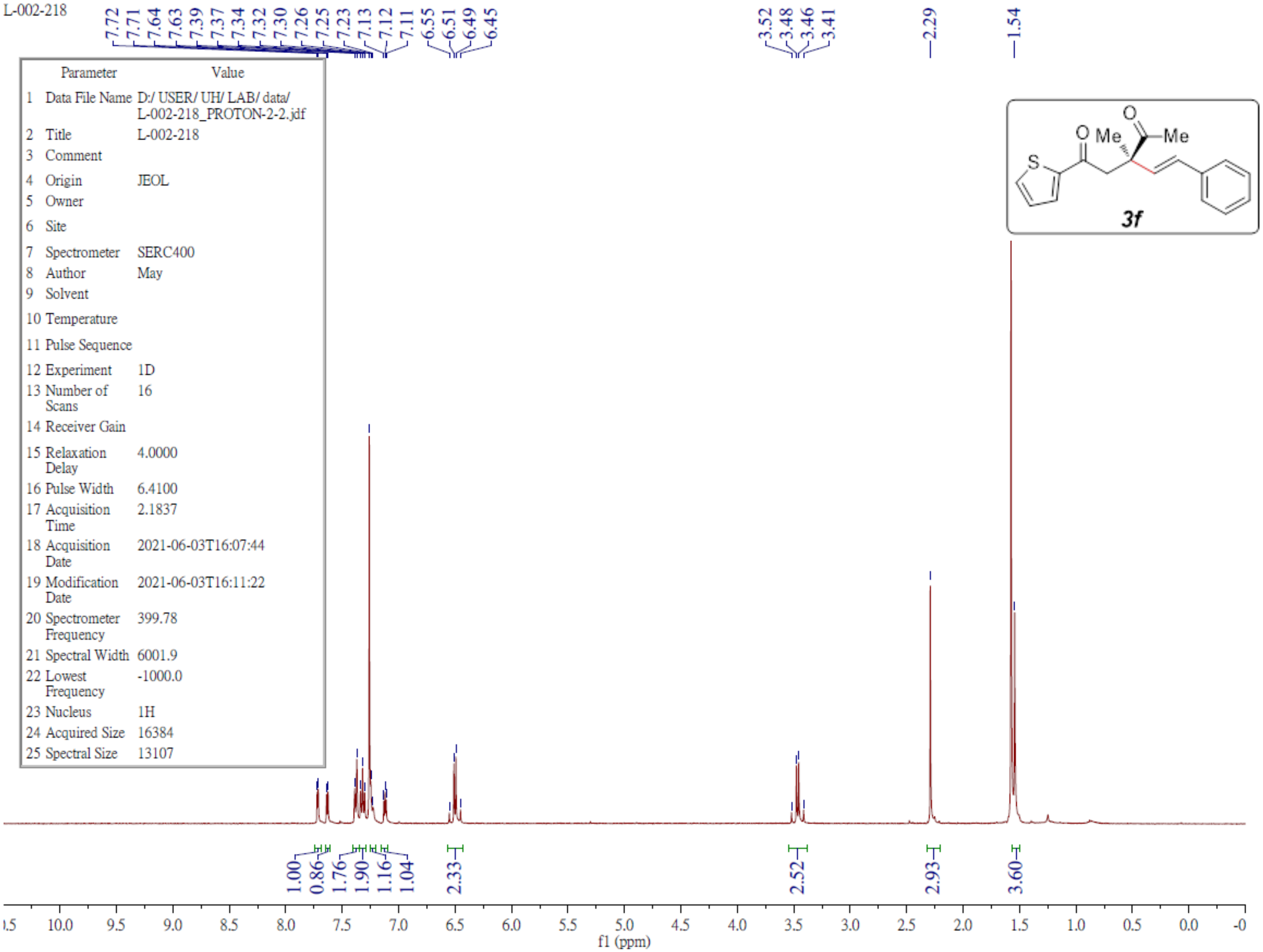

	Parameter	Value
1	Data File Name	D:/ USER/ UH/ LAB/ data/ L-002-218_PROTON-2-2.jdf
2	Title	L-002-218
3	Comment	
4	Origin	JEOL
5	Owner	
6	Site	
7	Spectrometer	SERC400
8	Author	May
9	Solvent	
10	Temperature	
11	Pulse Sequence	
12	Experiment	1D
13	Number of Scans	16
14	Receiver Gain	
15	Relaxation Delay	4.0000
16	Pulse Width	6.4100
17	Acquisition Time	2.1837
18	Acquisition Date	2021-06-03T16:07:44
19	Modification Date	2021-06-03T16:11:22
20	Spectrometer Frequency	399.78
21	Spectral Width	6001.9
22	Lowest Frequency	-1000.0
23	Nucleus	1H
24	Acquired Size	16384
25	Spectral Size	13107

3f

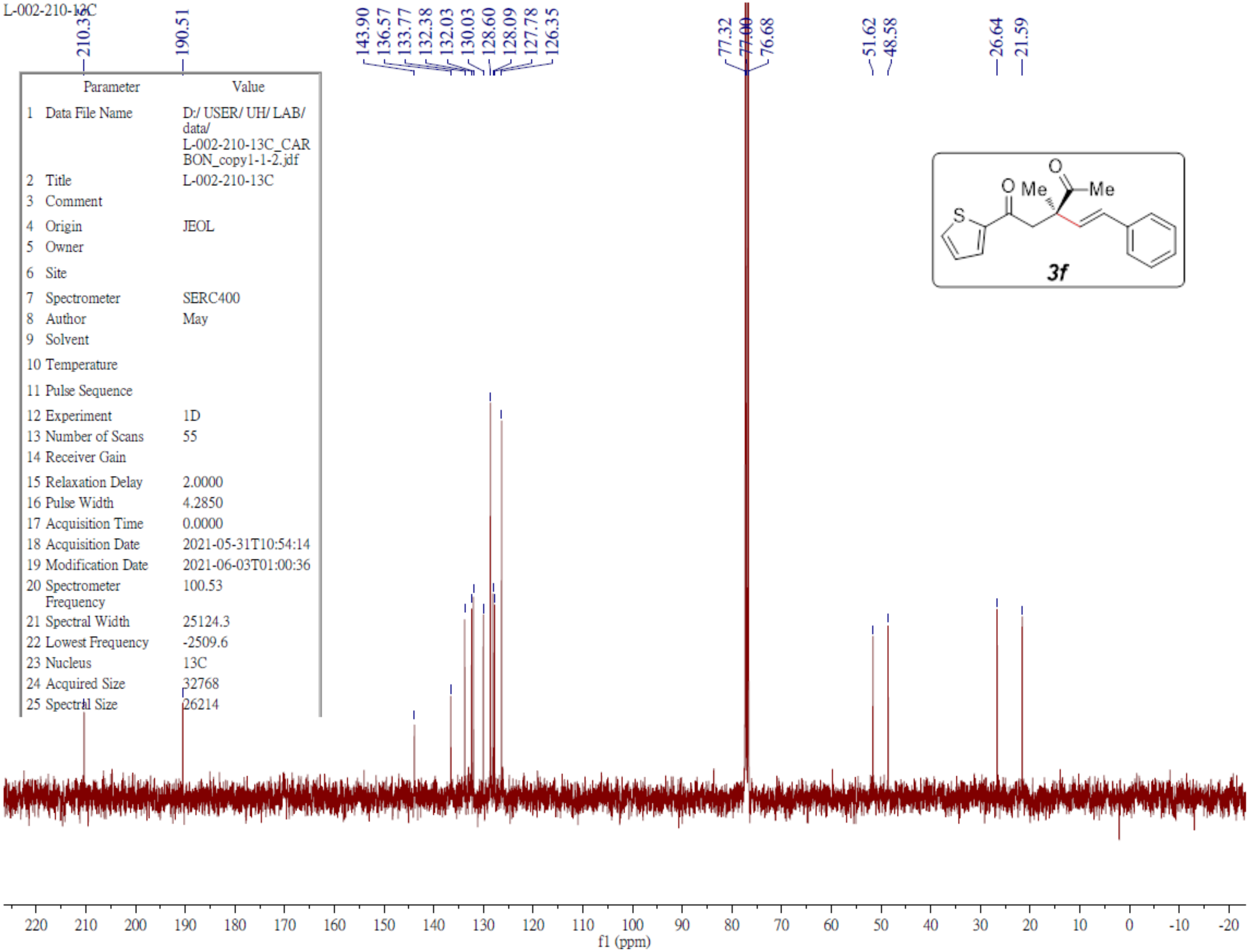
L-002-210-13C
210.35
190.51
143.90
136.57
133.77
132.38
132.03
130.03
128.60
128.09
127.78
126.35
77.32
77.00
76.68
51.62
48.58
26.64
21.59
Parameter
Value
1 Data File Name
D:/ USER/ UH/ LAB/ data/ L-002-210-13C_CARBON_copy1-1-2.jdf
2 Title
L-002-210-13C
3 Comment
4 Origin
JEOL
5 Owner
6 Site
7 Spectrometer
SERC400
8 Author
May
9 Solvent
10 Temperature
11 Pulse Sequence
12 Experiment
1D
13 Number of Scans
55
14 Receiver Gain
15 Relaxation Delay
2.0000
16 Pulse Width
4.2850
17 Acquisition Time
0.0000
18 Acquisition Date
2021-05-31T10:54:14
19 Modification Date
2021-06-03T01:00:36
20 Spectrometer Frequency
100.53
21 Spectral Width
25124.3
22 Lowest Frequency
-2509.6
23 Nucleus
13C
24 Acquired Size
32768
25 Spectral Size
26214
S
O
O
Me
Me
3f
220 210 200 190 180 170 160 150 140 130 120 110 100 90 80 70 60 50 40 30 20 10 0 -10 -20
f1 (ppm)

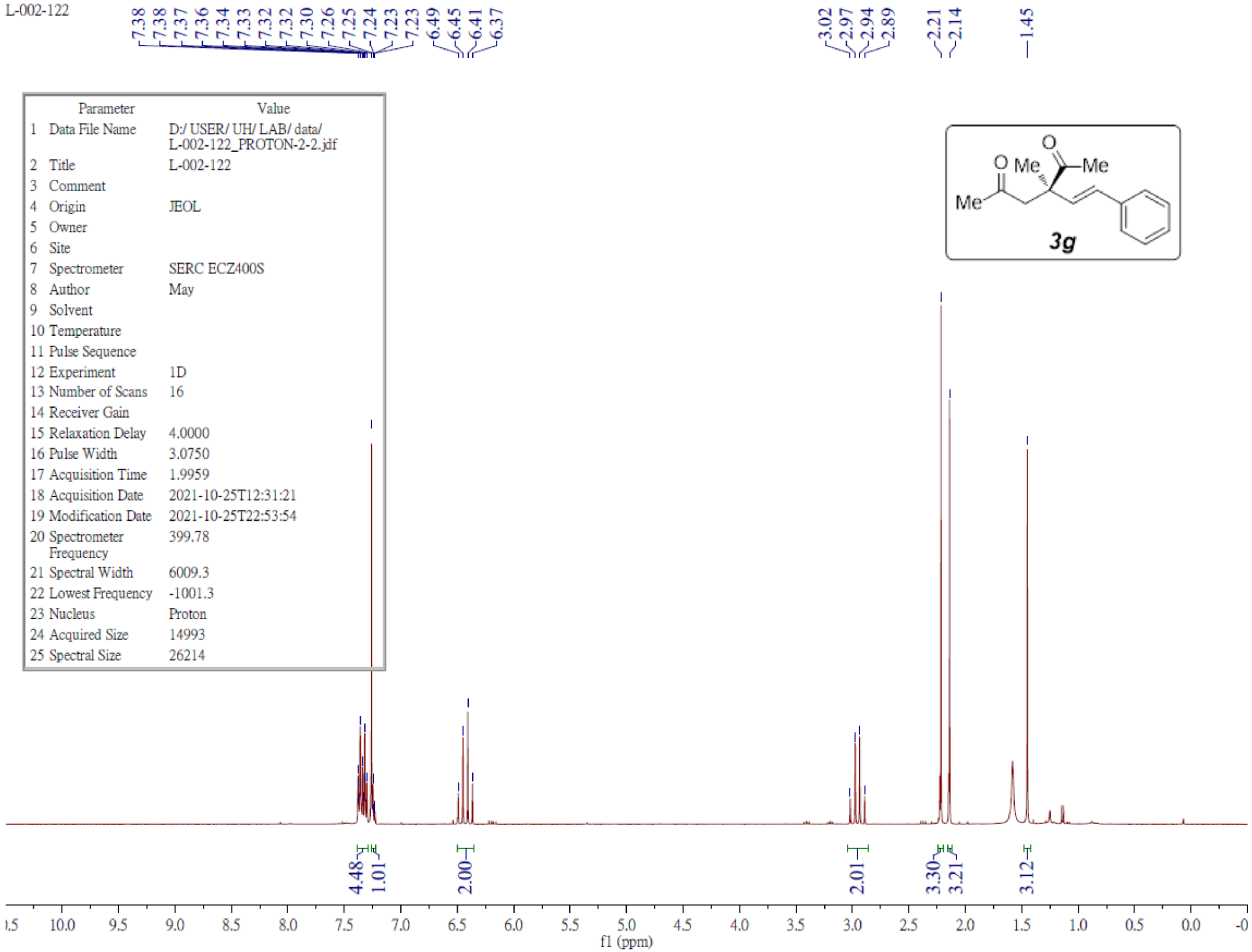

Parameter	Value
1 Data File Name	D:/ USER/ UH/ LAB/ data/ L-002-122_PROTON-2-2.jdf
2 Title	L-002-122
3 Comment	
4 Origin	JEOL
5 Owner	
6 Site	
7 Spectrometer	SERC ECZ400S
8 Author	May
9 Solvent	
10 Temperature	
11 Pulse Sequence	
12 Experiment	1D
13 Number of Scans	16
14 Receiver Gain	
15 Relaxation Delay	4.0000
16 Pulse Width	3.0750
17 Acquisition Time	1.9959
18 Acquisition Date	2021-10-25T12:31:21
19 Modification Date	2021-10-25T22:53:54
20 Spectrometer Frequency	399.78
21 Spectral Width	6009.3
22 Lowest Frequency	-1001.3
23 Nucleus	Proton
24 Acquired Size	14993
25 Spectral Size	26214

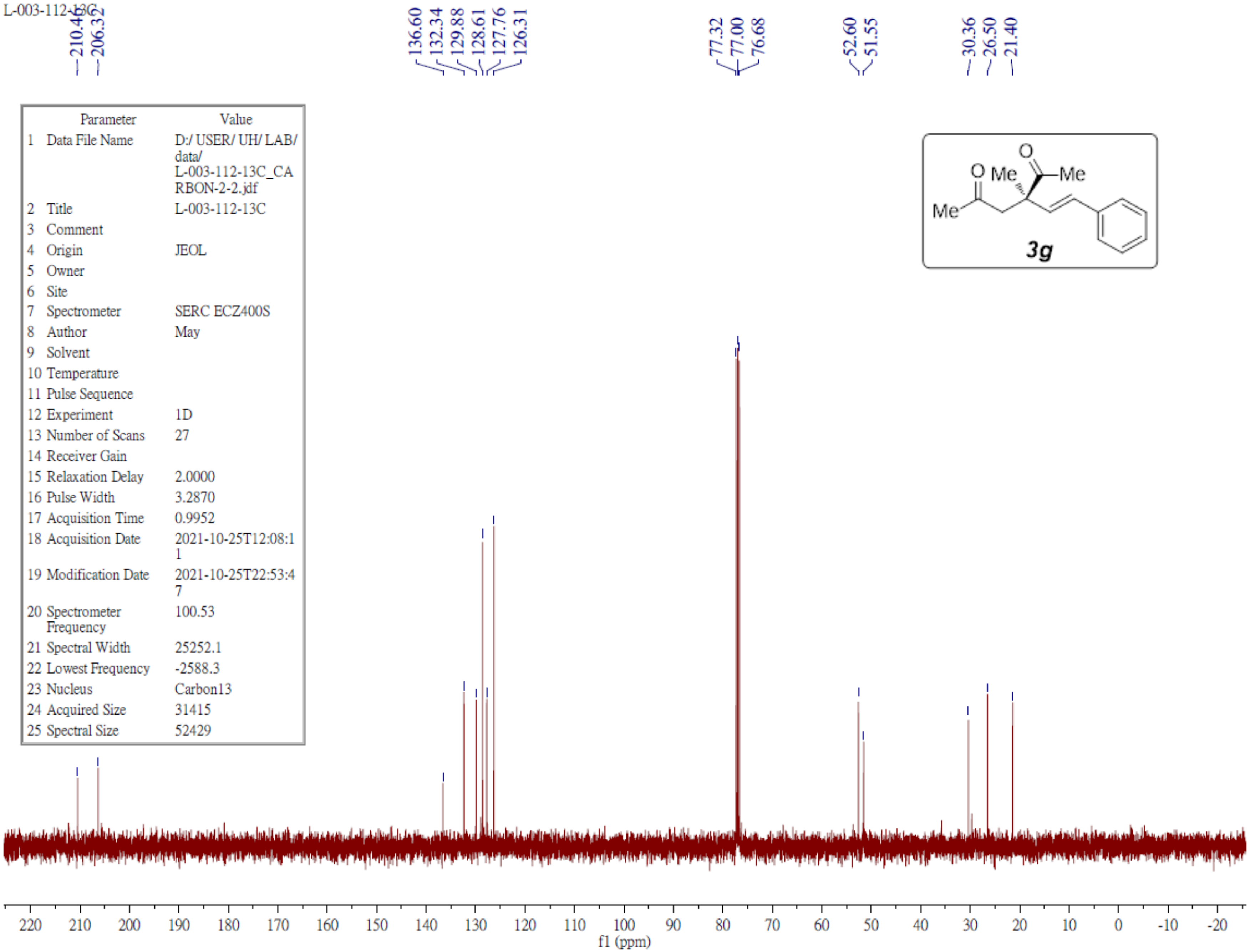

	Parameter	Value
1	Data File Name	D:/ USER/ UH/ LAB/ data/ L-003-112-13C_CARBON-2-2.jdf
2	Title	L-003-112-13C
3	Comment	
4	Origin	JEOL
5	Owner	
6	Site	
7	Spectrometer	SERC ECZ400S
8	Author	May
9	Solvent	
10	Temperature	
11	Pulse Sequence	
12	Experiment	1D
13	Number of Scans	27
14	Receiver Gain	
15	Relaxation Delay	2.0000
16	Pulse Width	3.2870
17	Acquisition Time	0.9952
18	Acquisition Date	2021-10-25T12:08:11
19	Modification Date	2021-10-25T22:53:47
20	Spectrometer Frequency	100.53
21	Spectral Width	25252.1
22	Lowest Frequency	-2588.3
23	Nucleus	Carbon13
24	Acquired Size	31415
25	Spectral Size	52429

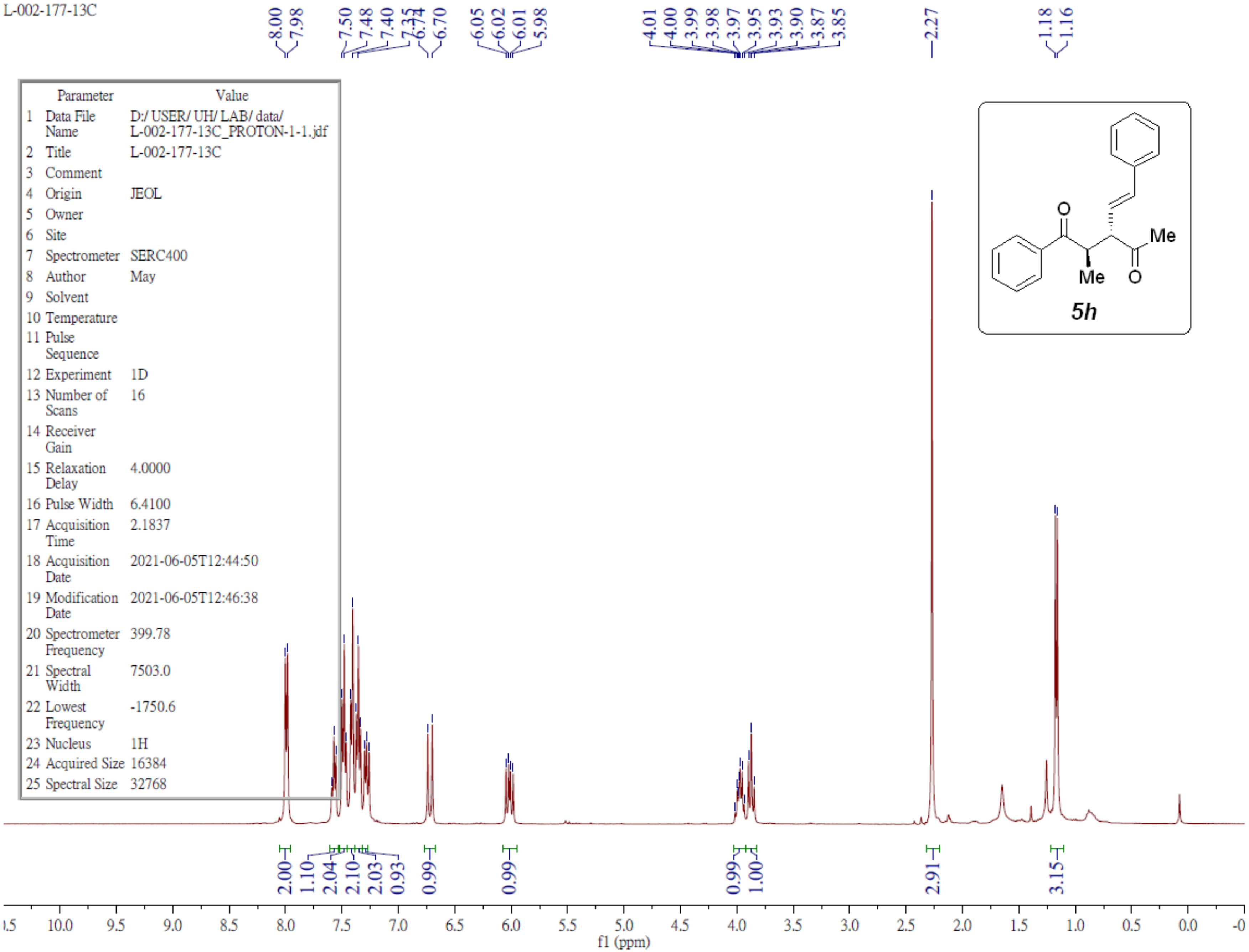
L-002-177-13C
Parameter Value
1 Data File Name D:/ USER/ UH/ LAB/ data/ L-002-177-13C_PROTON-1-1.jdf
2 Title L-002-177-13C
3 Comment
4 Origin JEOL
5 Owner
6 Site
7 Spectrometer SERC400
8 Author May
9 Solvent
10 Temperature
11 Pulse Sequence
12 Experiment 1D
13 Number of Scans 16
14 Receiver Gain
15 Relaxation Delay 4.0000
16 Pulse Width 6.4100
17 Acquisition Time 2.1837
18 Acquisition Date 2021-06-05T12:44:50
19 Modification Date 2021-06-05T12:46:38
20 Spectrometer Frequency 399.78
21 Spectral Width 7503.0
22 Lowest Frequency -1750.6
23 Nucleus 1H
24 Acquired Size 16384
25 Spectral Size 32768
8.00 7.98 7.50 7.48 7.40 7.35 6.74 6.70 6.05 6.02 6.01 5.98 4.01 4.00 3.99 3.98 3.97 3.95 3.93 3.90 3.87 3.85 2.27 1.18 1.16
O
Me
Me
O
5h
2.00 1.10 2.04 2.10 2.03 0.93 0.99 0.99 0.99 1.00 2.91 3.15
f1 (ppm)

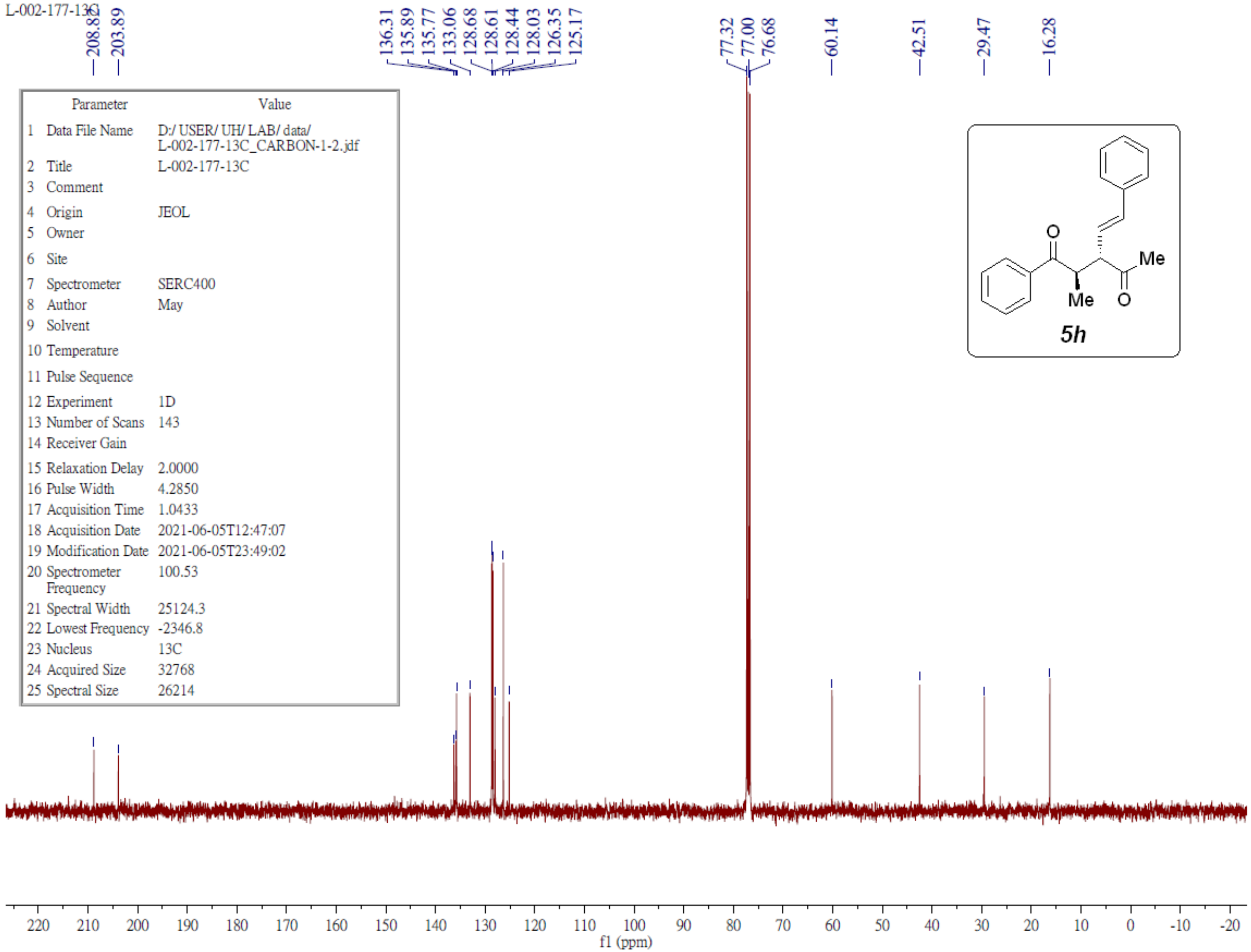
L-002-177-13C
208.82
203.89
136.31
135.89
135.77
133.06
128.68
128.61
128.44
128.03
126.35
125.17
77.32
77.00
76.68
60.14
42.51
29.47
16.28
Parameter
Value
1 Data File Name
D:/ USER/ UH/ LAB/ data/ L-002-177-13C_CARBON-1-2.jdf
2 Title
L-002-177-13C
3 Comment
4 Origin
JEOL
5 Owner
6 Site
7 Spectrometer
SERC400
8 Author
May
9 Solvent
10 Temperature
11 Pulse Sequence
12 Experiment
1D
13 Number of Scans
143
14 Receiver Gain
15 Relaxation Delay
2.0000
16 Pulse Width
4.2850
17 Acquisition Time
1.0433
18 Acquisition Date
2021-06-05T12:47:07
19 Modification Date
2021-06-05T23:49:02
20 Spectrometer Frequency
100.53
21 Spectral Width
25124.3
22 Lowest Frequency
-2346.8
23 Nucleus
13C
24 Acquired Size
32768
25 Spectral Size
26214
O
Me
Me
O
5h
220
210
200
190
180
170
160
150
140
130
120
110
100
90
80
70
60
50
40
30
20
10
0
-10
-20
f1 (ppm)

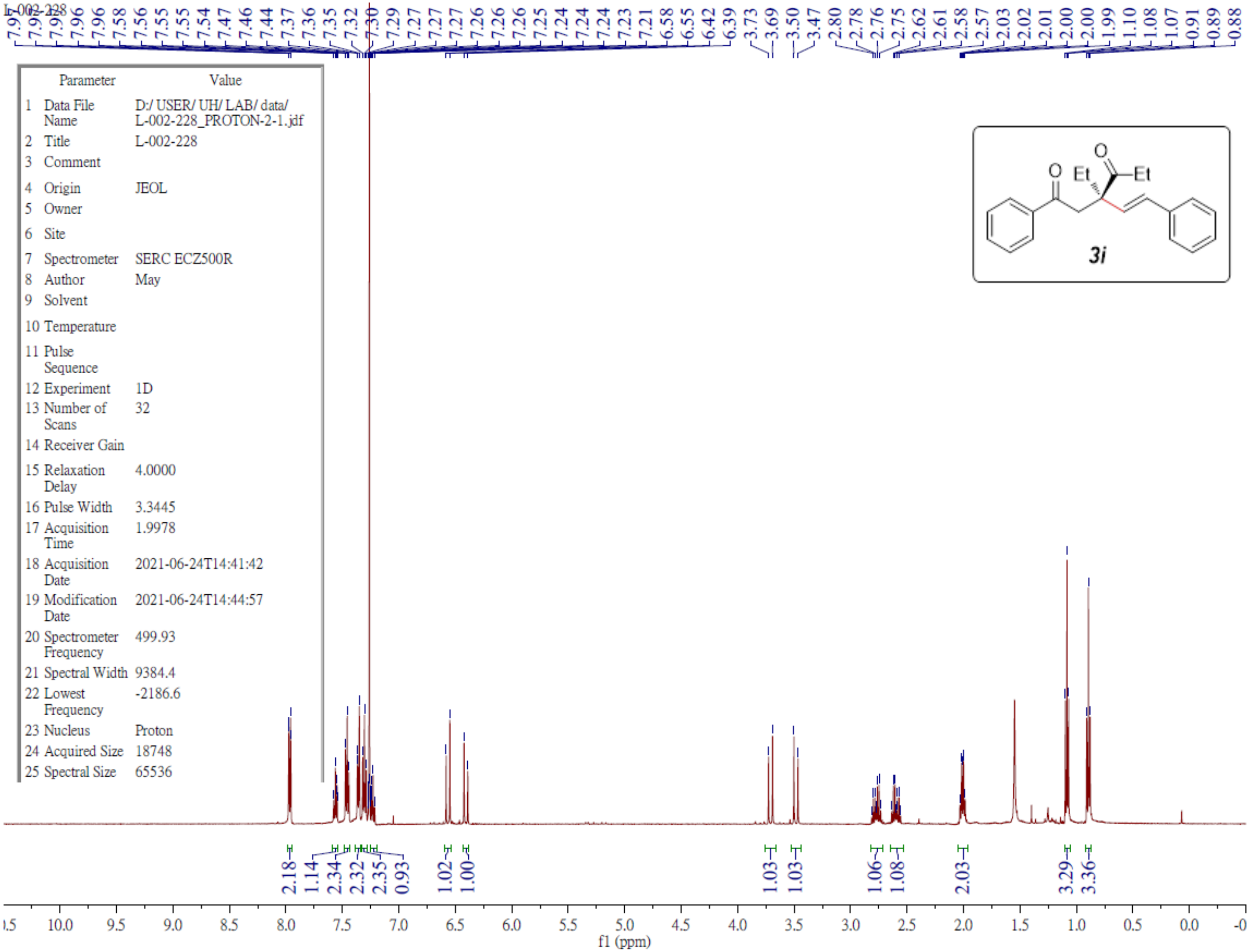
L-002-228
Parameter Value
1 Data File Name D:/ USER/ UH/ LAB/ data/ L-002-228_PROTON-2-1.jdf
2 Title L-002-228
3 Comment
4 Origin JEOL
5 Owner
6 Site
7 Spectrometer SERC ECZ500R
8 Author May
9 Solvent
10 Temperature
11 Pulse Sequence
12 Experiment 1D
13 Number of Scans 32
14 Receiver Gain
15 Relaxation Delay 4.0000
16 Pulse Width 3.3445
17 Acquisition Time 1.9978
18 Acquisition Date 2021-06-24T14:41:42
19 Modification Date 2021-06-24T14:44:57
20 Spectrometer Frequency 499.93
21 Spectral Width 9384.4
22 Lowest Frequency -2186.6
23 Nucleus Proton
24 Acquired Size 18748
25 Spectral Size 65536
O
O
Et
Et
3i
f1 (ppm)

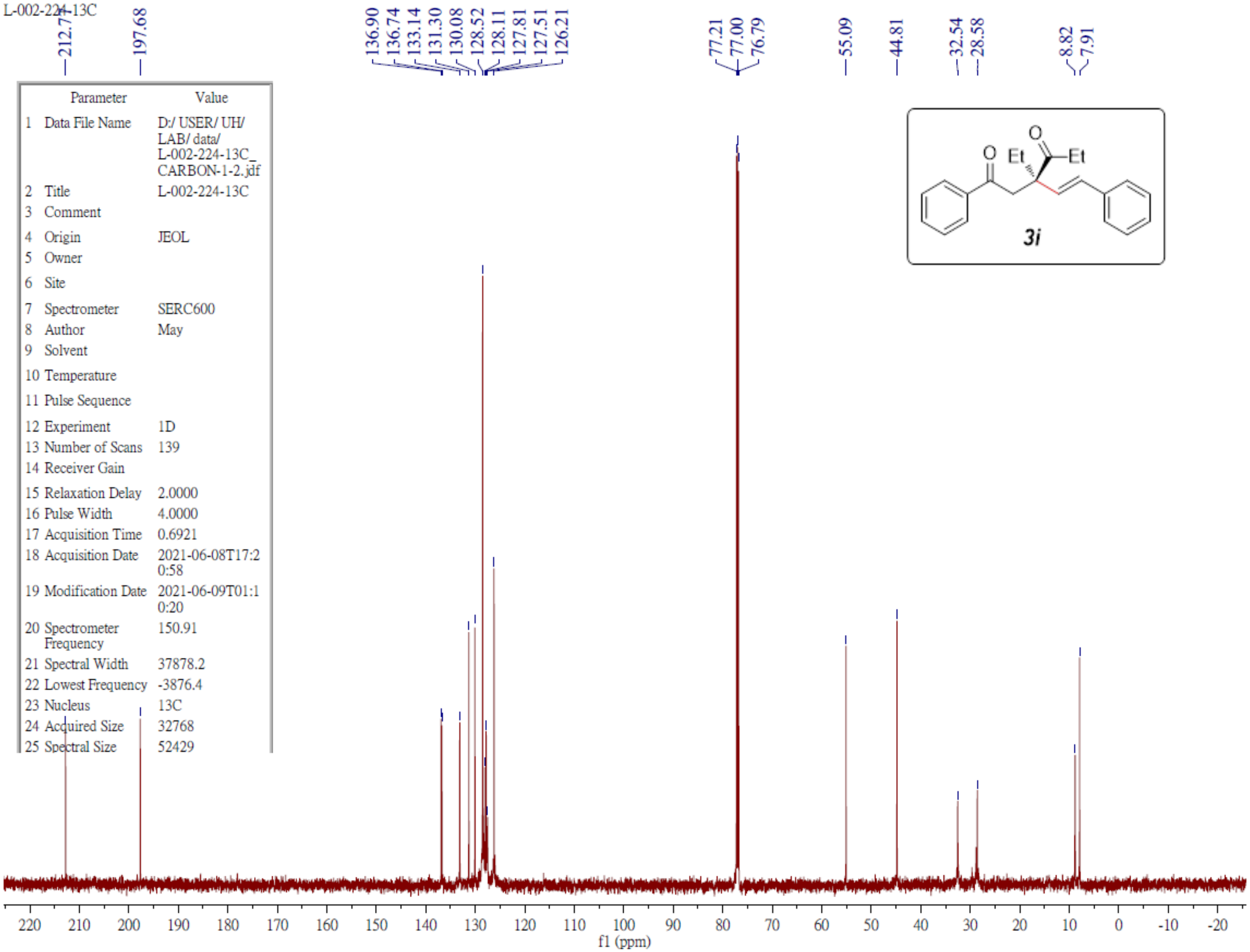

L-002-224-13C
212.77
197.68
136.90
136.74
133.14
131.30
130.08
128.52
128.11
127.81
127.51
126.21
77.21
77.00
76.79
55.09
44.81
32.54
28.58
8.82
7.91
Parameter Value
1 Data File Name D:/ USER/ UH/ LAB/ data/ L-002-224-13C_ CARBON-1-2.jdf
2 Title L-002-224-13C
3 Comment
4 Origin JEOL
5 Owner
6 Site
7 Spectrometer SERC600
8 Author May
9 Solvent
10 Temperature
11 Pulse Sequence
12 Experiment 1D
13 Number of Scans 139
14 Receiver Gain
15 Relaxation Delay 2.0000
16 Pulse Width 4.0000
17 Acquisition Time 0.6921
18 Acquisition Date 2021-06-08T17:20:58
19 Modification Date 2021-06-09T01:10:20
20 Spectrometer Frequency 150.91
21 Spectral Width 37878.2
22 Lowest Frequency -3876.4
23 Nucleus 13C
24 Acquired Size 32768
25 Spectral Size 52429
O
Et
O
Et
3i
220 210 200 190 180 170 160 150 140 130 120 110 100 90 80 70 60 50 40 30 20 10 0 -10 -20
f1 (ppm)

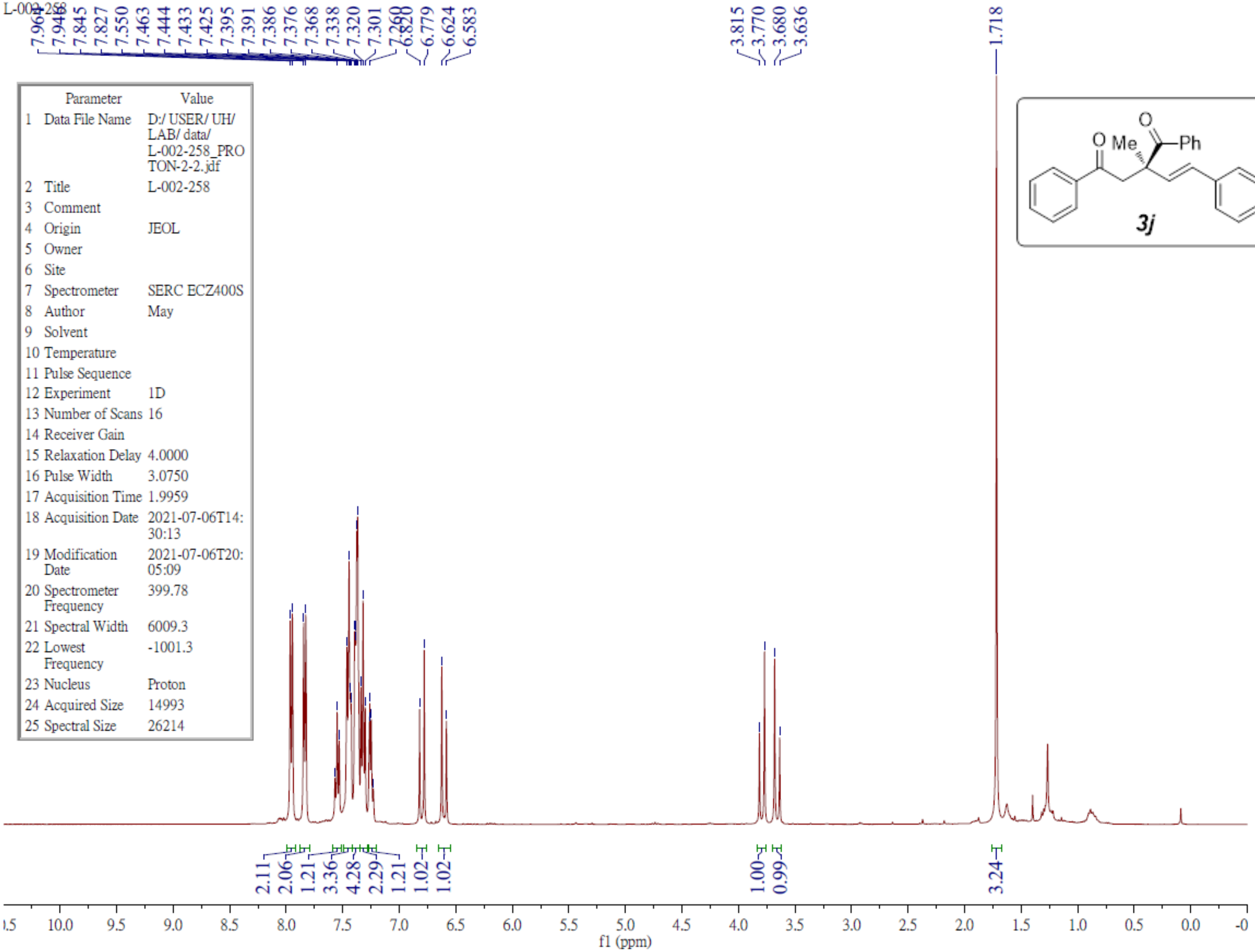

	Parameter	Value
1	Data File Name	D:/ USER/ UH/ LAB/ data/ L-002-258_PROTON-2-2.jdf
2	Title	L-002-258
3	Comment	
4	Origin	JEOL
5	Owner	
6	Site	
7	Spectrometer	SERC ECZ400S
8	Author	May
9	Solvent	
10	Temperature	
11	Pulse Sequence	
12	Experiment	1D
13	Number of Scans	16
14	Receiver Gain	
15	Relaxation Delay	4.0000
16	Pulse Width	3.0750
17	Acquisition Time	1.9959
18	Acquisition Date	2021-07-06T14:30:13
19	Modification Date	2021-07-06T20:05:09
20	Spectrometer Frequency	399.78
21	Spectral Width	6009.3
22	Lowest Frequency	-1001.3
23	Nucleus	Proton
24	Acquired Size	14993
25	Spectral Size	26214

3j

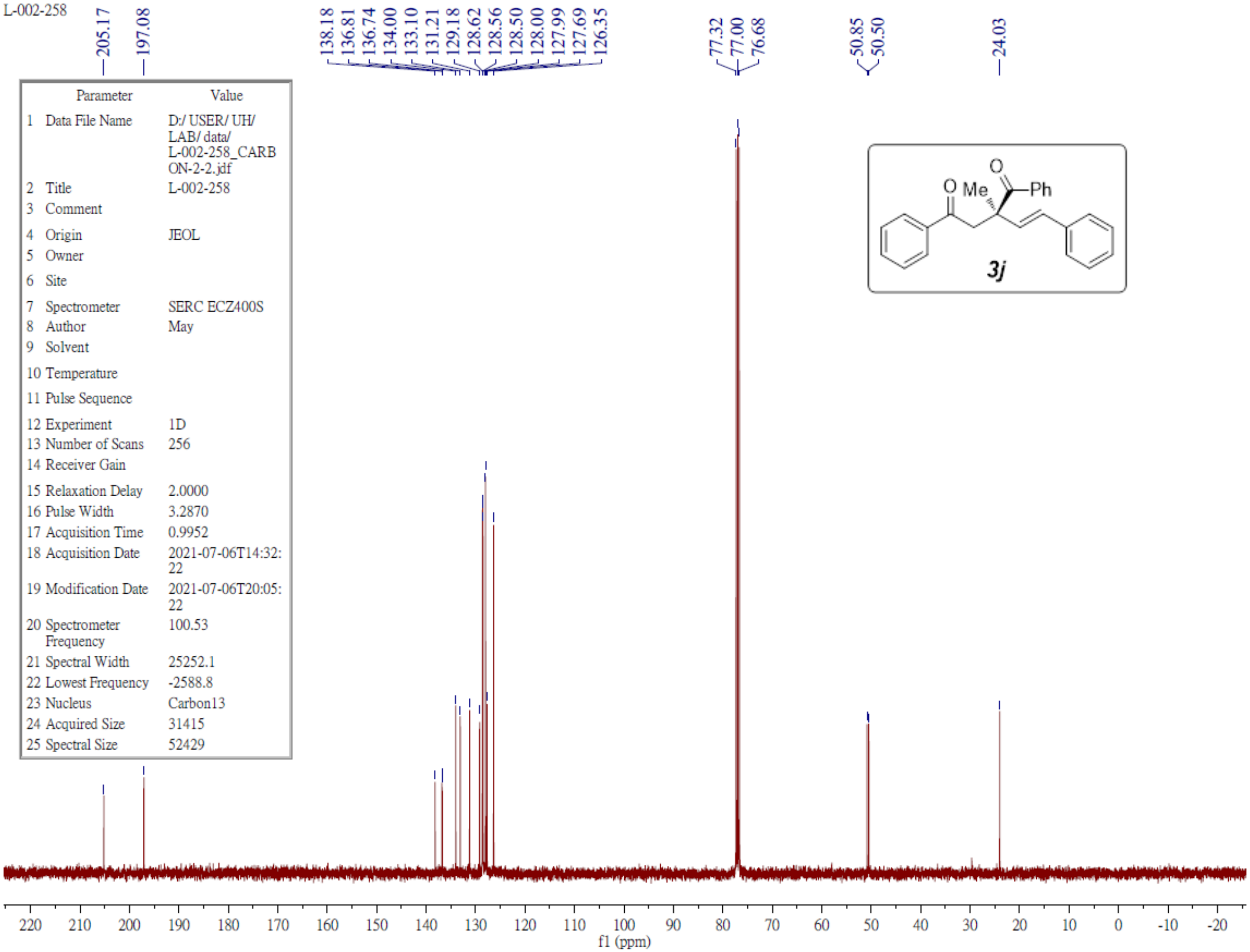

L-002-258
205.17
197.08
138.18
136.81
136.74
134.00
133.10
131.21
129.18
128.62
128.56
128.50
128.00
127.99
127.69
126.35
77.32
77.00
76.68
50.85
50.50
24.03
Parameter
Value
1 Data File Name
D:/ USER/ UH/ LAB/ data/ L-002-258_CARBON-2-2.jdf
2 Title
L-002-258
3 Comment
4 Origin
JEOL
5 Owner
6 Site
7 Spectrometer
SERC ECZ400S
8 Author
May
9 Solvent
10 Temperature
11 Pulse Sequence
12 Experiment
1D
13 Number of Scans
256
14 Receiver Gain
15 Relaxation Delay
2.0000
16 Pulse Width
3.2870
17 Acquisition Time
0.9952
18 Acquisition Date
2021-07-06T14:32:22
19 Modification Date
2021-07-06T20:05:22
20 Spectrometer Frequency
100.53
21 Spectral Width
25252.1
22 Lowest Frequency
-2588.8
23 Nucleus
Carbon13
24 Acquired Size
31415
25 Spectral Size
52429
O
Me
O
Ph
3j
220
210
200
190
180
170
160
150
140
130
120
110
100
90
80
70
60
50
40
30
20
10
0
-10
-20
f1 (ppm)

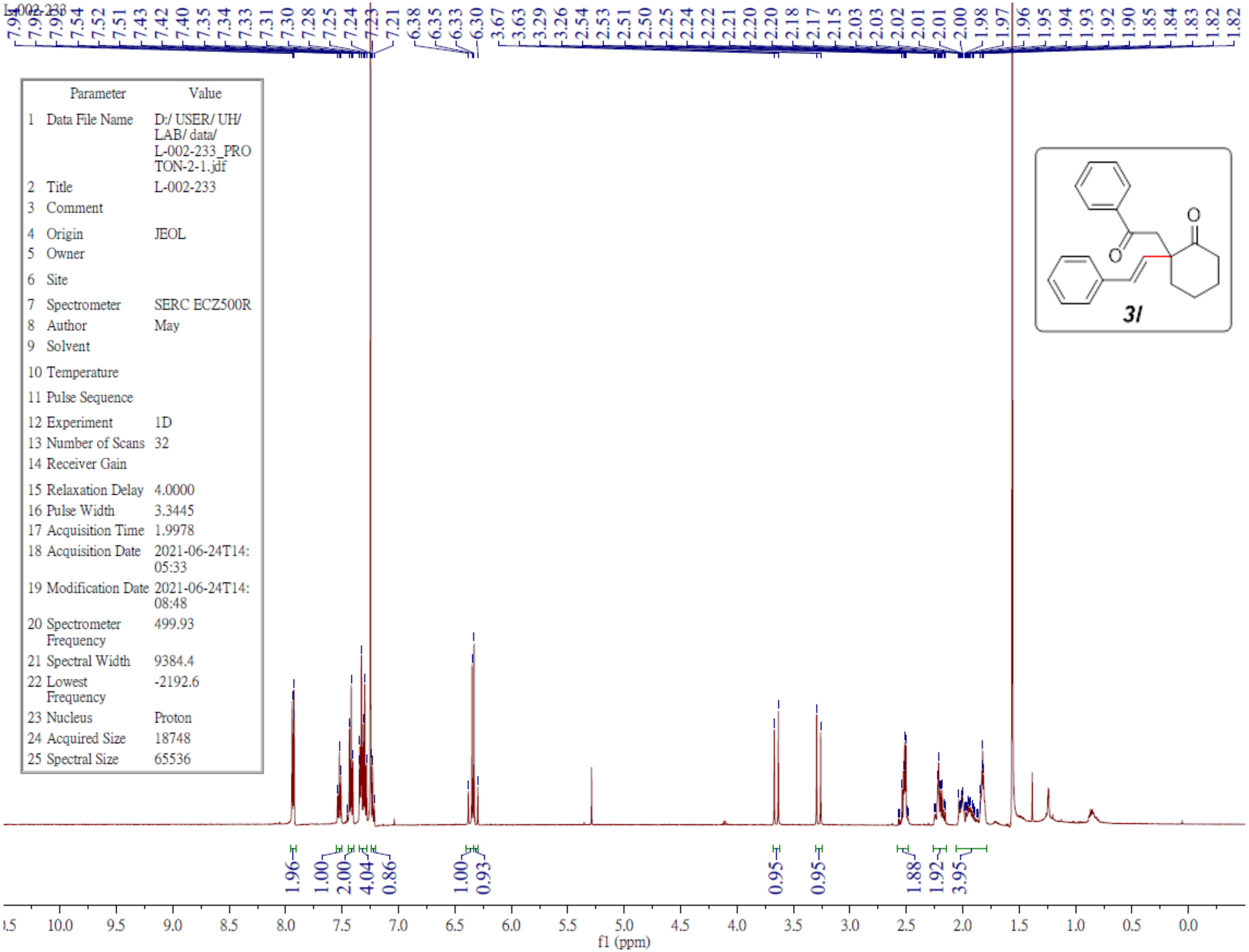
L-002-233
Parameter Value
1 Data File Name D:/ USER/ UH/ LAB/ data/ L-002-233_PROTON-2-1.jdf
2 Title L-002-233
3 Comment
4 Origin JEOL
5 Owner
6 Site
7 Spectrometer SERC ECZ500R
8 Author May
9 Solvent
10 Temperature
11 Pulse Sequence
12 Experiment 1D
13 Number of Scans 32
14 Receiver Gain
15 Relaxation Delay 4.0000
16 Pulse Width 3.3445
17 Acquisition Time 1.9978
18 Acquisition Date 2021-06-24T14:05:33
19 Modification Date 2021-06-24T14:08:48
20 Spectrometer Frequency 499.93
21 Spectral Width 9384.4
22 Lowest Frequency -2192.6
23 Nucleus Proton
24 Acquired Size 18748
25 Spectral Size 65536
3l
f1 (ppm)

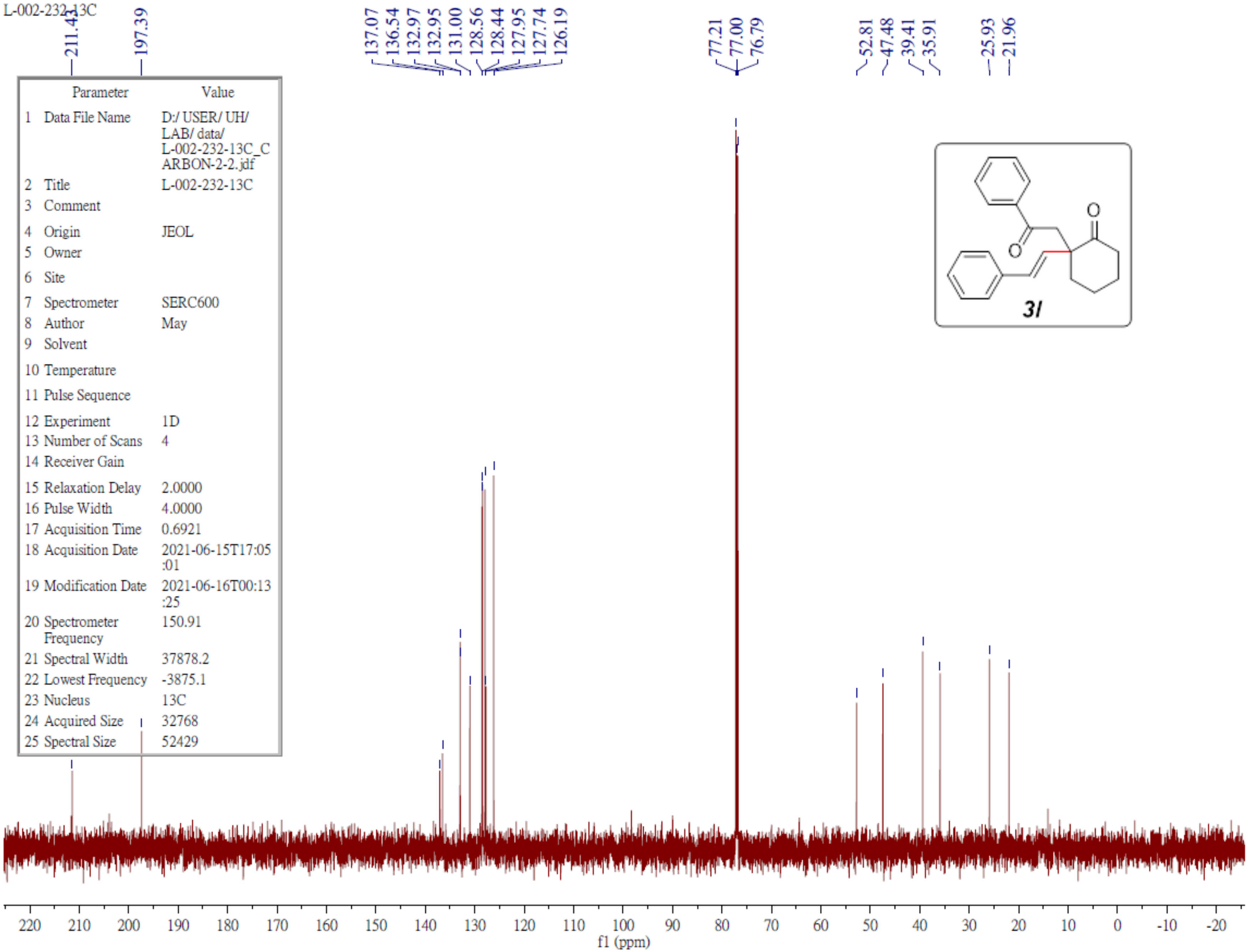
L-002-232-13C
211.41
197.39
137.07
136.54
132.97
132.95
131.00
128.56
128.44
127.95
127.74
126.19
77.21
77.00
76.79
52.81
47.48
39.41
35.91
25.93
21.96
Parameter
Value
1 Data File Name D:/ USER/ UH/ LAB/ data/ L-002-232-13C_CARBON-2-2.jdf
2 Title L-002-232-13C
3 Comment
4 Origin JEOL
5 Owner
6 Site
7 Spectrometer SERC600
8 Author May
9 Solvent
10 Temperature
11 Pulse Sequence
12 Experiment 1D
13 Number of Scans 4
14 Receiver Gain
15 Relaxation Delay 2.0000
16 Pulse Width 4.0000
17 Acquisition Time 0.6921
18 Acquisition Date 2021-06-15T17:05:01
19 Modification Date 2021-06-16T00:13:25
20 Spectrometer Frequency 150.91
21 Spectral Width 37878.2
22 Lowest Frequency -3875.1
23 Nucleus 13C
24 Acquired Size 32768
25 Spectral Size 52429
O
O
3l
220 210 200 190 180 170 160 150 140 130 120 110 100 90 80 70 60 50 40 30 20 10 0 -10 -20
f1 (ppm)

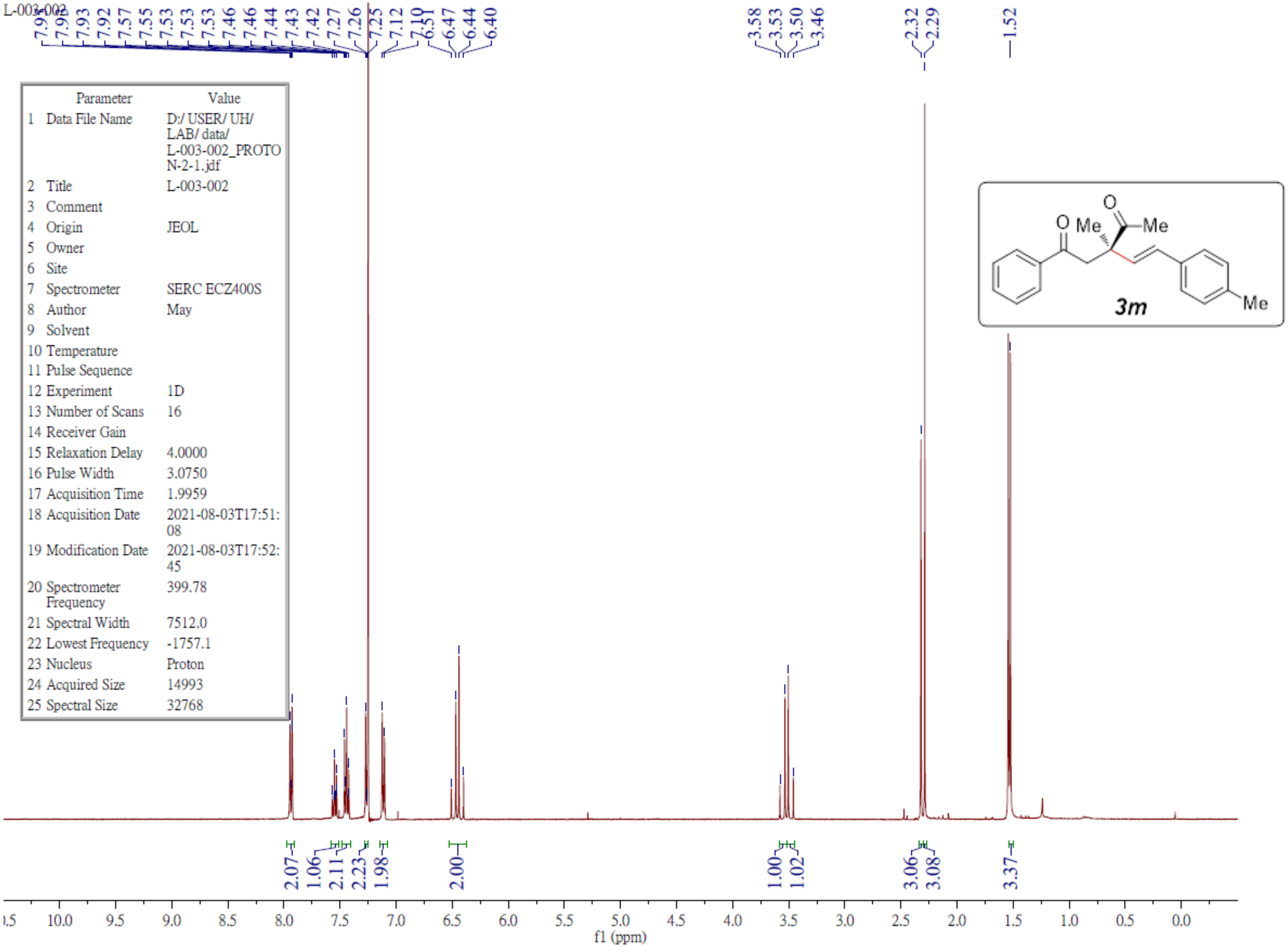
L-003-002
7.95 7.93 7.93 7.92 7.57 7.55 7.53 7.53 7.53 7.46 7.46 7.44 7.43 7.42 7.27 7.26 7.25 7.12 7.10 6.51 6.47 6.44 6.40
3.58 3.53 3.50 3.46
2.32 2.29
1.52
Parameter Value
1 Data File Name D:/ USER/ UH/ LAB/ data/ L-003-002_PROTON-2-1.jdf
2 Title L-003-002
3 Comment
4 Origin JEOL
5 Owner
6 Site
7 Spectrometer SERC ECZ400S
8 Author May
9 Solvent
10 Temperature
11 Pulse Sequence
12 Experiment 1D
13 Number of Scans 16
14 Receiver Gain
15 Relaxation Delay 4.0000
16 Pulse Width 3.0750
17 Acquisition Time 1.9959
18 Acquisition Date 2021-08-03T17:51:08
19 Modification Date 2021-08-03T17:52:45
20 Spectrometer Frequency 399.78
21 Spectral Width 7512.0
22 Lowest Frequency -1757.1
23 Nucleus Proton
24 Acquired Size 14993
25 Spectral Size 32768
O Me O Me
Me
3m
2.07 1.06 2.11 2.23 1.98 2.00 1.00 1.02 3.06 3.08 3.37
10.0 9.5 9.0 8.5 8.0 7.5 7.0 6.5 6.0 5.5 5.0 4.5 4.0 3.5 3.0 2.5 2.0 1.5 1.0 0.5 0.0
f1 (ppm)

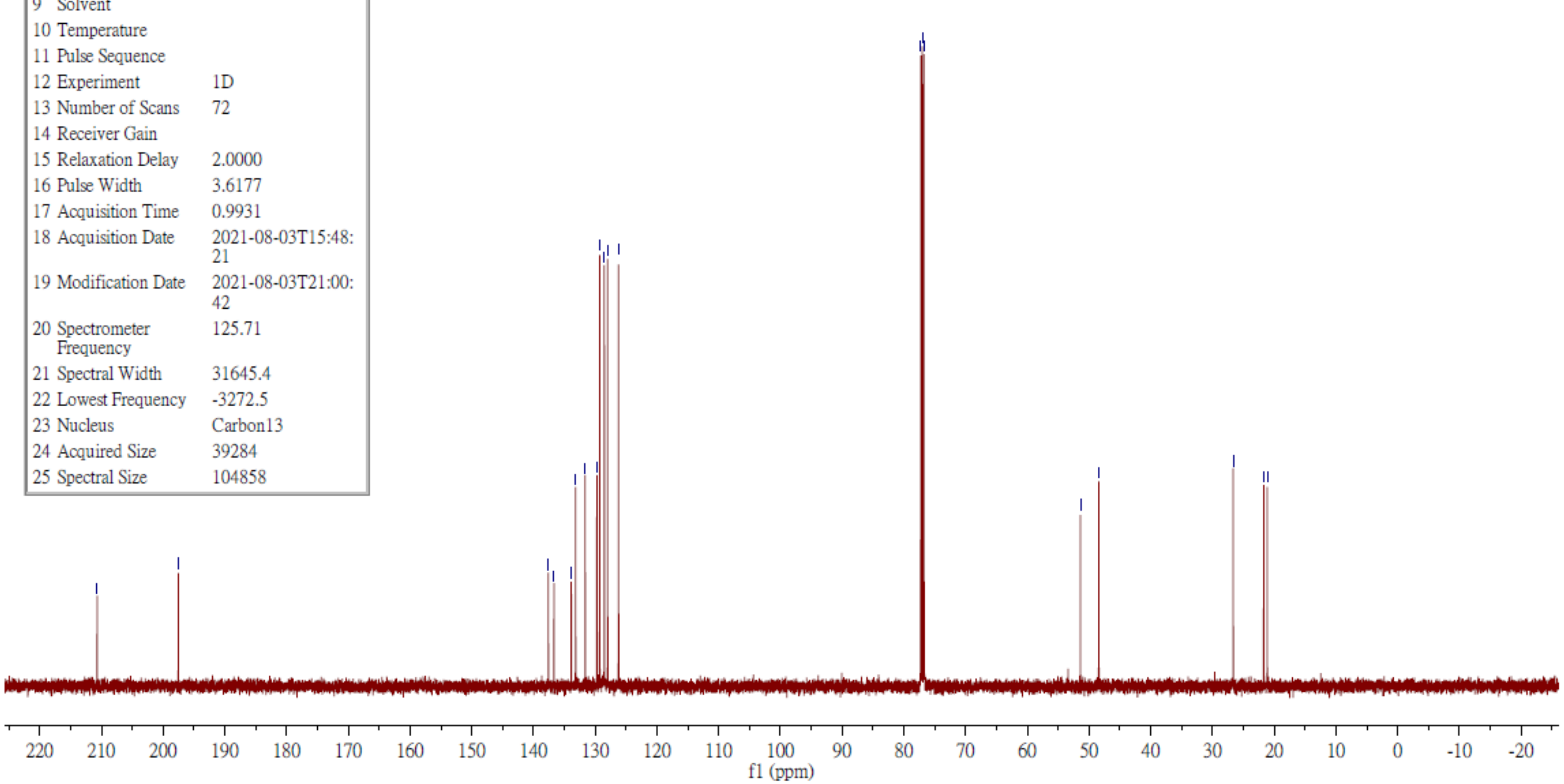

	Parameter	Value
1	Data File Name	D:/ USER/ UH/ LAB/ data/ L-003-002-13C_CARBON-2-2.jdf
2	Title	L-003-002-13C
3	Comment	
4	Origin	JEOL
5	Owner	
6	Site	
7	Spectrometer	SERC ECZ500R
8	Author	May
9	Solvent	
10	Temperature	
11	Pulse Sequence	
12	Experiment	1D
13	Number of Scans	72
14	Receiver Gain	
15	Relaxation Delay	2.0000
16	Pulse Width	3.6177
17	Acquisition Time	0.9931
18	Acquisition Date	2021-08-03T15:48:21
19	Modification Date	2021-08-03T21:00:42
20	Spectrometer Frequency	125.71
21	Spectral Width	31645.4
22	Lowest Frequency	-3272.5
23	Nucleus	Carbon13
24	Acquired Size	39284
25	Spectral Size	104858

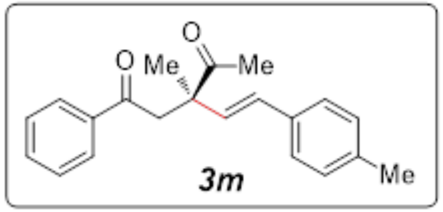

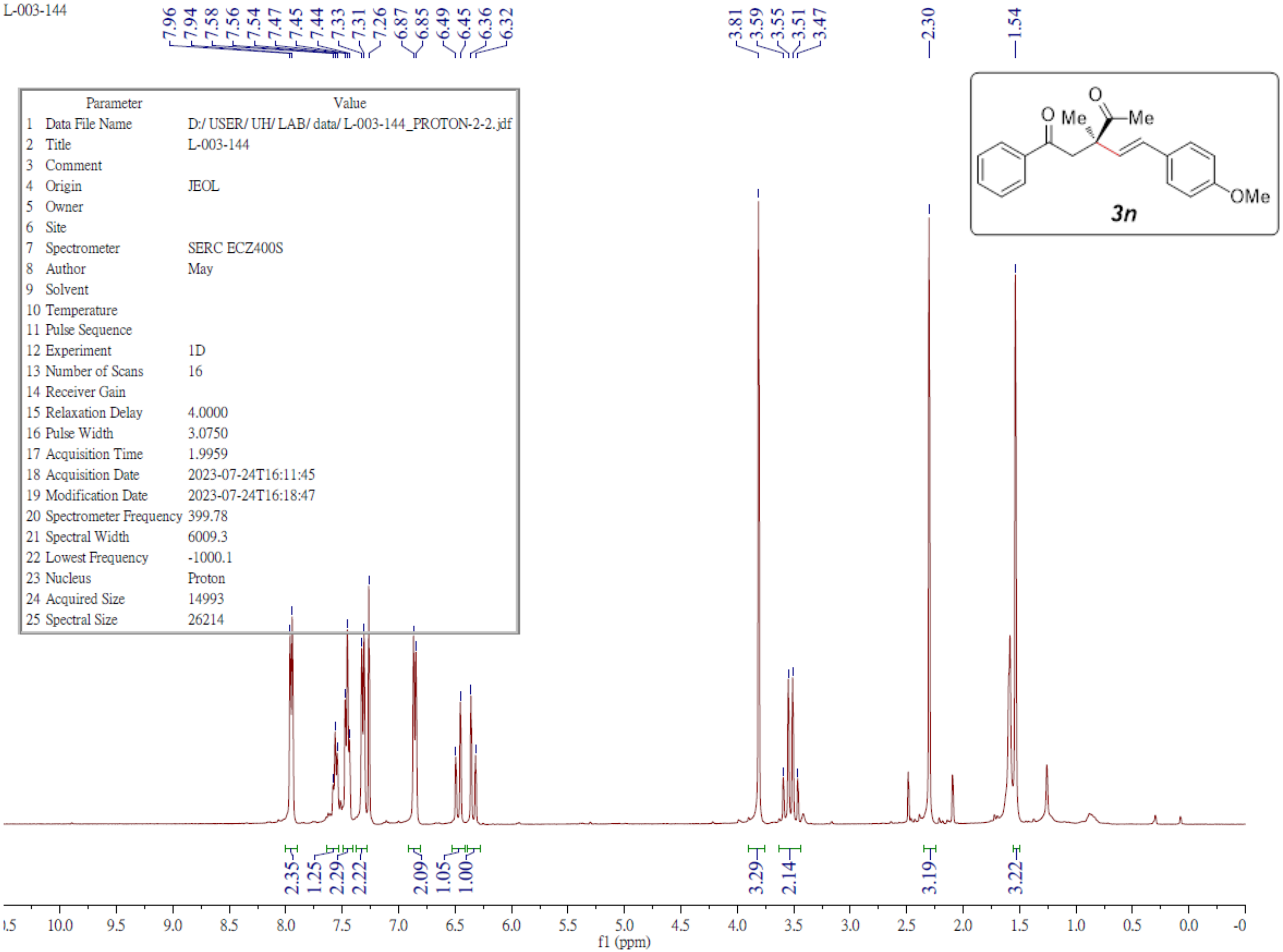

	Parameter	Value
1	Data File Name	D:/ USER/ UH/ LAB/ data/ L-003-144_PROTON-2-2.jdf
2	Title	L-003-144
3	Comment	
4	Origin	JEOL
5	Owner	
6	Site	
7	Spectrometer	SERC ECZ400S
8	Author	May
9	Solvent	
10	Temperature	
11	Pulse Sequence	
12	Experiment	1D
13	Number of Scans	16
14	Receiver Gain	
15	Relaxation Delay	4.0000
16	Pulse Width	3.0750
17	Acquisition Time	1.9959
18	Acquisition Date	2023-07-24T16:11:45
19	Modification Date	2023-07-24T16:18:47
20	Spectrometer Frequency	399.78
21	Spectral Width	6009.3
22	Lowest Frequency	-1000.1
23	Nucleus	Proton
24	Acquired Size	14993
25	Spectral Size	26214

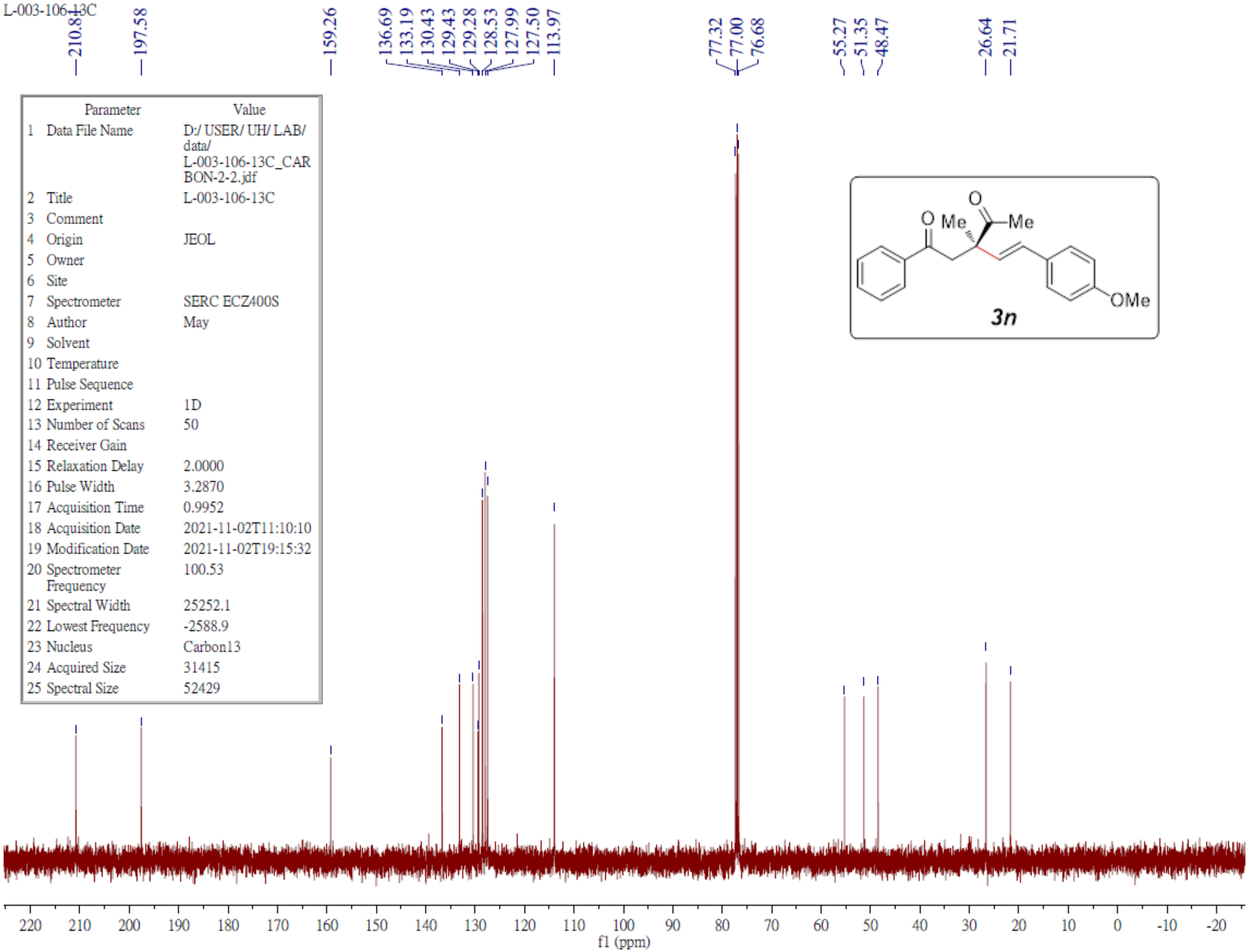

	Parameter	Value
1	Data File Name	D:/ USER/ UH/ LAB/ data/ L-003-106-13C_CARBON-2-2.jdf
2	Title	L-003-106-13C
3	Comment	
4	Origin	JEOL
5	Owner	
6	Site	
7	Spectrometer	SERC ECZ400S
8	Author	May
9	Solvent	
10	Temperature	
11	Pulse Sequence	
12	Experiment	1D
13	Number of Scans	50
14	Receiver Gain	
15	Relaxation Delay	2.0000
16	Pulse Width	3.2870
17	Acquisition Time	0.9952
18	Acquisition Date	2021-11-02T11:10:10
19	Modification Date	2021-11-02T19:15:32
20	Spectrometer Frequency	100.53
21	Spectral Width	25252.1
22	Lowest Frequency	-2588.9
23	Nucleus	Carbon13
24	Acquired Size	31415
25	Spectral Size	52429

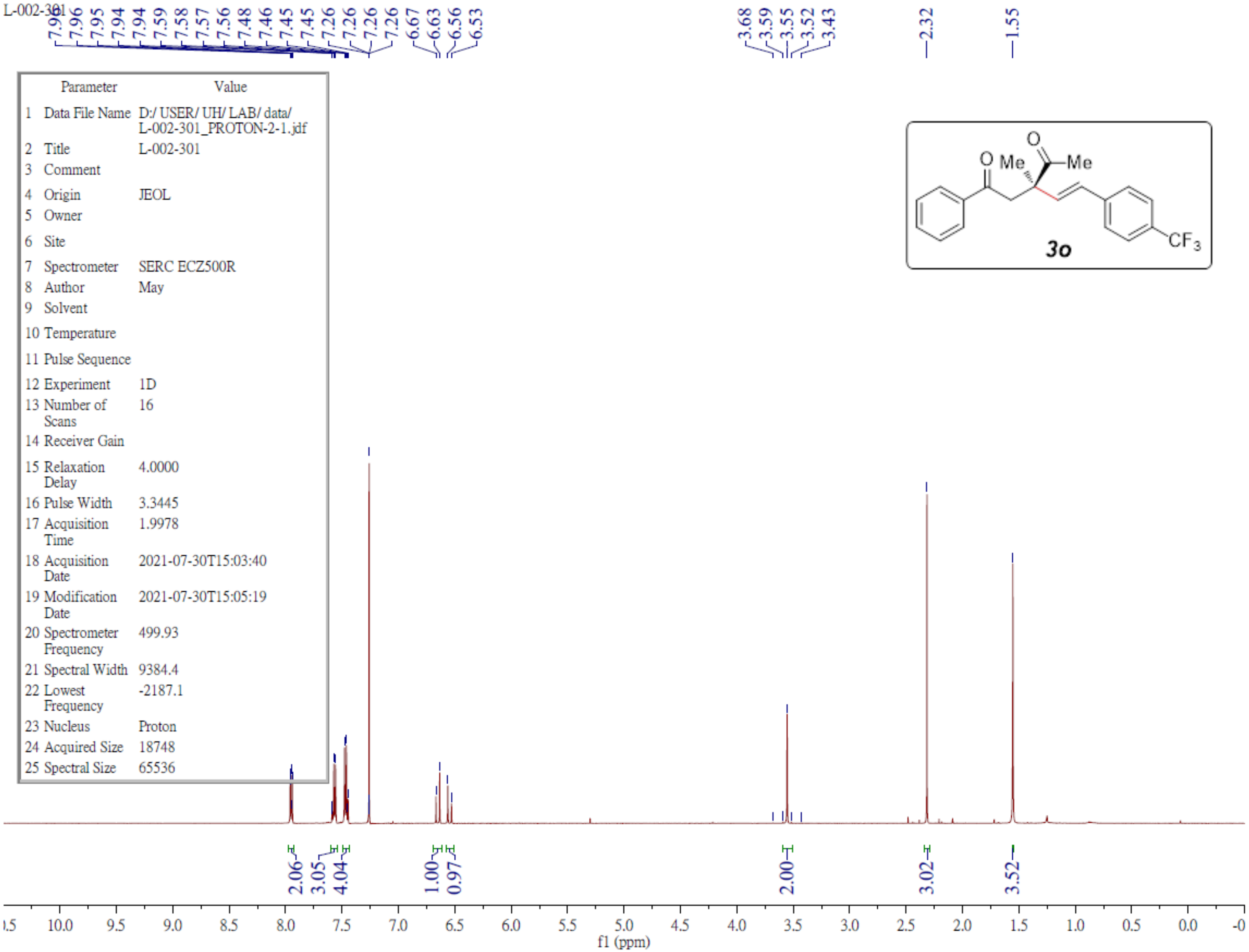

Parameter		Value
1	Data File Name	D:/ USER/ UH/ LAB/ data/ L-002-301_PROTON-2-1.jdf
2	Title	L-002-301
3	Comment	
4	Origin	JEOL
5	Owner	
6	Site	
7	Spectrometer	SERC ECZ500R
8	Author	May
9	Solvent	
10	Temperature	
11	Pulse Sequence	
12	Experiment	1D
13	Number of Scans	16
14	Receiver Gain	
15	Relaxation Delay	4.0000
16	Pulse Width	3.3445
17	Acquisition Time	1.9978
18	Acquisition Date	2021-07-30T15:03:40
19	Modification Date	2021-07-30T15:05:19
20	Spectrometer Frequency	499.93
21	Spectral Width	9384.4
22	Lowest Frequency	-2187.1
23	Nucleus	Proton
24	Acquired Size	18748
25	Spectral Size	65536

3o

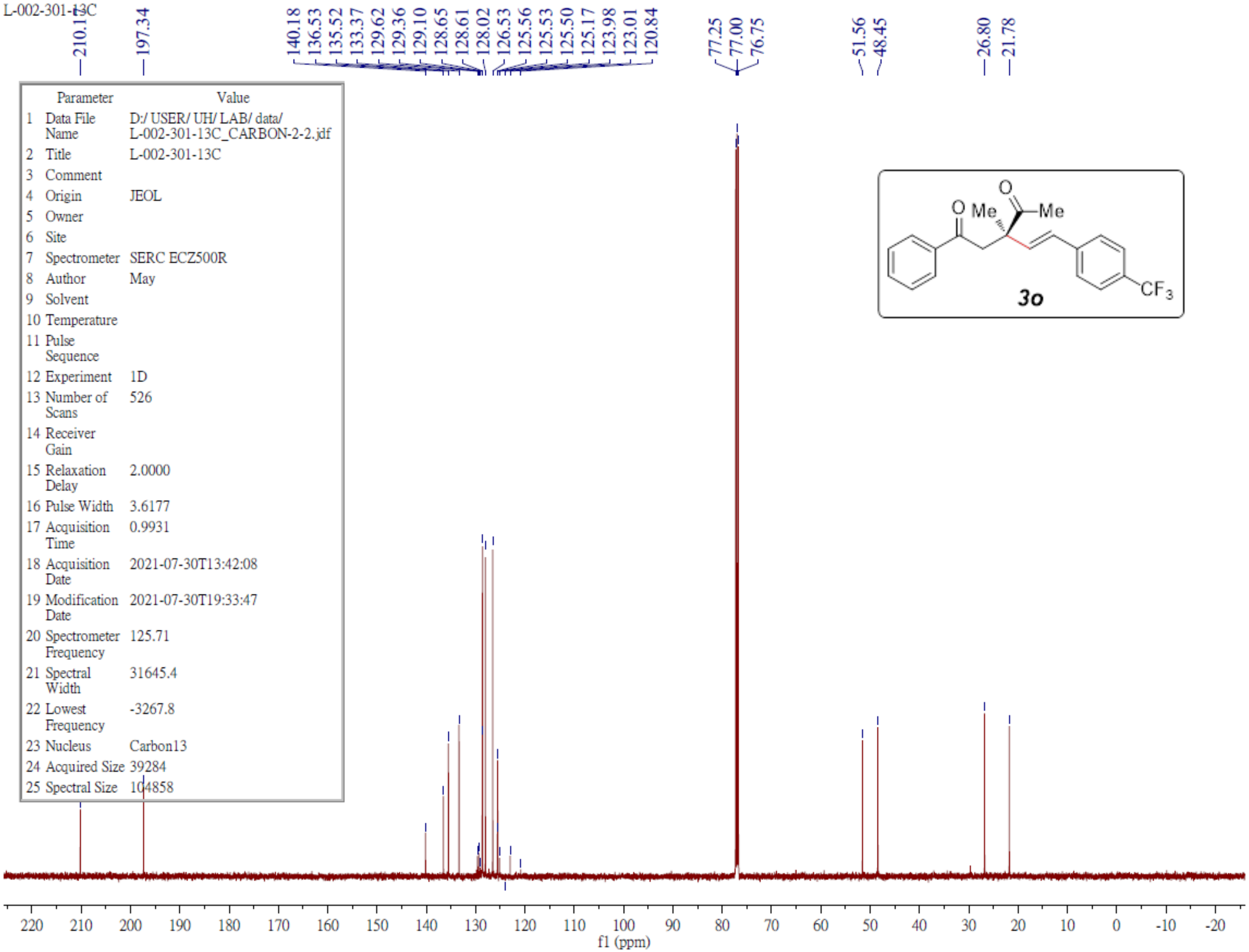

	Parameter	Value
1	Data File Name	D:/ USER/ UH/ LAB/ data/ L-002-301-13C_CARBON-2-2.jdf
2	Title	L-002-301-13C
3	Comment	
4	Origin	JEOL
5	Owner	
6	Site	
7	Spectrometer	SERC ECZ500R
8	Author	May
9	Solvent	
10	Temperature	
11	Pulse Sequence	
12	Experiment	1D
13	Number of Scans	526
14	Receiver Gain	
15	Relaxation Delay	2.0000
16	Pulse Width	3.6177
17	Acquisition Time	0.9931
18	Acquisition Date	2021-07-30T13:42:08
19	Modification Date	2021-07-30T19:33:47
20	Spectrometer Frequency	125.71
21	Spectral Width	31645.4
22	Lowest Frequency	-3267.8
23	Nucleus	Carbon13
24	Acquired Size	39284
25	Spectral Size	104858

L-002-301-F

	Parameter	Value
1	Data File Name	D:/ USER/ UH/ LAB/ data/ L-002-301-F_Fluorine-2-2.jdf
2	Title	L-002-301-F
3	Comment	
4	Origin	JEOL
5	Owner	
6	Site	
7	Spectrometer	SERC ECZ400S
8	Author	May
9	Solvent	
10	Temperature	
11	Pulse Sequence	
12	Experiment	1D
13	Number of Scans	5
14	Receiver Gain	
15	Relaxation Delay	4.0000
16	Pulse Width	3.1850
17	Acquisition Time	1.9862
18	Acquisition Date	2023-07-14T12:02:36
19	Modification Date	2023-07-14T12:28:30
20	Spectrometer Frequency	376.17
21	Spectral Width	75757.3
22	Lowest Frequency	-75495.8
23	Nucleus	Fluorine19
24	Acquired Size	188087
25	Spectral Size	419430

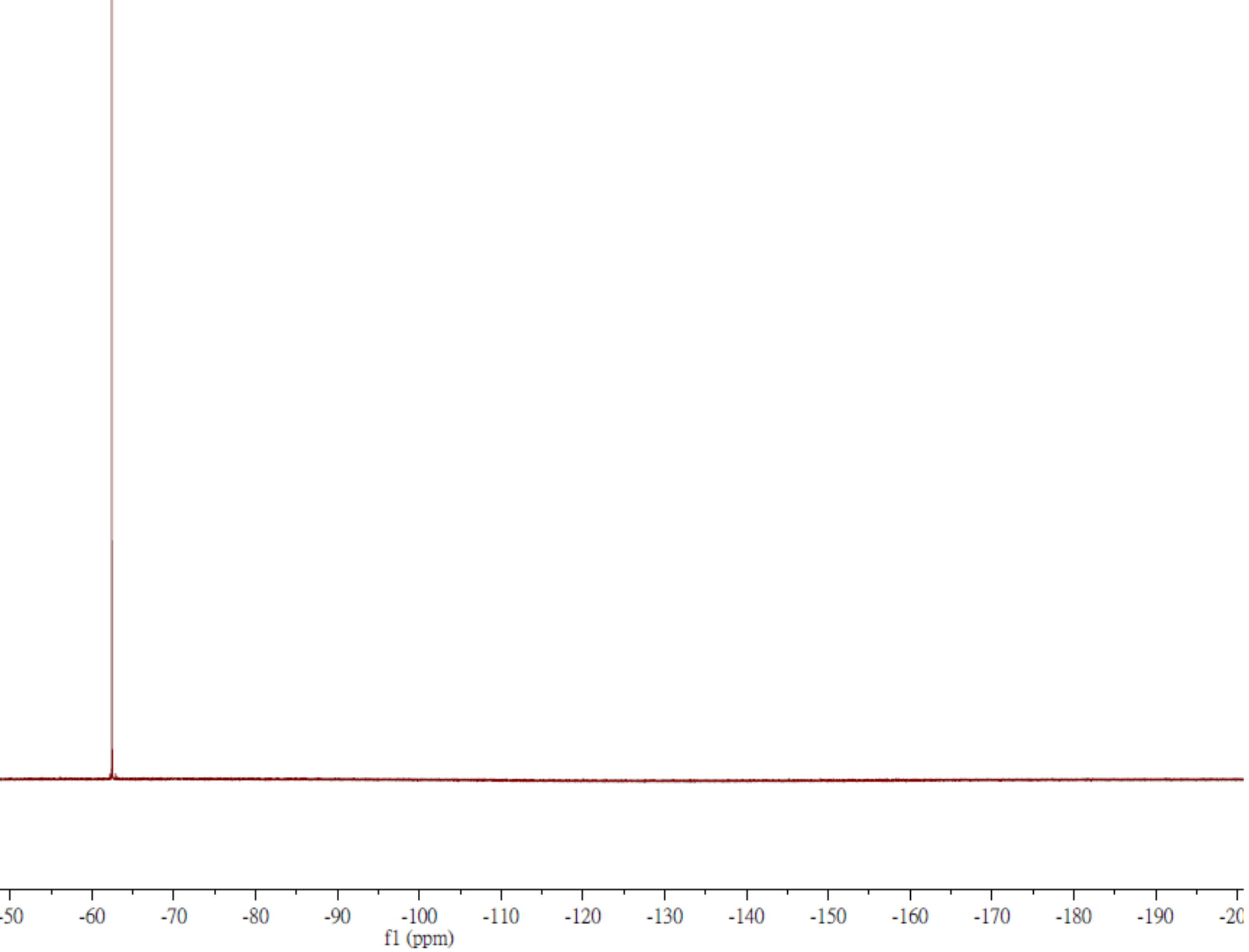

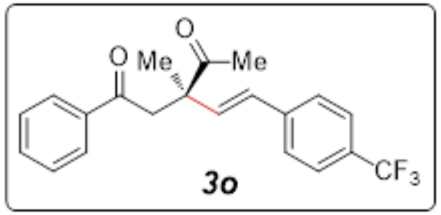

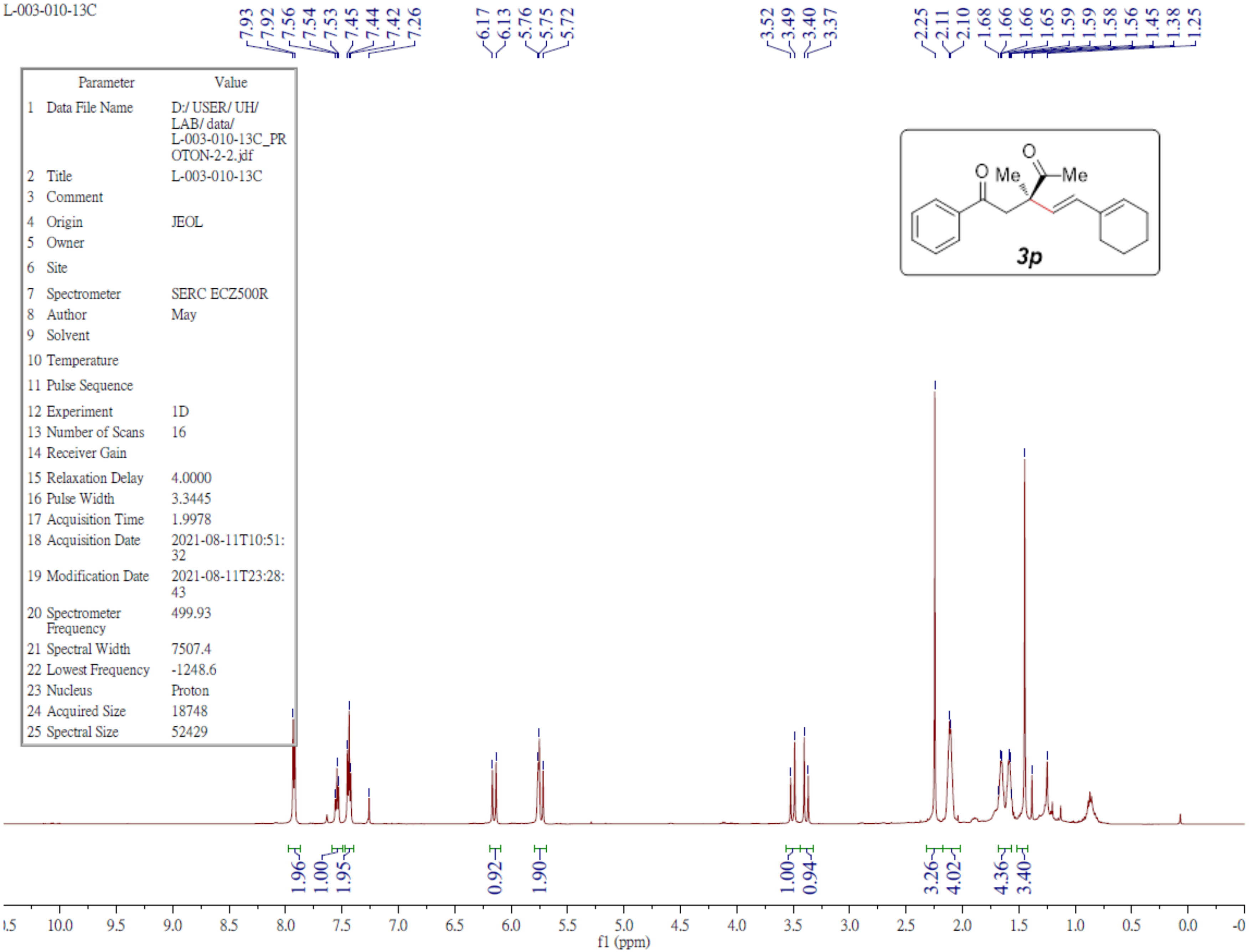

	Parameter	Value
1	Data File Name	D:/ USER/ UH/ LAB/ data/ L-003-010-13C_PROTON-2-2.jdf
2	Title	L-003-010-13C
3	Comment	
4	Origin	JEOL
5	Owner	
6	Site	
7	Spectrometer	SERC ECZ500R
8	Author	May
9	Solvent	
10	Temperature	
11	Pulse Sequence	
12	Experiment	1D
13	Number of Scans	16
14	Receiver Gain	
15	Relaxation Delay	4.0000
16	Pulse Width	3.3445
17	Acquisition Time	1.9978
18	Acquisition Date	2021-08-11T10:51:32
19	Modification Date	2021-08-11T23:28:43
20	Spectrometer Frequency	499.93
21	Spectral Width	7507.4
22	Lowest Frequency	-1248.6
23	Nucleus	Proton
24	Acquired Size	18748
25	Spectral Size	52429

L-003-010-13C

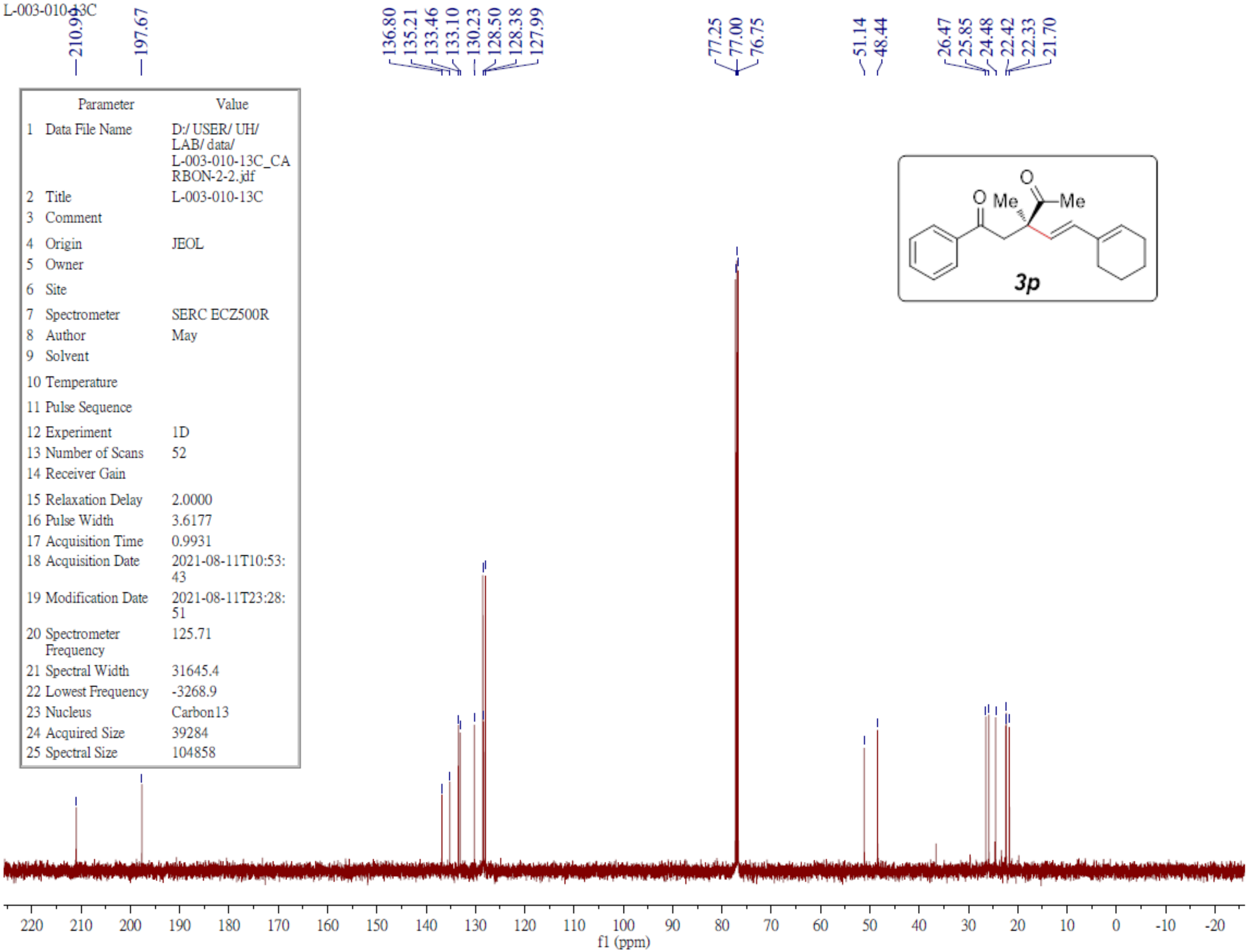

	Parameter	Value
1	Data File Name	D:/ USER/ UH/ LAB/ data/ L-003-010-13C_CARBON-2-2.jdf
2	Title	L-003-010-13C
3	Comment	
4	Origin	JEOL
5	Owner	
6	Site	
7	Spectrometer	SERC ECZ500R
8	Author	May
9	Solvent	
10	Temperature	
11	Pulse Sequence	
12	Experiment	1D
13	Number of Scans	52
14	Receiver Gain	
15	Relaxation Delay	2.0000
16	Pulse Width	3.6177
17	Acquisition Time	0.9931
18	Acquisition Date	2021-08-11T10:53:43
19	Modification Date	2021-08-11T23:28:51
20	Spectrometer Frequency	125.71
21	Spectral Width	31645.4
22	Lowest Frequency	-3268.9
23	Nucleus	Carbon13
24	Acquired Size	39284
25	Spectral Size	104858

L-003-294-13C

	Parameter	Value
1	Data File Name	D:/ USER/ UH/ LAB/ data/ L-003-294-13C_ PROTON-2-2.jdf
2	Title	L-003-294-13C
3	Comment	
4	Origin	JEOL
5	Owner	
6	Site	
7	Spectrometer	SERC ECZ500R
8	Author	May
9	Solvent	
10	Temperature	
11	Pulse Sequence	
12	Experiment	1D
13	Number of Scans	10
14	Receiver Gain	
15	Relaxation Delay	4.0000
16	Pulse Width	3.3445
17	Acquisition Time	1.9978
18	Acquisition Date	2022-06-18T14:41:53
19	Modification Date	2022-06-19T01:13:40
20	Spectrometer Frequency	499.93
21	Spectral Width	7507.4
22	Lowest Frequency	-1248.6
23	Nucleus	Proton
24	Acquired Size	18748
25	Spectral Size	52429

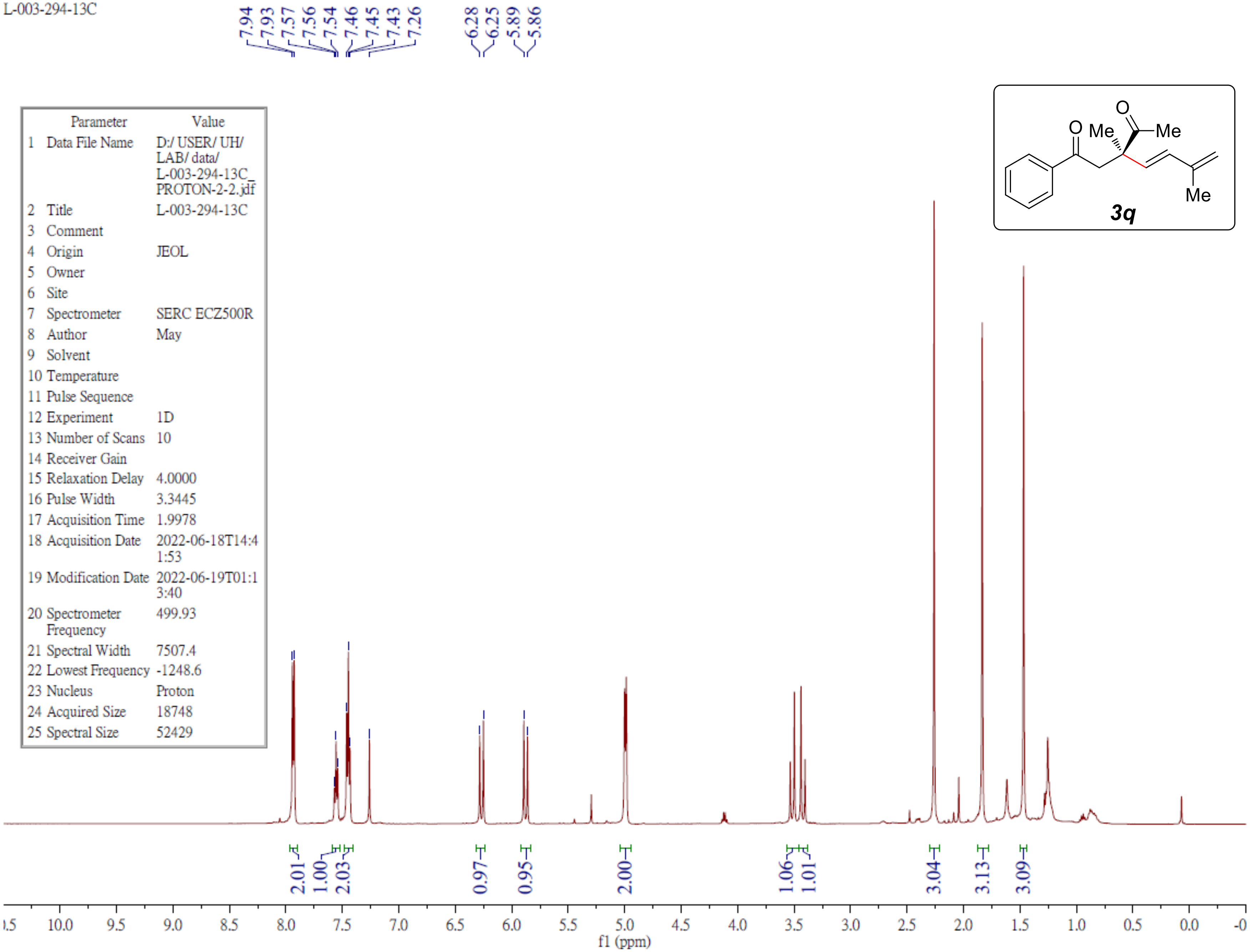

3q

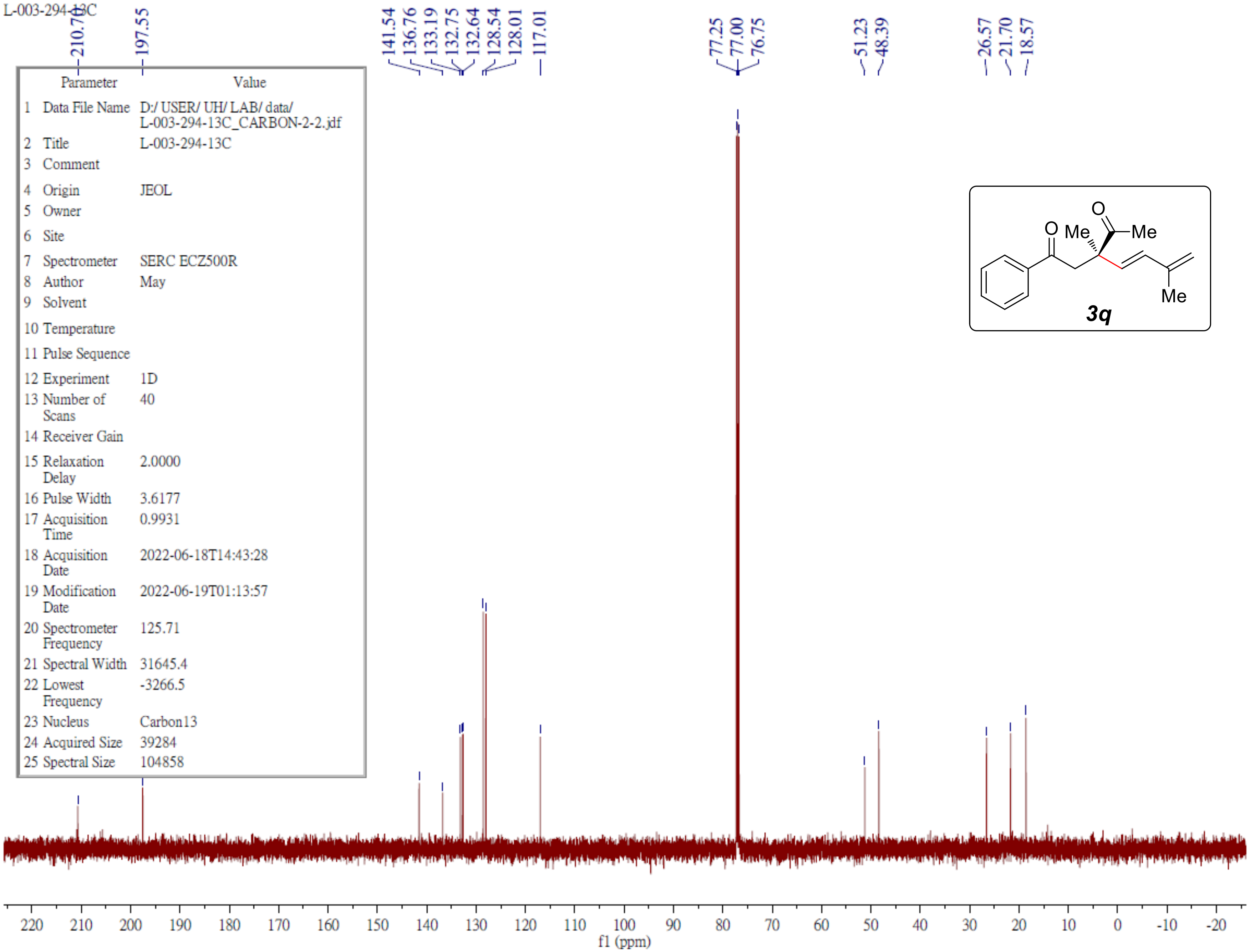

	Parameter	Value
1	Data File Name	D:/ USER/ UH/ LAB/ data/ L-003-294-13C_CARBON-2-2.jdf
2	Title	L-003-294-13C
3	Comment	
4	Origin	JEOL
5	Owner	
6	Site	
7	Spectrometer	SERC ECZ500R
8	Author	May
9	Solvent	
10	Temperature	
11	Pulse Sequence	
12	Experiment	1D
13	Number of Scans	40
14	Receiver Gain	
15	Relaxation Delay	2.0000
16	Pulse Width	3.6177
17	Acquisition Time	0.9931
18	Acquisition Date	2022-06-18T14:43:28
19	Modification Date	2022-06-19T01:13:57
20	Spectrometer Frequency	125.71
21	Spectral Width	31645.4
22	Lowest Frequency	-3266.5
23	Nucleus	Carbon13
24	Acquired Size	39284
25	Spectral Size	104858

3q

L-002-158-13C

	Parameter	Value
1	Data File Name	D:/ USER/ UH/ LAB/ data/ L-002-158-13C_ PROTON-1-2.jdf
2	Title	L-002-158-13C
3	Comment	
4	Origin	JEOL
5	Owner	
6	Site	
7	Spectrometer	SERC400
8	Author	May
9	Solvent	
10	Temperature	
11	Pulse Sequence	
12	Experiment	1D
13	Number of Scans	8
14	Receiver Gain	
15	Relaxation Delay	4.0000
16	Pulse Width	6.4100
17	Acquisition Time	2.1837
18	Acquisition Date	2021-04-26T12: 41:28
19	Modification Date	2021-04-28T23: 37:29
20	Spectrometer Frequency	399.78
21	Spectral Width	6001.9
22	Lowest Frequency	-998.8
23	Nucleus	1H
24	Acquired Size	16384
25	Spectral Size	13107

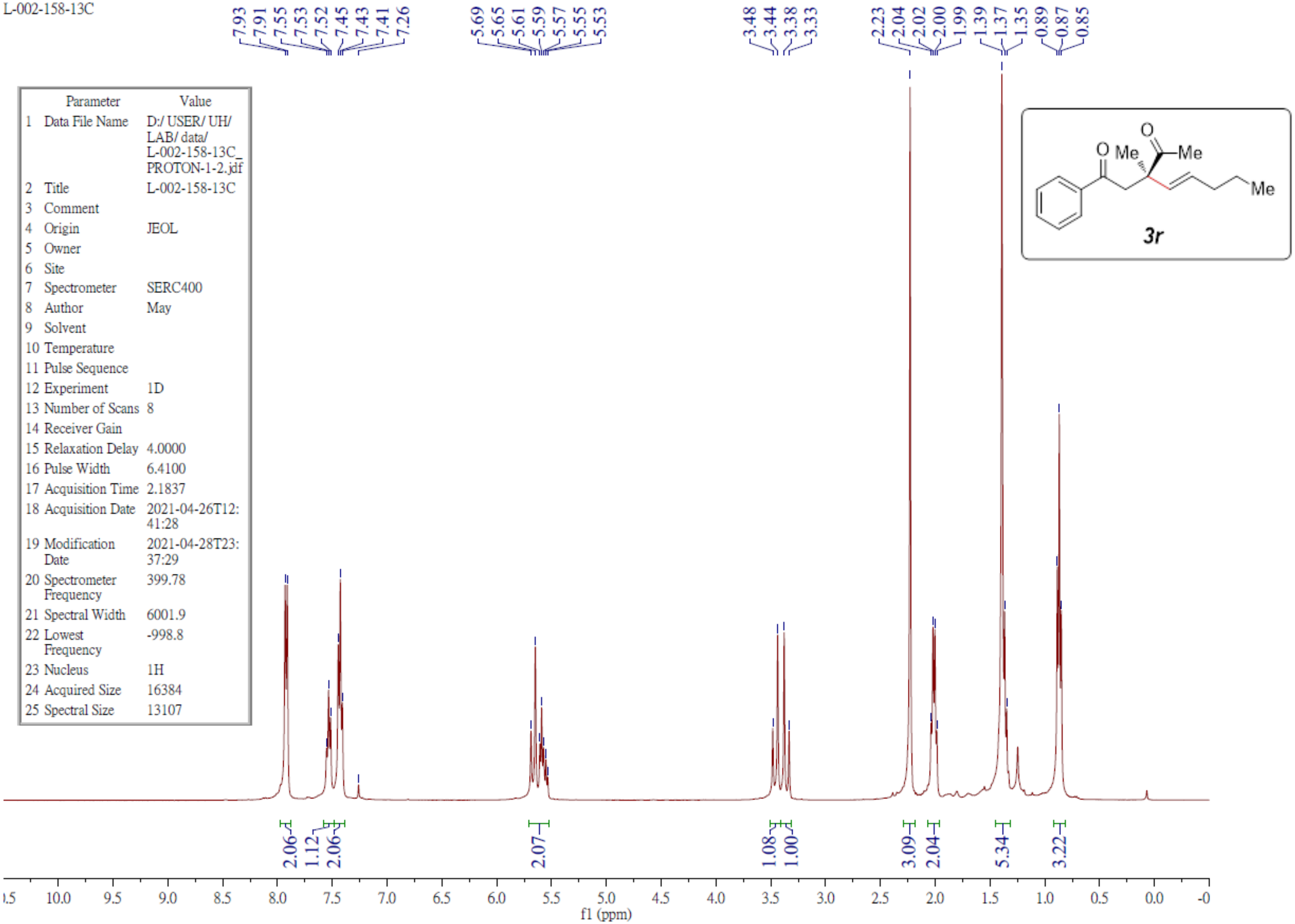

3r

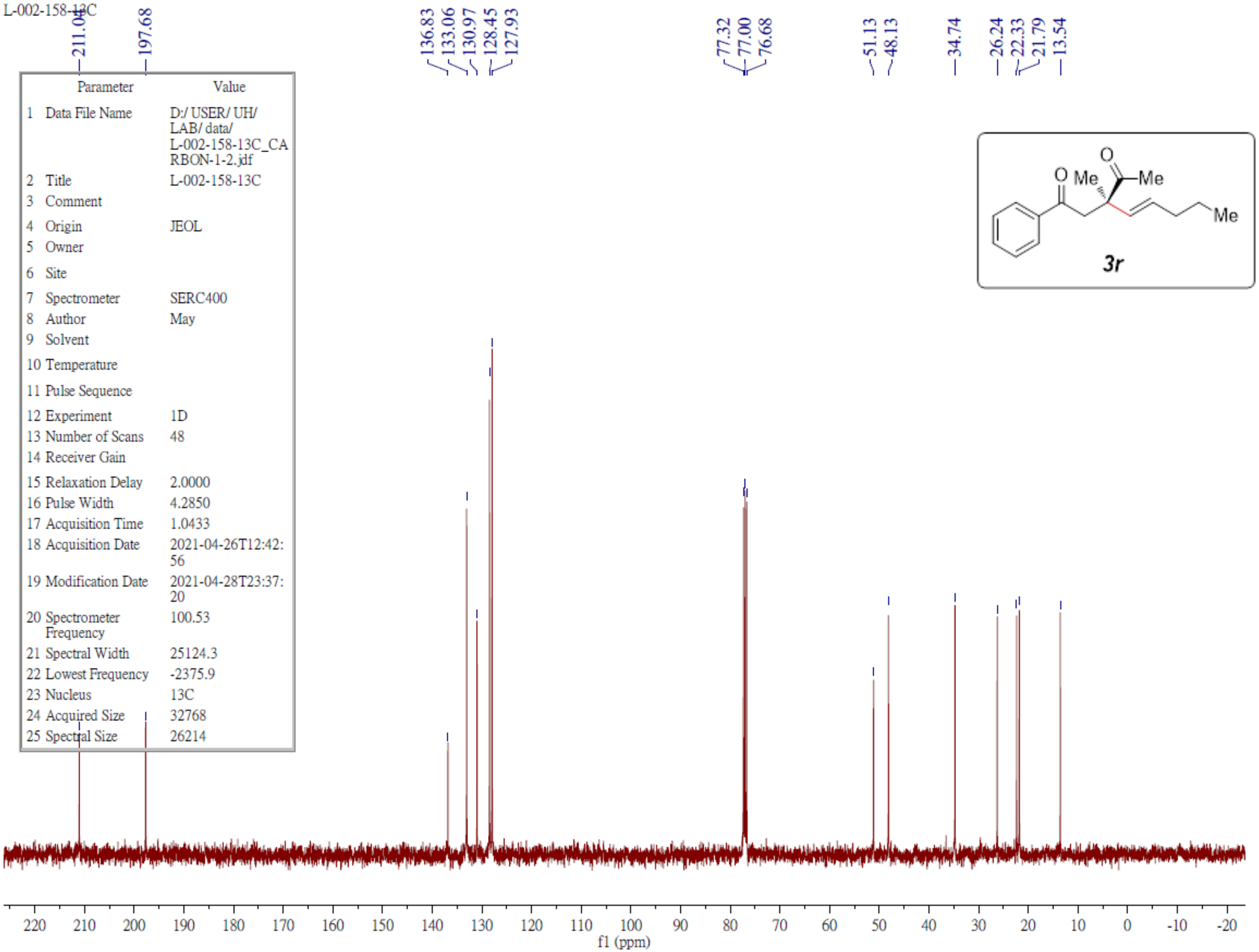

L-002-158-13C
211.04
197.68
136.83
133.06
130.97
128.45
127.93
77.32
77.00
76.68
51.13
48.13
34.74
26.24
22.33
21.79
13.54
Parameter | Value
1 Data File Name | D:/ USER/ UH/ LAB/ data/ L-002-158-13C_CARBON-1-2.jdf
2 Title | L-002-158-13C
3 Comment
4 Origin | JEOL
5 Owner
6 Site
7 Spectrometer | SERC400
8 Author | May
9 Solvent
10 Temperature
11 Pulse Sequence
12 Experiment | 1D
13 Number of Scans | 48
14 Receiver Gain
15 Relaxation Delay | 2.0000
16 Pulse Width | 4.2850
17 Acquisition Time | 1.0433
18 Acquisition Date | 2021-04-26T12:42:56
19 Modification Date | 2021-04-28T23:37:20
20 Spectrometer Frequency | 100.53
21 Spectral Width | 25124.3
22 Lowest Frequency | -2375.9
23 Nucleus | 13C
24 Acquired Size | 32768
25 Spectral Size | 26214
O
Me
O
Me
Me
3r
220 210 200 190 180 170 160 150 140 130 120 110 100 90 80 70 60 50 40 30 20 10 0 -10 -20
f1 (ppm)

L-003-296

Parameter	Value
1 Data File Name	D:/ USER/ UH/ LAB/ data/ L-003-296_PROTON-1-2.jdf
2 Title	L-003-296
3 Comment	
4 Origin	JEOL
5 Owner	
6 Site	
7 Spectrometer	SERC600
8 Author	May
9 Solvent	
10 Temperature	
11 Pulse Sequence	
12 Experiment	1D
13 Number of Scans	16
14 Receiver Gain	
15 Relaxation Delay	4.0000
16 Pulse Width	5.0950
17 Acquisition Time	1.4549
18 Acquisition Date	2022-06-20T13:26:12
19 Modification Date	2022-06-20T19:21:11
20 Spectrometer Frequency	600.17
21 Spectral Width	9008.2
22 Lowest Frequency	-1496.0
23 Nucleus	1H
24 Acquired Size	16384
25 Spectral Size	13107

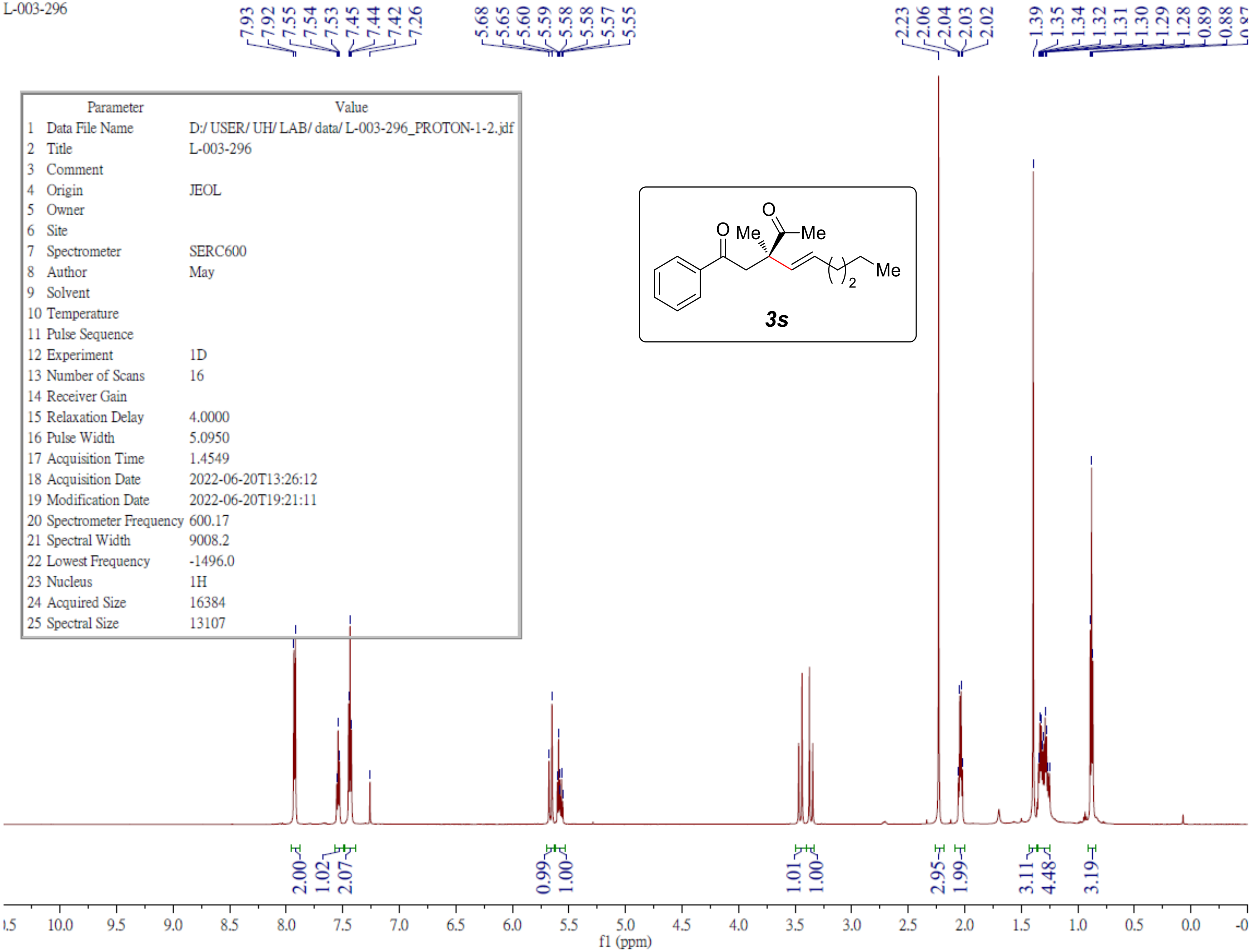

3s

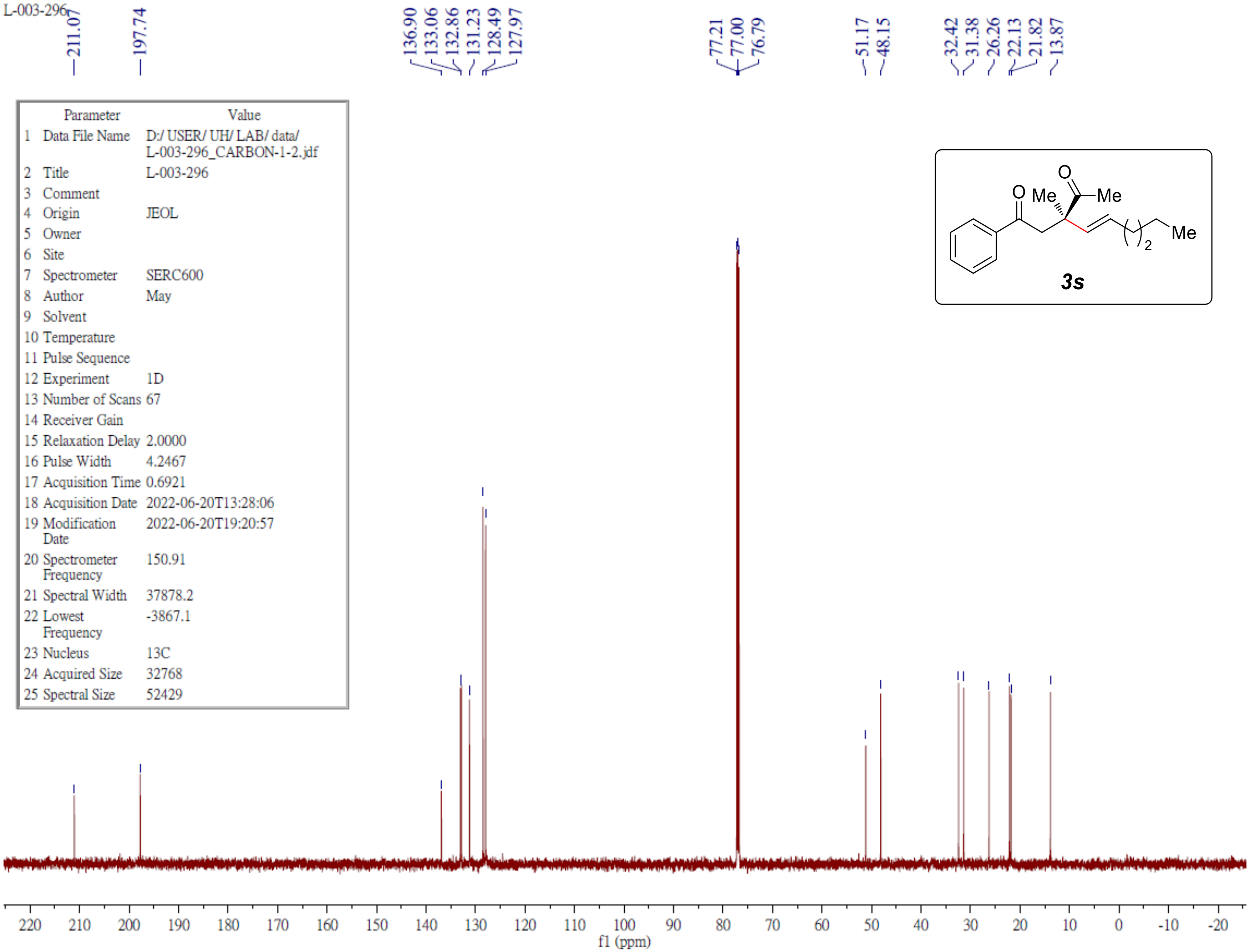

	Parameter	Value
1	Data File Name	D:/ USER/ UH/ LAB/ data/ L-003-296_CARBON-1-2.jdf
2	Title	L-003-296
3	Comment	
4	Origin	JEOL
5	Owner	
6	Site	
7	Spectrometer	SERC600
8	Author	May
9	Solvent	
10	Temperature	
11	Pulse Sequence	
12	Experiment	1D
13	Number of Scans	67
14	Receiver Gain	
15	Relaxation Delay	2.0000
16	Pulse Width	4.2467
17	Acquisition Time	0.6921
18	Acquisition Date	2022-06-20T13:28:06
19	Modification Date	2022-06-20T19:20:57
20	Spectrometer Frequency	150.91
21	Spectral Width	37878.2
22	Lowest Frequency	-3867.1
23	Nucleus	13C
24	Acquired Size	32768
25	Spectral Size	52429

3s

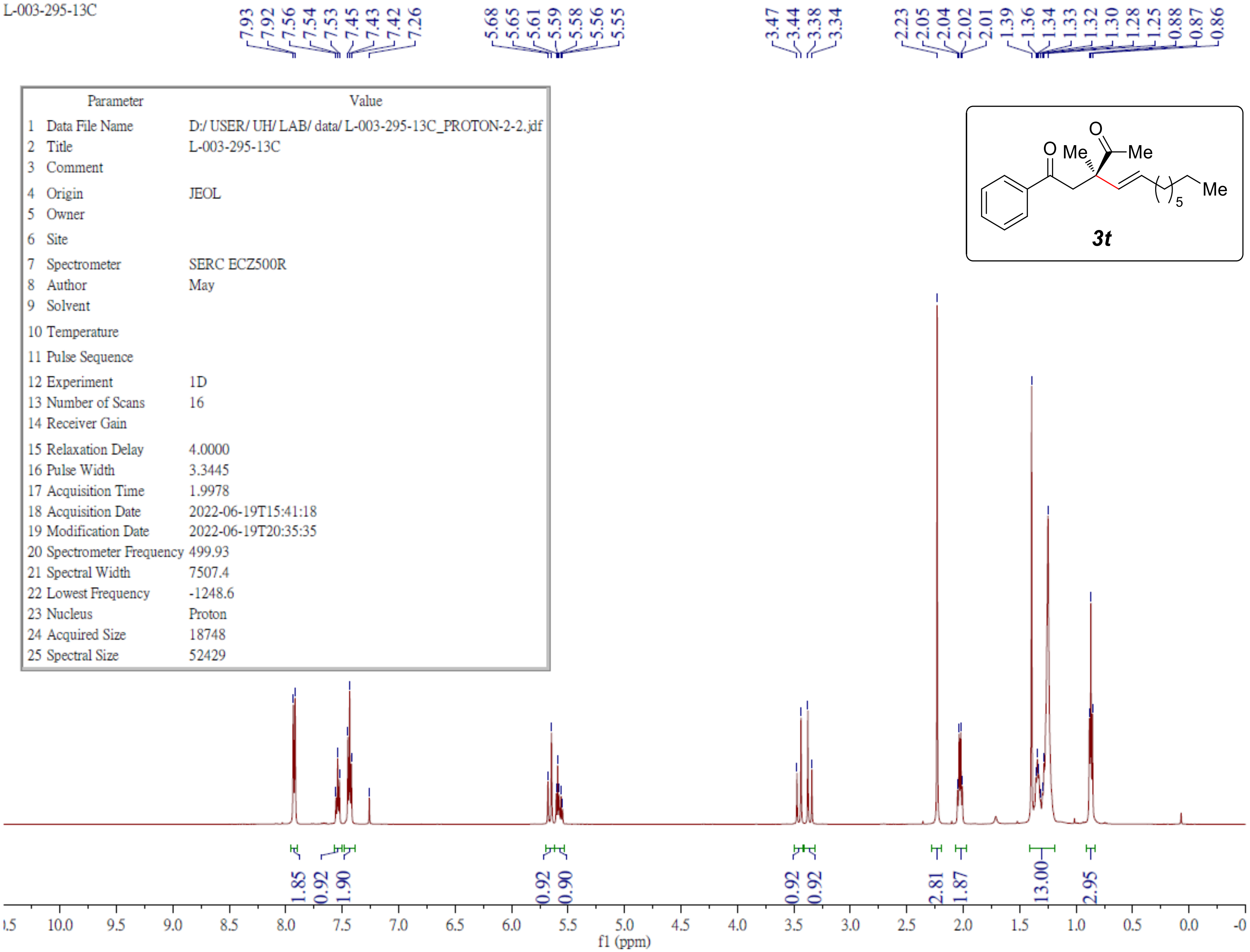

Parameter	Value
1 Data File Name	D:/ USER/ UH/ LAB/ data/ L-003-295-13C_PROTON-2-2.jdf
2 Title	L-003-295-13C
3 Comment	
4 Origin	JEOL
5 Owner	
6 Site	
7 Spectrometer	SERC ECZ500R
8 Author	May
9 Solvent	
10 Temperature	
11 Pulse Sequence	
12 Experiment	1D
13 Number of Scans	16
14 Receiver Gain	
15 Relaxation Delay	4.0000
16 Pulse Width	3.3445
17 Acquisition Time	1.9978
18 Acquisition Date	2022-06-19T15:41:18
19 Modification Date	2022-06-19T20:35:35
20 Spectrometer Frequency	499.93
21 Spectral Width	7507.4
22 Lowest Frequency	-1248.6
23 Nucleus	Proton
24 Acquired Size	18748
25 Spectral Size	52429

3t

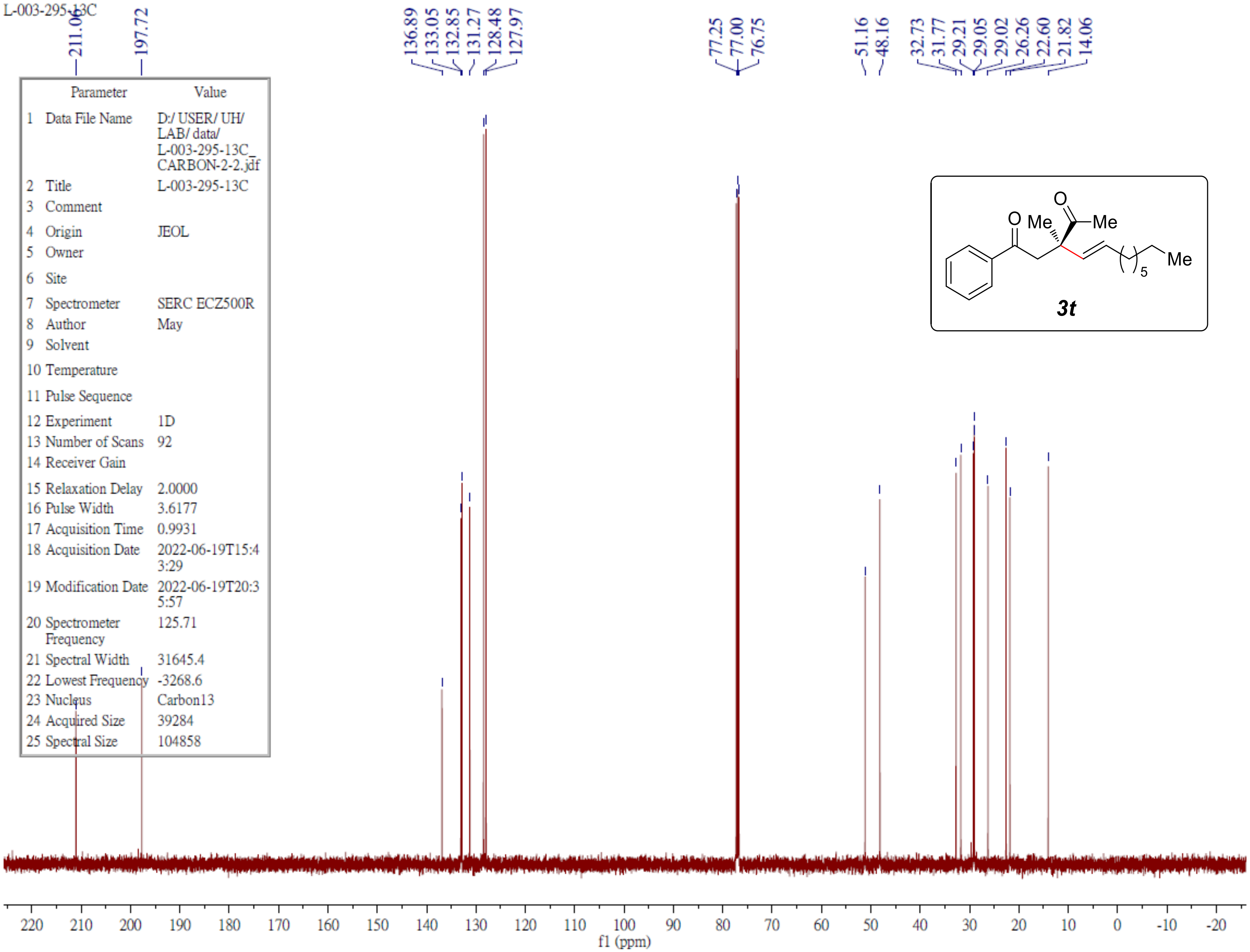

	Parameter	Value
1	Data File Name	D:/ USER/ UH/ LAB/ data/ L-003-295-13C_ CARBON-2-2.jdf
2	Title	L-003-295-13C
3	Comment	
4	Origin	JEOL
5	Owner	
6	Site	
7	Spectrometer	SERC ECZ500R
8	Author	May
9	Solvent	
10	Temperature	
11	Pulse Sequence	
12	Experiment	1D
13	Number of Scans	92
14	Receiver Gain	
15	Relaxation Delay	2.0000
16	Pulse Width	3.6177
17	Acquisition Time	0.9931
18	Acquisition Date	2022-06-19T15:4 3:29
19	Modification Date	2022-06-19T20:3 5:57
20	Spectrometer Frequency	125.71
21	Spectral Width	31645.4
22	Lowest Frequency	-3268.6
23	Nucleus	Carbon13
24	Acquired Size	39284
25	Spectral Size	104858

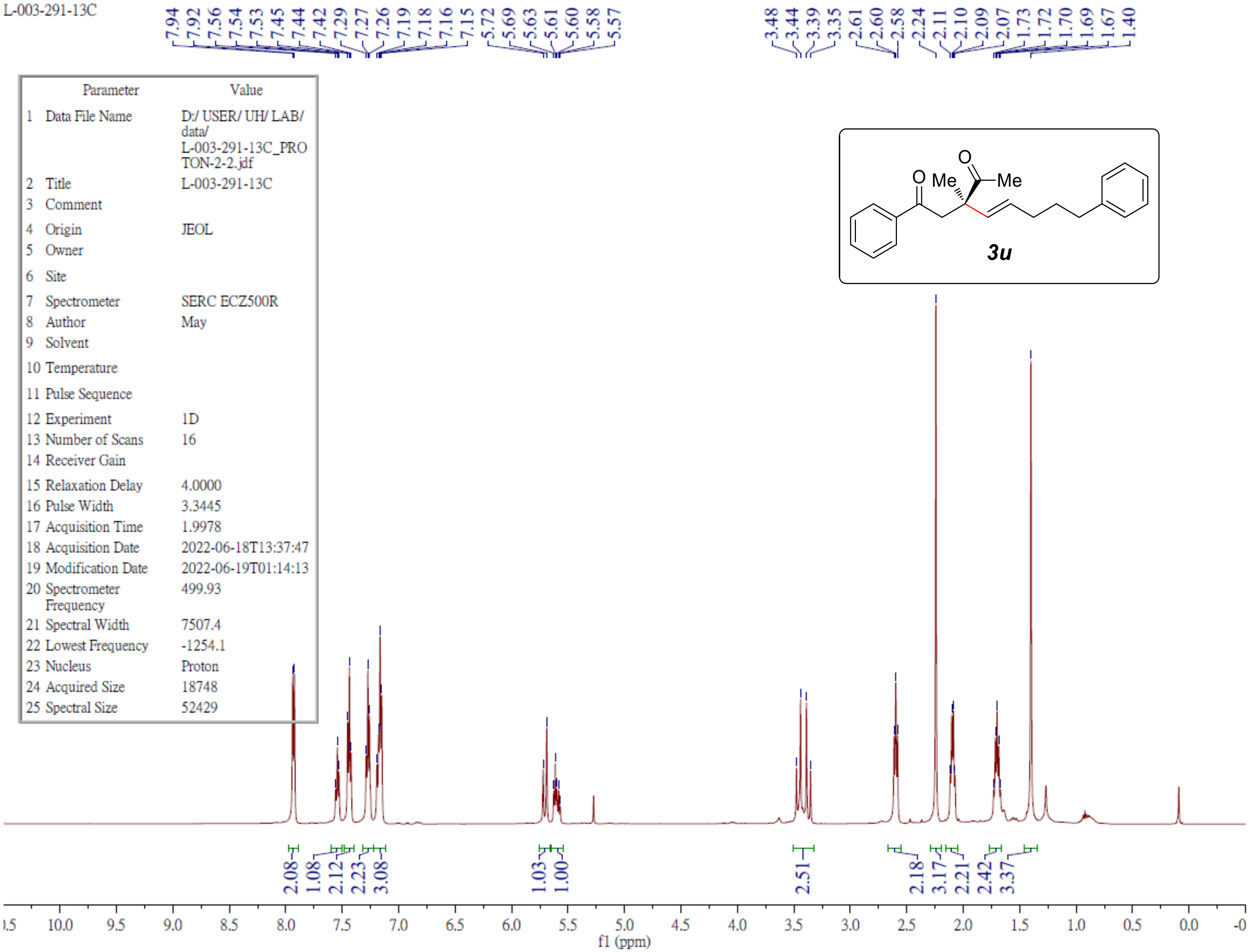

	Parameter	Value
1	Data File Name	D:/ USER/ UH/ LAB/ data/ L-003-291-13C_PROTON-2-2.jdf
2	Title	L-003-291-13C
3	Comment	
4	Origin	JEOL
5	Owner	
6	Site	
7	Spectrometer	SERC ECZ500R
8	Author	May
9	Solvent	
10	Temperature	
11	Pulse Sequence	
12	Experiment	1D
13	Number of Scans	16
14	Receiver Gain	
15	Relaxation Delay	4.0000
16	Pulse Width	3.3445
17	Acquisition Time	1.9978
18	Acquisition Date	2022-06-18T13:37:47
19	Modification Date	2022-06-19T01:14:13
20	Spectrometer Frequency	499.93
21	Spectral Width	7507.4
22	Lowest Frequency	-1254.1
23	Nucleus	Proton
24	Acquired Size	18748
25	Spectral Size	52429

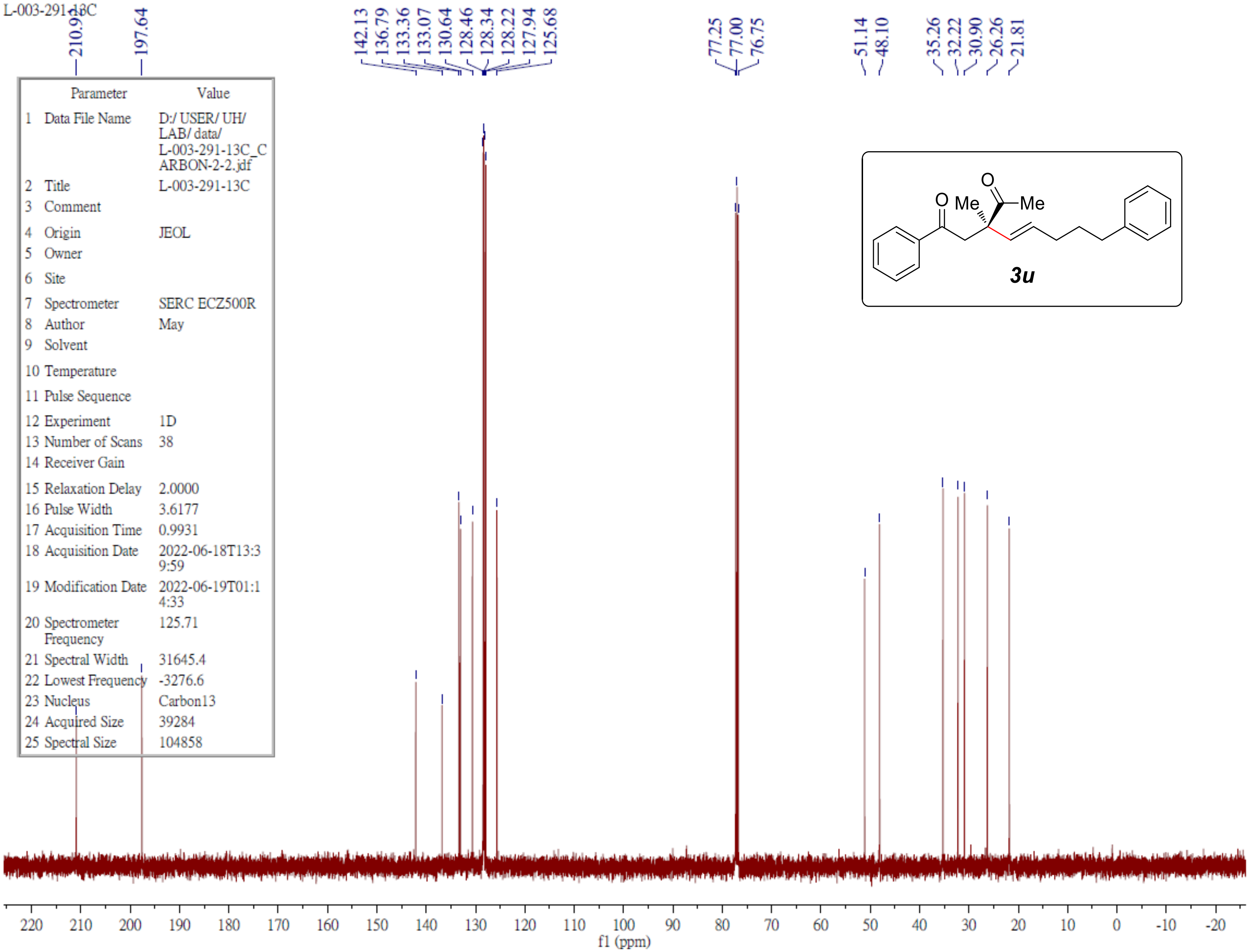

	Parameter	Value
1	Data File Name	D:/ USER/ UH/ LAB/ data/ L-003-291-13C_CARBON-2-2.jdf
2	Title	L-003-291-13C
3	Comment	
4	Origin	JEOL
5	Owner	
6	Site	
7	Spectrometer	SERC ECZ500R
8	Author	May
9	Solvent	
10	Temperature	
11	Pulse Sequence	
12	Experiment	1D
13	Number of Scans	38
14	Receiver Gain	
15	Relaxation Delay	2.0000
16	Pulse Width	3.6177
17	Acquisition Time	0.9931
18	Acquisition Date	2022-06-18T13:39:59
19	Modification Date	2022-06-19T01:14:33
20	Spectrometer Frequency	125.71
21	Spectral Width	31645.4
22	Lowest Frequency	-3276.6
23	Nucleus	Carbon13
24	Acquired Size	39284
25	Spectral Size	104858

3u

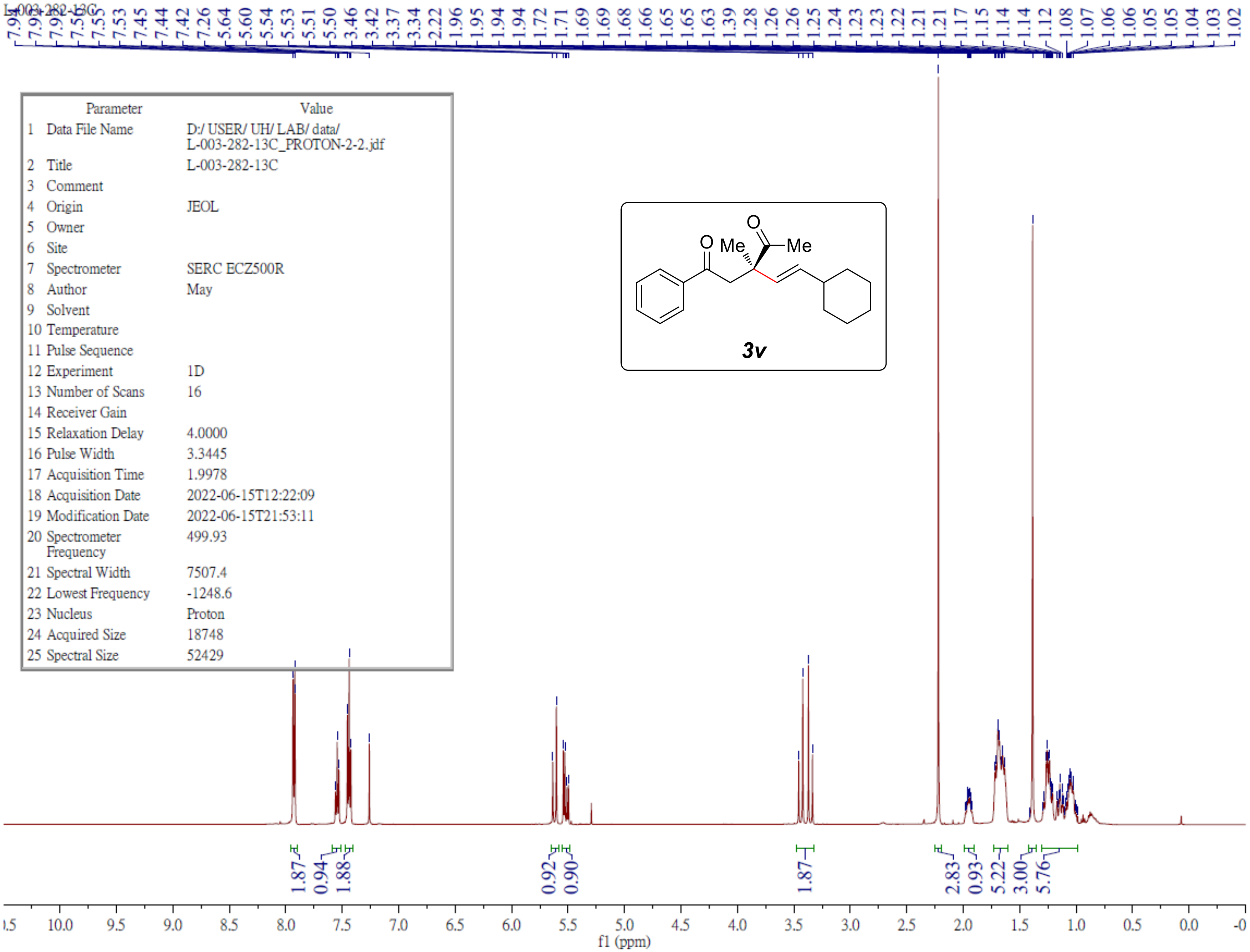

	Parameter	Value
1	Data File Name	D:/ USER/ UH/ LAB/ data/ L-003-282-13C_PROTON-2-2.jdf
2	Title	L-003-282-13C
3	Comment	
4	Origin	JEOL
5	Owner	
6	Site	
7	Spectrometer	SERC ECZ500R
8	Author	May
9	Solvent	
10	Temperature	
11	Pulse Sequence	
12	Experiment	1D
13	Number of Scans	16
14	Receiver Gain	
15	Relaxation Delay	4.0000
16	Pulse Width	3.3445
17	Acquisition Time	1.9978
18	Acquisition Date	2022-06-15T12:22:09
19	Modification Date	2022-06-15T21:53:11
20	Spectrometer Frequency	499.93
21	Spectral Width	7507.4
22	Lowest Frequency	-1248.6
23	Nucleus	Proton
24	Acquired Size	18748
25	Spectral Size	52429

L-003-282-13C

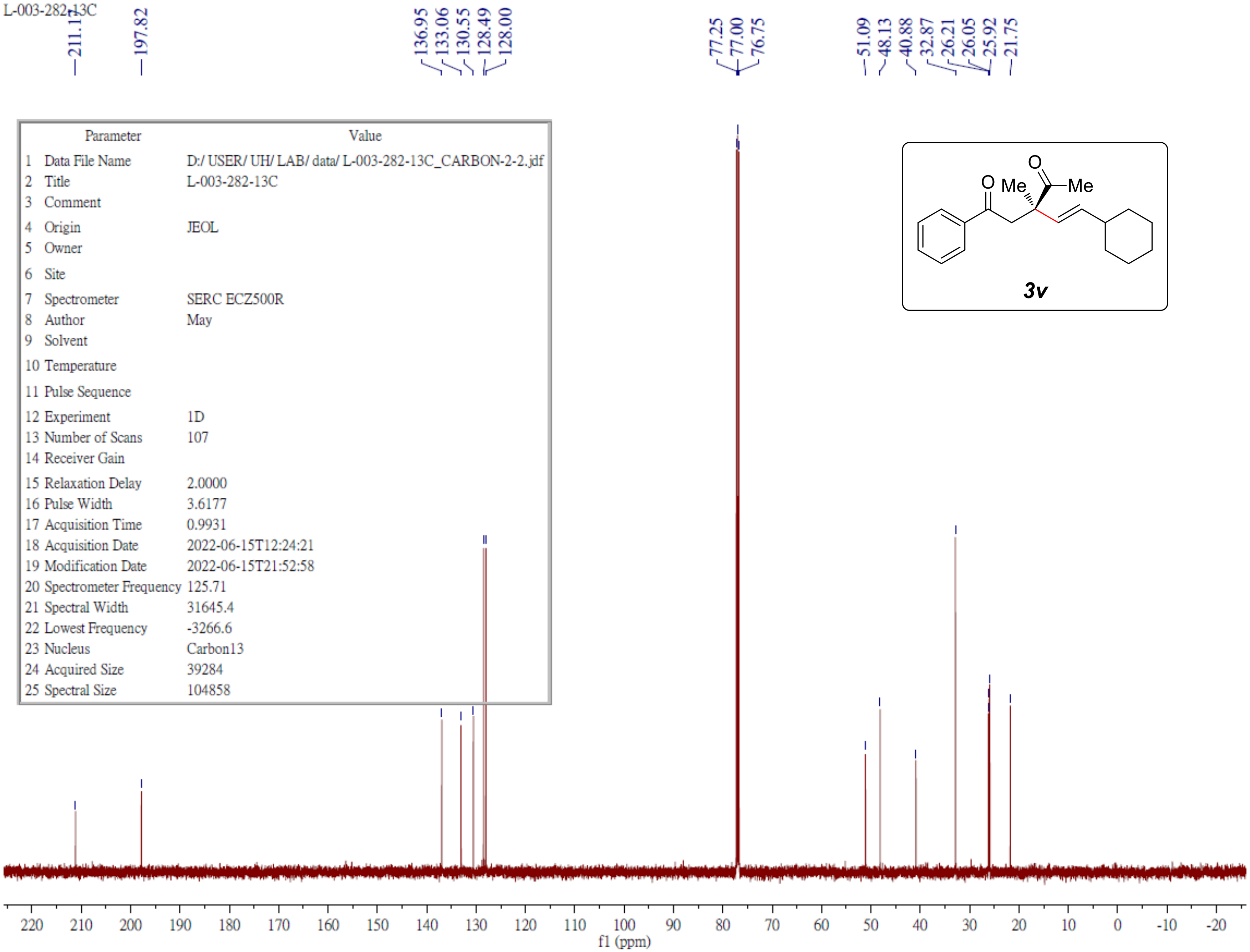

	Parameter	Value
1	Data File Name	D:/ USER/ UH/ LAB/ data/ L-003-282-13C_CARBON-2-2.jdf
2	Title	L-003-282-13C
3	Comment	
4	Origin	JEOL
5	Owner	
6	Site	
7	Spectrometer	SERC ECZ500R
8	Author	May
9	Solvent	
10	Temperature	
11	Pulse Sequence	
12	Experiment	1D
13	Number of Scans	107
14	Receiver Gain	
15	Relaxation Delay	2.0000
16	Pulse Width	3.6177
17	Acquisition Time	0.9931
18	Acquisition Date	2022-06-15T12:24:21
19	Modification Date	2022-06-15T21:52:58
20	Spectrometer Frequency	125.71
21	Spectral Width	31645.4
22	Lowest Frequency	-3266.6
23	Nucleus	Carbon13
24	Acquired Size	39284
25	Spectral Size	104858

3v

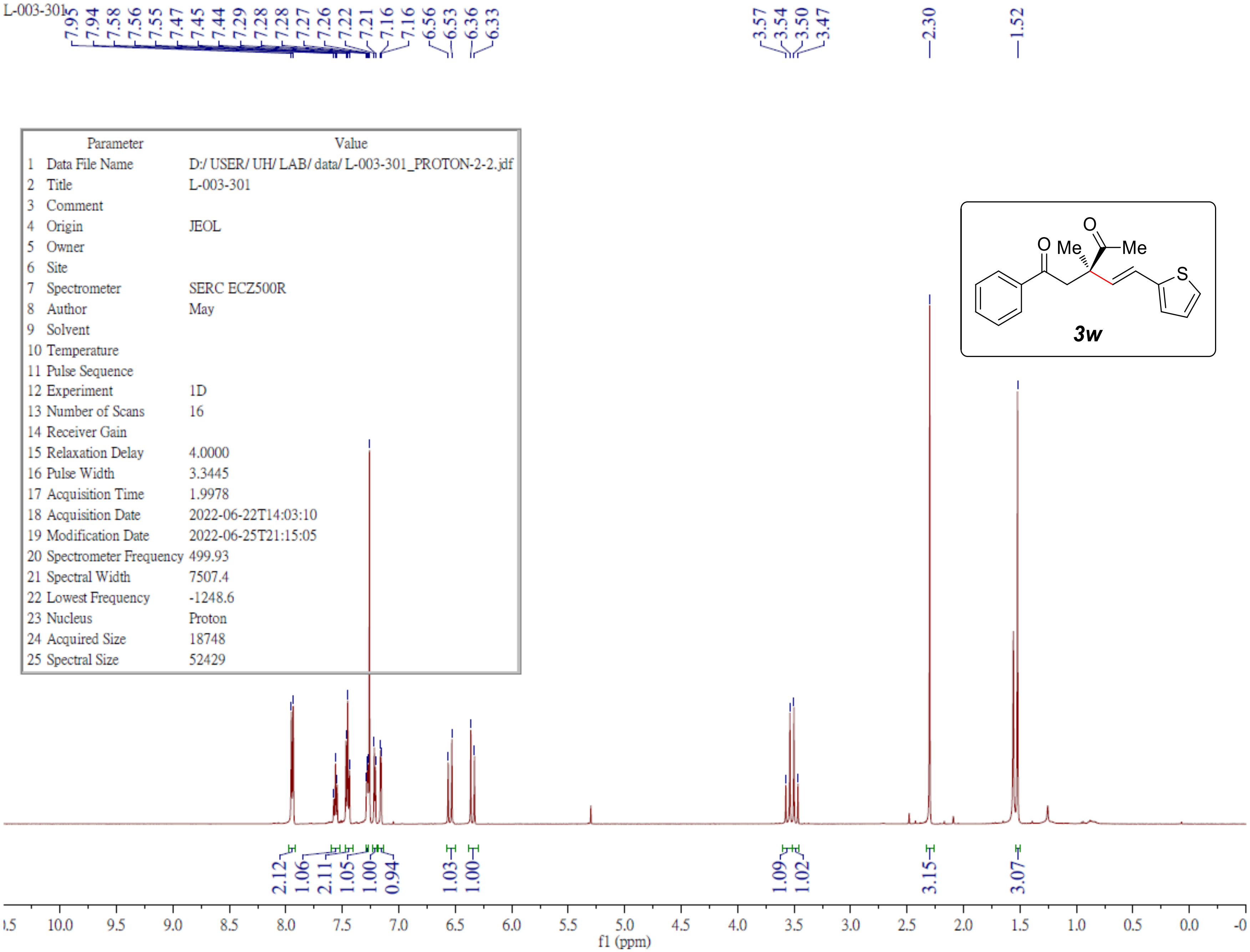

	Parameter	Value
1	Data File Name	D:/ USER/ UH/ LAB/ data/ L-003-301_PROTON-2-2.jdf
2	Title	L-003-301
3	Comment	
4	Origin	JEOL
5	Owner	
6	Site	
7	Spectrometer	SERC ECZ500R
8	Author	May
9	Solvent	
10	Temperature	
11	Pulse Sequence	
12	Experiment	1D
13	Number of Scans	16
14	Receiver Gain	
15	Relaxation Delay	4.0000
16	Pulse Width	3.3445
17	Acquisition Time	1.9978
18	Acquisition Date	2022-06-22T14:03:10
19	Modification Date	2022-06-25T21:15:05
20	Spectrometer Frequency	499.93
21	Spectral Width	7507.4
22	Lowest Frequency	-1248.6
23	Nucleus	Proton
24	Acquired Size	18748
25	Spectral Size	52429

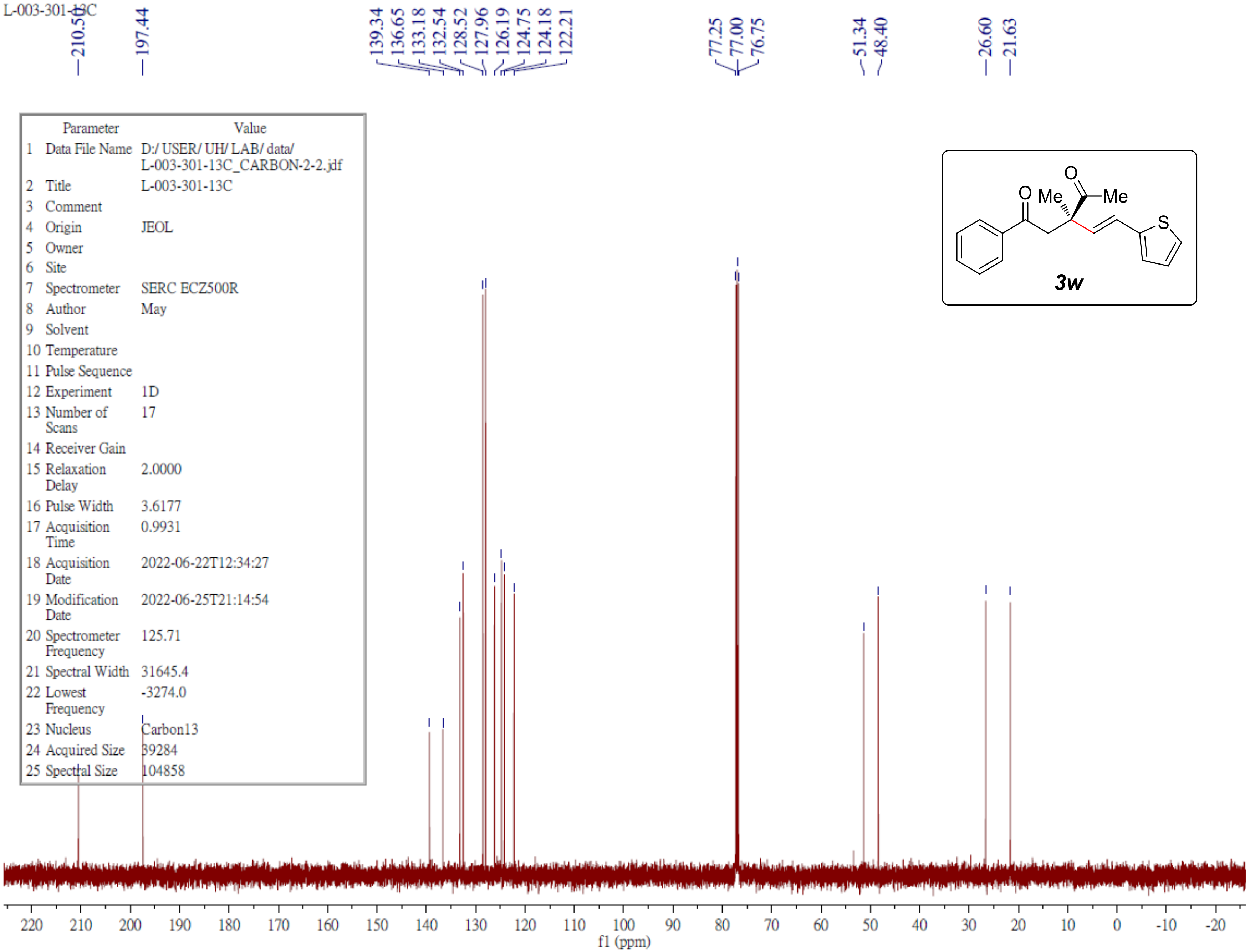

	Parameter	Value
1	Data File Name	D:/ USER/ UH/ LAB/ data/ L-003-301-13C_CARBON-2-2.jdf
2	Title	L-003-301-13C
3	Comment	
4	Origin	JEOL
5	Owner	
6	Site	
7	Spectrometer	SERC ECZ500R
8	Author	May
9	Solvent	
10	Temperature	
11	Pulse Sequence	
12	Experiment	1D
13	Number of Scans	17
14	Receiver Gain	
15	Relaxation Delay	2.0000
16	Pulse Width	3.6177
17	Acquisition Time	0.9931
18	Acquisition Date	2022-06-22T12:34:27
19	Modification Date	2022-06-25T21:14:54
20	Spectrometer Frequency	125.71
21	Spectral Width	31645.4
22	Lowest Frequency	-3274.0
23	Nucleus	Carbon13
24	Acquired Size	39284
25	Spectral Size	104858

L-003-280-13C

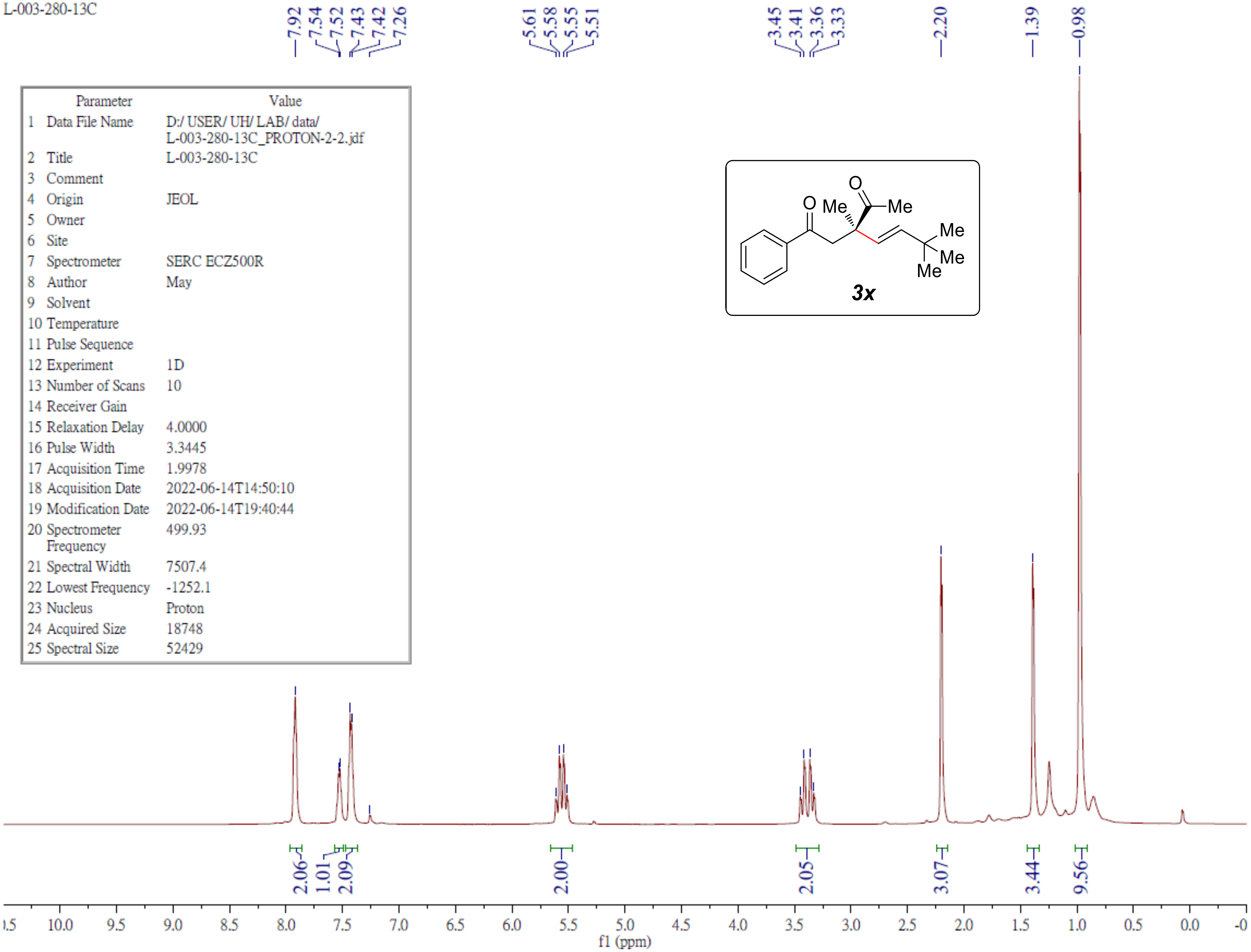

	Parameter	Value
1	Data File Name	D:/ USER/ UH/ LAB/ data/ L-003-280-13C_PROTON-2-2.jdf
2	Title	L-003-280-13C
3	Comment	
4	Origin	JEOL
5	Owner	
6	Site	
7	Spectrometer	SERC ECZ500R
8	Author	May
9	Solvent	
10	Temperature	
11	Pulse Sequence	
12	Experiment	1D
13	Number of Scans	10
14	Receiver Gain	
15	Relaxation Delay	4.0000
16	Pulse Width	3.3445
17	Acquisition Time	1.9978
18	Acquisition Date	2022-06-14T14:50:10
19	Modification Date	2022-06-14T19:40:44
20	Spectrometer Frequency	499.93
21	Spectral Width	7507.4
22	Lowest Frequency	-1252.1
23	Nucleus	Proton
24	Acquired Size	18748
25	Spectral Size	52429

3x

L-003-280-13C

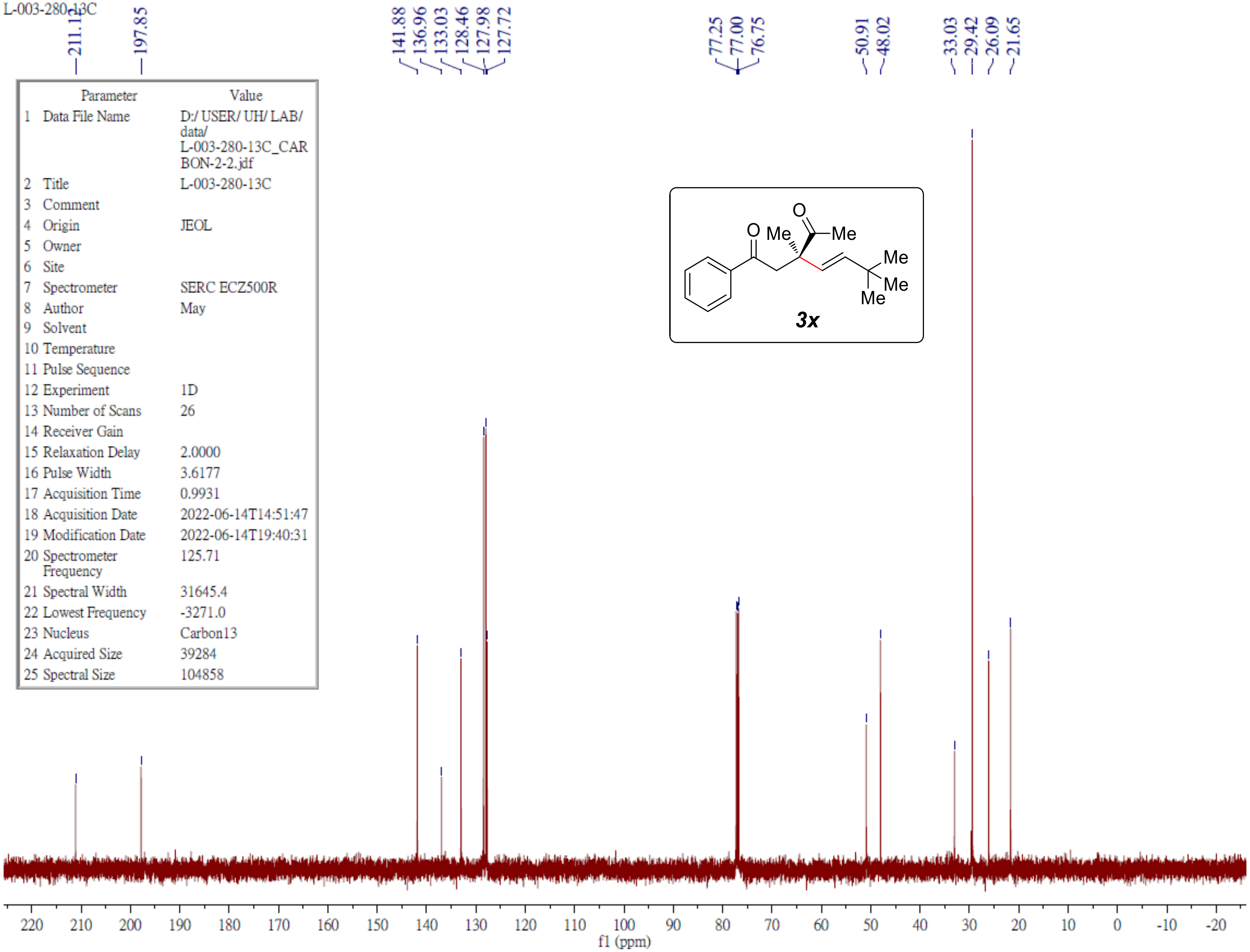

	Parameter	Value
1	Data File Name	D:/ USER/ UH/ LAB/ data/ L-003-280-13C_CARBON-2-2.jdf
2	Title	L-003-280-13C
3	Comment	
4	Origin	JEOL
5	Owner	
6	Site	
7	Spectrometer	SERC ECZ500R
8	Author	May
9	Solvent	
10	Temperature	
11	Pulse Sequence	
12	Experiment	1D
13	Number of Scans	26
14	Receiver Gain	
15	Relaxation Delay	2.0000
16	Pulse Width	3.6177
17	Acquisition Time	0.9931
18	Acquisition Date	2022-06-14T14:51:47
19	Modification Date	2022-06-14T19:40:31
20	Spectrometer Frequency	125.71
21	Spectral Width	31645.4
22	Lowest Frequency	-3271.0
23	Nucleus	Carbon13
24	Acquired Size	39284
25	Spectral Size	104858

L-002-248

Parameter	Value
1 Data File Name	D:/ USER/ UH/ LAB/ data/ L-002-248_PROTON-2-2.jdf
2 Title	L-002-248
3 Comment	
4 Origin	JEOL
5 Owner	
6 Site	
7 Spectrometer	SERC ECZ500R
8 Author	May
9 Solvent	
10 Temperature	
11 Pulse Sequence	
12 Experiment	1D
13 Number of Scans	16
14 Receiver Gain	
15 Relaxation Delay	4.0000
16 Pulse Width	3.3445
17 Acquisition Time	1.9978
18 Acquisition Date	2021-06-28T14:18:22
19 Modification Date	2021-06-28T19:40:16
20 Spectrometer Frequency	499.93
21 Spectral Width	7507.4
22 Lowest Frequency	-1248.1
23 Nucleus	Proton
24 Acquired Size	18748
25 Spectral Size	52429

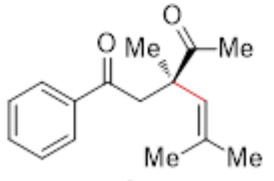

3y

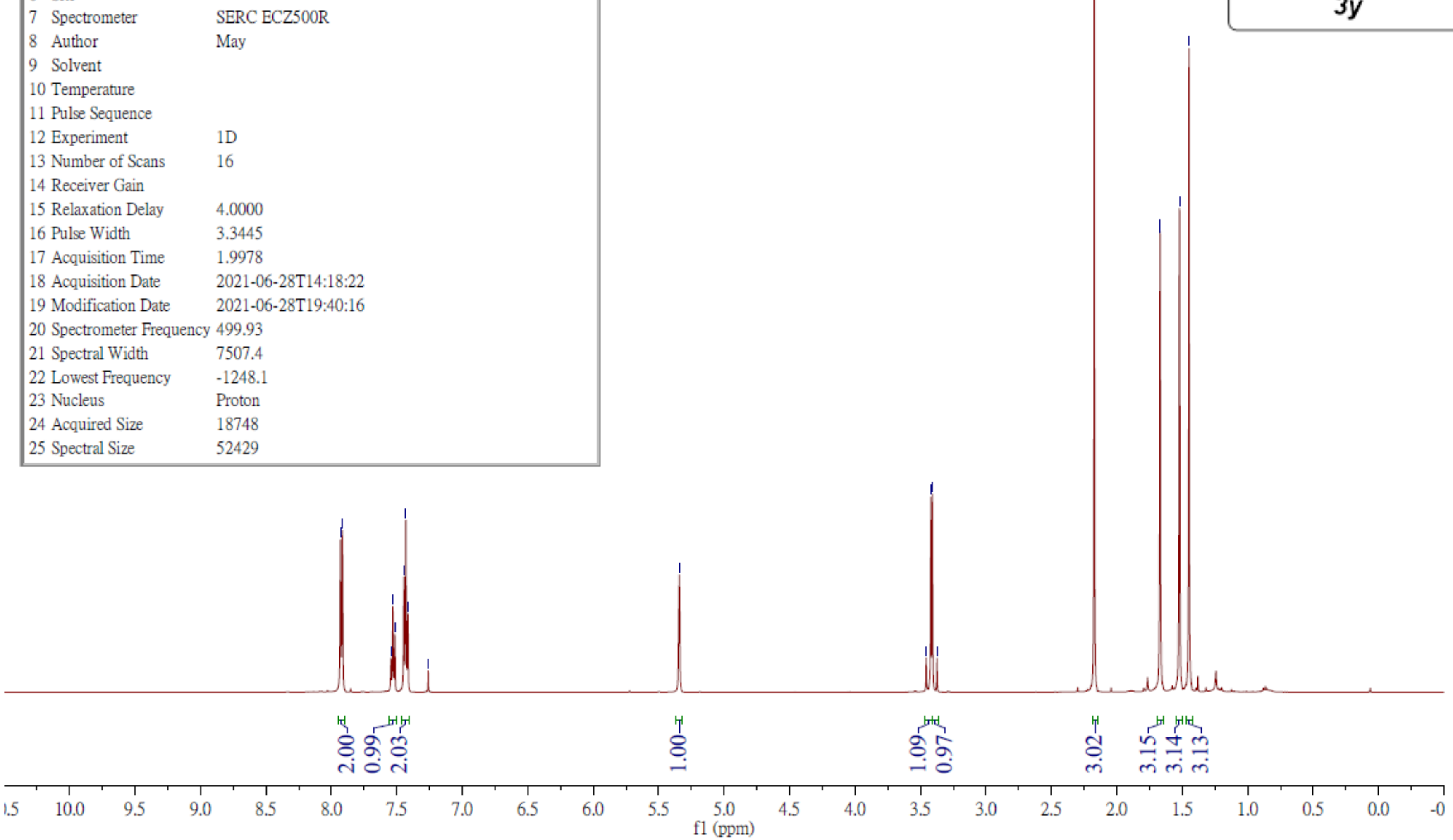

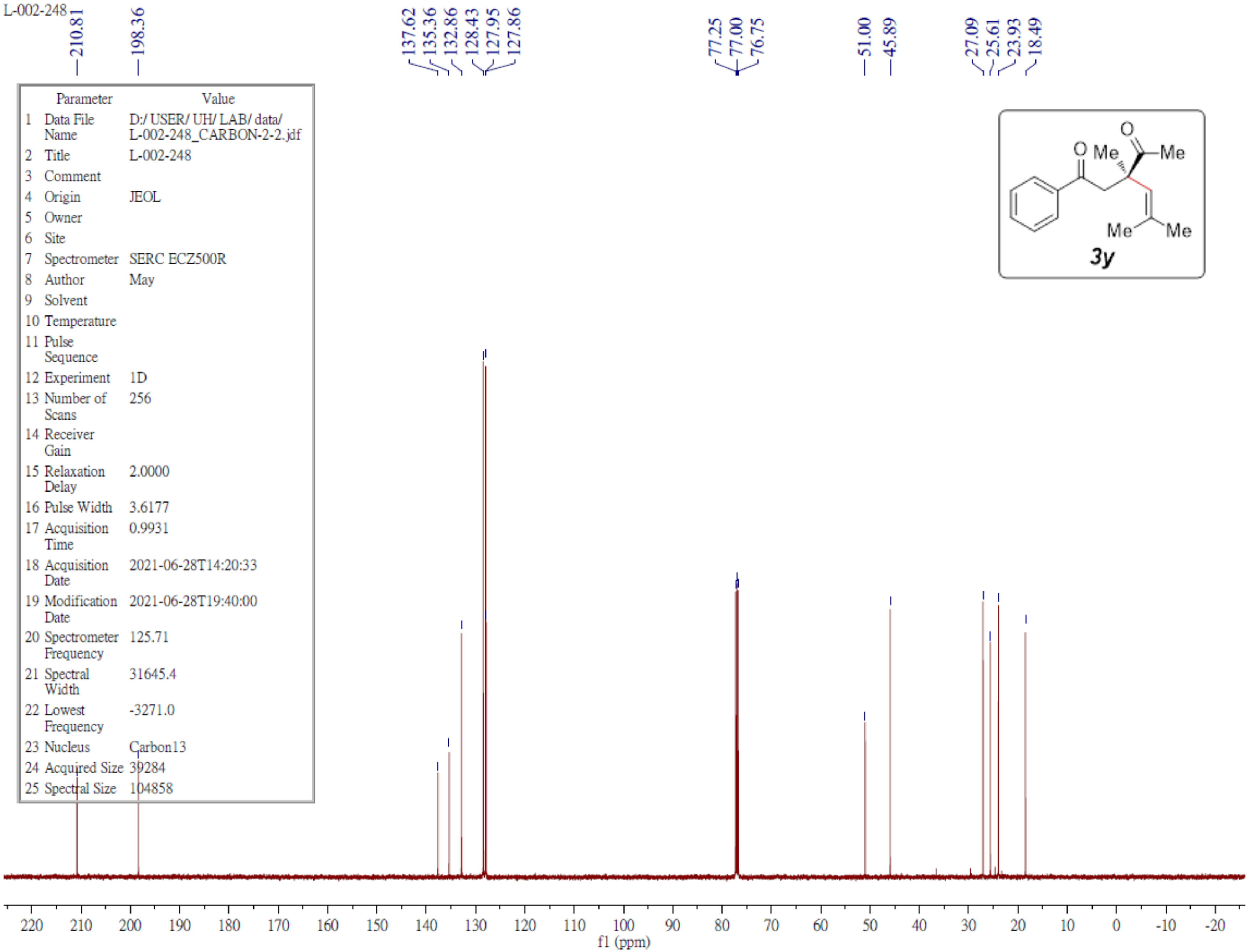

	Parameter	Value
1	Data File Name	D:/ USER/ UH/ LAB/ data/ L-002-248_CARBON-2-2.jdf
2	Title	L-002-248
3	Comment	
4	Origin	JEOL
5	Owner	
6	Site	
7	Spectrometer	SERC ECZ500R
8	Author	May
9	Solvent	
10	Temperature	
11	Pulse Sequence	
12	Experiment	1D
13	Number of Scans	256
14	Receiver Gain	
15	Relaxation Delay	2.0000
16	Pulse Width	3.6177
17	Acquisition Time	0.9931
18	Acquisition Date	2021-06-28T14:20:33
19	Modification Date	2021-06-28T19:40:00
20	Spectrometer Frequency	125.71
21	Spectral Width	31645.4
22	Lowest Frequency	-3271.0
23	Nucleus	Carbon13
24	Acquired Size	39284
25	Spectral Size	104858

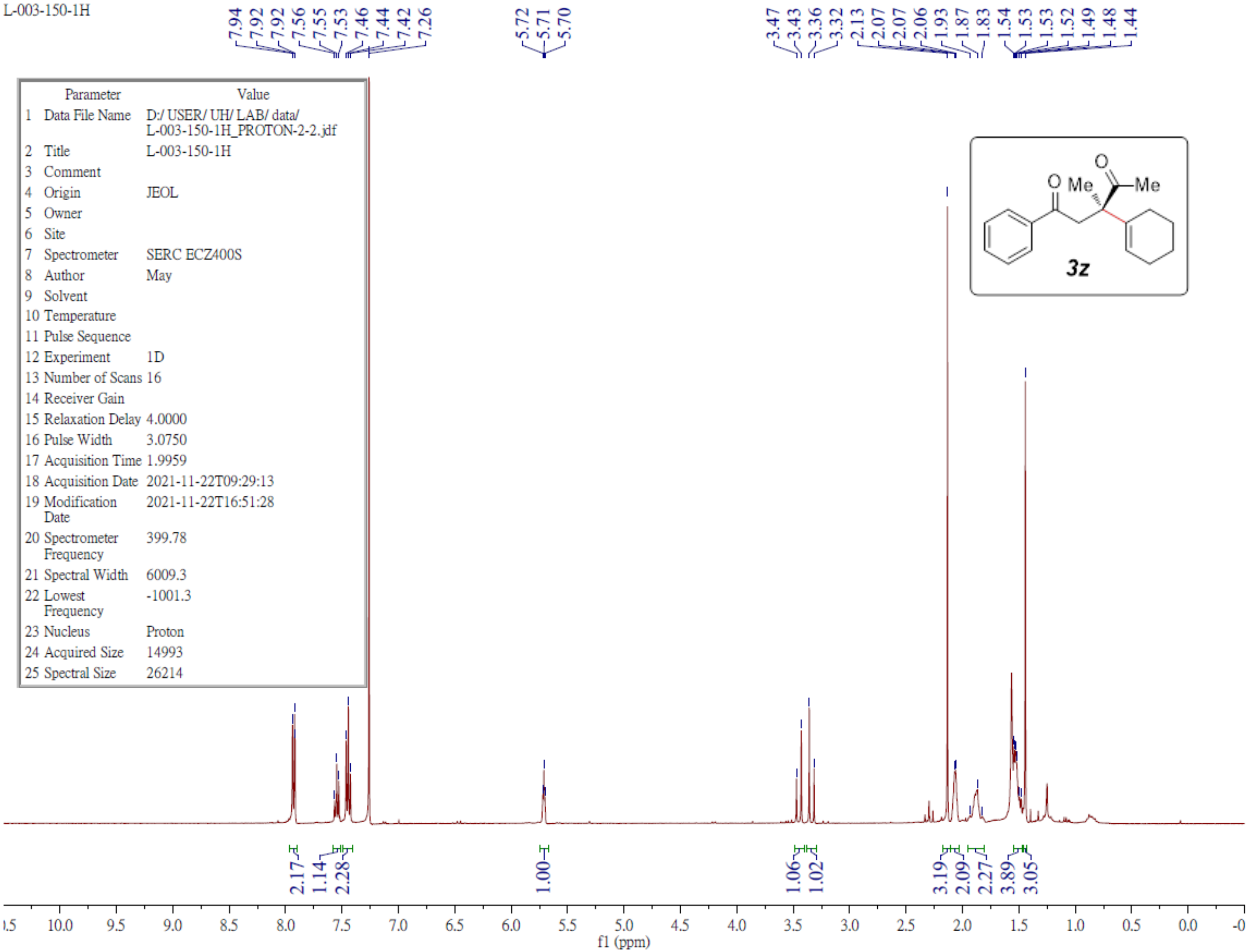

	Parameter	Value
1	Data File Name	D:/ USER/ UH/ LAB/ data/ L-003-150-1H_PROTON-2-2.jdf
2	Title	L-003-150-1H
3	Comment	
4	Origin	JEOL
5	Owner	
6	Site	
7	Spectrometer	SERC ECZ400S
8	Author	May
9	Solvent	
10	Temperature	
11	Pulse Sequence	
12	Experiment	1D
13	Number of Scans	16
14	Receiver Gain	
15	Relaxation Delay	4.0000
16	Pulse Width	3.0750
17	Acquisition Time	1.9959
18	Acquisition Date	2021-11-22T09:29:13
19	Modification Date	2021-11-22T16:51:28
20	Spectrometer Frequency	399.78
21	Spectral Width	6009.3
22	Lowest Frequency	-1001.3
23	Nucleus	Proton
24	Acquired Size	14993
25	Spectral Size	26214

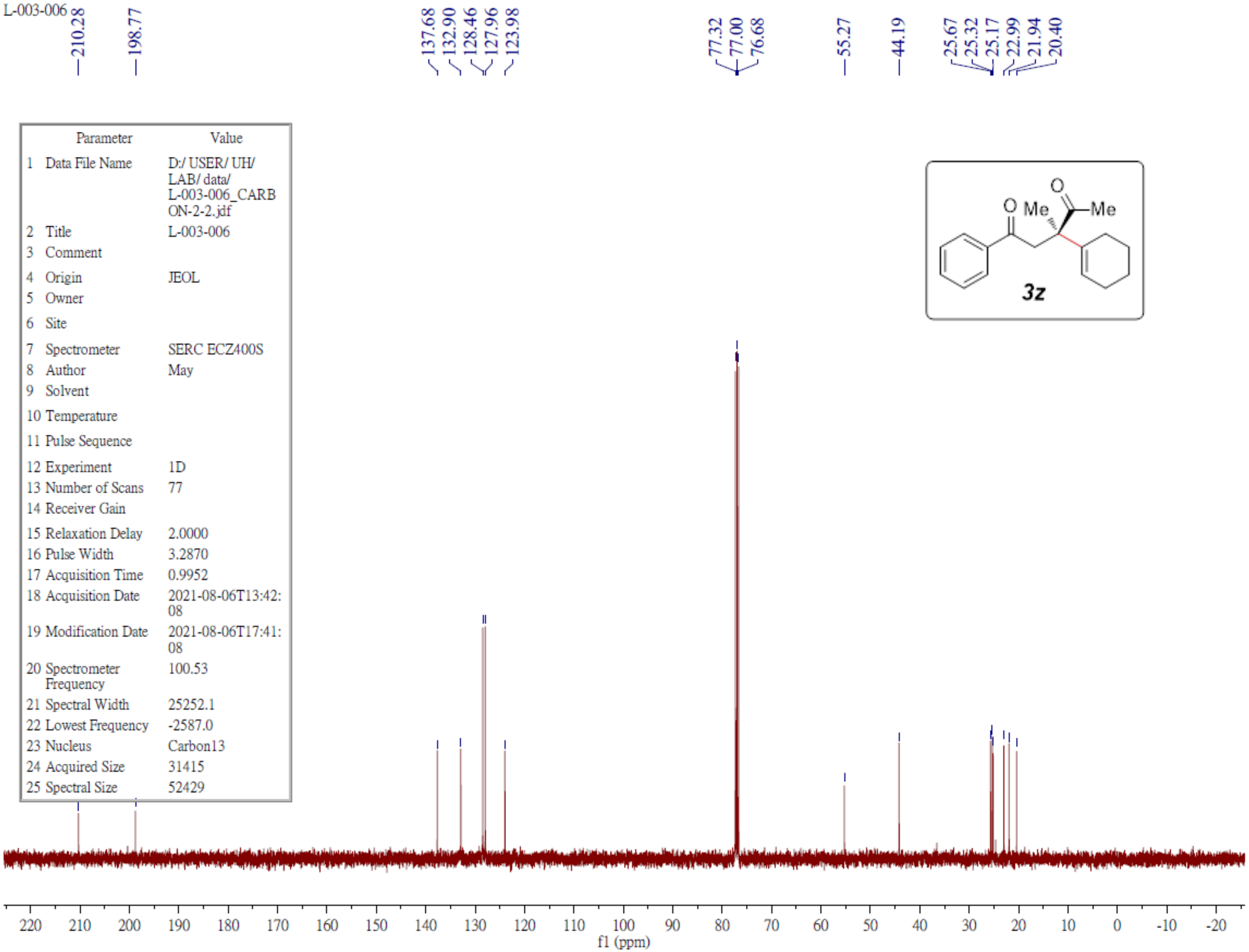

	Parameter	Value
1	Data File Name	D:/ USER/ UH/ LAB/ data/ L-003-006_CARBON-2-2.jdf
2	Title	L-003-006
3	Comment	
4	Origin	JEOL
5	Owner	
6	Site	
7	Spectrometer	SERC ECZ400S
8	Author	May
9	Solvent	
10	Temperature	
11	Pulse Sequence	
12	Experiment	1D
13	Number of Scans	77
14	Receiver Gain	
15	Relaxation Delay	2.0000
16	Pulse Width	3.2870
17	Acquisition Time	0.9952
18	Acquisition Date	2021-08-06T13:42:08
19	Modification Date	2021-08-06T17:41:08
20	Spectrometer Frequency	100.53
21	Spectral Width	25252.1
22	Lowest Frequency	-2587.0
23	Nucleus	Carbon13
24	Acquired Size	31415
25	Spectral Size	52429

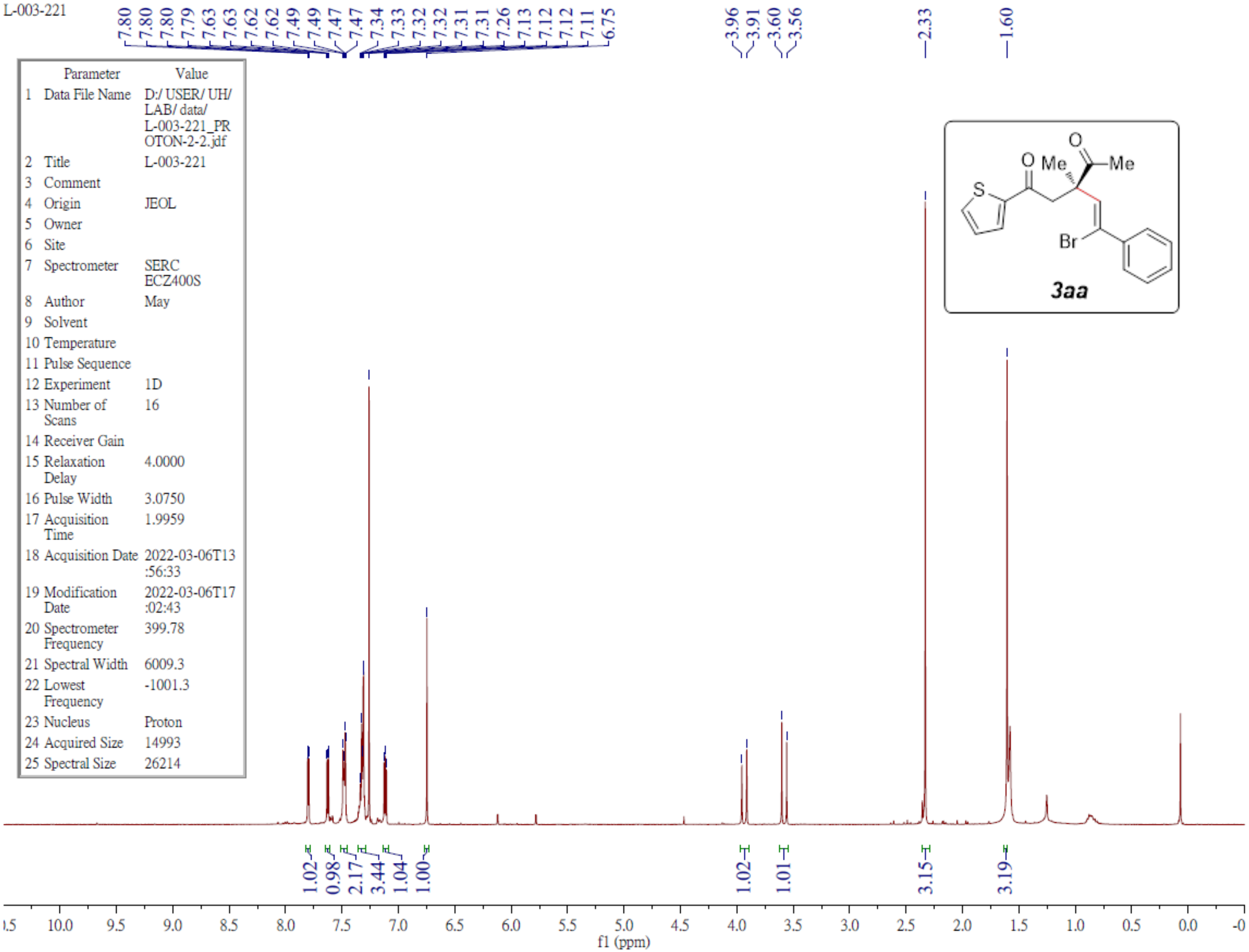
L-003-221
7.80 7.80 7.80 7.79 7.63 7.63 7.62 7.62 7.49 7.49 7.47 7.47 7.34 7.33 7.32 7.32 7.31 7.31 7.26 7.13 7.12 7.12 7.11 6.75
3.96 3.91 3.60 3.56
2.33
1.60
Parameter Value
1 Data File Name D:/ USER/ UH/ LAB/ data/ L-003-221_PROTON-2-2.jdf
2 Title L-003-221
3 Comment
4 Origin JEOL
5 Owner
6 Site
7 Spectrometer SERC ECZ400S
8 Author May
9 Solvent
10 Temperature
11 Pulse Sequence
12 Experiment 1D
13 Number of Scans 16
14 Receiver Gain
15 Relaxation Delay 4.0000
16 Pulse Width 3.0750
17 Acquisition Time 1.9959
18 Acquisition Date 2022-03-06T13:56:33
19 Modification Date 2022-03-06T17:02:43
20 Spectrometer Frequency 399.78
21 Spectral Width 6009.3
22 Lowest Frequency -1001.3
23 Nucleus Proton
24 Acquired Size 14993
25 Spectral Size 26214
O
O
Me
Me
S
Br
3aa
1.02 0.98 2.17 3.44 1.04 1.00
1.02 1.01
3.15
3.19
10.0 9.5 9.0 8.5 8.0 7.5 7.0 6.5 6.0 5.5 5.0 4.5 4.0 3.5 3.0 2.5 2.0 1.5 1.0 0.5 0.0
f1 (ppm)

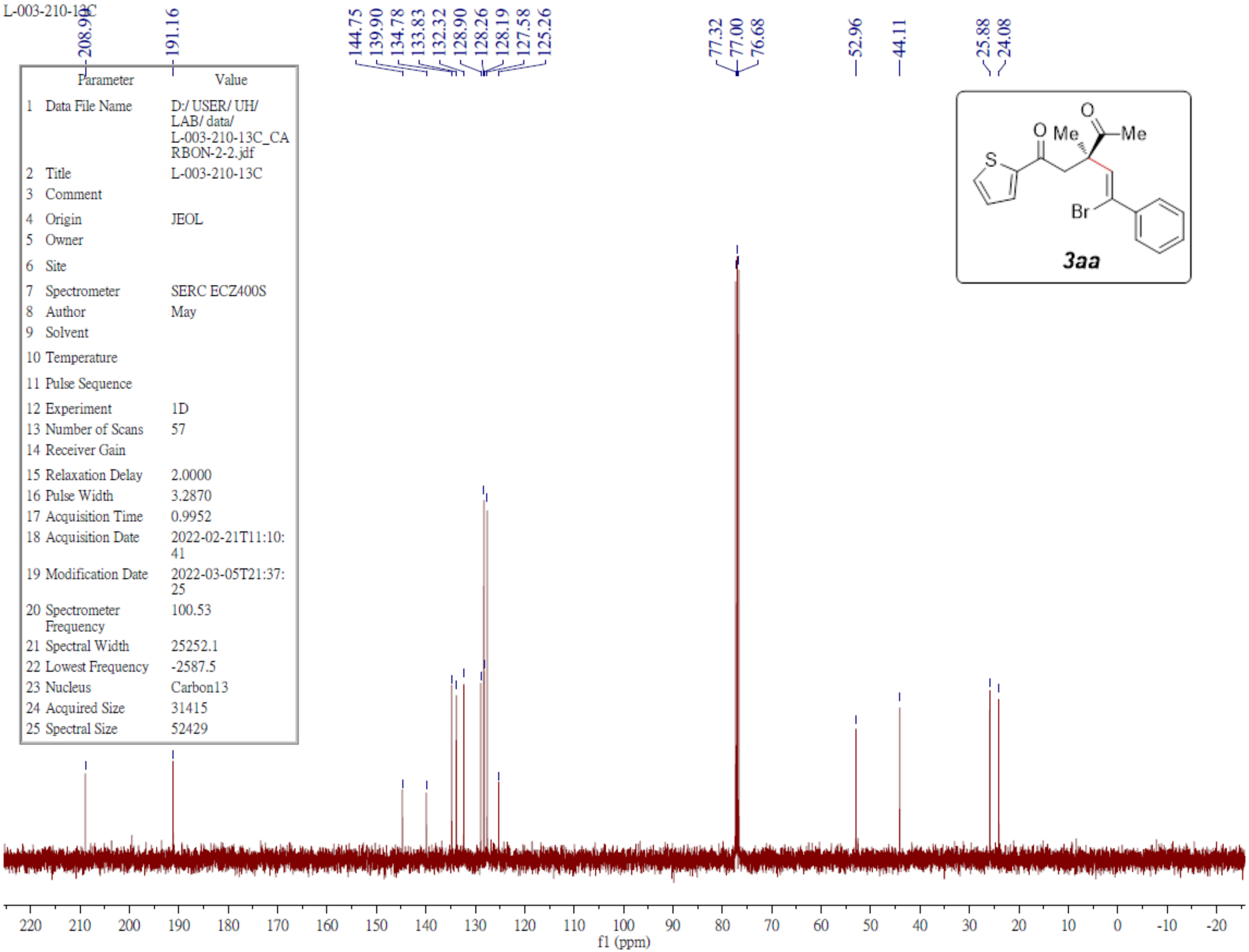
L-003-210-13C
208.98
191.16
144.75
139.90
134.78
133.83
132.32
128.90
128.26
128.19
127.58
125.26
77.32
77.00
76.68
52.96
44.11
25.88
24.08
Parameter Value
1 Data File Name D:/ USER/ UH/ LAB/ data/ L-003-210-13C_CARBON-2-2.jdf
2 Title L-003-210-13C
3 Comment
4 Origin JEOL
5 Owner
6 Site
7 Spectrometer SERC ECZ400S
8 Author May
9 Solvent
10 Temperature
11 Pulse Sequence
12 Experiment 1D
13 Number of Scans 57
14 Receiver Gain
15 Relaxation Delay 2.0000
16 Pulse Width 3.2870
17 Acquisition Time 0.9952
18 Acquisition Date 2022-02-21T11:10:41
19 Modification Date 2022-03-05T21:37:25
20 Spectrometer Frequency 100.53
21 Spectral Width 25252.1
22 Lowest Frequency -2587.5
23 Nucleus Carbon13
24 Acquired Size 31415
25 Spectral Size 52429
O
Me
O
Me
S
Br
3aa
220 210 200 190 180 170 160 150 140 130 120 110 100 90 80 70 60 50 40 30 20 10 0 -10 -20
f1 (ppm)

L-002-118

	Parameter	Value
1	Data File Name	D:/ USER/ UH/ LAB/ data/ L-002-118_PROTON-1-2.jdf
2	Title	L-002-118
3	Comment	
4	Origin	JEOL
5	Owner	
6	Site	
7	Spectrometer	SERC400
8	Author	May
9	Solvent	
10	Temperature	
11	Pulse Sequence	
12	Experiment	1D
13	Number of Scans	16
14	Receiver Gain	
15	Relaxation Delay	4.0000
16	Pulse Width	6.4100
17	Acquisition Time	2.1837
18	Acquisition Date	2021-03-19T11:15:03
19	Modification Date	2021-03-19T20:44:03
20	Spectrometer Frequency	399.78
21	Spectral Width	6001.9
22	Lowest Frequency	-999.2
23	Nucleus	1H
24	Acquired Size	16384
25	Spectral Size	13107

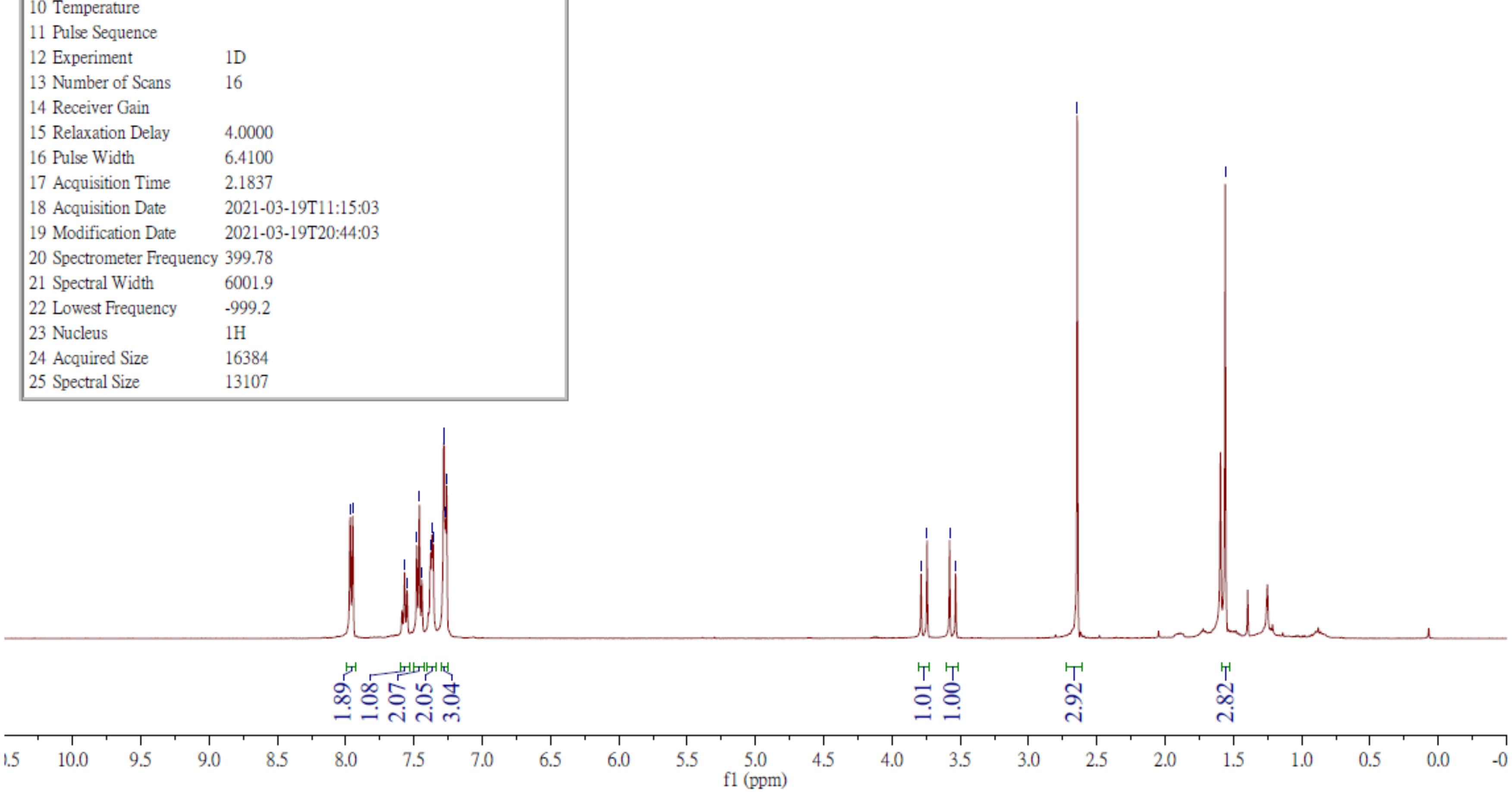

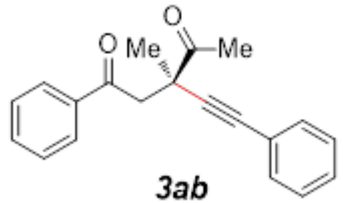

3ab

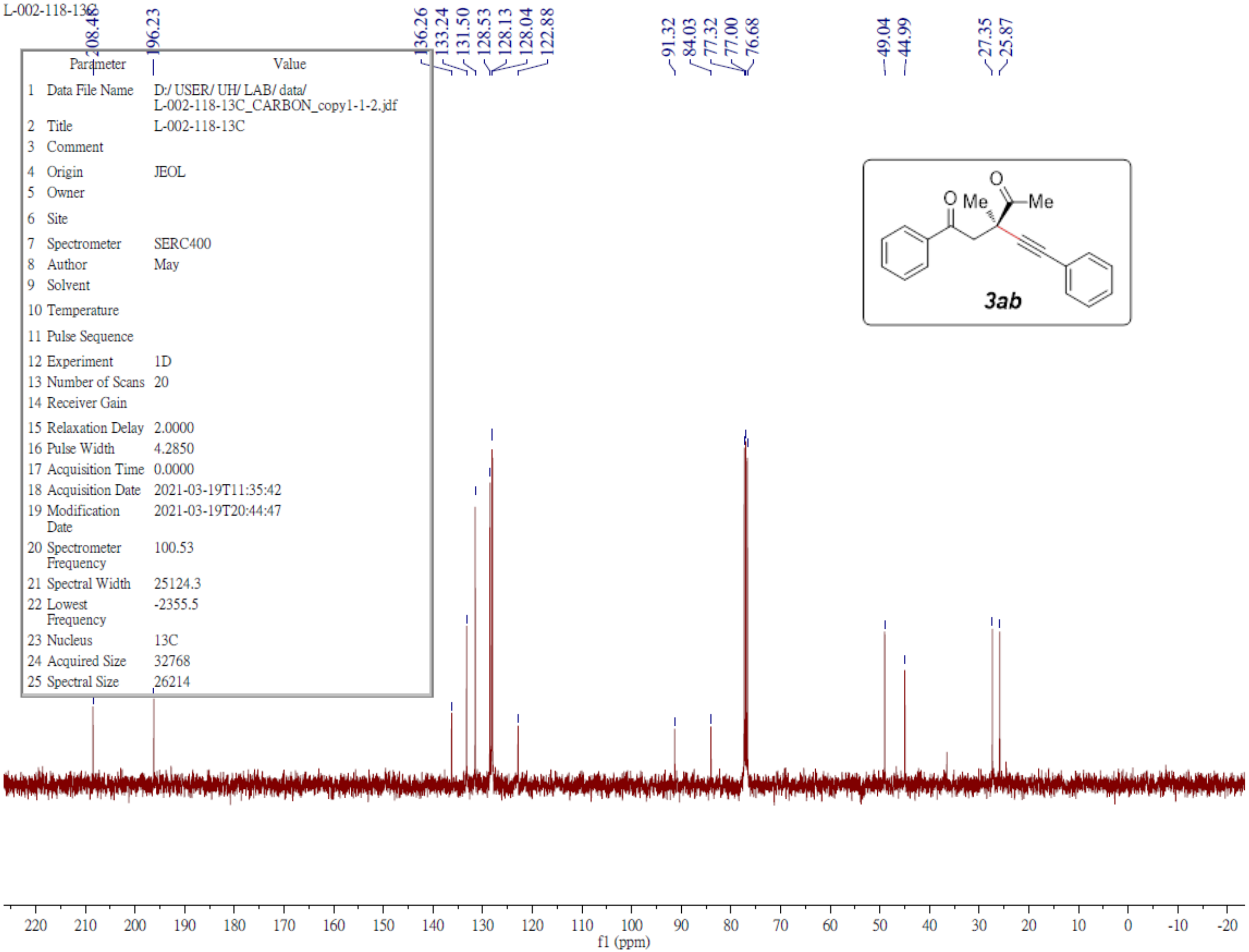
L-002-118-13C
208.48
196.23
136.26
133.24
131.50
128.53
128.13
128.04
122.88
91.32
84.03
77.32
77.00
76.68
49.04
44.99
27.35
25.87
Parameter Value
1 Data File Name D:/ USER/ UH/ LAB/ data/ L-002-118-13C_CARBON_copy1-1-2.jdf
2 Title L-002-118-13C
3 Comment
4 Origin JEOL
5 Owner
6 Site
7 Spectrometer SERC400
8 Author May
9 Solvent
10 Temperature
11 Pulse Sequence
12 Experiment 1D
13 Number of Scans 20
14 Receiver Gain
15 Relaxation Delay 2.0000
16 Pulse Width 4.2850
17 Acquisition Time 0.0000
18 Acquisition Date 2021-03-19T11:35:42
19 Modification Date 2021-03-19T20:44:47
20 Spectrometer Frequency 100.53
21 Spectral Width 25124.3
22 Lowest Frequency -2355.5
23 Nucleus 13C
24 Acquired Size 32768
25 Spectral Size 26214
O
Me
O
Me
3ab
220 210 200 190 180 170 160 150 140 130 120 110 100 90 80 70 60 50 40 30 20 10 0 -10 -20
f1 (ppm)

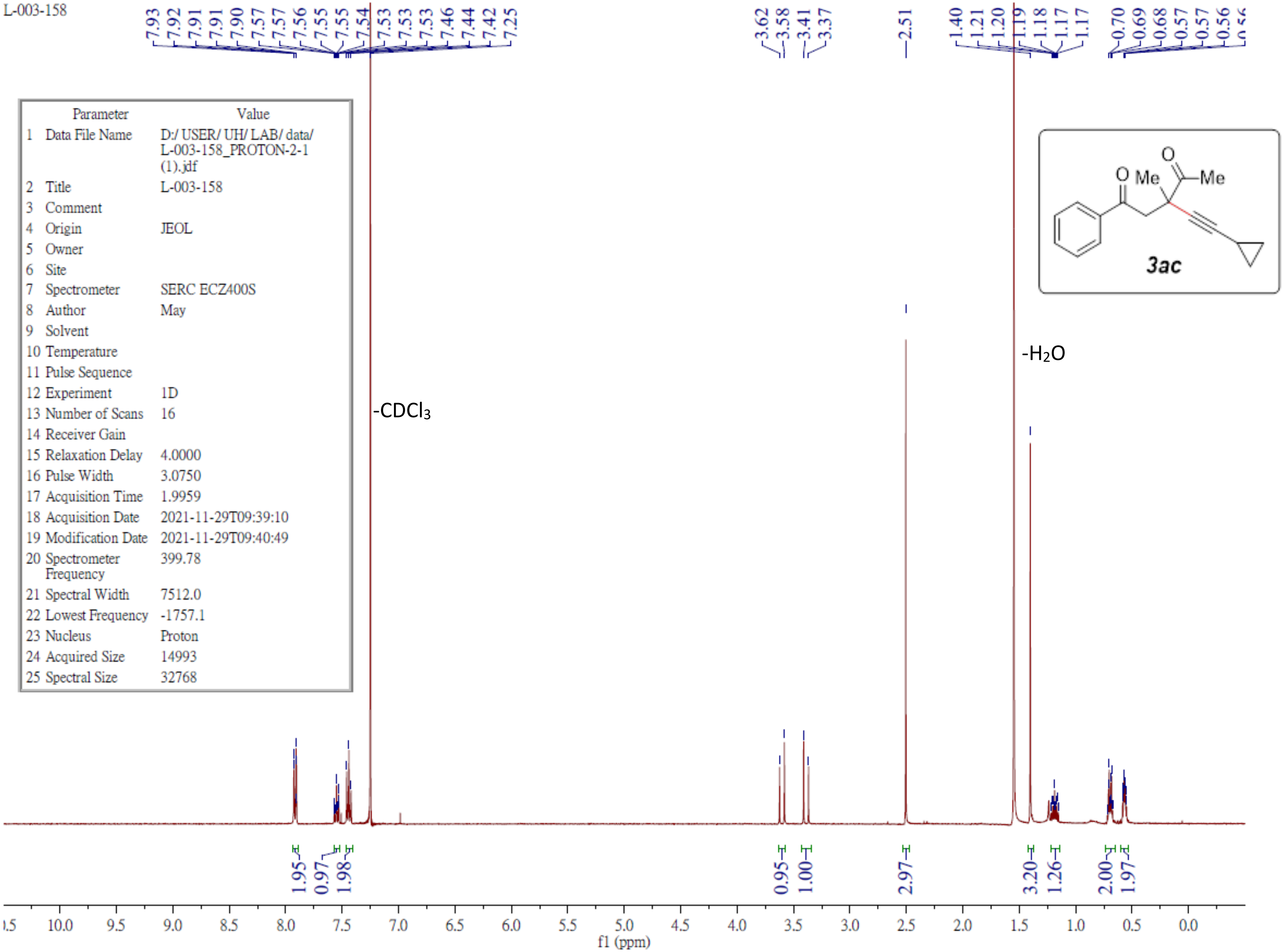

	Parameter	Value
1	Data File Name	D:/ USER/ UH/ LAB/ data/ L-003-158_PROTON-2-1 (1).jdf
2	Title	L-003-158
3	Comment	
4	Origin	JEOL
5	Owner	
6	Site	
7	Spectrometer	SERC ECZ400S
8	Author	May
9	Solvent	
10	Temperature	
11	Pulse Sequence	
12	Experiment	1D
13	Number of Scans	16
14	Receiver Gain	
15	Relaxation Delay	4.0000
16	Pulse Width	3.0750
17	Acquisition Time	1.9959
18	Acquisition Date	2021-11-29T09:39:10
19	Modification Date	2021-11-29T09:40:49
20	Spectrometer Frequency	399.78
21	Spectral Width	7512.0
22	Lowest Frequency	-1757.1
23	Nucleus	Proton
24	Acquired Size	14993
25	Spectral Size	32768

3ac

L-003-158

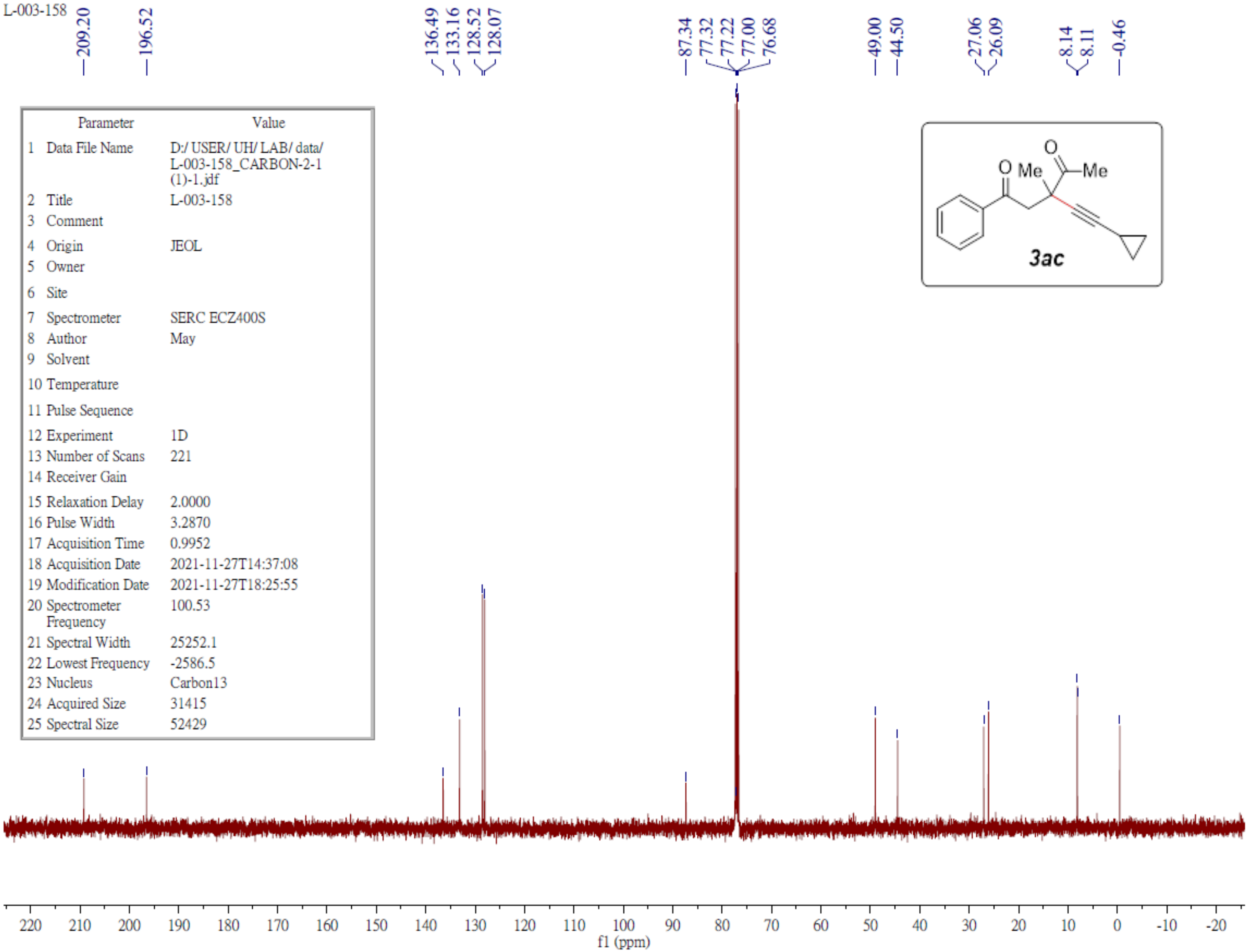

	Parameter	Value
1	Data File Name	D:/ USER/ UH/ LAB/ data/ L-003-158_CARBON-2-1 (1)-1.jdf
2	Title	L-003-158
3	Comment	
4	Origin	JEOL
5	Owner	
6	Site	
7	Spectrometer	SERC ECZ400S
8	Author	May
9	Solvent	
10	Temperature	
11	Pulse Sequence	
12	Experiment	1D
13	Number of Scans	221
14	Receiver Gain	
15	Relaxation Delay	2.0000
16	Pulse Width	3.2870
17	Acquisition Time	0.9952
18	Acquisition Date	2021-11-27T14:37:08
19	Modification Date	2021-11-27T18:25:55
20	Spectrometer Frequency	100.53
21	Spectral Width	25252.1
22	Lowest Frequency	-2586.5
23	Nucleus	Carbon13
24	Acquired Size	31415
25	Spectral Size	52429

3ac

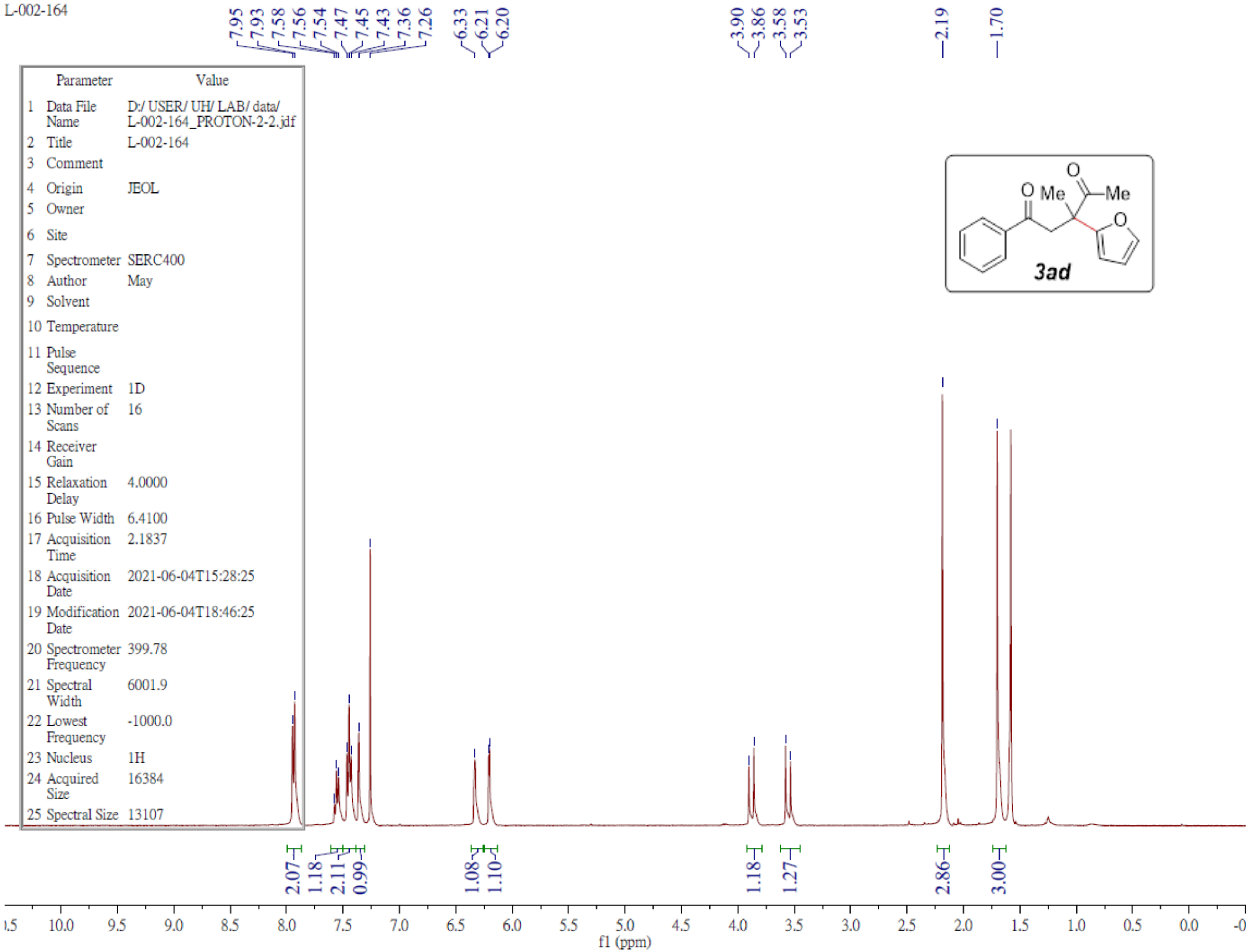
L-002-164
7.95 7.93 7.58 7.56 7.54 7.47 7.45 7.43 7.36 7.26
6.33 6.21 6.20
3.90 3.86 3.58 3.53
2.19
1.70
Parameter Value
1 Data File Name D:/ USER/ UH/ LAB/ data/ L-002-164_PROTON-2-2.jdf
2 Title L-002-164
3 Comment
4 Origin JEOL
5 Owner
6 Site
7 Spectrometer SERC400
8 Author May
9 Solvent
10 Temperature
11 Pulse Sequence
12 Experiment 1D
13 Number of Scans 16
14 Receiver Gain
15 Relaxation Delay 4.0000
16 Pulse Width 6.4100
17 Acquisition Time 2.1837
18 Acquisition Date 2021-06-04T15:28:25
19 Modification Date 2021-06-04T18:46:25
20 Spectrometer Frequency 399.78
21 Spectral Width 6001.9
22 Lowest Frequency -1000.0
23 Nucleus 1H
24 Acquired Size 16384
25 Spectral Size 13107
O
Me
O
Me
O
3ad
2.07 1.18 2.11 0.99
1.08 1.10
1.18 1.27
2.86
3.00
10.0 9.5 9.0 8.5 8.0 7.5 7.0 6.5 6.0 5.5 5.0 4.5 4.0 3.5 3.0 2.5 2.0 1.5 1.0 0.5 0.0
f1 (ppm)

L-002-164-13C

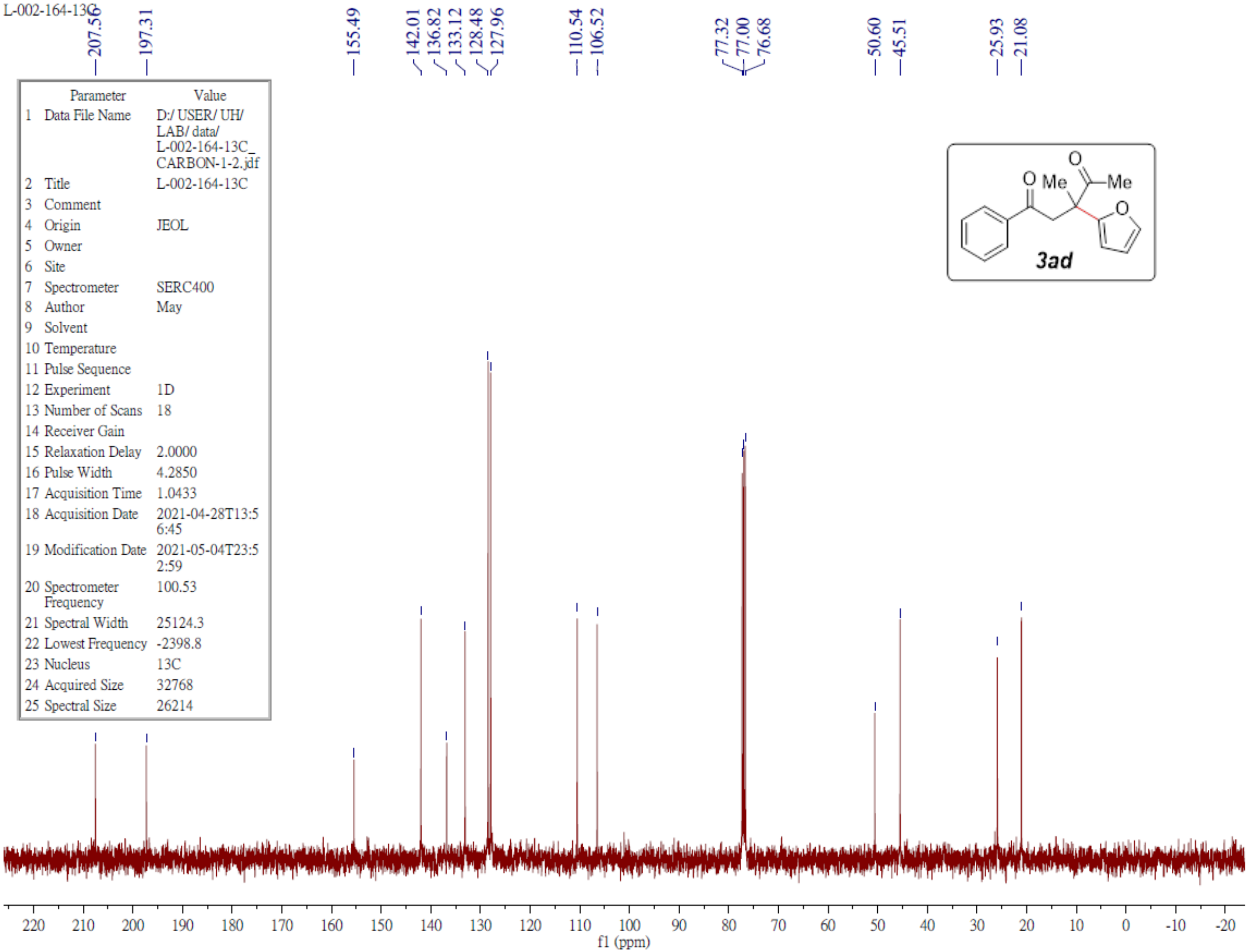

	Parameter	Value
1	Data File Name	D:/ USER/ UH/ LAB/ data/ L-002-164-13C_ CARBON-1-2.jdf
2	Title	L-002-164-13C
3	Comment	
4	Origin	JEOL
5	Owner	
6	Site	
7	Spectrometer	SERC400
8	Author	May
9	Solvent	
10	Temperature	
11	Pulse Sequence	
12	Experiment	1D
13	Number of Scans	18
14	Receiver Gain	
15	Relaxation Delay	2.0000
16	Pulse Width	4.2850
17	Acquisition Time	1.0433
18	Acquisition Date	2021-04-28T13:56:45
19	Modification Date	2021-05-04T23:52:59
20	Spectrometer Frequency	100.53
21	Spectral Width	25124.3
22	Lowest Frequency	-2398.8
23	Nucleus	13C
24	Acquired Size	32768
25	Spectral Size	26214

L-002-165

	Parameter	Value
1	Data File Name	D:/ USER/ UH/ LAB/ data/ L-002-165_PROTON-2-2.jdf
2	Title	L-002-165
3	Comment	
4	Origin	JEOL
5	Owner	
6	Site	
7	Spectrometer	SERC400
8	Author	May
9	Solvent	
10	Temperature	
11	Pulse Sequence	
12	Experiment	1D
13	Number of Scans	16
14	Receiver Gain	
15	Relaxation Delay	4.0000
16	Pulse Width	6.4100
17	Acquisition Time	2.1837
18	Acquisition Date	2021-04-28T12:09:27
19	Modification Date	2021-04-28T23:36:27
20	Spectrometer Frequency	399.78
21	Spectral Width	6001.9
22	Lowest Frequency	-999.2
23	Nucleus	1H
24	Acquired Size	16384
25	Spectral Size	13107

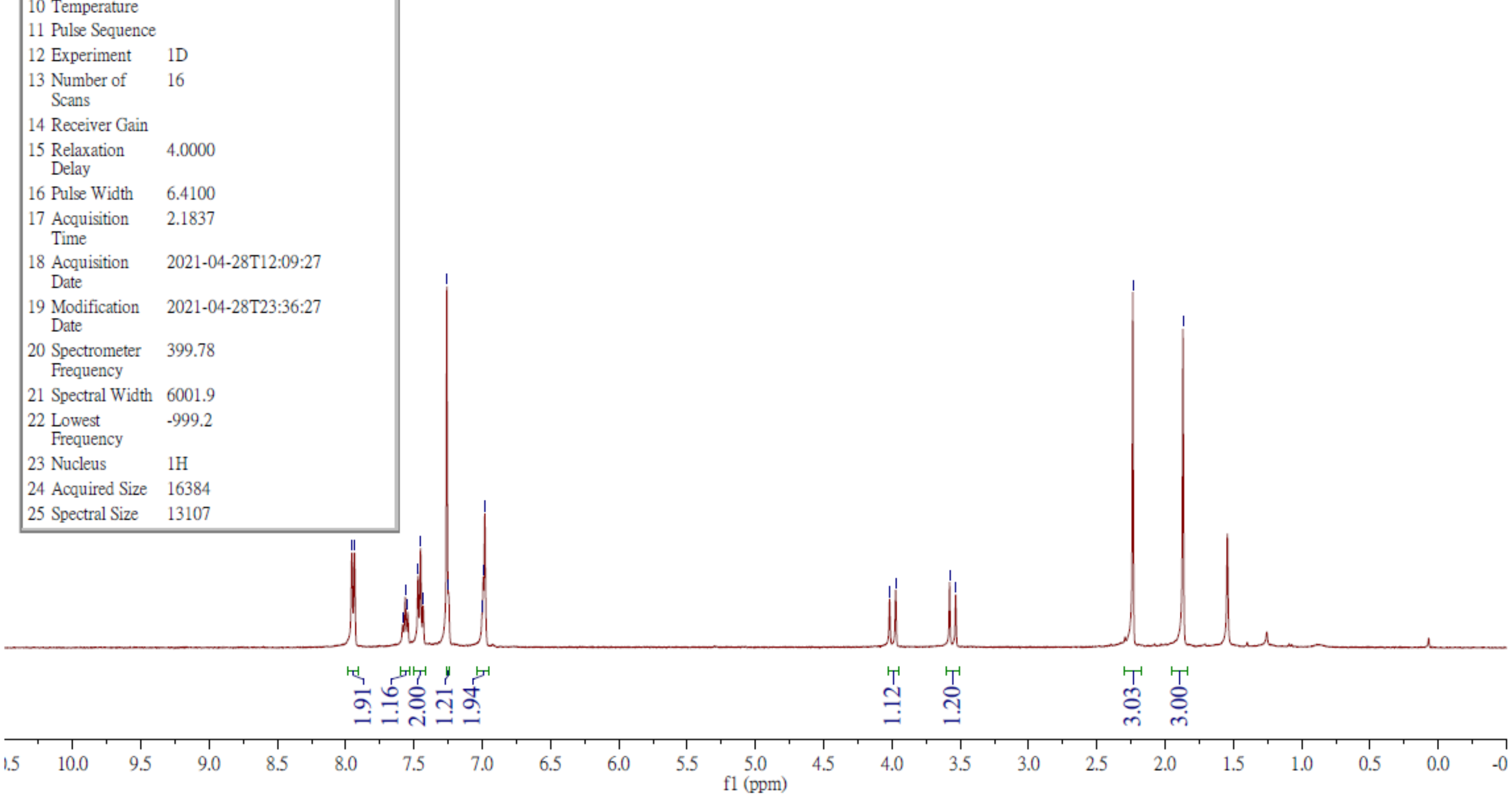

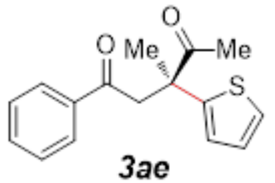

3ae

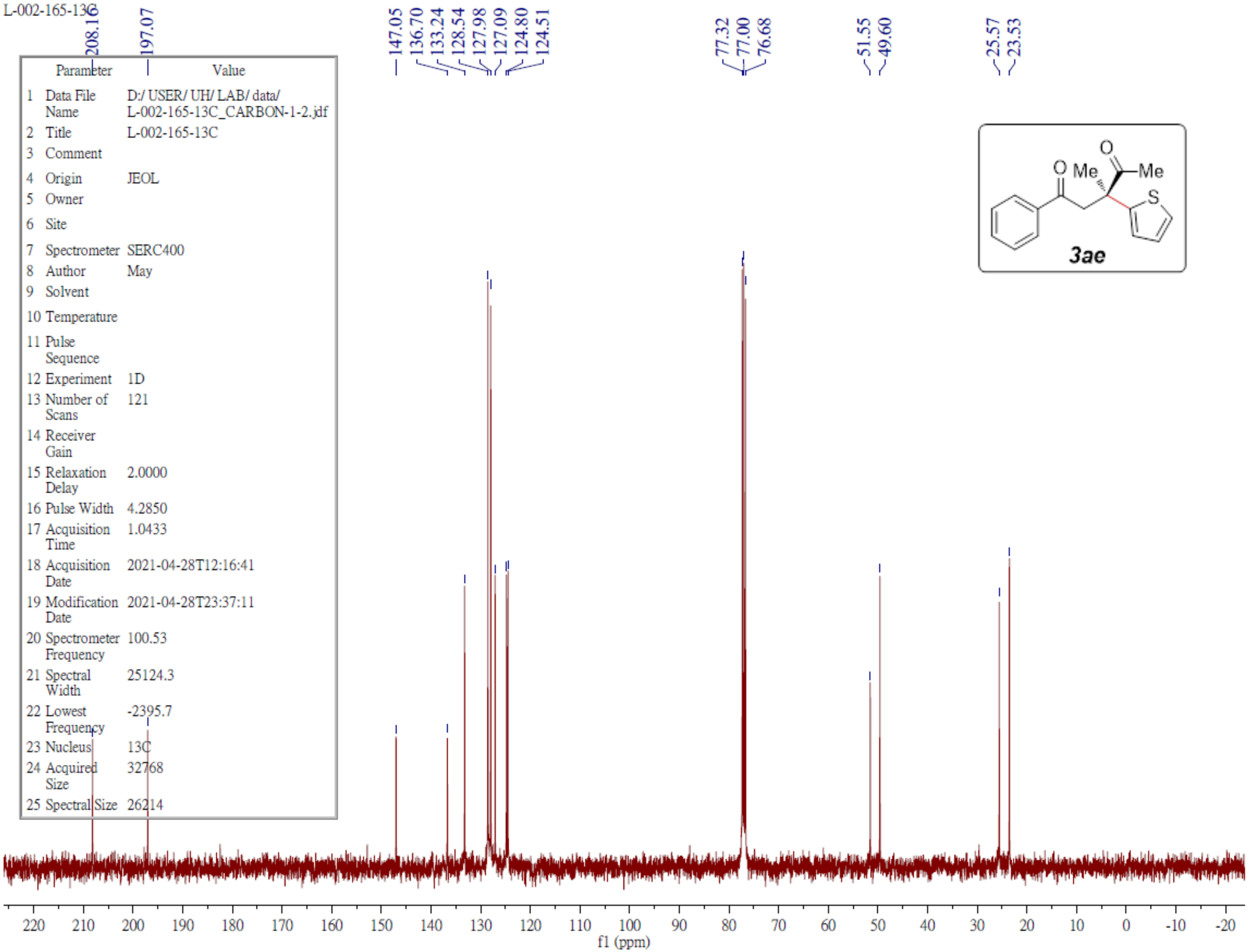
L-002-165-13C
208.16
197.07
147.05
136.70
133.24
128.54
127.98
127.09
124.80
124.51
77.32
77.00
76.68
51.55
49.60
25.57
23.53
Parameter
Value
1 Data File Name D:/ USER/ UH/ LAB/ data/ L-002-165-13C_CARBON-1-2.jdf
2 Title L-002-165-13C
3 Comment
4 Origin JEOL
5 Owner
6 Site
7 Spectrometer SERC400
8 Author May
9 Solvent
10 Temperature
11 Pulse Sequence
12 Experiment 1D
13 Number of Scans 121
14 Receiver Gain
15 Relaxation Delay 2.0000
16 Pulse Width 4.2850
17 Acquisition Time 1.0433
18 Acquisition Date 2021-04-28T12:16:41
19 Modification Date 2021-04-28T23:37:11
20 Spectrometer Frequency 100.53
21 Spectral Width 25124.3
22 Lowest Frequency -2395.7
23 Nucleus 13C
24 Acquired Size 32768
25 Spectral Size 26214
O
Me
O
Me
S
3ae
220 210 200 190 180 170 160 150 140 130 120 110 100 90 80 70 60 50 40 30 20 10 0 -10 -20
f1 (ppm)

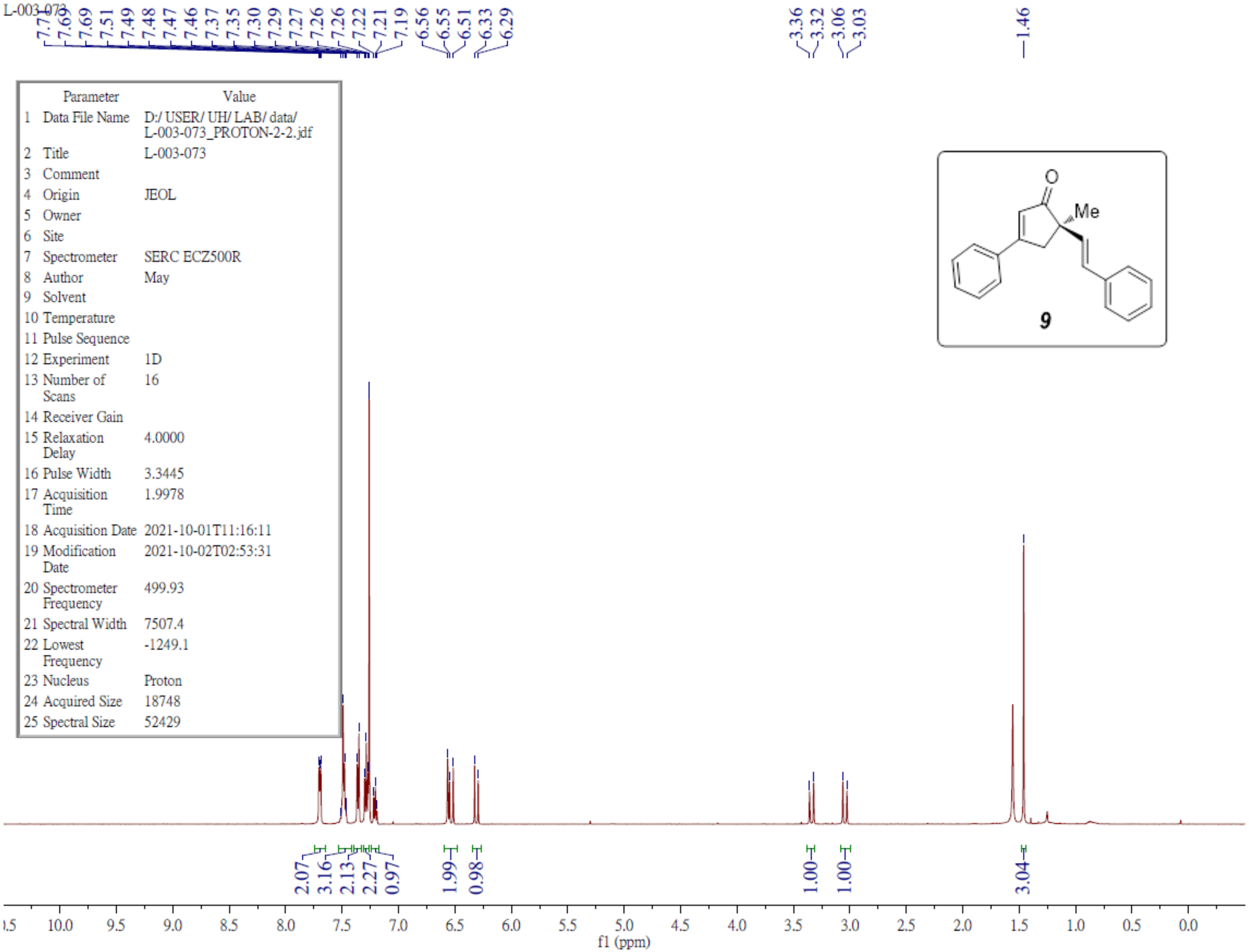

	Parameter	Value
1	Data File Name	D:/ USER/ UH/ LAB/ data/ L-003-073_PROTON-2-2.jdf
2	Title	L-003-073
3	Comment	
4	Origin	JEOL
5	Owner	
6	Site	
7	Spectrometer	SERC ECZ500R
8	Author	May
9	Solvent	
10	Temperature	
11	Pulse Sequence	
12	Experiment	1D
13	Number of Scans	16
14	Receiver Gain	
15	Relaxation Delay	4.0000
16	Pulse Width	3.3445
17	Acquisition Time	1.9978
18	Acquisition Date	2021-10-01T11:16:11
19	Modification Date	2021-10-02T02:53:31
20	Spectrometer Frequency	499.93
21	Spectral Width	7507.4
22	Lowest Frequency	-1249.1
23	Nucleus	Proton
24	Acquired Size	18748
25	Spectral Size	52429

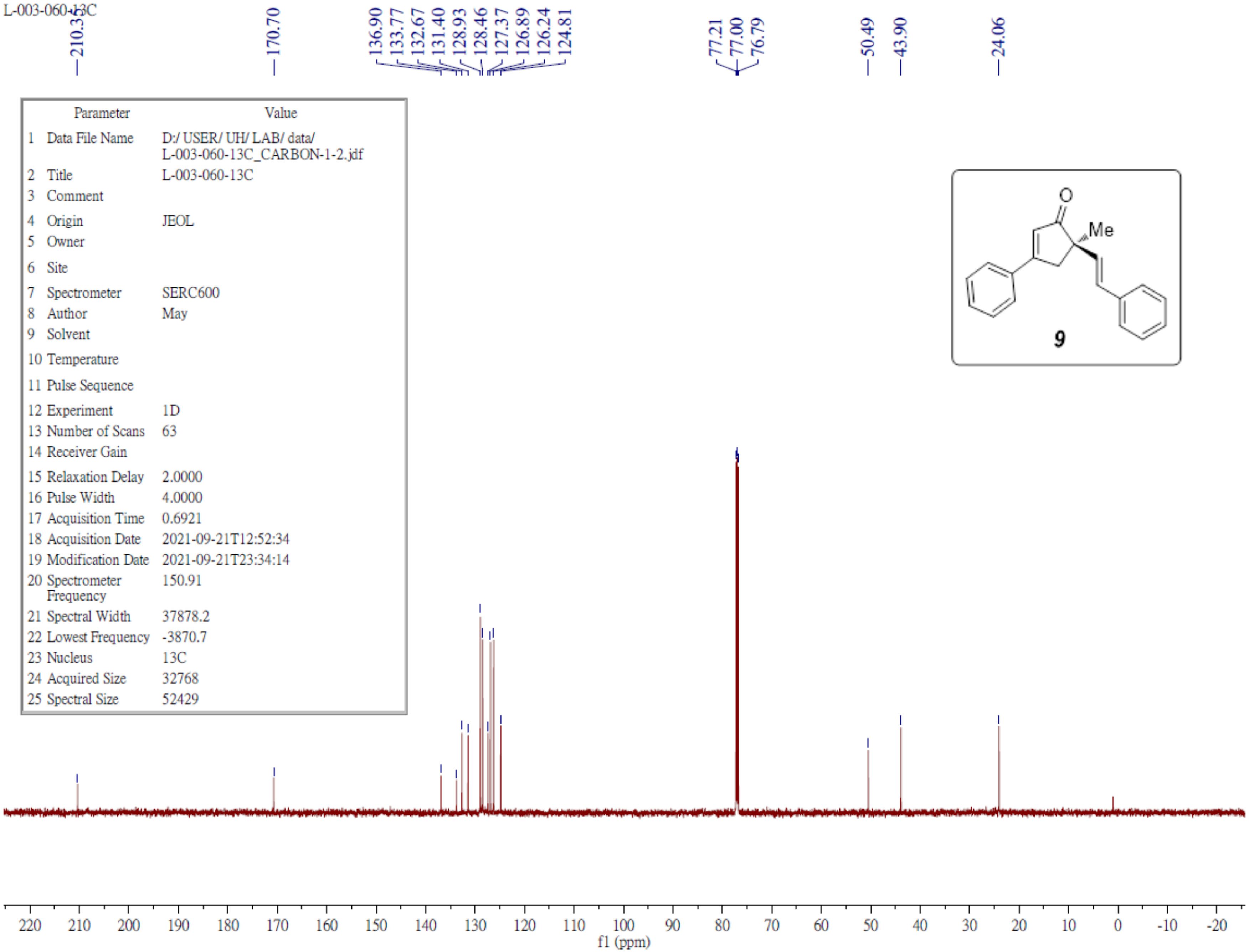

	Parameter	Value
1	Data File Name	D:/ USER/ UH/ LAB/ data/ L-003-060-13C_CARBON-1-2.jdf
2	Title	L-003-060-13C
3	Comment	
4	Origin	JEOL
5	Owner	
6	Site	
7	Spectrometer	SERC600
8	Author	May
9	Solvent	
10	Temperature	
11	Pulse Sequence	
12	Experiment	1D
13	Number of Scans	63
14	Receiver Gain	
15	Relaxation Delay	2.0000
16	Pulse Width	4.0000
17	Acquisition Time	0.6921
18	Acquisition Date	2021-09-21T12:52:34
19	Modification Date	2021-09-21T23:34:14
20	Spectrometer Frequency	150.91
21	Spectral Width	37878.2
22	Lowest Frequency	-3870.7
23	Nucleus	13C
24	Acquired Size	32768
25	Spectral Size	52429

L-003-044

Parameter		Value
1	Data File Name	D:/ USER/ UH/ LAB/ data/ L-003-044_PROTON-2-2.jdf
2	Title	L-003-044
3	Comment	
4	Origin	JEOL
5	Owner	
6	Site	
7	Spectrometer	SERC ECZ400S
8	Author	May
9	Solvent	
10	Temperature	
11	Pulse Sequence	
12	Experiment	1D
13	Number of Scans	16
14	Receiver Gain	
15	Relaxation Delay	4.0000
16	Pulse Width	3.0750
17	Acquisition Time	1.9959
18	Acquisition Date	2021-09-12T16:39:37
19	Modification Date	2021-09-12T19:08:14
20	Spectrometer Frequency	399.78
21	Spectral Width	6009.3
22	Lowest Frequency	-1001.3
23	Nucleus	Proton
24	Acquired Size	14993
25	Spectral Size	26214

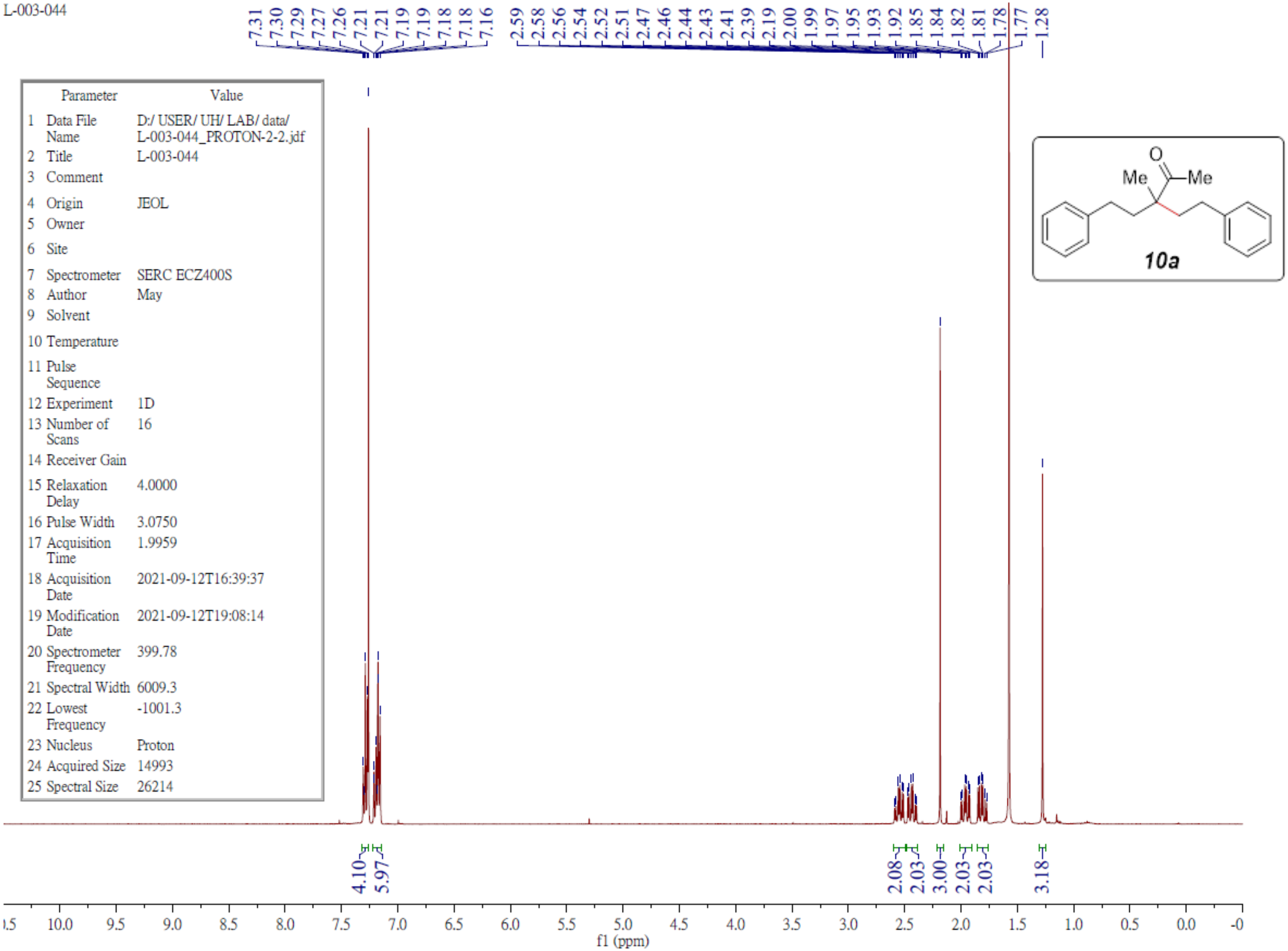

10a

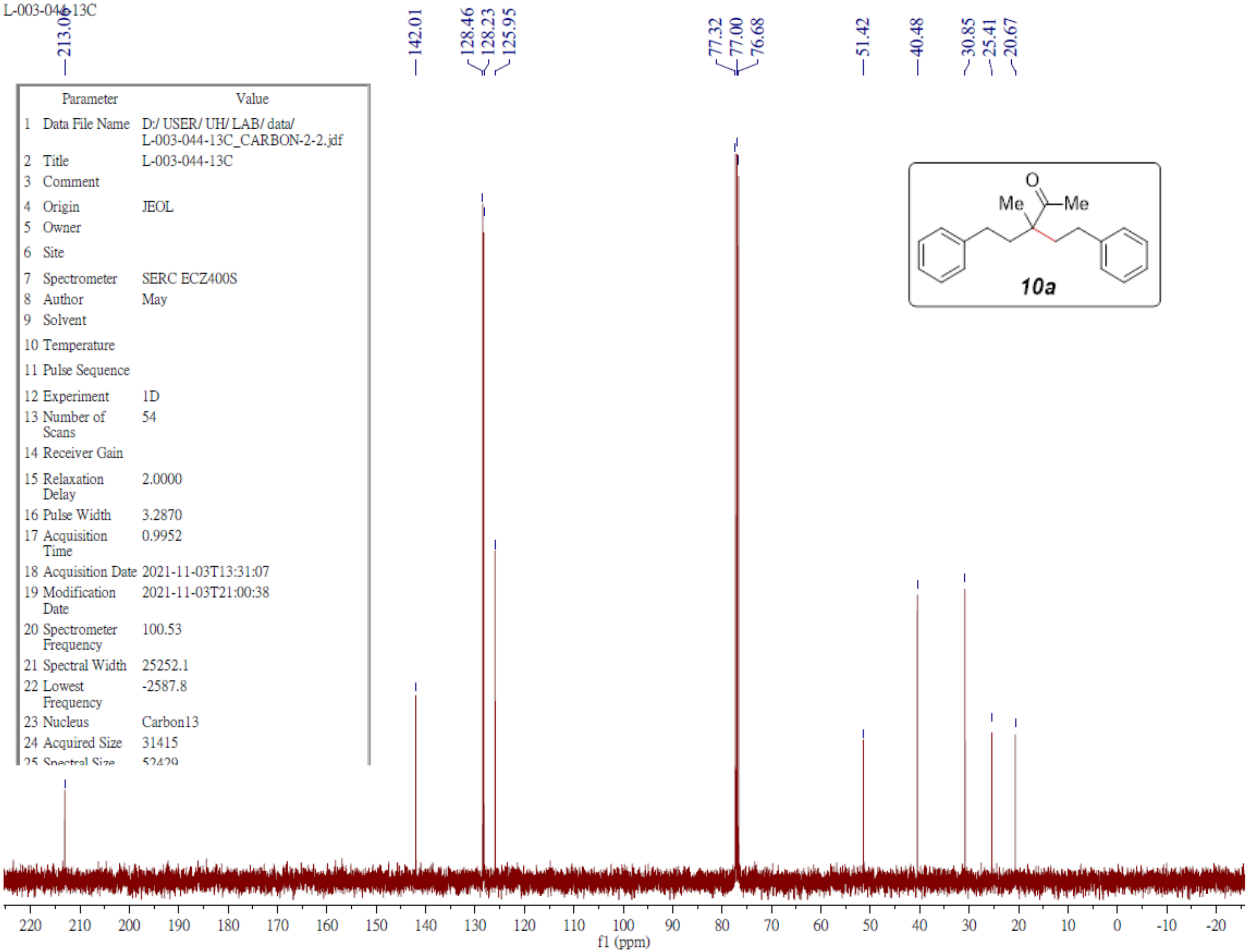

	Parameter	Value
1	Data File Name	D:/ USER/ UH/ LAB/ data/ L-003-044-13C_CARBON-2-2.jdf
2	Title	L-003-044-13C
3	Comment	
4	Origin	JEOL
5	Owner	
6	Site	
7	Spectrometer	SERC ECZ400S
8	Author	May
9	Solvent	
10	Temperature	
11	Pulse Sequence	
12	Experiment	1D
13	Number of Scans	54
14	Receiver Gain	
15	Relaxation Delay	2.0000
16	Pulse Width	3.2870
17	Acquisition Time	0.9952
18	Acquisition Date	2021-11-03T13:31:07
19	Modification Date	2021-11-03T21:00:38
20	Spectrometer Frequency	100.53
21	Spectral Width	25252.1
22	Lowest Frequency	-2587.8
23	Nucleus	Carbon13
24	Acquired Size	31415
25	Spectral Size	52429

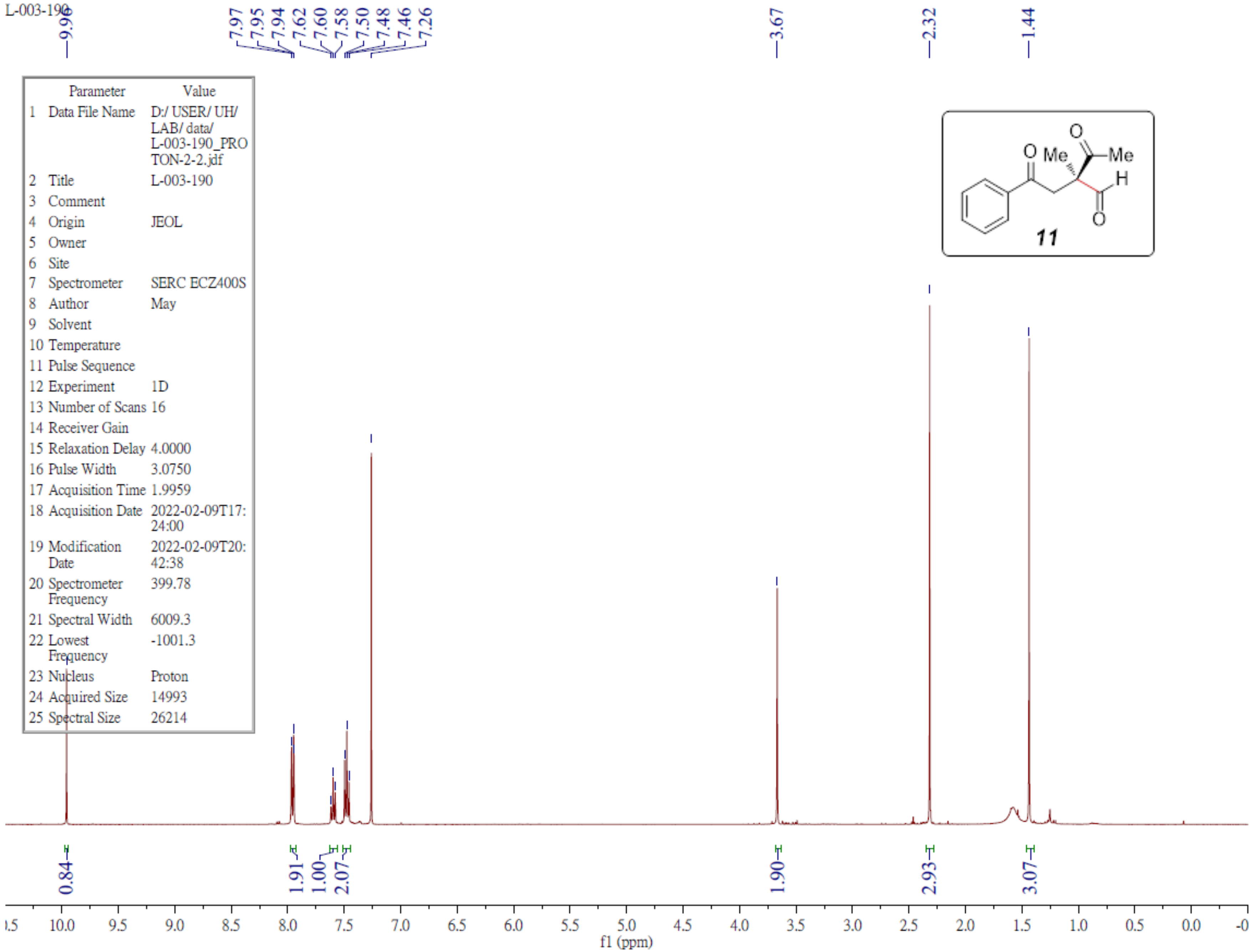

L-003-190
9.96
7.97
7.95
7.94
7.62
7.60
7.58
7.50
7.48
7.46
7.26
3.67
2.32
1.44
Parameter Value
1 Data File Name D:/ USER/ UH/ LAB/ data/ L-003-190_PROTON-2-2.jdf
2 Title L-003-190
3 Comment
4 Origin JEOL
5 Owner
6 Site
7 Spectrometer SERC ECZ400S
8 Author May
9 Solvent
10 Temperature
11 Pulse Sequence
12 Experiment 1D
13 Number of Scans 16
14 Receiver Gain
15 Relaxation Delay 4.0000
16 Pulse Width 3.0750
17 Acquisition Time 1.9959
18 Acquisition Date 2022-02-09T17:24:00
19 Modification Date 2022-02-09T20:42:38
20 Spectrometer Frequency 399.78
21 Spectral Width 6009.3
22 Lowest Frequency -1001.3
23 Nucleus Proton
24 Acquired Size 14993
25 Spectral Size 26214
O
Me
Me
H
11
0.84
1.91
1.00
2.07
1.90
2.93
3.07
f1 (ppm)

L-003-177

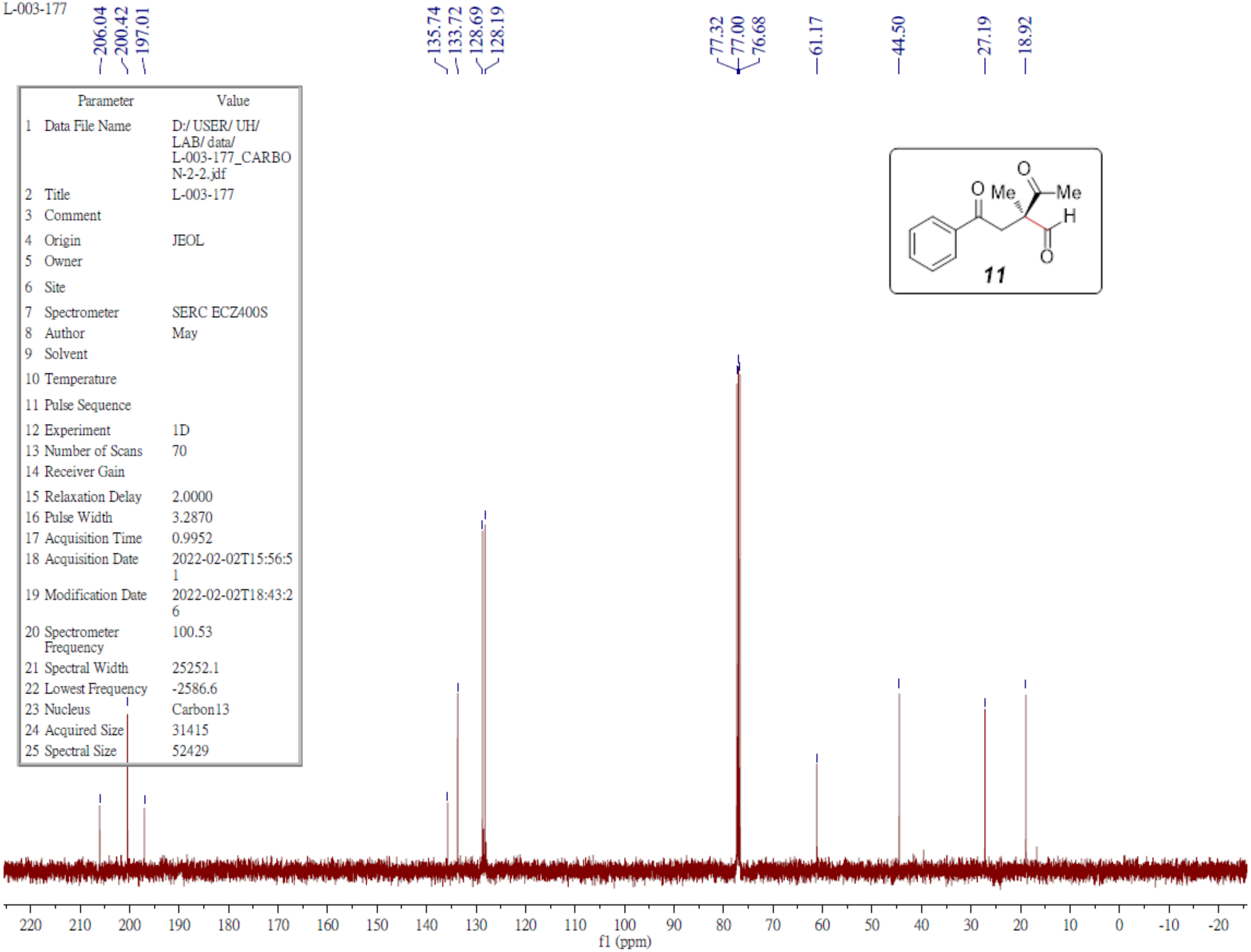

	Parameter	Value
1	Data File Name	D:/ USER/ UH/ LAB/ data/ L-003-177_CARBON-2-2.jdf
2	Title	L-003-177
3	Comment	
4	Origin	JEOL
5	Owner	
6	Site	
7	Spectrometer	SERC ECZ400S
8	Author	May
9	Solvent	
10	Temperature	
11	Pulse Sequence	
12	Experiment	1D
13	Number of Scans	70
14	Receiver Gain	
15	Relaxation Delay	2.0000
16	Pulse Width	3.2870
17	Acquisition Time	0.9952
18	Acquisition Date	2022-02-02T15:56:51
19	Modification Date	2022-02-02T18:43:26
20	Spectrometer Frequency	100.53
21	Spectral Width	25252.1
22	Lowest Frequency	-2586.6
23	Nucleus	Carbon13
24	Acquired Size	31415
25	Spectral Size	52429

11

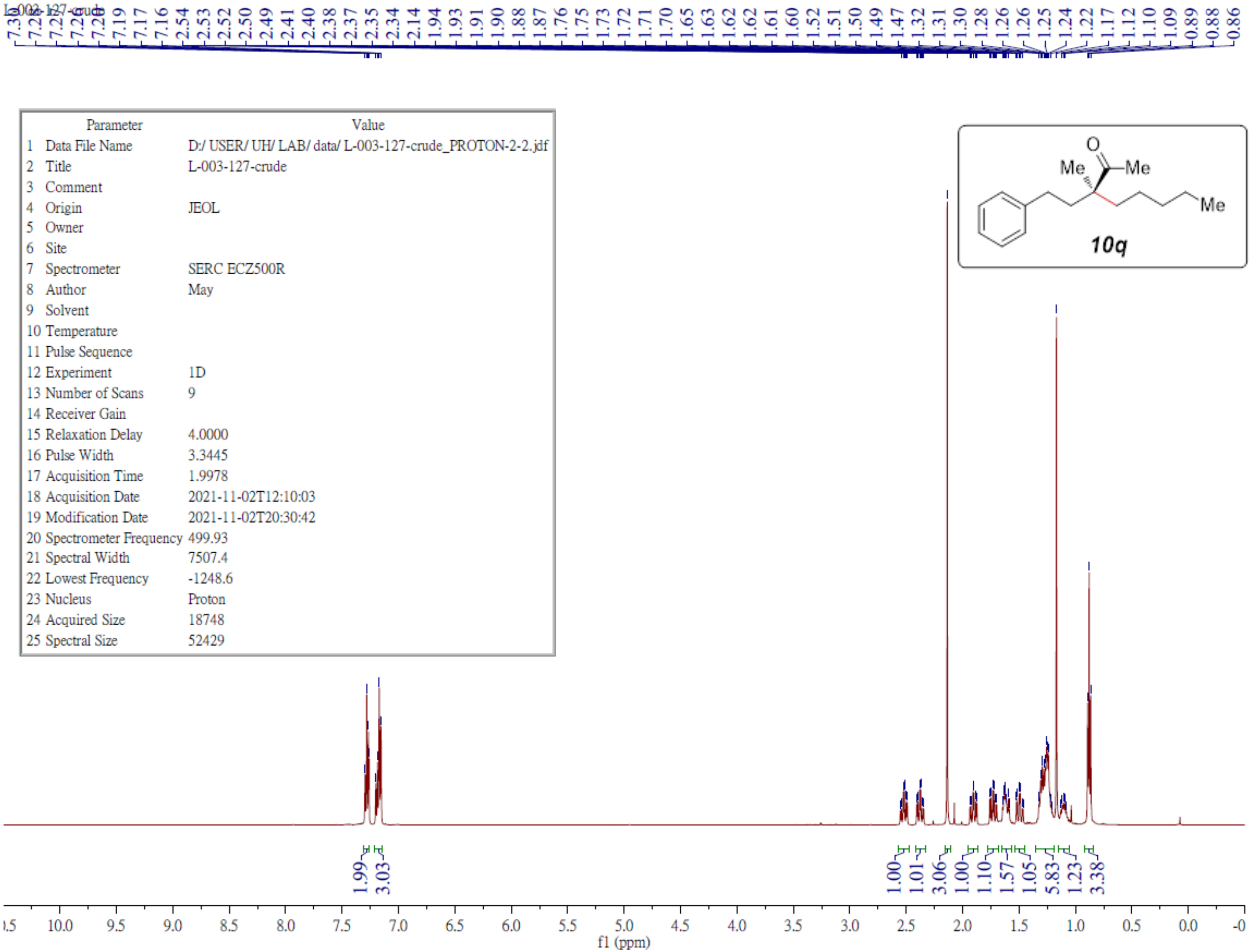

Parameter	Value
1 Data File Name	D:/ USER/ UH/ LAB/ data/ L-003-127-crude_PROTON-2-2.jdf
2 Title	L-003-127-crude
3 Comment	
4 Origin	JEOL
5 Owner	
6 Site	
7 Spectrometer	SERC ECZ500R
8 Author	May
9 Solvent	
10 Temperature	
11 Pulse Sequence	
12 Experiment	1D
13 Number of Scans	9
14 Receiver Gain	
15 Relaxation Delay	4.0000
16 Pulse Width	3.3445
17 Acquisition Time	1.9978
18 Acquisition Date	2021-11-02T12:10:03
19 Modification Date	2021-11-02T20:30:42
20 Spectrometer Frequency	499.93
21 Spectral Width	7507.4
22 Lowest Frequency	-1248.6
23 Nucleus	Proton
24 Acquired Size	18748
25 Spectral Size	52429

10q

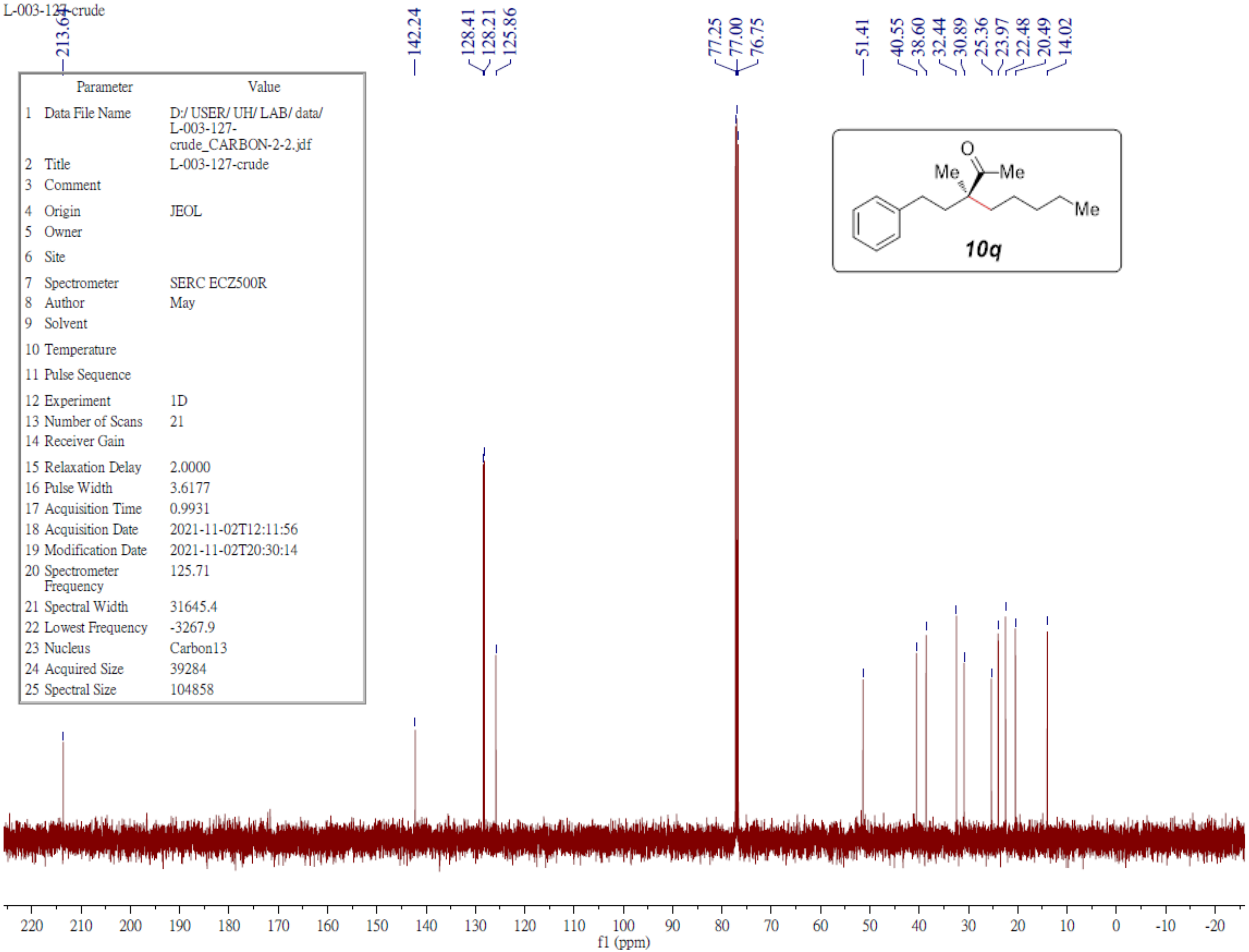

	Parameter	Value
1	Data File Name	D:/ USER/ UH/ LAB/ data/ L-003-127-crude_CARBON-2-2.jdf
2	Title	L-003-127-crude
3	Comment	
4	Origin	JEOL
5	Owner	
6	Site	
7	Spectrometer	SERC ECZ500R
8	Author	May
9	Solvent	
10	Temperature	
11	Pulse Sequence	
12	Experiment	1D
13	Number of Scans	21
14	Receiver Gain	
15	Relaxation Delay	2.0000
16	Pulse Width	3.6177
17	Acquisition Time	0.9931
18	Acquisition Date	2021-11-02T12:11:56
19	Modification Date	2021-11-02T20:30:14
20	Spectrometer Frequency	125.71
21	Spectral Width	31645.4
22	Lowest Frequency	-3267.9
23	Nucleus	Carbon13
24	Acquired Size	39284
25	Spectral Size	104858

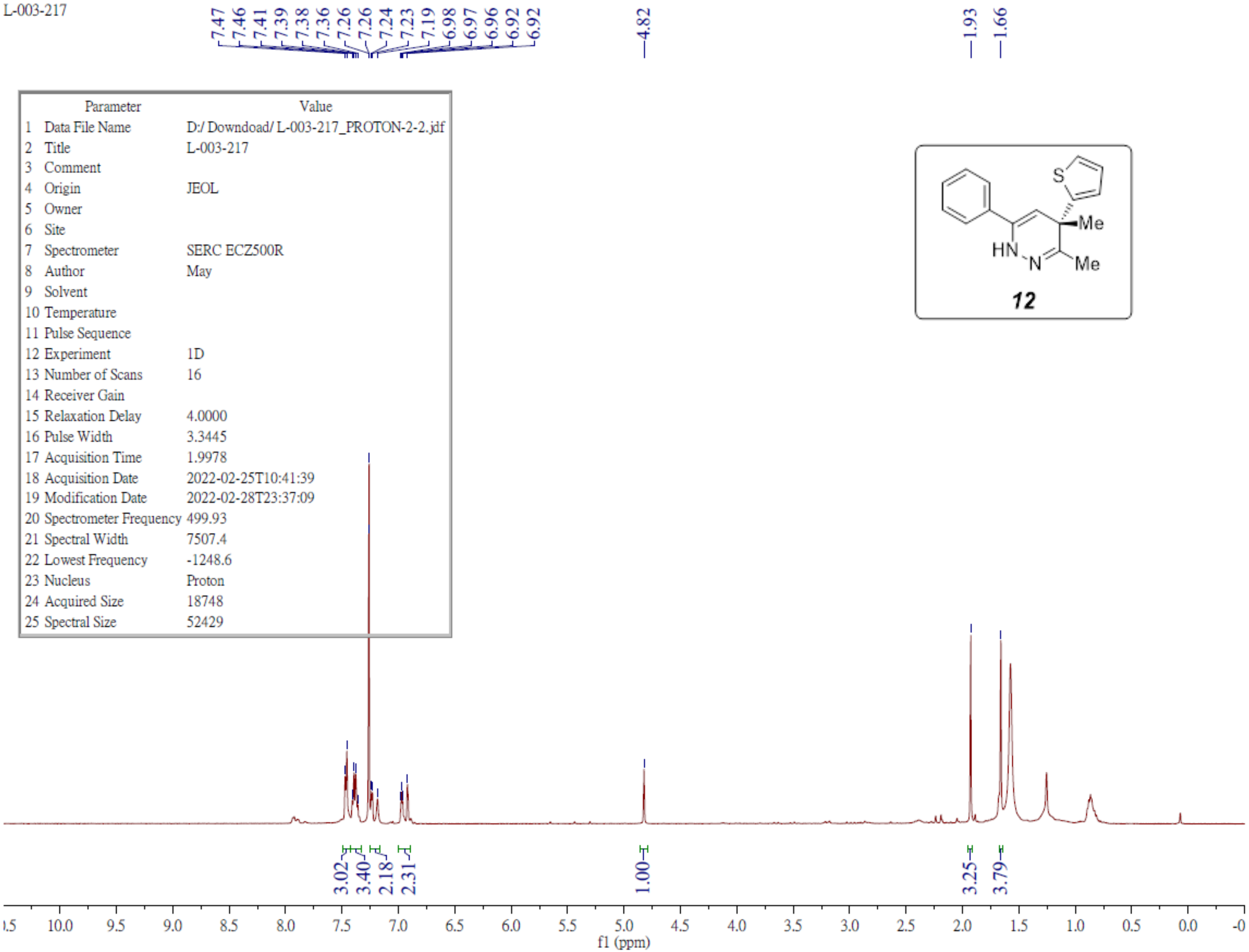

Parameter	Value
1 Data File Name	D:/ Downdoad/ L-003-217_PROTON-2-2.jdf
2 Title	L-003-217
3 Comment	
4 Origin	JEOL
5 Owner	
6 Site	
7 Spectrometer	SERC ECZ500R
8 Author	May
9 Solvent	
10 Temperature	
11 Pulse Sequence	
12 Experiment	1D
13 Number of Scans	16
14 Receiver Gain	
15 Relaxation Delay	4.0000
16 Pulse Width	3.3445
17 Acquisition Time	1.9978
18 Acquisition Date	2022-02-25T10:41:39
19 Modification Date	2022-02-28T23:37:09
20 Spectrometer Frequency	499.93
21 Spectral Width	7507.4
22 Lowest Frequency	-1248.6
23 Nucleus	Proton
24 Acquired Size	18748
25 Spectral Size	52429

12

L-003-217-13C

Parameter		Value
1	Data File Name	D:/ USER/ UH/ LAB/ data/ L-003-217-13C_CARBON-2-2.jdf
2	Title	L-003-217-13C
3	Comment	
4	Origin	JEOL
5	Owner	
6	Site	
7	Spectrometer	SERC ECZ500R
8	Author	May
9	Solvent	
10	Temperature	
11	Pulse Sequence	
12	Experiment	1D
13	Number of Scans	63
14	Receiver Gain	
15	Relaxation Delay	2.0000
16	Pulse Width	3.6177
17	Acquisition Time	0.9931
18	Acquisition Date	2022-02-25T13:07:08
19	Modification Date	2022-02-25T20:32:44
20	Spectrometer Frequency	125.71
21	Spectral Width	31645.4
22	Lowest Frequency	-3267.6
23	Nucleus	Carbon13
24	Acquired Size	39284
25	Spectral Size	104858

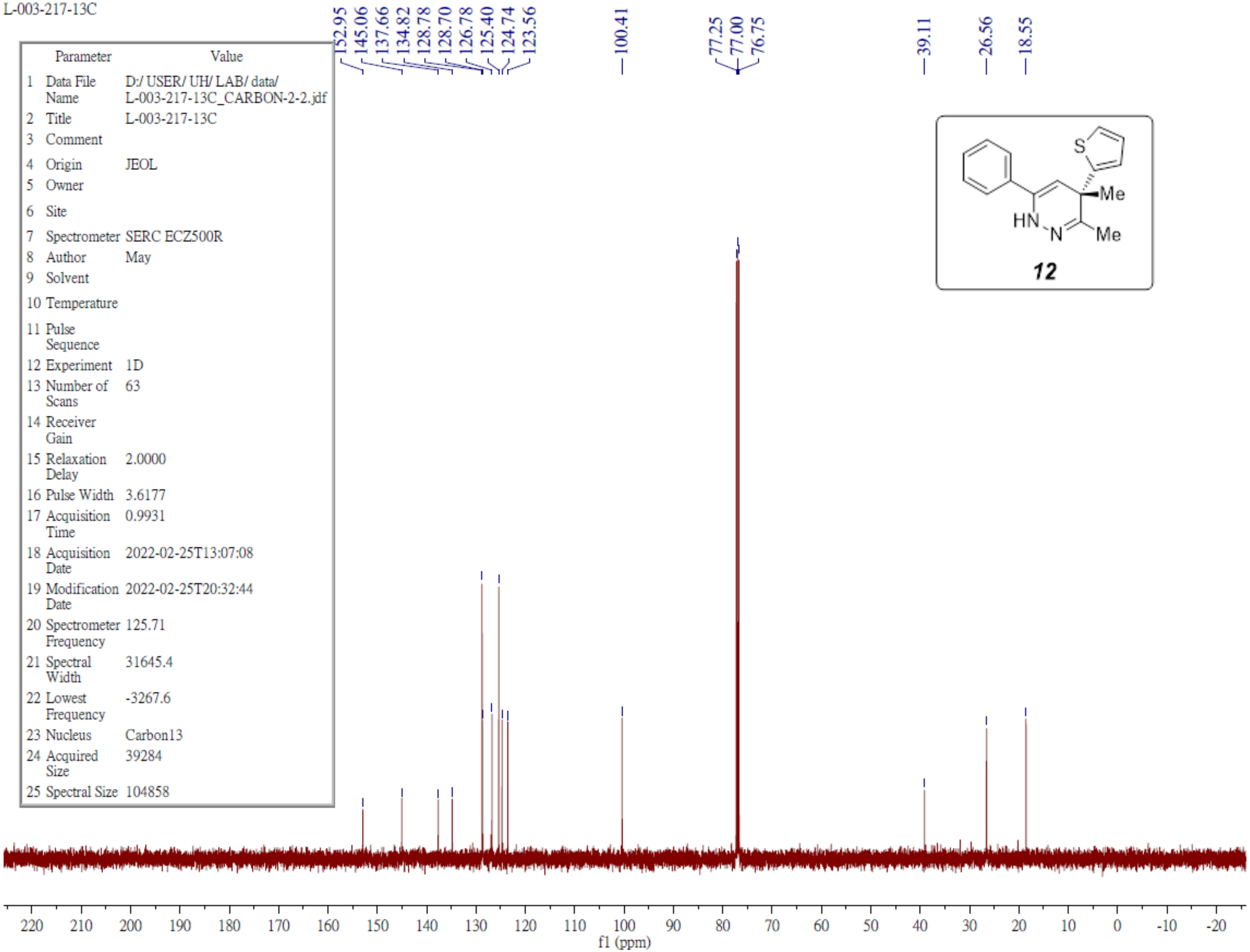

12

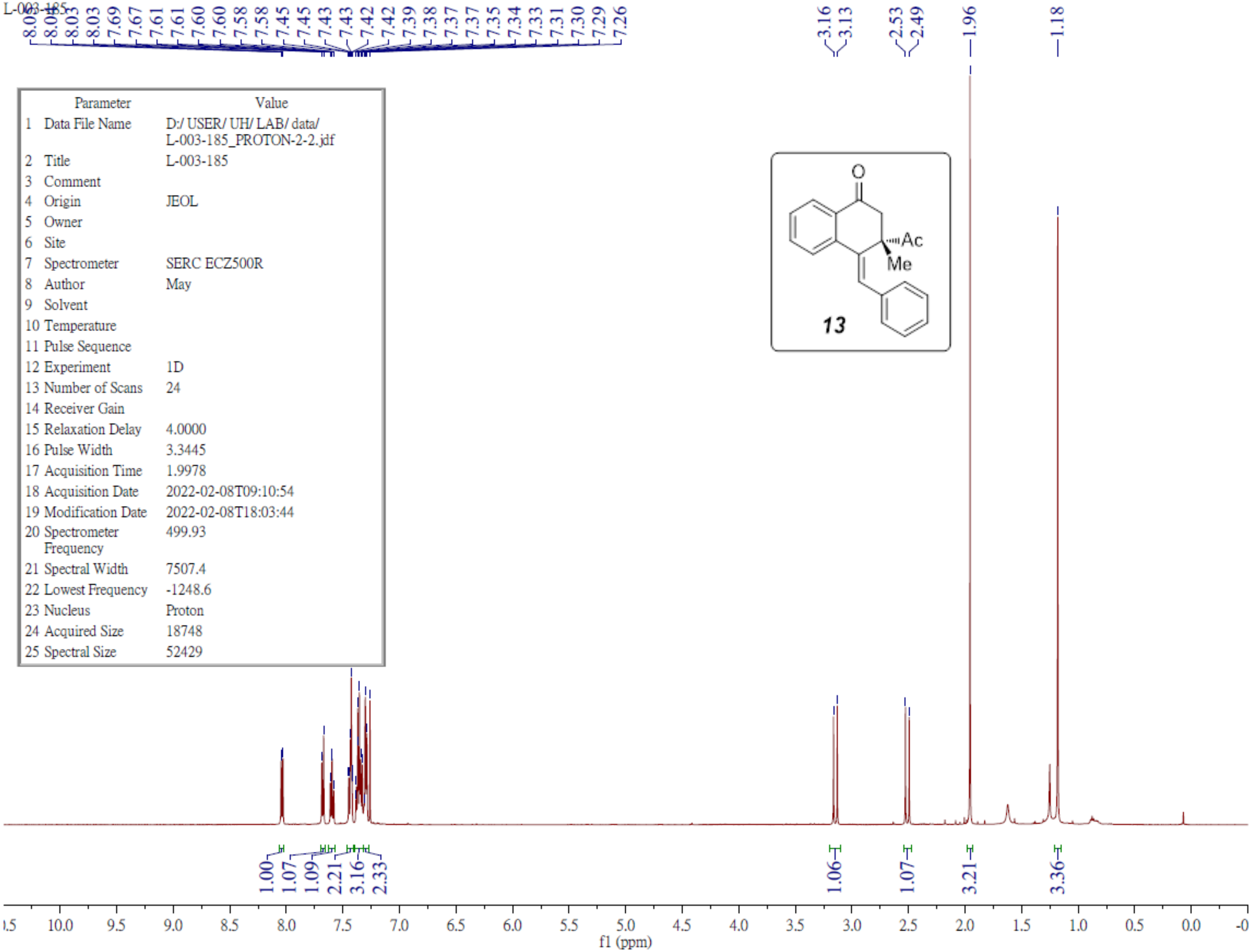
L-003-185
8.05 8.05 8.03 8.03 7.69 7.67 7.61 7.61 7.60 7.60 7.58 7.58 7.45 7.45 7.43 7.43 7.42 7.42 7.39 7.38 7.37 7.37 7.35 7.34 7.33 7.31 7.30 7.29 7.26
3.16 3.13 2.53 2.49 1.96 1.18
Parameter Value
1 Data File Name D:/ USER/ UH/ LAB/ data/ L-003-185_PROTON-2-2.jdf
2 Title L-003-185
3 Comment
4 Origin JEOL
5 Owner
6 Site
7 Spectrometer SERC ECZ500R
8 Author May
9 Solvent
10 Temperature
11 Pulse Sequence
12 Experiment 1D
13 Number of Scans 24
14 Receiver Gain
15 Relaxation Delay 4.0000
16 Pulse Width 3.3445
17 Acquisition Time 1.9978
18 Acquisition Date 2022-02-08T09:10:54
19 Modification Date 2022-02-08T18:03:44
20 Spectrometer Frequency 499.93
21 Spectral Width 7507.4
22 Lowest Frequency -1248.6
23 Nucleus Proton
24 Acquired Size 18748
25 Spectral Size 52429
1.00 1.07 1.09 2.21 3.16 2.33 1.06 1.07 3.21 3.36
10.0 9.5 9.0 8.5 8.0 7.5 7.0 6.5 6.0 5.5 5.0 4.5 4.0 3.5 3.0 2.5 2.0 1.5 1.0 0.5 0.0
f1 (ppm)
O
Ac
Me
13

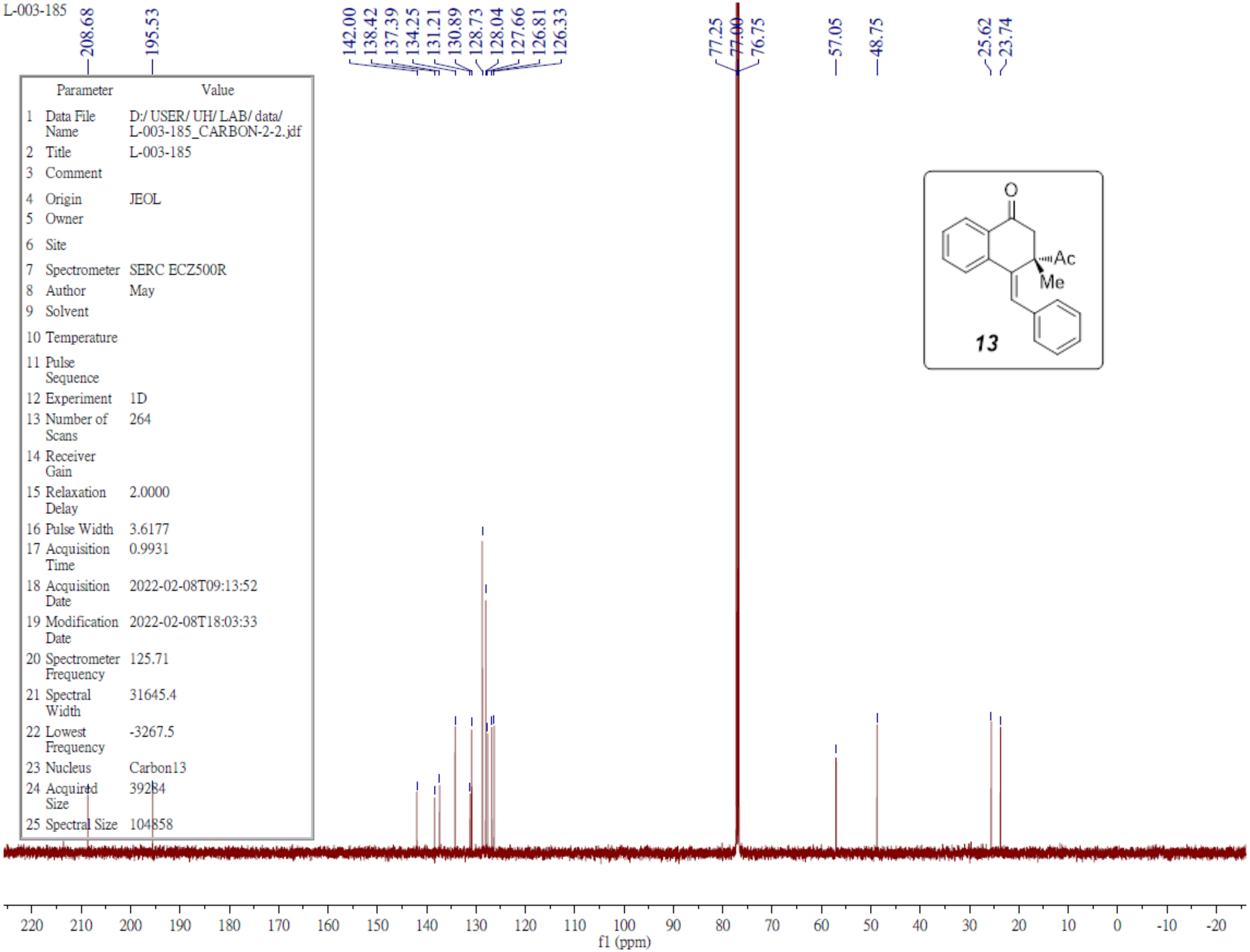

	Parameter	Value
1	Data File Name	D:/ USER/ UH/ LAB/ data/ L-003-185_CARBON-2-2.jdf
2	Title	L-003-185
3	Comment	
4	Origin	JEOL
5	Owner	
6	Site	
7	Spectrometer	SERC ECZ500R
8	Author	May
9	Solvent	
10	Temperature	
11	Pulse Sequence	
12	Experiment	1D
13	Number of Scans	264
14	Receiver Gain	
15	Relaxation Delay	2.0000
16	Pulse Width	3.6177
17	Acquisition Time	0.9931
18	Acquisition Date	2022-02-08T09:13:52
19	Modification Date	2022-02-08T18:03:33
20	Spectrometer Frequency	125.71
21	Spectral Width	31645.4
22	Lowest Frequency	-3267.5
23	Nucleus	Carbon13
24	Acquired Size	39284
25	Spectral Size	104858

==== Shimadzu LCsolution Analysis Report ====

C:\Users\user\Desktop\PoKai\L-002-117-90%-3-10min-1.0.lcd

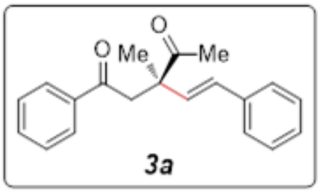

Acquired by	: Admin
Sample Name	: L-002-117-90%-3-10min
Sample ID	: L-002-117-90%-3-10min
Tray#	: 1
Vail #	: 1
Injection Volume	: 10 uL
Data File Name	: L-002-117-90%-3-10min-1.0.lcd
Method File Name	: pos3-90%_10MIN_1_d2.lcm
Batch File Name	: Batch table C3 _90%_10min_1.0_D2.lcb
Report File Name	: Default.lcr
Data Acquired	: 3/19/2021 3:03:27 PM
Data Processed	: 3/19/2021 3:13:29 PM

<Chromatogram>

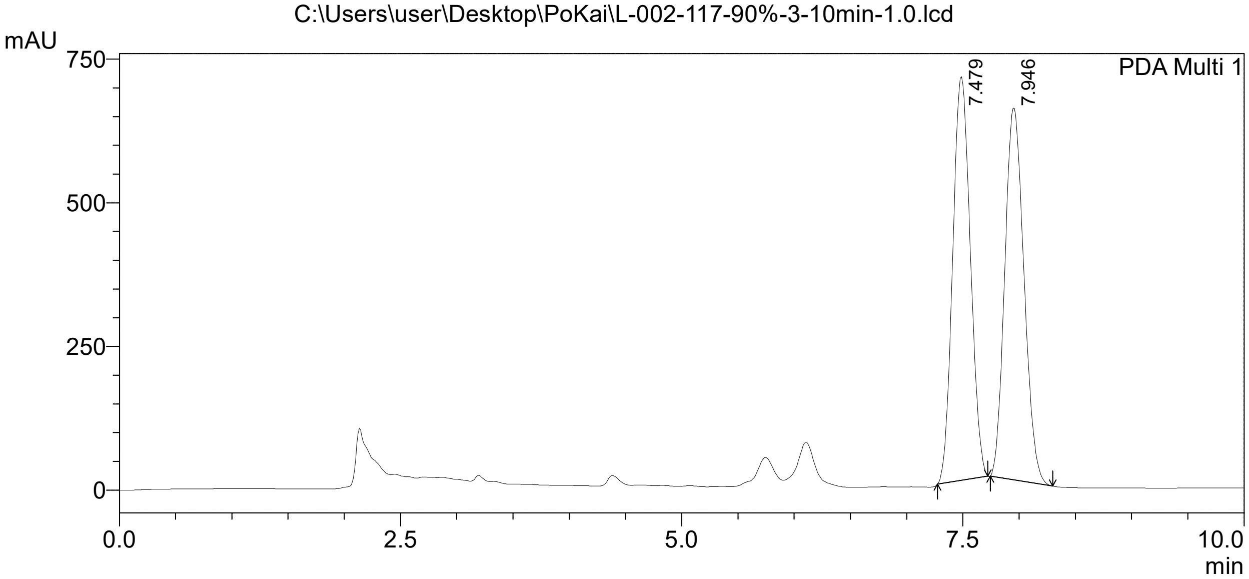

1 PDA Multi 1/254nm 4nm

PeakTable

PDA Ch1 254nm 4nm

Peak#	Ret. Time	Area	Height	Area %	Height %
1	7.479	7667444	702597	49.971	52.066
2	7.946	7676393	646827	50.029	47.934
Total		15343837	1349424	100.000	100.000

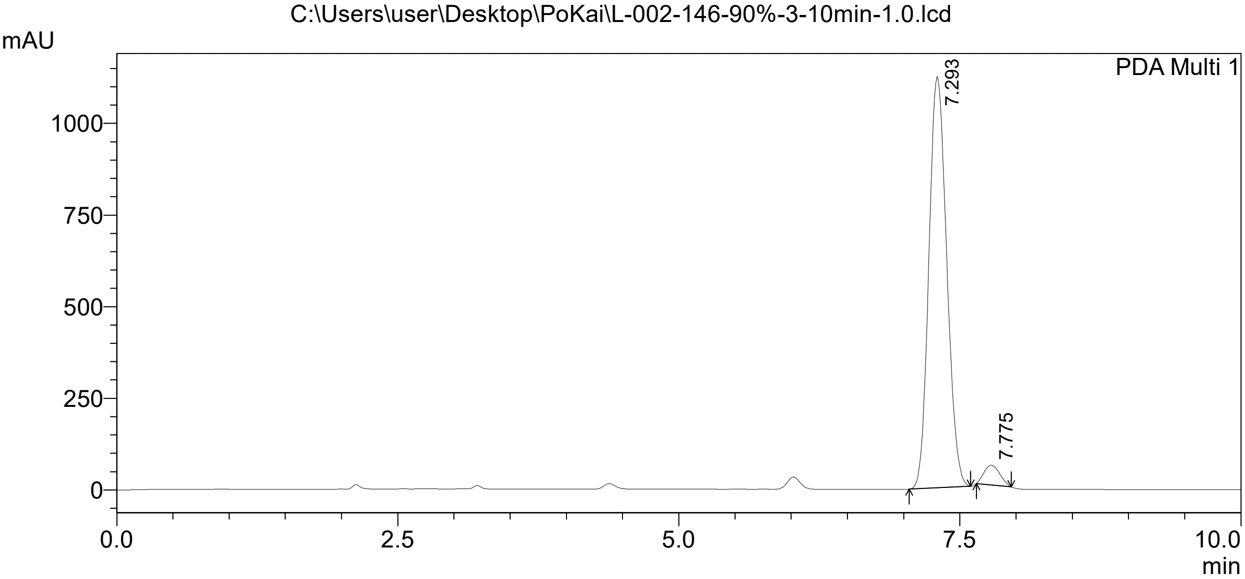

1 PDA Multi 1/254nm 4nm

PeakTable

PDA Ch1 254nm 4nm

Peak#	Ret. Time	Area	Height	Area %	Height %
1	7.293	12375349	1121802	95.941	95.438
2	7.775	523574	53619	4.059	4.562
Total		12898924	1175421	100.000	100.000

==== Shimadzu LCsolution Analysis Report ====

C:\Users\user\Desktop\PoKai\L-003-133-90%-3-30min-1.0.lcd

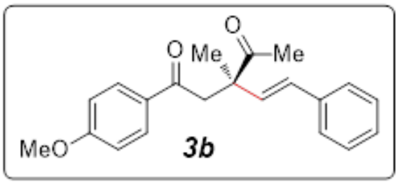

Acquired by	: Admin
Sample Name	: L-003-133-90%-3-30min
Sample ID	: L-003-133-90%-3-30min
Tray#	: 1
Vail #	: 1
Injection Volume	: 10 uL
Data File Name	: L-003-133-90%-3-30min-1.0.lcd
Method File Name	: pos3-90%_30MIN_1_d2.lcm
Batch File Name	: Batch table C3 _90%_30min_1.0_D2.lcb
Report File Name	: Default.lcr
Data Acquired	: 11/10/2021 11:29:52 AM
Data Processed	: 11/10/2021 11:59:53 AM

<Chromatogram>

C:\Users\user\Desktop\PoKai\L-003-133-90%-3-30min-1.0.lcd

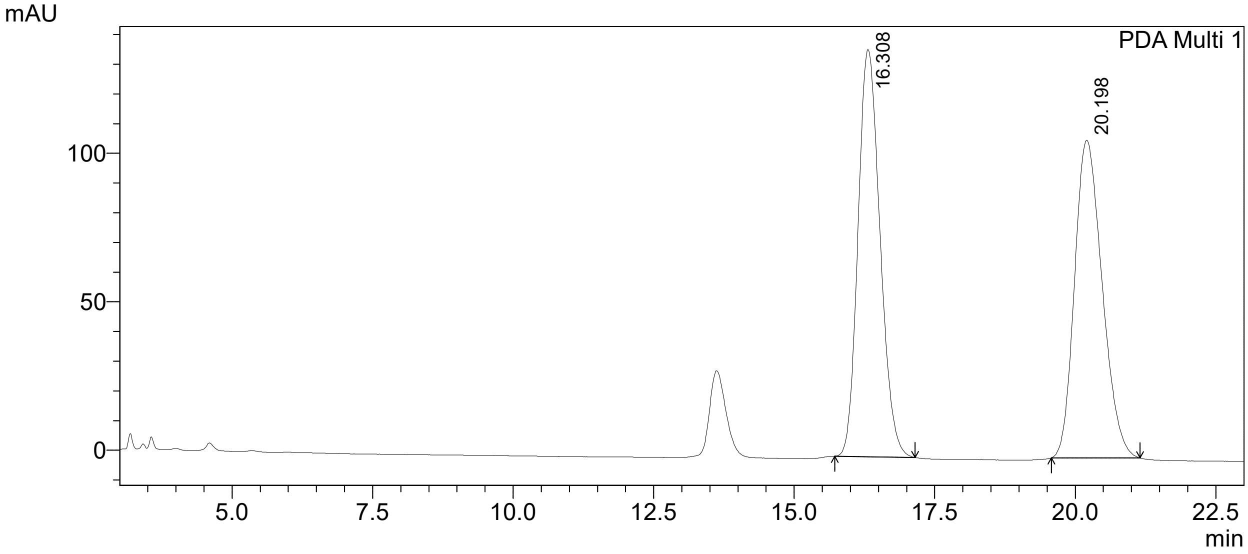

1 PDA Multi 1/254nm 4nm

PeakTable

PDA Ch1 254nm 4nm

Peak#	Ret. Time	Area	Height	Area %	Height %
1	16.308	3740251	137104	50.533	56.163
2	20.198	3661302	107012	49.467	43.837
Total		7401552	244115	100.000	100.000

C:\Users\user\Desktop\PoKai\L-002-206-90%-3-20min-1.0.lcd

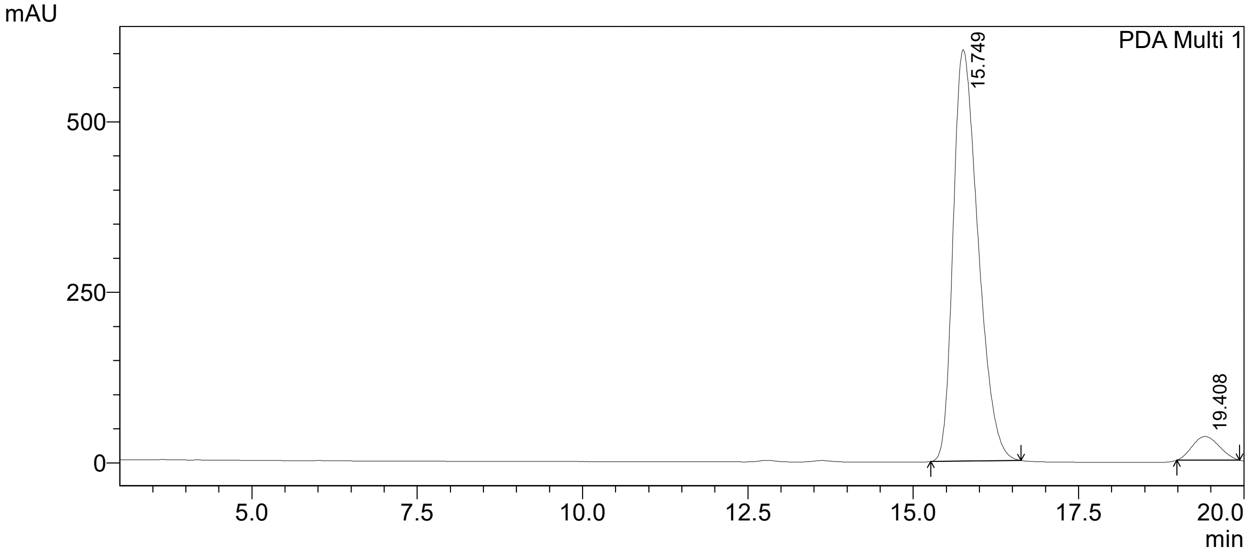

1 PDA Multi 1/254nm 4nm

PeakTable

PDA Ch1 254nm 4nm

Peak#	Ret. Time	Area	Height	Area %	Height %
1	15.749	15852173	603241	94.138	94.573
2	19.408	987177	34618	5.862	5.427
Total		16839350	637858	100.000	100.000

==== Shimadzu LCsolution Analysis Report ====

C:\Users\user\Desktop\PoKai\L-003-048-90%-3-10min-1.0.lcd

Acquired by	: Admin
Sample Name	: L-003-048-90%-3-10min
Sample ID	: L-003-048-90%-3-10min
Tray#	: 1
Vail #	: 1
Injection Volume	: 10 uL
Data File Name	: L-003-048-90%-3-10min-1.0.lcd
Method File Name	: pos3-90%_10MIN_1_d2.lcm
Batch File Name	: Batch table C3 _90%_10min_1.0_D2.lcb
Report File Name	: Default.lcr
Data Acquired	: 9/13/2021 12:53:59 PM
Data Processed	: 9/13/2021 1:04:01 PM

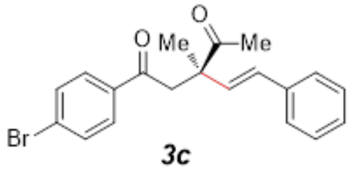

3c

<Chromatogram>

C:\Users\user\Desktop\PoKai\L-003-048-90%-3-10min-1.0.lcd

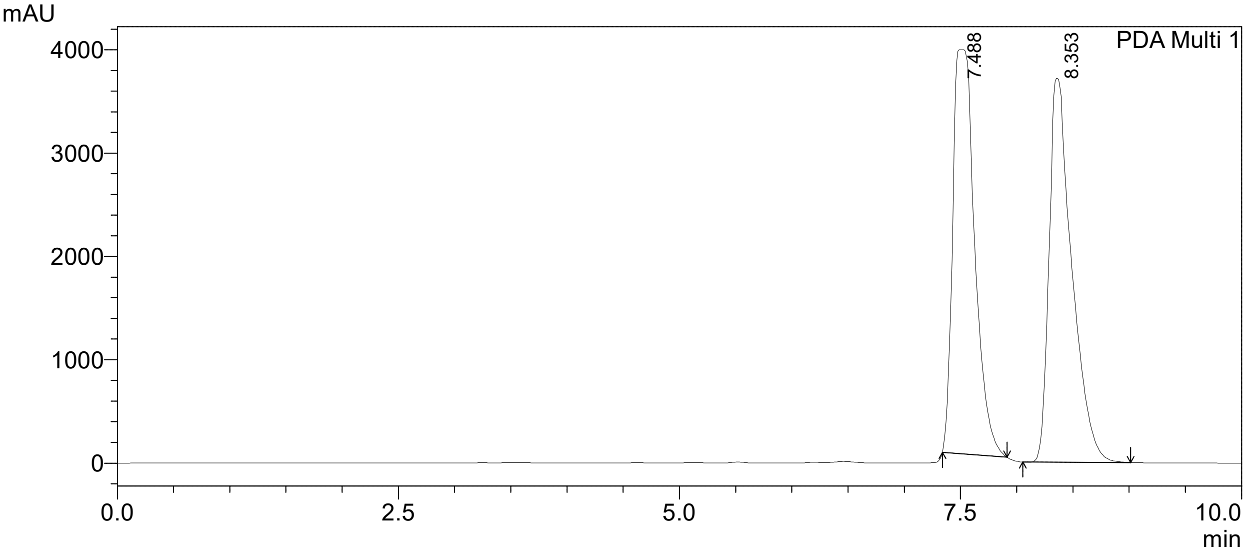

1 PDA Multi 1/254nm 4nm

PeakTable

PDA Ch1 254nm 4nm

Peak#	Ret. Time	Area	Height	Area %	Height %
1	7.488	53324348	3909166	50.419	51.257
2	8.353	52438489	3717457	49.581	48.743
Total		105762837	7626622	100.000	100.000

C:\Users\user\Desktop\PoKai\L-003-047-90%-3-10min-1.0.lcd

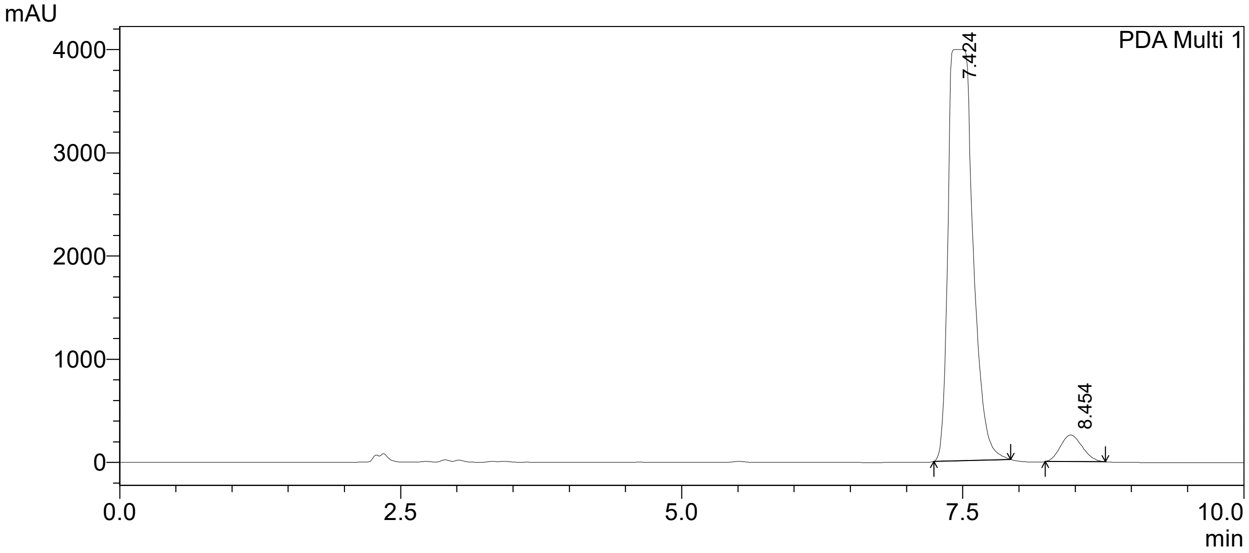

1 PDA Multi 1/254nm 4nm

PeakTable

PDA Ch1 254nm 4nm

Peak#	Ret. Time	Area	Height	Area %	Height %
1	7.424	60660309	3984890	94.541	93.916
2	8.454	3502606	258125	5.459	6.084
Total		64162915	4243015	100.000	100.000

==== Shimadzu LCsolution Analysis Report ====

C:\Users\user\Desktop\PoKai\L-002-214-90%-3-20min-1.0.lcd

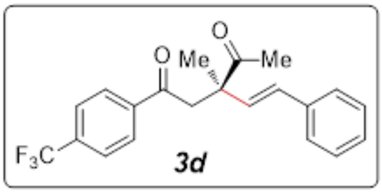

Acquired by	: Admin
Sample Name	: L-002-214-90%-3-20min
Sample ID	: L-002-214-90%-3-20min
Tray#	: 1
Vail #	: 1
Injection Volume	: 10 uL
Data File Name	: L-002-214-90%-3-20min-1.0.lcd
Method File Name	: pos3-90%_20MIN_1_d2.lcm
Batch File Name	: Batch table C3 _90%_20min_1.0_D2.lcb
Report File Name	: Default.lcr
Data Acquired	: 6/2/2021 10:54:26 AM
Data Processed	: 6/2/2021 11:14:28 AM

<Chromatogram>

C:\Users\user\Desktop\PoKai\L-002-214-90%-3-20min-1.0.lcd

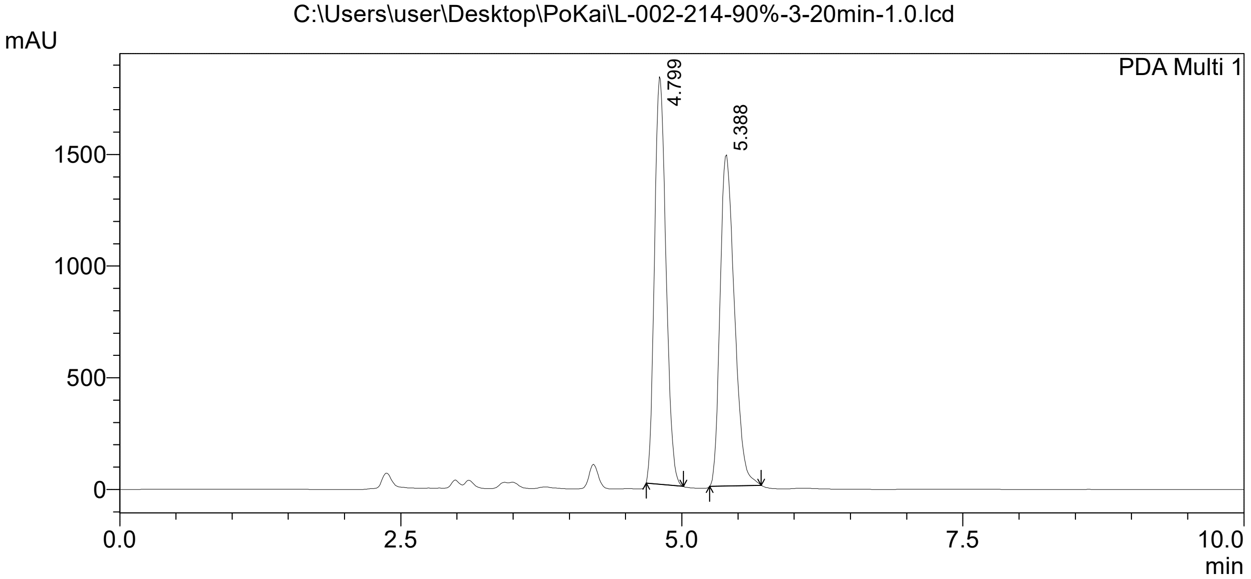

1 PDA Multi 1/254nm 4nm

PeakTable

PDA Ch1 254nm 4nm

Peak#	Ret. Time	Area	Height	Area %	Height %
1	4.799	13008870	1824717	49.547	55.157
2	5.388	13246875	1483487	50.453	44.843
Total		26255745	3308204	100.000	100.000

C:\Users\user\Desktop\PoKai\L-002-208-90%-3-10min-1.0.lcd

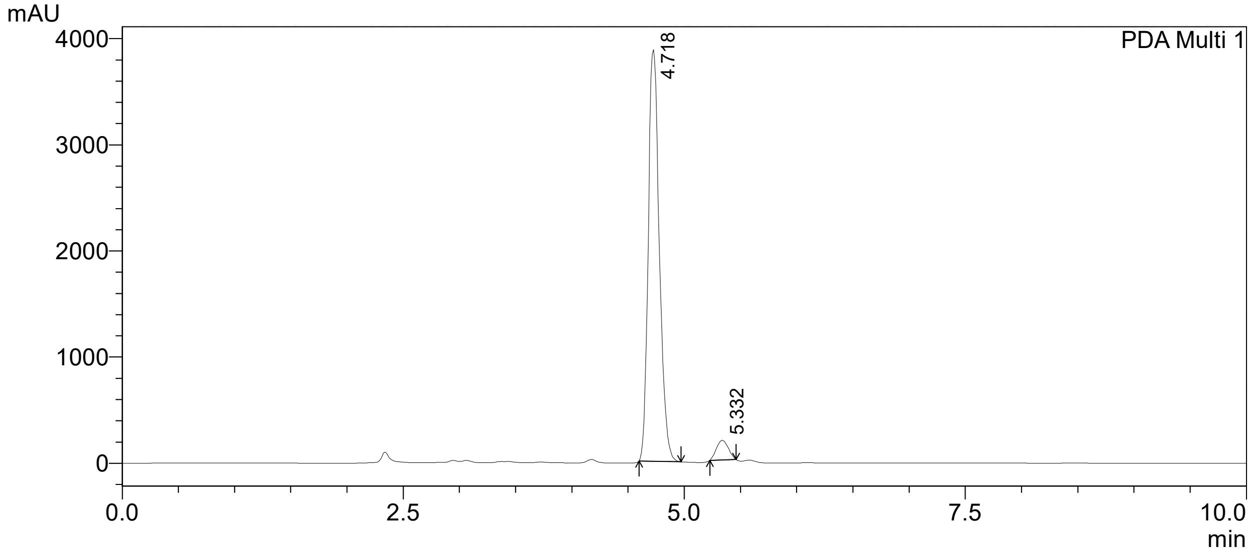

1 PDA Multi 1/254nm 4nm

PeakTable

PDA Ch1 254nm 4nm

Peak#	Ret. Time	Area	Height	Area %	Height %
1	4.718	26671994	3876290	95.144	95.457
2	5.332	1361186	184478	4.856	4.543
Total		28033181	4060768	100.000	100.000

==== Shimadzu LCsolution Analysis Report ====

C:\Users\user\Desktop\PoKai\L-003-213-90%-3-10min-1.0.lcd

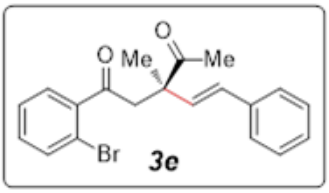

Acquired by	: Admin
Sample Name	: L-003-213-90%-3-10min
Sample ID	: L-003-213-90%-3-10min
Tray#	: 1
Vail #	: 1
Injection Volume	: 10 uL
Data File Name	: L-003-213-90%-3-10min-1.0.lcd
Method File Name	: pos3-90%_10MIN_1_d2.lcm
Batch File Name	: Batch table C3 _90%_10min_1.0_D2.lcb
Report File Name	: Default.lcr
Data Acquired	: 2/23/2022 9:24:20 AM
Data Processed	: 2/23/2022 9:34:22 AM

<Chromatogram>

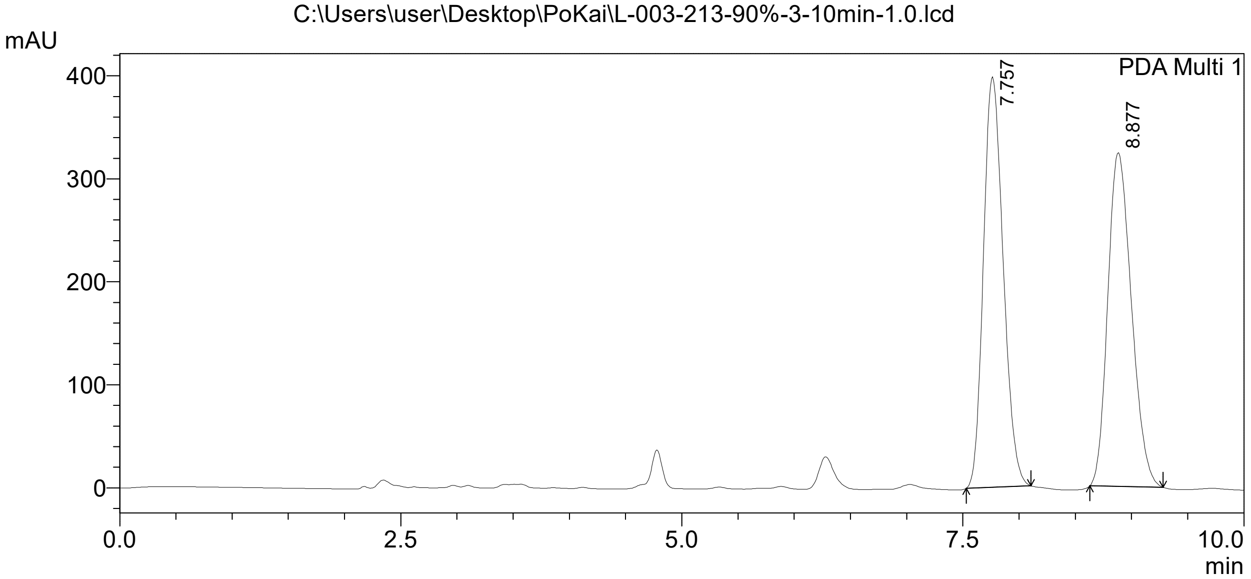

1 PDA Multi 1/254nm 4nm

PeakTable

PDA Ch1 254nm 4nm

Peak#	Ret. Time	Area	Height	Area %	Height %
1	7.757	4879307	398595	50.702	55.167
2	8.877	4744156	323930	49.298	44.833
Total		9623463	722525	100.000	100.000

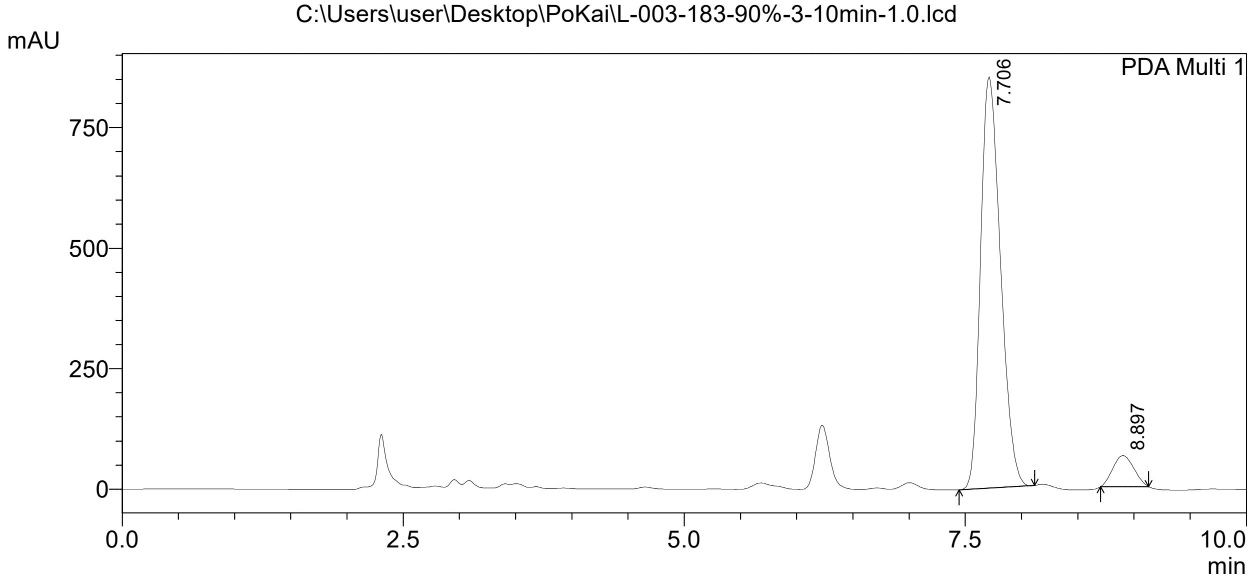

1 PDA Multi 1/254nm 4nm

PeakTable

PDA Ch1 254nm 4nm

Peak#	Ret. Time	Area	Height	Area %	Height %
1	7.706	10544283	853078	92.653	93.007
2	8.897	836136	64145	7.347	6.993
Total		11380419	917223	100.000	100.000

==== Shimadzu LCsolution Analysis Report ====

C:\Users\user\Desktop\PoKai\L-002-218-90%-3-20min-1.0.lcd

Acquired by	: Admin
Sample Name	: L-002-218-90%-3-20min
Sample ID	: L-002-218-90%-3-20min
Tray#	: 1
Vail #	: 1
Injection Volume	: 10 uL
Data File Name	: L-002-218-90%-3-20min-1.0.lcd
Method File Name	: pos3-90%_20MIN_1_d2.lcm
Batch File Name	: Batch table C3 _90%_20min_1.0_D2.lcb
Report File Name	: Default.lcr
Data Acquired	: 6/3/2021 3:00:27 PM
Data Processed	: 6/3/2021 3:20:30 PM

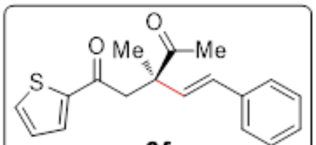

3f

<Chromatogram>

C:\Users\user\Desktop\PoKai\L-002-218-90%-3-20min-1.0.lcd

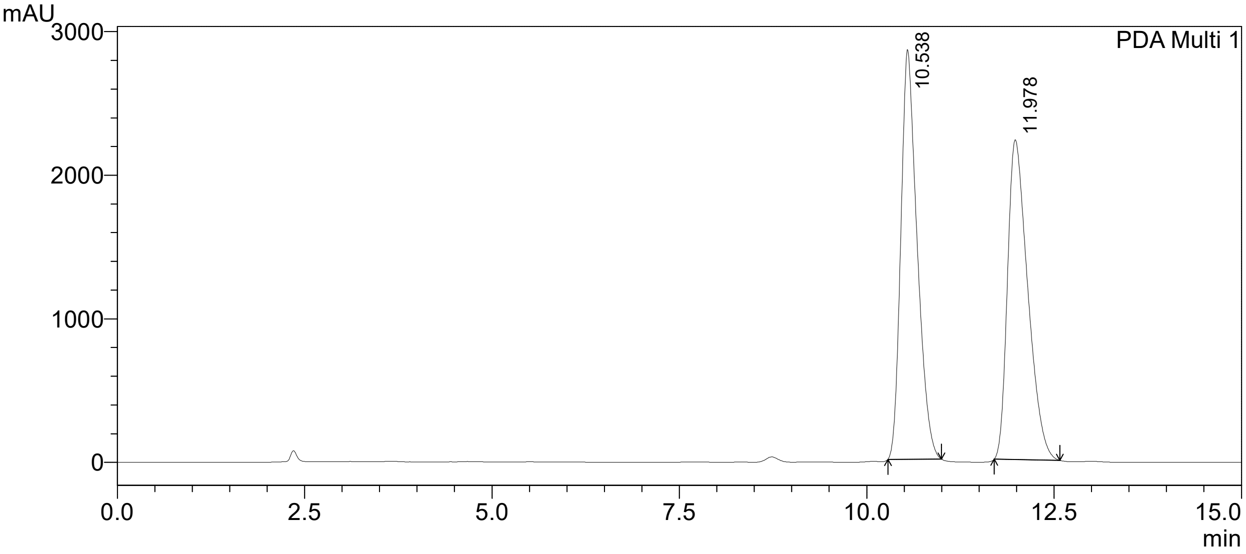

1 PDA Multi 1/254nm 4nm

PeakTable

PDA Ch1 254nm 4nm

Peak#	Ret. Time	Area	Height	Area %	Height %
1	10.538	43129030	2853276	50.723	56.163
2	11.978	41898975	2227060	49.277	43.837
Total		85028005	5080335	100.000	100.000

C:\Users\user\Desktop\PoKai\L-002-210-90%-3-20min-1.0.lcd

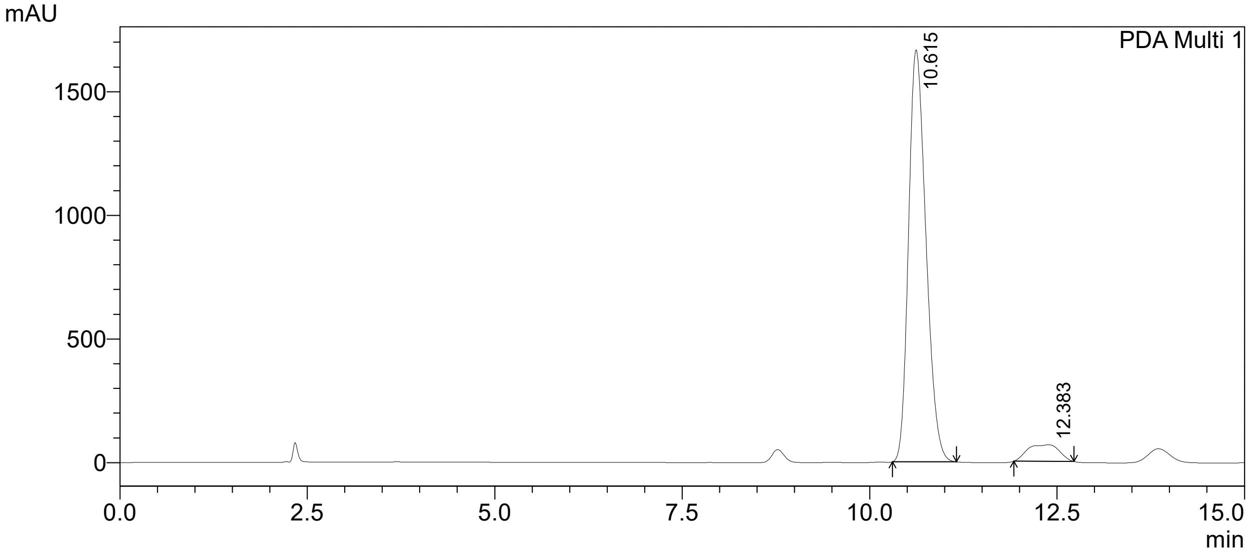

1 PDA Multi 1/254nm 4nm

PeakTable

PDA Ch1 254nm 4nm

Peak#	Ret. Time	Area	Height	Area %	Height %
1	10.615	26922674	1665760	93.177	96.122
2	12.383	1971385	67202	6.823	3.878
Total		28894059	1732962	100.000	100.000

==== Shimadzu LCsolution Analysis Report ====

C:\Users\user\Desktop\PoKai\L-002-122-90%-3-20min-1.0.lcd

Acquired by	: Admin
Sample Name	: L-002-122-90%-3-20min
Sample ID	: L-002-122-90%-3-20min
Tray#	: 1
Vail #	: 1
Injection Volume	: 10 uL
Data File Name	: L-002-122-90%-3-20min-1.0.lcd
Method File Name	: pos3-90%_20MIN_1_d2.lcm
Batch File Name	: Batch table C3 _90%_20min_1.0_D2.lcb
Report File Name	: Default.lcr
Data Acquired	: 10/25/2021 10:48:15 AM
Data Processed	: 10/25/2021 11:08:18 AM

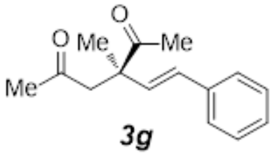

3g

<Chromatogram>

C:\Users\user\Desktop\PoKai\L-002-122-90%-3-20min-1.0.lcd

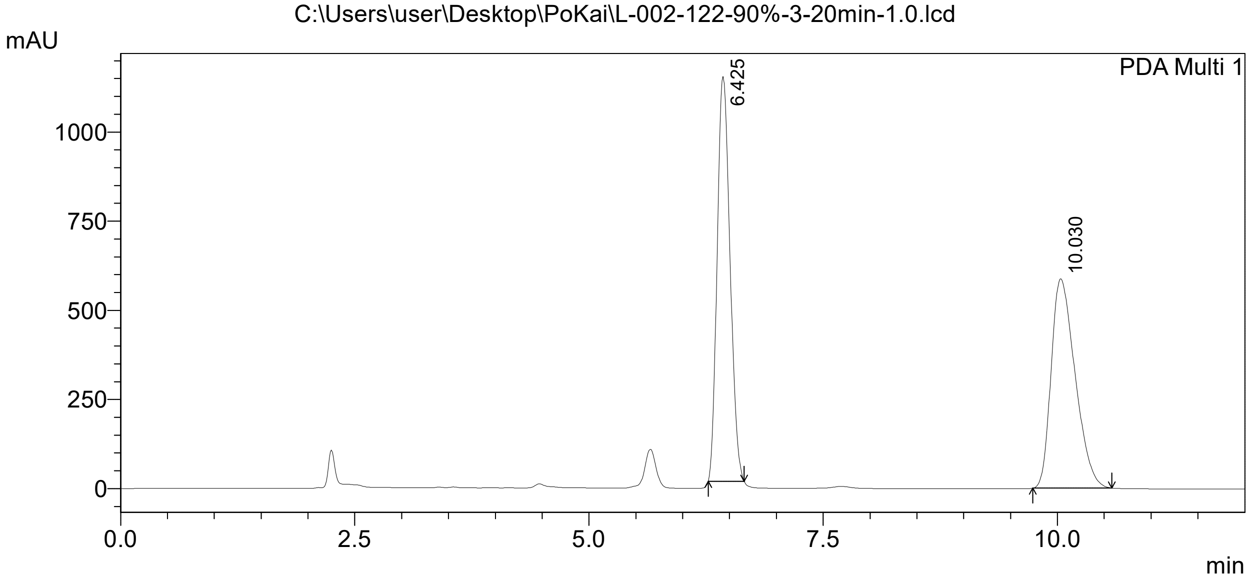

1 PDA Multi 1/254nm 4nm

PeakTable

PDA Ch1 254nm 4nm

Peak#	Ret. Time	Area	Height	Area %	Height %
1	6.425	10821336	1134759	50.883	65.895
2	10.030	10445738	587311	49.117	34.105
Total		21267074	1722071	100.000	100.000

C:\Users\user\Desktop\PoKai\L-003-112-90%-3-20min-1.0.lcd

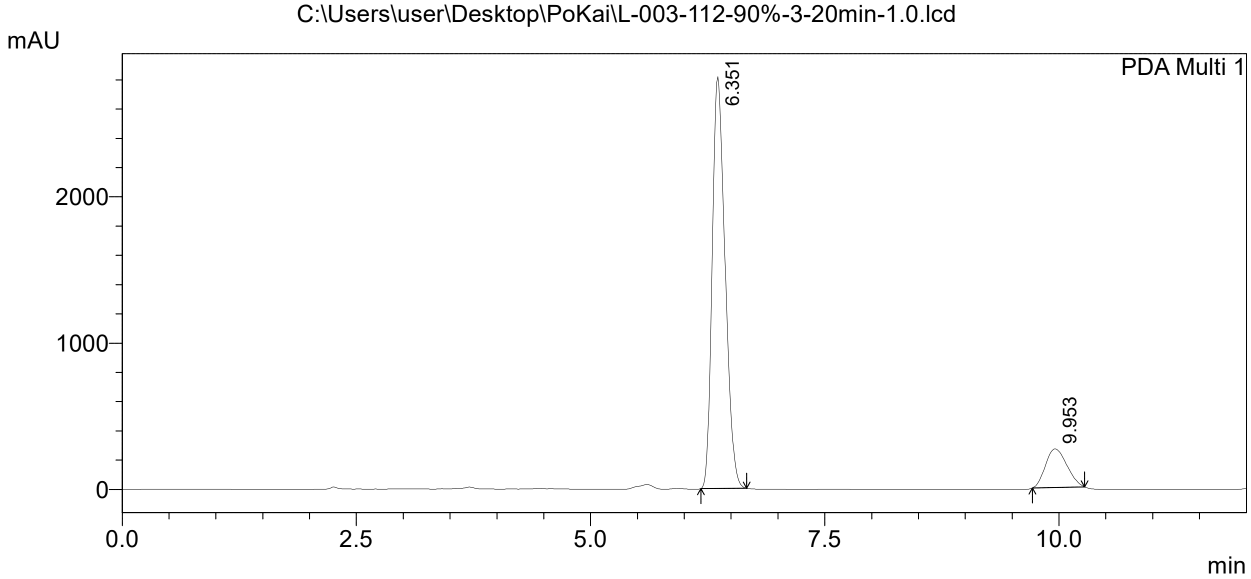

1 PDA Multi 1/254nm 4nm

PeakTable

PDA Ch1 254nm 4nm

Peak#	Ret. Time	Area	Height	Area %	Height %
1	6.351	26987107	2814412	86.557	91.408
2	9.953	4191202	264537	13.443	8.592
Total		31178309	3078949	100.000	100.000

==== Shimadzu LCsolution Analysis Report ====

C:\Users\user\Desktop\PoKai\L-002-228-90%-3-10min-1.0.lcd

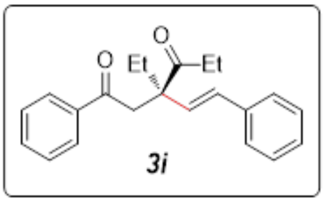

3i

Acquired by	: Admin
Sample Name	: L-002-228-90%-3-10min
Sample ID	: L-002-228-90%-3-10min
Tray#	: 1
Vail #	: 1
Injection Volume	: 10 uL
Data File Name	: L-002-228-90%-3-10min-1.0.lcd
Method File Name	: pos3-90%_10MIN_1_d2.lcm
Batch File Name	: Batch table C3 _90%_10min_1.0_D2.lcb
Report File Name	: Default.lcr
Data Acquired	: 6/10/2021 4:28:50 PM
Data Processed	: 6/10/2021 4:38:51 PM

<Chromatogram>

C:\Users\user\Desktop\PoKai\L-002-228-90%-3-10min-1.0.lcd

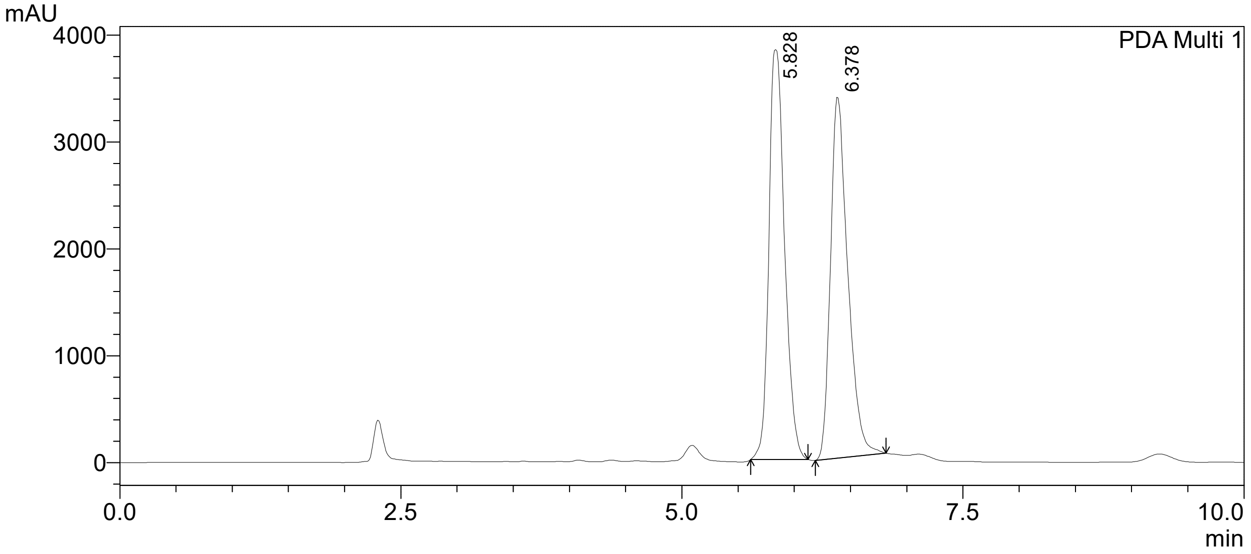

1 PDA Multi 1/254nm 4nm

PeakTable

PDA Ch1 254nm 4nm

Peak#	Ret. Time	Area	Height	Area %	Height %
1	5.828	37423693	3835618	50.760	53.177
2	6.378	36302791	3377296	49.240	46.823
Total		73726483	7212914	100.000	100.000

C:\Users\user\Desktop\PoKai\L-002-224-90%-3-10min-1.0.lcd

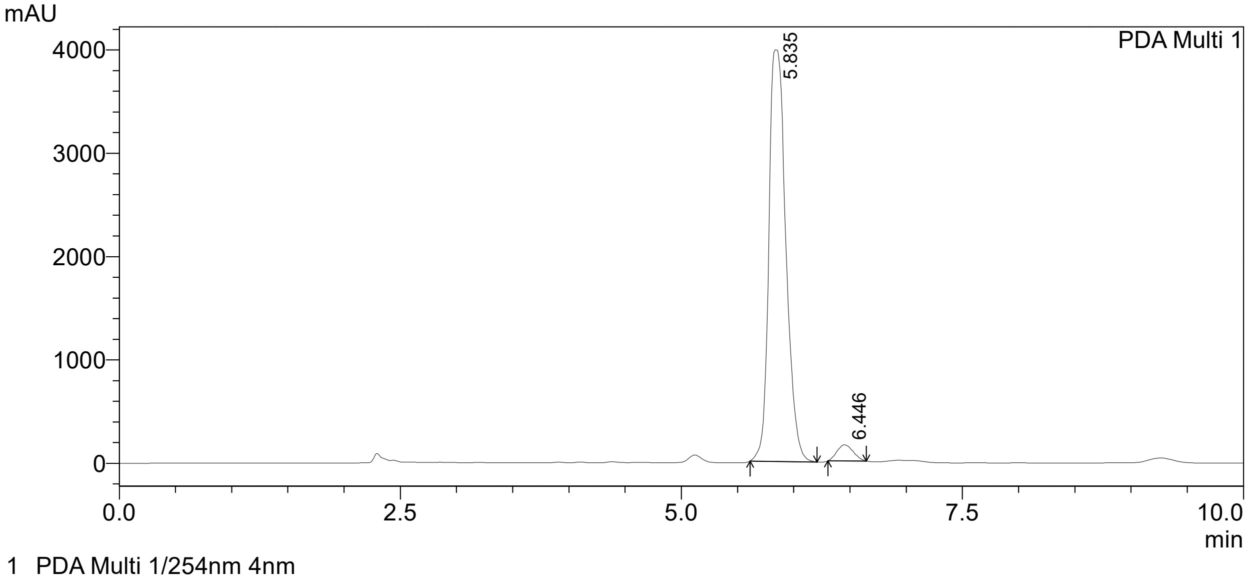

1 PDA Multi 1/254nm 4nm

PeakTable

PDA Ch1 254nm 4nm

Peak#	Ret. Time	Area	Height	Area %	Height %
1	5.835	41906706	3981304	96.408	96.234
2	6.446	1561406	155820	3.592	3.766
Total		43468112	4137123	100.000	100.000

==== Shimadzu LCsolution Analysis Report ====

C:\Users\user\Desktop\PoKai\L-003-157-90%-3-20min-1.0.lcd

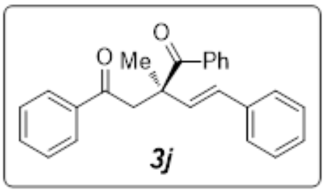

Acquired by	: Admin
Sample Name	: L-003-157-90%-3-20min
Sample ID	: L-003-157-90%-3-20min
Tray#	: 1
Vail #	: 1
Injection Volume	: 10 uL
Data File Name	: L-003-157-90%-3-20min-1.0.lcd
Method File Name	: pos3-90%_20MIN_1_d2.lcm
Batch File Name	: Batch table C3 _90%_20min_1.0_D2.lcb
Report File Name	: Default.lcr
Data Acquired	: 11/22/2021 12:28:21 PM
Data Processed	: 11/22/2021 1:22:26 PM

<Chromatogram>

C:\Users\user\Desktop\PoKai\L-003-157-90%-3-20min-1.0.lcd

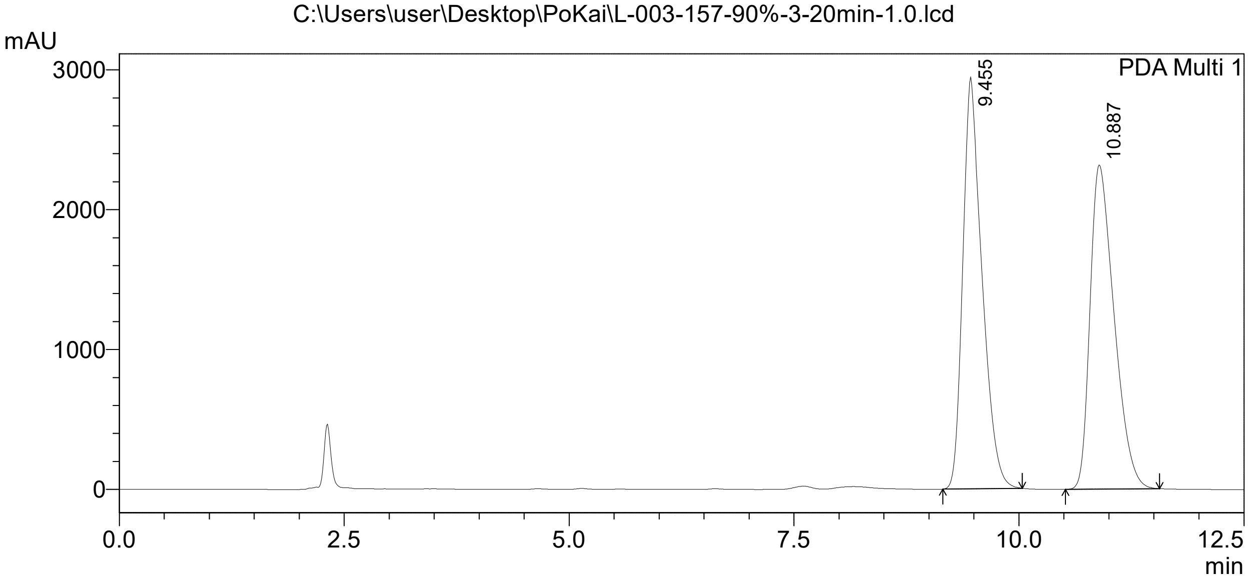

1 PDA Multi 1/254nm 4nm

PeakTable

PDA Ch1 254nm 4nm

Peak#	Ret. Time	Area	Height	Area %	Height %
1	9.455	44075180	2945872	50.598	55.956
2	10.887	43033963	2318738	49.402	44.044
Total		87109143	5264610	100.000	100.000

C:\Users\user\Desktop\PoKai\L-003-224-90%-3-20min-1.0.lcd

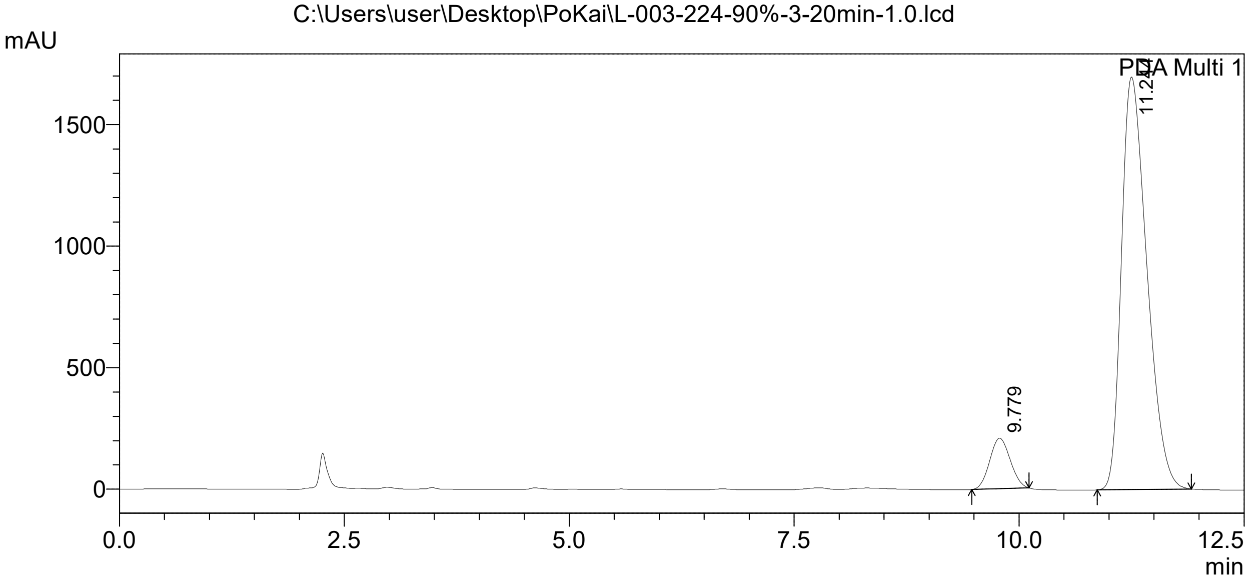

1 PDA Multi 1/254nm 4nm

PeakTable

PDA Ch1 254nm 4nm

Peak#	Ret. Time	Area	Height	Area %	Height %
1	9.779	3303308	207838	9.002	10.908
2	11.244	33389974	1697615	90.998	89.092
Total		36693282	1905453	100.000	100.000

==== Shimadzu LCsolution Analysis Report ====

C:\Users\user\Desktop\PoKai\L-002-177-90%-1.0-6.lcd

Acquired by	: Admin
Sample Name	: L-002-177-6-90%-1.0
Sample ID	: L-002-177
Tray#	: 1
Vail #	: 1
Injection Volume	: 10 uL
Data File Name	: L-002-177-90%-1.0-6.lcd
Method File Name	: pos6-90%_20min_1.0.lcm
Batch File Name	: Batch_table_C6-90_20min 1.0.lcb
Report File Name	: Default.lcr
Data Acquired	: 7/7/2021 2:00:14 PM
Data Processed	: 7/7/2021 2:20:16 PM

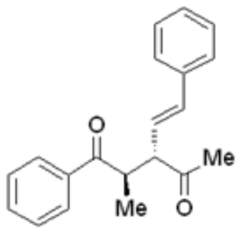

5h

<Chromatogram>

C:\Users\user\Desktop\PoKai\L-002-177-90%-1.0-6.lcd

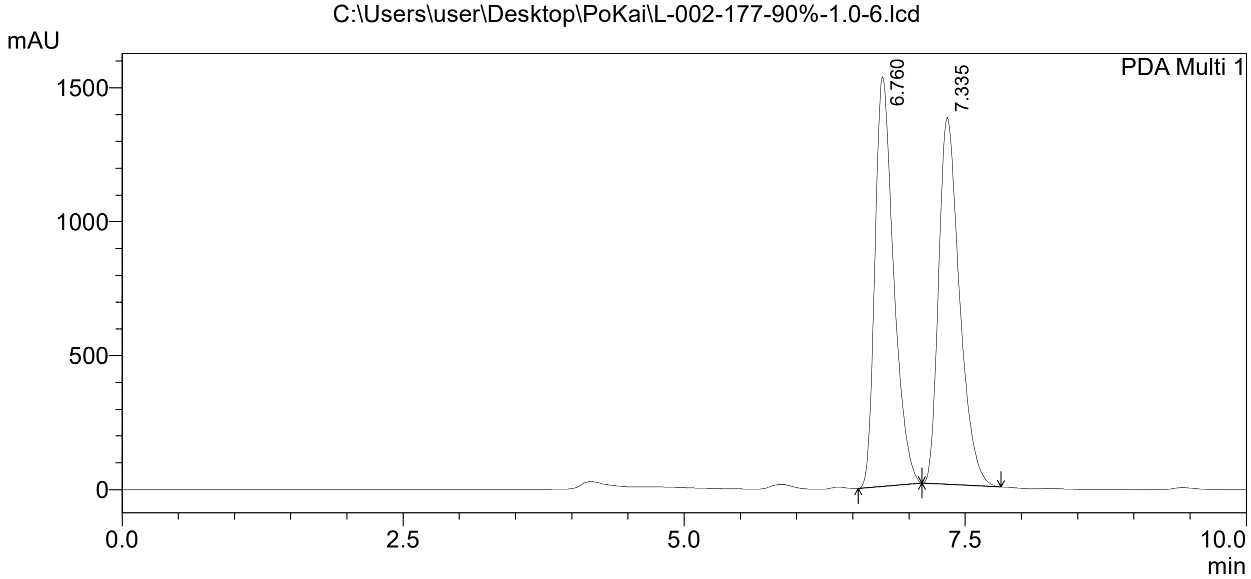

1 PDA Multi 1/254nm 4nm

PeakTable

PDA Ch1 254nm 4nm

Peak#	Ret. Time	Area	Height	Area %	Height %
1	6.760	17735520	1529242	50.069	52.754
2	7.335	17686423	1369550	49.931	47.246
Total		35421944	2898792	100.000	100.000

C:\Users\user\Desktop\PoKai\L-002-265-90%-1.0-6.lcd

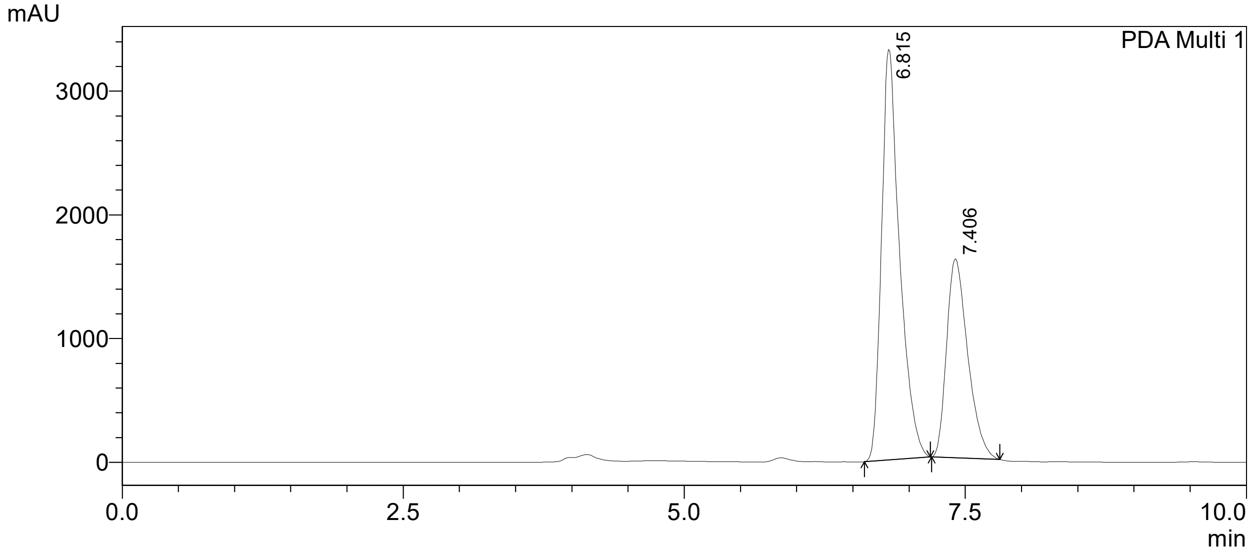

1 PDA Multi 1/254nm 4nm

PeakTable

PDA Ch1 254nm 4nm

Peak#	Ret. Time	Area	Height	Area %	Height %
1	6.815	37325877	3318163	63.901	67.363
2	7.406	21085714	1607643	36.099	32.637
Total		58411591	4925806	100.000	100.000

==== Shimadzu LCsolution Analysis Report ====

C:\Users\user\Desktop\PoKai\L-002-233-90%-3-20min-1.0.lcd

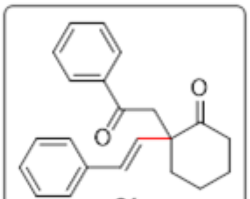

3l

Acquired by	: Admin
Sample Name	: L-002-233-90%-3-20min
Sample ID	: L-002-233-90%-3-20min
Tray#	: 1
Vail #	: 1
Injection Volume	: 10 uL
Data File Name	: L-002-233-90%-3-20min-1.0.lcd
Method File Name	: pos3-90%_20MIN_1_d2.lcm
Batch File Name	: Batch table C3 _90%_20min_1.0_D2.lcb
Report File Name	: Default.lcr
Data Acquired	: 6/16/2021 2:42:17 PM
Data Processed	: 6/16/2021 3:04:00 PM

<Chromatogram>

C:\Users\user\Desktop\PoKai\L-002-233-90%-3-20min-1.0.lcd

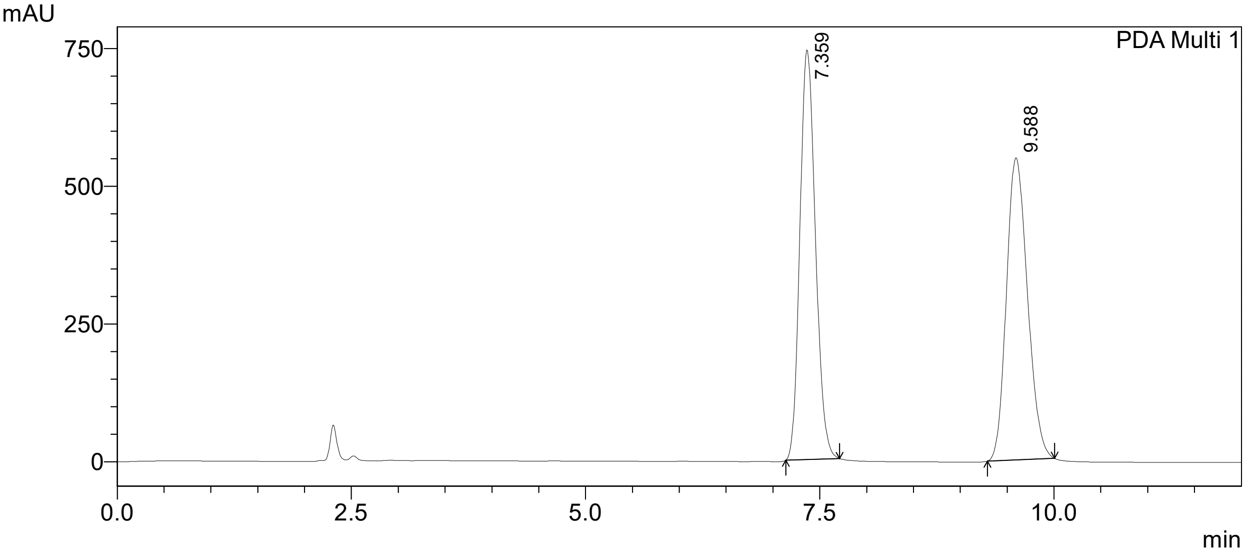

1 PDA Multi 1/254nm 4nm

PeakTable

PDA Ch1 254nm 4nm

Peak#	Ret. Time	Area	Height	Area %	Height %
1	7.359	8611276	743667	50.042	57.560
2	9.588	8596692	548309	49.958	42.440
Total		17207968	1291976	100.000	100.000

C:\Users\user\Desktop\PoKai\L-002-232-90%-3-20min-1.0.lcd

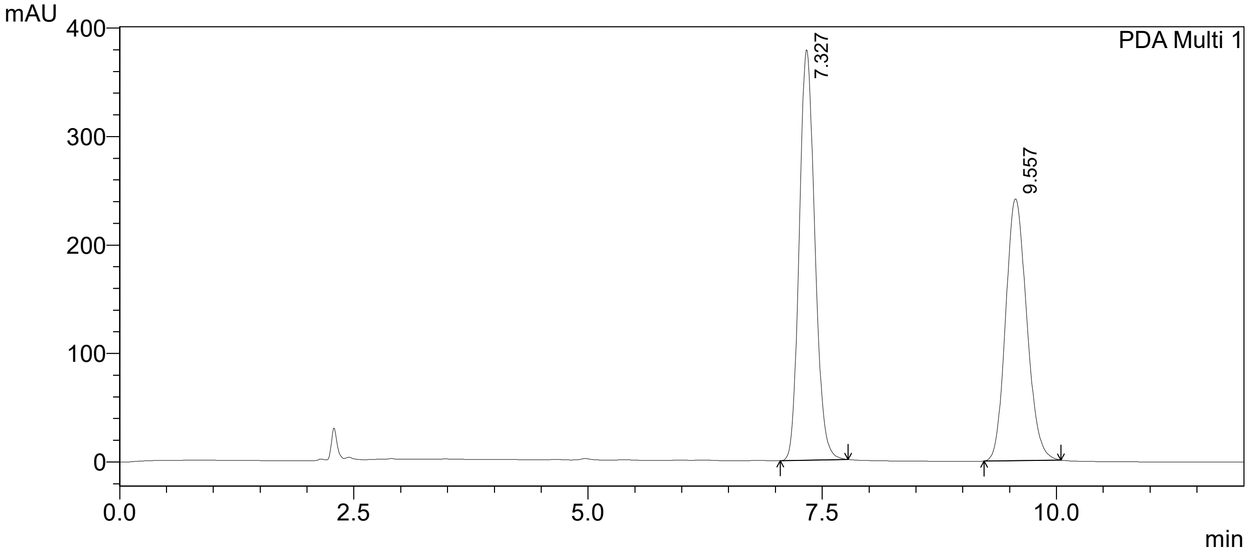

1 PDA Multi 1/254nm 4nm

PeakTable

PDA Ch1 254nm 4nm

Peak#	Ret. Time	Area	Height	Area %	Height %
1	7.327	4397072	378357	53.724	61.071
2	9.557	3787502	241177	46.276	38.929
Total		8184574	619534	100.000	100.000

==== Shimadzu LCsolution Analysis Report ====

C:\Users\user\Desktop\PoKai\L-003-126-90%-3-20min-1.0.lcd

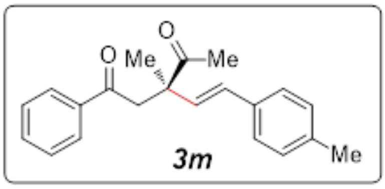

3m

Acquired by	: Admin
Sample Name	: L-003-126-90%-3-20min
Sample ID	: L-003-126-90%-3-20min
Tray#	: 1
Vail #	: 1
Injection Volume	: 10 uL
Data File Name	: L-003-126-90%-3-20min-1.0.lcd
Method File Name	: pos3-90%_20MIN_1_d2.lcm
Batch File Name	: Batch table C3 _90%_20min_1.0_D2.lcb
Report File Name	: Default.lcr
Data Acquired	: 11/2/2021 10:00:06 AM
Data Processed	: 11/2/2021 10:20:08 AM

<Chromatogram>

C:\Users\user\Desktop\PoKai\L-003-126-90%-3-20min-1.0.lcd

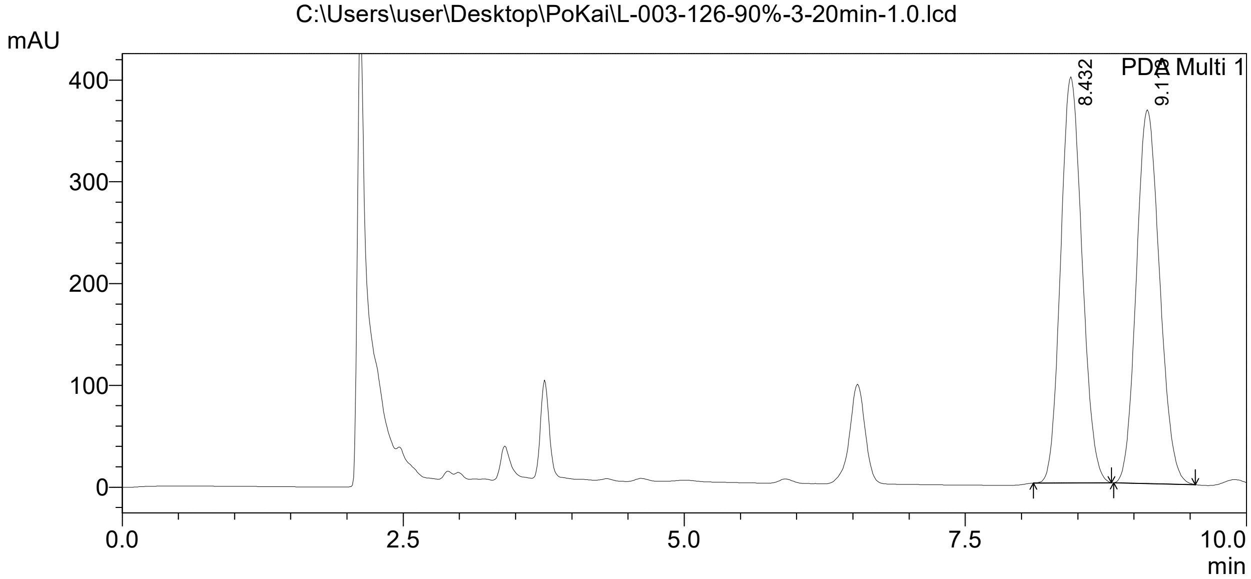

1 PDA Multi 1/254nm 4nm

PeakTable

PDA Ch1 254nm 4nm

Peak#	Ret. Time	Area	Height	Area %	Height %
1	8.432	5313933	399093	50.076	52.064
2	9.113	5297722	367443	49.924	47.936
Total		10611655	766536	100.000	100.000

C:\Users\user\Desktop\PoKai\L-003-146-90%-3-10min-1.0.lcd

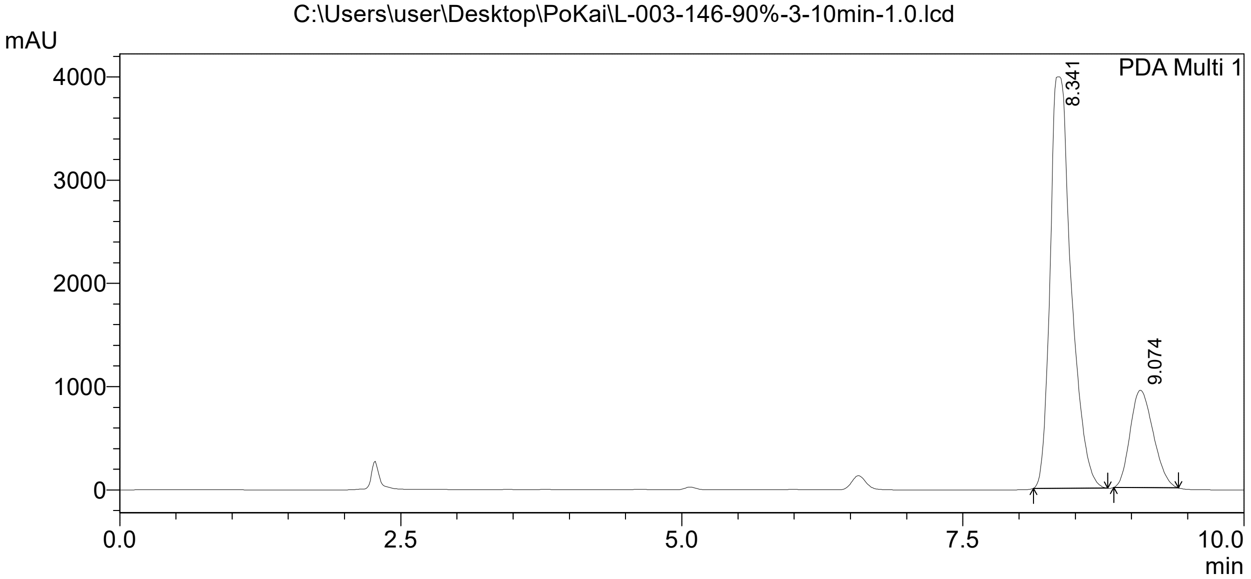

1 PDA Multi 1/254nm 4nm

PeakTable

PDA Ch1 254nm 4nm

Peak#	Ret. Time	Area	Height	Area %	Height %
1	8.341	51977922	3984931	79.299	80.824
2	9.074	13568997	945424	20.701	19.176
Total		65546919	4930355	100.000	100.000

==== Shimadzu LCsolution Analysis Report ====

C:\Users\user\Desktop\PoKai\L-003-106-90%-3-30min-1.0.lcd

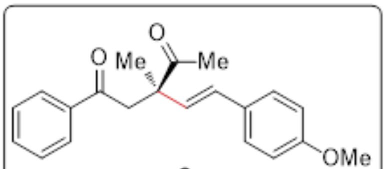

3n

Acquired by	: Admin
Sample Name	: L-003-106-90%-3-30min
Sample ID	: L-003-106-90%-3-30min
Tray#	: 1
Vail #	: 1
Injection Volume	: 10 uL
Data File Name	: L-003-106-90%-3-30min-1.0.lcd
Method File Name	: pos3-90%_30MIN_1_d2.lcm
Batch File Name	: Batch table C3 _90%_30min_1.0_D2.lcb
Report File Name	: Default.lcr
Data Acquired	: 11/17/2021 12:15:06 PM
Data Processed	: 11/17/2021 12:36:25 PM

<Chromatogram>

C:\Users\user\Desktop\PoKai\L-003-106-90%-3-30min-1.0.lcd

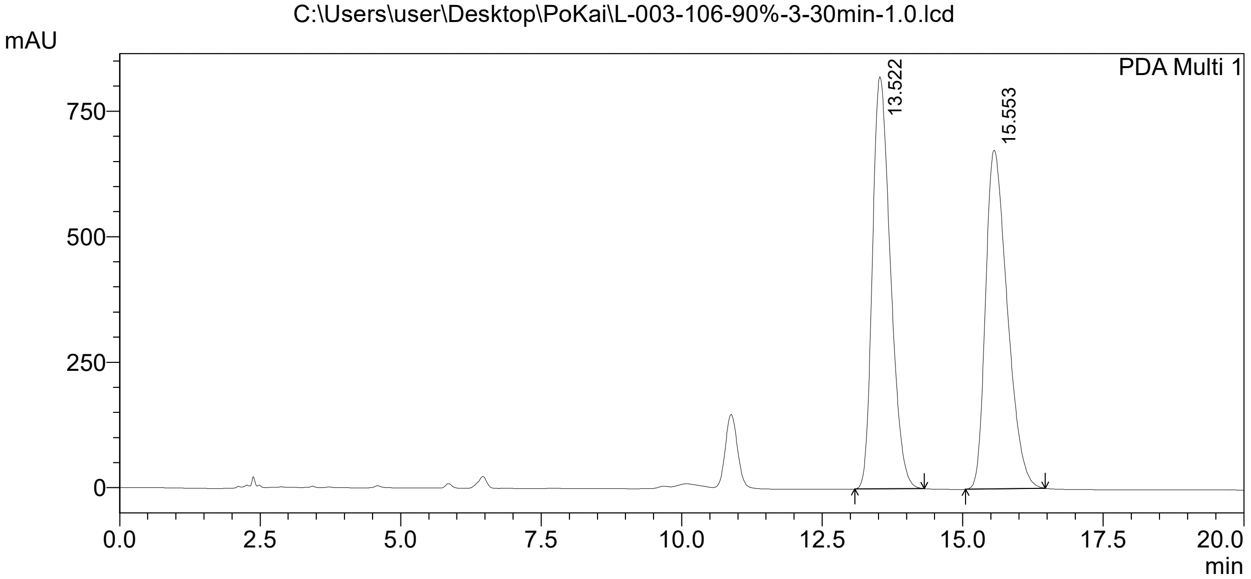

1 PDA Multi 1/254nm 4nm

PeakTable

PDA Ch1 254nm 4nm

Peak#	Ret. Time	Area	Height	Area %	Height %
1	13.522	18339469	820899	50.196	54.893
2	15.553	18196107	674560	49.804	45.107
Total		36535576	1495459	100.000	100.000

C:\Users\user\Desktop\PoKai\L-003-144-90%-3-30min-1.0.lcd

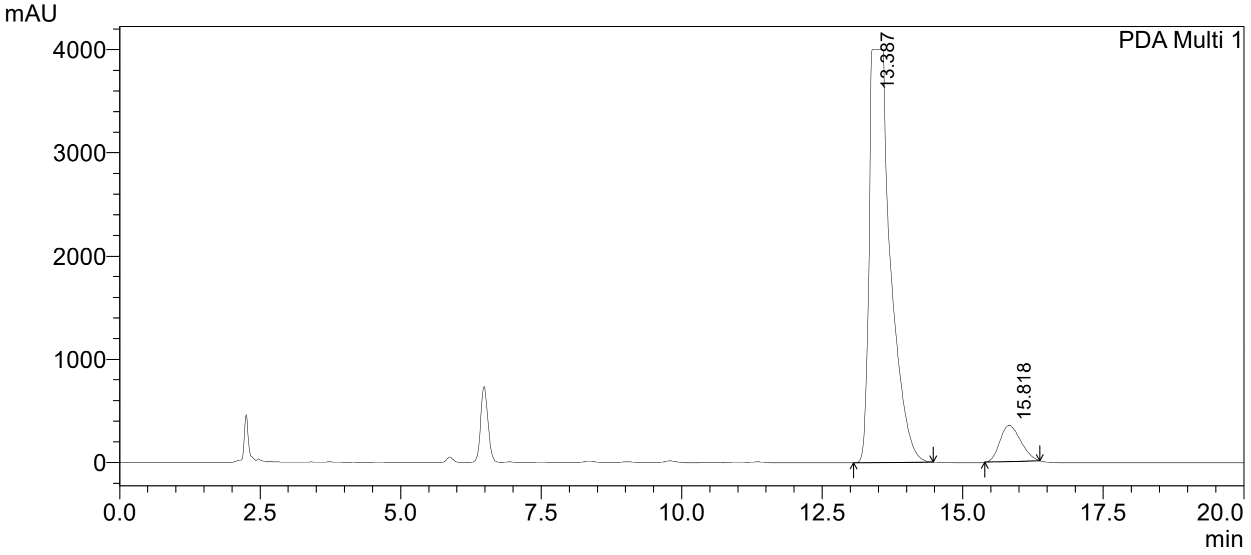

1 PDA Multi 1/254nm 4nm

PeakTable

PDA Ch1 254nm 4nm

Peak#	Ret. Time	Area	Height	Area %	Height %
1	13.387	105601360	4000560	92.111	91.974
2	15.818	9044360	349117	7.889	8.026
Total		114645720	4349677	100.000	100.000

==== Shimadzu LCsolution Analysis Report ====

C:\Users\user\Desktop\PoKai\L-002-304-92%-3-20min.lcd

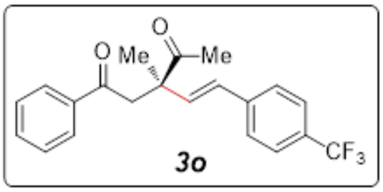

Acquired by	: Admin
Sample Name	: L-002-304-92%-3-20min
Sample ID	: L-002-304-92%-3-20min
Tray#	: 1
Vail #	: 1
Injection Volume	: 1 uL
Data File Name	: L-002-304-92%-3-20min.lcd
Method File Name	: pos3-92%_20MIN_1.0_D2.lcm
Batch File Name	: Batch table C3 _92%_20min_1.0_D2.lcb
Report File Name	: Default.lcr
Data Acquired	: 8/2/2021 4:07:02 PM
Data Processed	: 8/2/2021 4:27:04 PM

<Chromatogram>

C:\Users\user\Desktop\PoKai\L-002-304-92%-3-20min.lcd

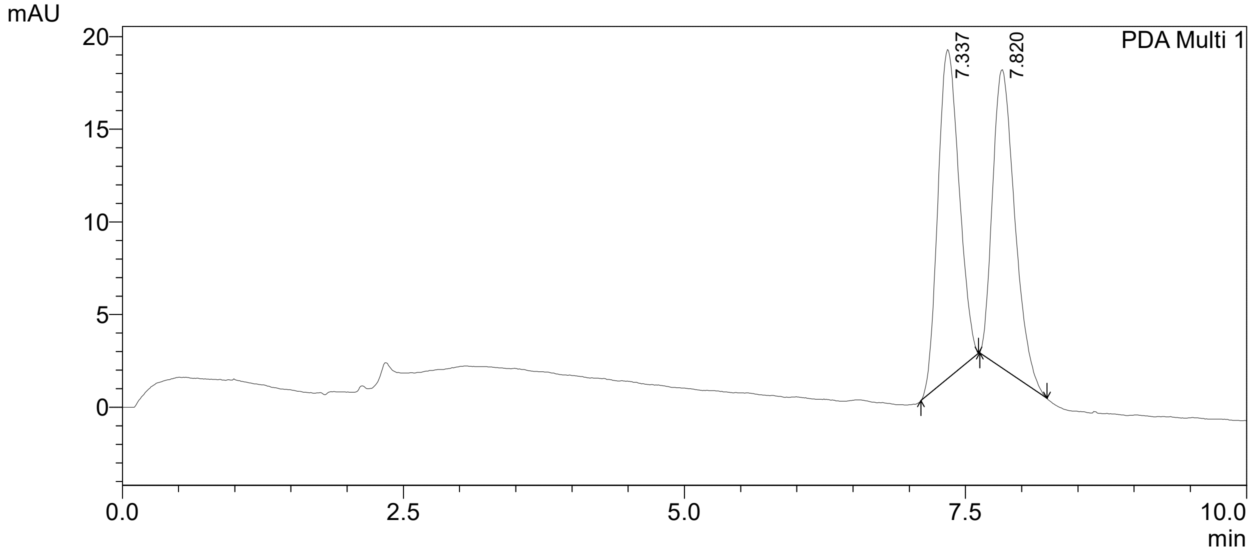

1 PDA Multi 1/254nm 4nm

PeakTable

PDA Ch1 254nm 4nm

Peak#	Ret. Time	Area	Height	Area %	Height %
1	7.337	233592	17779	51.426	52.536
2	7.820	220635	16063	48.574	47.464
Total		454227	33842	100.000	100.000

C:\Users\user\Desktop\PoKai\L-002-301-92%-3-20min.lcd

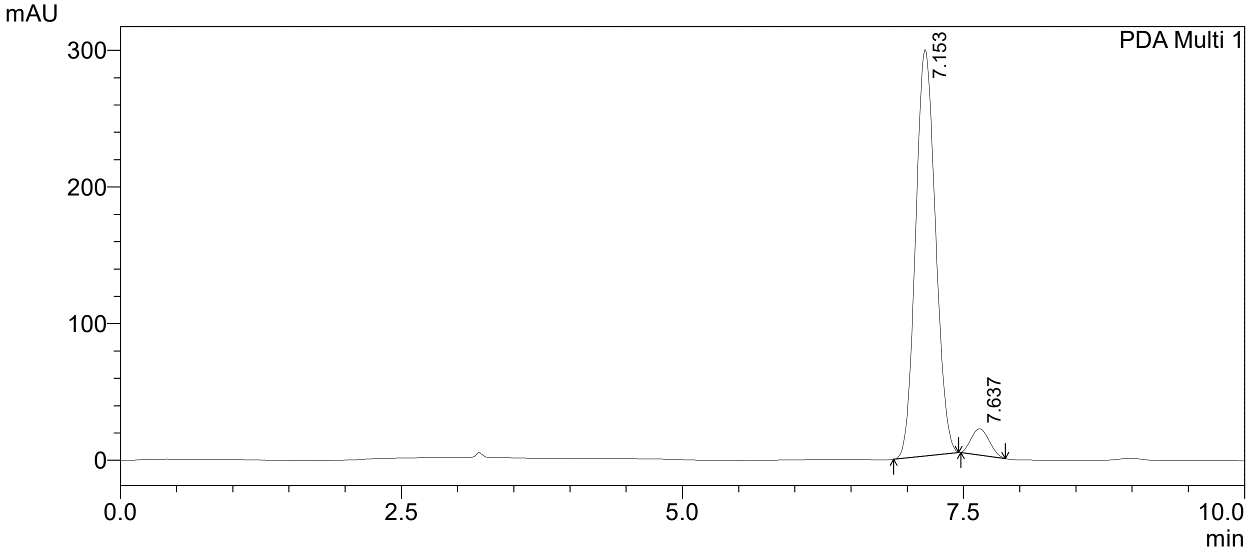

1 PDA Multi 1/254nm 4nm

PeakTable

PDA Ch1 254nm 4nm

Peak#	Ret. Time	Area	Height	Area %	Height %
1	7.153	3657549	297359	94.454	93.939
2	7.637	214777	19185	5.546	6.061
Total		3872326	316544	100.000	100.000

==== Shimadzu LCsolution Analysis Report ====

C:\Users\user\Desktop\PoKai\L-003-011-99%-2-20min.lcd

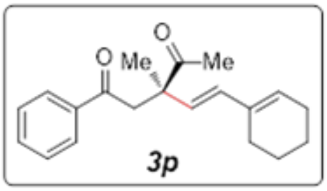

Acquired by	: Admin
Sample Name	: L-003-011-99%-2-20min-1
Sample ID	: L-003-011-99%-2-20min
Tray#	: 1
Vail #	: 1
Injection Volume	: 10 uL
Data File Name	: L-003-011-99%-2-20min.lcd
Method File Name	: pos2-99%_20min_1_D2.lcm
Batch File Name	: Batch table C2_99%_20min_1_D2.lcb
Report File Name	: Default.lcr
Data Acquired	: 8/13/2021 12:35:44 PM
Data Processed	: 8/13/2021 12:55:46 PM

<Chromatogram>

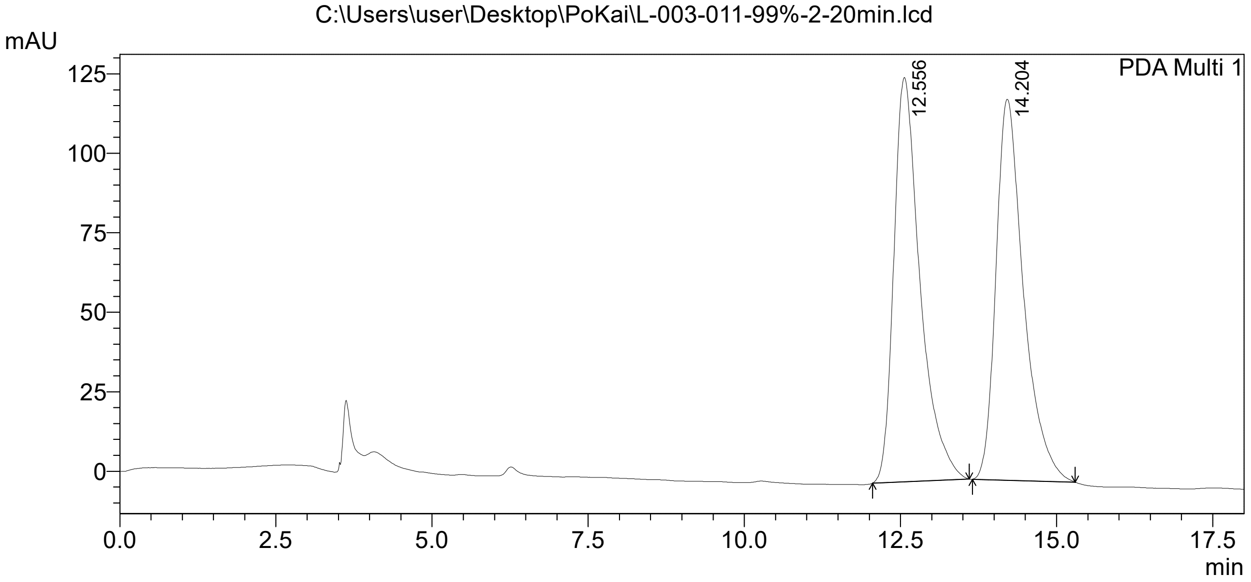

1 PDA Multi 1/254nm 4nm

PeakTable

PDA Ch1 254nm 4nm

Peak#	Ret. Time	Area	Height	Area %	Height %
1	12.556	3728345	127173	50.228	51.486
2	14.204	3694512	119833	49.772	48.514
Total		7422856	247007	100.000	100.000

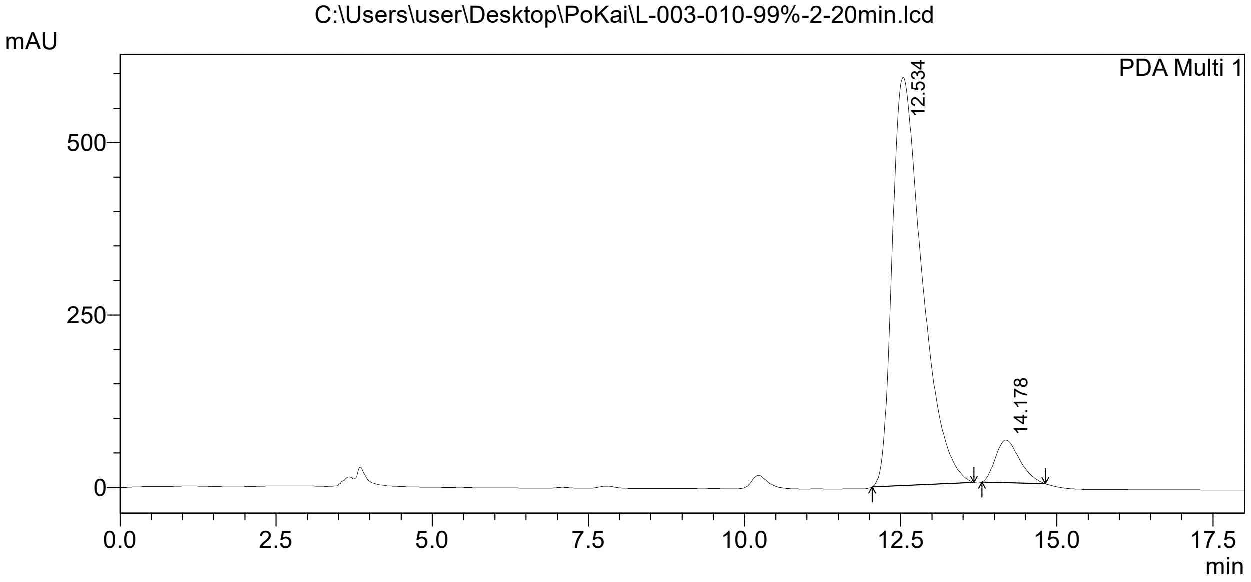

1 PDA Multi 1/254nm 4nm

PeakTable

PDA Ch1 254nm 4nm

Peak#	Ret. Time	Area	Height	Area %	Height %
1	12.534	19746522	591994	92.151	90.561
2	14.178	1682012	61699	7.849	9.439
Total		21428534	653693	100.000	100.000

==== Shimadzu LCsolution Analysis Report ====

C:\Users\user\Desktop\PoKai\L-003-298-92%-0.7-6.lcd

Acquired by	: Admin
Sample Name	: L-003-298-6-92%-0.7
Sample ID	: L-003-298-6-92%-0.7
Tray#	: 1
Vail #	: 1
Injection Volume	: 10 uL
Data File Name	: L-003-298-92%-0.7-6.lcd
Method File Name	: pos6-92%_20min_0.7.lcm
Batch File Name	: Batch_table_C6-92_20min 1.0.lcb
Report File Name	: Default.lcr
Data Acquired	: 6/22/2022 10:49:22 AM
Data Processed	: 6/22/2022 11:01:31 AM

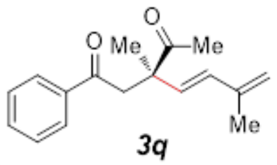

3q

<Chromatogram>

C:\Users\user\Desktop\PoKai\L-003-298-92%-0.7-6.lcd

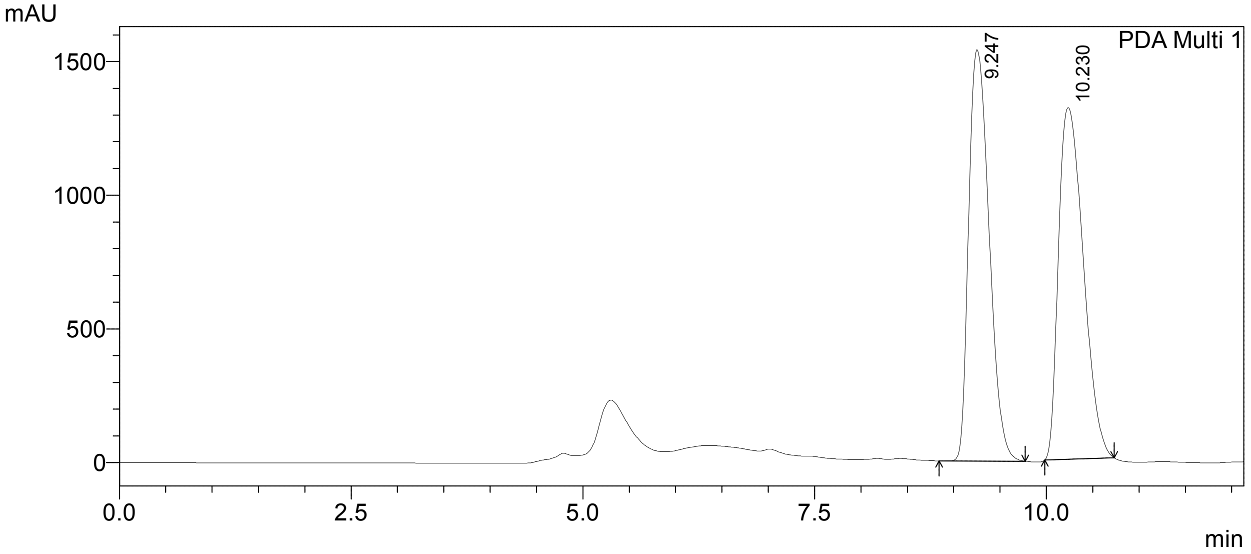

1 PDA Multi 1/254nm 4nm

PeakTable

PDA Ch1 254nm 4nm

Peak#	Ret. Time	Area	Height	Area %	Height %
1	9.247	23652251	1538504	49.108	53.908
2	10.230	24511600	1315439	50.892	46.092
Total		48163851	2853943	100.000	100.000

C:\Users\user\Desktop\PoKai\L-003-293-92%-0.7-6.lcd

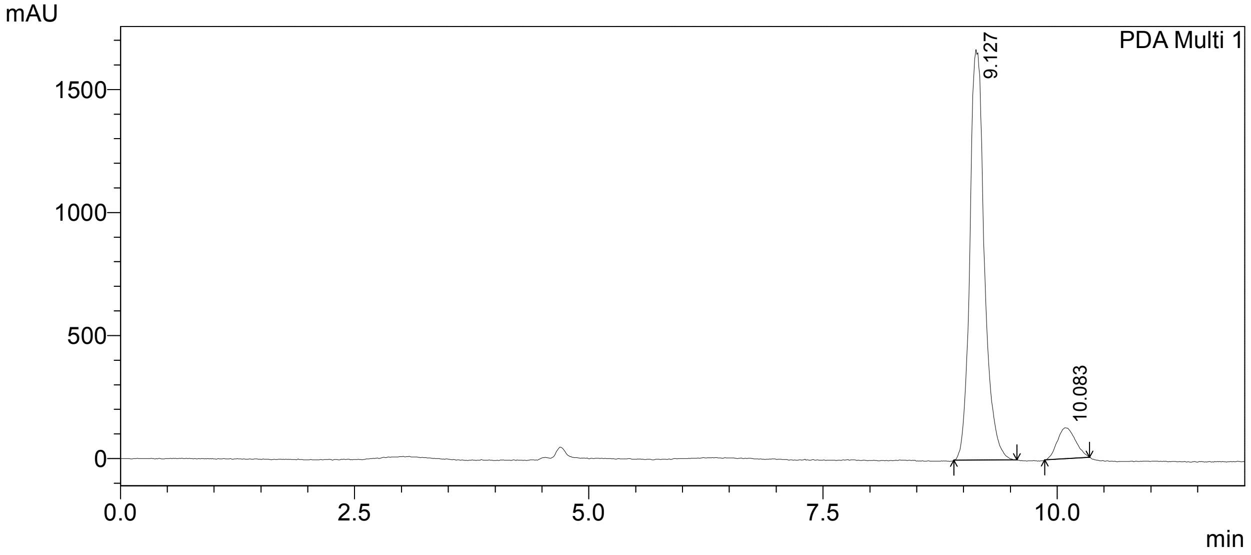

1 PDA Multi 1/190nm 4nm

PeakTable

PDA Ch1 190nm 4nm

Peak#	Ret. Time	Area	Height	Area %	Height %
1	9.127	18083199	1668478	91.365	92.971
2	10.083	1709035	126154	8.635	7.029
Total		19792234	1794632	100.000	100.000

==== Shimadzu LCsolution Analysis Report ====

C:\Users\user\Desktop\PoKai\L-002-158-98%-5-1.0-10min-D2.lcd

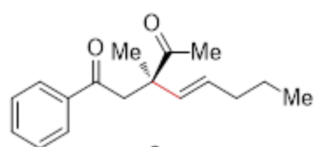

3r

Acquired by	: Admin
Sample Name	: L-002-158-98%-5-1.0-30min-D2
Sample ID	: L-002-158-98%-5-1.0-30min-D2
Tray#	: 1
Vail #	: 1
Injection Volume	: 10 uL
Data File Name	: L-002-158-98%-5-1.0-10min-D2.lcd
Method File Name	: pos5_98%_10min_1.lcm
Batch File Name	: Batch_table_C5-98_10min_1.0.lcb
Report File Name	: Default.lcr
Data Acquired	: 4/29/2021 4:57:30 PM
Data Processed	: 4/29/2021 5:07:33 PM

<Chromatogram>

C:\Users\user\Desktop\PoKai\L-002-158-98%-5-1.0-10min-D2.lcd

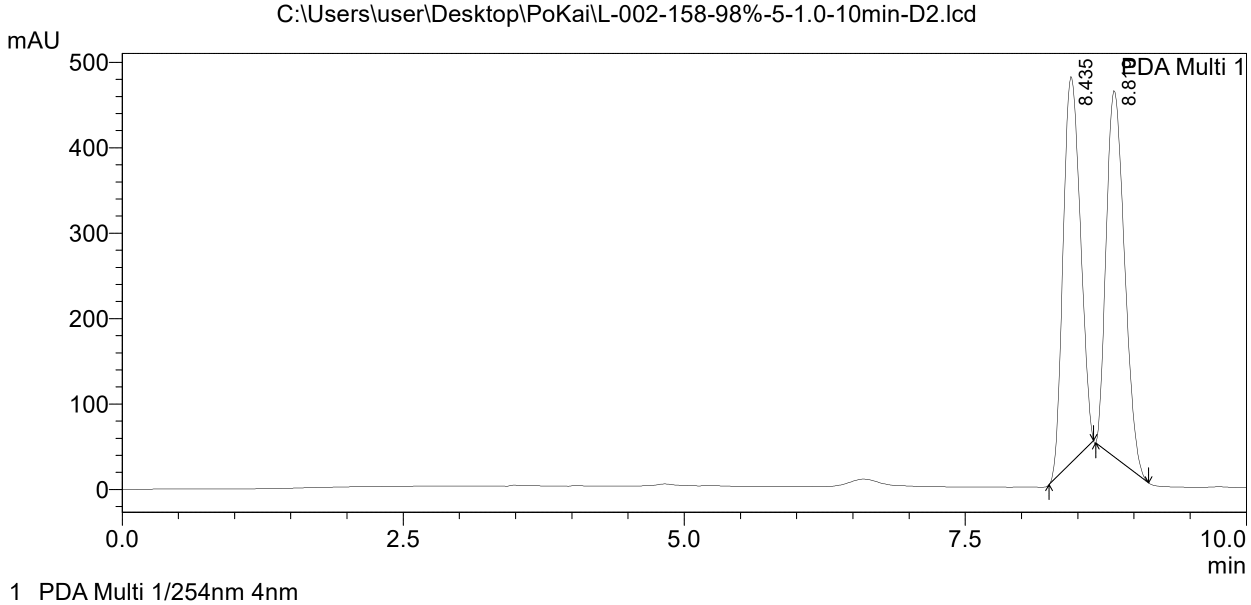

1 PDA Multi 1/254nm 4nm

PeakTable

PDA Ch1 254nm 4nm

Peak#	Ret. Time	Area	Height	Area %	Height %
1	8.435	4828420	452713	49.969	51.392
2	8.819	4834350	428186	50.031	48.608
Total		9662769	880899	100.000	100.000

C:\Users\user\Desktop\PoKai\L-003-246-99%-5-1.0-20min-D2.lcd

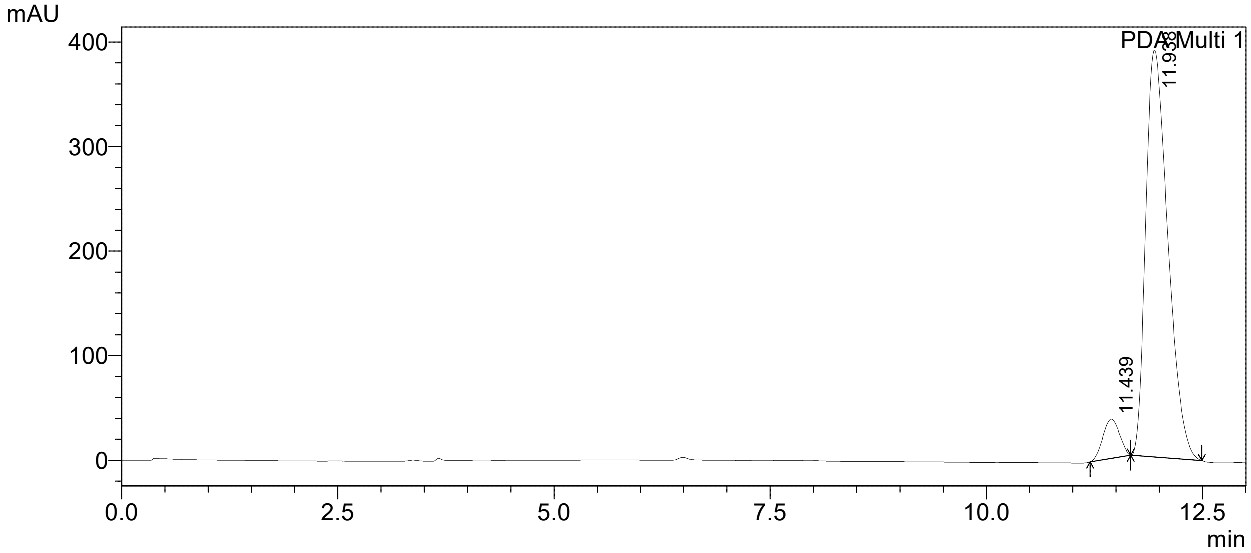

1 PDA Multi 1/254nm 4nm

PeakTable

PDA Ch1 254nm 4nm

Peak#	Ret. Time	Area	Height	Area %	Height %
1	11.439	505581	37738	6.790	8.838
2	11.938	6940851	389266	93.210	91.162
Total		7446432	427004	100.000	100.000

==== Shimadzu LCsolution Analysis Report ====

C:\Users\user\Desktop\PoKai\L-003-296-1-99%-5-1.0-20min-D2.lcd

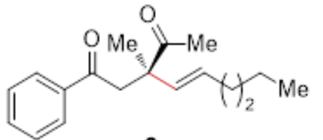

3s

Acquired by	: Admin
Sample Name	: L-003-296-1-99%-5-1.0-20min-D2
Sample ID	: L-003-296-1-99%-5-1.0-20min-D2
Tray#	: 1
Vail #	: 1
Injection Volume	: 10 uL
Data File Name	: L-003-296-1-99%-5-1.0-20min-D2.lcd
Method File Name	: pos5_99%_20min_1.0_D2.lcm
Batch File Name	: Batch_table_C5_99%_20min_1.0.lcb
Report File Name	: Default.lcr
Data Acquired	: 6/21/2022 4:46:28 PM
Data Processed	: 6/21/2022 4:58:38 PM

<Chromatogram>

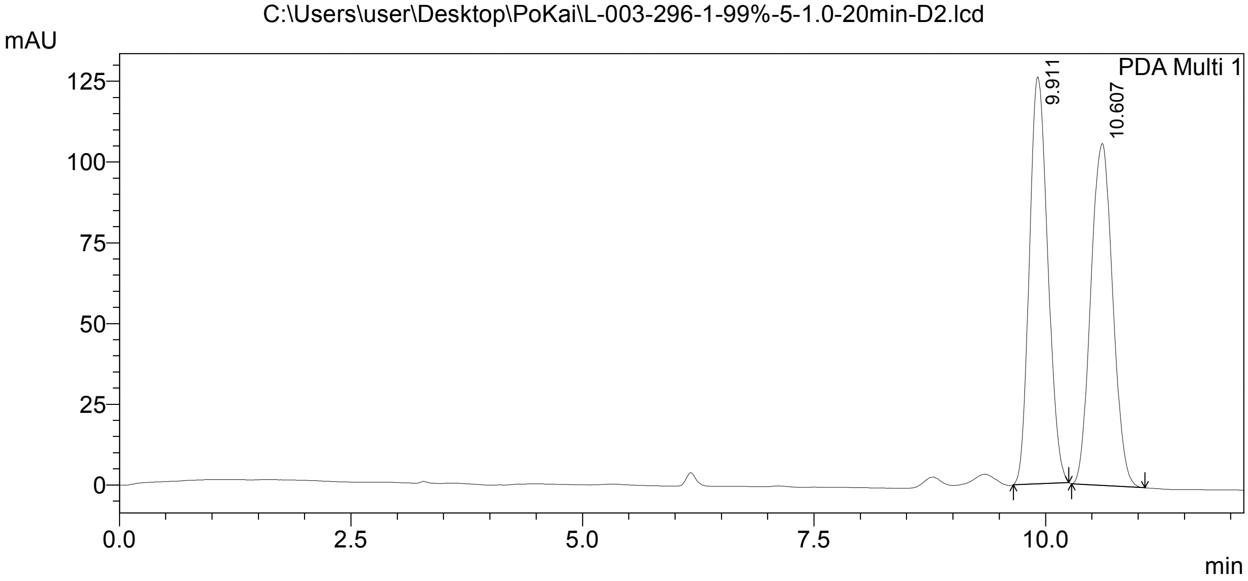

1 PDA Multi 1/254nm 4nm

PeakTable

PDA Ch1 254nm 4nm

Peak#	Ret. Time	Area	Height	Area %	Height %
1	9.911	1742578	126017	49.975	54.329
2	10.607	1744311	105934	50.025	45.671
Total		3486889	231950	100.000	100.000

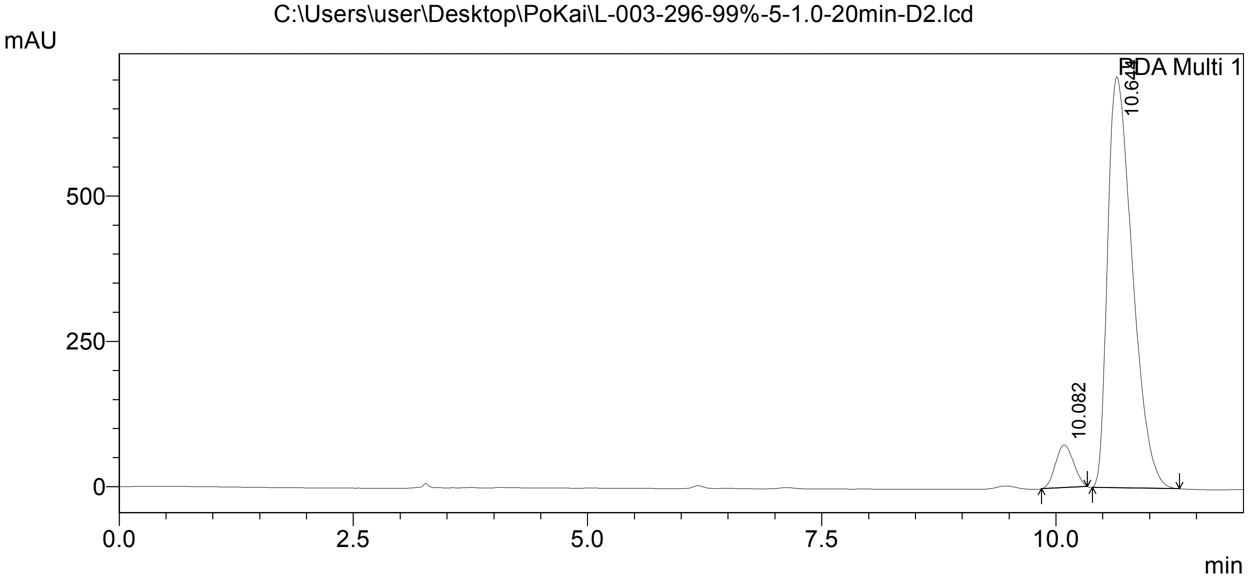

1 PDA Multi 1/254nm 4nm

PeakTable

PDA Ch1 254nm 4nm

Peak#	Ret. Time	Area	Height	Area %	Height %
1	10.082	986425	73353	7.039	9.404
2	10.644	13027776	706696	92.961	90.596
Total		14014201	780049	100.000	100.000

==== Shimadzu LCsolution Analysis Report ====

C:\Users\user\Desktop\PoKai\L-003-295-1-99%-5-1.0-20min-D2.lcd

Acquired by	: Admin
Sample Name	: L-003-295-1-99%-5-1.0-20min-D2
Sample ID	: L-003-295-1-99%-5-1.0-20min-D2
Tray#	: 1
Vail #	: 1
Injection Volume	: 10 uL
Data File Name	: L-003-295-1-99%-5-1.0-20min-D2.lcd
Method File Name	: pos5_99%_20min_1.0_D2.lcm
Batch File Name	: Batch_table_C5_99%_20min_1.0.lcb
Report File Name	: Default.lcr
Data Acquired	: 6/20/2022 3:09:34 PM
Data Processed	: 6/20/2022 3:28:07 PM

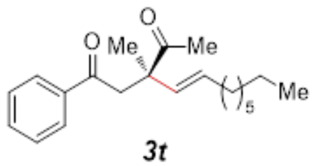

3t

<Chromatogram>

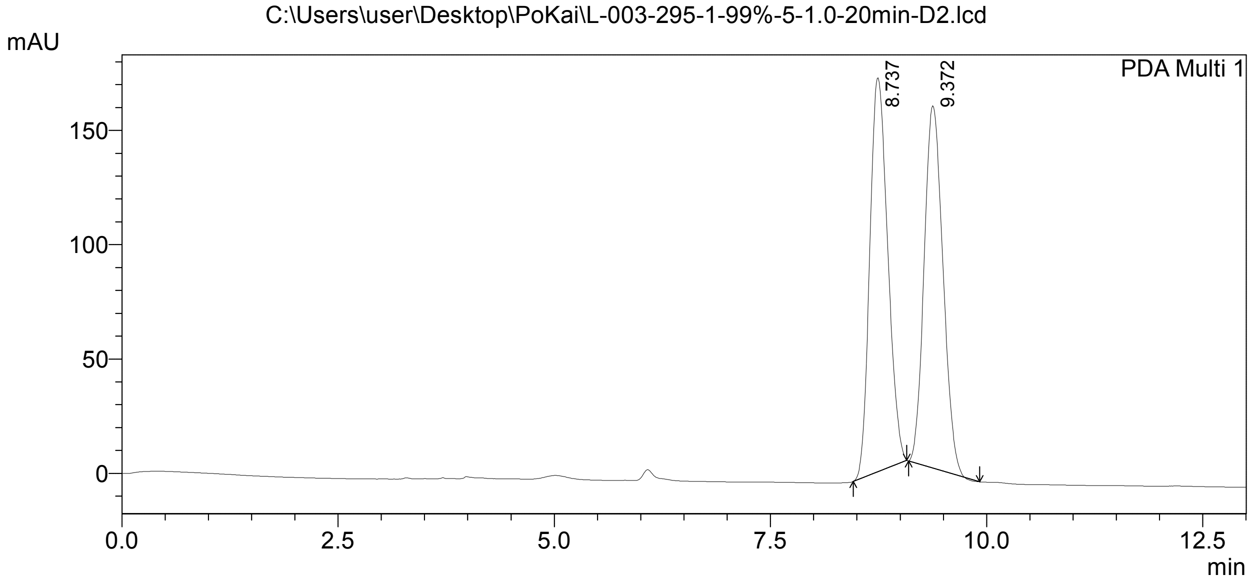

1 PDA Multi 1/254nm 4nm

PeakTable

PDA Ch1 254nm 4nm

Peak#	Ret. Time	Area	Height	Area %	Height %
1	8.737	2495193	172345	50.981	52.102
2	9.372	2399158	158437	49.019	47.898
Total		4894351	330782	100.000	100.000

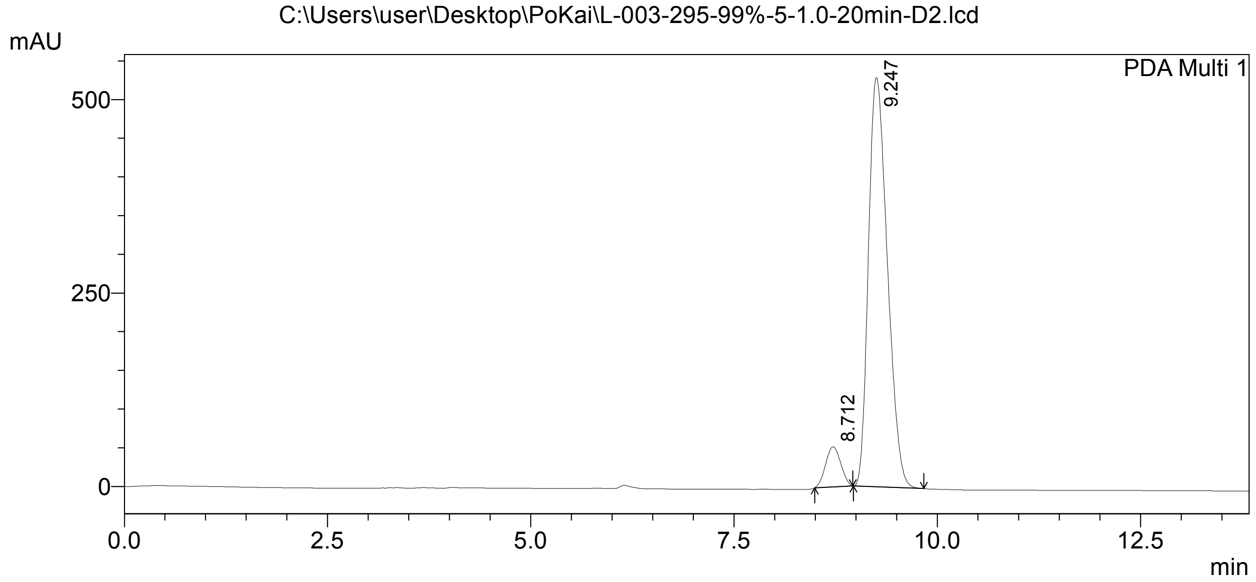

1 PDA Multi 1/254nm 4nm

PeakTable

PDA Ch1 254nm 4nm

Peak#	Ret. Time	Area	Height	Area %	Height %
1	8.712	671886	51829	7.265	8.927
2	9.247	8576160	528770	92.735	91.073
Total		9248046	580599	100.000	100.000

==== Shimadzu LCsolution Analysis Report ====

C:\Users\user\Desktop\PoKai\L-003-291-1-98%-2-1.0.lcd

Acquired by	: Admin
Sample Name	: L-003-291-1-98%-2-1.0
Sample ID	: L-003-291-1-98%-2-1.0
Tray#	: 1
Vail #	: 1
Injection Volume	: 5 uL
Data File Name	: L-003-291-1-98%-2-1.0.lcd
Method File Name	: pos2-98%_10min_1_D2.lcm
Batch File Name	: Batch table C2_98%_10min_1_D2.lcb
Report File Name	: Default.lcr
Data Acquired	: 6/19/2022 3:36:34 PM
Data Processed	: 6/19/2022 3:56:36 PM

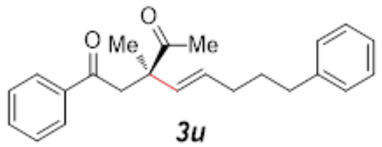

3u

<Chromatogram>

C:\Users\user\Desktop\PoKai\L-003-291-1-98%-2-1.0.lcd

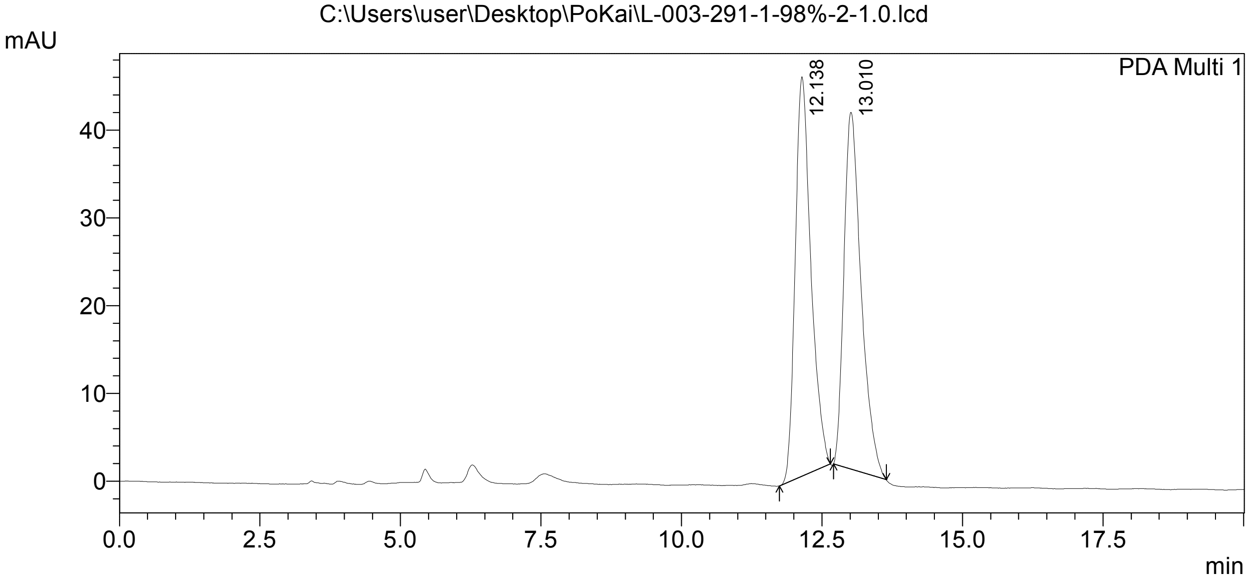

1 PDA Multi 1/254nm 4nm

PeakTable

PDA Ch1 254nm 4nm

Peak#	Ret. Time	Area	Height	Area %	Height %
1	12.138	887868	45533	50.585	52.827
2	13.010	867335	40659	49.415	47.173
Total		1755203	86193	100.000	100.000

C:\Users\user\Desktop\PoKai\L-003-291-98%-2-1.0.lcd

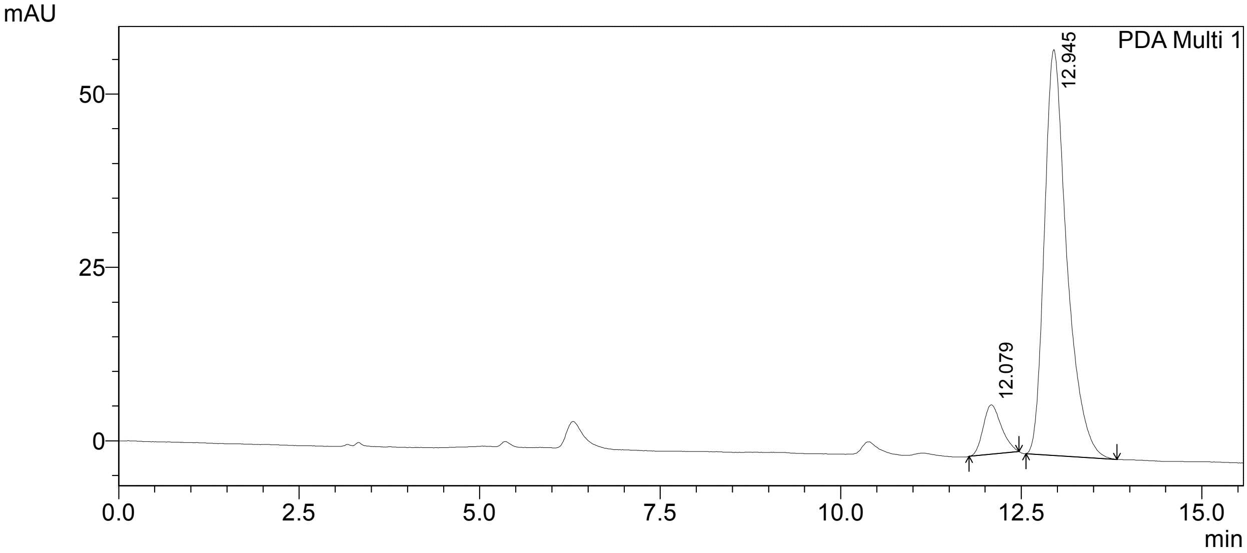

1 PDA Multi 1/254nm 4nm

PeakTable

PDA Ch1 254nm 4nm

Peak#	Ret. Time	Area	Height	Area %	Height %
1	12.079	125948	7147	9.042	10.886
2	12.945	1266964	58509	90.958	89.114
Total		1392912	65656	100.000	100.000

==== Shimadzu LCsolution Analysis Report ====

C:\Users\user\Desktop\PoKai\L-003-285-99%-5-1.0-20min-D2.lcd

Acquired by	: Admin
Sample Name	: L-003-285-99%-5-1.0-20min-D2
Sample ID	: L-003-285-99%-5-1.0-20min-D2
Tray#	: 1
Vail #	: 1
Injection Volume	: 10 uL
Data File Name	: L-003-285-99%-5-1.0-20min-D2.lcd
Method File Name	: pos5_99%_20min_1.0_D2.lcm
Batch File Name	: Batch_table_C5_99%_10min_1.0.lcb
Report File Name	: Default.lcr
Data Acquired	: 6/16/2022 11:34:41 AM
Data Processed	: 6/16/2022 11:54:45 AM

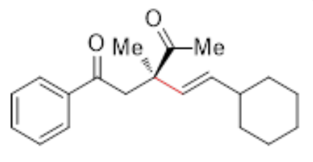

3v

<Chromatogram>

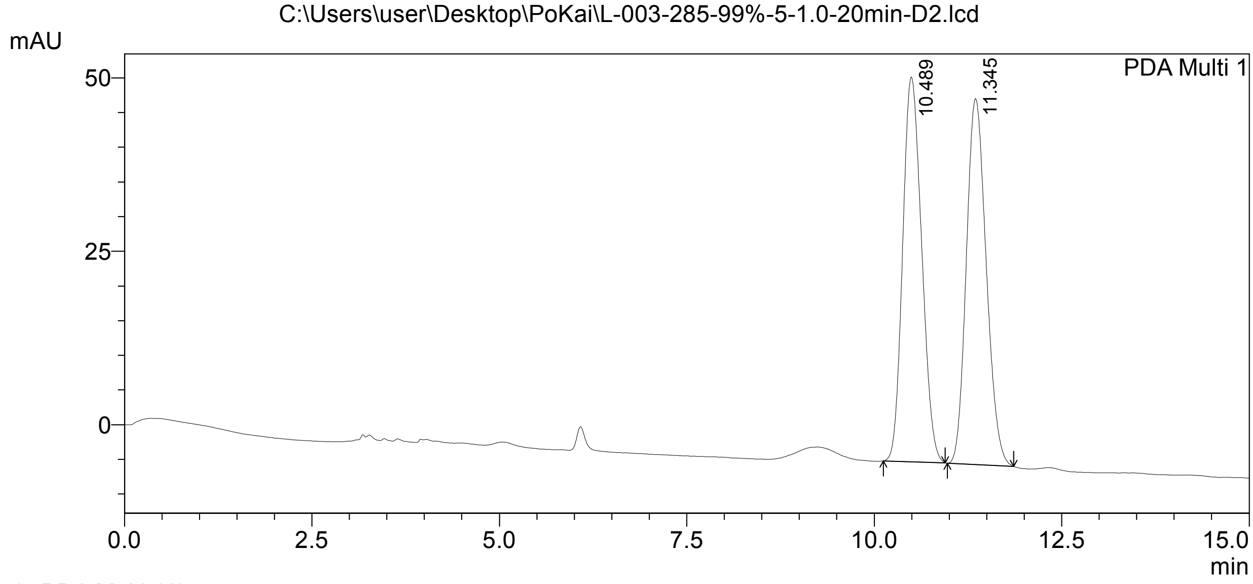

1 PDA Multi 1/254nm 4nm

PeakTable

PDA Ch1 254nm 4nm

Peak#	Ret. Time	Area	Height	Area %	Height %
1	10.489	974394	55469	49.859	51.240
2	11.345	979925	52784	50.141	48.760
Total		1954319	108252	100.000	100.000

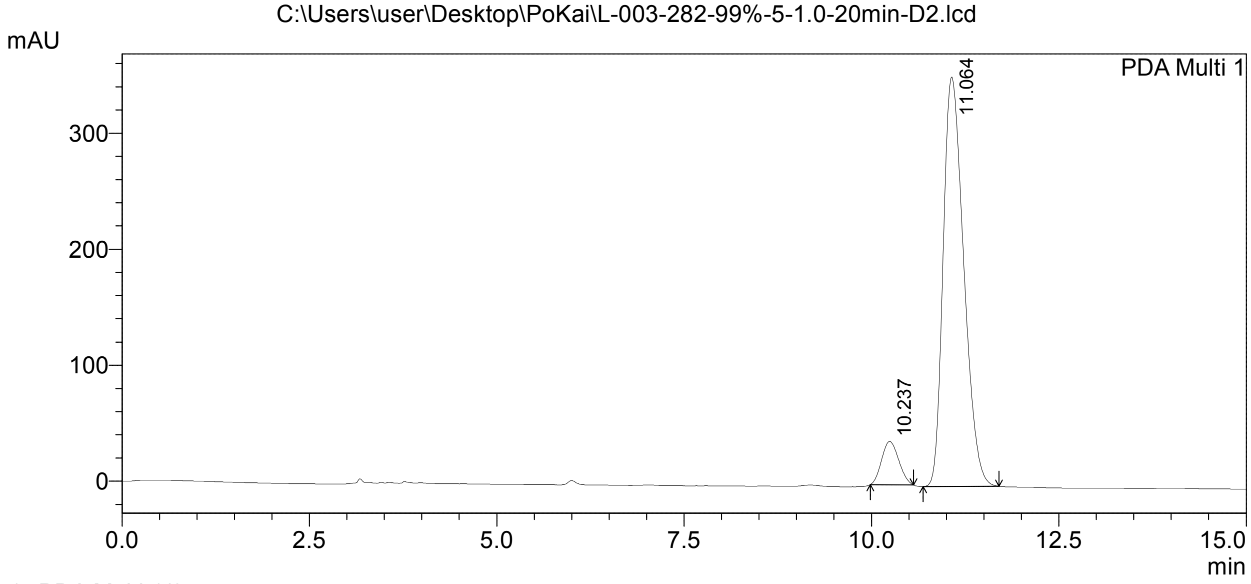

1 PDA Multi 1/254nm 4nm

PeakTable

PDA Ch1 254nm 4nm

Peak#	Ret. Time	Area	Height	Area %	Height %
1	10.237	593102	37407	8.078	9.583
2	11.064	6749490	352948	91.922	90.417
Total		7342591	390356	100.000	100.000

==== Shimadzu LCsolution Analysis Report ====

C:\Users\user\Desktop\PoKai\L-003-301-1-92%-0.7-6.lcd

Acquired by	: Admin
Sample Name	: L-003-301-1-6-92%-0.7
Sample ID	: L-003-301-1-6-92%-0.7
Tray#	: 1
Vail #	: 1
Injection Volume	: 10 uL
Data File Name	: L-003-301-1-92%-0.7-6.lcd
Method File Name	: pos6-92%_30min_0.7.lcm
Batch File Name	: Batch_table_C6-92_30min 0.7.lcb
Report File Name	: Default.lcr
Data Acquired	: 6/22/2022 3:40:55 PM
Data Processed	: 6/22/2022 4:07:04 PM

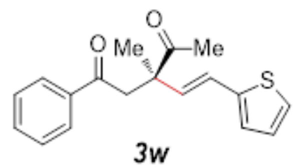

3w

<Chromatogram>

C:\Users\user\Desktop\PoKai\L-003-301-1-92%-0.7-6.lcd

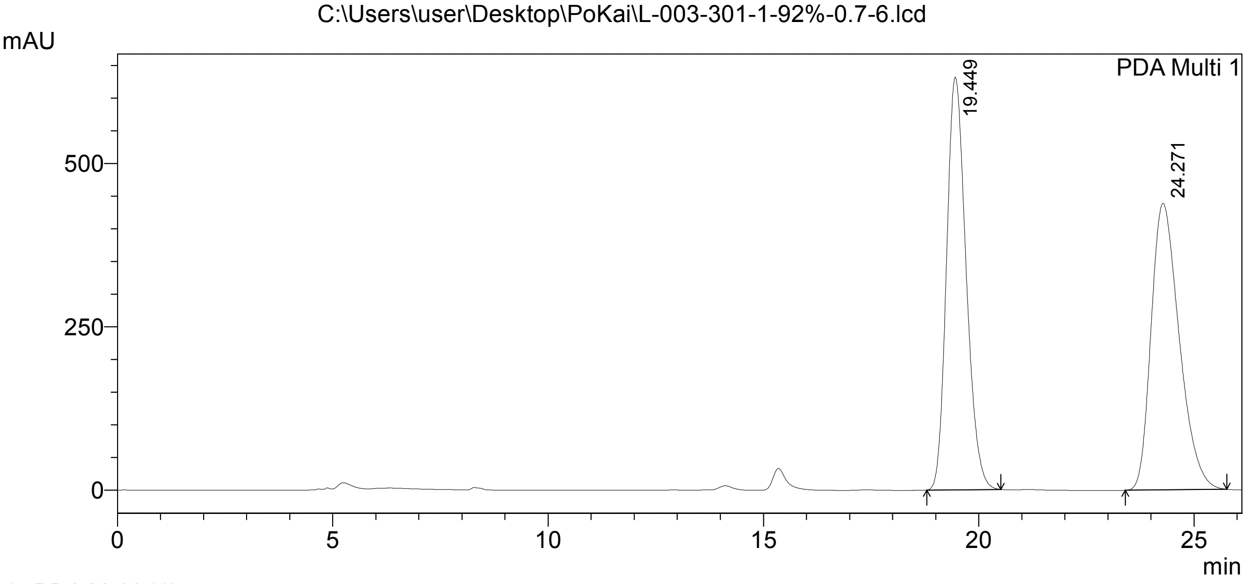

1 PDA Multi 1/254nm 4nm

PeakTable

PDA Ch1 254nm 4nm

Peak#	Ret. Time	Area	Height	Area %	Height %
1	19.449	19946017	631974	50.026	59.012
2	24.271	19925093	438959	49.974	40.988
Total		39871111	1070933	100.000	100.000

C:\Users\user\Desktop\PoKai\L-003-301-92%-0.7-6.lcd

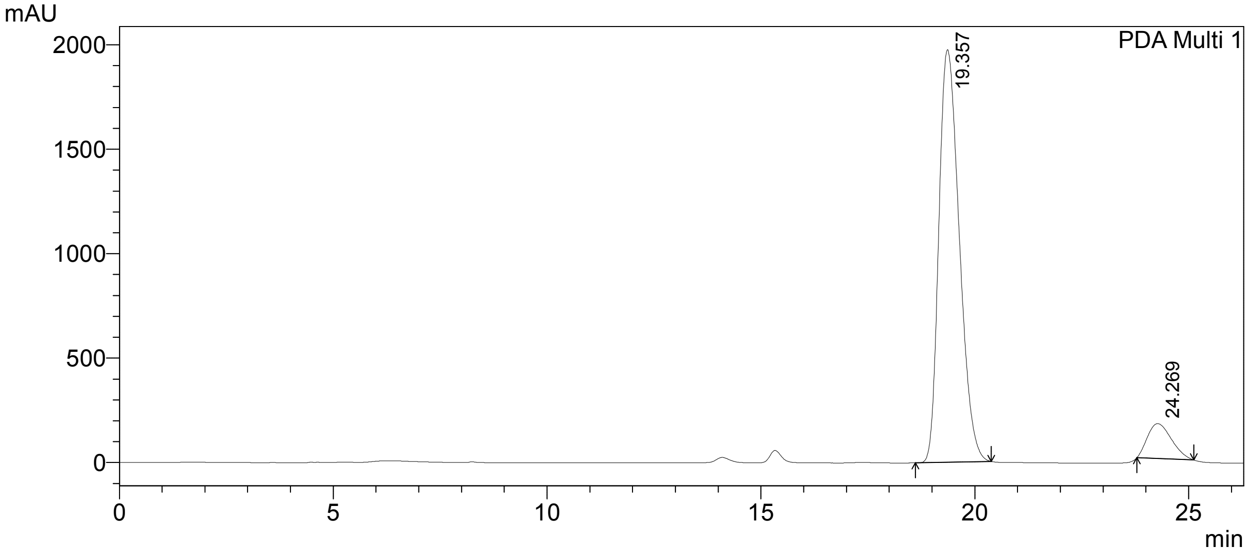

1 PDA Multi 1/254nm 4nm

PeakTable

PDA Ch1 254nm 4nm

Peak#	Ret. Time	Area	Height	Area %	Height %
1	19.357	65745782	1975414	90.929	92.199
2	24.269	6558762	167142	9.071	7.801
Total		72304544	2142555	100.000	100.000

==== Shimadzu LCsolution Analysis Report ====

C:\Users\user\Desktop\PoKai\L-003-280-99%-5-1.0-20min-D2.lcd

Acquired by	: Admin
Sample Name	: L-003-280-99%-5-1.0-20min-D2
Sample ID	: L-003-280-99%-5-1.0-20min-D2
Tray#	: 1
Vail #	: 1
Injection Volume	: 10 uL
Data File Name	: L-003-280-99%-5-1.0-20min-D2.lcd
Method File Name	: pos5_99%_20min_1.0_D2.lcm
Batch File Name	: Batch_table_C5_99%_10min_1.0.lcb
Report File Name	: Default.lcr
Data Acquired	: 6/14/2022 4:00:22 PM
Data Processed	: 6/14/2022 5:07:13 PM

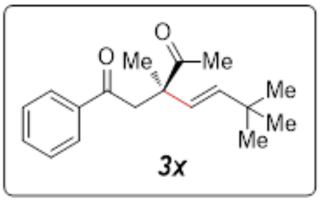

<Chromatogram>

C:\Users\user\Desktop\PoKai\L-003-280-99%-5-1.0-20min-D2.lcd

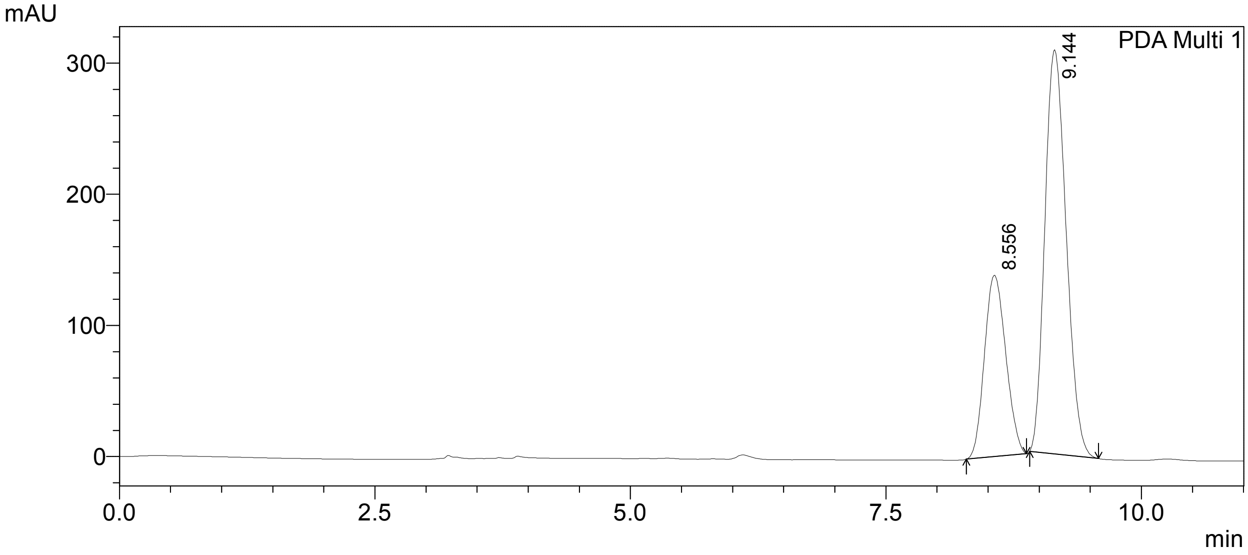

1 PDA Multi 1/254nm 4nm

PeakTable

PDA Ch1 254nm 4nm

Peak#	Ret. Time	Area	Height	Area %	Height %
1	8.556	2008502	138332	30.977	30.981
2	9.144	4475262	308175	69.023	69.019
Total		6483764	446507	100.000	100.000

C:\Users\user\Desktop\PoKai\L-003-283-99%-5-1.0-20min-D2.lcd

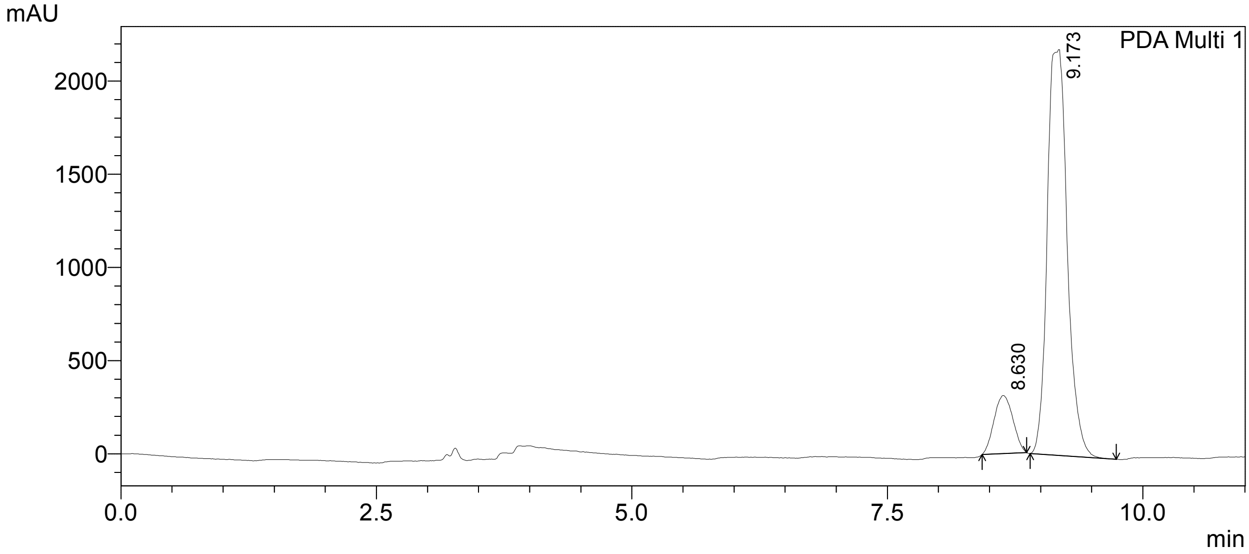

1 PDA Multi 1/190nm 4nm

PeakTable

PDA Ch1 190nm 4nm

Peak#	Ret. Time	Area	Height	Area %	Height %
1	8.630	4013268	311405	11.907	12.515
2	9.173	29692486	2176941	88.093	87.485
Total		33705754	2488346	100.000	100.000

==== Shimadzu LCsolution Analysis Report ====

C:\Users\user\Desktop\PoKai\L-003-128-95%-5-1.0-30min-D2.lcd

Acquired by	: Admin
Sample Name	: L-003-128-95%-5-1.0-30min-D2
Sample ID	: L-003-128-95%-5-1.0-30min-D2
Tray#	: 1
Vail #	: 1
Injection Volume	: 10 uL
Data File Name	: L-003-128-95%-5-1.0-30min-D2.lcd
Method File Name	: pos5_95%_10min_1.lcm
Batch File Name	: Batch_table_C5-95_10min_1.0.lcb
Report File Name	: Default.lcr
Data Acquired	: 11/3/2021 2:04:29 PM
Data Processed	: 11/3/2021 2:14:32 PM

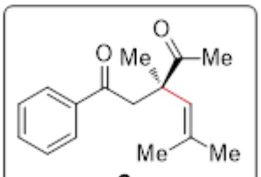

3y

<Chromatogram>

C:\Users\user\Desktop\PoKai\L-003-128-95%-5-1.0-30min-D2.lcd

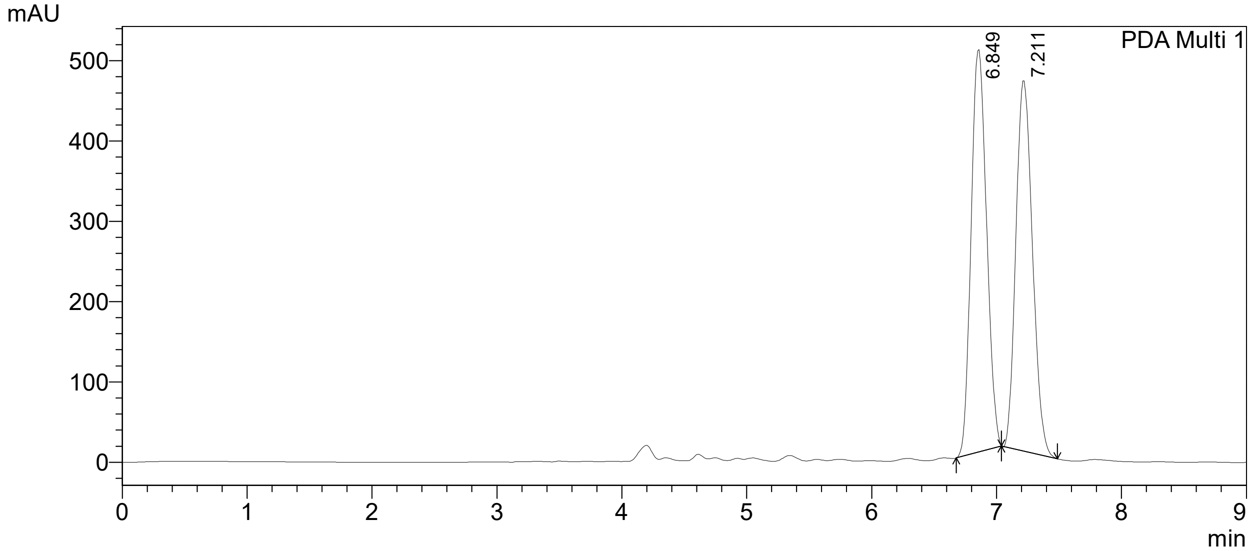

1 PDA Multi 1/254nm 4nm

PeakTable

PDA Ch1 254nm 4nm

Peak#	Ret. Time	Area	Height	Area %	Height %
1	6.849	4328764	501682	50.416	52.125
2	7.211	4257383	460776	49.584	47.875
Total		8586147	962457	100.000	100.000

C:\Users\user\Desktop\PoKai\L-002-292-95%-5-1.0-30min-D2.lcd

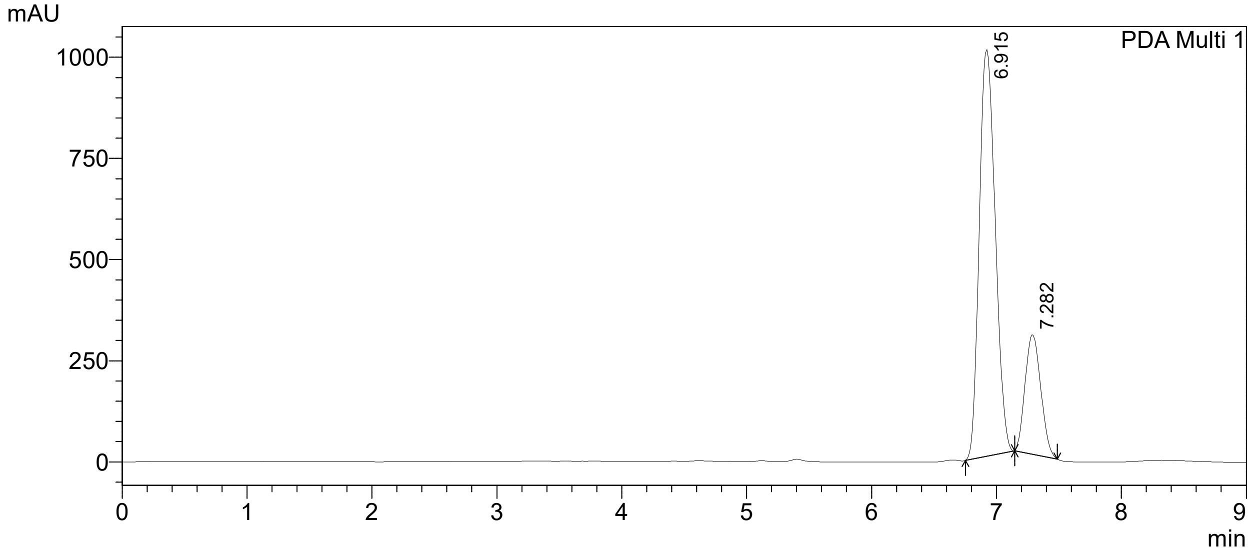

1 PDA Multi 1/254nm 4nm

PeakTable

PDA Ch1 254nm 4nm

Peak#	Ret. Time	Area	Height	Area %	Height %
1	6.915	8976546	1005364	77.633	77.295
2	7.282	2586225	295324	22.367	22.705
Total		11562770	1300687	100.000	100.000

==== Shimadzu LCsolution Analysis Report ====

C:\Users\user\Desktop\PoKai\L-003-156-92%-3-20min.lcd

Acquired by	: Admin
Sample Name	: L-003-156-92%-3-20min
Sample ID	: L-003-156-92%-3-20min
Tray#	: 1
Vail #	: 1
Injection Volume	: 1 uL
Data File Name	: L-003-156-92%-3-20min.lcd
Method File Name	: pos3-92%_20MIN_1.0_D2.lcm
Batch File Name	: Batch table C3 _92%_20min_1.0_D2.lcb
Report File Name	: Default.lcr
Data Acquired	: 11/24/2021 11:57:12 AM
Data Processed	: 11/24/2021 12:17:15 PM

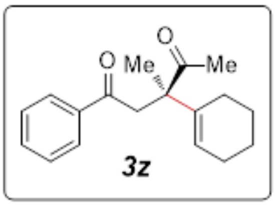

<Chromatogram>

C:\Users\user\Desktop\PoKai\L-003-156-92%-3-20min.lcd

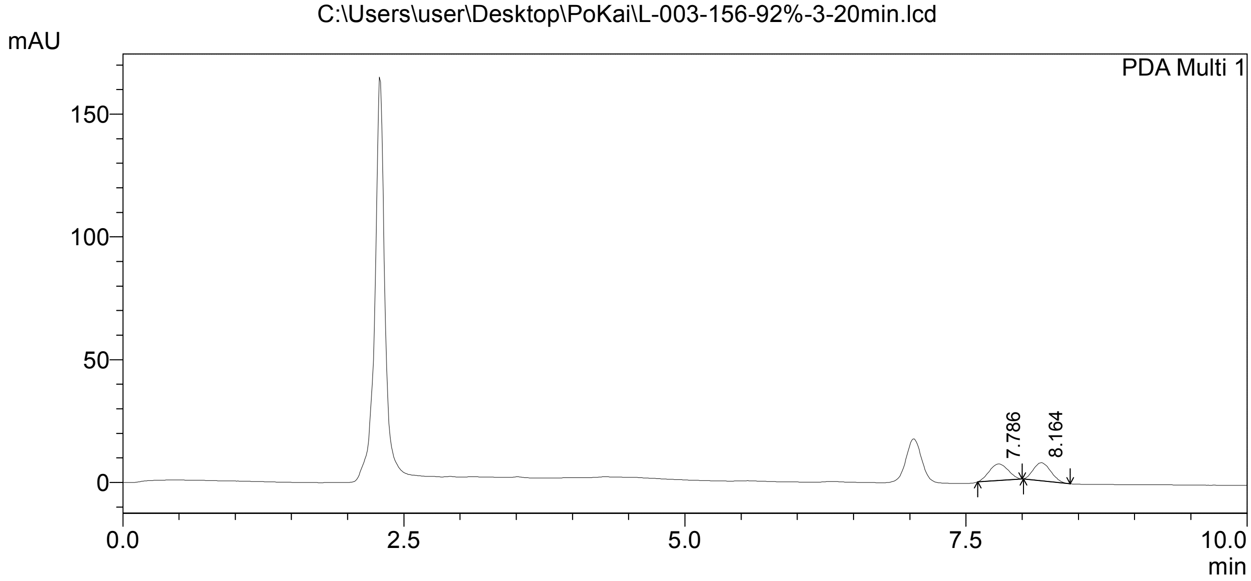

1 PDA Multi 1/254nm 4nm

PeakTable

PDA Ch1 254nm 4nm

Peak#	Ret. Time	Area	Height	Area %	Height %
1	7.786	79114	6746	50.213	47.969
2	8.164	78442	7317	49.787	52.031
Total		157556	14063	100.000	100.000

C:\Users\user\Desktop\PoKai\L-003-006-98%-10min-1.0-D2.lcd

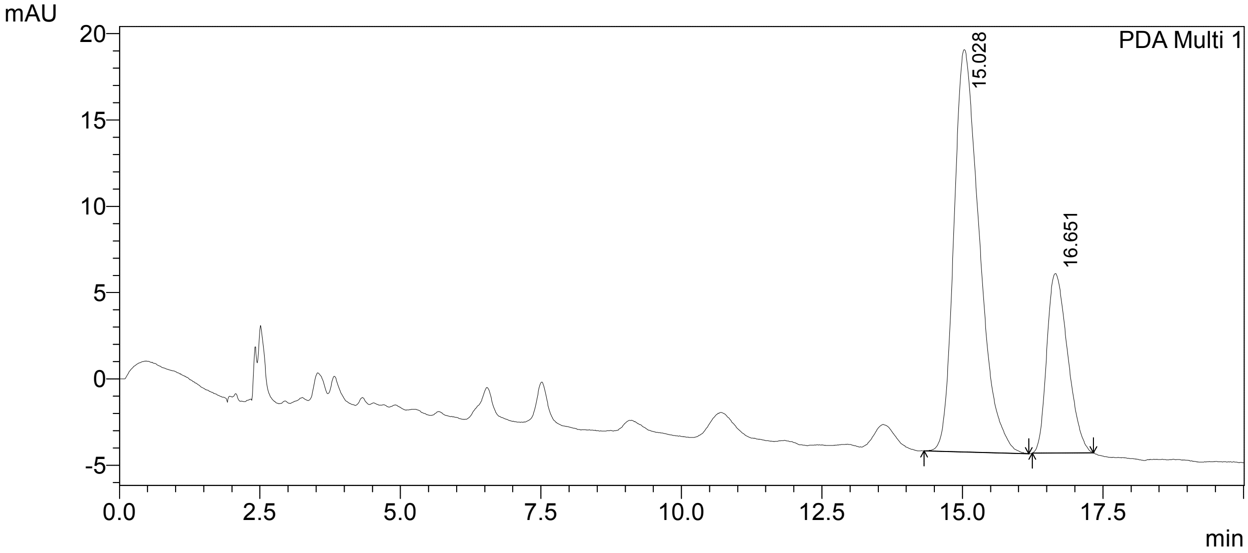

1 PDA Multi 1/254nm 4nm

PeakTable

PDA Ch1 254nm 4nm

Peak#	Ret. Time	Area	Height	Area %	Height %
1	15.028	736607	23303	73.228	69.164
2	16.651	269298	10389	26.772	30.836
Total		1005905	33692	100.000	100.000

==== Shimadzu LCsolution Analysis Report ====

C:\Users\user\Desktop\PoKai\L-003-221-90%-3-10min-1.0.lcd

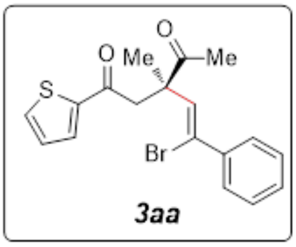

Acquired by	: Admin
Sample Name	: L-003-221-90%-3-10min
Sample ID	: L-003-221-90%-3-10min
Tray#	: 1
Vail #	: 1
Injection Volume	: 10 uL
Data File Name	: L-003-221-90%-3-10min-1.0.lcd
Method File Name	: pos3-90%_10MIN_1_d2.lcm
Batch File Name	: Batch table C3 _90%_10min_1.0_D2.lcb
Report File Name	: Default.lcr
Data Acquired	: 4/11/2022 5:18:09 PM
Data Processed	: 4/11/2022 5:27:33 PM

<Chromatogram>

C:\Users\user\Desktop\PoKai\L-003-221-90%-3-10min-1.0.lcd

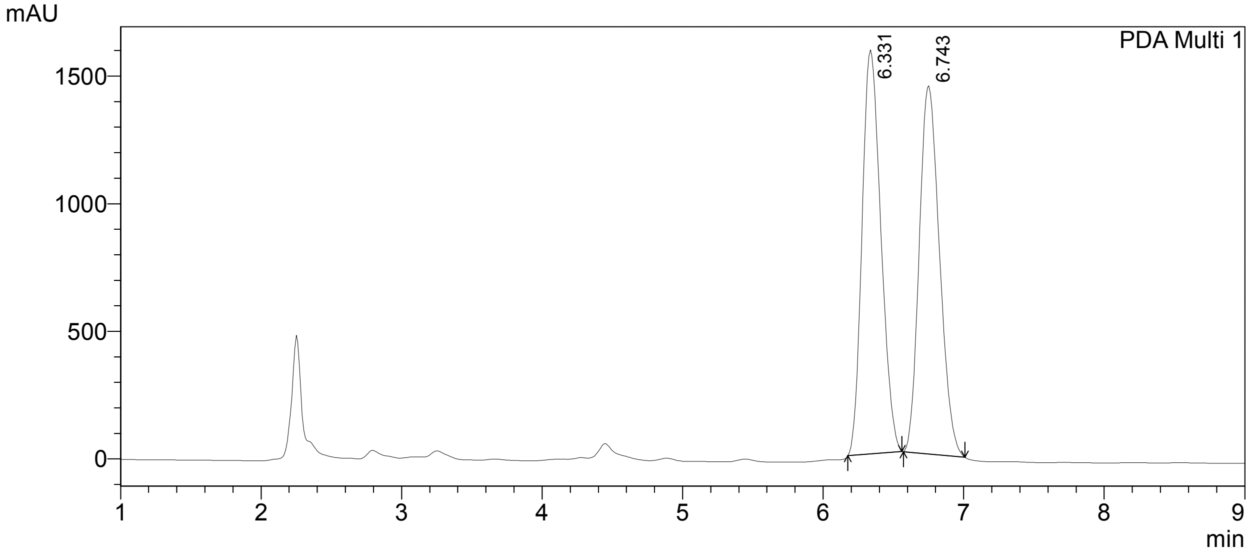

1 PDA Multi 1/254nm 4nm

PeakTable

PDA Ch1 254nm 4nm

Peak#	Ret. Time	Area	Height	Area %	Height %
1	6.331	14751705	1583020	50.479	52.331
2	6.743	14471642	1441987	49.521	47.669
Total		29223347	3025007	100.000	100.000

C:\Users\user\Desktop\PoKai\L-003-210-90%-3-10min-1.0.lcd

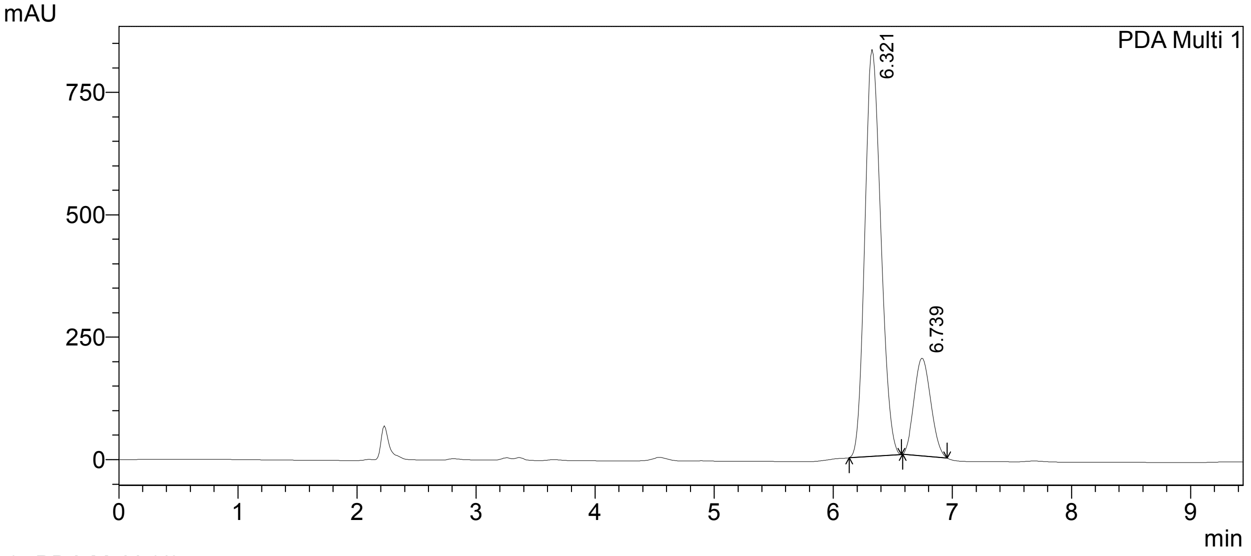

1 PDA Multi 1/254nm 4nm

PeakTable

PDA Ch1 254nm 4nm

Peak#	Ret. Time	Area	Height	Area %	Height %
1	6.321	7848262	830688	80.166	80.631
2	6.739	1941753	199550	19.834	19.369
Total		9790014	1030238	100.000	100.000

==== Shimadzu LCsolution Analysis Report ====

C:\Users\user\Desktop\PoKai\L-002-118-92%-3-20min.lcd

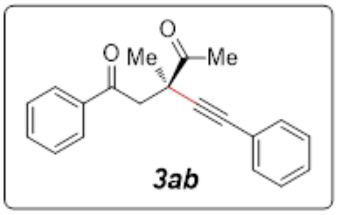

Acquired by	: Admin
Sample Name	: L-002-118-92%-3-20min
Sample ID	: L-002-118-92%-3-20min
Tray#	: 1
Vail #	: 1
Injection Volume	: 1 uL
Data File Name	: L-002-118-92%-3-20min.lcd
Method File Name	: pos3-92%_10MIN_1_d2.lcm
Batch File Name	: Batch table C3 _92%_20min_1.0_D2.lcb
Report File Name	: Default.lcr
Data Acquired	: 4/12/2022 1:05:04 PM
Data Processed	: 4/12/2022 1:12:31 PM

<Chromatogram>

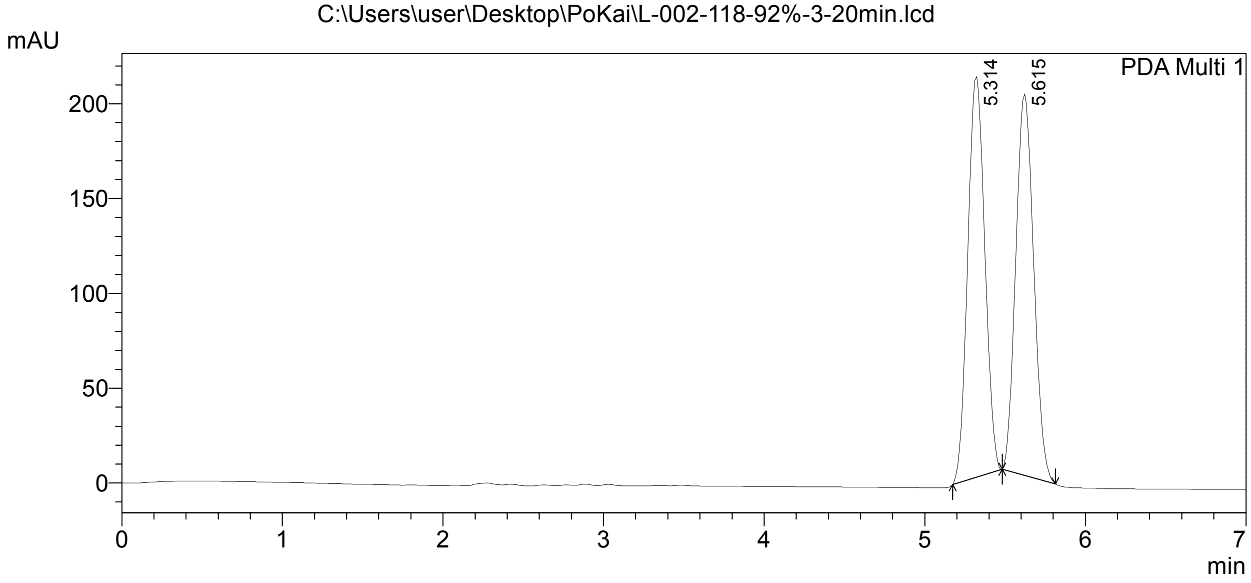

1 PDA Multi 1/254nm 4nm

PeakTable

PDA Ch1 254nm 4nm

Peak#	Ret. Time	Area	Height	Area %	Height %
1	5.314	1566899	211388	50.121	51.269
2	5.615	1559340	200922	49.879	48.731
Total		3126239	412310	100.000	100.000

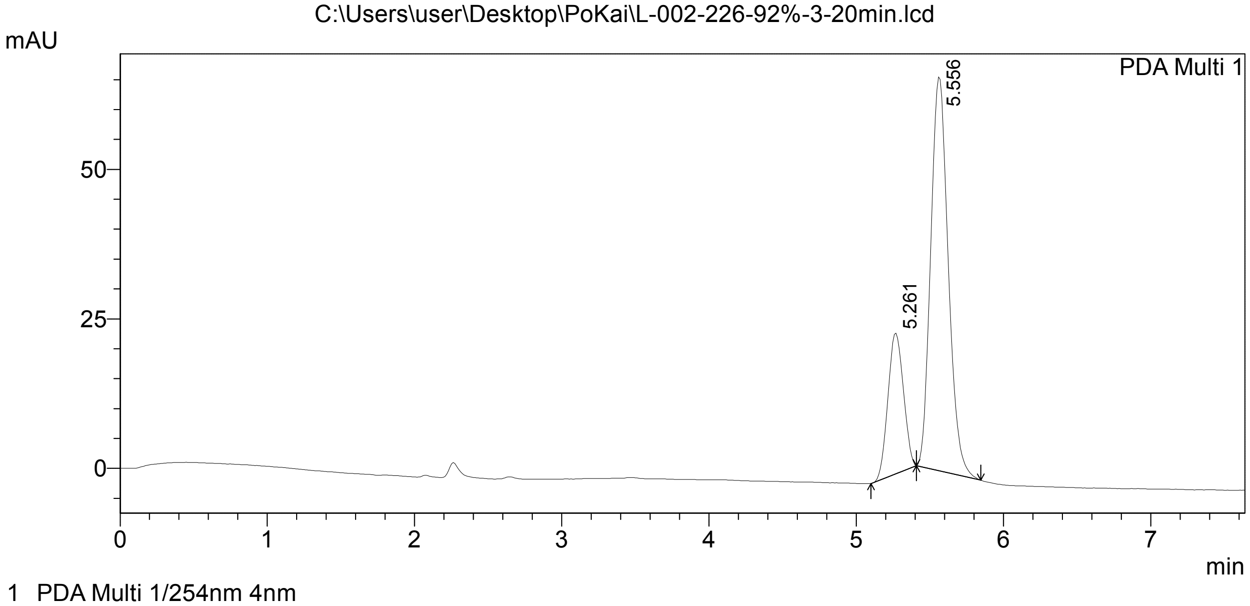

1 PDA Multi 1/254nm 4nm

PeakTable

PDA Ch1 254nm 4nm

Peak#	Ret. Time	Area	Height	Area %	Height %
1	5.261	173064	23590	24.124	26.381
2	5.556	544341	65830	75.876	73.619
Total		717405	89421	100.000	100.000

==== Shimadzu LCsolution Analysis Report ====

C:\Users\user\Desktop\PoKai\L-002-165-90%-3-10min-1.0.lcd

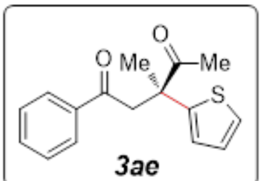

3ae

Acquired by	: Admin
Sample Name	: L-002-165-90%-3-10min
Sample ID	: L-002-165-90%-3-10min
Tray#	: 1
Vail #	: 1
Injection Volume	: 10 uL
Data File Name	: L-002-165-90%-3-10min-1.0.lcd
Method File Name	: pos3-90%_10MIN_1_d2.lcm
Batch File Name	: Batch table C3 _90%_10min_1.0_D2.lcb
Report File Name	: Default.lcr
Data Acquired	: 5/4/2021 1:35:42 PM
Data Processed	: 5/4/2021 1:45:43 PM

<Chromatogram>

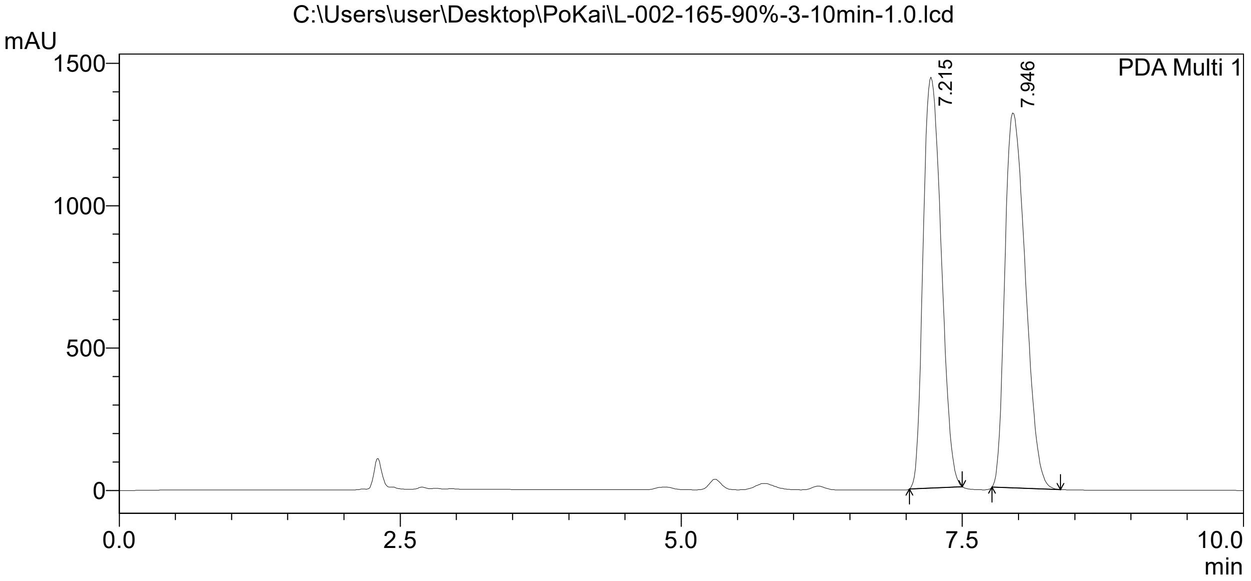

1 PDA Multi 1/254nm 4nm

PeakTable

PDA Ch1 254nm 4nm

Peak#	Ret. Time	Area	Height	Area %	Height %
1	7.215	16150966	1442403	49.071	52.294
2	7.946	16762694	1315852	50.929	47.706
Total		32913660	2758256	100.000	100.000

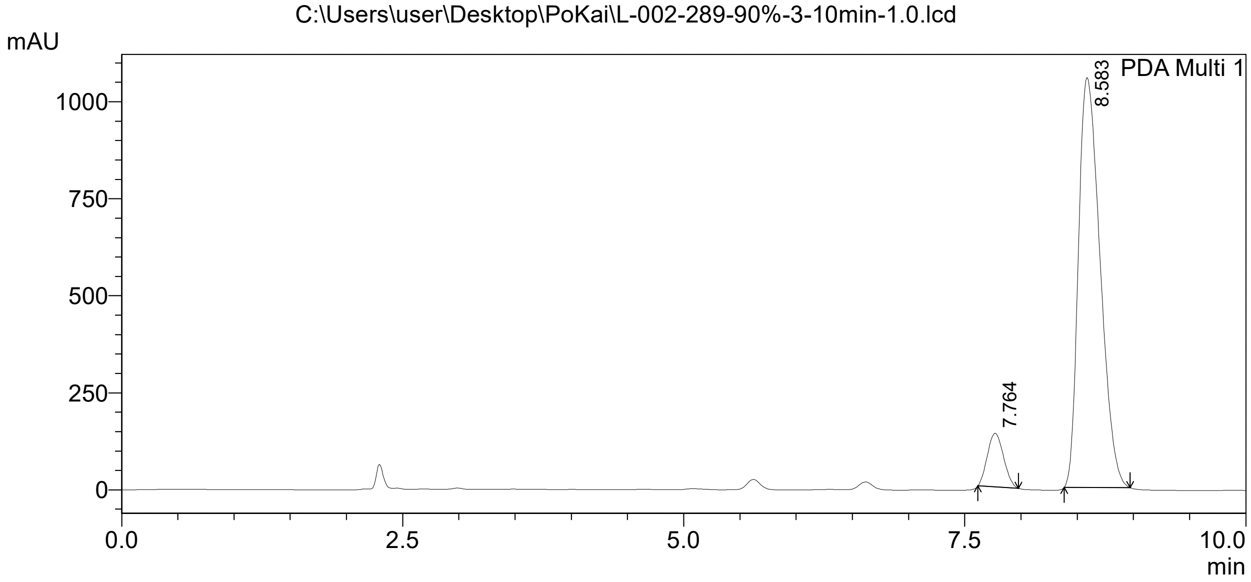

1 PDA Multi 1/254nm 4nm

PeakTable

PDA Ch1 254nm 4nm

Peak#	Ret. Time	Area	Height	Area %	Height %
1	7.764	1382916	138185	8.873	11.567
2	8.583	14202739	1056458	91.127	88.433
Total		15585655	1194643	100.000	100.000

==== Shimadzu LCsolution Analysis Report ====

C:\Users\user\Desktop\PoKai\L-003-073-90%-3-20min-1.0.lcd

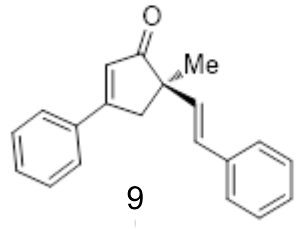

9

Acquired by	: Admin
Sample Name	: L-003-073-90%-3-20min
Sample ID	: L-003-073-90%-3-20min
Tray#	: 1
Vail #	: 1
Injection Volume	: 10 uL
Data File Name	: L-003-073-90%-3-20min-1.0.lcd
Method File Name	: pos3-90%_20MIN_1_d2.lcm
Batch File Name	: Batch table C3 _90%_20min_1.0_D2.lcb
Report File Name	: Default.lcr
Data Acquired	: 10/1/2021 1:19:04 PM
Data Processed	: 10/1/2021 1:39:06 PM

<Chromatogram>

C:\Users\user\Desktop\PoKai\L-003-073-90%-3-20min-1.0.lcd

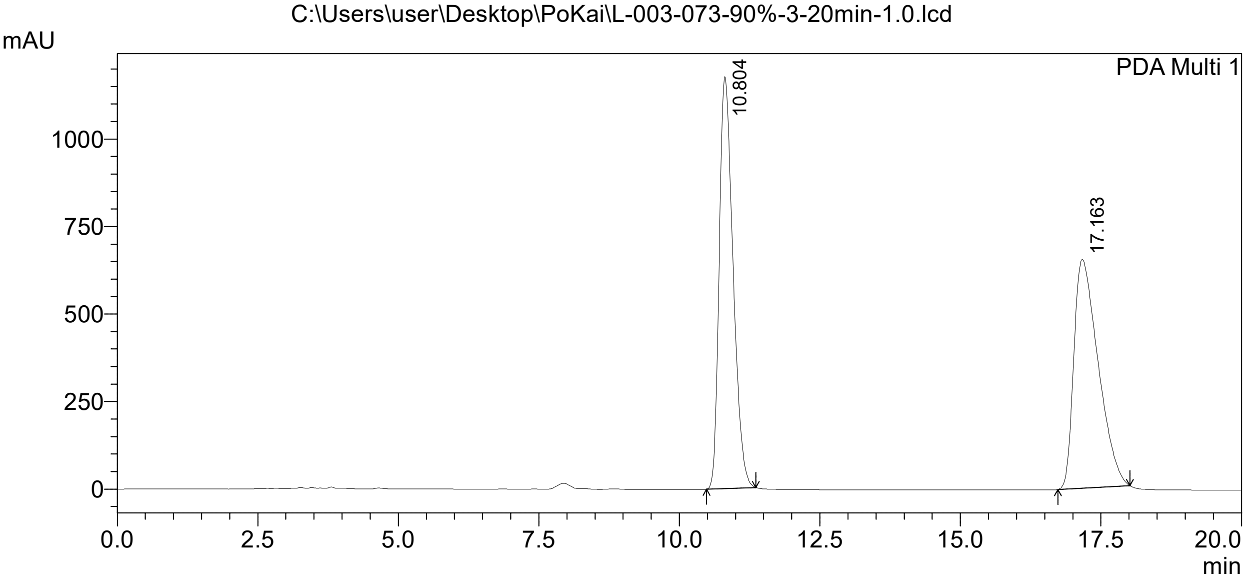

1 PDA Multi 1/254nm 4nm

PeakTable

PDA Ch1 254nm 4nm

Peak#	Ret. Time	Area	Height	Area %	Height %
1	10.804	20159834	1176546	50.562	64.296
2	17.163	19711856	653339	49.438	35.704
Total		39871689	1829885	100.000	100.000

C:\Users\user\Desktop\PoKai\L-003-060-90%-3-20min-1.0.lcd

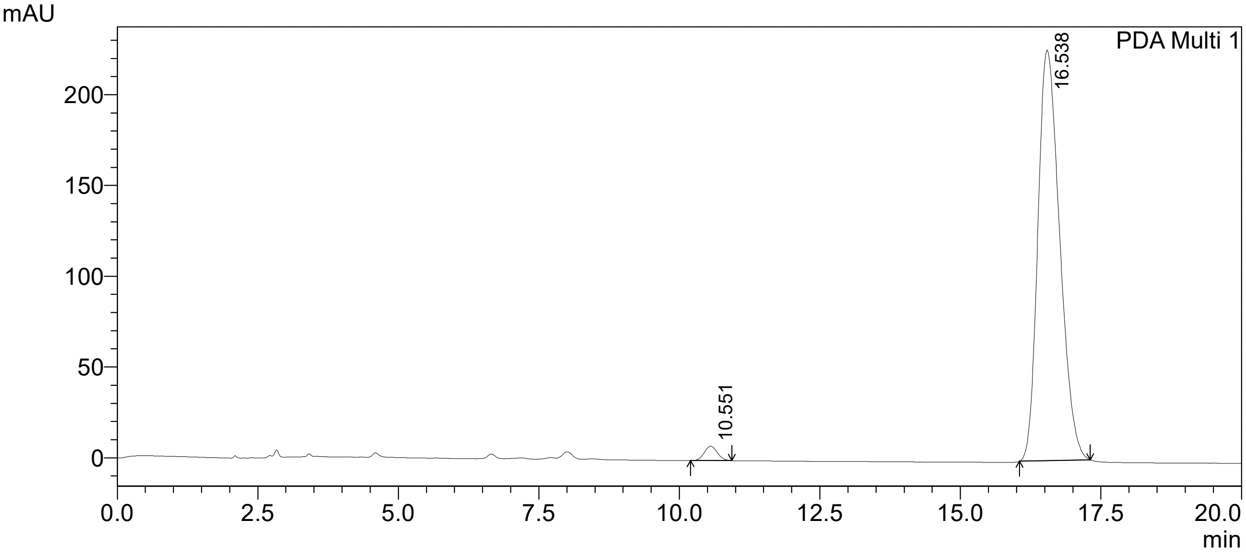

1 PDA Multi 1/254nm 4nm

PeakTable

PDA Ch1 254nm 4nm

Peak#	Ret. Time	Area	Height	Area %	Height %
1	10.551	128603	7918	2.062	3.382
2	16.538	6106854	226212	97.938	96.618
Total		6235456	234130	100.000	100.000

==== Shimadzu LCsolution Analysis Report ====

C:\Users\user\Desktop\PoKai\L-003-190-90%-5-1.0-30min-D2.lcd

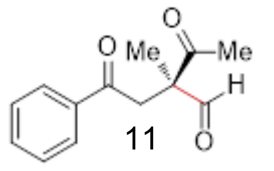

11

Acquired by	: Admin
Sample Name	: L-003-190-90%-5-1.0-30min-D2
Sample ID	: L-003-190-90%-5-1.0-30min-D2
Tray#	: 1
Vail #	: 1
Injection Volume	: 10 uL
Data File Name	: L-003-190-90%-5-1.0-30min-D2.lcd
Method File Name	: pos5_90%_30min_1.0.lcm
Batch File Name	: Batch_table_C5-90_30min.lcb
Report File Name	: Default.lcr
Data Acquired	: 2/9/2022 4:01:42 PM
Data Processed	: 2/9/2022 4:18:28 PM

<Chromatogram>

C:\Users\user\Desktop\PoKai\L-003-190-90%-5-1.0-30min-D2.lcd

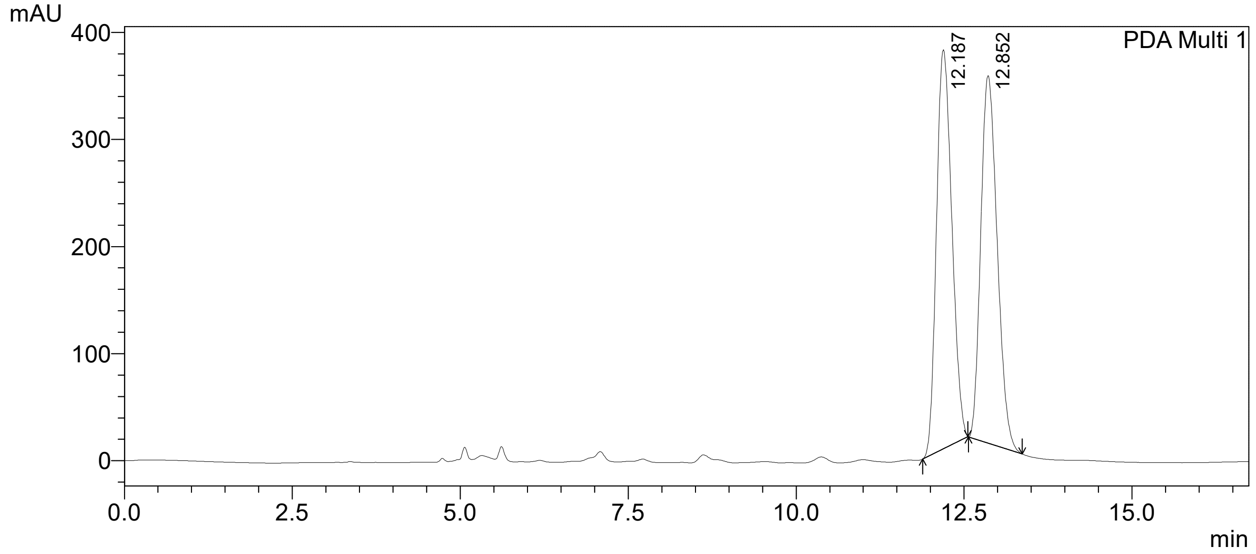

1 PDA Multi 1/254nm 4nm

PeakTable

PDA Ch1 254nm 4nm

Peak#	Ret. Time	Area	Height	Area %	Height %
1	12.187	5935846	372687	50.395	52.064
2	12.852	5842821	343142	49.605	47.936
Total		11778667	715829	100.000	100.000

C:\Users\user\Desktop\PoKai\L-003-194-90%-5-1.0-30min-D2.lcd

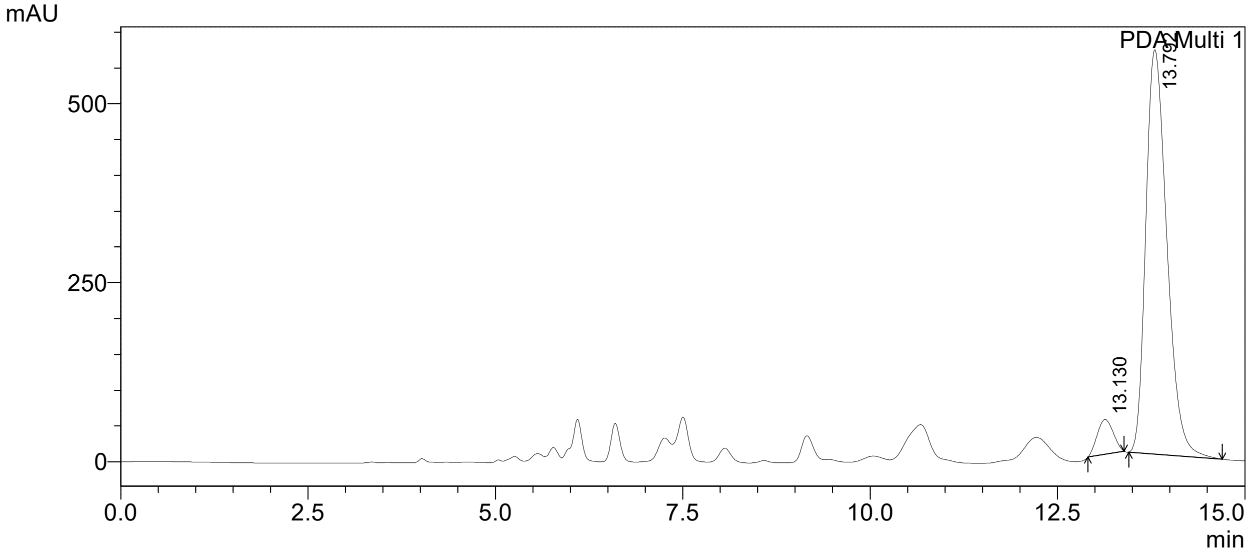

1 PDA Multi 1/254nm 4nm

PeakTable

PDA Ch1 254nm 4nm

Peak#	Ret. Time	Area	Height	Area %	Height %
1	13.130	723692	48620	6.142	7.931
2	13.792	11059606	564411	93.858	92.069
Total		11783297	613031	100.000	100.000

==== Shimadzu LCsolution Analysis Report ====

C:\Users\user\Desktop\Jessica\Data\L-003-127-99%-2-0.5.lcd

Acquired by	: Admin
Sample Name	: L-003-127-99%-2-0.5
Sample ID	: L-003-127-99%-2-0.5
Tray#	: 1
Vail #	: 1
Injection Volume	: 5 uL
Data File Name	: L-003-127-99%-2-0.5.lcd
Method File Name	: pos2-99%_20min_0.5_D2.lcm
Batch File Name	: Batch table C2_99%_20min_0.5_D2.lcb
Report File Name	: Default.lcr
Data Acquired	: 11/2/2021 3:04:45 PM
Data Processed	: 11/2/2021 3:24:47 PM

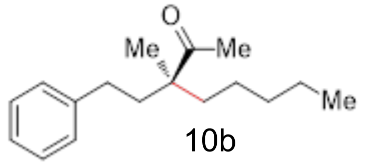

10b

C:\Users\user\Desktop\PoKai\L-003-127-99%-2-0.5.lcd

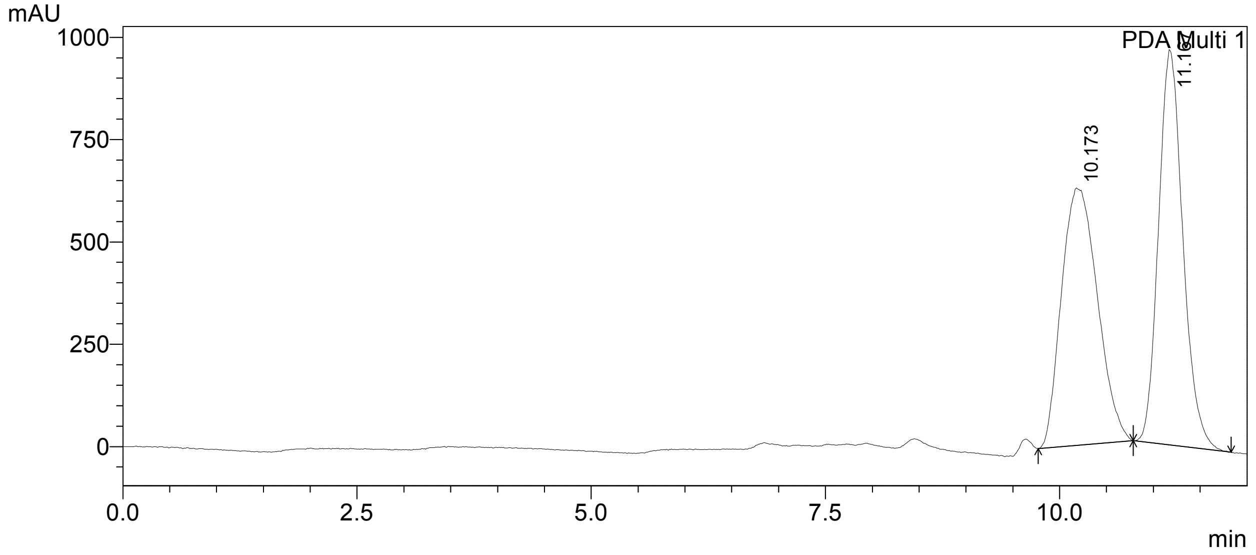

1 PDA Multi 1/190nm 4nm

PeakTable

PDA Ch1 190nm 4nm

Peak#	Ret. Time	Area	Height	Area %	Height %
1	10.173	16834839	628800	49.067	39.432
2	11.167	17475241	965844	50.933	60.568
Total		34310080	1594644	100.000	100.000

C:\Users\user\Desktop\PoKai\L-003-129-99%-2-0.5.lcd

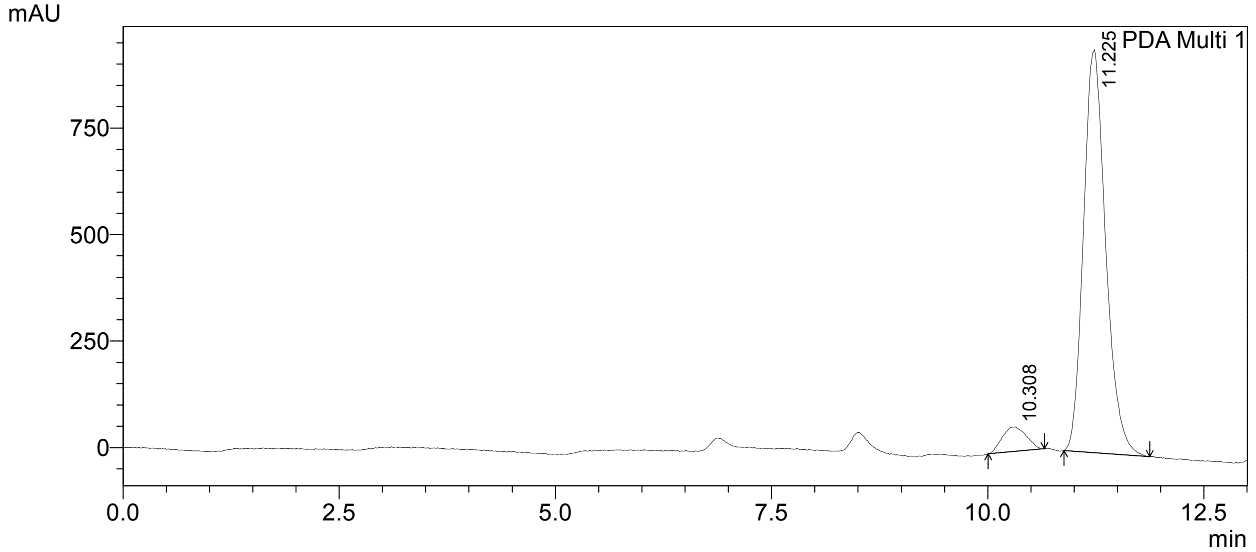

1 PDA Multi 1/190nm 4nm

PeakTable

PDA Ch1 190nm 4nm

Peak#	Ret. Time	Area	Height	Area %	Height %
1	10.308	1124614	57804	6.245	5.761
2	11.225	16884475	945613	93.755	94.239
Total		18009089	1003417	100.000	100.000

==== Shimadzu LCsolution Analysis Report ====

C:\Users\user\Desktop\PoKai\L-003-217-90%-5-1.0-30min-D2.lcd

Acquired by	: Admin
Sample Name	: L-003-217-90%-5-1.0-30min-D2
Sample ID	: L-003-217-90%-5-1.0-30min-D2
Tray#	: 1
Vail #	: 1
Injection Volume	: 10 uL
Data File Name	: L-003-217-90%-5-1.0-30min-D2.lcd
Method File Name	: pos5_90%_30min_1.0.lcm
Batch File Name	: Batch_table_C5-90_30min.lcb
Report File Name	: Default.lcr
Data Acquired	: 2/26/2022 1:33:09 PM
Data Processed	: 2/26/2022 1:54:07 PM

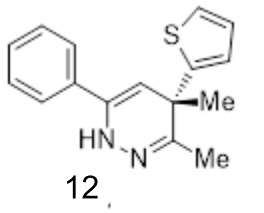

12

<Chromatogram>

C:\Users\user\Desktop\PoKai\L-003-217-90%-5-1.0-30min-D2.lcd

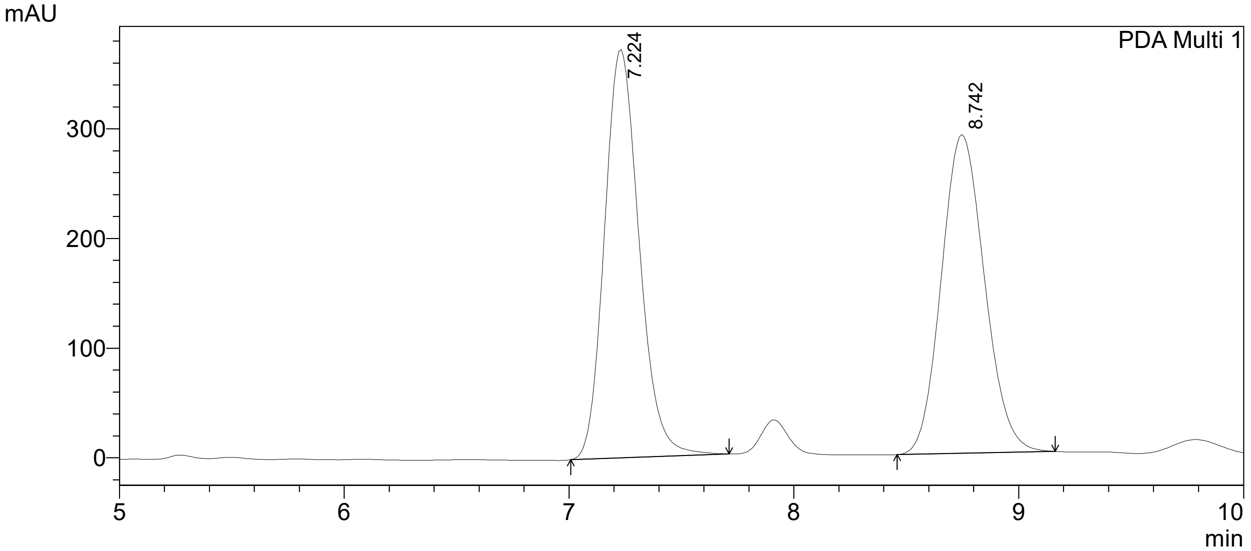

1 PDA Multi 1/254nm 4nm

PeakTable

PDA Ch1 254nm 4nm

Peak#	Ret. Time	Area	Height	Area %	Height %
1	7.224	3999210	372591	50.772	56.173
2	8.742	3877595	290702	49.228	43.827
Total		7876805	663293	100.000	100.000

C:\Users\user\Desktop\PoKai\L-003-218-90%-5-1.0-30min-D2.lcd

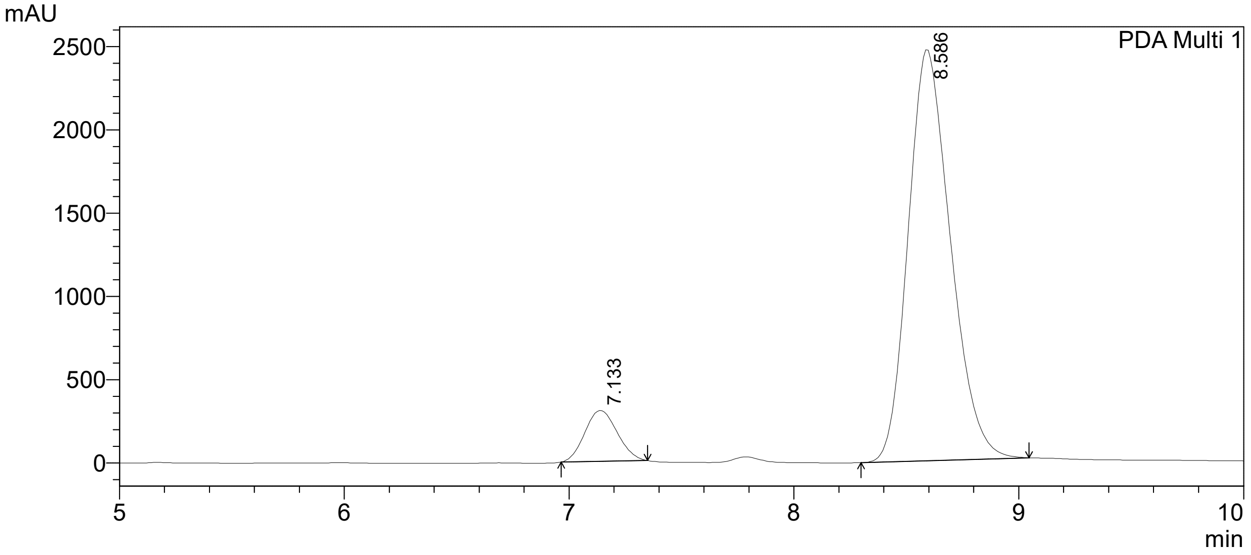

1 PDA Multi 1/254nm 4nm

PeakTable

PDA Ch1 254nm 4nm

Peak#	Ret. Time	Area	Height	Area %	Height %
1	7.133	3074044	306606	8.665	11.055
2	8.586	32401297	2466765	91.335	88.945
Total		35475341	2773371	100.000	100.000

==== Shimadzu LCsolution Analysis Report ====

C:\Users\user\Desktop\PoKai\L-003-220-90%-3-30min-1.0.lcd

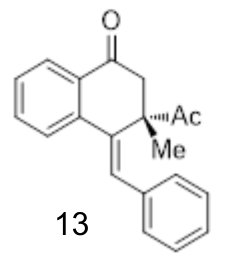

13

Acquired by	: Admin
Sample Name	: L-003-220-90%-3-30min
Sample ID	: L-003-220-90%-3-30min
Tray#	: 1
Vail #	: 1
Injection Volume	: 10 uL
Data File Name	: L-003-220-90%-3-30min-1.0.lcd
Method File Name	: pos3-90%_30MIN_1_d2.lcm
Batch File Name	: Batch table C3 _90%_30min_1.0_D2.lcb
Report File Name	: Default.lcr
Data Acquired	: 3/2/2022 9:27:43 AM
Data Processed	: 3/2/2022 9:55:42 AM

<Chromatogram>

C:\Users\user\Desktop\PoKai\L-003-220-90%-3-30min-1.0.lcd

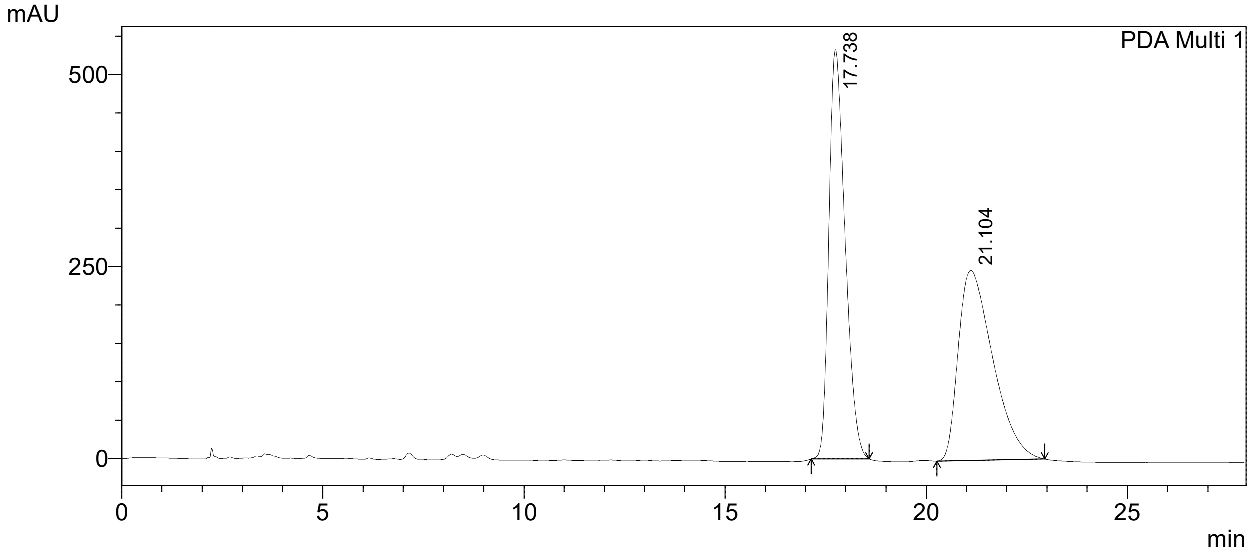

1 PDA Multi 1/254nm 4nm

PeakTable

PDA Ch1 254nm 4nm

Peak#	Ret. Time	Area	Height	Area %	Height %
1	17.738	14993739	532963	50.381	68.293
2	21.104	14767231	247440	49.619	31.707
Total		29760970	780404	100.000	100.000

C:\Users\user\Desktop\PoKai\L-003-185-90%-3-30min-1.0.lcd

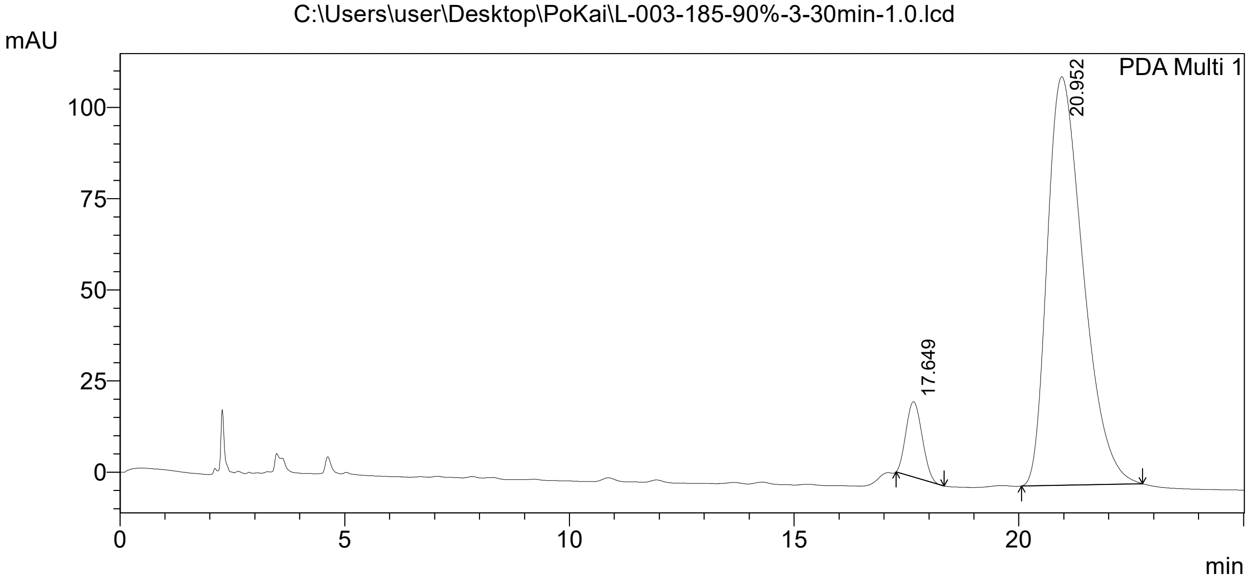

1 PDA Multi 1/254nm 4nm

PeakTable

PDA Ch1 254nm 4nm

Peak#	Ret. Time	Area	Height	Area %	Height %
1	17.649	514012	20610	7.633	15.547
2	20.952	6219754	111952	92.367	84.453
Total		6733765	132562	100.000	100.000

Computational Details:

Density Functional Theory (DFT) calculations were performed at the B3LYP/6-31G** level of theory[10] using Gaussian16 (Revision C.01).[11] Dispersion corrections were implemented using Grimme et al.'s D3 correction with the Becke-Jonhson damping scheme.[12] TS Structures feature 3,3´-difluorobisphenol ligands in lieu of the full 3,3´-diperfluorotoluyl-BINOL catalyst.

Electronic energies were adjusted with zero-point and thermal corrections to energy and free energy (298K and 1 atm) when comparing structures. Transition state geometries were determined by first solving for the Lewis acid/Lewis base intermediate structure, then employing relaxed potential energy surface scans to determine local energy maxima and local energy minima along the bond forming path. To ensure that the input geometries were close to a global minimum, conformer searches were performed using the VEGA ZZ software.[13]

Local energy maxima were subject to TS optimizations, while local energy minima were optimized to stable points. Transition state geometries were confirmed by the presence of one imaginary frequency and through convergence of intrinsic reaction coordinate (IRC) calculations.

Transition states with very low energy barriers were verified by reoptimizing structures to stable points that matched either the starting material or product geometry. Stable structures were confirmed by the absence of imaginary frequencies. An implicit solvent environment was incorporated through the CPCM method (solvent=toluene).[14] Natural Bond Orbitals (NBOs) and Canonical Molecular Orbitals (CMOs) were examined through the NBO7 program.[15] Optimized structures were visualized using the CYLview20 software,[16] and orbitals were rendered through the Chemcraft software[17]. HOMOs are represented by red and blue phases, while LUMOs are depicted with purple and gold orbitals.

Please refer to source data for xyz coordinates.

Supplementary Figure 4: Comparison of Computed Transition States of Potential Products

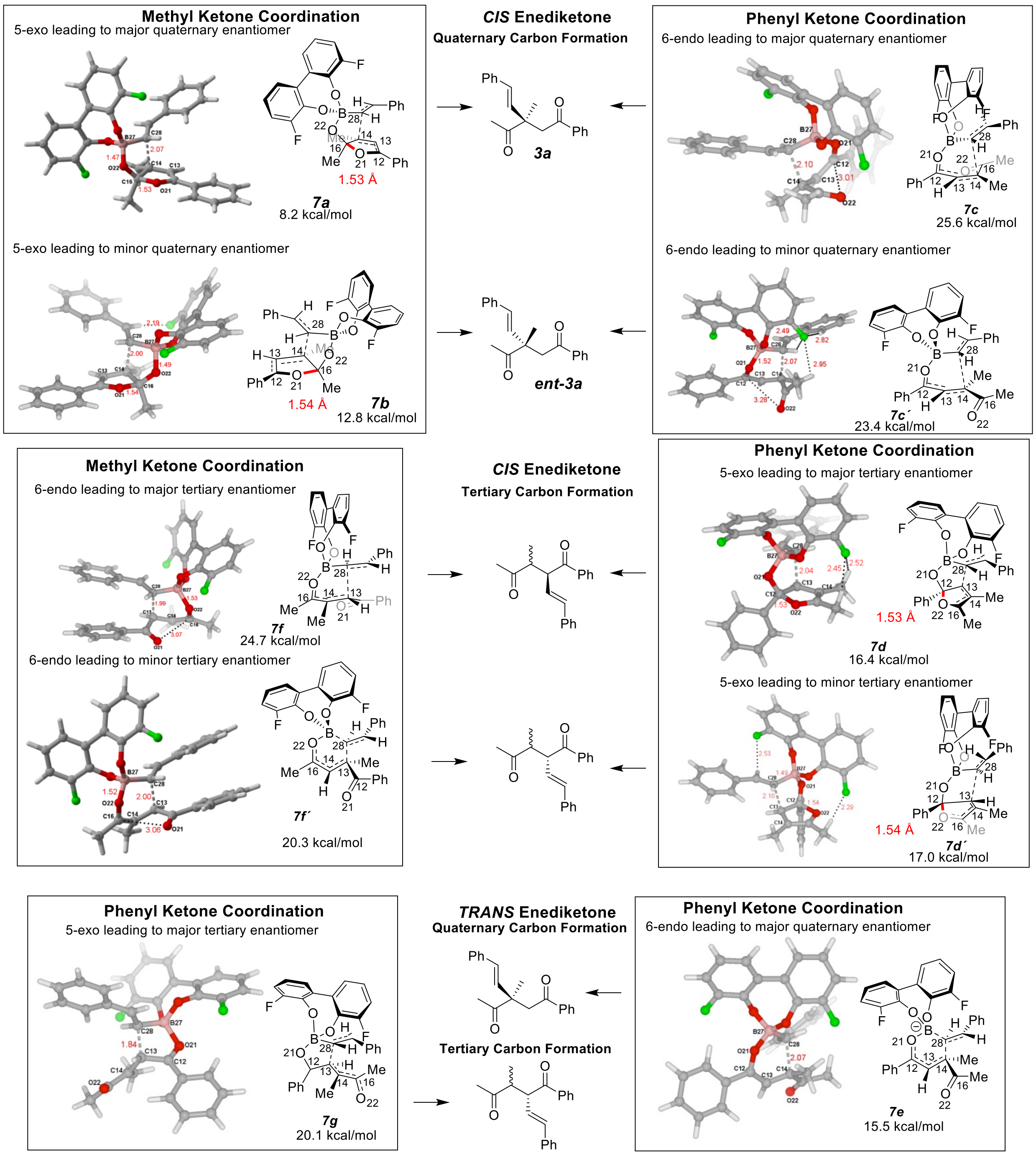

Supplementary Table 8. B3LYP/6-31G** Electronic Energies including Zero-point Energy (ZPE) Corrections, Thermal Correction to Enthalpy, Thermal Correction to Gibbs Free Energy, and imaginary frequencies for structures in the Conjugate Addition of Organoboranes to ene-diketones.

Structure	E_o	E_{ZPE}	G	H	Imag. Freq.
SM					
Trans ene-dione	-615.003895	-614.794344	-614.836445	-614.780835	None
PhCO Major	-614.998472	-614.776045	-614.830892	-614.776045	None
PhCO Minor	-614.998471	-614.789505	-614.830880	-614.776039	None
MeCO Major	-615.002050	-614.792936	-614.834518	-614.779367	None
MeCO Minor	-615.002051	-614.792935	-614.834518	-614.779366	None
Catalyst	-812.270832	-812.096755	-812.136244	-812.083822	None
Boronic Acid	-485.722582	-485.563839	-485.600957	-485.553662	None
Organoborane Diol	-1145.126639	-1144.843863	-1144.894064	-1144.824351	None
Cis ene-diketone: MeCO Pathway					
Major Down					
SM	-1760.157953	-1759.66471	-1759.733725	-1759.6300	None
LA/la	-1760.154741	-1759.66055	-1759.727756	-1759.6270	None
5 mem ts	-1760.145389	-1759.65015	-1759.715735	-1759.6185	-230.97
Prod	-1760.198674	-1759.7005	-1759.764712	-1759.6685	None
Major Down					
SM	-1760.157953	-1759.66471	-1759.733725	-1759.6300	None
LA/la	-1760.154741	-1759.66055	-1759.727756	-1759.6270	None
6 mem ts	-1760.123751	-1759.63027	-1759.694662	-1759.5977	-386.37
Prod	-1760.191248	-1759.69429	-1759.761446	-1759.6609	None
Major Up					
SM	-1760.156454	-1759.66348	-1759.733809	-1759.6285	None
LA/la	-1760.158295	-1759.66415	-1759.729692	-1759.6308	None
5 mem ts	-1760.137929	-1759.64318	-1759.705923	-1759.6115	-319.73
Prod	-1760.1751	-1759.67676	-1759.741595	-1759.6449	None
Major up					
SM	-	-1759.66348	-	-1759.6285	None

	1760.156454		1759.733809		
LA/la	-1760.158295	-1759.66415	-1759.729692	-1759.6308	None
5 mem ts	-1760.126247	-1759.63261	-1759.697723	-1759.5999	-321.75
Prod	-1760.193957	-1759.69745	-1759.762827	-1759.6643	None
Minor Down					
SM	-1760.157953	-1759.66578	-1759.734794	-1759.6310	None
LA/la	-1760.154741	-1759.66145	-1759.727312	-1759.6280	None
5 mem ts	-1760.145389	-1759.64055	-1759.703484	-1759.6088	-332.58
Prod	-1760.198674	-1759.67362	-1759.738693	-1759.6418	None
Minor Down					
SM	-1760.157953	-1759.66578	-1759.734794	-1759.6310	None
LA/la	-1760.154741	-1759.66145	-1759.727312	-1759.6280	None
6 mem ts	-1760.123751	-1759.63486	-1759.699698	-1759.6021	-145.84
Prod	-1760.191248	-1759.69291	-1759.758416	-1759.6597	None
Minor Up					
SM	-1760.156454	-1759.66511	-1759.734826	-1759.6303	None
LA/la	-1760.158295	-1759.66372	-1759.730642	-1759.6300	None
5 mem ts	-1760.137929	-1759.64765	-1759.712064	-1759.6161	-345.05
Prod	-1760.1751	-1759.70318	-1759.766333	-1759.6715	None
Minor up					
SM	-1760.156454	-1759.66511	-1759.734826	-1759.6303	None
LA/la	-1760.158295	-1759.66372	-1759.730642	-1759.6300	None
5 mem ts	-1760.126247	-1759.62488	-1759.690973	-1759.5921	-375.6
Prod	-1760.193957	-1759.69744	-1759.762835	-1759.6643	None
Cis ene-diketone: PhCO Pathway					
Major					

LA/la	-1760.157536	-1759.663726	-1759.730399	-1759.62997	None
5 mem ts	-1760.132878	-1759.638623	-1759.703777	-1759.606500	-169.11
Prod	-1760.197921	-1759.700759	-1759.766743	-1759.66806	None
Major Down					
LA/la	-1760.157536	-1759.663726	-1759.730399	-1759.62997	None
6 mem ts	-1760.12857	-1759.635159	-1759.700165	-1759.60261	-135.97
Prod	-1760.195099	-1759.699132	-1759.764743	-1759.66593	None
Minor Up					
LA/la	-1760.152668	-1759.659172	-1759.72737	-1759.62533	None
5 mem ts	-1760.133717	-1759.639354	-1759.704817	-1759.6072	-295.41
Prod	-1760.205376	-1759.70826	-1759.77294	-1759.67571	None
Minor up					
LA/la	-1760.152668	-1759.659172	-1759.72737	-1759.62533	None
6 mem ts	-1760.121581	-1759.628044	-1759.692903	-1759.59564	-234.63
Prod	-1760.195099	-1759.699132	-1759.764743	-1759.665929	None
Trans ene-diketone: PhCO Pathway					
Major					
LA/la	-1760.149524	-1759.655975	-1759.725071	-1759.621920	None
5 mem ts	-1760.119754	-1759.626085	-1759.693106	-1759.593167	-220.81
Prod	-1760.181409	-1759.685313	-1759.752065	-1759.652161	None
Major Down					
LA/la	-1760.157536	-1759.663726	-1759.730399	-1759.62997	None
6 mem ts	-1760.129685	-1759.635959	-1759.700589	-1759.603439	-242.07
Prod	-1760.195558	-1759.699782	-1759.766627	-1759.666375	None

Supplementary Figure 5: Conformation **S1** of PhCO donation to MeCO in cis-enediketone **1a** leading to major enantiomer. Minor enantiomer conformer is energetically identical.

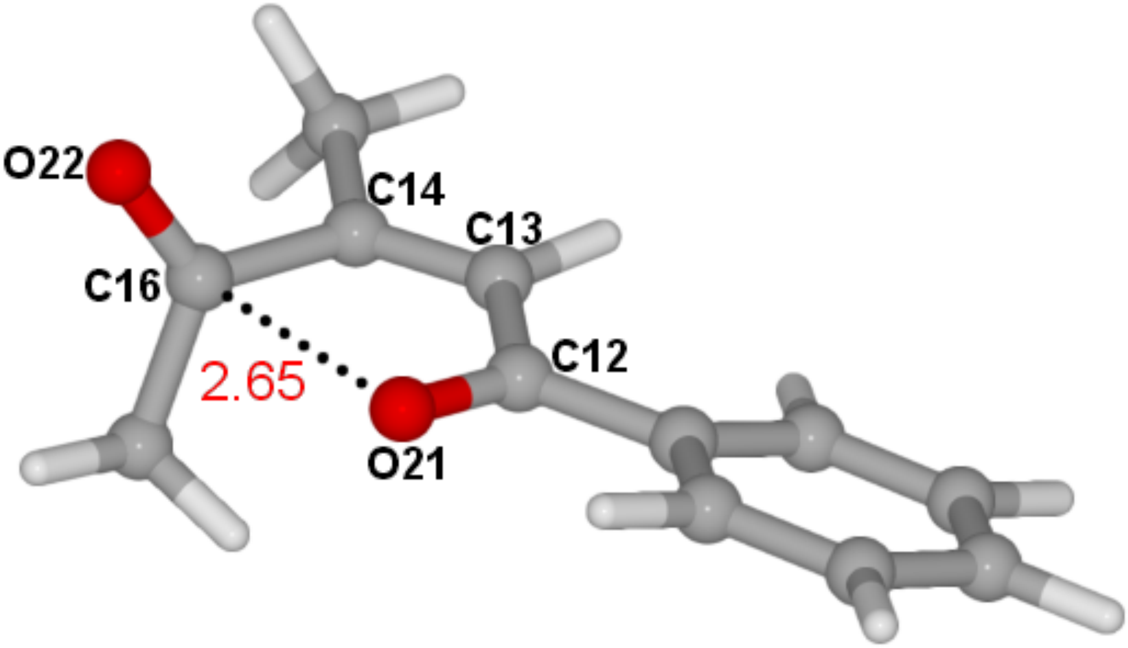

Electronic Energy	= -615.002050 Hartrees			
NBO Donor		NBO Acceptor		Stabilization Energy (kcal/mol)
15. LP (1) O 21		76. BD*(2) C 16- O 22		0.78
16. LP (2) O 21		76. BD*(2) C 16- O 22		4.36

Supplementary Figure 6: Conformation **S2** of MeCO donation to PhCO in cis-enediketone **1a** leading to major enantiomer. Minor enantiomer conformer is energetically identical.

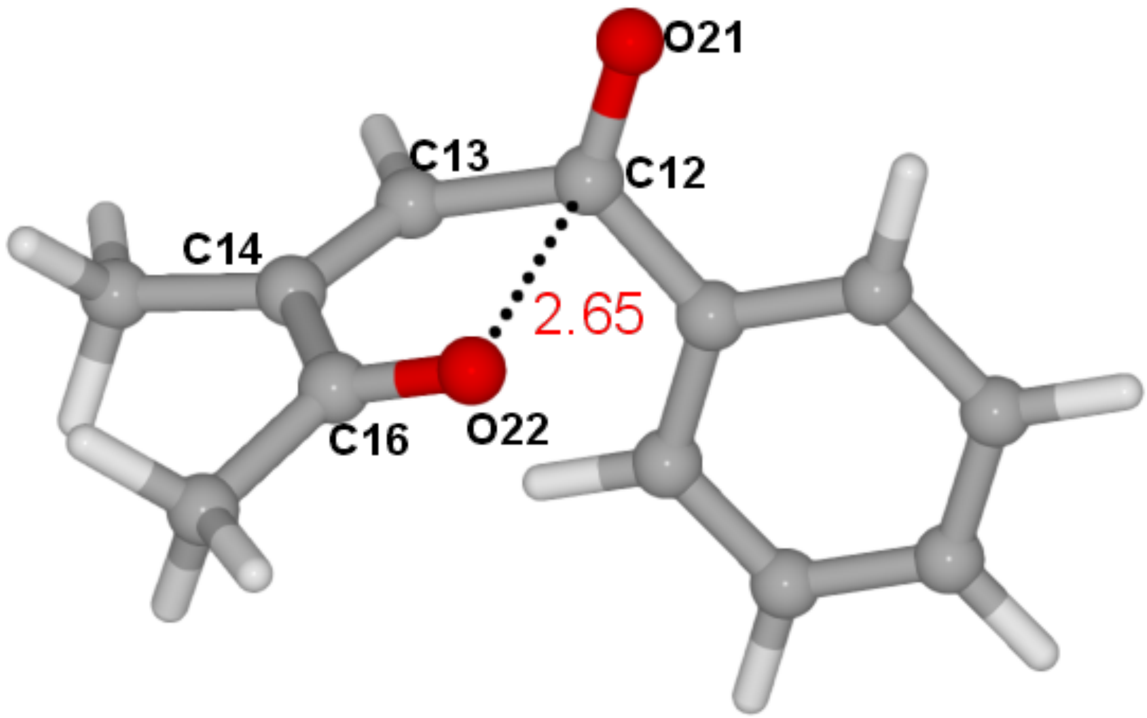

Electronic Energy = -614.998472 Hartrees

NBO Donor	NBO Acceptor	Stabilization Energy (kcal/mol)
17. LP (1) O 22	68. BD*(2) C 12- O 21	0.73
18. LP (2) O 22	68. BD*(2) C 12- O 21	4.26

Supplementary Figure 7: Conformation of trans-enediketone **1a** (***S3***)

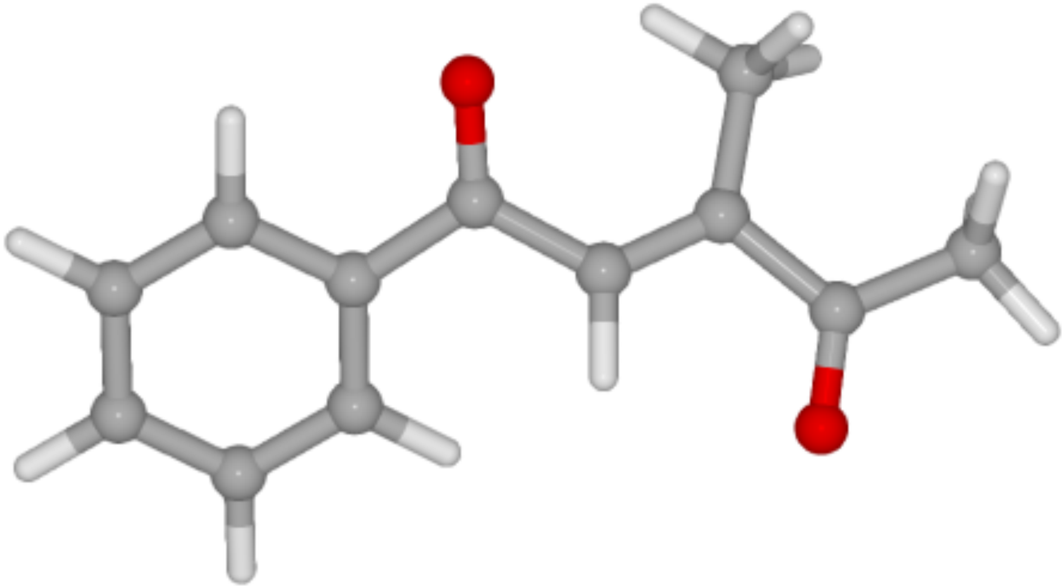

Electronic Energy = -812.270832 Hartrees

Supplementary Figure 8: Conformation of Bisphenol (***S4***)

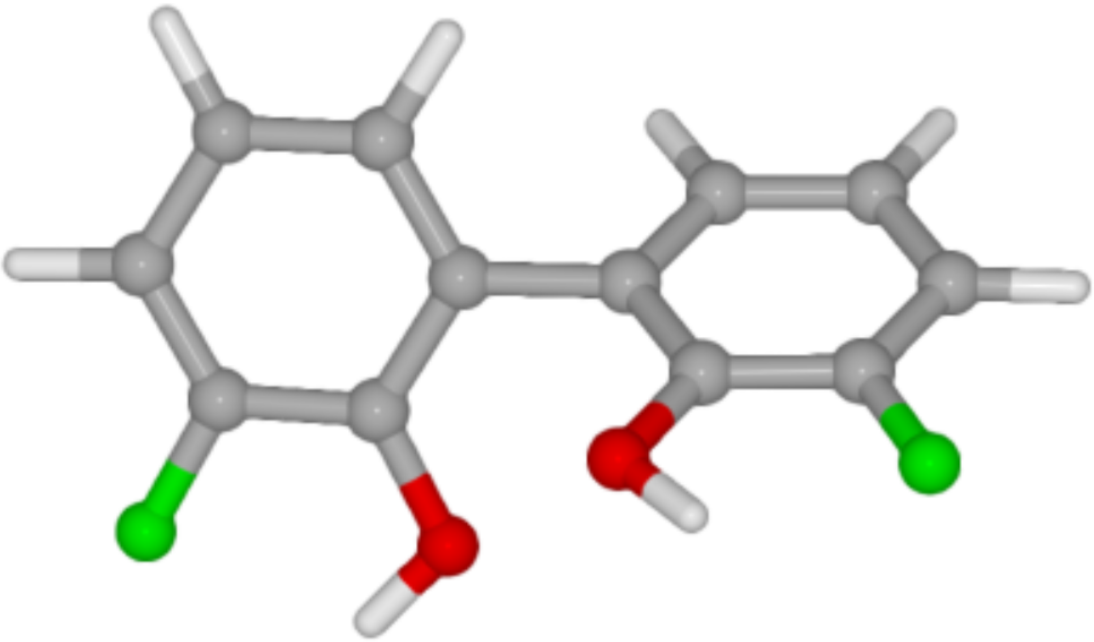

Electronic Energy = -812.270832 Hartrees

Supplementary Figure 9: Conformation of boronic acid **2a (S5)**.

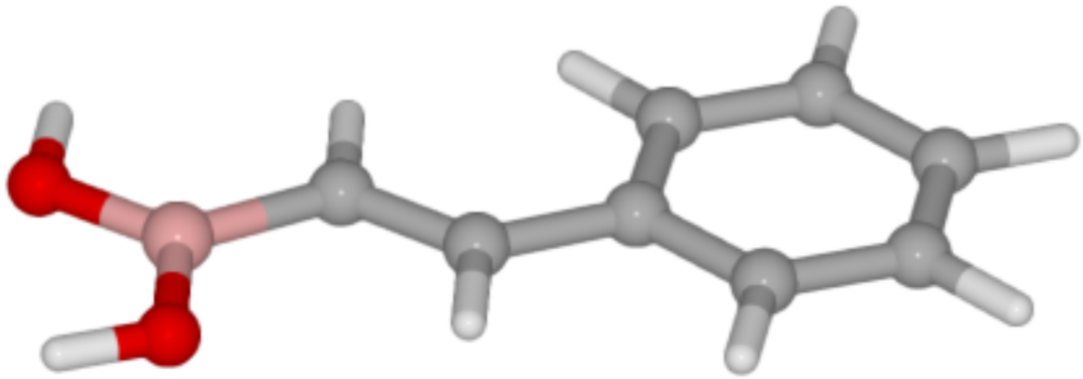

Electronic Energy = -485.722582 Hartrees

Supplementary Figure 10: Conformation of diol catalyst/boronic acid adduct (**S6**).

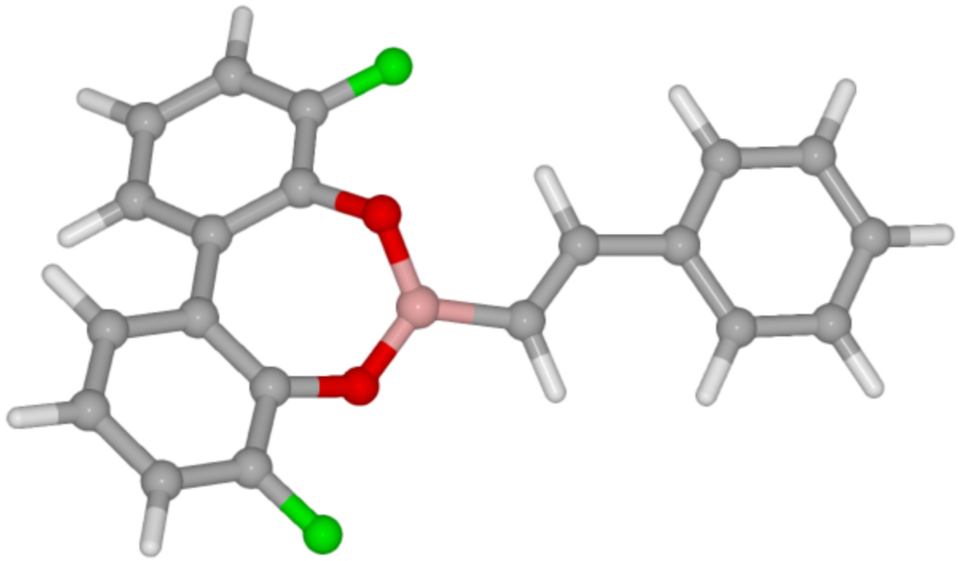

Electronic Energy = -1145.126639 Hartrees

Supplementary Figure 11: Reactants Precoordination.

Methyl Ketone Major quaternary product pathway through 5-exo TS

A) <u>MeCO SM (pre-LA/**la** intermediate) Major</u>

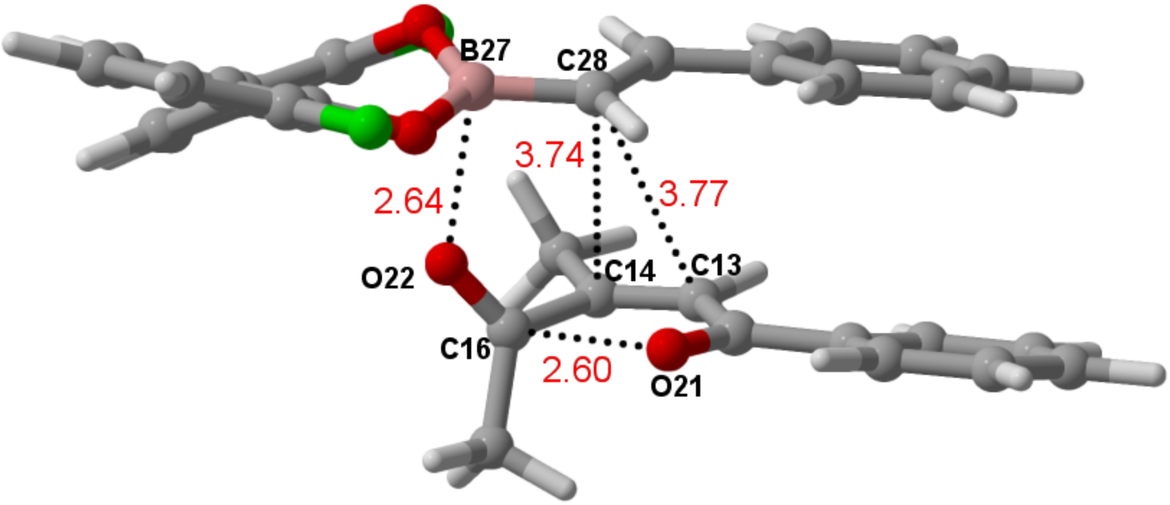

Supplementary Figure 12: Frontier molecular orbital overlap between HOMO (red/blue) and LUMO (purple/gold) orbitals.

Electronic Energy = -1760.157953 Hartrees
HOMO-LUMO gap = 3.81723366679564 eV

NBO Donor	NBO Acceptor	Stabilization Energy (kcal/mol)
40. LP (1) O 21	165. BD*(2) C 16- O 22	0.99
41. LP (2) O 21	165. BD*(2) C 16- O 22	5.76

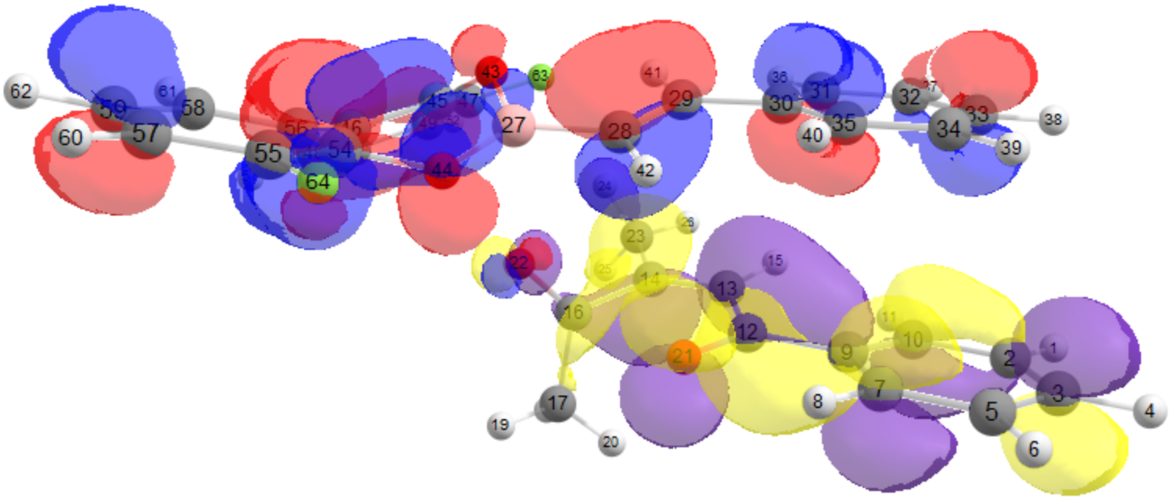

Supplementary Figure 13: Completed formation of Lewis acid/Lewis base complex.

MeCO-bound Major enantiomer Down LA/**la** intermediate
(THIS IS THE COMMON INTERMEDIATE FOR THE 5-EXO AND 6-ENDO TSs)

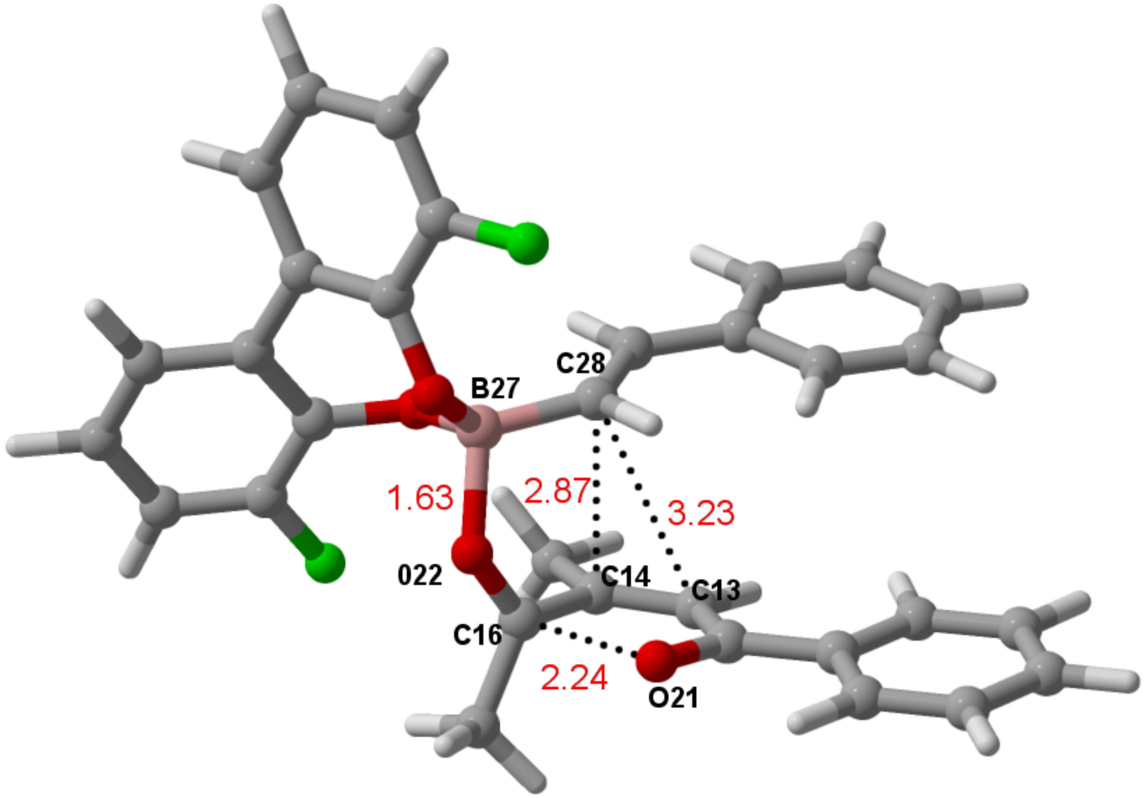

Supplementary Figure 14: Frontier molecular orbital overlap between HOMO (red/blue) and LUMO (purple/gold) orbitals.

Electronic Energy	= -1760.154741 Hartrees
HOMO-LUMO gap	= 2.8784189113108 eV

NBO Donor	NBO Acceptor	Stabilization Energy (kcal/mol)
43. LP (2) O 22	138. LV (2) B 27	26.82
41. LP (2) O 21	165. BD*(2) C 16- O 22	20.22

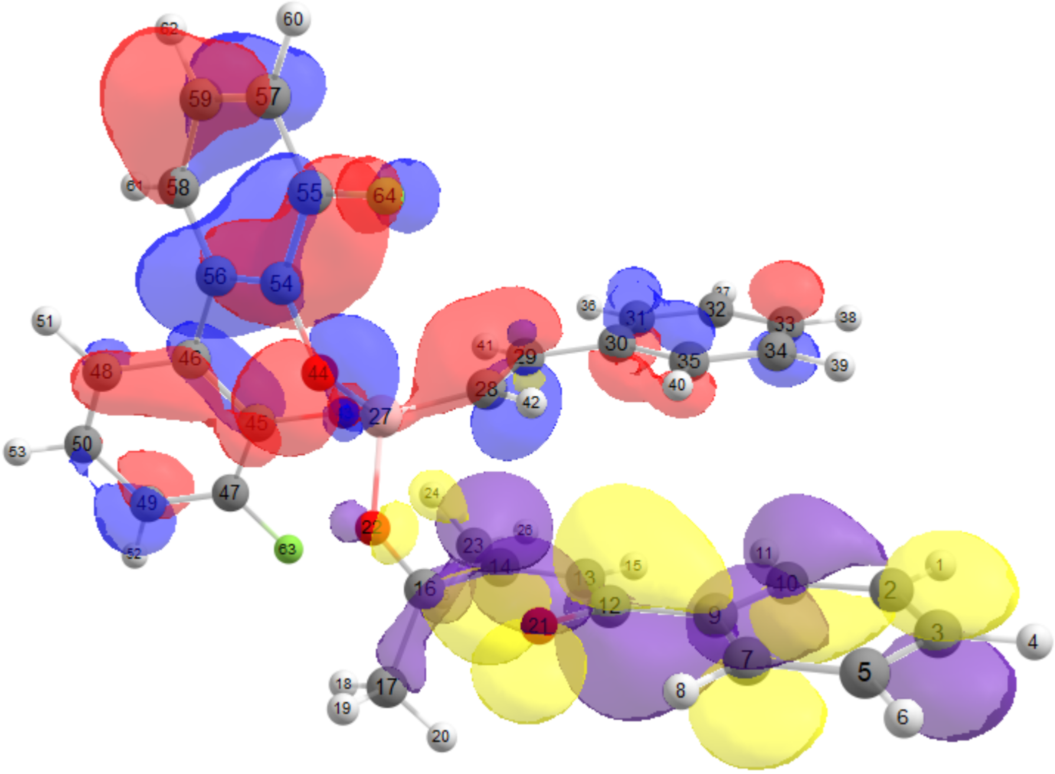

Supplementary Figure 15: 5-exo-trig Transition State from methyl ketone coordination for major enantiomer.

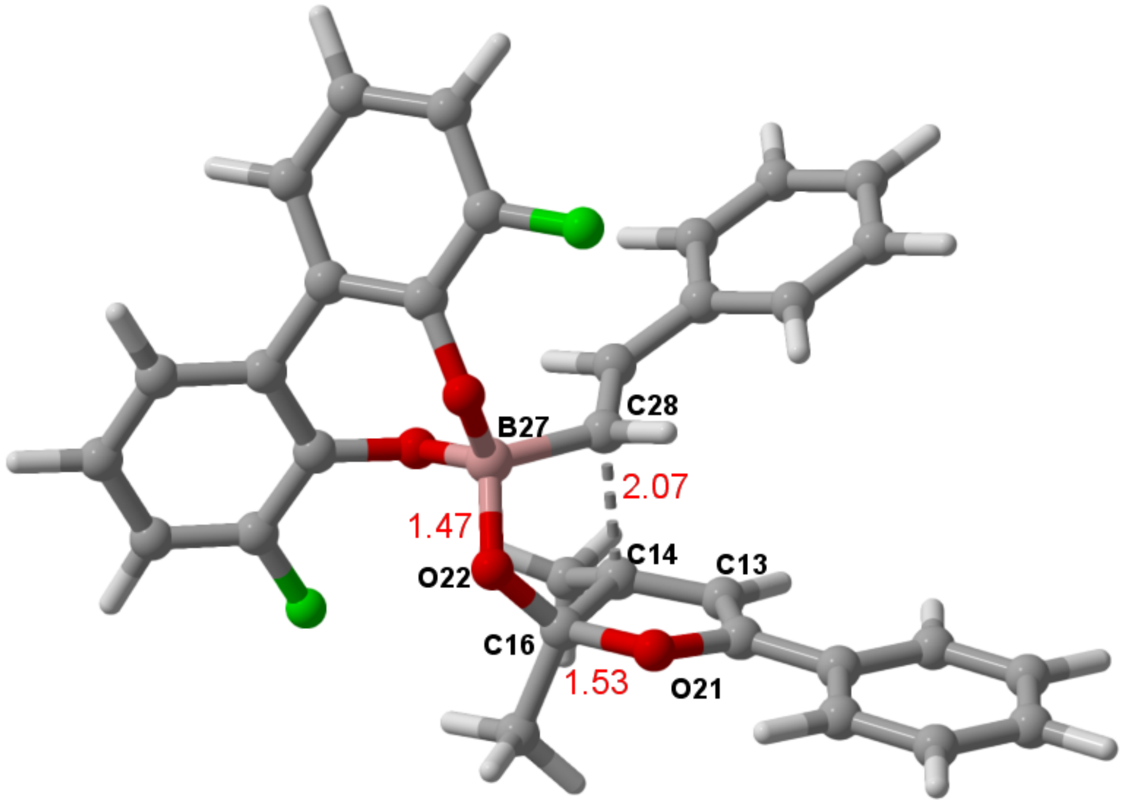

Electronic Energy = -1760.145389 Hartrees
HOMO-LUMO gap = 4.39876644914096 eV

Imaginary Frequency	= -230.97 cm^{-1}

NBO Donor	NBO Acceptor	Stabilization Energy (kcal/mol)
88. BD (1) B 27- C 28	137. LV (1) C 14	53.99
90. BD (2) C 28- C 29	137. LV (1) C 14	97.16

Supplementary Figure 16: C–C bond formation product from Transition State in Figure 15.

MeCO-bound Major enantiomer DOWN quaternary product .

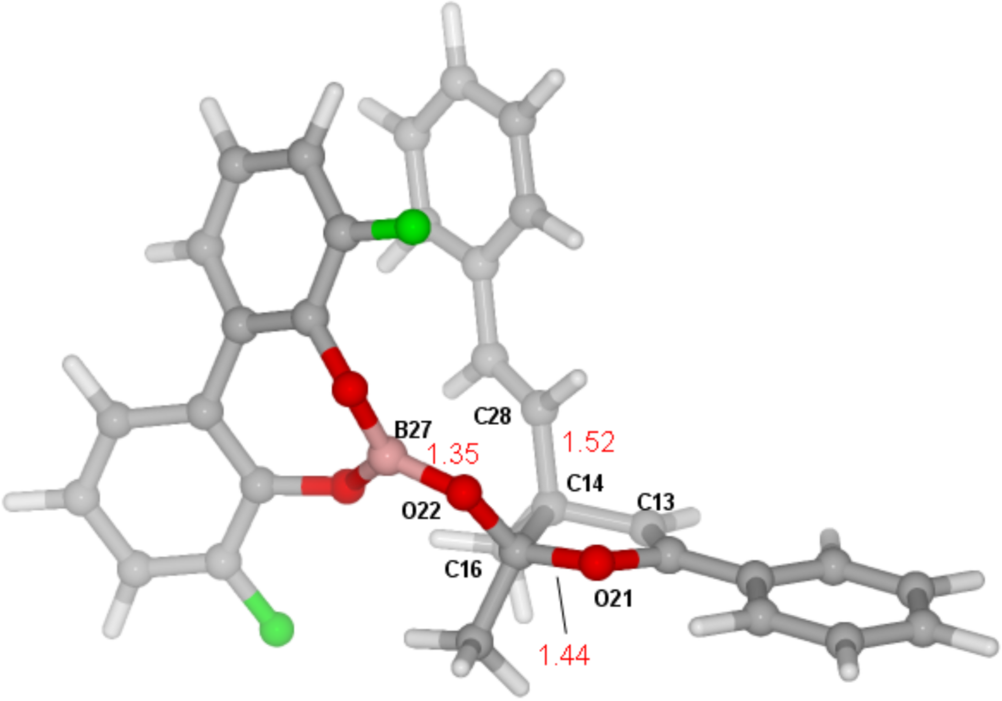

Electronic Energy = -1760.198674 Hartrees

Supplementary Figure 17: Energies reported are the sum of the zero-point corrected electronic energy and the thermal correction to Gibbs free energy. The first point on the energy profile diagram corresponds to the sum of the corrected energies of the organoborane and ene-diketone. The second step represents the pre-Lewis acid/Lewis base intermediate, where there is coordination with the carbonyl and the empty p-orbital of the organoborane.

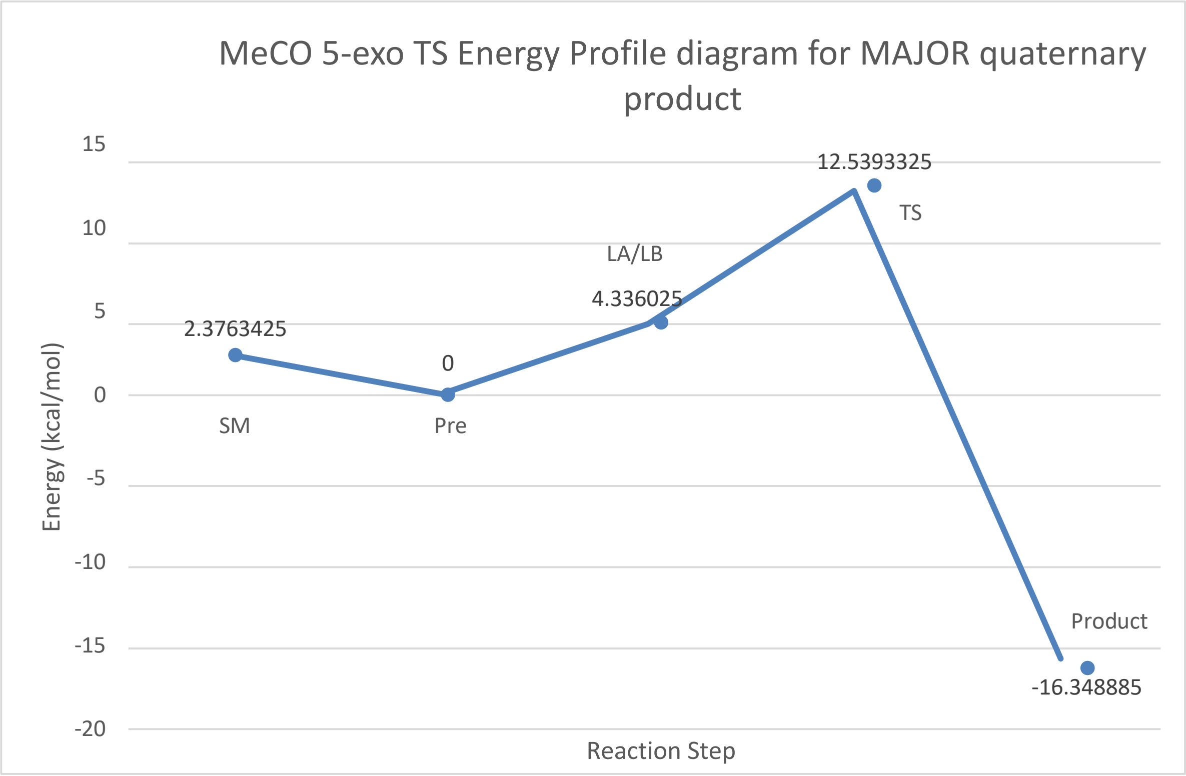

Supplementary Figure 18: This is the pathway Transition State for MeCO-bound 6-endo-trig Transition State (styrene is exo/"down") leading to minor tertiary product**.**

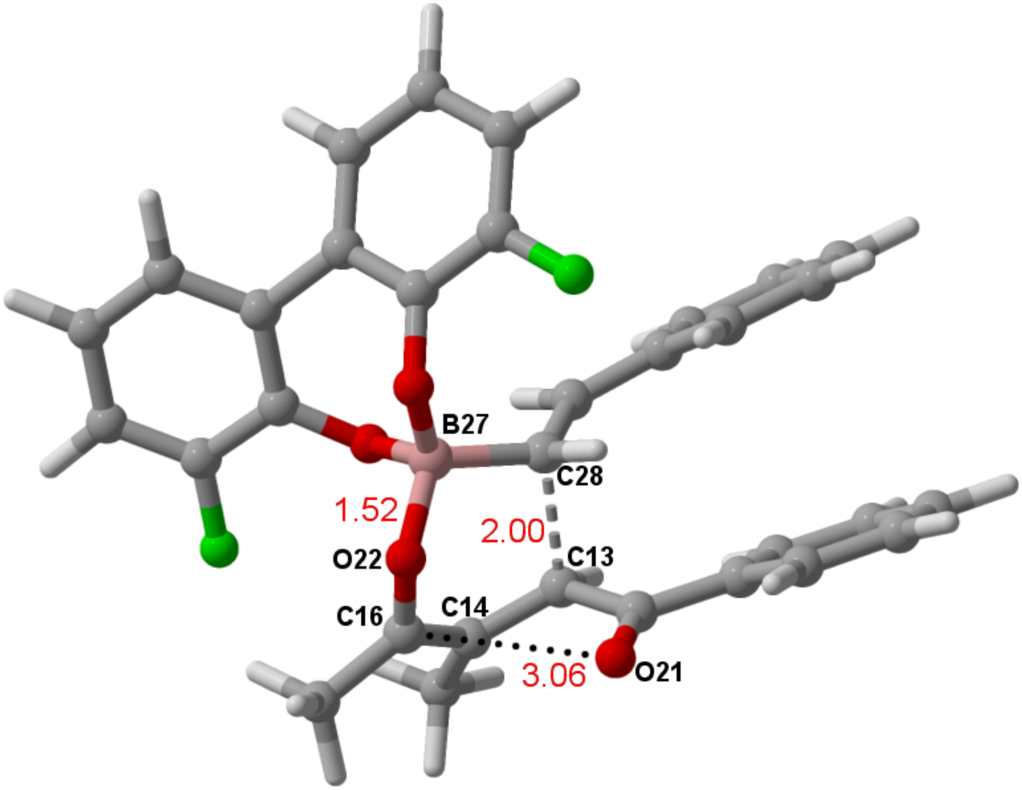

Electronic Energy	= -1760.123751 Hartrees
HOMO-LUMO gap	= 4.39876644914096 eV
Imaginary Frequency	= -386.37 cm^{-1}

Supplementary Figure 19: MeCO-bound MAJOR enantiomer DOWN Immediate tertiary product

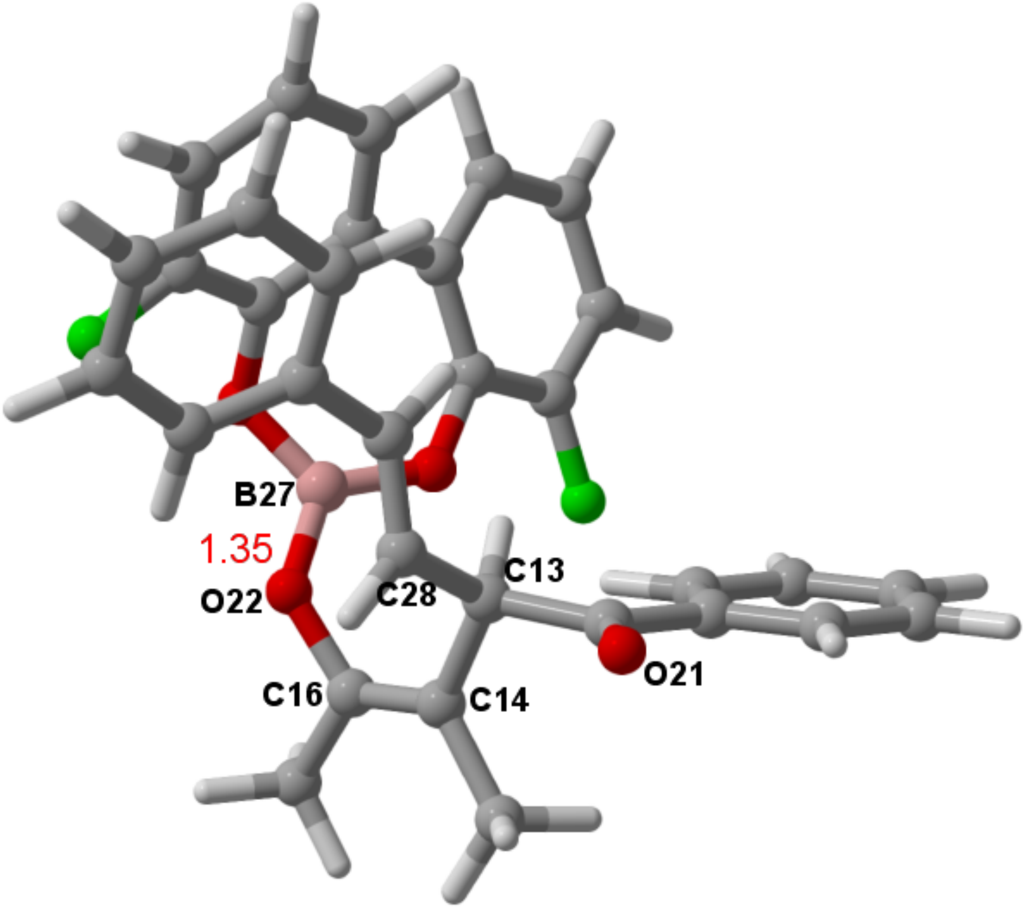

Electronic Energy = -1760.191248 Hartrees

Supplementary Figure 20: Energies reported are the sum of the zero-point corrected electronic energy and the thermal correction to Gibbs free energy. The first point on the energy profile diagram corresponds to the sum of the corrected energies of the organoborane and ene-diketone. The second step represents the pre-Lewis acid/Lewis base intermediate, where there is coordination with the carbonyl and the empty p-orbital of the organoborane.

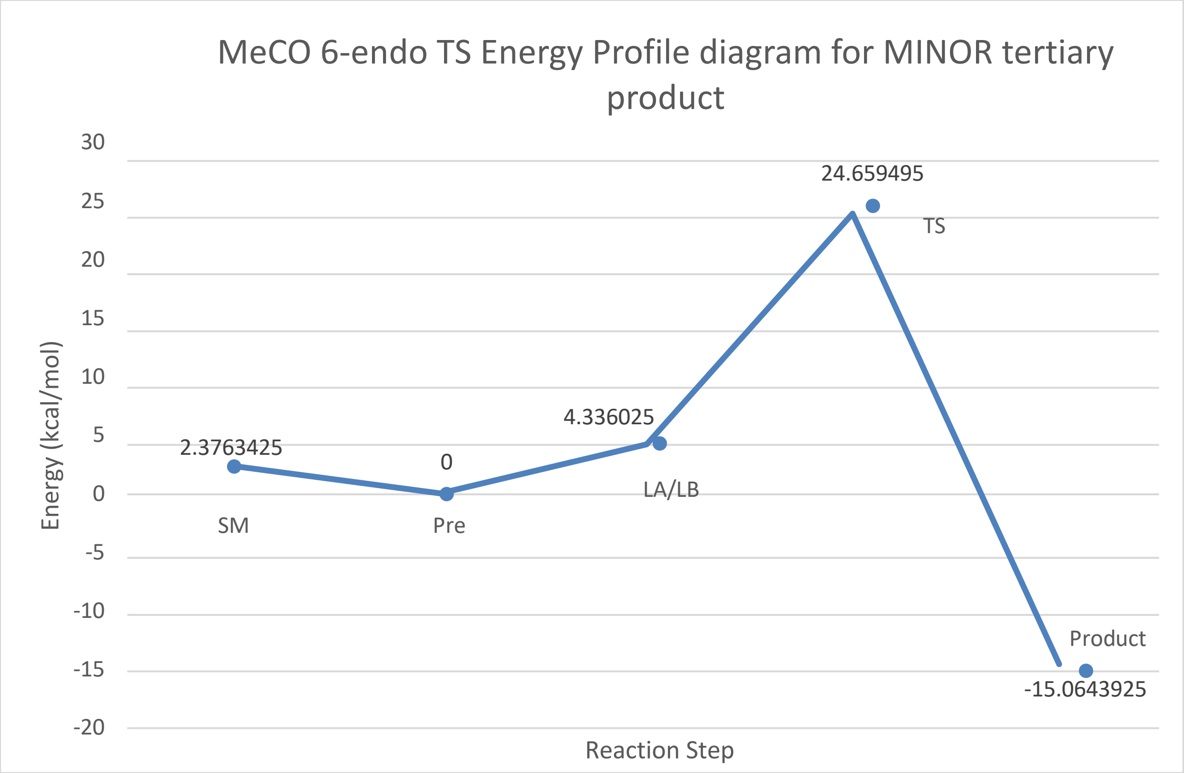

Supplementary Figure 21: MeCO-bound Major enantiomer Diels-Alder Pathway Pre-LA/**1a**.

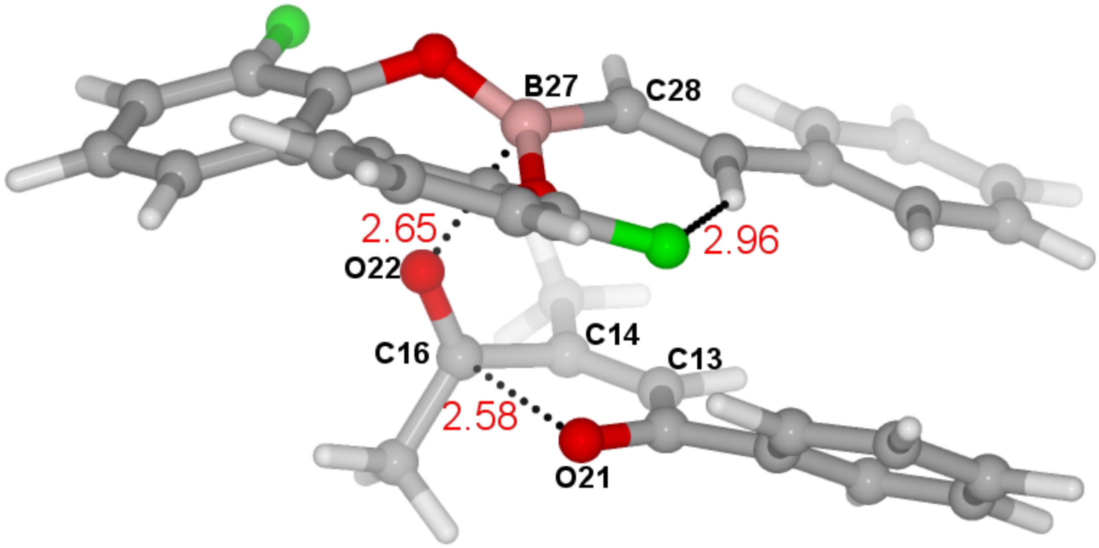

Supplementary Figure 22: Frontier molecular orbital overlap between HOMO (red/blue) and LUMO (red/blue) orbitals.

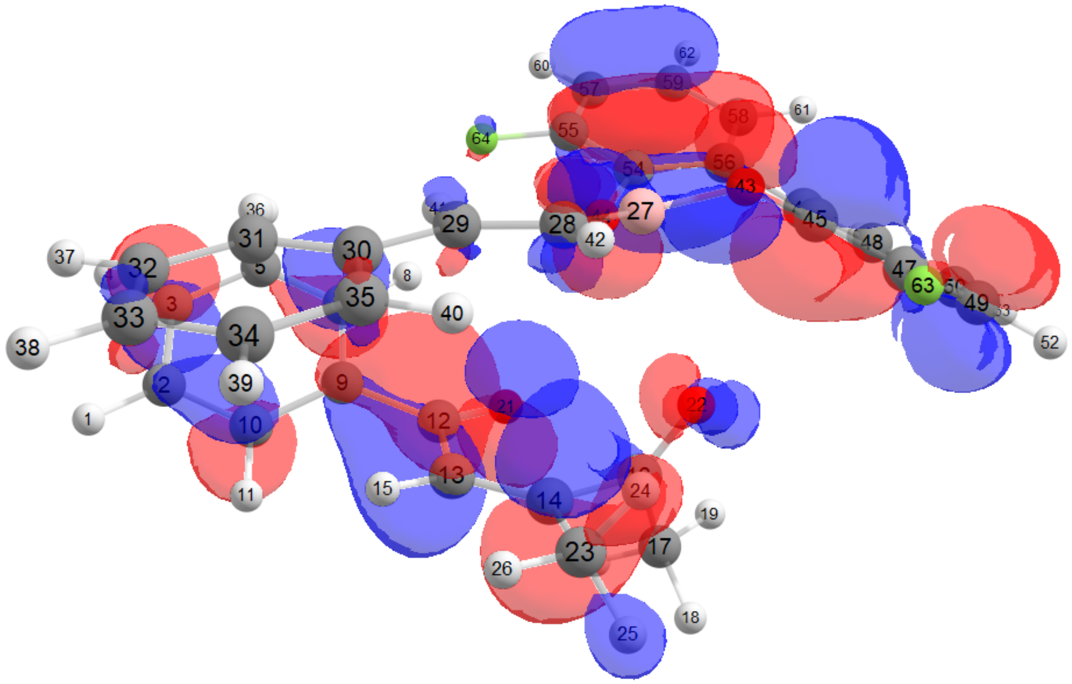

Electronic Energy	= -1760.156454 Hartrees
HOMO-LUMO gap	= 3.85044168300064 eV

NBO Donor	NBO Acceptor	Stabilization Energy (kcal/mol)
40. LP (1) O 21	165. BD*(2) C 16- O 22	1.06
41. LP (2) O 21	165. BD*(2) C 16- O 22	6.46

Supplementary Figure 23: Pre-transition state for MeCO-bound LA/**Ib** intermediate MAJOR enantiomer UP (endo styrene)

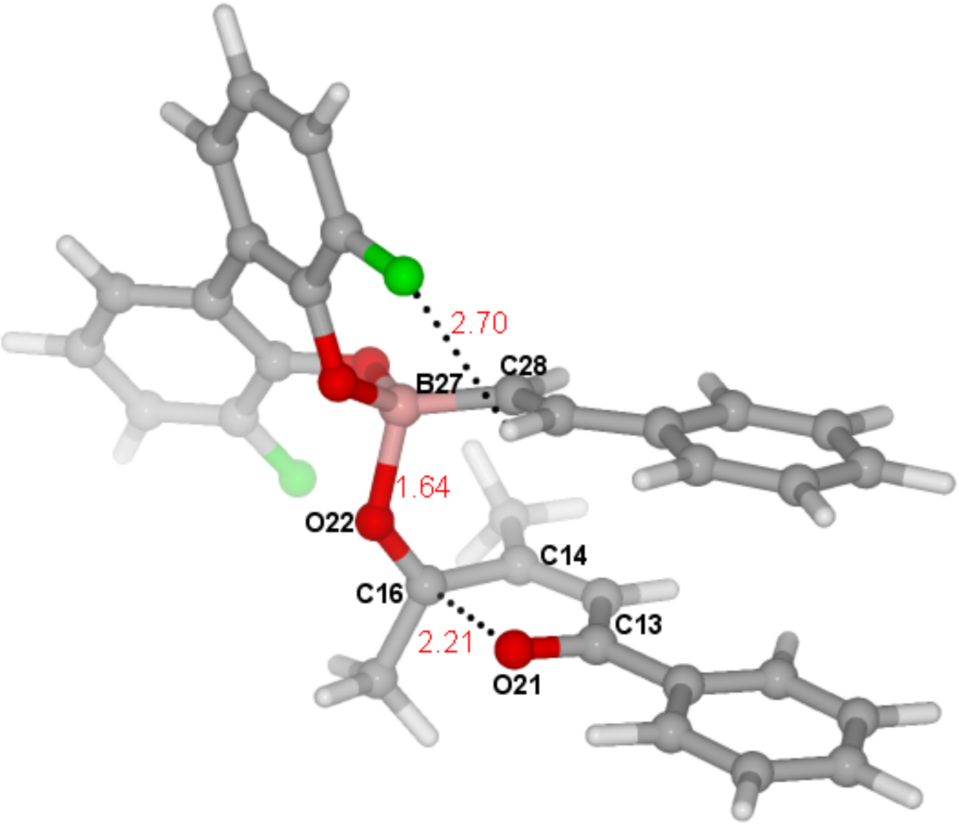

Supplementary Figure 24: Frontier molecular orbital overlap between HOMO (red/blue) and LUMO (purple/gold) orbitals.

Electronic Energy = -1760.158295 Hartrees
HOMO-LUMO gap = 2.930961436 eV

NBO Donor	NBO Acceptor	Stabilization Energy (kcal/mol)
41. LP (2) O 21	137. LV (1) C 16	101.73
42. LP (3) O 21	137. LV (1) C 16	39.66

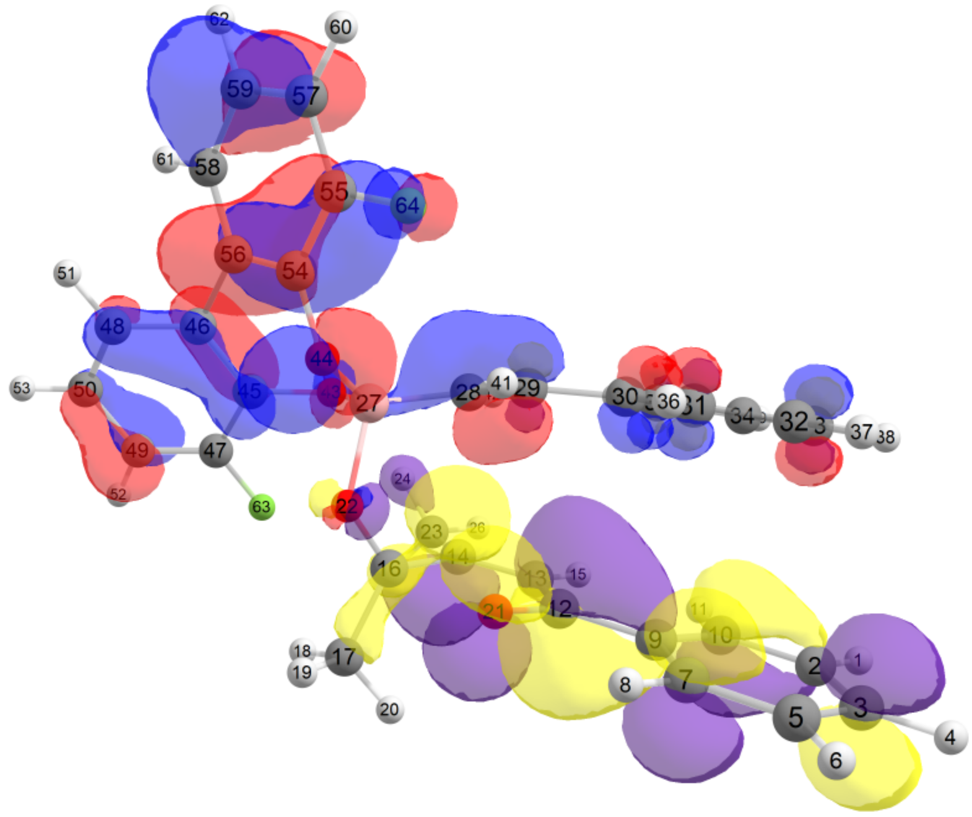

Supplementary Figure 25: MeCO-bound Transition State with 5-exo-trig for MAJOR enantiomer UP

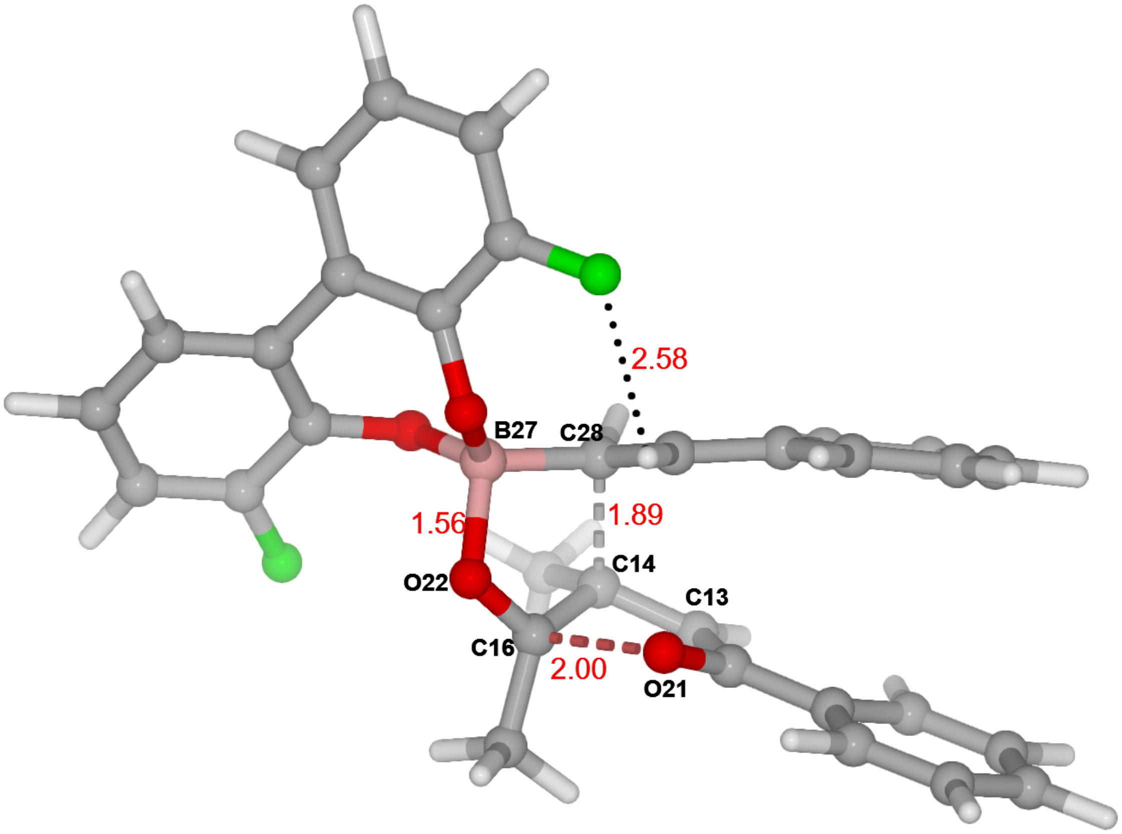

Electronic Energy	= -1760.137929 Hartrees
Imaginary Frequency	= -319.73 cm^{-1}

Supplementary Figure 26: MeCO-bound product from 5-exo-trig, MAJOR enantiomer, UP.

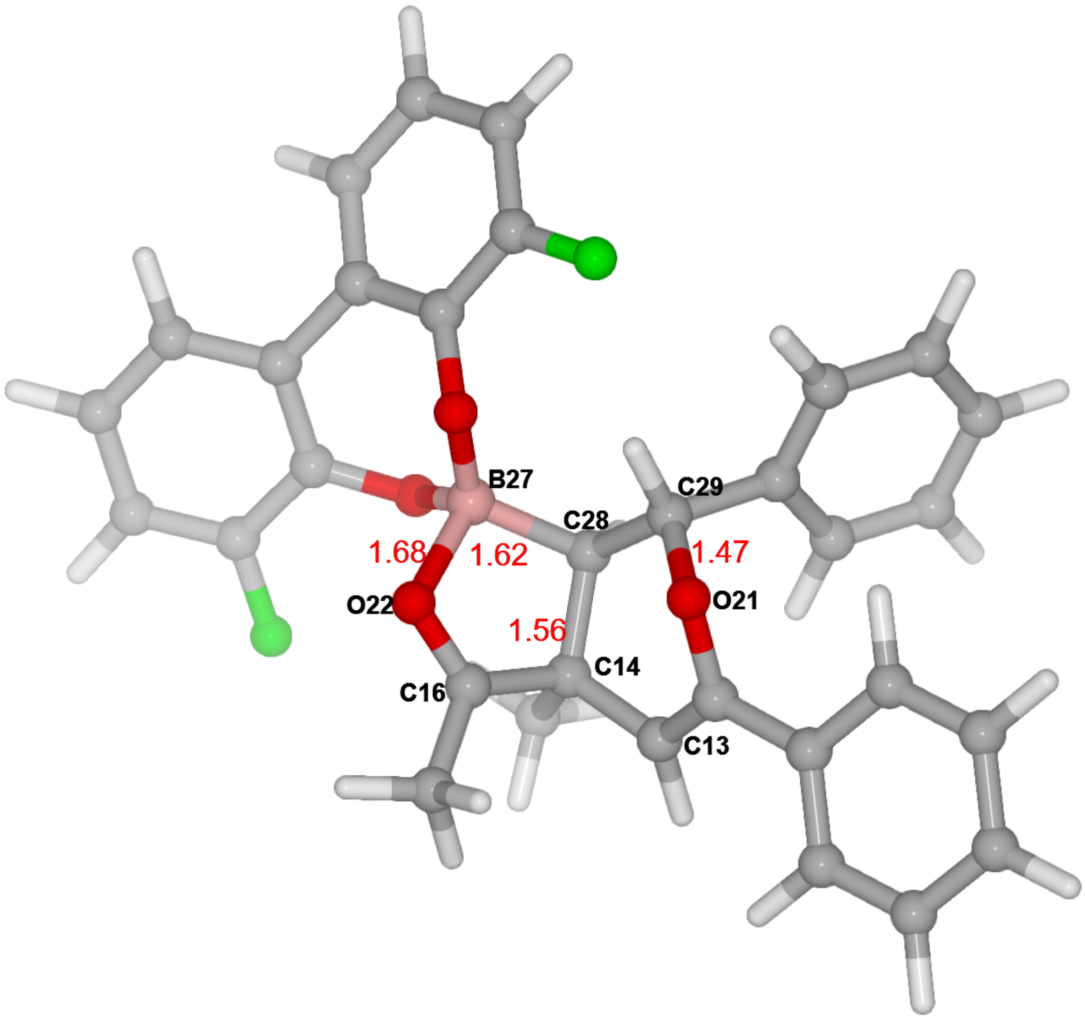

Electronic Energy = -1760.1751 Hartrees

Supplementary Figure 27: Energies reported are the sum of the zero-point corrected electronic energy and the thermal correction to Gibbs free energy. The first point on the energy profile diagram corresponds to the sum of the corrected energies of the organoborane and ene-diketone. The second step represents the pre-Lewis acid/Lewis base intermediate, where there is coordination with the carbonyl and the empty p-orbital of the organoborane.

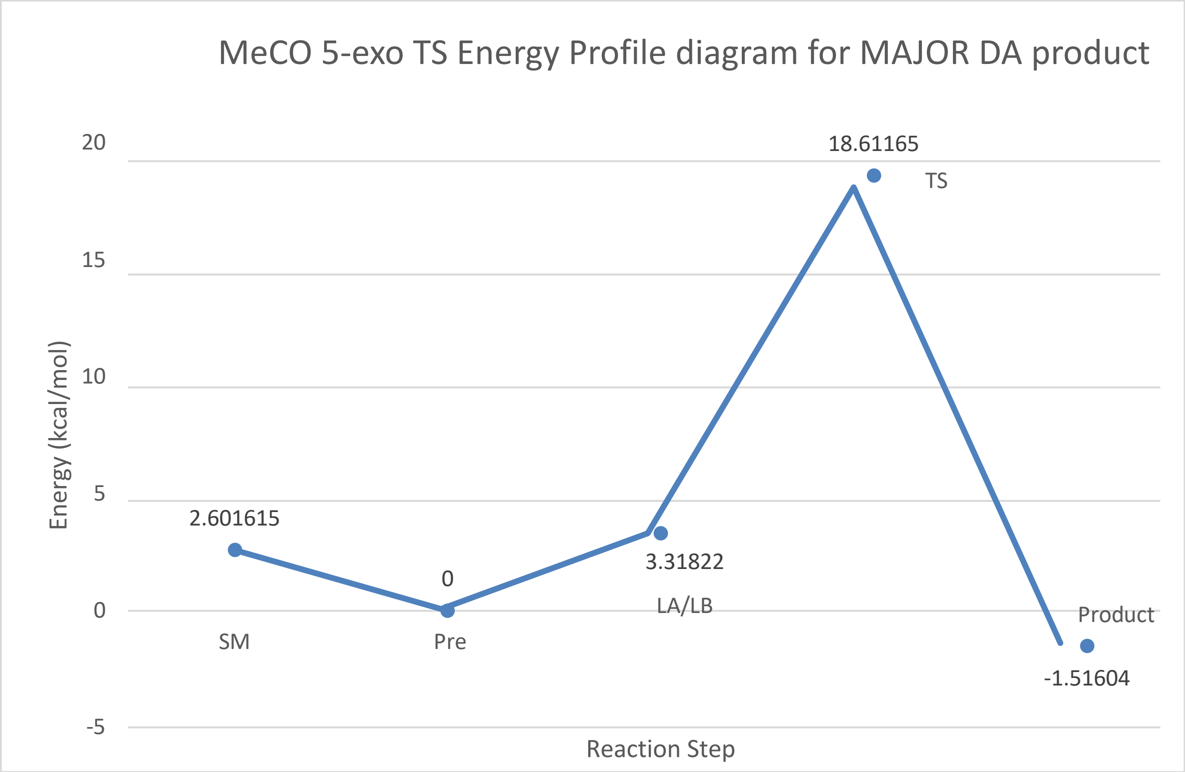

Supplementary Figure 28: MeCO-bound 6-endo-trig Transition State, MAJOR enantiomer, UP.

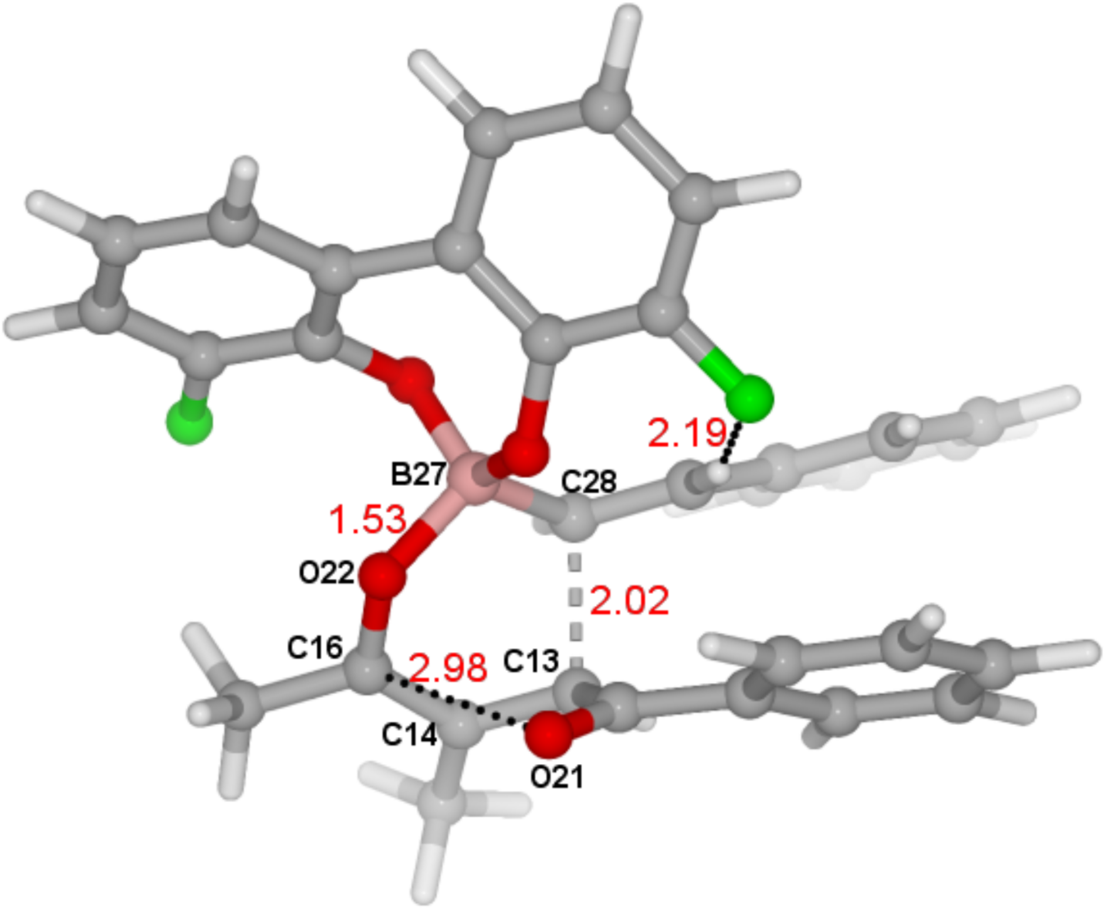

Electronic Energy	= -1760.126247 Hartrees
Imaginary Frequency	= -321.75 cm^{-1}

Supplementary Figure 29: MeCO-bound tertiary product, MAJOR enantiomer, UP.

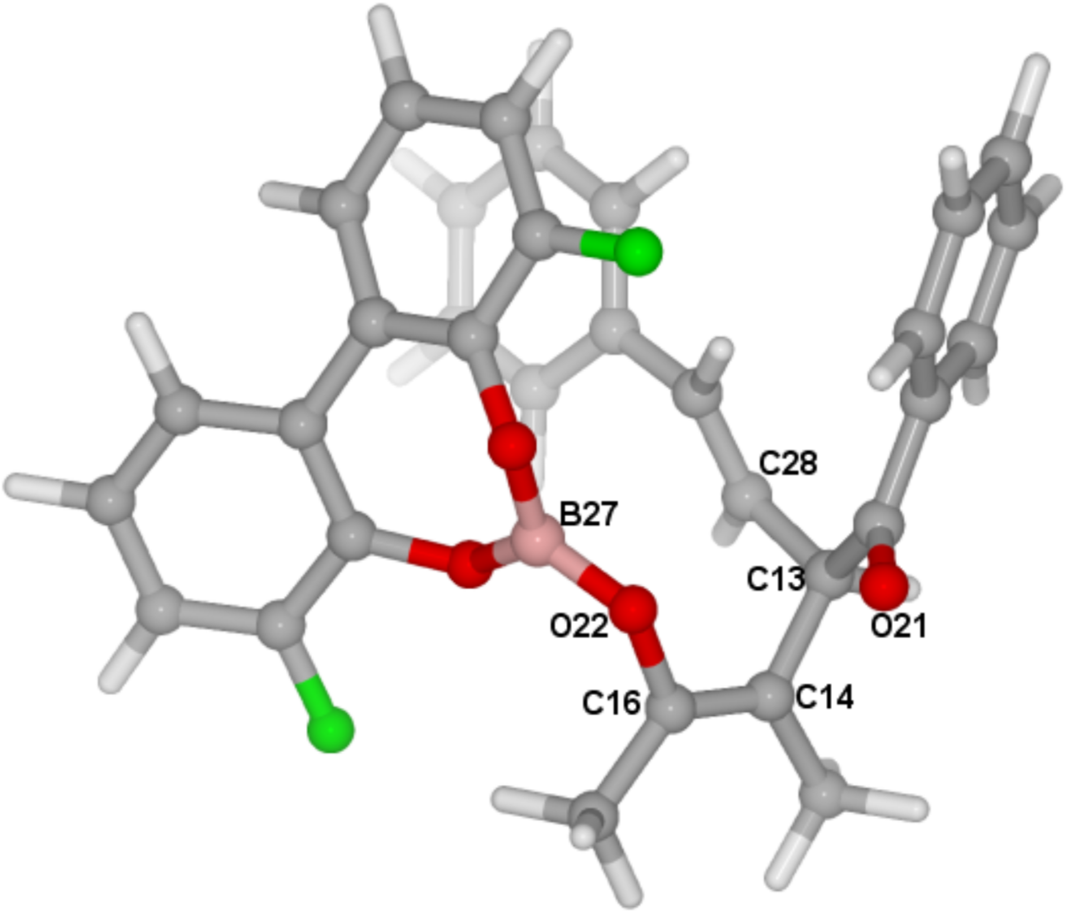

Electronic Energy = -1760.193957 Hartrees

Supplementary Figure 30: Energies reported are the sum of the zero-point corrected electronic energy and the thermal correction to Gibbs free energy. The first point on the energy profile diagram corresponds to the sum of the corrected energies of the organoborane and ene-diketone. The second step represents the pre-Lewis acid/Lewis base intermediate, where there is coordination with the carbonyl and the empty p-orbital of the organoborane.

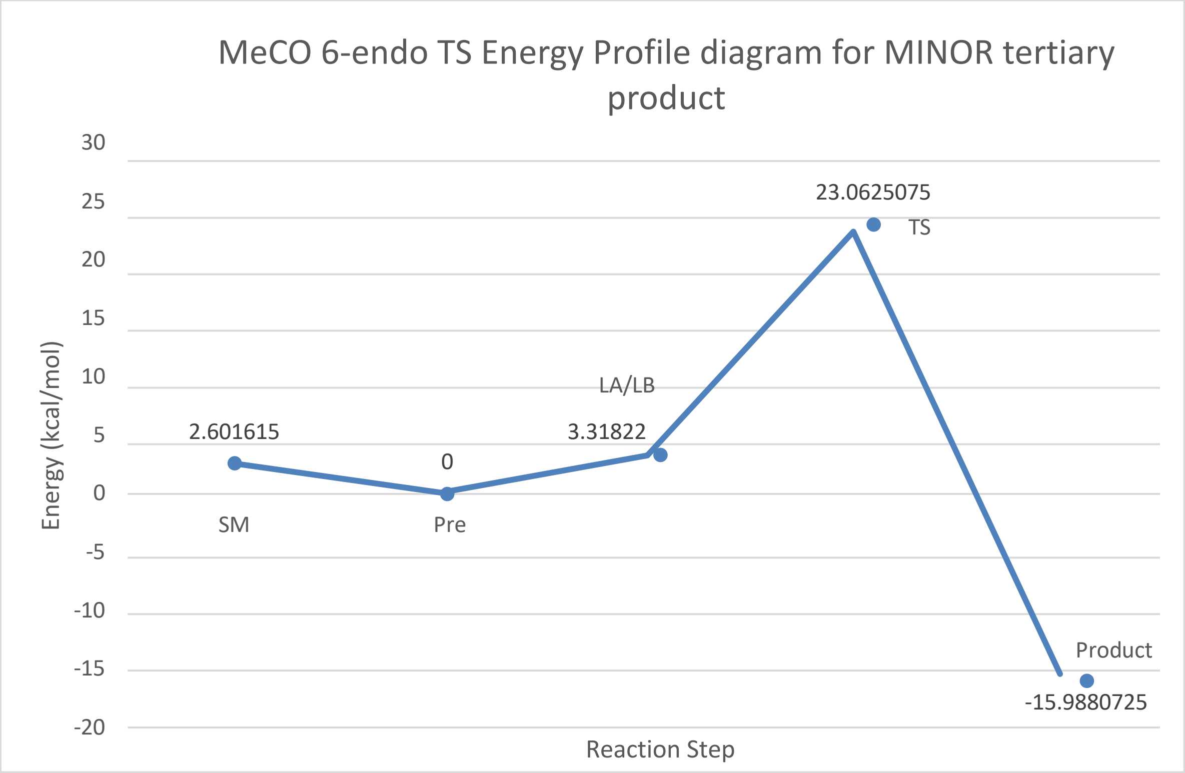

Supplementary Figure 31: Pre-LA/**1a** for MeCO-bound Minor enantiomer Diels-Alder pathway (SM)

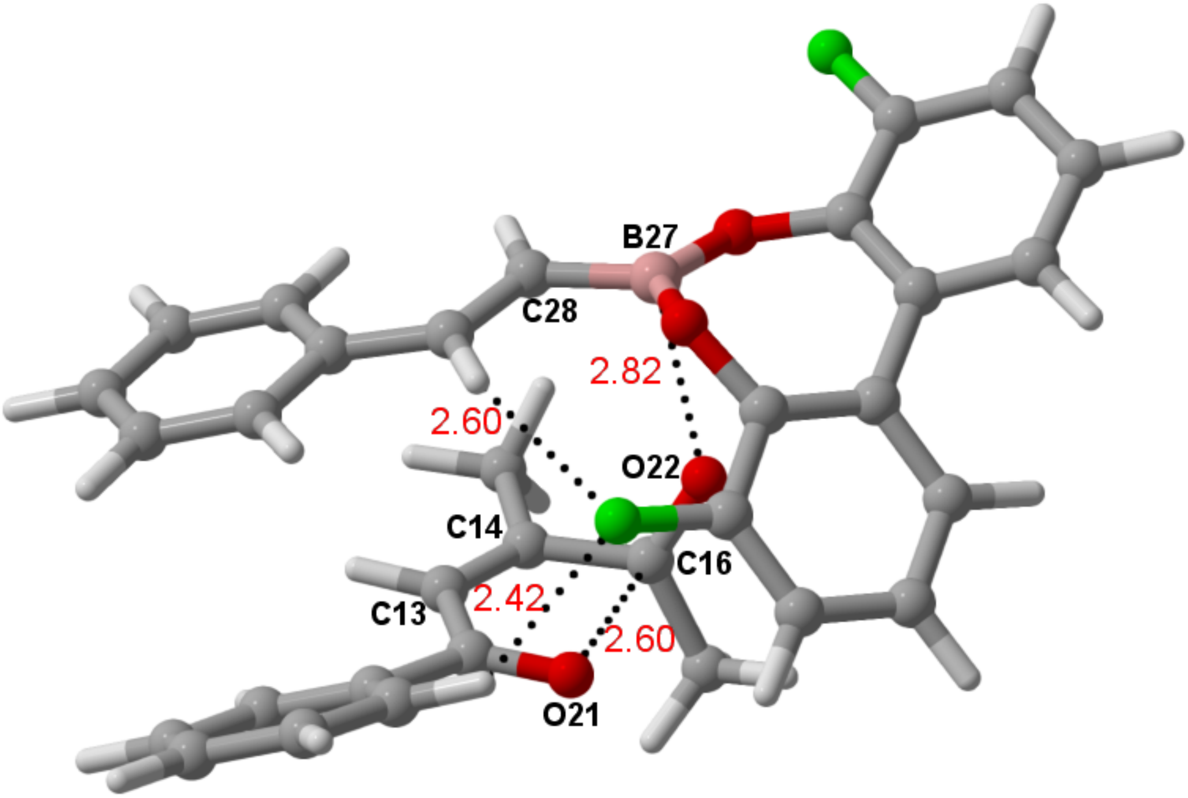

Supplementary Figure 32: Frontier molecular orbital overlap between HOMO (red/blue) and LUMO (purple/gold) orbitals.

Electronic Energy	= -1760.157953 Hartrees
HOMO-LUMO gap	= 3.7497999504784 eV

NBO Donor	NBO Acceptor	Stabilization Energy (kcal/mol)
40. LP (1) O 21	165. BD*(2) C 16- O 22	0.9
41. LP (2) O 21	165. BD*(2) C 16- O 22	5.63

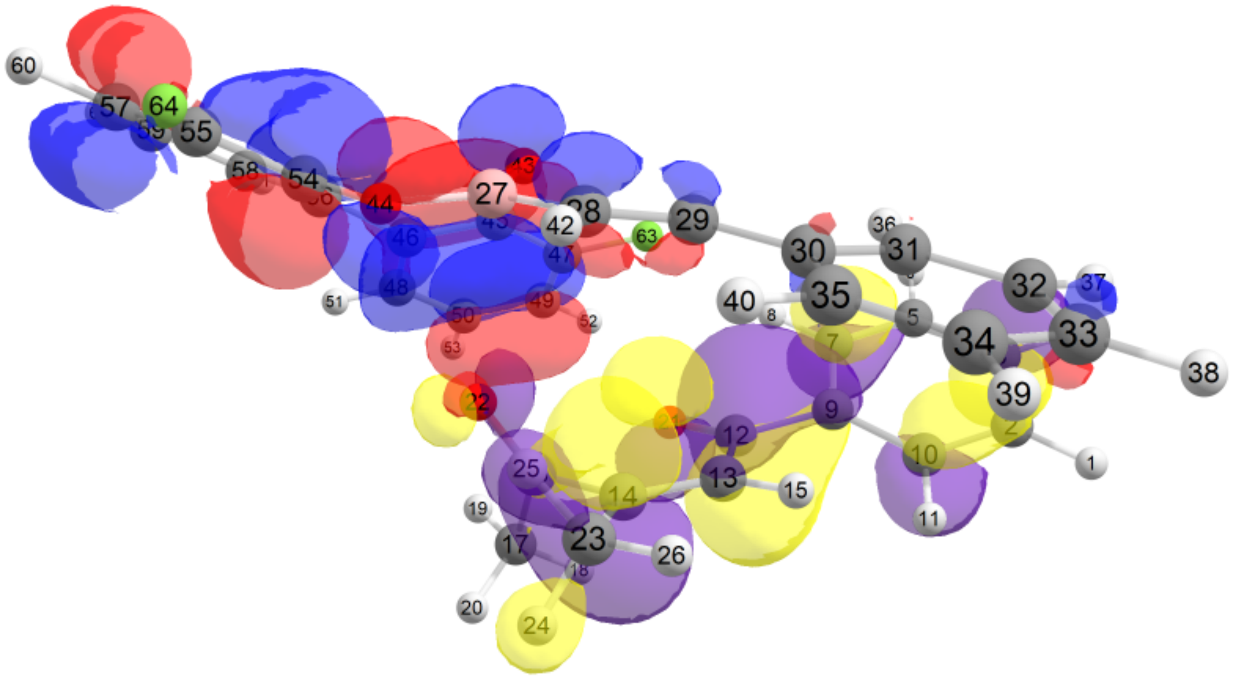

Supplementary Figure 33: MeCO-bound LA/**la**/**lb** Minor enantiomer, DOWN.

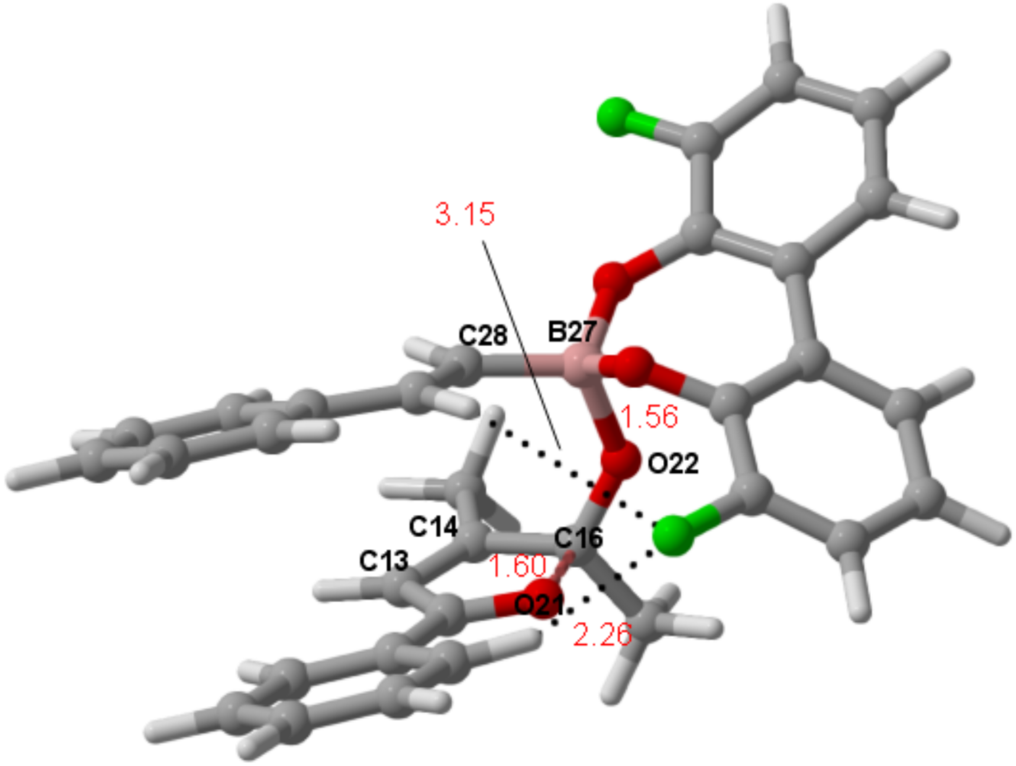

Supplementary Figure 34: Frontier molecular orbital overlap between HOMO (red/blue) and LUMO (purple/gold) orbitals.

Electronic Energy = -1760.154741 Hartrees
HOMO-LUMO gap = 2.95988966345904 eV

NBO Donor	NBO Acceptor	Stabilization Energy (kcal/mol)
40. LP (1) O 21	165. BD*(2) C 16- O 22	3.48
41. LP (2) O 21	165. BD*(2) C 16- O 22	28.41

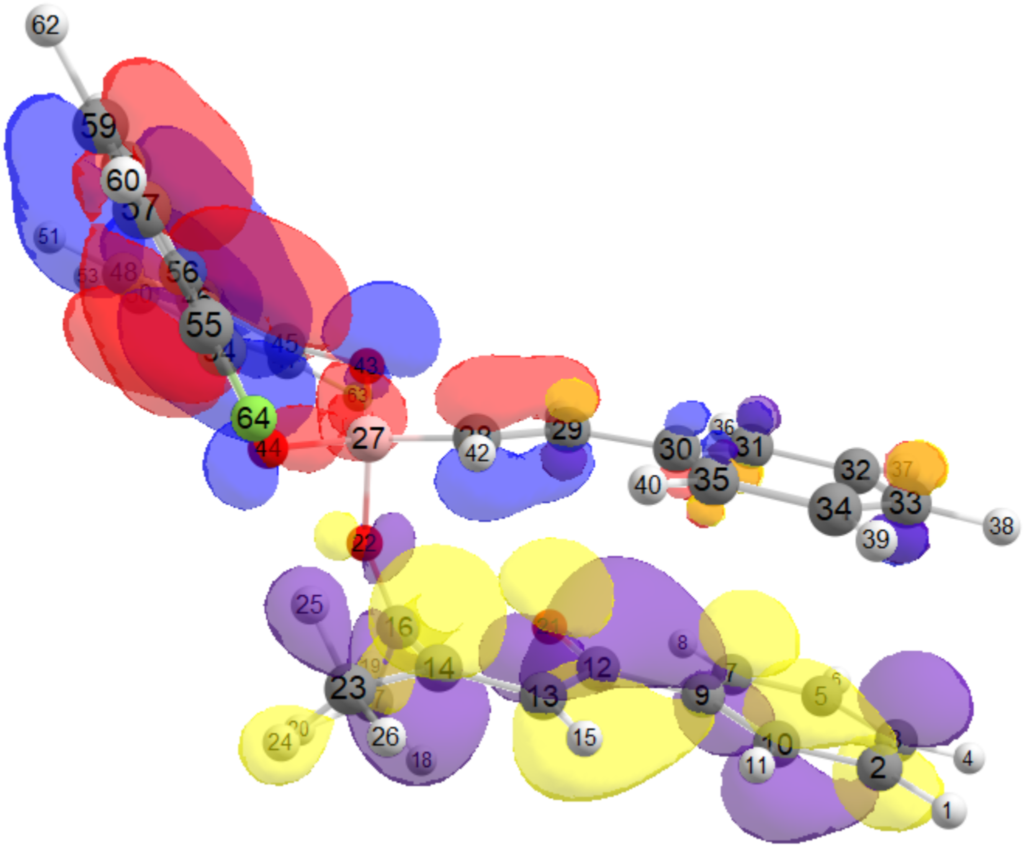

Supplementary Figure 35: Transition State for MeCO-bound 5-exo-trig, MINOR enantiomer, DOWN.

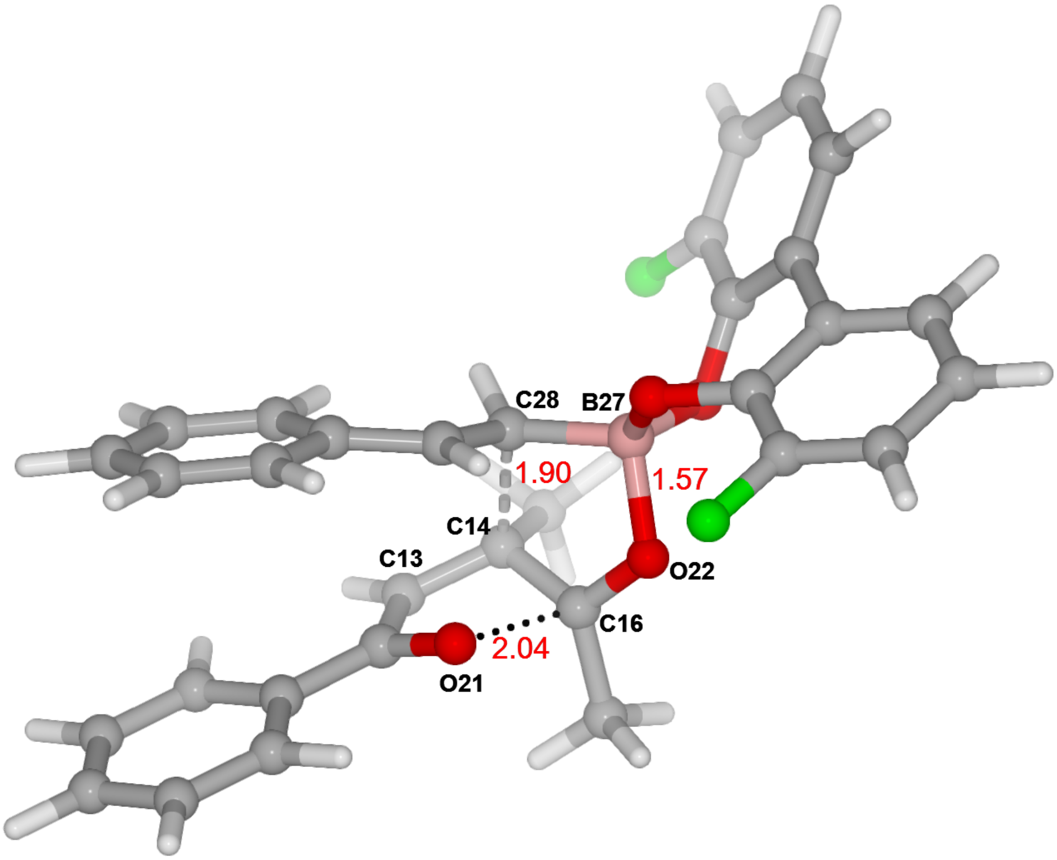

Electronic Energy	= -1760.145389 Hartrees
Imaginary Frequency	= -332.58 cm^{-1}

Supplementary Figure 36: MeCO-bound quaternary product from 5-exo-trig, MINOR enantiomer, DOWN.

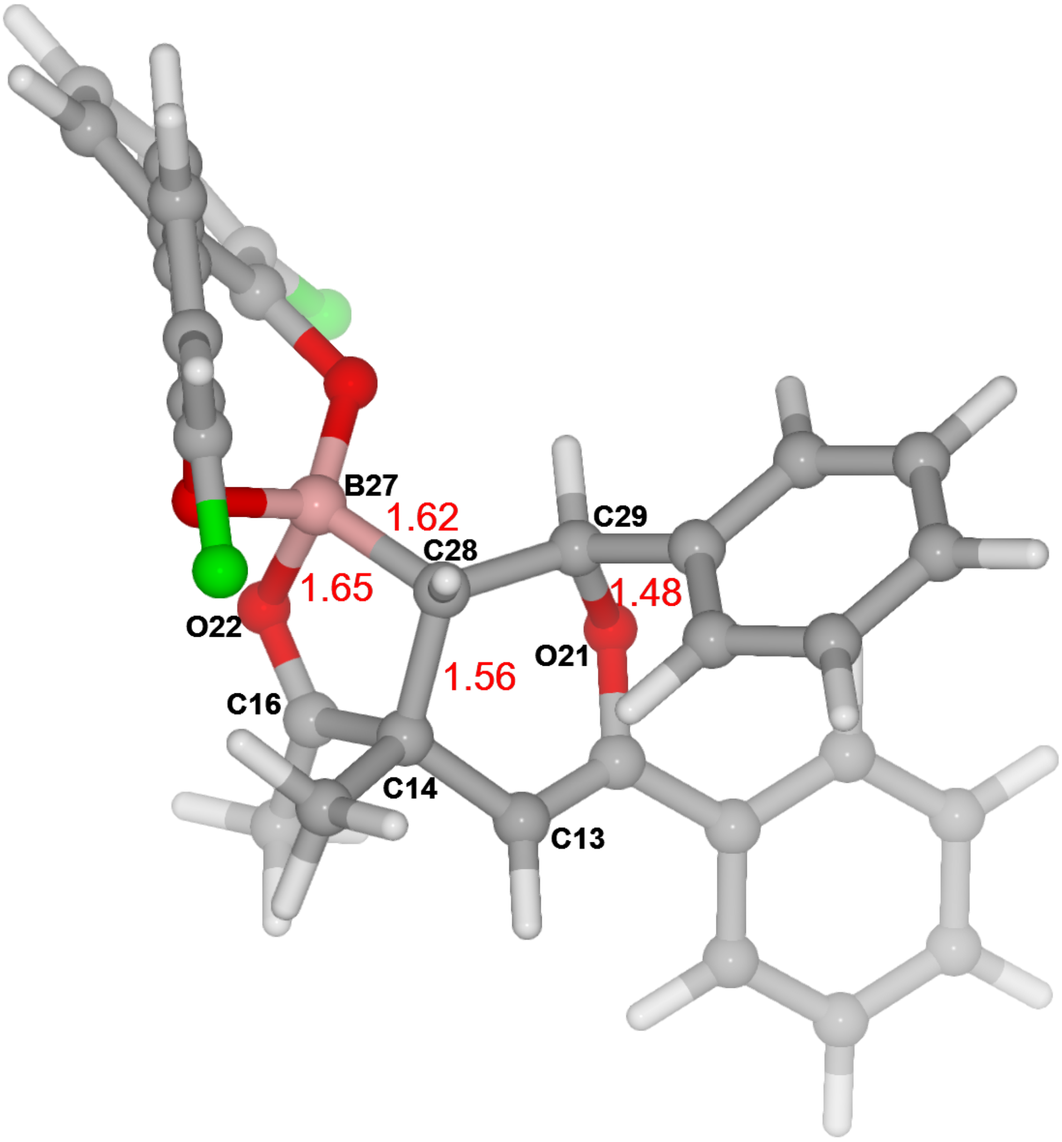

Electronic Energy = -1760.198674 Hartrees

Supplementary Figure 37: Energies reported are the sum of the zero-point corrected electronic energy and the thermal correction to Gibbs free energy. The first point on the energy profile diagram corresponds to the sum of the corrected energies of the organoborane and ene-diketone. The second step represents the pre-Lewis acid/Lewis base intermediate, where there is coordination with the carbonyl and the empty p-orbital of the organoborane.

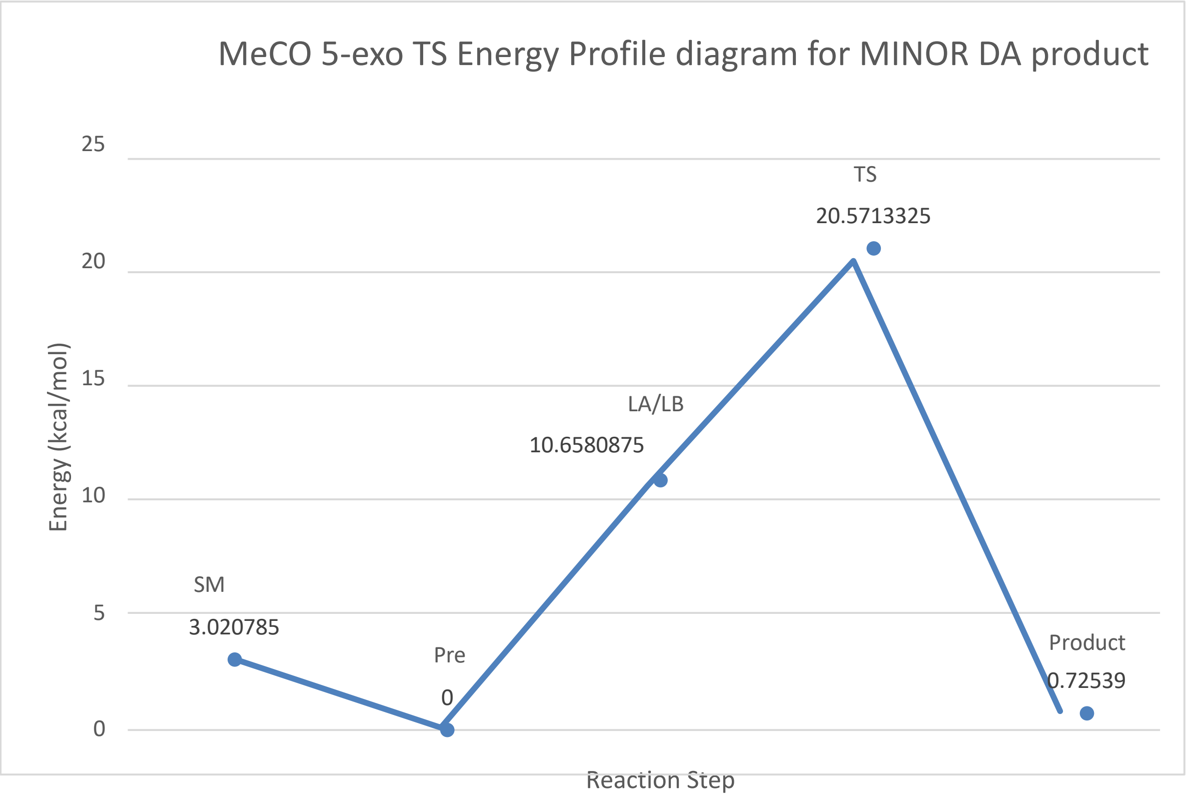

Supplementary Figure 38: Transition State for MeCO-bound 6-endo-trig, MINOR enantiomer, DOWN.

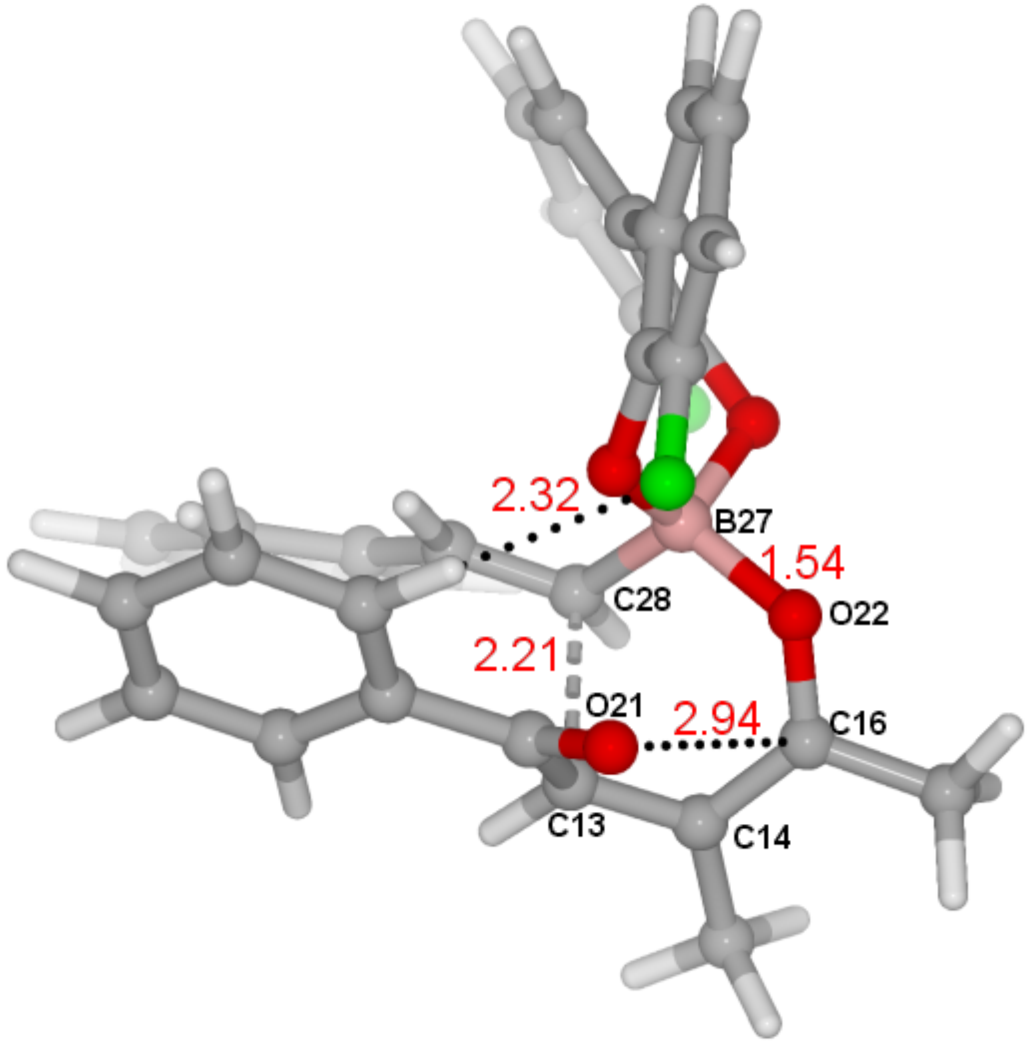

Electronic Energy	= -1760.123751 Hartrees
Imaginary Frequency	= -145.58 cm^{-1}

Supplementary Figure 39: MeCO-bound tertiary product, MINOR enantiomer, DOWN.

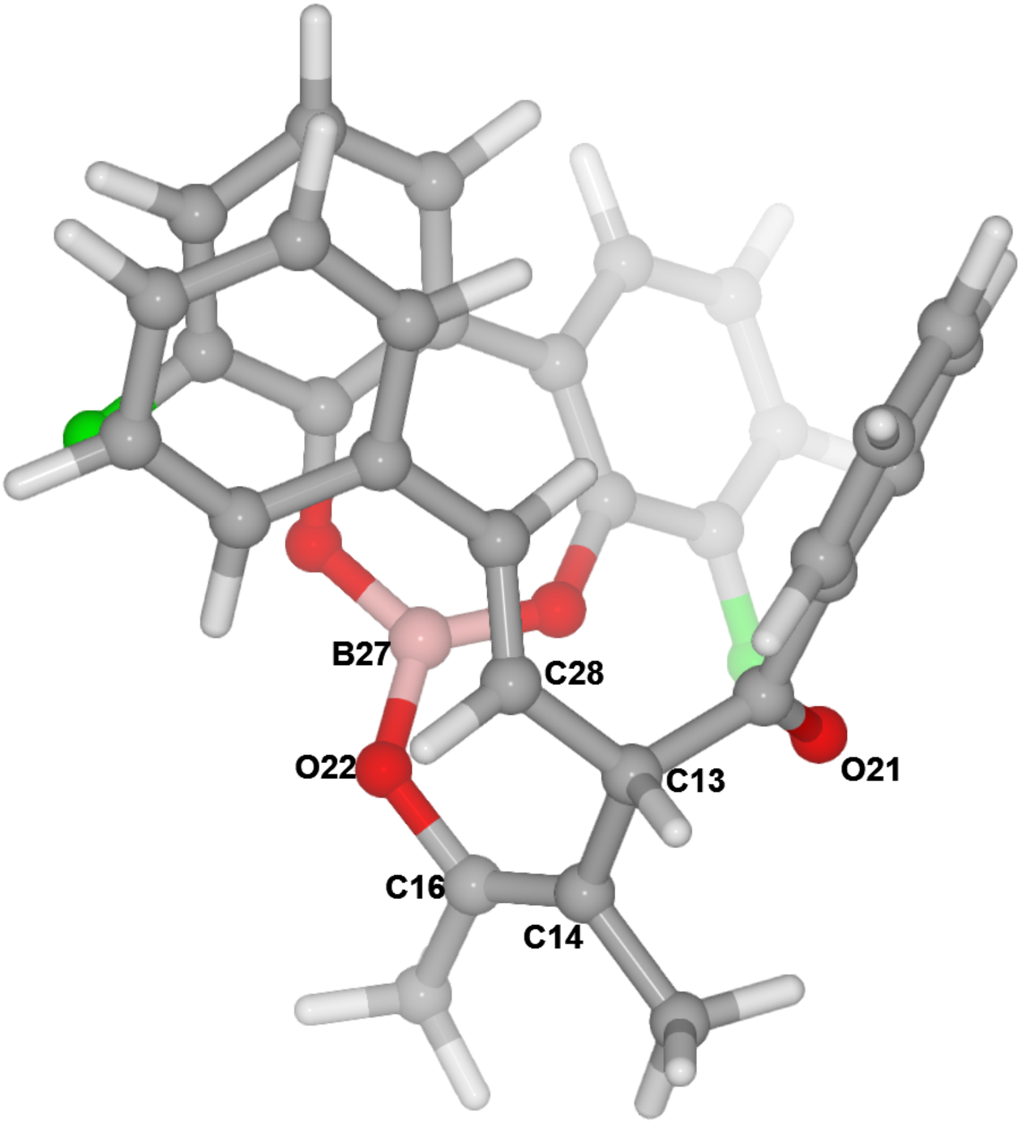

Electronic Energy = -1760.191248 Hartrees

Supplementary Figure 40: Energies reported are the sum of the zero-point corrected electronic energy and the thermal correction to Gibbs free energy. The first point on the energy profile diagram corresponds to the sum of the corrected energies of the organoborane and ene-diketone. The second step represents the pre-Lewis acid/Lewis base intermediate, where there is coordination with the carbonyl and the empty p-orbital of the organoborane.

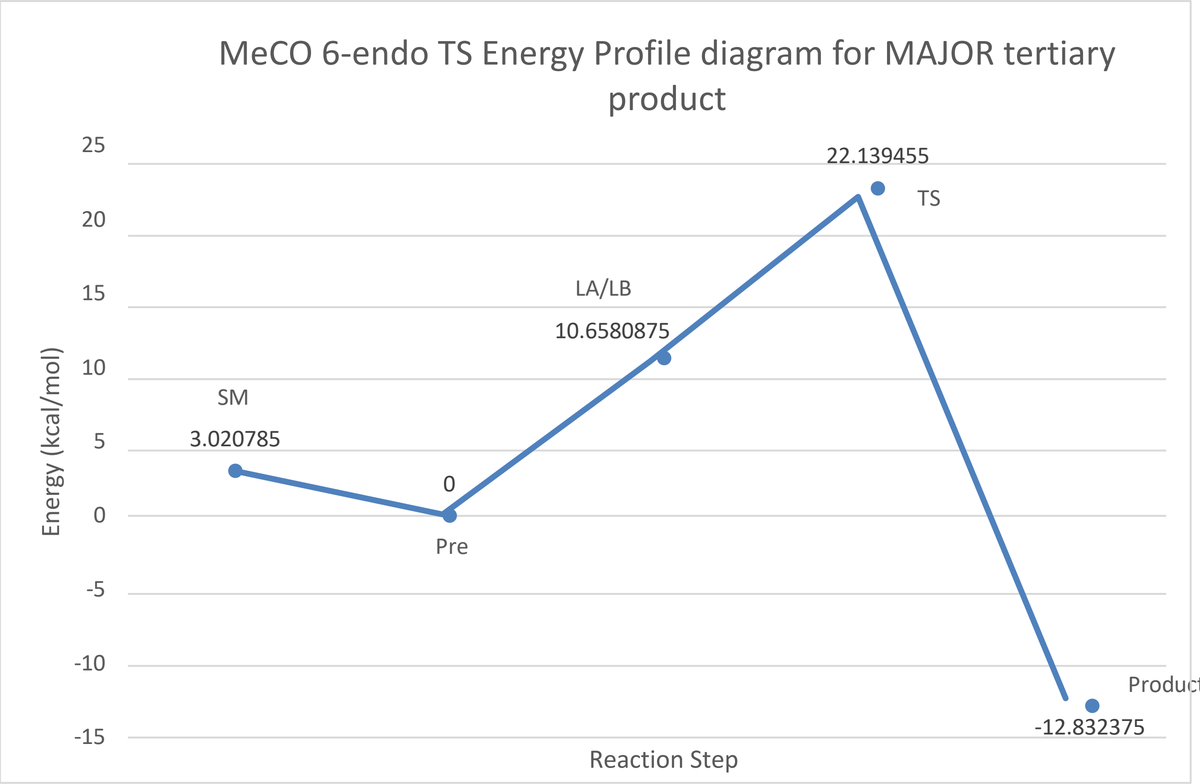

Supplementary Figure 41: MeCO-bound Pre-LA/**la** intermediate, Minor enantiomer, UP.

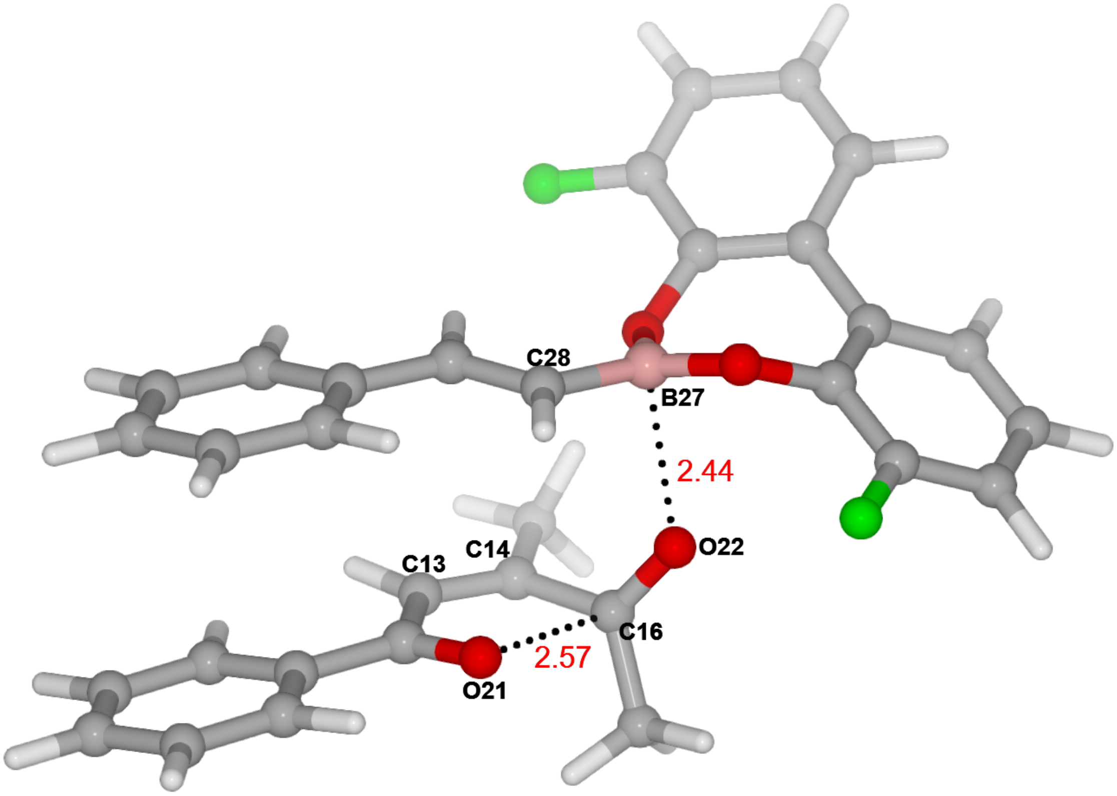

Supplementary Figure 42: Frontier molecular orbital overlap between HOMO (red/blue) and LUMO (purple/gold) orbitals.

Electronic Energy = -1760.156454 Hartrees

HOMO-LUMO gap = 3.82578349165012 eV

NBO Donor	NBO Acceptor	Stabilization Energy (kcal/mol)
40. LP (1) O 21	165. BD*(2) C 16- O 22	1.12
41. LP (2) O 21	165. BD*(2) C 16- O 22	6.51

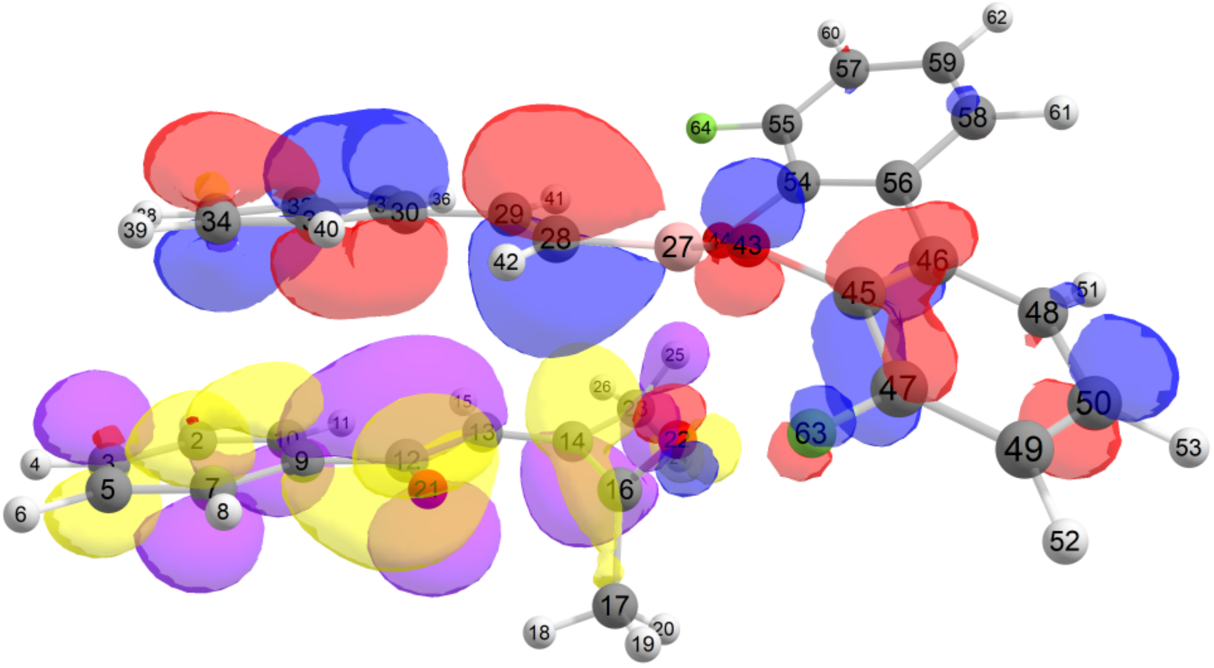

Supplementary Figure 42: MeCO-bound-LA/**Ia** intermediate, Minor enantiomer, UP.

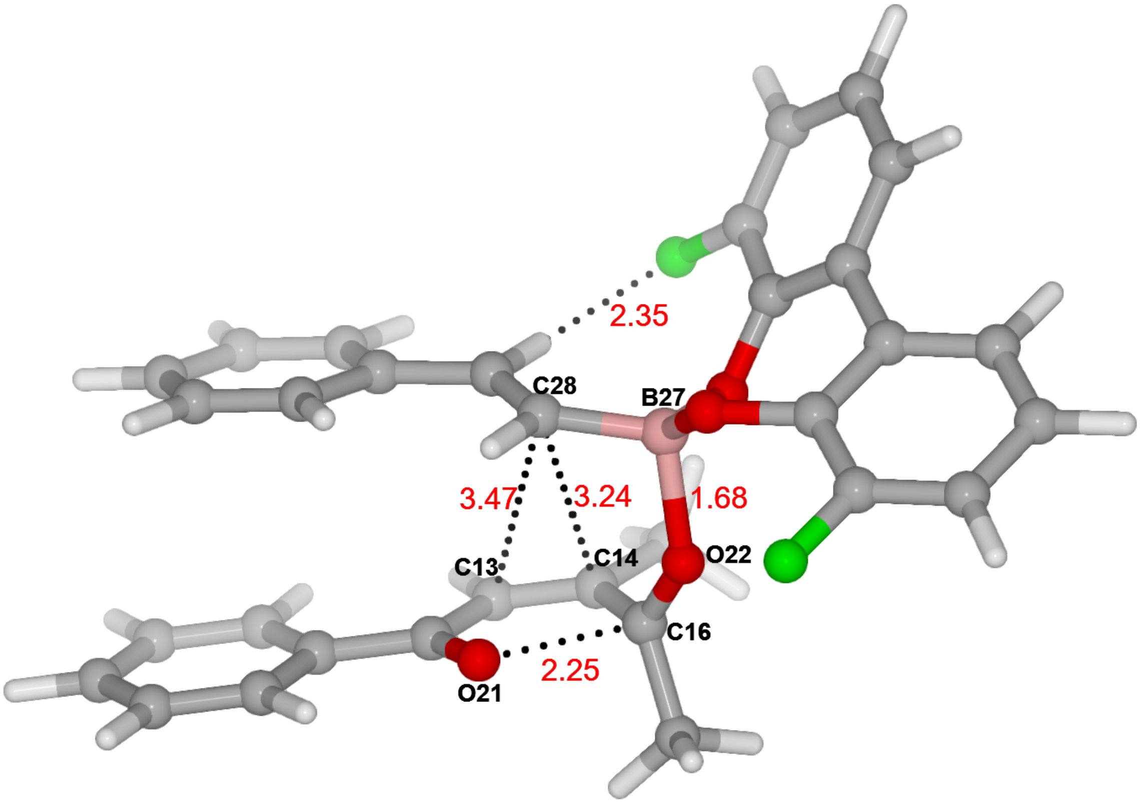

Supplementary Figure 43: Frontier molecular orbital overlap between HOMO (red/ blue) and LUMO (purple/gold) orbitals.

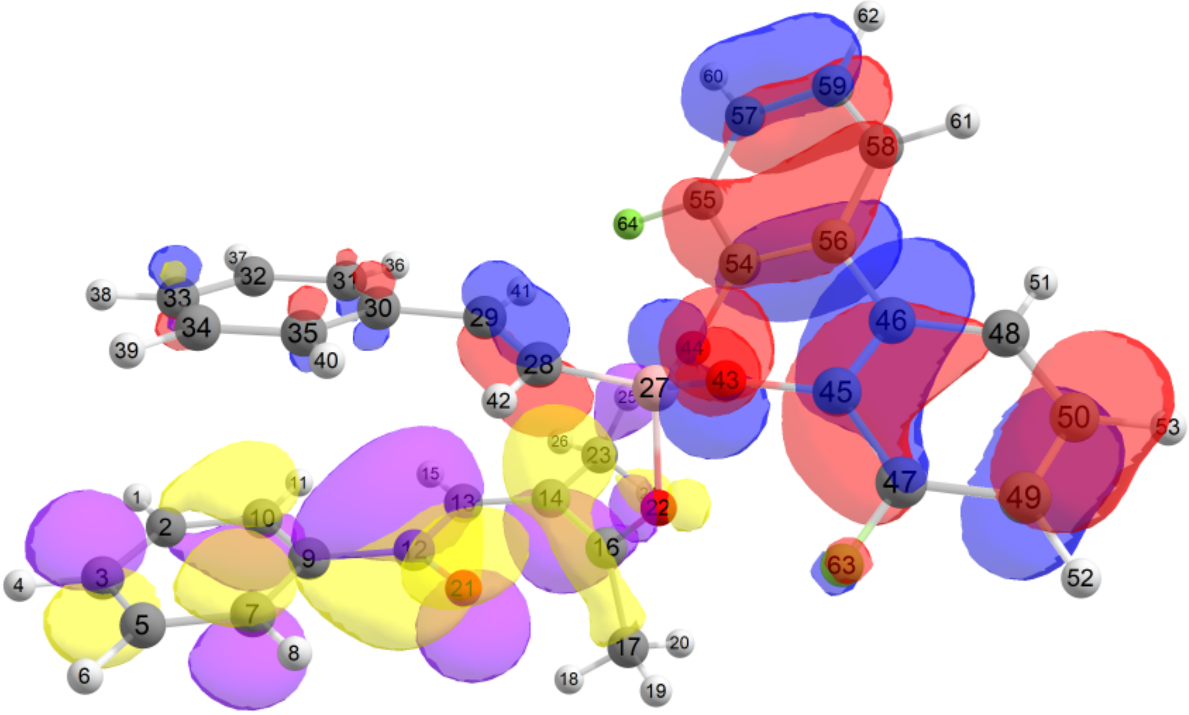

Supplementary References:

1. For **4**, see: Shih, J.-L.; Nguyen, T. S.; May, J. A. *Angew. Chem., Int. Ed.* **2015**, *54*, 9931–9935.
For **4b**, see: Han, Y.-Q.; Ding, Y.; Zhou, T.; Yan, S.-Y.; Song, H.; Shi, B.-F. *J. Am. Chem. Soc.* **2019**, *141*, 4558−4563.
For **4c**, see: Storer, R. I.; Carrera, D. E.; Ni, Y.; MacMillan, D. W. C. *J. Am. Chem. Soc.* **2006**, *128*, 84–86.
2. Yamashita, Y.; Ishitani, H.; Shimizu, H.; Kobayashi, S. *J. Am. Chem. Soc.* **2002**, *124*, 3292−3302.
3. Moquist, P. N.; Kodama, T.; Schaus, S. E. *Angew. Chem. Int. Ed.* **2010**, *49*, 7096−7100.
4. Romanov-Michailidis, F.; Guénée, L.; Alexakis, A. *Org. Lett.* **2013**, *15*, 5890−5893.
5. Yao, C.; Wu, P.; Huang, Y.; Chen, Y.; Li, L.; Li, Y.-M., *Org. Biomol. Chem.* **2020**, *18*, 9712−9725.
6. Sälinger, D.; Brückner, R. *Chem. Eur. J.* **2009**, *15*, 6688−6703.
7. Chen, L.; Du, Y.; Zeng, X.-P.; Shi, T.-D.; Zhou, F.; Zhou, J. *Org. Lett.* **2015**, *17*, 1557−1560.
8. Williams, P. D.; LeGoff, E. *Tetrahedron Lett.* **1985**, *26*, 1367−1370.
9. Blanc, A.; Tenbrink, K.; Weibel, J.-M.; Pale, P. *J. Org. Chem*. **2009**, *74*, 5342−5348.
10. (a) Becke, A. D. *J. Phys. Chem.* **1993**, *98*, 5648−5652. (b) Lee, C.; Yang, W.; Parr, R.G. *Phys. Rev. B* 1988, *37,* 785−789. (c) Vosko, S. H.; Wilk, L.; Nusair, M. *Can. J. Phys.* **1980**, *58*, 1200−1211. (d) Stephens, P. J.; Devlin, F. J.; Chabalowski, C. F.; Frisch, M. J. *J. Phys. Chem.* **1994**, *98*, 11623−11627.
11. Gaussian 16, Revision C.01, Frisch, M. J.; Trucks, G. W.; Schlegel, H. B.; Scuseria, G. E.; Robb, M. A.; Cheeseman, J. R.; Scalmani, G.; Barone, V.; Petersson, G. A.; Nakatsuji, H.; Li, X.; Caricato, M.; Marenich, A. V.; Bloino, J.; Janesko, B. G.; Gomperts, R.; Mennucci, B.; Hratchian, H. P.; Ortiz, J. V.; Izmaylov, A. F.; Sonnenberg, J. L.; Williams-Young, D.; Ding, F.; Lipparini, F.; Egidi, F.; Goings, J.; Peng, B.; Petrone, A.; Henderson, T.; Ranasinghe, D.; Zakrzewski, V. G.; Gao, J.; Rega, N.; Zheng, G.; Liang, W.; Hada, M.; Ehara, M.; Toyota, K.; Fukuda, R.; Hasegawa, J.; Ishida, M.; Nakajima, T.; Honda, Y.; Kitao, O.; Nakai, H.; Vreven, T.; Throssell, K.; Montgomery, J. A., Jr.; Peralta, J. E.; Ogliaro, F.; Bearpark, M. J.; Heyd, J. J.; Brothers, E. N.; Kudin, K. N.; Staroverov, V. N.; Keith, T. A.; Kobayashi, R.; Normand, J.; Raghavachari, K.; Rendell, A. P.; Burant, J. C.; Iyengar, S. S.; Tomasi, J.; Cossi, M.; Millam, J. M.; Klene, M.; Adamo, C.; Cammi, R.; Ochterski, J. W.; Martin, R. L.; Morokuma, K.; Farkas, O.; Foresman, J. B.; Fox, D. J. Gaussian, Inc., Wallingford CT, **2016**.
12. (a) Grimme, S.; Ehrlich, S.; Goerigk, L. *J. Comput. Chem*. **2011**, *32*, 1456–1465. (b) Grimme, S.; Antony, J.; Ehrlich, S.; Krieg, H. A. *J. Chem. Phys*. **2010** *132*, 154104.
13. Pedretti, A.; Mazzolari, A.; Gervasoni, S.; Fumagalli, L.; Vistoli, G. *Bioinformatics* **2021**, *37*, 1174-1175.
14. Barone, V.; Cossi, M. *J. Phys. Chem*. **1998** *102*, 1995−2001.
15. NBO 7.0. Glendening, E. D.; Badenhoop, J. K.; Reed, A. E.; Carpenter, J. E.; Bohmann, J. A.; Morales, C. M.; Karafiloglou, P.; Landis, C. R.; Weinhold, F. Theoretical Chemistry Institute, University of Wisconsin, Madison, WI (2018)
16. CYLview20; Legault, C. Y., Université de Sherbrooke, 2020 (http://www.cylview.org)
17. Chemcraft - graphical software for visualization of quantum chemistry computations. Version 1.8, build 654. https://www.chemcraftprog.com
